# Yiai Fuzheng decoction inhibits triple-negative breast cancer by remodeling the immune microenvironment

**DOI:** 10.3389/fimmu.2025.1615631

**Published:** 2025-09-30

**Authors:** Chaochao Yu, Chengqian Jia, Guopeng Chen, Yi Li, Yixin Liu, Yingwen Zhang

**Affiliations:** ^1^ Department of Rehabilitation, Union Hospital, Tongji Medical College, Huazhong University of Science and Technology, Wuhan, Hubei, China; ^2^ Department of Pain, The Central Hospital of Wuhan, Tongji Medical College, Huazhong University of Science and Technology, Wuhan, Hubei, China; ^3^ Department of Hematology, Zhongnan Hospital of Wuhan University, Wuhan University, Wuhan, Hubei, China; ^4^ Department of Medical Oncology, First Affiliated Hospital of Nanchang University, Nanchang, Jiangxi, China; ^5^ Department of Radiation and Medical Oncology, Zhongnan Hospital of Wuhan University, Wuhan University, Wuhan, Hubei, China; ^6^ Department of Integrated Chinese and Western Medicine, Zhongnan Hospital of Wuhan University, Wuhan University, Wuhan, Hubei, China

**Keywords:** Yiai Fuzheng decoction, triple-negative breast cancer, tumor microenvironment, transcriptomic profiling, metabonomic profiling

## Abstract

**Objective:**

This study aimed to examine the potential anticancer properties of Yiai Fuzheng decoction (YFD), along with its mechanism of action against triple-negative breast cancer (TNBC).

**Methods:**

A TNBC mouse model was established by inoculating 4T1 cells into the 4th mammary fat pad. Micropositron emission tomography (micro-PET), hematoxylin and eosin (HE) staining, immunohistochemistry, immunofluorescence assays, flow cytometry, and western blotting were used to assess the therapeutic effects of YFD. The components of YFD were identified via UHPLC-Q/Orbitrap MS. Nontargeted metabolomic analysis was performed to identify changes in tumor metabolites via gas chromatography-t*ime-of-flight mass* spectrometry (GC-TOF/MS). The Illumina sequencing platform was used to identify differentially expressed genes in the tumors.

**Results:**

A total of 20 bioactive components of YFD were screened and identified. We found that YFD treatment resulted in a substantial increase in CD4^+^ and CD8^+^ T cells, a reduction in myeloid-derived suppressor cells (MDSCs) and tumor-associated macrophages (TAMs), and an increase in the M1/M2 ratio of TAMs in tumors. These changes create a tumor-suppressive microenvironment that inhibits tumor growth and metastasis in TNBC mice. YFD can affect various immune regulatory pathways, such as inactivation of the mitogen-activated protein kinase kinase/extracellular signal-regulated kinase 1 and 2 (MEK/ERK1/2) pathway. Additionally, metabolomic analysis suggested that YFD could reprogram several altered metabolic pathways, including the urea cycle; metabolism of arginine and proline; pyruvate; the Warburg effect; D-arginine; and D-ornithine, glutamate, glycine, serine, and tryptophan, to suppress cancer progression.

**Conclusion:**

Our findings provide preclinical evidence that supports the application of YFD in TNBC treatment.

## Introduction

Breast cancer (BC) is the most frequently diagnosed cancer in women worldwide. In 2022, 287,850 new BC cases and 43,250 fatalities from BC occurred in the United States alone (1). A recent investigation revealed that BC surpassed lung cancer as the most common cancer globally (2). In 2020, there were more than 2 million new cases, accounting for 11.7% of all cancer cases, and 684,996 new fatalities, accounting for 6.9% of all cancer-related deaths (2). BC is highly heterogeneous with varying genetic profiles and histopathological changes. These subtypes are divided into luminal A, luminal B, human epidermal growth factor receptor 2 (HER2)-enriched, and triple-negative breast cancer (TNBC) subtypes (3). TNBC has unusual molecular characteristics, as it does not express any of the three major receptors: estrogen, progesterone, or HER2. It is aggressive and tends to spread to other areas of the body, such as the lungs, brain, and bones (4). Patients with TNBC have a poor prognosis and a high recurrence rate (5). TNBC is not sensitive to molecular-targeted or endocrine therapy (3). Currently, chemotherapy is the principal therapeutic option for TNBC (6). Currently, approved chemotherapeutics, such as taxanes and anthracyclines, have shown less satisfactory efficacy in TNBC owing to the heterogeneity and development of chemoresistance (7). Therefore, identifying an effective therapy that can slow disease progression and improve patient survival is crucial.

The tumor microenvironment (TME) plays a pivotal role in the malignant progression and therapeutic response of BC (8). It is composed of various components, including cancer cells, cancer stem cells, tumor-associated macrophages (TAMs), myeloid-derived suppressor cells (MDSCs), lymphocytes, natural killer cells, cancer-associated fibroblasts, the extracellular matrix, cytokines, and growth factors (9). The exponential proliferation of BC cells induces a highly hypoxic environment, which results in metabolic reprogramming of BC cells, immune cells, and other surrounding TME cells, thus driving tumor growth, angiogenesis, stemness, metastasis, and therapeutic resistance (10). Consequently, TME remodeling could be a promising method for treating TNBC (10). MDSCs are the predominant immunosuppressive cells in the TME (11). MDSCs are immature myeloid cells that can produce immunosuppressive cells, such as regulatory T cells and T helper 17 cells, and limit T-cell proliferation and activation (12). TAMs are the major types of tumor-infiltrating immune cells (13). They are divided into activated M1-like TAMs, which have antitumor effects, and activated M2-like TAMs, which promote cancer growth (14). The accumulation of MDSCs and TAMs can suppress antitumor immunity and contribute to BC progression (15, 16). Additionally, clinical studies have shown that an increased population of MDSCs or TAMs is associated with metastasis and decreased survival in patients with BC (17, 18). Thus, targeting MDSCs or TAMs to remodel the TME may be an encouraging approach for BC immunotherapy (12, 19).

An increasing body of evidence suggests that traditional Chinese herbal medicines and ingredients originating from medicinal plants have significant potential as adjuvant treatments for BC (20–24). Furthermore, studies have revealed that traditional Chinese medicine (TCM) can slow cancer growth by modifying the TME (25, 26). According to Li et al., the Chinese medicine decoction Aiduqing inhibits TAM/CXCL1-induced Treg differentiation and infiltration, thereby dramatically suppressing cancer growth and lung metastasis (27). Wang et al. demonstrated that the classical Chinese medicine formula Yu-Ping-Feng significantly extended the survival of mice with Lewis lung cancer by activating M1 macrophage polarization and increasing CD4^+^ T-cell cytotoxicity (28). However, there are few reports of effective Chinese herbal decoctions that can remodel the TME to prevent TNBC progression.

Yiai Fengzheng decoction (YFD) is a custom-made compound formula developed by Prof. Yingwen Zhang on the basis of TCM theory and long-term clinical experience. YFD can effectively decrease BC-related fatigue, reduce the incidence of cancer recurrence, prolong survival, and treat chemotherapy-related adverse reactions (29). Additionally, the YFD successfully obtained invention patent certification (30). Therefore, uncovering the underlying mechanisms by which YFD prevents BC progression and providing compelling data to support its wider therapeutic application are worthwhile. In recent years, omics research techniques, such as transcriptomics, metabolomics, proteomics, and phenomics, have been increasingly used to elucidate the biological mechanisms of TCM prescriptions for the treatment of diverse ailments from a systematic and holistic perspective (31, 32). Owing to the intricate pathophysiology and evolution of TNBC, multiomic profiling may be more appropriate for understanding the landscape of the TNBC microenvironment (33, 34). Accordingly, it is reasonable to assume that multiomic techniques may be more useful for understanding the mechanism of the antitumor actions of YFD and for identifying potential biomarkers for prognosis and treatment. In this study, multiomics technologies, including transcriptomic and metabolomic profiling, were used to elucidate the mechanisms through which YFD reshaped the TME in TNBC. UHPLC-Q/Orbitrap MS was used to identify bioactive ingredients in YFD. This study not only identified the bioactive components of YFD but also elucidated its anti-BC mechanisms from the perspective of systematic biology and TME remodeling, which have not been sufficiently reported.

**Figure 1:**
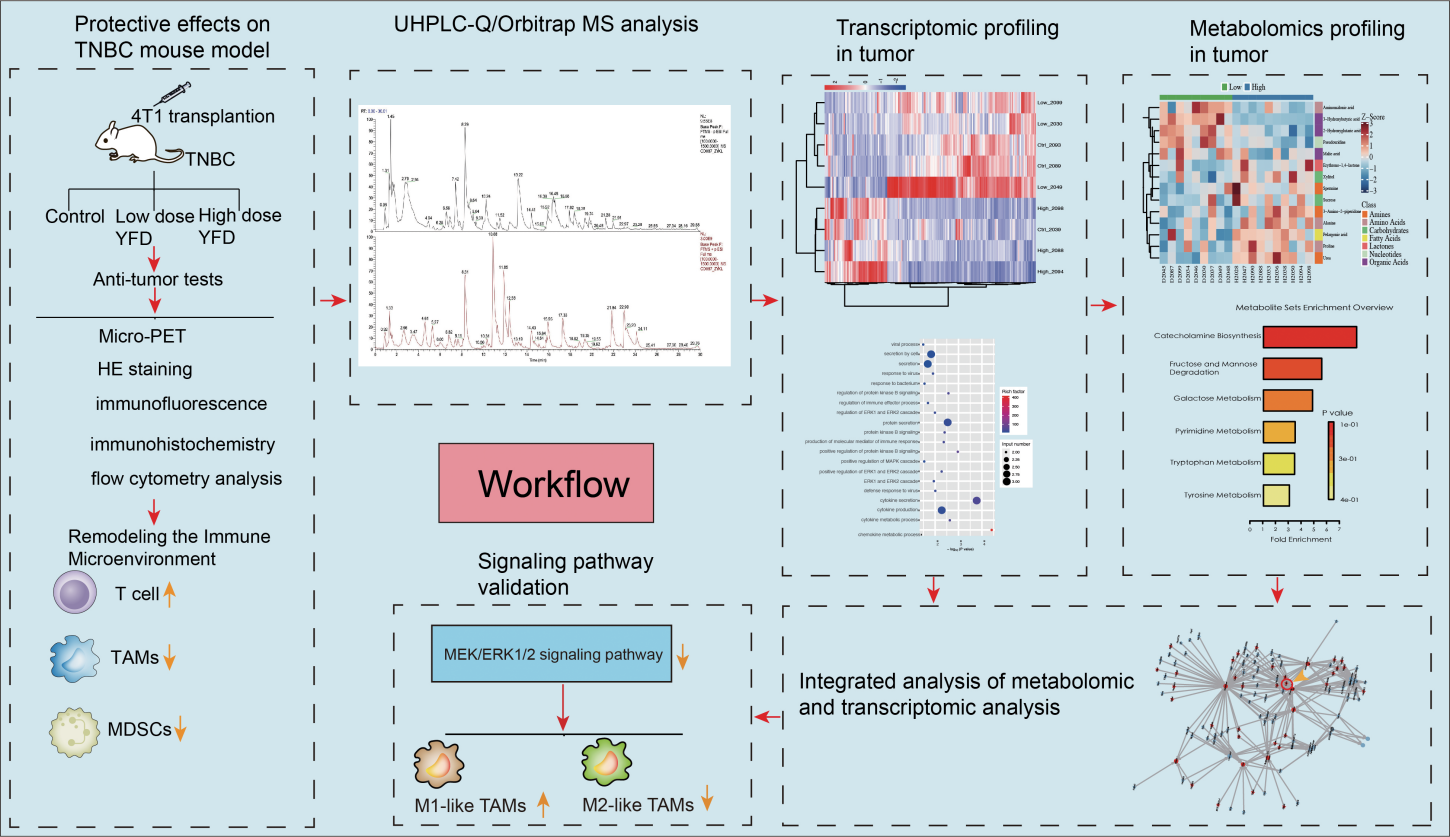
Workflow of the present study.

The present study pioneers the role of YFD in MEK/ERK1/2 signaling-mediated immune microenvironment remodeling, metabolome-driven TAM polarization and MDSC inhibition. This study provides convincing experimental evidence supporting the application of YFD in treatment. The workflow is illustrated in **Figure 1**.

## Materials and methods

### Preparation of YFD decoction

All of the herbs were prepared by the pharmacy of Zhongnan Hospital of Wuhan University. All herbal components of YFD were purchased from Hubei Chenmei Chinese Traditional Medicine Co., Ltd. (Huanggang, China).The herbal material consisted of 15 medicinal herbs: 15 g *Huang Qi* (root of *Astragalus membranaceus*), 15 g *Fu Ling* (dried sclerotium of *Poria cocos*), 12 g *Shen Jin Cao* (whole dried *Lycopodium japonicum Thunb*), 15 g *Si Gua Luo* (vascular bundle of *Luffa cylindrica Roem*), 12 g *Kun Bu* (thallus of *Laminaria japonica Aresch*), 15 g *Zhe Bei Mu* (dry bulb part of *Fritillaria thunbergii Miq*), 15 g *San Leng* (dry tuber part of *Sparganium stoloniferun Buch*), 6 g *Shui Zhi* (whole dried body of *Whitmania pigra Whitman*), 15 g *Yu Jin* (tuberous root of *Curcuma longa L*), 15 g *Xia Ku Cao* (dry fruit cluster of *Prunella vulgaris L*), 15 g *Bai Hua She She Cao* (whole part of *Oldenlandia diffusa*), 15 g *E Zhu* (dry tuberous root of *Curcuma phaeocaulis Valeton*), 15 g *Pu Gong Ying* (whole part of dried *Taraxacum mongolicum Hand*), 15 g *Zao Jiao Ci* (dry caltrop of *Gleditsia sinensis Linn*), and 12 g *Hong Teng* (dry rattan of *Sargentodoxa cuneata*). All the herbs were soaked and washed in a 6-fold volume of water for half an hour, followed by boiling for 30 min. The final concentration of the herbal medicine solution was 4.4 g/ml. Finally, the decoction was stored at 4°C.

### UHPLC-Q/orbitrap MS analysis of YFD

The freeze-dried YFD formulation (drug concentration: 6.37 g/g) was reconstituted in 30 mL of heated deionized water. A 100 μL aliquot of this mixture was subsequently mixed with 400 μL of methanol and vortexed for 10 min. After centrifugation (4°C, 13,000 ×g, 10 min), the resulting mixture was filtered and subjected to chromatographic analysis via an LC–MS system. The detailed LC–MS parameters are provided in **Table 1** and **Table 2**. High-resolution LC–MS/MS datasets were computationally processed via Compound Discoverer 3.3 (CD 3.3) with reference to the McCloud metabolomic database for compound annotation.

**Table 1:** The mass spectrometry conditions.

Condition items	Parameters
ion source	electrospray ionization (ESI)
scan method	switching between positive and negative ion modes
detection method	full mass/dd-MS_2_
resolution	70000 (full mass) and 17500 (dd-MS2)
scan range	100.0-1500.0 m/z
spray voltage	3.2 kV (positive, negative)
capillary temperature	300°C
collision gas	high-purity argon gas (≥99.999% purity)
collision energy (N)CE	30, 40, 60
sheath gas	nitrogen (≥99.999% purity) at 40 Arb
auxiliary gas	nitrogen (≥99.999% purity) at 15Arb and 350 °C
data acquisition time	30 min

**Table 2:** The liquid chromatography conditions.

Condition items	Parameters
chromatographic column	AQ-C18, 150 × 2.1 mm, 1.8 μm, Welch
flow velocity	0.30 mL/min
aqueous phase	0.1% of formic acid in water
organic phase	methanol
column oven temperature	35°C
autosampler temperature	10°C
sample injection volume	5 μL

### Ethics statement

All experimental procedures involving animals were approved by the Institutional Animal Ethical Review Board of Zhongnan Hospital, Wuhan University (Approval ID: ZN2022059). This study strictly complied with Wuhan University’s institutional guidelines for laboratory animal care and utilization throughout the experimental protocol.

### Animal experiments

Female C57BL/6 mice (8 weeks old) were procured from SPF Biotechnology Co., Ltd. (Beijing; Certification: SCXK[Jing]2019-0010) and maintained under controlled environmental conditions (12-hour photocycle, 20 – 22°C, 30 – 70% relative humidity; Facility License: 110324220104570773SCXK-2020–100). After a 7-day acclimation period, TNBC models were surgically established via the orthotopic implantation of 1×10^4^ 4T1 cells into the fourth mammary fat pad. The tumor-bearing mice were randomized into three groups (n=10/group): the control, low-dose (YFD^low^), and high-dose (YFD^high^) groups. Dosages were calculated via interspecies dose translation (animal equivalent dose = human dose × 12.3 km ratio) (35). YFD solutions (11.07 g/kg (YFD^low^) and 44.28 g/kg (YFD^high^)) were administered intragastrically as previously reported. Our previous *in vitro* cytotoxicity tests demonstrated that different concentrations ranging from 15 – 60 mg/ml had few harmful effects (36). Biometric parameters (body mass and tumor dimensions) were recorded triweekly, and the tumor volume was calculated as 0.5×length×width². Pharmacological intervention commenced upon confirmed tumor engraftment (days 7 – 9 postimplantation), which consisted of daily oral gavage for 14 consecutive days.

### Micropositron emission tomography imaging

A TransPE BioCaliburn LH instrument (RAYCAN, Suzhou, China) was used to performed the microPET scans. After anesthesia with 2% isoflurane, the mice were placed in the prone position, and 18F**-** *fluorodeoxyglucose* (FDG) was injected into the mice via the tail vein. Scanning was started 50 min after the injection. Each mouse was scanned for 30 min. Next, the microPET data were reconstructed via a 3D ordered subset expectation-maximum (OSEM) algorithm. The mean standardized uptake values (SUVs) were calculated via region-of-interest (ROI) analysis.

### Sample collection and preparation

Following microPET imaging, tumor-bearing mice were anesthetized via inhalation of 2% isoflurane, followed by immediate procurement of the peripheral blood serum. Splenic, pulmonary, and neoplastic tissues were subjected to cryogenic storage (0 – 4°C). The harvested serum and splenic samples were processed for immunophenotypic profiling via flow cytometry. Concurrently, pulmonary and tumor tissue aliquots were flash-frozen in liquid nitrogen vapor for cryopreservation at -80°C, while residual tissue segments were immersed in a 4% paraformaldehyde (PFA) solution.

### Flow cytometry analysis

First, we separated the peripheral blood lymphocytes and splenocytes. Next, the erythrocytes were subsequently lysed. Then, we incubated the obtained cell suspensions with anti-mouse CD16/32. To identify different subtypes of T cells, we stained the cells with PE-conjugated anti-CD8, FITC-conjugated anti-CD4, and APC-conjugated anti-CD3 antibodies. A flow cytometer was used to detect the labeled cells. FlowJo software (Tree Star Inc., Ashland, OR, USA) was used to calculate the number of labeled cells.

### Hematoxylin and eosin staining and immunohistochemistry

Lung and tumor tissues were fixed in 4% paraformaldehyde for 24 h. The fixed tissues were dehydrated and embedded in paraffin. The embedded sections were cut into 5 µm thick slices. HE staining was used to detect metastatic nodules in the lungs, and a BX53 microscope was used to determine the area of the metastatic lesions (Olympus, Center Valley, PA, USA). Immunohistochemical analysis was performed to validate the effect of YFD on metastatic potential. The samples were subjected to antigen retrieval by heating in sodium citrate buffer, followed by endogenous peroxidase blocking. The sections were then incubated with N-cadherin (1:500, GB12135; Servicebio, Wuhan, China), vimentin (1:500, GB11192; Servicebio), and Ki67 (1:500, GB111141; Servicebio) antibodies at 4°C overnight. The slides were then incubated with a suitable secondary antibody for one hour at 37°C. Immunostaining was performed by incubation with diaminobenzidine and counterstaining with hematoxylin. Finally, a BX53 microscope was used to observe the staining results. ImageJ software (National Institutes of Health, Bethesda, MD, USA) was used for data analysis.

### Immunofluorescence assay

The tumor tissue sections were subjected to an antigen retrieval procedure by heating in sodium citrate buffer and blocked for 1 h with 10% goat serum. To detect TAMs, the slides were incubated with an anti-F4/80 antibody (1:200, sc-377009, Santa Cruz Biotechnology, Santa Cruz, CA, USA) overnight at 4°C. The sections were then washed with PBS and incubated with secondary antibody (1:500, ab150116, Abcam, Inc., USA) for 1 h. Next, the slides were washed with PBS 4 times (5 min/wash) and blocked for 1 h with 10% goat serum. The slides were subsequently incubated with an anti-CD11b antibody (1:500, ab184308, Abcam, Inc., USA) at 4°C overnight. The cells were incubated with a secondary antibody (1:500, ab150116, Abcam, Inc., USA) for 1 h. After several washes with PBS, the nuclei were stained with DAPI. The procedure used to detect MDSCs was similar to that described above, but the labeling antibodies used were anti-Ly6G antibody (1:300, sc-53515, Santa Cruz, CA, USA) and anti-CD11b antibody. To detect T cells, the sections were incubated with an anti-CD3 antibody (1:300, sc-20047, Santa Cruz, CA, USA) and secondary antibodies (1:500, ab150116, Abcam, Inc., USA). To detect M1-type TAMs, the slides were incubated with anti-F4/80 and anti-CD86 antibodies (1:400, sc-28347, Santa Cruz, CA, USA). To detect M2-type TAMs, the slides were incubated with anti-F4/80 and anti-CD206 antibodies (1:300, sc-58986, Santa Cruz, CA, USA). The appropriate secondary antibodies were selected on the basis of the reactivity of the primary antibodies. A laser confocal fluorescence microscope (STELLARIS 5 SR, Leica, Mannheim, Germany) was used to observe the immunofluorescence results.

### Western blot analysis

Tumor tissues from three mice in each group were randomly selected for western blot analysis. First, 10% SDS–PAGE gels were used to separate equal amounts of the loaded proteins. The isolated proteins were transferred onto polyvinylidene fluoride membranes. Next, the membranes were incubated overnight at 4 °C with the following primary antibodies: MEK-1/2 mouse mAb (1:500, sc-81504, Santa Cruz, CA, USA), phospho-MEK1/MEK2-S217/S221 rabbit mAb (1:500, AP1349, ABclonal, Wuhan, China), ERK1+ERK2 rabbit mAb (1:10000, ab184699, Abcam, Inc., USA), phospho-ERK1-T202/Y204+ERK2-T185/Y187 rabbit pAb (1:500, AP0472, ABclonal, Wuhan, China), LAD1 Rabbit pAb (1:500, AP17506, ABclonal, Wuhan, China), and TNFα mouse mAb (1:500, sc-52746, Santa Cruz, CA, USA).), IL-10 mouse mAb (1:500, sc-365858; Santa Cruz Biotechnology, CA, USA), and GAPDH (1:5000, HRP-60004; Proteintech, Wuhan, China). After incubation with the appropriate secondary antibodies, protein signals were detected via a ChemiDocXRS+Imaging System (Tanon-5200, Tanon, Shanghai, China.) and quantified via ImageJ software.

### RNA extraction and sequencing

TRIzol was used to separate total RNA from the tumor tissues. After RNA was extracted by DNaseI, the DNA digestion procedure was performed. The RNA quality was then assessed via a NanodropTM OneC spectrophotometer (Thermo Fisher Scientific, Inc., MA, USA). Subsequently, 1.5% agarose gel electrophoresis was performed to assess RNA integrity. Additionally, qualifying RNAs were quantified via a Qubit 3.0 instrument with a QubitTM RNA Broad Range Assay Kit. A stranded RNA sequencing library was created with 2 µg of total RNA via the KCTM Stranded mRNA Library Prep Kit for Illumina^®^ (catalog no. DR08402; Wuhan SeqHealth Co., Ltd. Wuhan, China). PCR products in the range of 200 – 500 bp were isolated and sequenced via a HiSeq × 10 sequencer.

### RNA-seq data analysis

Using the STRA software and the default parameters, the acquired data were mapped to the reference genome of *Homo sapiens* (Homosapiens. GRCh38; ftp://ftp.ensembl.org/pub/release-87/fasta/homo_sapiens/dna/). Based on feature counts (Subread-1.5.1; Bioconductor), reads mapped to each gene’s exon regions were counted, and RPKMs were then computed. The edgeR program was used to identify the genes that were differentially expressed between groups. The statistical significance of variations in gene expression was assessed via a false discovery rate (FDR)-adjusted *p* value threshold of 0.05 and a fold change criterion of 2. On the basis of a corrected *p* value cutoff of 0.05, to determine statistically significant enrichment, KOBAS software was used to perform gene ontology (GO) and KEGG enrichment analyses for the DEGs. Alternative splicing events were identified via rMATS with an FDR value threshold of 0.05 and an absolute value of 0.05.

### Metabolomic analysis

Untargeted metabolomic analysis across the control, low-, and high-dose cohorts was conducted via the XploreMET platform. Specifically, 50 mg of tumor samples from each group were homogenized with 25 mg of precooled zirconium oxide beads supplemented with 10 μL of DL-chlorophenylalanine (internal standard). This quality control marker was introduced prior to metabolite extraction to systematically quantify the technical variations arising from sample preparation and instrumental analysis. The overall coefficient of variation (CV), defined as the ratio of the standard deviation to the mean peak signal intensity, was assumed to be within 20% for each analytical block of 180 samples. After homogenization with 50% precooled methanol and centrifugation at 14000 rpm and 4°C for 20 min, the mixture was homogenized in 175 μL of precooled methanol/chloroform (v:v=3:1) for one more round of extraction, followed by centrifugation at 14000 rpm and 4°C for 20 min. The chloroform in the remaining supernatant was removed and lyophilized via a FreeZone freeze-dryer. A robotic multipurpose MPS2 sample with dual heads was used for sample derivatization and injection. Specifically, the dried sample was derivatized with 50 μL of methoxyamine (20 mg/mL in pyridine) at 30°C for 2 h, followed by the addition of 50 μL of MSTFA (1% TMCS) at 37.5°C for another 1 h via the sample preparation head. After derivatization, the samples were injected via a sample injection head. Quality control was conducted to ensure repeatability and stability.

A gas chromatography-t*ime-of-flight mass* spectrometry (GC-TOF/MS) machine (Pegasus HT, Leco Corp., St. Joseph, MO, USA) with an Agilent 7890 B gas chromatograph and a Gerstel multipurpose pattern MPS2 with dual heads (Gerstel, Muehlheim, Germany) was used for untargeted metabolic profiling. The parameters were set as follows: column, DB-5MS (5% diphenyl/95% dimethyl polysiloxane) 30 m (length) × 250 μm I.D., 0.25-μm film thickness; oven programmed temperature, 80°C (2 minutes), 80 – 300°C (12°C/min), 300°C (8 minutes); inlet temperature, 270°C; injection volume, 1.0 μL (splitless); carrier gas, helium (99.9999%); transfer interface temperature, 270°C; flow rate, 1.0 mL/min; ionization mode electron energy, 70 EV; detector voltage, -1700 V; source temperature, 220°C; acquisition rate, 25 spectra/sec; and mass range, 50 – 500 Da.

ChromaTOF (v4.71, Leco Corp., St. Joseph, MO, USA) was used to process the raw data obtained by GC-TOF/MS. Metabolites were identified by comparison with the JiaLib metabolite database. Principal component analysis (PCA), projection to latent structure discriminant analysis (PLS-DA), and orthogonal PLS-DA (OPLS-DA) were used. The importance of variables in the projection (VIP) of each identified metabolite was subsequently calculated. Metabolites with VIP > 1, *p*< 0.05, and |log_2_fold change (FC)|≥0 were considered differentially abundant metabolites. Pathway-associated metabolite sets were used for pathway enrichment analysis. The KEGG database was used to conduct functional annotation and enrichment analyses of the differentially expressed metabolites.

### Statistical analysis

The results are expressed as the means ± standard deviations (means ± SDs). The data were subjected to one-way analysis of variance via GraphPad software (version 8), followed by either Dunnett’s t test or Tukey’s test. Statistical significance was defined as a p value< 0.05.

## Results

### Components of YFD

**Figure 2:**
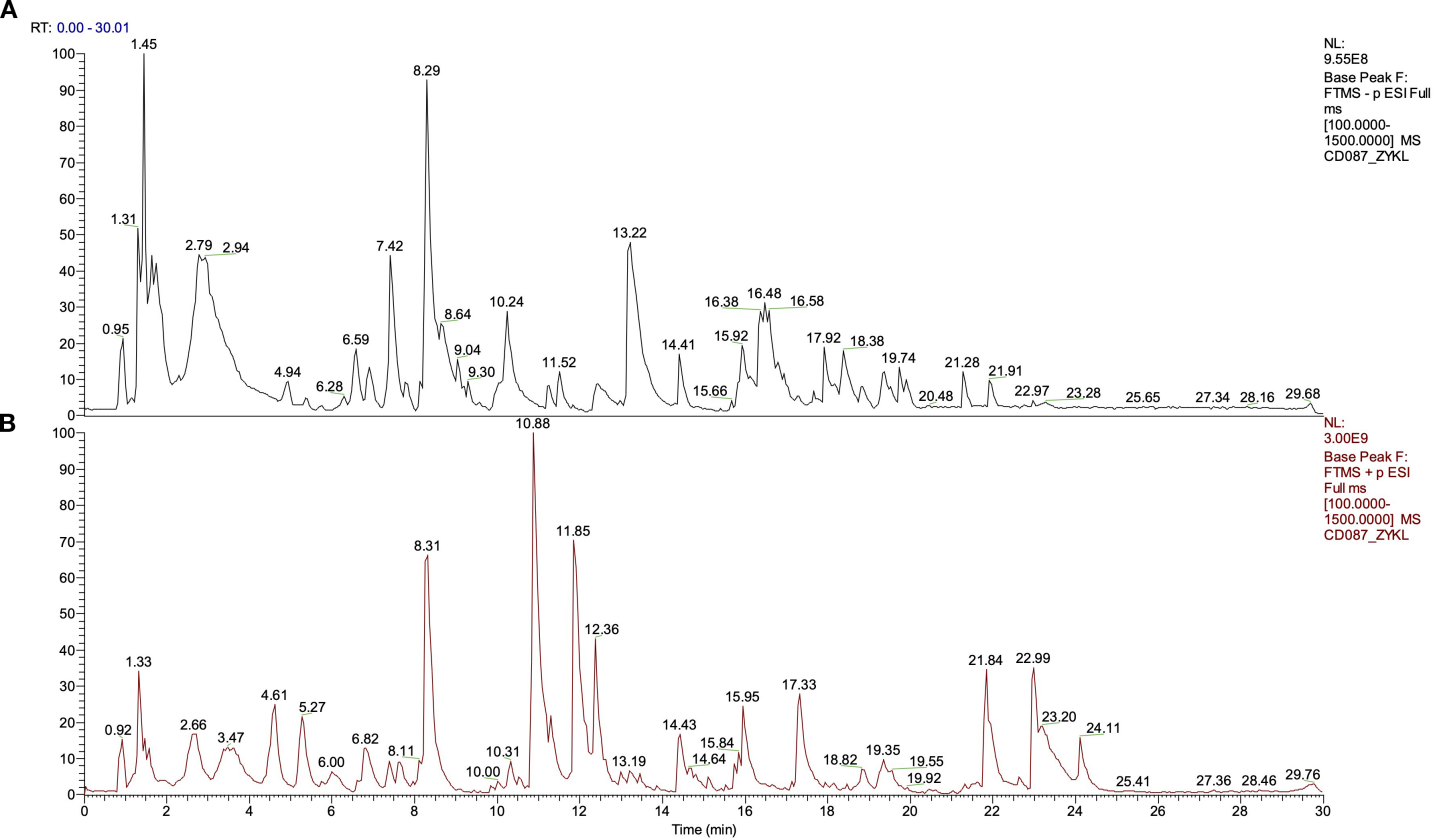
Total ion current diagram in positive and negative modes of Yiai Fengzheng decoction (YFD). **(A)** Negative ion mode of YFD. **(B)** Positive ion mode of YFD.

The phytochemical components of YFD were studied via UHPLC-Q/Orbitrap MS in positive and negative ion modes. The total ion chromatograms are shown in **Figure 2**. The bioactive ingredients in YFD were identified by comparison with standard materials and chemical information obtained from the mzCloud mass spectrometry library. The top 20 bioactive compounds were screened and identified and are presented in **Table 3**.

**Table 3:** Top 20 components of YFD.

Compound	Formula	Calculated molecular weight	Retention time(min)	mzCloud best match(%)
L-Phenylalanine	C_9_H_11_NO_2_	165.07878	5.284	100
Nicotinic acid	C_6_ H_5_ NO_2_	123.03217	2.161	100
Linoleic acid	C_18_ H_32_ O_2_	280.23985	22.052	99.9
Benzoic acid	C_7_ H_6_ O_2_	122.03545	9.719	99.9
Palmitic acid	C_16_ H_32_O_2_	256.23993	22.565	99.9
Caffeic acid	C_9_ H_8_ O_4_	180.04117	10.245	99.8
Formononetin	C_16_ H_12_ O_4_	268.0729	15.857	99.8
Apigenin 7-O-glucuronide	C_21_ H_18_ O_11_	446.08455	13.917	99.8
Salicylic acid	C_7_ H_6_ O_3_	138.03038	12.952	99.8
Catechol	C_6_H_6_O_2_	110.03258	7.366	99.7
Oleanolic acid	C_30_H_48_O_3_	456.3595	22.1	99.6
Chlorogenic	C_16_H_18_O_9_	354.09508	10.205	99.6
Rutin	C_27_H_30_O_16_	610.15256	12.982	99.5
Apigenin	C_15_H_10_O_5_	270.05238	15.774	97.9
Asiatic acid	C_30_H_48_O_5_	488.34978	19.537	97.9
Catechin	C_15_H_14_O_6_	290.07895	9.114	97.6
Eicosapentaenoic acid	C_20_H_30_O_2_	302.22389	21.748	97.3
Quercetin	C_15_H_10_O_7_	302.04216	12.987	96.5
Vanillin	C_8_H_8_O_3_	152.04724	10.577	96.4
Astragalin	C_21_H_20_O_11_	402.095	13.668	95.2

### YFD inhibited breast tumor growth and metastasis *in vivo*

**Figure 3:**
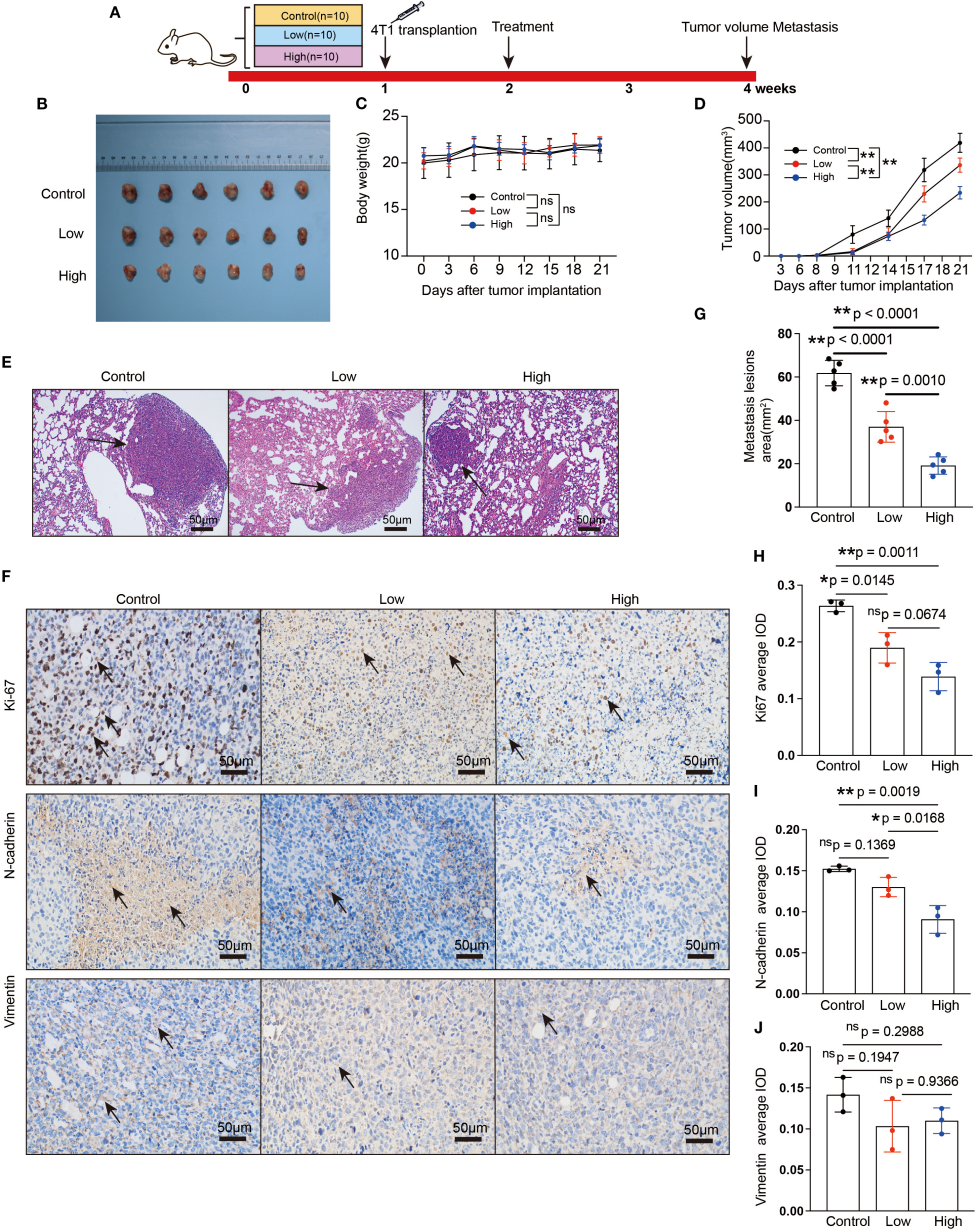
YFD inhibited breast cancer growth and metastasis *in vivo*. **(A)** Schematic flowchart of the experiment. The mice in the low- and high-dose groups received YFD orally once daily for 2 successive weeks, whereas the mice in the control group received saline. **(B)** Dissociated tumor tissues from each group. **(C, D)** Body weight changes and tumor growth curves (n=10). Weight and tumor growth were measured every 3 days. **(E)** Representative HE-stained images of lung sections from each group. The black arrows indicate metastatic lesions. **(F)** Representative immunohistochemistry images of Ki67, N-cadherin, and vimentin in tumor tissues. **(G)** Comparison of metastatic lesion areas in lung sections. **(H-J)** Expression levels of Ki67, N-cadherin, and vimentin in tumor tissues from each group. The data are expressed as the means ± SDs (n=3 for each group). *p< 0.05, **p< 0.01, nsp>0.05 compared with the intended group by ANOVA followed by Dunnett’s t *post hoc* test.

**Figure 4:**
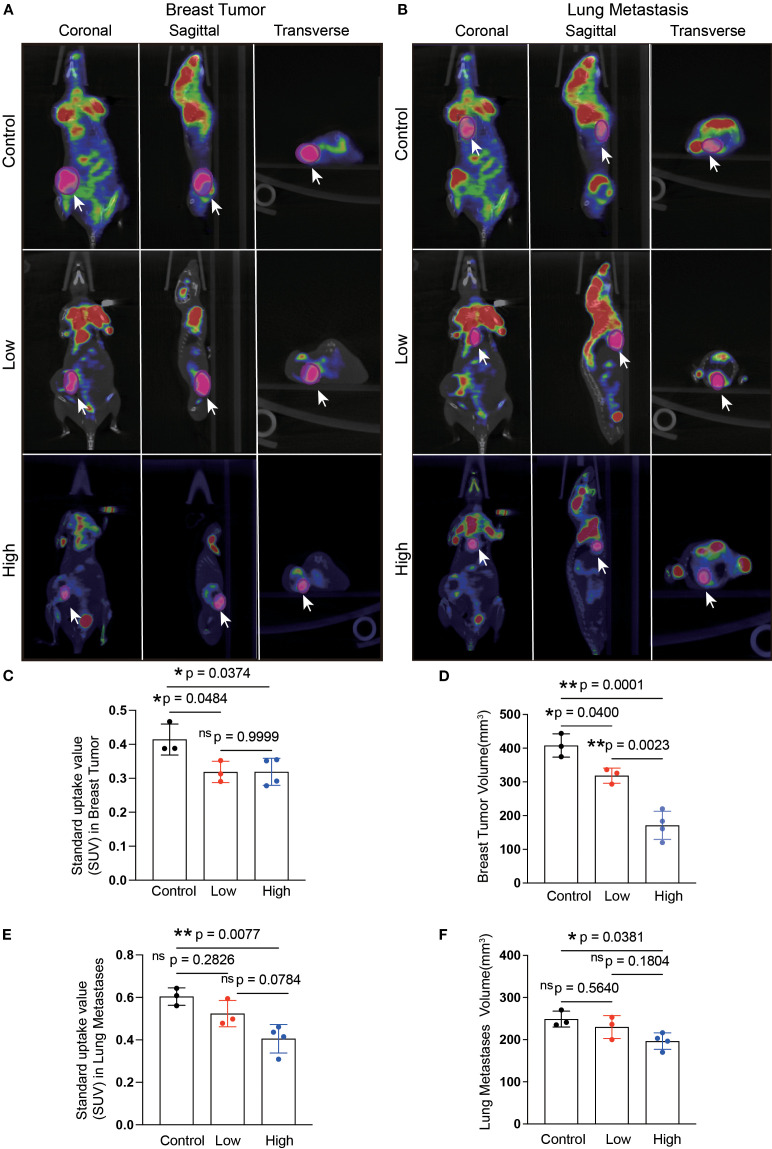
Microposition emission tomography (microPET) scanning after YFD treatment. **(A, B)** Representative microPET images of the breast tumors and lungs from each group. MicroPET scanning was used to evaluate the effects of YFD on tumor growth and lung metastasis. **(C, D)** Comparison of the calculated SUVs and breast tumor volumes among the groups. **(E, F)** Comparison of the calculated SUVs and lung metastasis volumes among the groups. The data are presented as the means ± SDs (n=3). *p< 0.05, **p< 0.01, nsp>0.05 compared with the intended group by ANOVA followed by Dunnett’s t *post hoc* test. *p< 0.05, **p< 0.01, ^ns^p>0.05 compared with the intended group by ANOVA followed by Dunnett’s t *post hoc* test. SUV, standardized uptake value.

A flowchart of the experiment is shown in **Figure 3A**. Tumor-bearing mice were orally administered YFD or saline once daily for two weeks, beginning in the second week after 4T1 breast cancer cells were inoculated. The body weights of the mice and the increase in tumor volume were measured every three days. During the treatment period, there was little significant variation in body weight among the groups (**Figure 3C**). Furthermore, the results from dynamic monitoring (**Figures 3B, D**) and microPET scanning (**Figures 4A, C, D**) revealed that YFD administration suppressed tumor development in a dose-dependent manner, with high-dose YFD treatment inhibiting tumor growth more than low-dose YFD treatment did. To assess the effects of YFD treatment on metastasis, immunohistochemistry was used to measure the levels of tumor metastasis-associated markers, including ki67, N-cadherin, and vimentin, in tumor tissues. The results showed that high-dose YFD therapy considerably decreased Ki67 and N-cadherin expression levels (**Figures 3H, I**) but had little effect on vimentin levels (**Figures 3J**). Furthermore, HE staining (**Figures 3E-G**) and microPET scanning (**Figures 4B, E, F**) revealed that YFD treatment reduced the number of metastatic lesions in the lungs. These findings strongly indicate that YFD inhibits breast tumor growth and lung metastasis.

### YFD reshaped the tumor immune microenvironment in breast cancer

**Figure 5:**
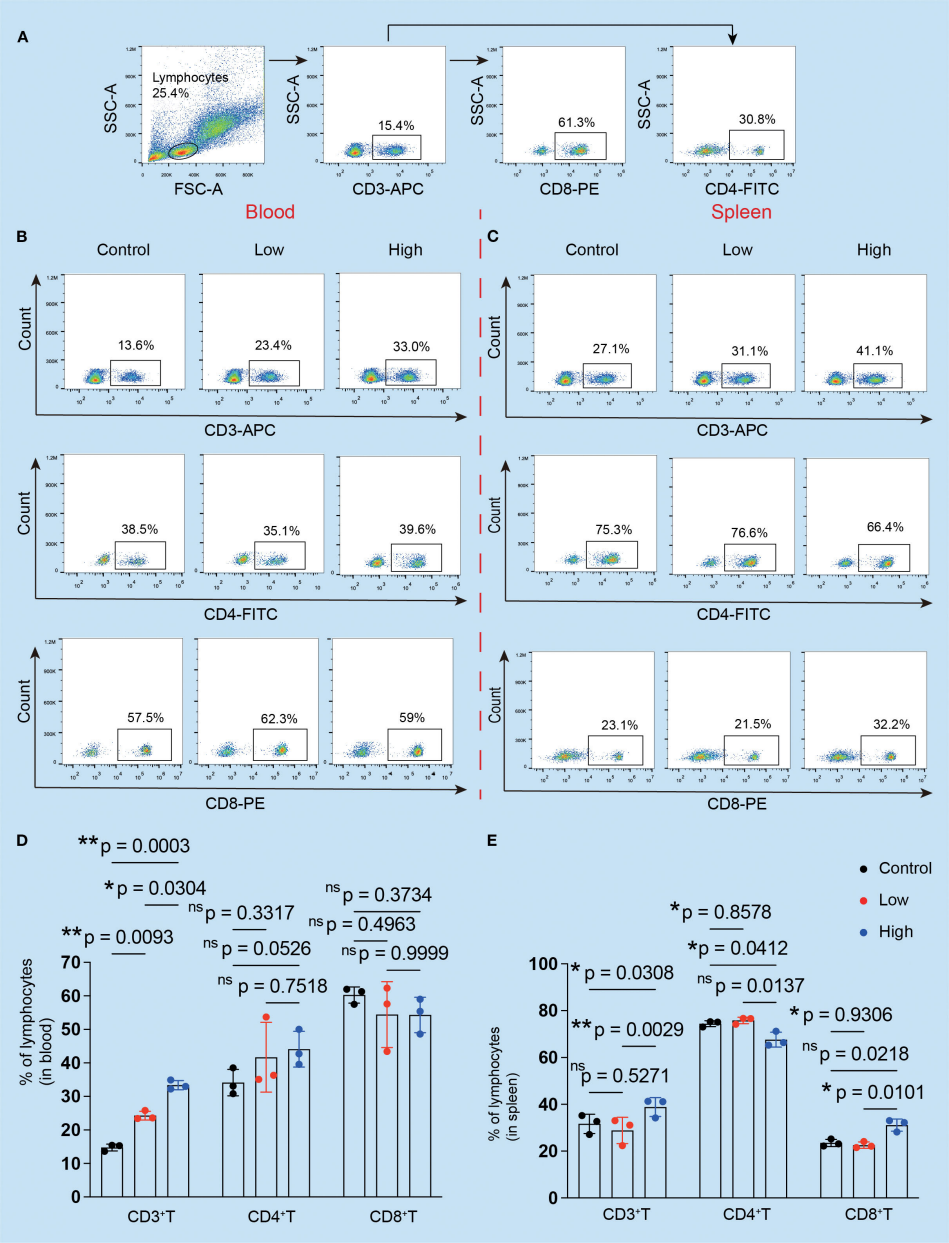
Effects of YFD treatment on the proportions of CD3^+^, CD4^+^, and CD8^+^ T cells. **(A)** The gating strategy. The peripheral serum and spleen tissues were subjected to flow cytometry analysis. **(B, C)** CD3^+^, CD4^+^, and CD8^+^ T cells in the blood and spleen were analyzed via flow cytometry. **(D, E)** Comparison of the proportions of CD3^+^, CD4^+^, and CD8^+^ T cells in the blood and spleen in each group (n=3 for each group). *p< 0.05, **p< 0.01, ^ns^p>0.05 compared with the intended group by ANOVA followed by Dunnett’s t *post hoc* test.

Flow cytometry and immunofluorescence were used to examine the primary tumor-inhibiting and tumor-promoting leukocytes, respectively, to observe changes in the immunogenic microenvironment of TNBC mice. The results revealed that the ratio of CD3^+^ T cells in the peripheral blood increased (**Figure 5A**), but there were no statistically significant differences between the YFD-treated groups in terms of CD4^+^ and CD8^+^ T cells (**Figures 5B, D**). In contrast, the fractions of CD3^+^ and CD4^+^ T cells and CD8^+^ T cells in the spleen increased only with high-dose YFD treatment (**Figures 5C, E**), indicating that high-dose YFD increased the antitumor immune reaction.

**Figure 6:**
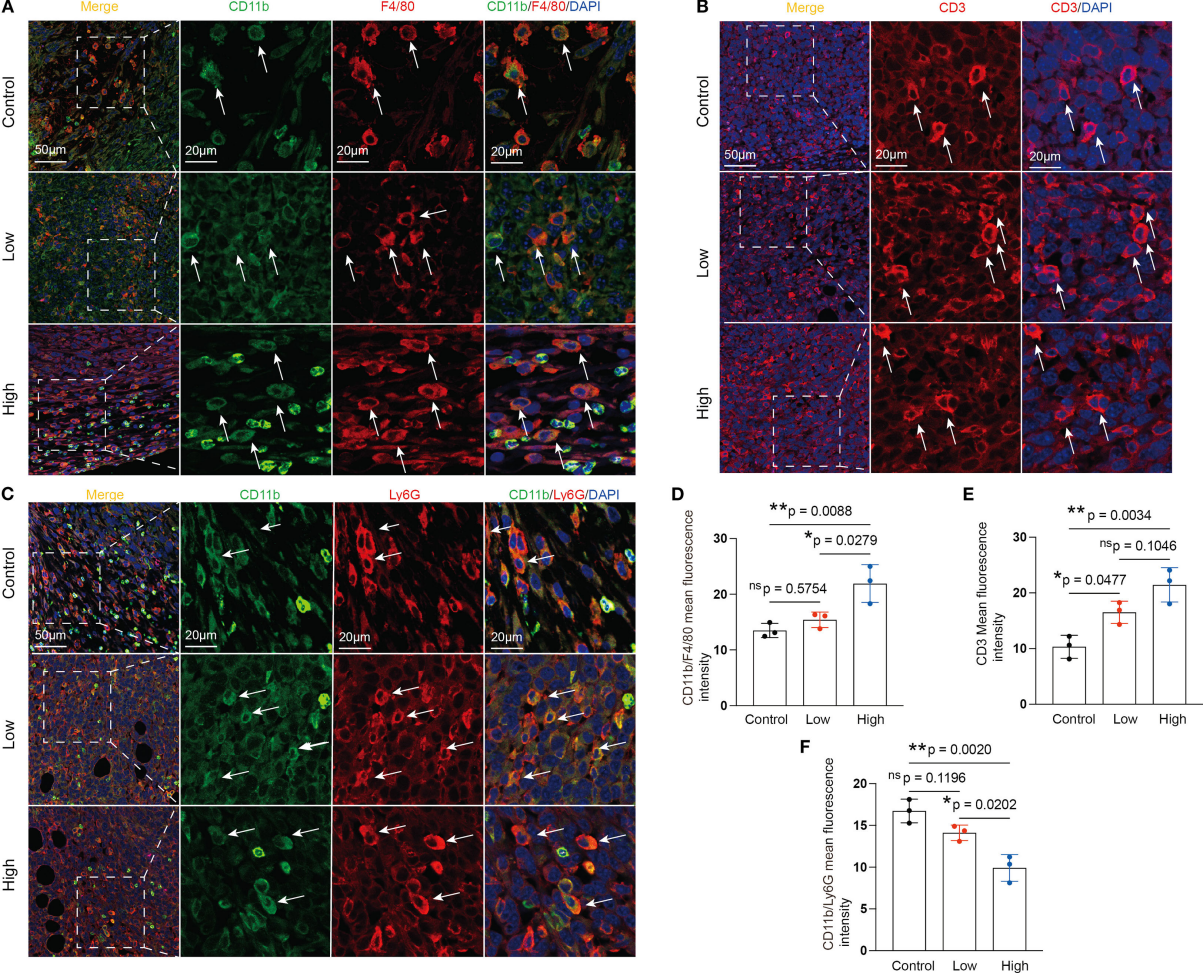
Effects of YFD treatment on tumor-associated macrophages (TAMs), myeloid-derived suppressor cells (MDSCs), and CD3^+^ T cells. **(A-C)** Representative images of IF-stained TAMs, CD3^+^ T cells, and MDSCs. TAMs were labeled with anti-F4/80 and anti-CD11b antibodies. MDSCs were labeled with anti-Ly6G and anti-CD11b antibodies. CD3^+^ T cells were labeled with an anti-CD3 antibody. **(D-F)** Immunofluorescence analysis of TAMs, CD3^+^ T cells, and MDSCs in tumor sections from each group. The data are presented as the means ± SDs (n=3). *p< 0.05, **p< 0.01, ^ns^p>0.05 compared with the intended group by ANOVA followed by Dunnett’s t *post hoc* test.

It is commonly acknowledged that MDSCs and TAMs predominate within the immunosuppressive TME (9). High-dose YFD therapy consistently increased the proportion of CD3^+^ T cells (**Figures 6B, E**) and dramatically decreased the proportions of TAMs (CD11b^+^F4/80^+^) (**Figures 6A, D**) and polymorphonuclear MDSCs (CD11b^+^Ly6G^+^; a crucial MDSC subtype) (**Figures 6C, F**). Collectively, our results suggest that YFD can create a tumor-inhibiting immunogenic milieu in the TME by significantly increasing the number of T cells and reducing the number of TAMs and MDSCs. High-dose YFD treatment had greater tumor-inhibiting effects than did low-dose YFD treatment.

### YFD regulated tumor metabolomic profiling in the breast cancer mouse model

**Figure 7:**
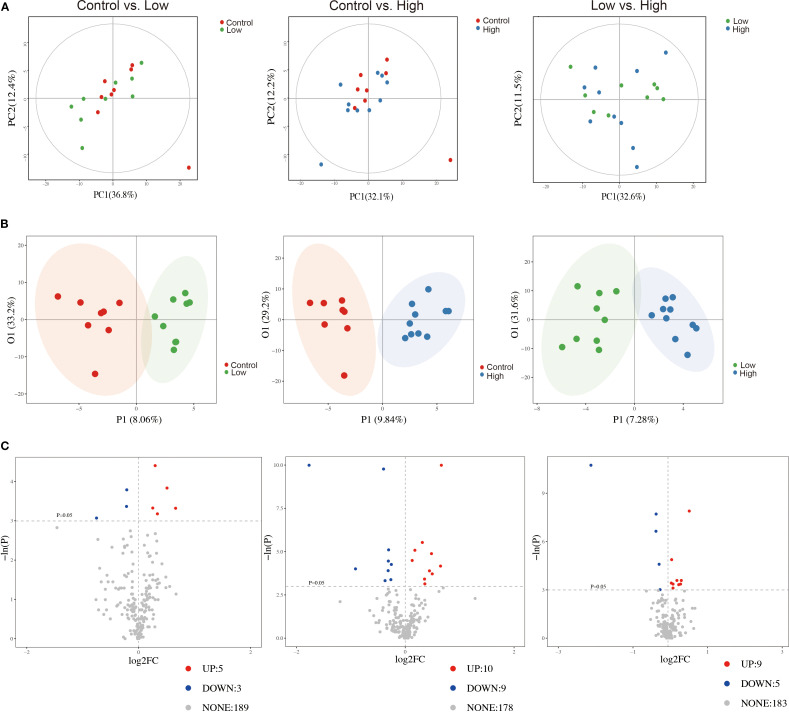
Comparisons of principal component analysis (PCA) score plots, orthogonal projections to latent structure-discriminant analysis (OPLS-DA) score plots, and volcano plots of metabolic profiles among the groups. **(A)** PCA score plots for the control group vs. low-dose group, control group vs. high-dose group, and low-dose group vs. high-dose group. **(B)** OPLS-DA score plots for the control group vs. low-dose group, control group vs. high-dose group, and low-dose group vs. high-dose group. **(C)** Volcano plots for the control group vs. the low-dose group, the control group vs. the high-dose group, and the low-dose group vs. the high-dose group.

We utilized a metabolomic approach to study the changes in metabolites in the breast tumor tissues of mice, and multivariate analysis was combined with PCA and OPLS-DA to identify potential biomarkers. The representative GC-TOF/MS chromatograms and detailed spectral data for metabolite identification are provided in **Supplementary File 2**. The PCA score plots displayed in **Figure 7A** show the overall differences among the groups (control vs. low, control vs. high, and low vs. high). A score plot with aggregated quality control samples indicates good quality control and a stable detection process. Next, we performed OPLS-DA to maximize the covariance among the data to distinguish the metabolites between the groups. As shown in **Figure 7B**, each comparison had good prediction ability, with high R^2^Y and Q^2^ values. In the OPLS-DA model, the parameters were as follows: control vs. low, R^2^Y=0.887, Q^2^=-0.423; control vs. high, R^2^Y=0.911, Q^2^ = 0.439; and low vs. high, R^2^Y=0.888, Q^2^ = 0.334. The separation differences were substantial between the control vs. low, control vs. high, and low vs. high groups, indicating that YFD treatment had a significant effect on the metabolites. A volcano plot combining the strengths of both the variable contributions (variable importance in projection, VIP) and variable reliability (correlation coefficients (**Figure 7C**), Corr. coefficients) was used to screen for potential biomarkers. In this analysis, the threshold values for identifying different metabolites were set at *p*< 0.05 and |log2FC| ≥0.

**Figure 8:**
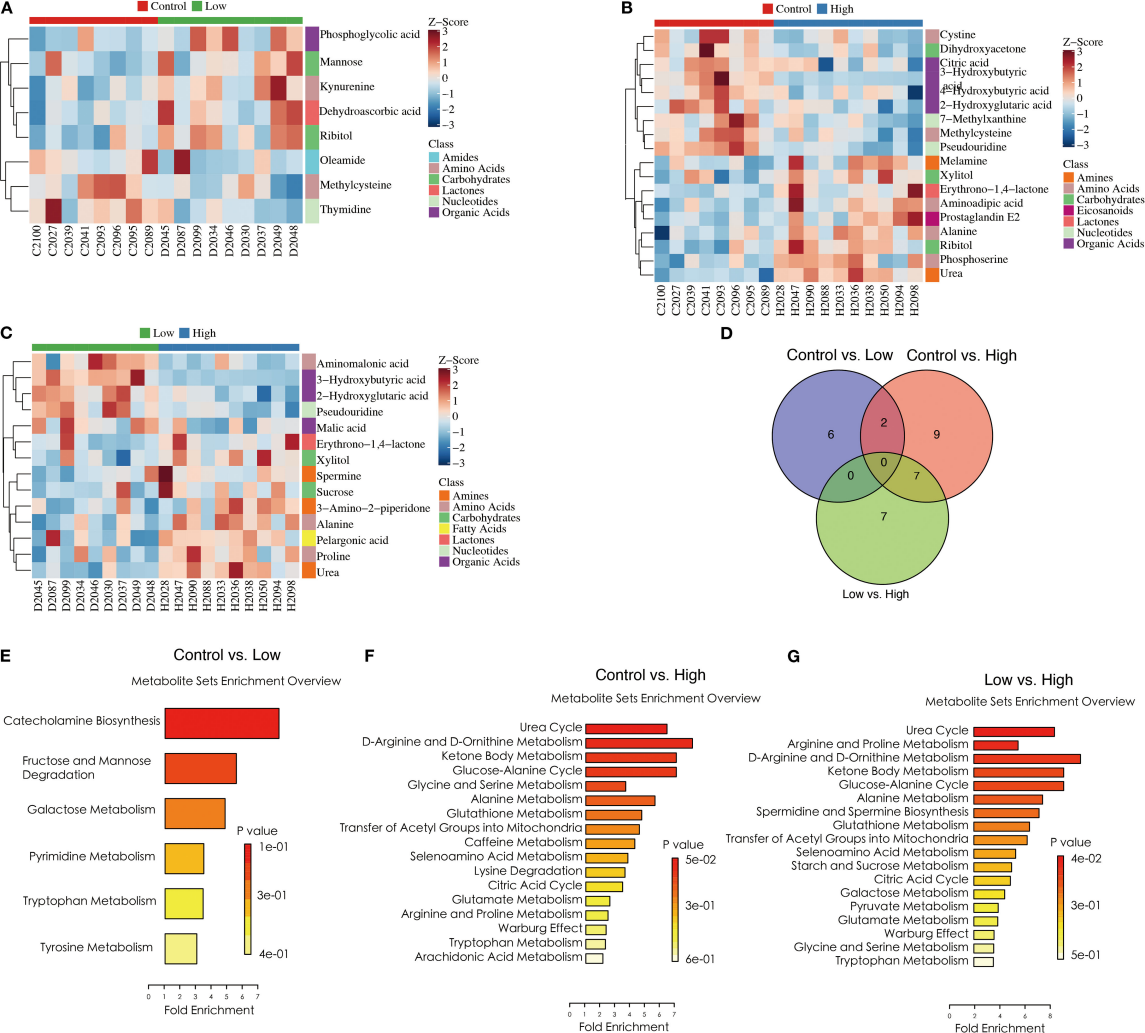
YFD-induced changes in metabolic profiles in a mouse model of breast cancer. **(A-C)** Heatmaps of potential biomarkers for each comparison, including the control group vs. the low-dose group, the control group vs. the high-dose group, and the low-dose group vs. the high-dose group. **(D)** Venn diagram of differentially abundant metabolites between the control group and the low-dose group, between the control group and the high-dose group, and between the low-dose group and the high-dose group. **(E-G)** Metabolite set enrichment in the control group vs. low-dose group, control group vs. high-dose group, and low-dose group vs. high-dose group.

According to the selection threshold, the low-dose YFD group presented five upregulated and three downregulated metabolites compared with the control group (**Figure 8A**), the majority of which fell within the categories of amides (oleamide), amino acids (kynurenine and methylcysteine), carbohydrates (ribitol and mannose), lactones (dehydroascorbic acid), nucleotides (thymidine), and organic acids (phosphoglycolic acid). Compared with those in the control group, ten upregulated and nine downregulated metabolites were detected in the high-dose YFD group, with the majority being amines (urea and melamine), amino acids (alanine, methylcysteine, aminoadipic acid, phosphoserine, and cystine), carbohydrates (dihydroxyacetone, xylitol, and ribitol), eicosanoids (prostaglandin E2), lactones (erythrono-1,4-lactone), nucleotides (7-methylxanthine and pseudouridine), and organic acids (3-hydroxybutyric acid, 2-hydroxyglutaric acid, citric acid, and 4-hydroxybutyric acid) (**Figure 8B**). Compared with the low-dose YFD group, the high-dose YFD group presented nine upregulated and five downregulated metabolites (**Figure 8C**). These metabolites include amines (urea, 3-amino-2-piperidone, and spermine), amino acids (alanine, proline, and aminomalonic acid), carbohydrates (xylitol and sucrose), fatty acids (pelargonic acid), lactones (erythrono-1,4-lactone), nucleotides (pseudouridine), and organic acids (3-hydroxybutyric acid, malic acid, and 2-hydroxyglutaric acid). Interestingly, as shown in **Figures 8A, B, D**, both low- and high-dose YFD increased the amount of ribitol and lowered the level of methylcysteine.

Pathway enrichment analysis was performed on the identified differentially abundant metabolites via the pathway-associated metabolite set (SMPDB) database. Our results revealed that catecholamine biosynthesis; fructose and mannose degradation; and galactose, pyrimidine, tryptophan, and tyrosine metabolism were the key metabolic pathways that differed between the control and low-dose YFD groups (**Figure 8E**). The main metabolic pathways included the urea cycle; D-arginine and D-ornithine metabolism; ketone bodies; the glucose–alanine cycle; glycine and serine metabolism; alanine metabolism; glutathione metabolism; the transfer of acetyl groups into the mitochondria; caffeine metabolism; selenoamino acid metabolism; lysine degradation; the citric acid cycle; glutamate metabolism; arginine and proline metabolism; the Warburg effect; and tryptophan metabolism (**Figure 8F**).

In contrast, the urea cycle, arginine and proline metabolism, D-arginine and D-ornithine metabolism, ketone body metabolism, the glucose–alanine cycle, alanine metabolism, spermidine and spermine biosynthesis, glutathione metabolism, transfer of acetyl groups into mitochondria, and caffeine metabolism were the metabolic pathways that differed between the low-dose and high-dose YFD groups (**Figure 8G**). This may partially explain why high-dose YFD had more potent antitumor effects than did low-dose YFD.

### YFD regulated the tumor transcriptome in BC mice

To investigate the molecular mechanisms underlying the reshaping of the immunogenic BC microenvironment by YFD treatment, we conducted a transcriptome analysis of the tumor tissue. The quality control data are presented in **Supplementary Table 1**. The average raw reads of all the samples were 45885130, with a mean effective rate of 83.60%. The clean Q20 ratios of these samples ranged from 98.97% to 99.12%, with an average ratio of 99.06%, and the clean Q30 ratios ranged from 95.51% to 96.12%, with a mean ratio of 95.89%. These data suggested that the RNA-Seq data were precise and could be used for subsequent analyses.

**Figure 9:**
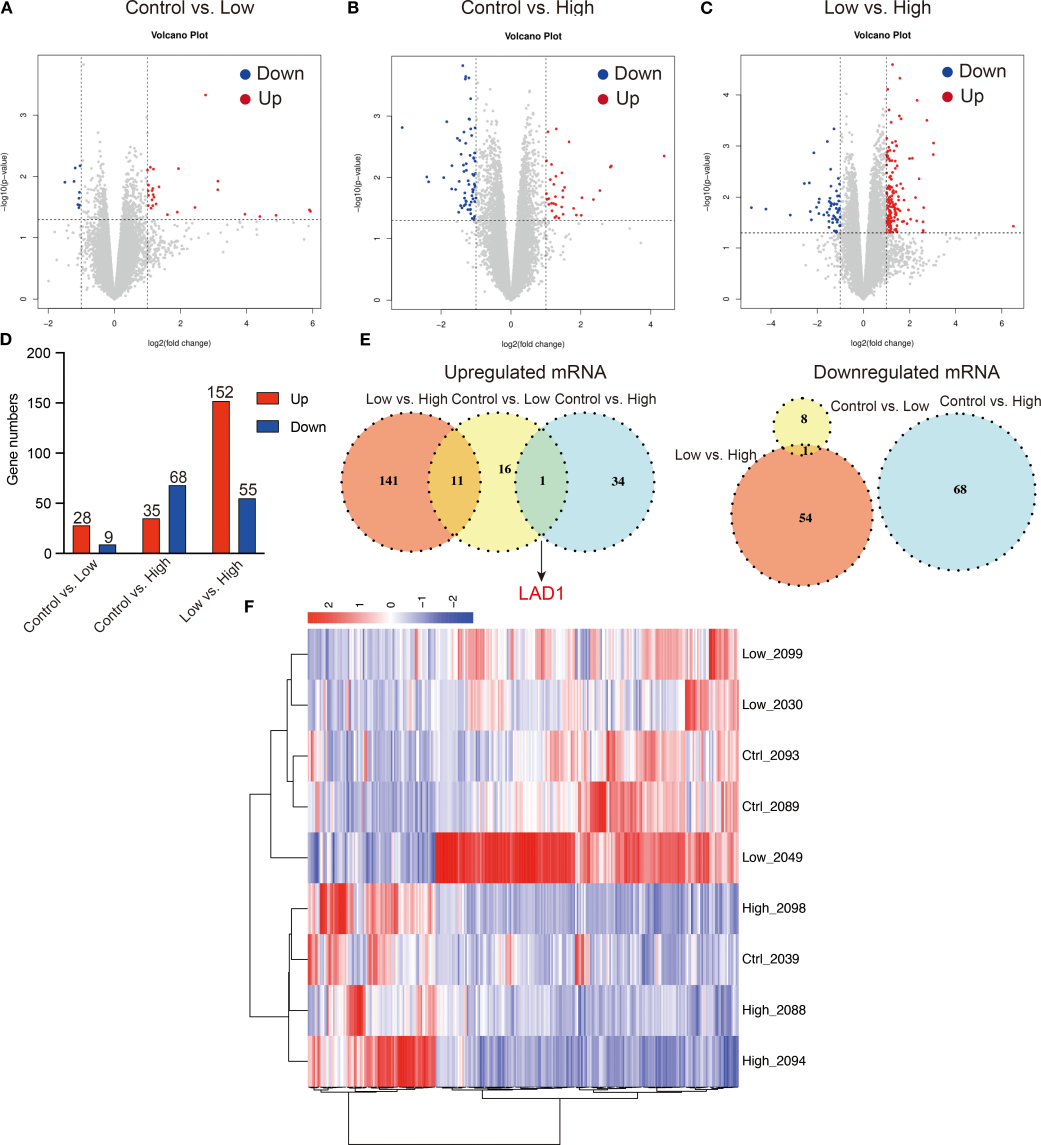
Differentially expressed genes (DEGs) among the groups. **(A-D)** Volcano plots for the control group vs. low-dose group, control group vs. high-dose group, and low-dose group vs. high-dose group. **(E)** Shared upregulated and downregulated DEGs among the groups. **(F)** DEG heatmap for the groups.

In this study, edge R was used to analyze the differentially expressed genes (DEGs), and the results are presented in **Figure 9**. Our findings indicate that low-dose YFD treatment resulted in 28 downregulated genes and nine upregulated genes compared with those in the control group (**Figures 9A, D**). Conversely, high-dose YFD treatment induced the downregulation of 35 genes and the upregulation of 68 genes (**Figures 9B, D**). In total, 207 DEGs were identified between the low- and high-dose YFD groups. Among these genes, 152 genes were upregulated and 55 genes were downregulated in the low-dose YFD group compared with the high-dose YFD group (**Figures 9C, D**). Notably, both low- and high-dose YFD treatments resulted in the downregulation of the ladinin-1 (*LAD1*) gene (**Figure 9E**), which has been associated with the metastatic potential of cancer (37, 38). We hypothesized that this gene could be the key gene responsible for the antigrowth and antimetastatic effects of YFD. High-dose YFD treatment also upregulated the expression of *Ank3, Ube2ql1, Baalc, Thbs4*, and *Dsg2*, which are known to participate in cancer growth and metastasis (39–43). These findings suggest that high-dose YFD may have stronger antitumor effects than low-dose YFD does. Additionally, a statistically significant difference in gene expression was observed among the three groups, as shown in the DEG heatmap (**Figure 9F**).

**Figure 10:**
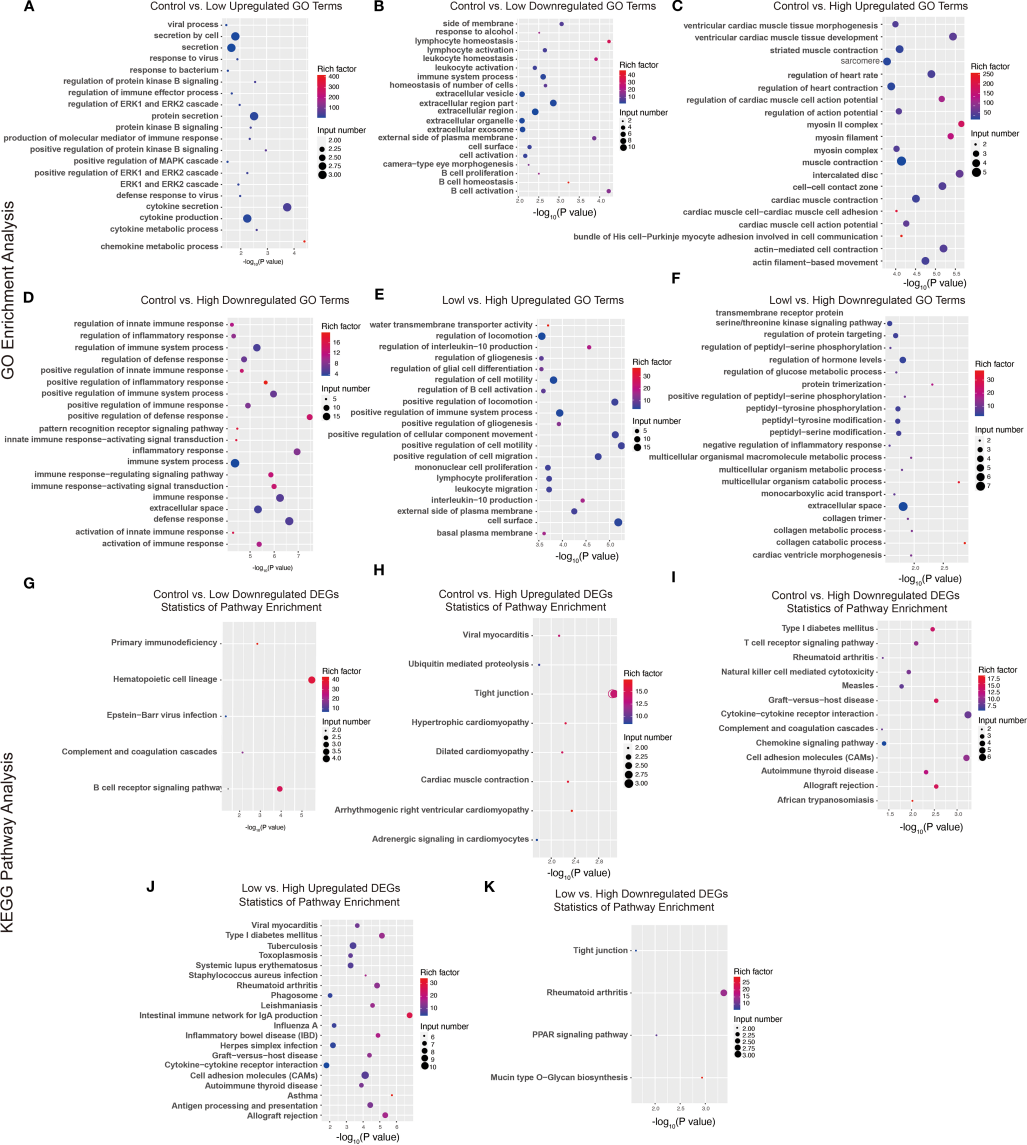
GO terms and KEGG pathways of DEGs. **(A-F)** GO terms enriched with upregulated and downregulated genes from the comparisons between the control group and the low-dose group, between the control group and the high-dose group, and between the low-dose group and the high-dose group. **(G-K)** KEGG pathways enriched with upregulated and downregulated DEGs between the control group and the low-dose group, between the control group and the high-dose group, and between the low-dose group and the high-dose group.

Furthermore, we performed functional annotation analysis of DEGs via the GO database and set the selection threshold for enriched GO terms at *p*< 0.05. Compared with the low-dose YFD group, the control group presented increased defense responses to viruses, protein kinase B signaling, the MAPK cascade, and ERK signaling (**Figure 10A**) and decreased regulation of immune system processes, lymphocytes, leukocytes, B cells, and extracellular regions (**Figure 10B**). The DEGs were enriched in five pathways: primary immunodeficiency, hematopoietic cell lineage, Epstein–Barr virus infection, complement and coagulation cascades, and the B-cell receptor signaling pathway (**Figure 10G**).

Compared with the high-dose YFD group, the control group presented an increase in GO terms related to the regulation of cardiac muscle function and heart rate (**Figure 10C**). In contrast, the GO terms related to the positive regulation of the innate immune response, inflammatory response, and immune response-regulating signaling pathways were downregulated in the control group (**Figure 10D**). These DEGs were enriched in tight junctions, hypertrophic cardiomyopathy, type I diabetes mellitus, the T-cell receptor signaling pathway, natural killer cell-mediated cytotoxicity, cytokine–cytokine receptor interactions, the chemokine signaling pathway, and cell adhesion molecules (**Figures 10H, I**).

Compared with the high-dose YFD group, the low-dose YFD group presented increased expression of GO terms such as regulation of interleukin-10 production, regulation of gliogenesis, regulation of B-cell activation, positive regulation of cell migration, and positive regulation of cell motility (**Figure 10E**). Conversely, the main downregulated GO terms were transmembrane receptor protein serine/threonine kinase signaling pathway, regulation of protein targeting, peptidyl-serine phosphorylation, glucose metabolic process, and collagen metabolic process (**Figure 10F**). These DEGs were enriched predominantly in pathways such as Type I diabetes mellitus, tuberculosis, rheumatoid arthritis, cytokine–cytokine receptor interaction, tight junctions, and the PPAR signaling pathway (**Figures 10J-K**).

### Integrative analysis of the metabolome and transcriptome

**Figure 11:**
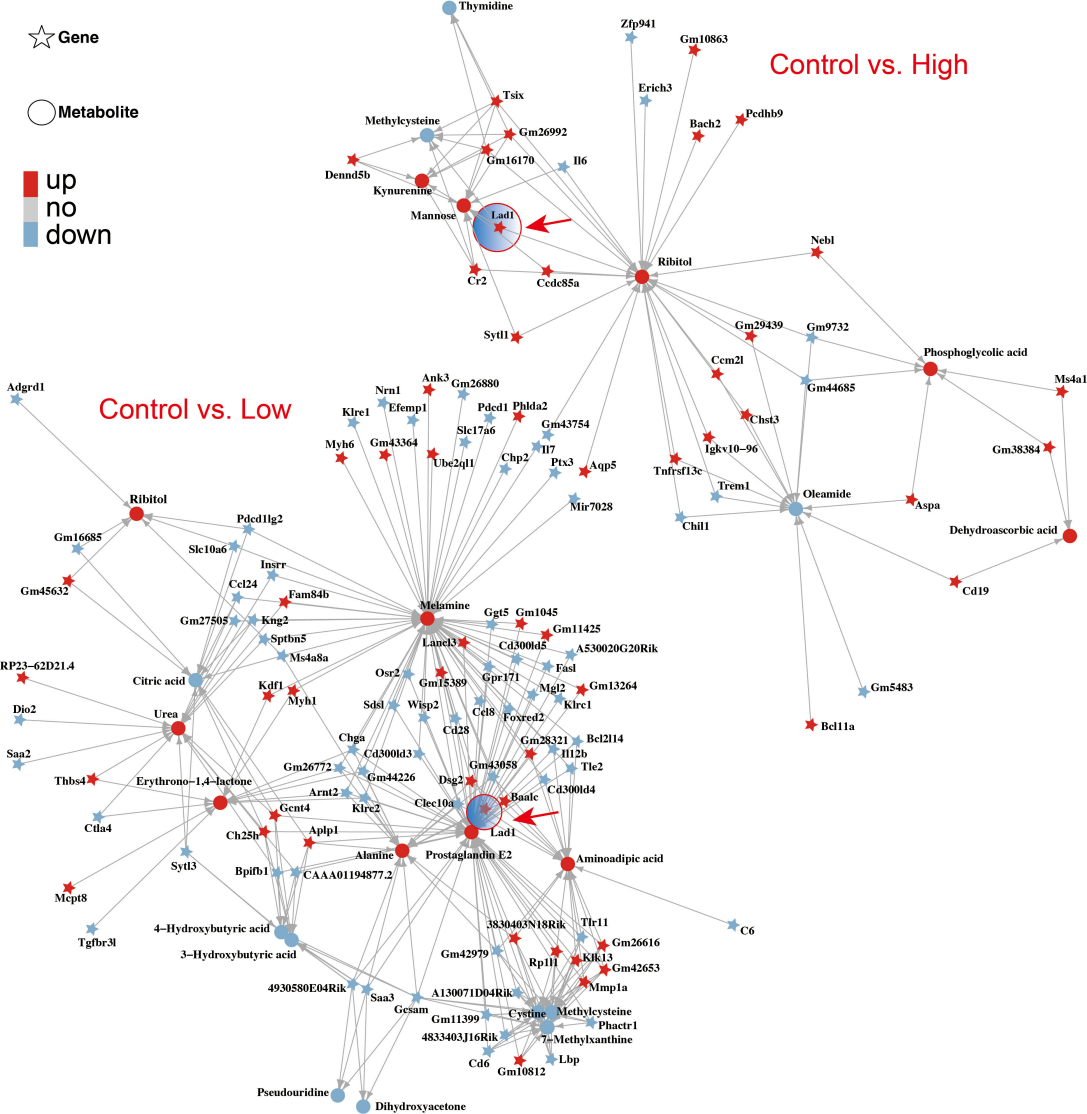
Interaction network of genes and metabolites affected by YFD. The correlation coefficient between genes and metabolites was measured via the Spearman correlation coefficient. The arrow direction represents the direction of regulation from the gene to the metabolite. Both low-dose and high-dose YFD treatment upregulated the *LAD1 gene*.

Next, we analyzed the correlations between the identified genes and their metabolites. The correlation coefficient was measured via the Spearman correlation coefficient method with the Cor function of R language. A correlation coefficient greater than 0.8 and a *p* value < 0.05 were used to determine a significant correlation between the metabolites and genes. An interaction network of gene–metabolite expression was constructed, and the igraph package of the R language was used to draw the network diagram. The molecular types (genes and metabolites) are marked on the network icon, and a line was drawn between the interacting molecules. The arrow represents the default direction of regulation from the gene to the metabolite. Our results revealed that both high and low doses of YFD upregulated *LAD1* expression in mice with TNBC. This leads to reduced expression of methylcysteine and increased expression of mannose, ribitol, melamine, alanine, aminoadipic acid, and prostaglandin E2 (**Figure 11**).

### YFD induced M1 macrophage polarization and inhibited M2 macrophages, likely by inactivating the MEK/ERK1/2 pathway

**Figure 12:**
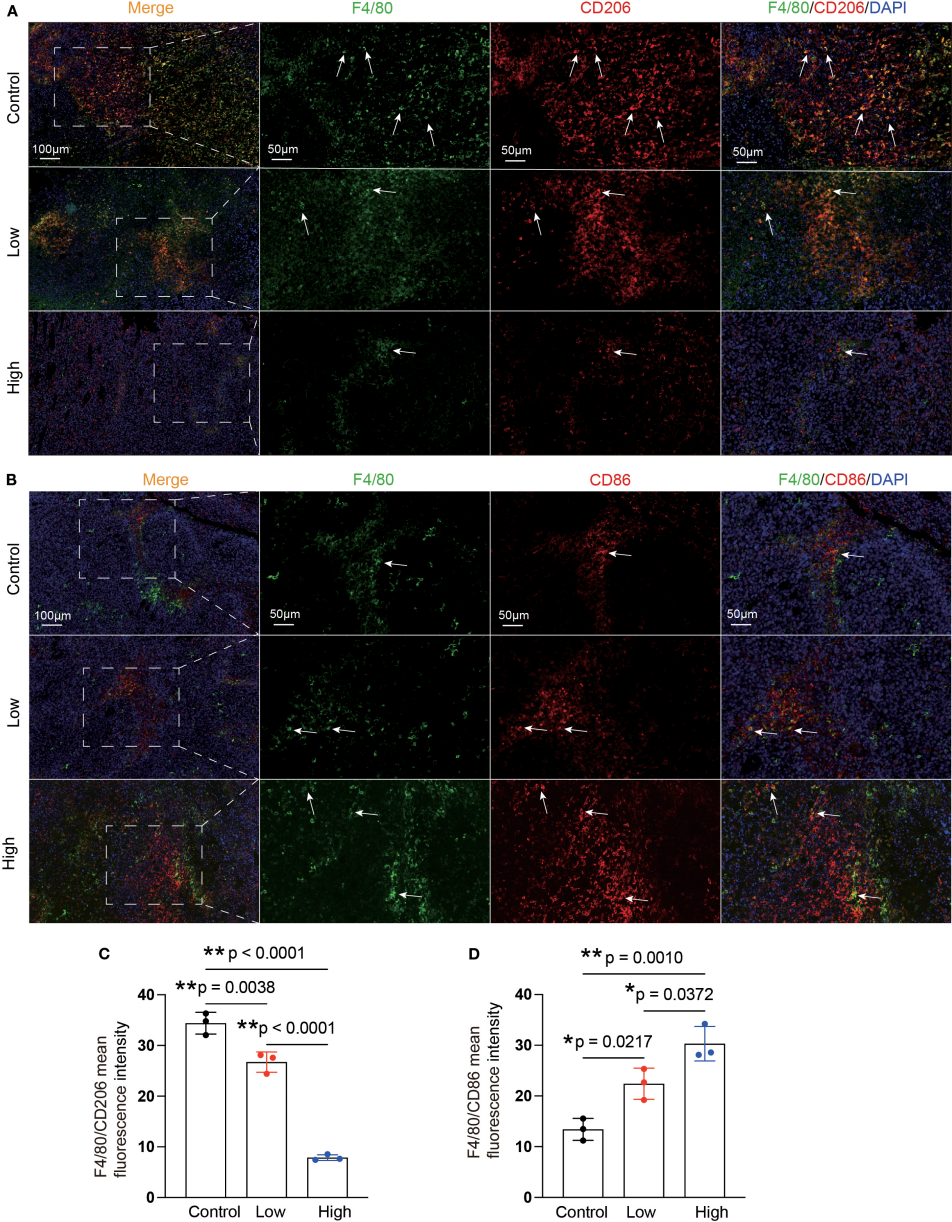
YFD can remodel the polarization phenotypes of TAMs. **(A, B)** Representative immunofluorescence images of M1-type TAMs and M2-type TAMs in tumor sections from each group. The white arrows indicate macrophages. **(C, D)** Immunofluorescence analysis of M1- and M2-like macrophages in tumor sections from each group. The data are presented as the means ± SDs (n=3). *p< 0.05, **p< 0.01, ^ns^p>0.05 compared with the intended group by ANOVA followed by Dunnett’s t *post hoc* test.

**Figure 13:**
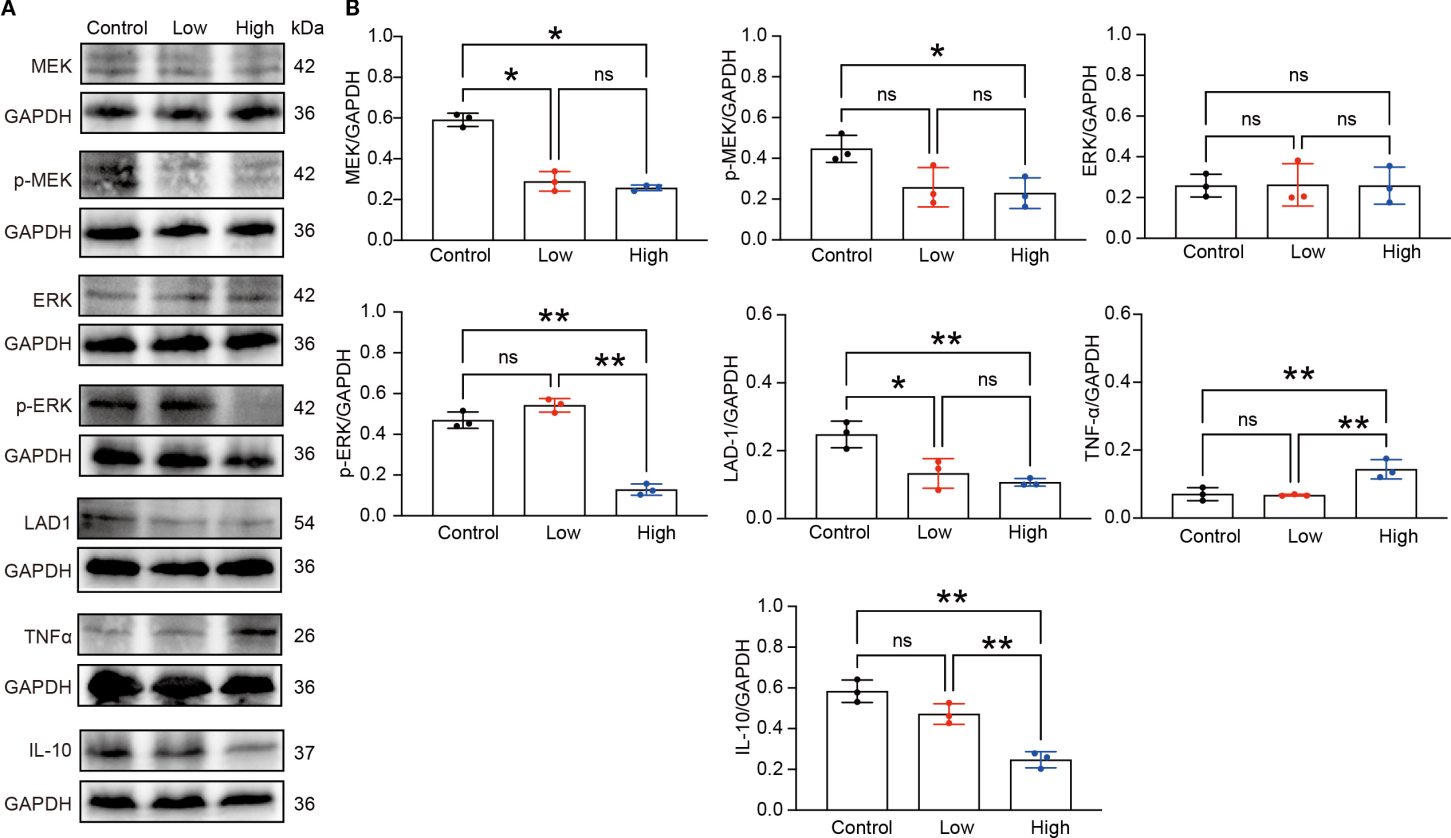
YFD inhibited the MEK/ERK1/2 pathway. **(A)** Expression of LAD1, TNFα, IL-10, and MEK/ERK1/2 signaling proteins in tumor tissues from each group, as measured by Western blot analysis. **(B)** Densitometry values for the proteins were normalized to those of GAPDH. All the data represent the means ± SDs of three independent experiments performed in triplicate. *p< 0.05, **p< 0.01, ^ns^p>0.05 compared with the intended group by ANOVA followed by Dunnett’s t *post hoc* test.

Enrichment analysis of the RNA-seq data revealed that, compared with the control group, the YFD treatment groups presented significant enrichment of terms related to the MAPK cascade, ERK signaling, and regulation of the inflammatory response. These findings indicate that the antitumor effects of YFD may be mediated through the MAPK and ERK pathways, which regulate the immune response. Recent studies have shown that *LAD1* can be affected by the EGFR/MEK/ERK1/2 cascade, which affects the invasion and migration of BC cells (38). Additionally, TAMs with an M2-like phenotype are correlated with an immunosuppressive TME and cancer metastases (14). To measure the levels of M1- and M2-like TAM markers, we analyzed the activated phenotype via the expression levels of F4/80/CD86 (M1-like marker) and F4/80/CD206 (M2-like marker). Our immunofluorescence results revealed a significant increase in the fluorescence intensity of M1-like TAMs and a decrease in that of M2-like TAMs in the YFD-treated groups (**Figures 12A-D**). YFD treatment resulted in a substantial increase in TNF-α and a decrease in IL-10 expression (**Figures 13A, B**). Additionally, we observed a decrease in the expression of MEK/ERK1/2 signaling pathway proteins and LAD1 following YFD administration (**Figures 13A, B**). Overall, these results suggest that YFD treatment induces M1 macrophage polarization and inhibits M2 macrophage polarization by partially inactivating the MEK/ERK1/2 pathway.

## Discussion

The relationship between the TME and the progression of various solid cancers, such as lung, breast, gastric, and colorectal cancers, is widely accepted (9, 44). TNBC, a highly aggressive and metastatic subtype of BC, lacks specific targets or targeted therapeutics (3). Therefore, reshaping the components of the TME, including immunosuppressive cells and metabolites, may be a promising therapeutic approach for TNBC (45). Our study aimed to validate the bioactive ingredients, anticancer effects, and underlying mechanisms of action of the multitarget Chinese medicine formula YFD in TNBC mice via a comprehensive approach.

Twenty compounds were identified, including l-phenylalanine, nicotinic acid, linoleic acid, benzoic acid, palmitic acid, caffeic acid, formononetin, apigenin 7-O-glucuronide, salicylic acid, catechol, oleanolic acid, chlorogenic acid, rutin, apigenin, asiatic acid, catechin, eicosapentaenoic acid, quercetin, vanillin, and astragalin. Our findings indicated that YFD significantly suppressed breast tumor growth and lung metastasis in a dose-dependent manner, with high-dose YFD treatment resulting in stronger antitumor effects. Importantly, YFD significantly affected the TME by increasing the number of T cells and decreasing the number of TAMs and MDSCs. Furthermore, YFD was found to target important immune regulatory pathways, including the *LAD1* gene, and promote the polarization of M1 macrophages while inhibiting M2 macrophage polarization and MDSC accumulation by inactivating the MEK/ERK1/2 pathway. Metabolomic analysis demonstrated that YFD affects various metabolic pathways related to immune response regulation. Recently, several studies have validated the anticancer mechanisms of ancient and classical Chinese medicine decoctions, such as the Tao Hong Si Wu decoction (46), Gegen Qinlian Decoction (47), Siwu Decoction (48), Liujunzi Decoction (49), and Banxia Xiexin Decoction (50), but less emphasis has been placed on their effects on the TME. In the present study, we demonstrated that YFD can inhibit TNBC progression by remodeling the TME. In addition, the material basis, effects on TME remodeling, and transcriptomic and metabolomic characteristics of the decoction were validated via combined and systematic approaches, in accordance with the holistic view of TCM.

Mounting evidence has shown that the identified components of YFD exert significant anticancer effects. Patients with BC who received L-phenylalanine mustard demonstrated prolonged disease-free survival and a significant survival benefit compared with those who received placebo (51). Meng et al. reported that a copolymer of L-phenylalanine and salicylic acid significantly inhibited the lung metastasis of BC (52). Additionally, derivatives of benzoic acid retard tumor growth and metastasis by inhibiting the TNFα/NF-κB and iNOS/NO pathways (53). A recent study revealed that palmitic acid-modified human serum albumin paclitaxel nanoparticles significantly polarized macrophages to the M1 type, thus reshaping the TME and inhibiting BC metastasis (54). Quercetin and astragalin are known to exert strong anticancer effects (55, 56). Nicotinic acid (57), linoleic acid (58), caffeic acid (59), oleanolic acid (60), chlorogenic acid (61), rutin (62), apigenin (63), asiatic acid (64), catechin (65), eicosapentaenoic acid (66), vanillin (67), and formononetin (68) are also considered promising anticancer agents.

There is significant evidence that TAMs and MDSCs are primarily responsible for immune suppression and evasion in cancer (69–72) and that they play a vital role in tumor growth and metastasis (73, 74). MDSCs can increase the expression of the immune checkpoint molecule PD-L1, which suppresses the T-cell response by interacting with PD-1 on T cells (75, 76). Furthermore, the production of ARG1 by MDSCs can lead to increased consumption of extracellular l-arginine, which is essential for T-cell metabolism and function, ultimately resulting in T-cell inhibition (77). Additionally, the increased secretion of nitric oxide, oxygen radicals, and reactive nitrogen species from MDSCs can impair T-cell function (77–79). Therefore, targeting immunosuppressive cells is a potential therapeutic approach for cancer treatment (11). Mounting evidence suggests that TCM may effectively inhibit tumor growth by suppressing MDSCs. One major biocomponent of TCM, icariin, reduces MDSCs and restores IFN-γ production in CD8^+^ T cells, ultimately leading to tumor growth (80). In addition, Gansui-Banxia decoction, a TCM formula, has notable antitumor effects by reducing the accumulation of MDSCs, which in turn inhibits the AKT/STAT3/ERK pathway (81). Consistently, in this study, a reduced accumulation of MDSCs in tumors was observed.

Similarly, we found that YFD reduced the proportion of TAMs in tumors. In addition, the inhibition of M2 polarization and promotion of M1 polarization of TAMs by YFD were observed. TAMs are closely associated with a poor prognosis in multiple cancers (82–84). M1-like macrophages generally have high levels of CD80, CD86, TNF-α, IL-6, and iNOS, which exert antitumorigenic effects. In contrast, M2-like macrophages usually express CD163, CD206, IL-10, and arginase 1, which are considered protumorigenic (85). Mounting evidence suggests that an increase in M2-like TAMs creates a microenvironment that promotes tumor progression within the TME. This increase in the number of M2-like TAMs is correlated with tumor growth and metastasis. Conversely, an increase in M1-like TAMs is closely associated with less aggressive tumors (86, 87). Switching from the M2-like phenotype to the M1-like phenotype has been shown to inhibit tumor angiogenesis and metastasis in BC (88). Transcriptomic analysis was conducted to investigate the mechanisms underlying the YFD-mediated reduction in TAMs and MDSCs in breast tumors. The RNA-seq data revealed that the GO terms related to the regulation of immune processes were enriched in the low- and high-dose YFD treatment groups compared with the control group. Additionally, several signaling pathways, including protein kinase B signaling, the ERK1/2 cascade, and the MAPK pathway, are involved in the immune-regulating properties of YFD (89, 90). The YFD treatment group presented decreased expression of *LAD1*. LAD1 is an anchoring filament protein in mammalian epidermal cells (91, 92) and has been implicated in the metastatic potential of breast (38), prostate (93), and colorectal cancers (37). A recent study explored genomic profiles and identified LAD1 as a potential target for TNBC therapy (94). Additionally, high levels of LAD1 transcripts have been linked to a poor prognosis in patients with BC (38). LAD1 is a downstream target of the EGFR/MEK/ERK1/2 signaling pathway, which affects actin polymerization and cross-linking, ultimately controlling BC cell migration and proliferation. LAD1 depletion reduces the invasion and migration of BC cells, and similar results have been reported in colorectal cancer cells (37). We also found that LAD1 transcription and expression were increased in TNBC mice but were restored by YFD administration, possibly through inhibition of the MEK/ERK1/2 signaling pathway.

The MEK/ERK/1/2 signaling pathway is a widely known MAPK pathway that plays crucial roles in apoptosis, cell proliferation, and the immune response (95). Studies have shown that this pathway is also involved in tumor invasion and metastasis (96, 97). Preclinical studies have revealed that the MEK/ERK pathway is hyperactivated in TNBC, suggesting that targeting this pathway may be an effective treatment strategy for TNBC (98, 99). Zhang et al. reported that the activation of EGFR/MEK/ERK signaling contributes to BC progression (100). Conversely, inhibition of the RAS/RAF/MEK/ERK and PI3K/AKT/mTOR signaling pathways suppressed the growth of BC cells (101). The activation of MEK/ERK1/2 signaling can reverse TAM polarization from the tumor-inhibiting M1-like phenotype to the tumor-promoting M2-like phenotype, leading to increased metastatic potential. However, Kang et al. demonstrated that puerarin, a major bioactive component of the TCM herb Ge-gen (Radix Puerariae), not only inhibits M2-like macrophage polarization but also suppresses tumor growth and metastasis. This was achieved through the partial inactivation of MEK/ERK1/2 signaling in a non-small cell lung carcinoma xenograft model (102, 103). ERK signaling cascades are involved in regulating MDSCs in cancer cells. Liu et al. reported that activation of the MEK/ERK1/2 pathway could increase MDSC recruitment to the spleen and tumor tissues of tumor-bearing mice. This ultimately promotes tumor growth and metastasis (104). A recent study revealed that the inhibitor SCH772984 induced apoptosis in MDSCs and increased the ratio of M1-like phenotype TAMs (105). Ras/MEK-dependent CXCL1/2 expression mediates the recruitment of immunosuppressive MDSCs to TNBC (106). A reduction in MDSC infiltration was partially achieved through the suppression of IL-6 via the inhibition of MEK (107). Our observations indicated that activation of the MEK/ERK1/2 signaling pathway led to increased accumulation of MDSCs and M2-like TAMs in TNBC mice, which was effectively reversed by YFD treatment. Therefore, we speculated that inactivation of MEK/ERK1/2 could play a role in the remodeling of the immune landscape by YFD.

We also investigated the metabolomic pathways affected by YFD administration in TNBC mice. Our findings revealed significant changes in various metabolic pathways, including the urea cycle, arginine and proline metabolism, D-arginine and D-ornithine metabolism, glutamate metabolism, the glucose–alanine cycle, alanine metabolism, the transfer of acetyl groups into the mitochondria, pyruvate metabolism, the Warburg effect, glycine and serine metabolism, and tryptophan metabolism. These changes may be responsible for the antitumor effects observed in YFD-treated mice.

Aberrant metabolism is correlated with the immune response in various cancers, including breast (108), lung (109), gastric, and colorectal cancers (110). The urea cycle (UC) in the liver converts excess nitrogen waste into disposable urea. Enzymes involved in UC, such as ornithine transcarbamylase (OTC), argininosuccinate synthase, argininosuccinate lyase, and arginase (ARG), are the primary sources of endogenous arginine, ornithine, and citrulline in the liver. These enzymes meet cellular needs (111). Changes in UC gene expression may contribute to cancer development and progression by affecting the expression of UC-related metabolites. Research has demonstrated that the overexpression of ARG1 and OTC leads to the accumulation of ammonia, which is often observed in cancer cells (112). This excess ammonia can be utilized and recycled by cancer cells through glutamate dehydrogenase to synthesize amino acids and nucleic acids, which fuel tumor growth (112). Moreover, modifications of UC enzymes within cancer cells can alter the TME, ultimately affecting the immune response and the initiation of metastasis.

Ovarian cancer cells reportedly secrete extracellular vesicles containing ARG1, thus suppressing antigen-specific T-cell proliferation. This leads to immune suppression and enhanced tumor growth (113). Cytoplasmic ornithine, an intermediate product of UC, is a substrate for ornithine decarboxylase (ODC) and plays a significant role in putrescine synthesis (114). Dysregulated ornithine metabolism and subsequently elevated polyamine biosynthesis have been linked to tumor growth (115). The inhibition of polyamine metabolism can result in decreased tumor growth by increasing T-cell infiltration and the accumulation of antitumorigenic M1-like TAMs (116, 117). Dysregulated biosynthesis of glycine, serine, and tryptophan, which are essential for the synthesis of proteins, nucleic acids, and lipids, has been implicated in immune function and cancer progression (118–120). A recent study reported that itaconate production by MDSCs inhibits the proliferation and function of CD8^+^ T cells by suppressing serine and glycine biosynthesis. This ultimately leads to increased tumor growth (121). Additionally, a study revealed that microbial indole production via tryptophan metabolism can activate aryl hydrocarbon receptors in TAMs. This leads to tumor-promoting polarization of TAMs and suppresses inflammatory CD8^+^ T-cell infiltration in the TME, ultimately promoting pancreatic ductal adenocarcinoma growth (122). Reprogramming the urea cycle; arginine, ornithine, and glutamate metabolism; and glycine, serine, and tryptophan metabolism could offer new perspectives for the development of anticancer therapies. Our study also revealed that YFD administration induced changes in metabolic profiles, which could explain the antitumor effects of YFD.

Several studies have demonstrated that abnormal glucose, amino acid, glutamine, and lipid metabolism are typical hallmarks of cancer (123) and can be used to predict the prognosis of patients with cancer (124–127). Tumor-derived exosomes can induce macrophages in a premetastatic environment to adopt an M2-like phenotype. This is achieved through the activation of NF-κB, which increases glycolysis and lactate production. These changes ultimately facilitate tumor metastasis (128). Aerobic glycolysis (the Warburg effect) has been shown to facilitate tumor invasion and metastasis by producing lactate, which plays a vital role as a proinflammatory and immunosuppressive mediator (129). Chen et al. reported that an acidic microenvironment caused by the Warburg effect can have a significant effect on the macrophage-mediated immunosurveillance of cancer cells. This was due to a shift from an M1-like phenotype to an M2-like phenotype (130). A recent study revealed that M2-like TAMs exhibit increased glucose uptake, leading to O-GlcNAcylation and the promotion of cancer metastasis and chemoresistance (73). Our study revealed that YFD administration could recondition aerobic glycolysis; however, further investigation is needed to understand the underlying mechanisms involved. Single-cell RNA transcriptomic and bioinformatics analyses can be used to screen for potential changes in the genes and pathways associated with TAMs and MDSCs induced by YFD.

We utilized a gene–metabolite expression interaction network to illustrate the relationships between the identified genes and metabolites. Correlation analysis revealed that the target gene *LAD1* may affect methylcysteine, mannose, ribitol, melamine, alanine, aminoadipic acid, and prostaglandin E2 levels. Among these metabolites, mannose, ribitol, alanine, and prostaglandin E2 are associated with immune regulation and tumor growth (131–134). However, the direct and indirect effects of *LAD1* on these metabolites remain to be elucidated.

This study has several limitations. First, although the bioactive components with anticancer properties of YFD were identified, the main ingredients that can reshape the TME and their targets or signaling pathways could not be validated. In the future, an approach combining integrated network pharmacology, molecular docking, and proteomics should be used to reveal the relationships underlying the component–target–action network. Second, the effects of YFD on TAMs and MDSCs should be tested in other breast cancer cell lines. This study primarily utilized the 4T1 syngeneic mouse model, which limits generalizability to other TNBC subtypes. Future work will validate YFD efficacy in additional models (e.g., MDA-MB-231 xenografts). Third, although YFD can recondition a wide range of metabolic pathways involved in cancer progression, the primary metabolic pathways involved in the anticancer effects of YFD on the TME have not been investigated. Finally, the limited efficacy observed at the low YFD dose (e.g., absence of a PET–CT metabolic response) suggests potential bioavailability thresholds (e.g., intestinal absorption barriers) and pharmacodynamic thresholds (e.g., insufficient target engagement). As this study prioritized validation of the formula’s holistic efficacy, systematic pharmacokinetic investigations (e.g., plasma exposure-AUC quantification) and dose-escalation experiments were not conducted, precluding a precise definition of the minimal effective dose and maximum tolerated dose. Future studies should quantify tumor drug concentrations via patient-derived xenograft (PDX) models coupled with LC–MS/MS and predict human dose–exposure relationships via physiologically based pharmacokinetic modeling. In addition, biomarker-guided dose exploration should be implemented to optimize the clinical therapeutic window of YFD.

## Conclusion

In the present study, we used UHPLC-Q/Orbitrap MS and metabolomic and transcriptomic approaches to investigate the components and potential mechanisms of YFD in YFD treatment. These results suggest that YFD may target various immune regulatory pathways, leading to reshaping of the TME. This effect may be achieved by inactivating the MEK/ERK1/2 and *LAD1* genes. Additionally, YFD may reprogram a wide range of altered metabolic pathways involved in cancer progression, such as the urea cycle and the metabolism of arginine, proline, D-arginine, D-ornithine, glutamate, pyruvate, the Warburg effect, glycine, serine, and tryptophan. Although YFD has been found to induce TME remodeling in BC, the underlying mechanisms require further investigation and validation. Specifically, the regulation of the urea cycle; aerobic glycolysis; and glycine, serine, tryptophan, and ornithine metabolism should be studied in more detail.

## Data Availability

The raw data supporting the conclusions of this article will be made available by the authors, without undue reservation.
