# Supplementary material for: Yiai Fuzheng decoction inhibits triple-negative breast cancer by remodeling the immune microenvironment
Source: Front Immunol. 2025 Sep 30;16:1615631. doi: 10.3389/fimmu.2025.1615631 (PMC12518410; doi:10.3389/fimmu.2025.1615631)
Supplement: Supplementary file 2 [file DataSheet1.zip › Supplementary File 2/Treatment/D_vs_H/06_Potential_Biomarkers/Markers_Boxplot.pdf]

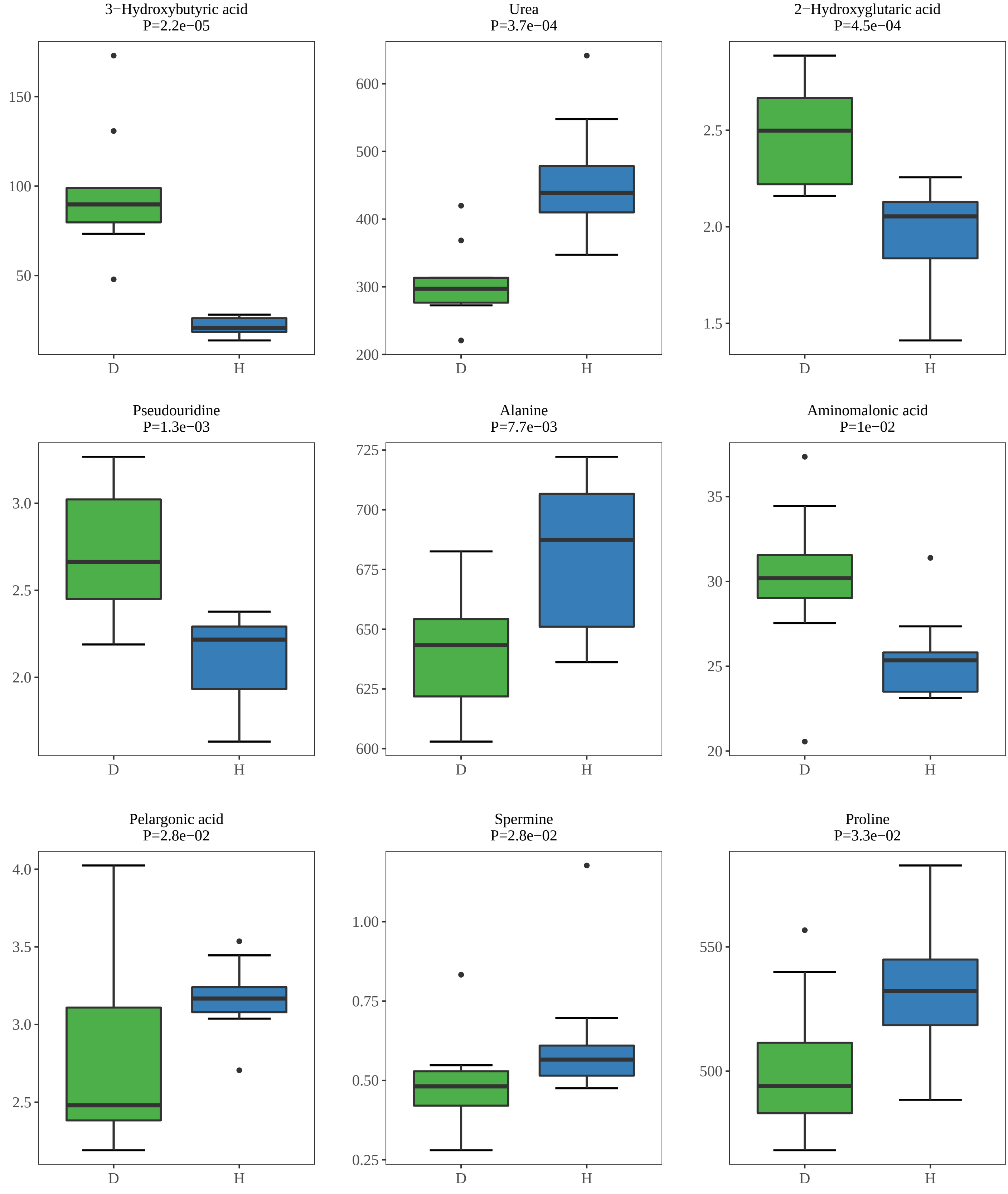

3−Hydroxybutyric acid
P=2.2e−05
Urea
P=3.7e−04
2−Hydroxyglutaric acid
P=4.5e−04
Pseudouridine
P=1.3e−03
Alanine
P=7.7e−03
Aminomalonic acid
P=1e−02
Pelargonic acid
P=2.8e−02
Spermine
P=2.8e−02
Proline
P=3.3e−02
D
H

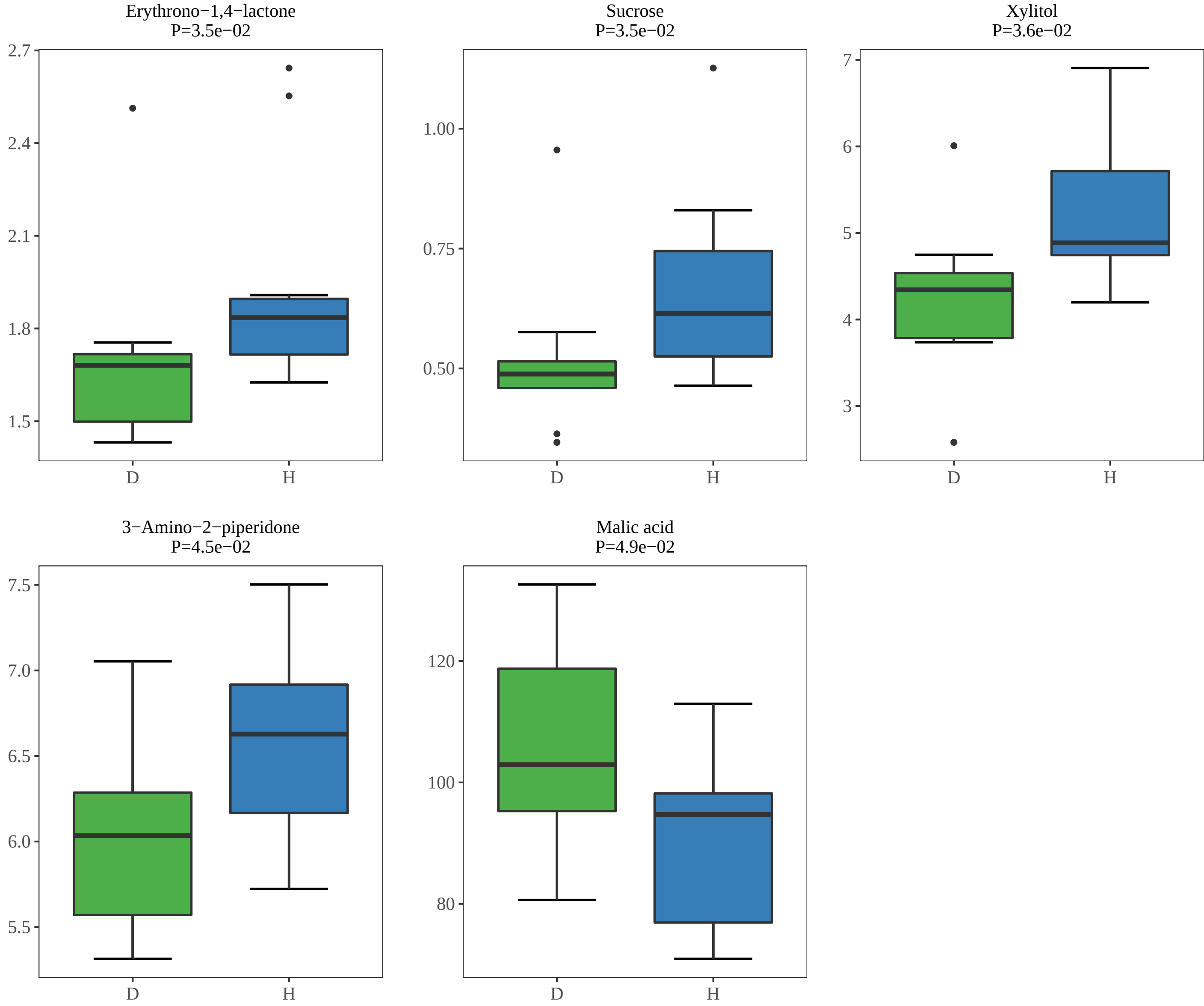

Erythrono−1,4−lactone
P=3.5e−02
Sucrose
P=3.5e−02
Xylitol
P=3.6e−02
3−Amino−2−piperidone
P=4.5e−02
Malic acid
P=4.9e−02
D
H
