# Supplementary material for: Yiai Fuzheng decoction inhibits triple-negative breast cancer by remodeling the immune microenvironment
Source: Front Immunol. 2025 Sep 30;16:1615631. doi: 10.3389/fimmu.2025.1615631 (PMC12518410; doi:10.3389/fimmu.2025.1615631)
Supplement: Supplementary file 2 [file DataSheet1.zip › Supplementary File 2/Treatment/D_vs_H/05_Univariate_Analysis/AllMet_Vioplot.pdf]

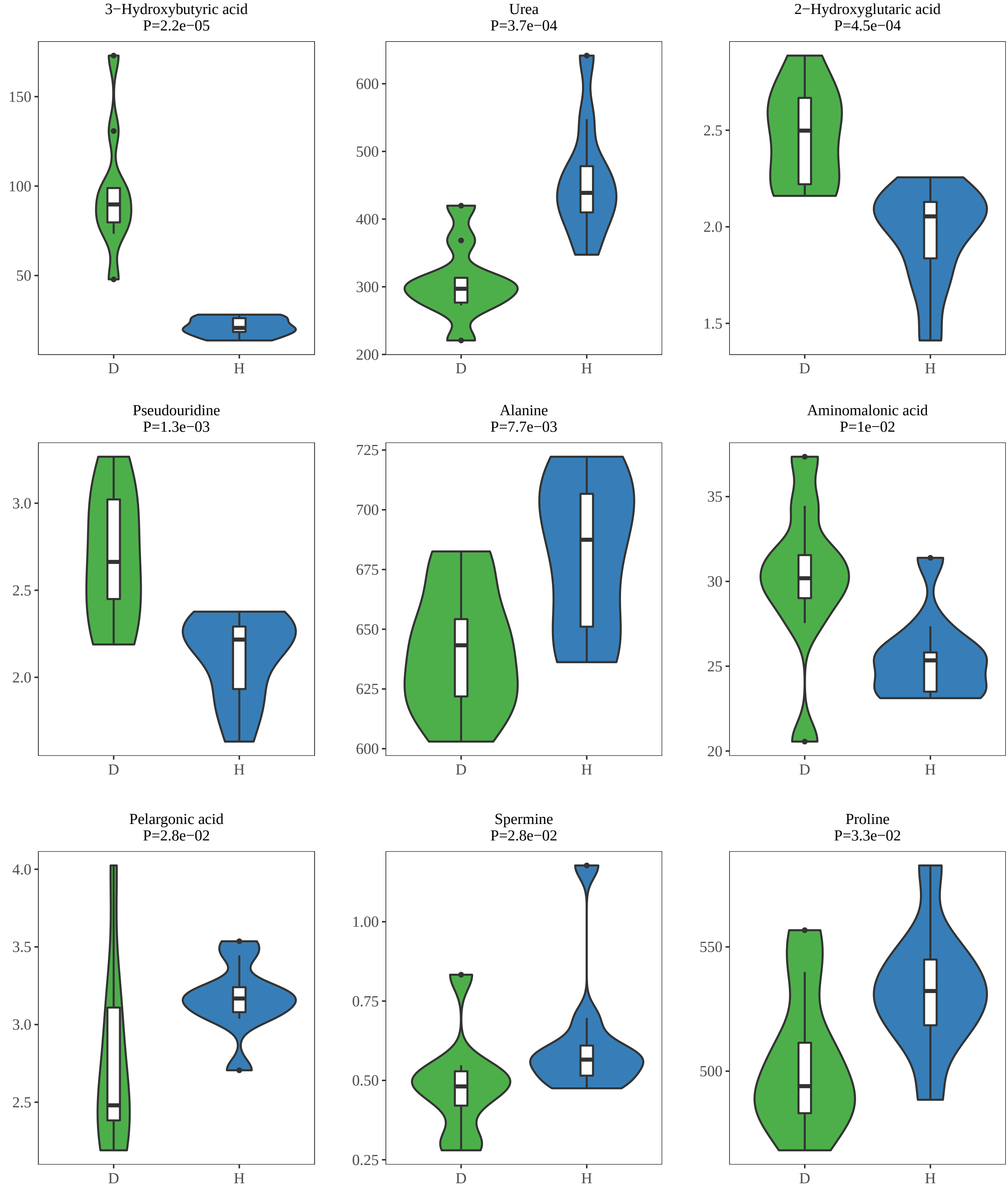
3−Hydroxybutyric acid
P=2.2e−05
150
100
50
D
H
Urea
P=3.7e−04
600
500
400
300
200
D
H
2−Hydroxyglutaric acid
P=4.5e−04
2.5
2.0
1.5
D
H
Pseudouridine
P=1.3e−03
3.0
2.5
2.0
D
H
Alanine
P=7.7e−03
725
700
675
650
625
600
D
H
Aminomalonic acid
P=1e−02
35
30
25
20
D
H
Pelargonic acid
P=2.8e−02
4.0
3.5
3.0
2.5
D
H
Spermine
P=2.8e−02
1.00
0.75
0.50
0.25
D
H
Proline
P=3.3e−02
550
500
D
H

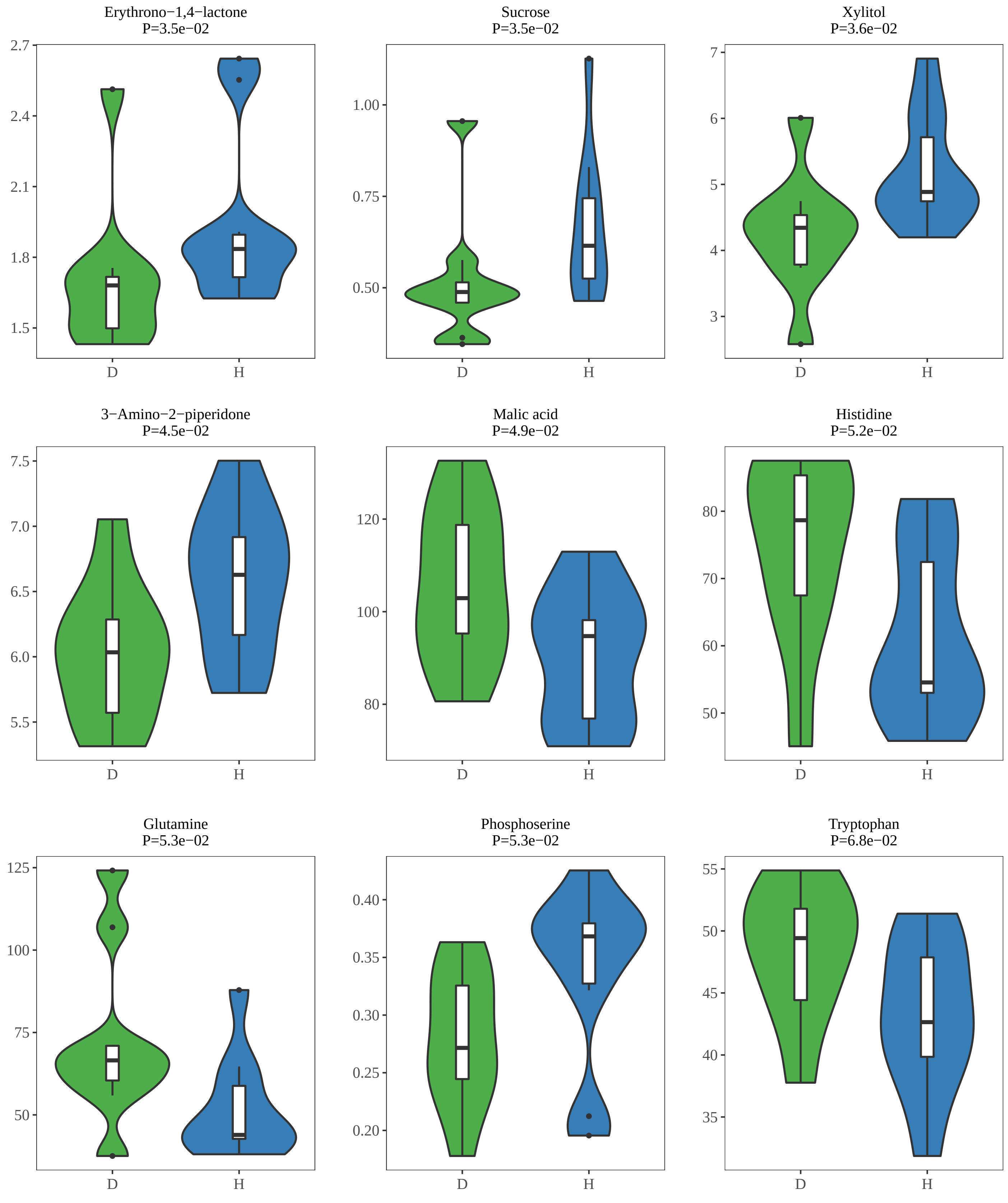

Erythrono−1,4−lactone
P=3.5e−02
Sucrose
P=3.5e−02
Xylitol
P=3.6e−02
3−Amino−2−piperidone
P=4.5e−02
Malic acid
P=4.9e−02
Histidine
P=5.2e−02
Glutamine
P=5.3e−02
Phosphoserine
P=5.3e−02
Tryptophan
P=6.8e−02
D
H

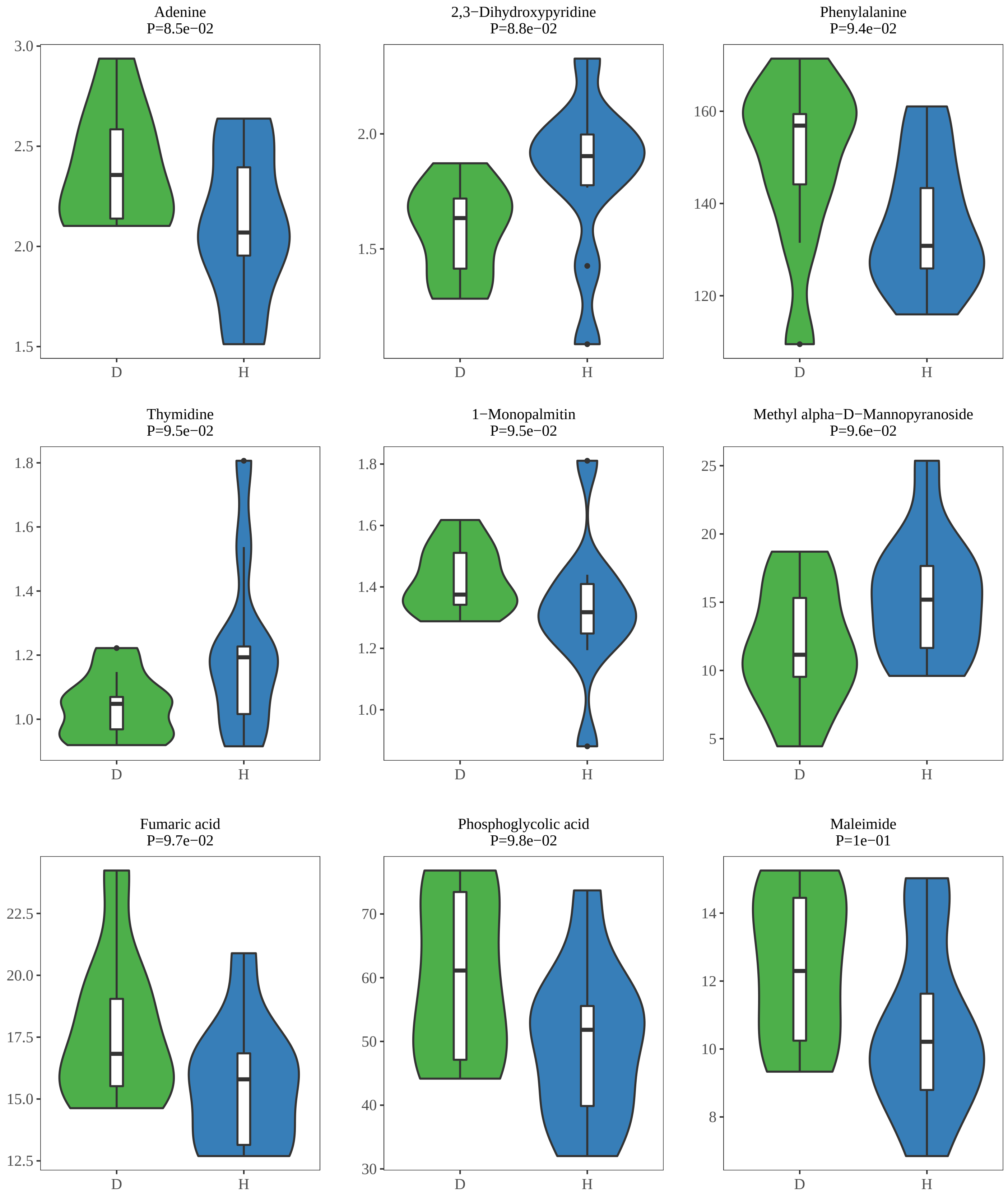
Adenine
P=8.5e−02
2,3−Dihydroxypyridine
P=8.8e−02
Phenylalanine
P=9.4e−02
Thymidine
P=9.5e−02
1−Monopalmitin
P=9.5e−02
Methyl alpha−D−Mannopyranoside
P=9.6e−02
Fumaric acid
P=9.7e−02
Phosphoglycolic acid
P=9.8e−02
Maleimide
P=1e−01
D
H

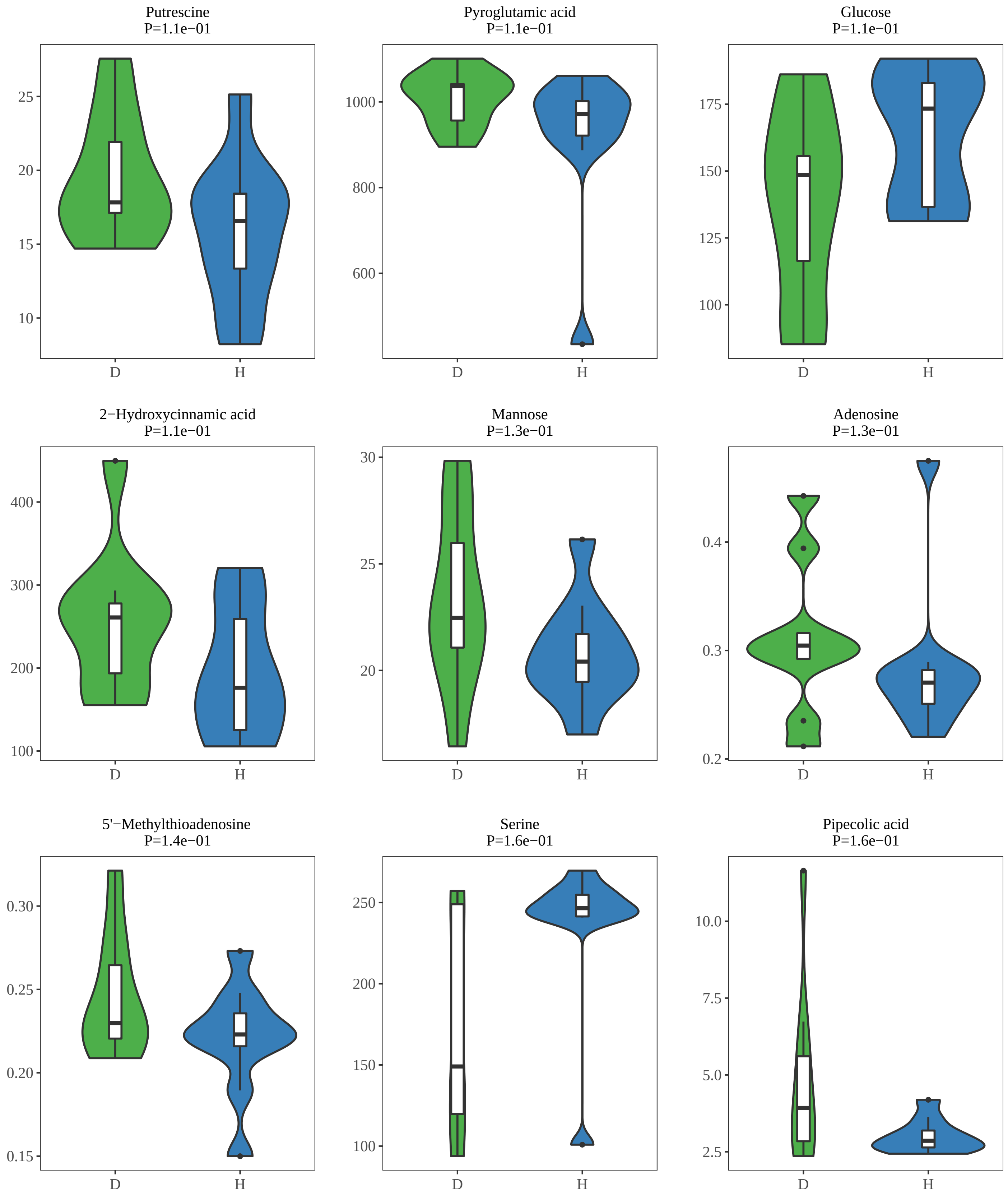
Putrescine
P=1.1e−01
Pyroglutamic acid
P=1.1e−01
Glucose
P=1.1e−01
2−Hydroxycinnamic acid
P=1.1e−01
Mannose
P=1.3e−01
Adenosine
P=1.3e−01
5'−Methylthioadenosine
P=1.4e−01
Serine
P=1.6e−01
Pipecolic acid
P=1.6e−01
D
H

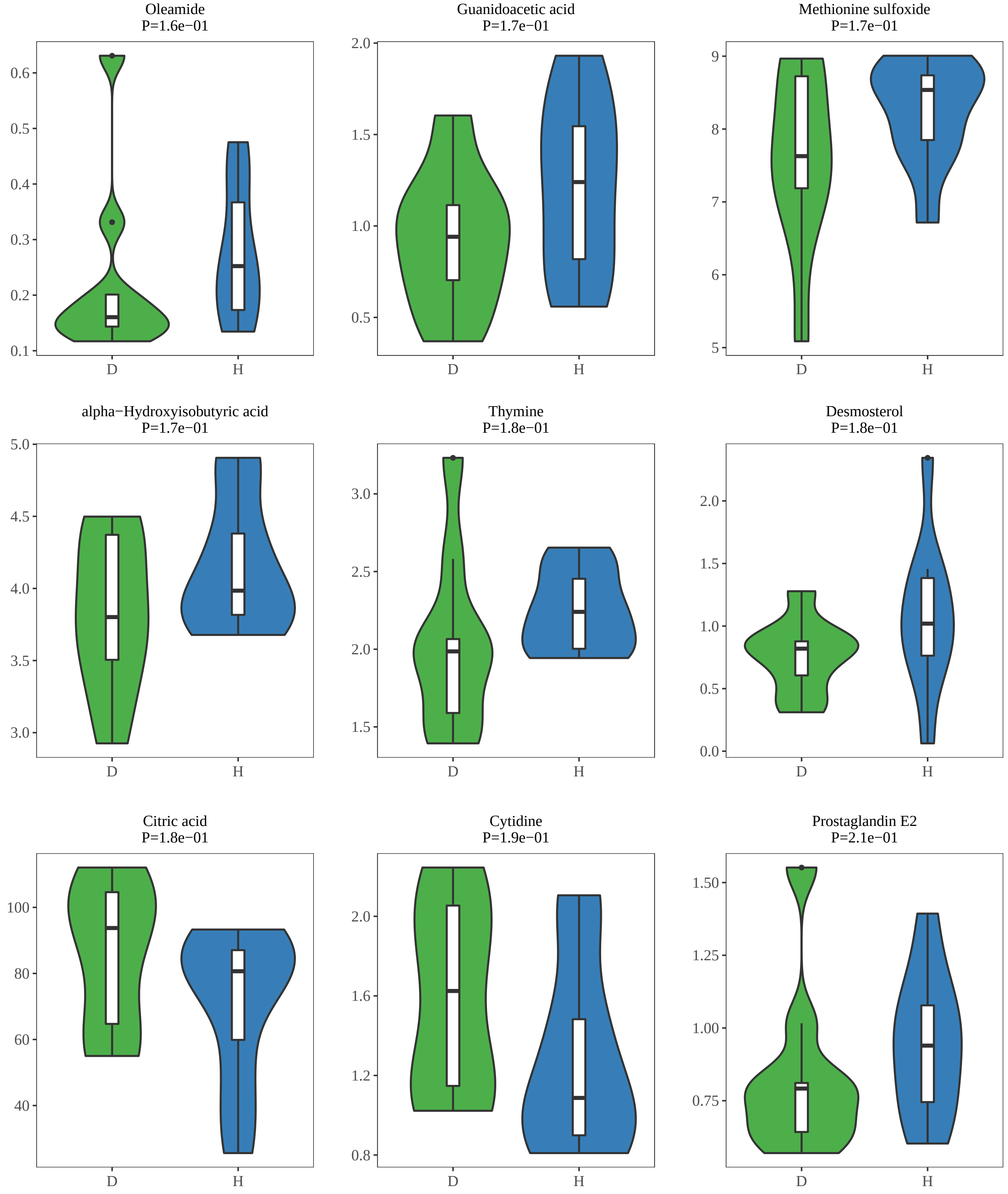
Oleamide
P=1.6e−01
Guanidoacetic acid
P=1.7e−01
Methionine sulfoxide
P=1.7e−01
alpha−Hydroxyisobutyric acid
P=1.7e−01
Thymine
P=1.8e−01
Desmosterol
P=1.8e−01
Citric acid
P=1.8e−01
Cytidine
P=1.9e−01
Prostaglandin E2
P=2.1e−01
D
H

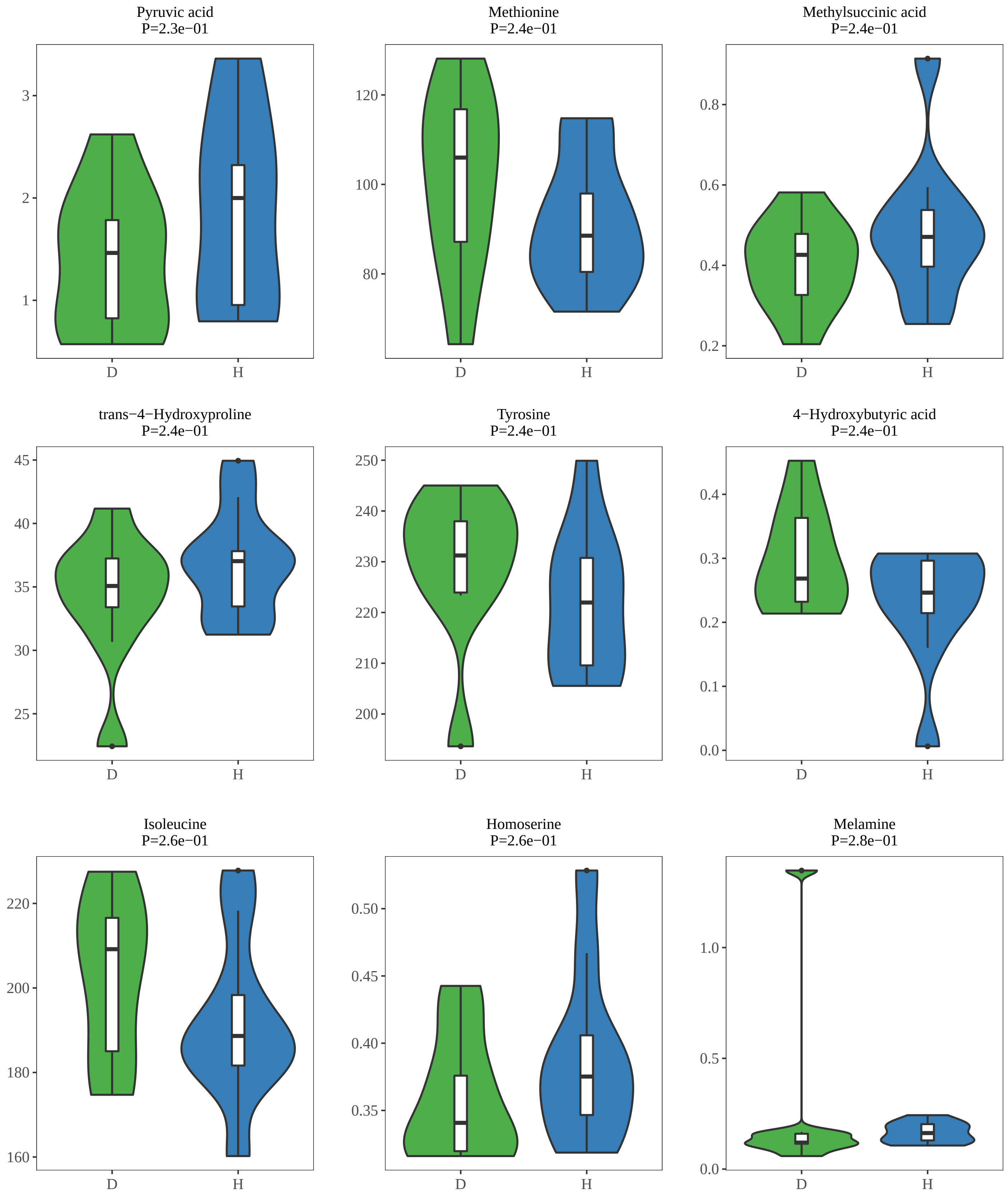

Pyruvic acid
P=2.3e−01
3
2
1
D
H
Methionine
P=2.4e−01
120
100
80
D
H
Methylsuccinic acid
P=2.4e−01
0.8
0.6
0.4
0.2
D
H
trans−4−Hydroxyproline
P=2.4e−01
45
40
35
30
25
D
H
Tyrosine
P=2.4e−01
250
240
230
220
210
200
D
H
4−Hydroxybutyric acid
P=2.4e−01
0.4
0.3
0.2
0.1
0.0
D
H
Isoleucine
P=2.6e−01
220
200
180
160
D
H
Homoserine
P=2.6e−01
0.50
0.45
0.40
0.35
D
H
Melamine
P=2.8e−01
1.0
0.5
0.0
D
H

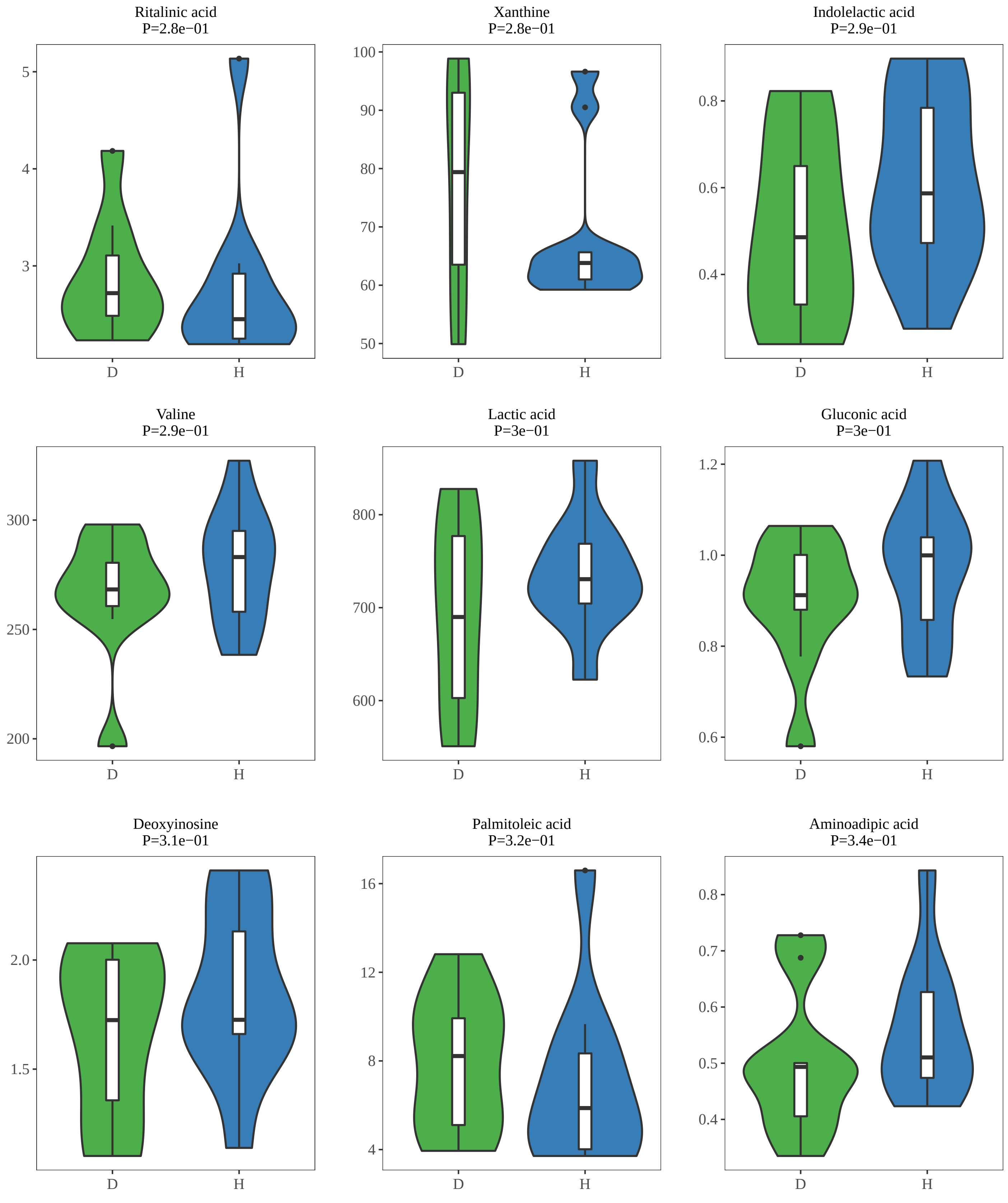

Ritalinic acid
P=2.8e−01
Xanthine
P=2.8e−01
Indolelactic acid
P=2.9e−01
Valine
P=2.9e−01
Lactic acid
P=3e−01
Gluconic acid
P=3e−01
Deoxyinosine
P=3.1e−01
Palmitoleic acid
P=3.2e−01
Aminoadipic acid
P=3.4e−01
D
H

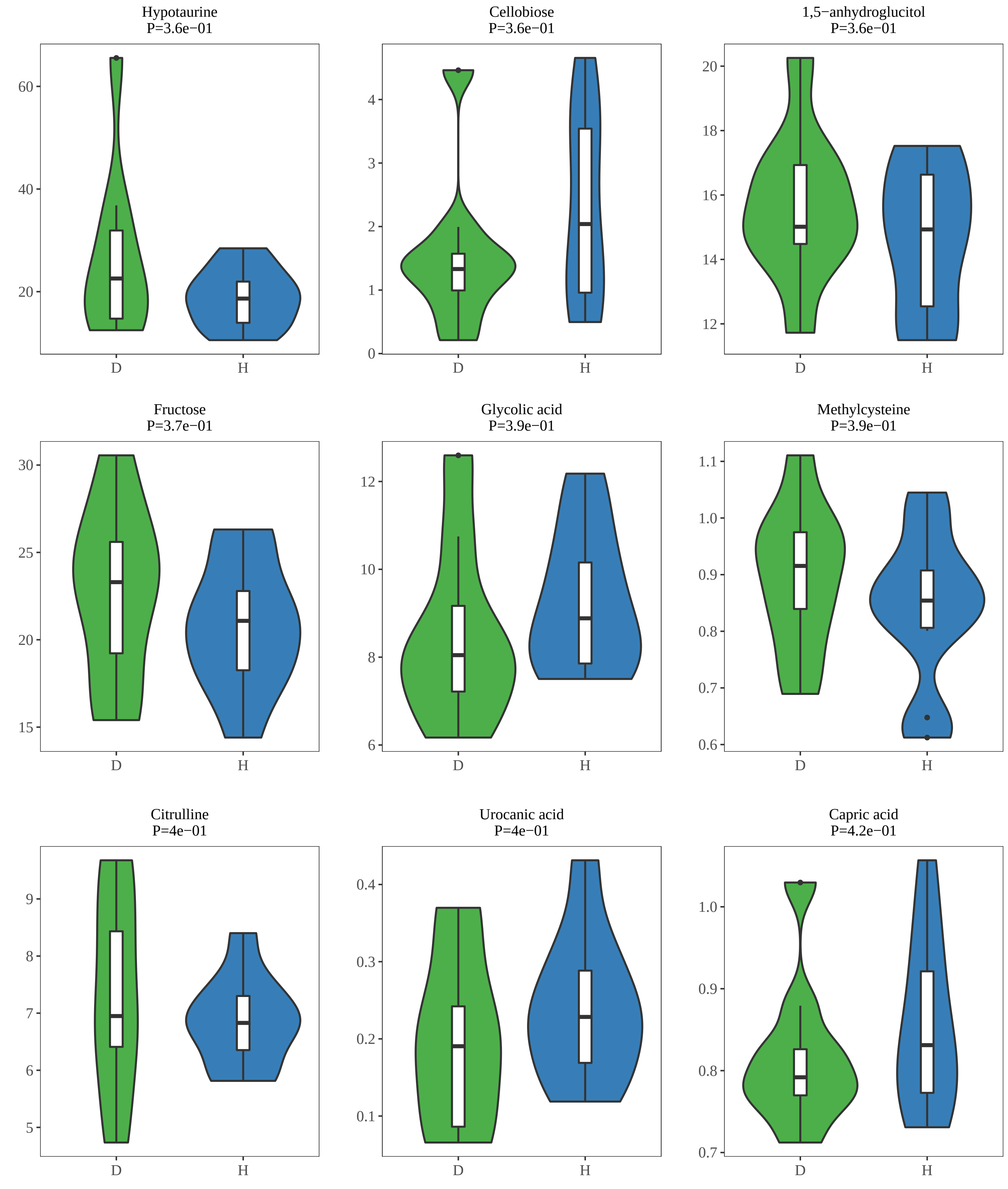
Hypotaurine
P=3.6e−01
60
40
20
D
H
Cellobiose
P=3.6e−01
4
3
2
1
0
D
H
1,5−anhydroglucitol
P=3.6e−01
20
18
16
14
12
D
H
Fructose
P=3.7e−01
30
25
20
15
D
H
Glycolic acid
P=3.9e−01
12
10
8
6
D
H
Methylcysteine
P=3.9e−01
1.1
1.0
0.9
0.8
0.7
0.6
D
H
Citrulline
P=4e−01
9
8
7
6
5
D
H
Urocanic acid
P=4e−01
0.4
0.3
0.2
0.1
D
H
Capric acid
P=4.2e−01
1.0
0.9
0.8
0.7
D
H

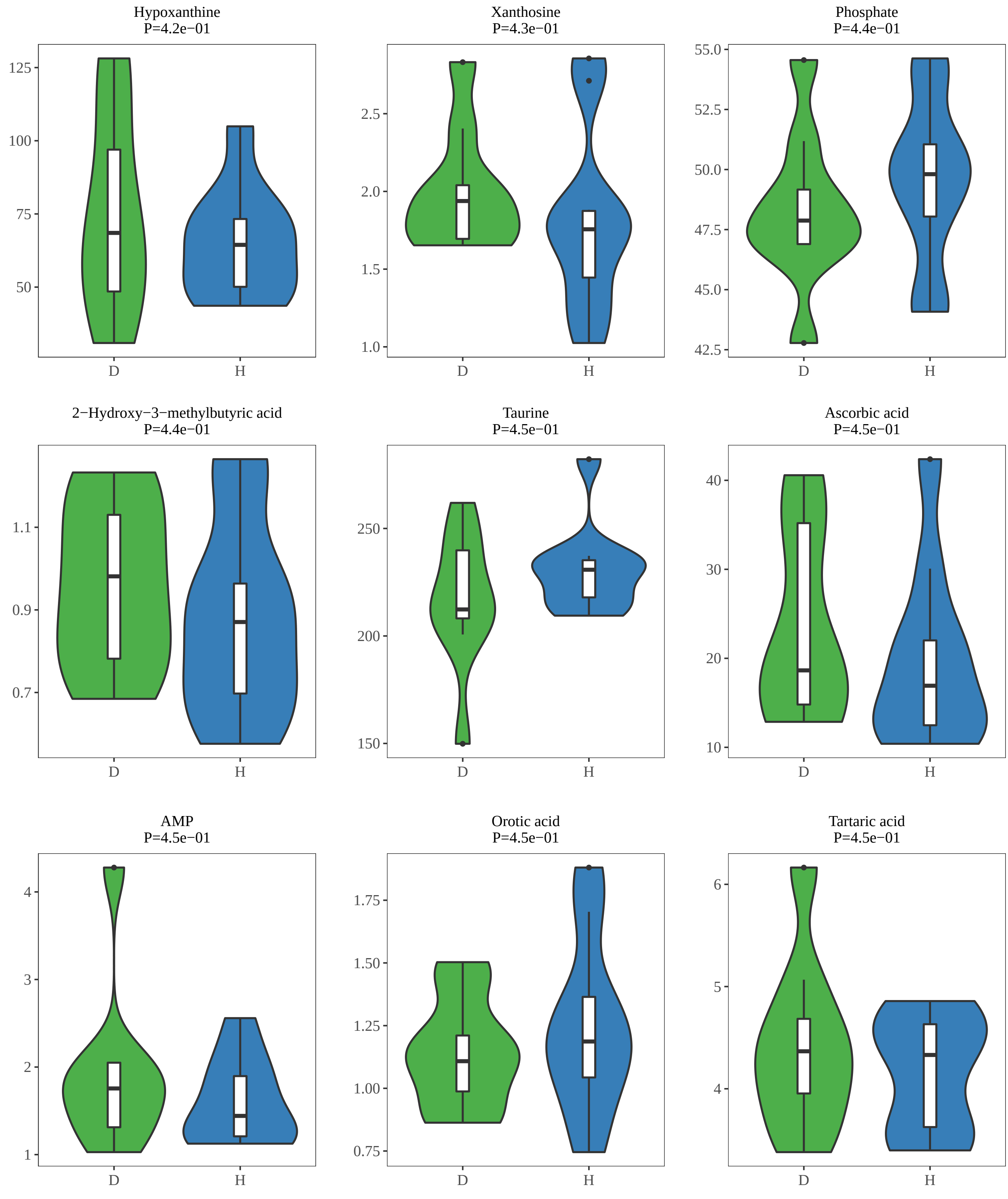
Hypoxanthine
P=4.2e−01
125
100
75
50
D
H
Xanthosine
P=4.3e−01
2.5
2.0
1.5
1.0
D
H
Phosphate
P=4.4e−01
55.0
52.5
50.0
47.5
45.0
42.5
D
H
2−Hydroxy−3−methylbutyric acid
P=4.4e−01
1.1
0.9
0.7
D
H
Taurine
P=4.5e−01
250
200
150
D
H
Ascorbic acid
P=4.5e−01
40
30
20
10
D
H
AMP
P=4.5e−01
4
3
2
1
D
H
Orotic acid
P=4.5e−01
1.75
1.50
1.25
1.00
0.75
D
H
Tartaric acid
P=4.5e−01
6
5
4
D
H

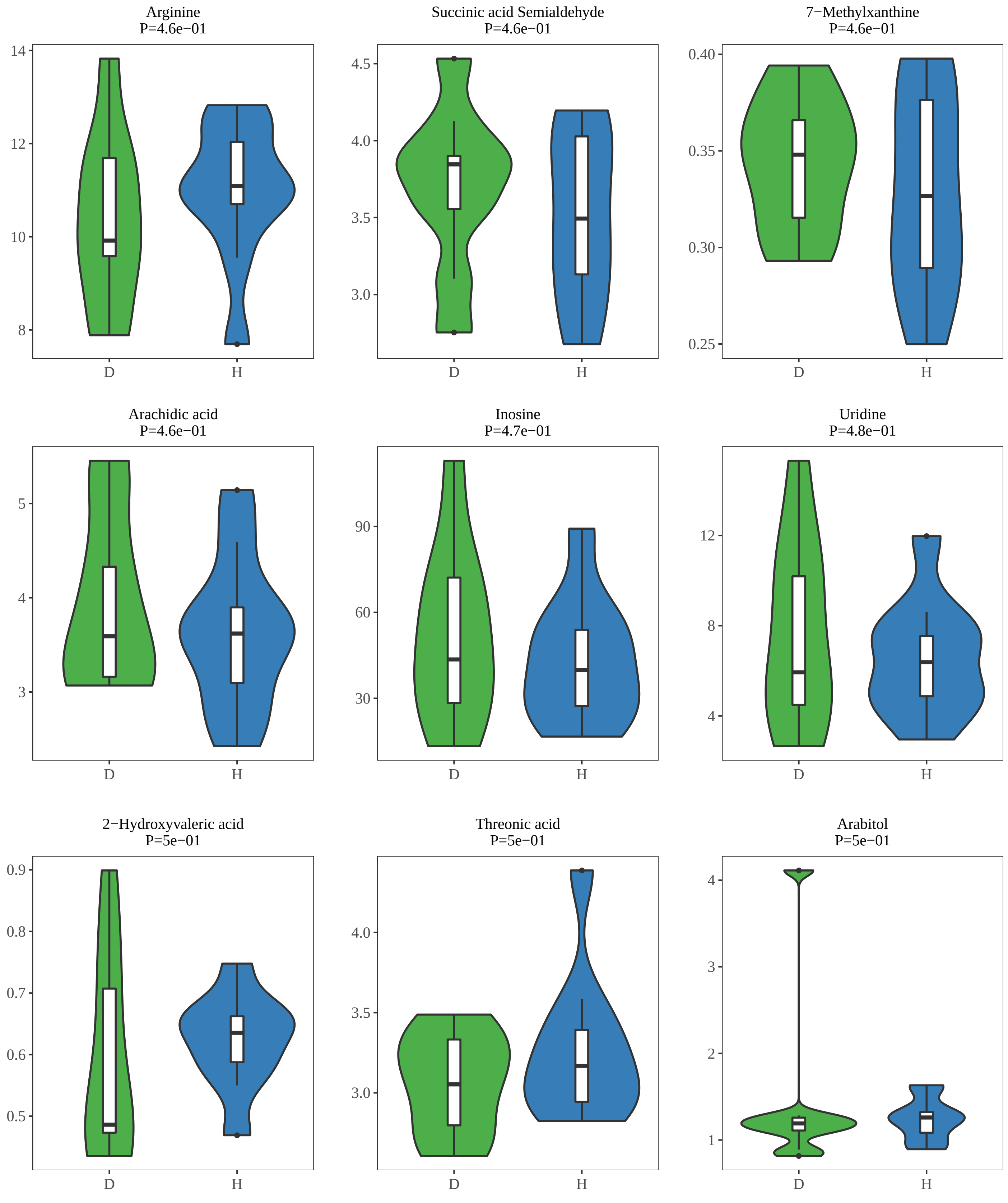

Arginine
P=4.6e−01
Succinic acid Semialdehyde
P=4.6e−01
7−Methylxanthine
P=4.6e−01
Arachidic acid
P=4.6e−01
Inosine
P=4.7e−01
Uridine
P=4.8e−01
2−Hydroxyvaleric acid
P=5e−01
Threonic acid
P=5e−01
Arabitol
P=5e−01
D
H

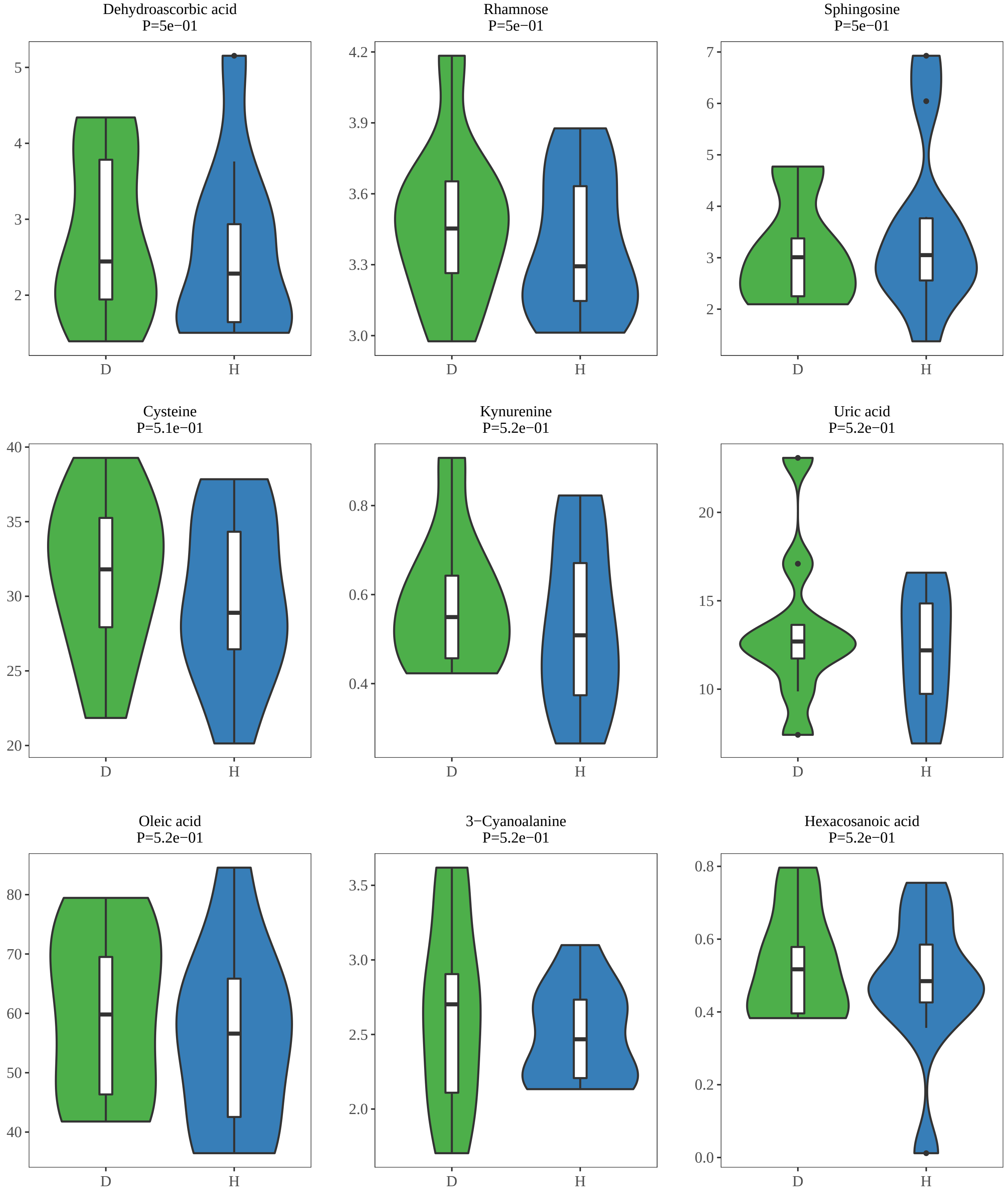
Dehydroascorbic acid
P=5e−01
Rhamnose
P=5e−01
Sphingosine
P=5e−01
Cysteine
P=5.1e−01
Kynurenine
P=5.2e−01
Uric acid
P=5.2e−01
Oleic acid
P=5.2e−01
3−Cyanoalanine
P=5.2e−01
Hexacosanoic acid
P=5.2e−01
D
H

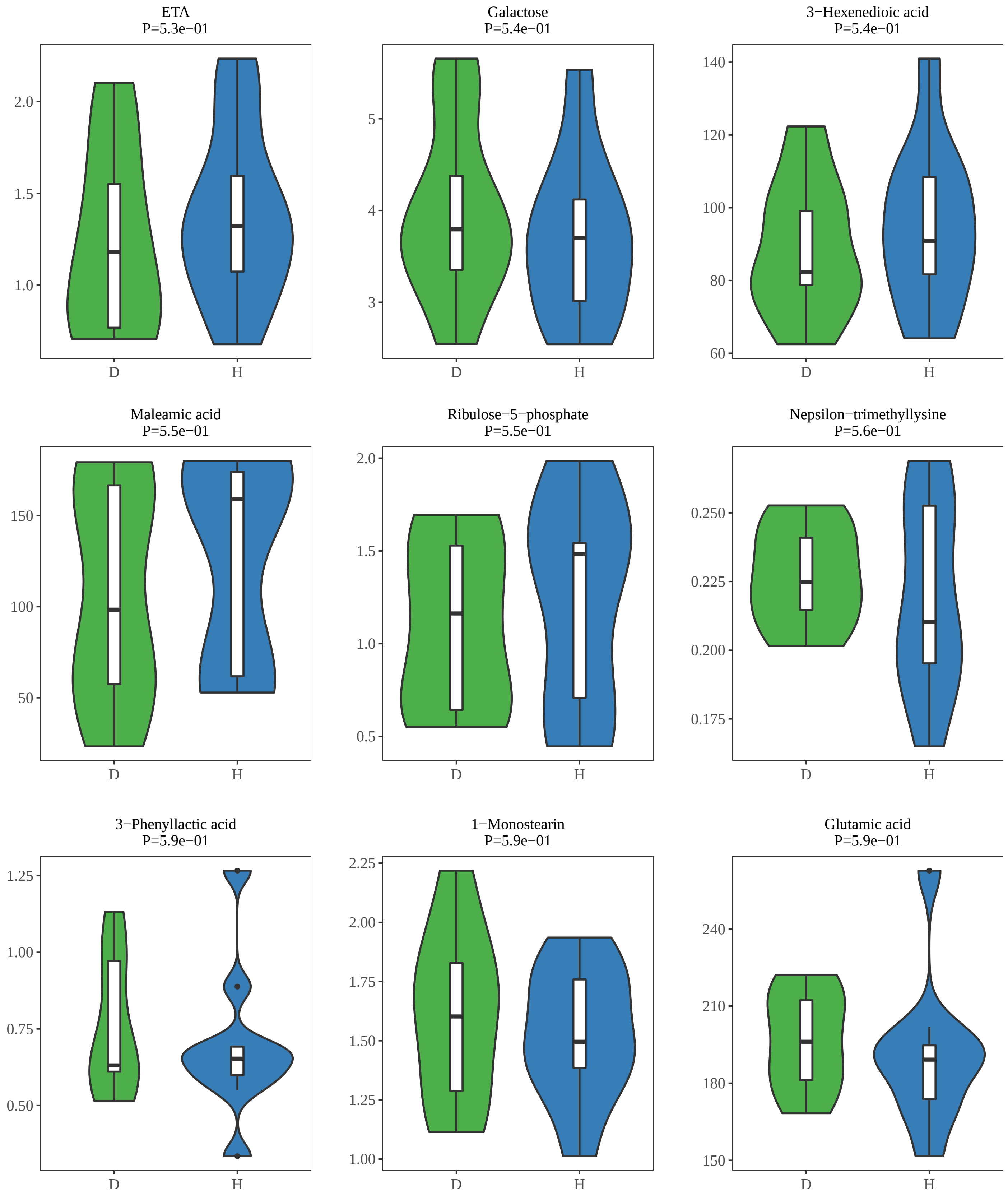

ETA
P=5.3e−01
2.0
1.5
1.0
D
H
Galactose
P=5.4e−01
5
4
3
D
H
3−Hexenedioic acid
P=5.4e−01
140
120
100
80
60
D
H
Maleamic acid
P=5.5e−01
150
100
50
D
H
Ribulose−5−phosphate
P=5.5e−01
2.0
1.5
1.0
0.5
D
H
Nepsilon−trimethyllysine
P=5.6e−01
0.250
0.225
0.200
0.175
D
H
3−Phenyllactic acid
P=5.9e−01
1.25
1.00
0.75
0.50
D
H
1−Monostearin
P=5.9e−01
2.25
2.00
1.75
1.50
1.25
1.00
D
H
Glutamic acid
P=5.9e−01
240
210
180
150
D
H

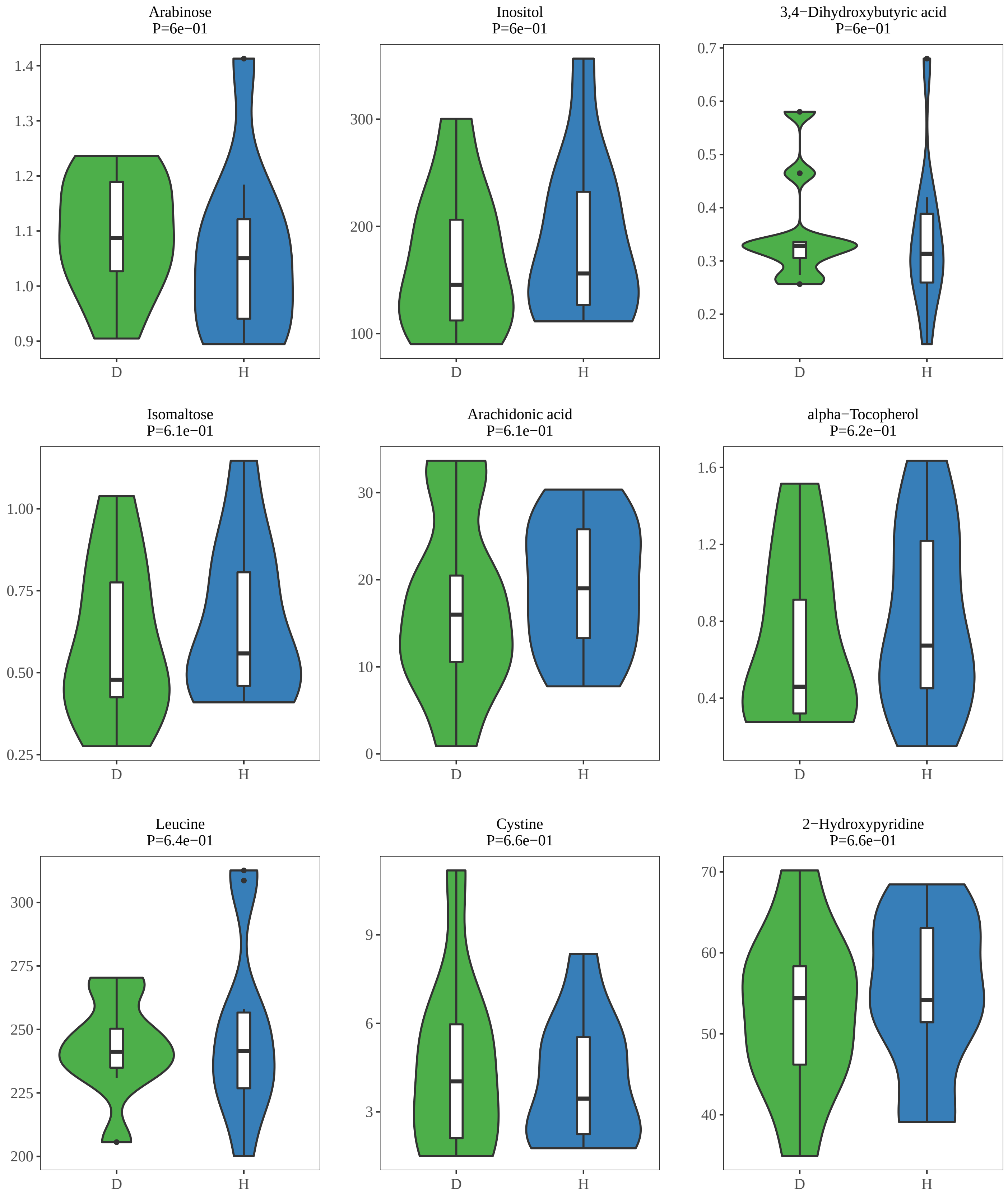

Arabinose
P=6e−01
1.4
1.3
1.2
1.1
1.0
0.9
D
H
Inositol
P=6e−01
300
200
100
D
H
3,4−Dihydroxybutyric acid
P=6e−01
0.7
0.6
0.5
0.4
0.3
0.2
D
H
Isomaltose
P=6.1e−01
1.00
0.75
0.50
0.25
D
H
Arachidonic acid
P=6.1e−01
30
20
10
0
D
H
alpha−Tocopherol
P=6.2e−01
1.6
1.2
0.8
0.4
D
H
Leucine
P=6.4e−01
300
275
250
225
200
D
H
Cystine
P=6.6e−01
9
6
3
D
H
2−Hydroxypyridine
P=6.6e−01
70
60
50
40
D
H

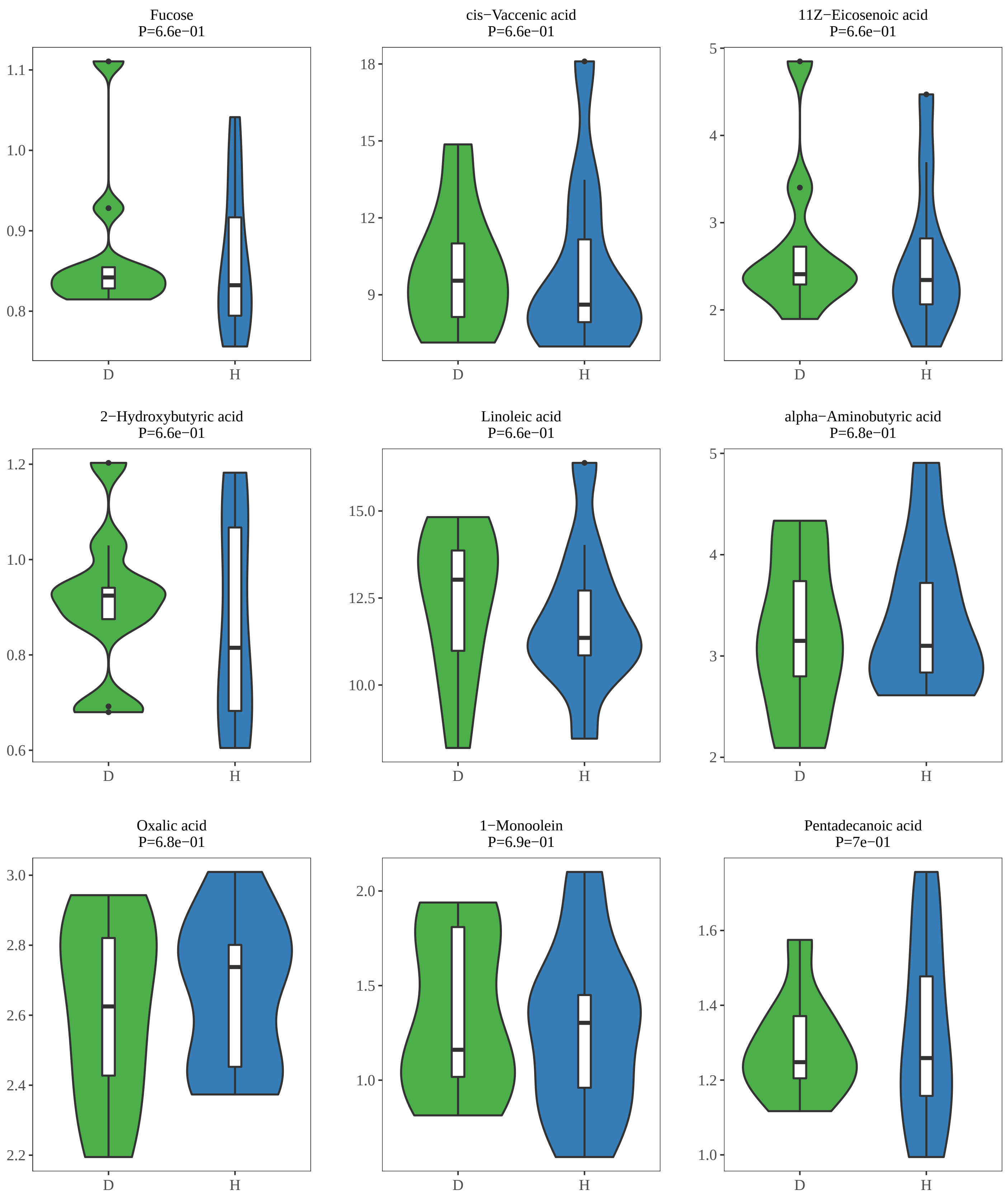

Fucose
P=6.6e−01
cis−Vaccenic acid
P=6.6e−01
11Z−Eicosenoic acid
P=6.6e−01
2−Hydroxybutyric acid
P=6.6e−01
Linoleic acid
P=6.6e−01
alpha−Aminobutyric acid
P=6.8e−01
Oxalic acid
P=6.8e−01
1−Monoolein
P=6.9e−01
Pentadecanoic acid
P=7e−01
D
H

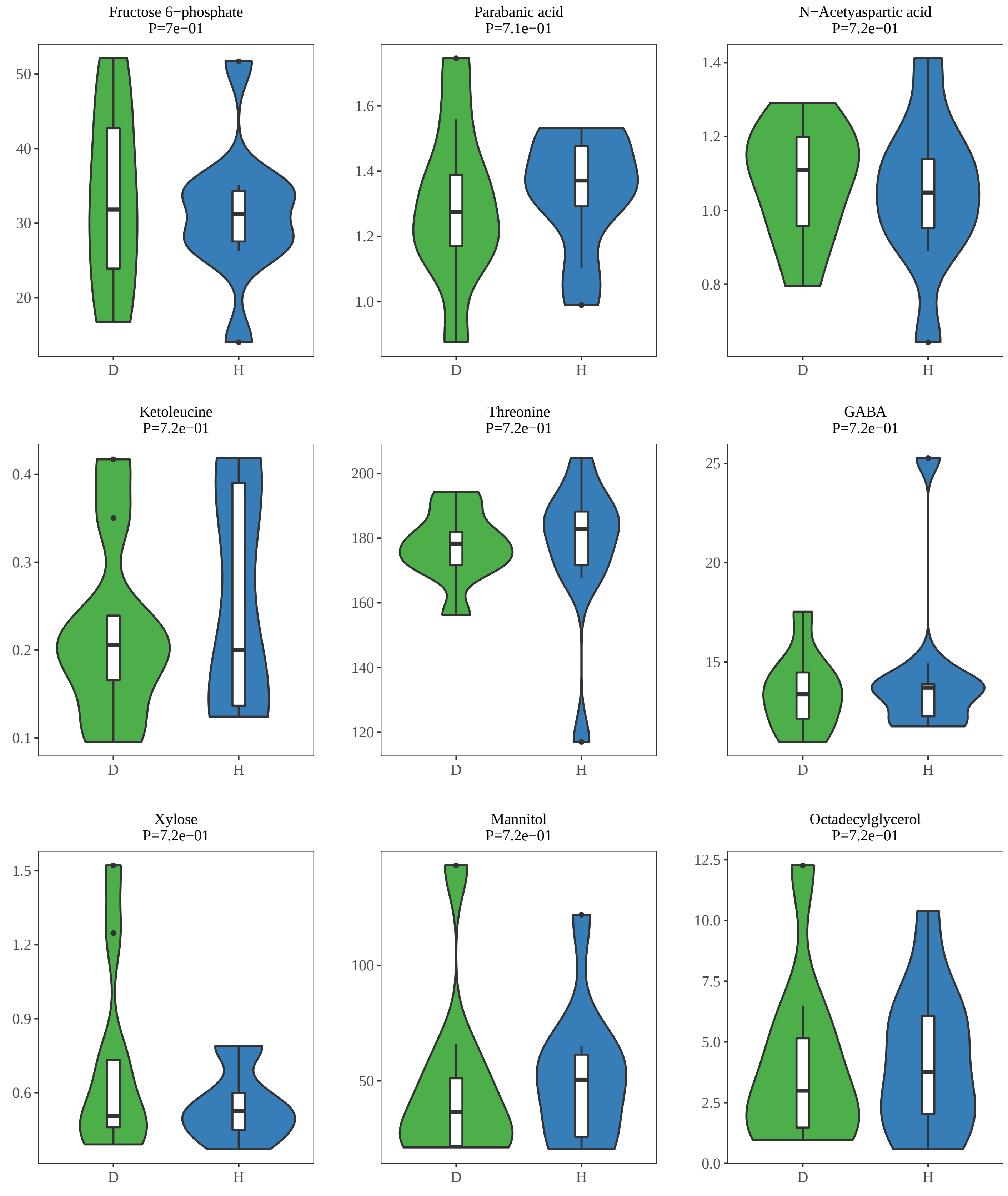

Fructose 6−phosphate
P=7e−01
Parabanic acid
P=7.1e−01
N−Acetyaspartic acid
P=7.2e−01
Ketoleucine
P=7.2e−01
Threonine
P=7.2e−01
GABA
P=7.2e−01
Xylose
P=7.2e−01
Mannitol
P=7.2e−01
Octadecylglycerol
P=7.2e−01
D
H

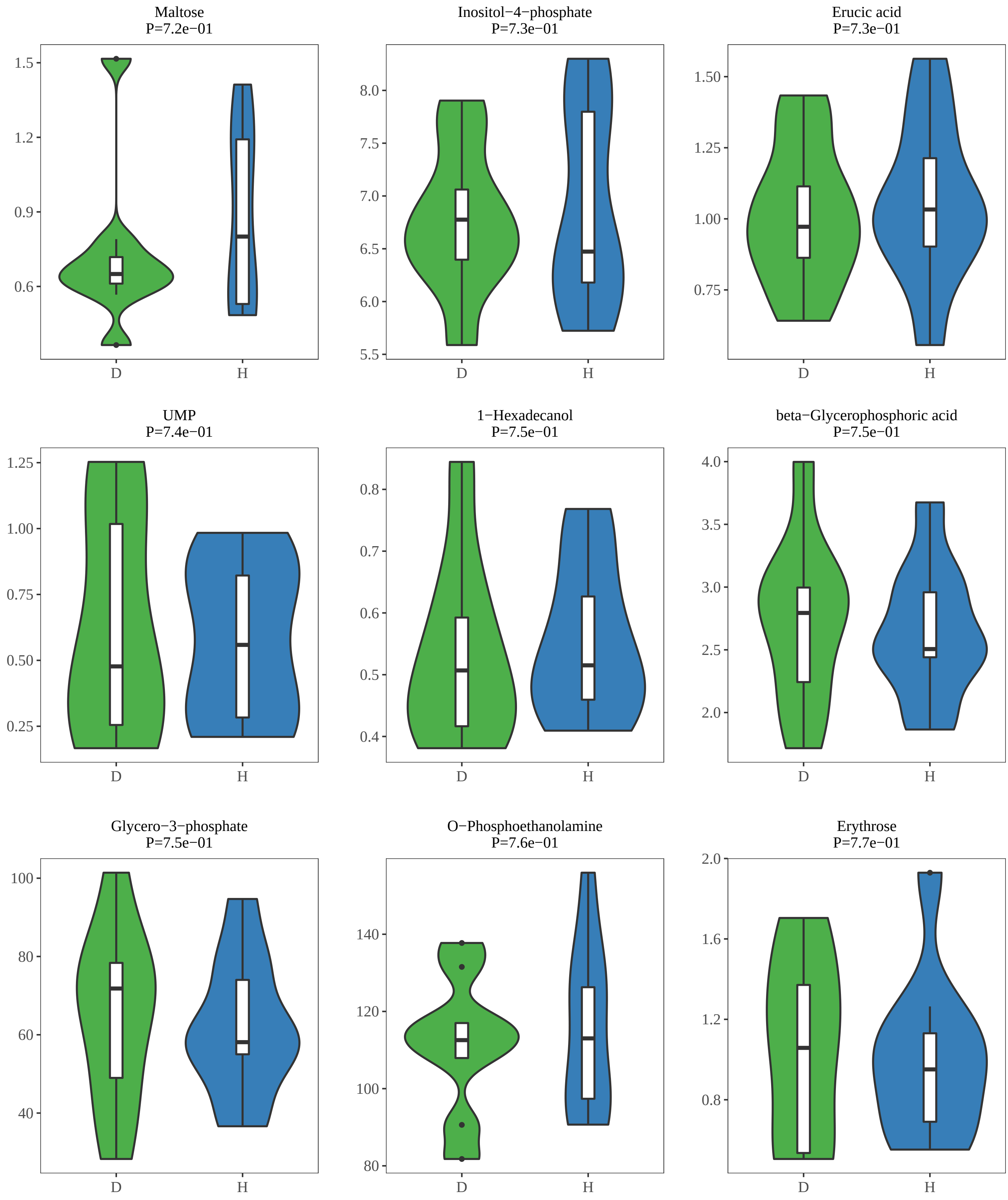
Maltose
P=7.2e−01
1.5
1.2
0.9
0.6
D
H
Inositol−4−phosphate
P=7.3e−01
8.0
7.5
7.0
6.5
6.0
5.5
D
H
Erucic acid
P=7.3e−01
1.50
1.25
1.00
0.75
D
H
UMP
P=7.4e−01
1.25
1.00
0.75
0.50
0.25
D
H
1−Hexadecanol
P=7.5e−01
0.8
0.7
0.6
0.5
0.4
D
H
beta−Glycerophosphoric acid
P=7.5e−01
4.0
3.5
3.0
2.5
2.0
D
H
Glycero−3−phosphate
P=7.5e−01
100
80
60
40
D
H
O−Phosphoethanolamine
P=7.6e−01
140
120
100
80
D
H
Erythrose
P=7.7e−01
2.0
1.6
1.2
0.8
D
H

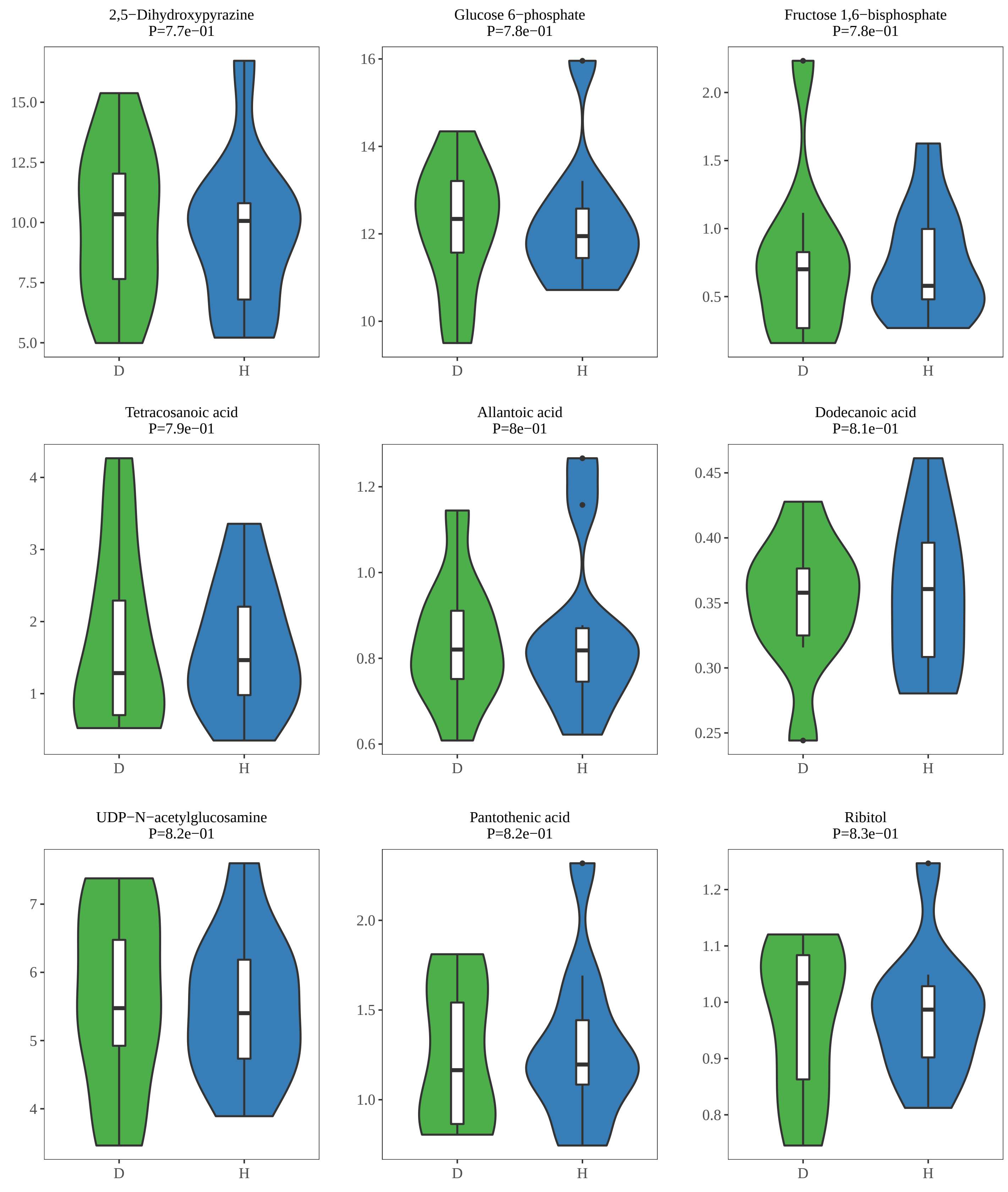

2,5−Dihydroxypyrazine
P=7.7e−01
15.0
12.5
10.0
7.5
5.0
D
H
Glucose 6−phosphate
P=7.8e−01
16
14
12
10
D
H
Fructose 1,6−bisphosphate
P=7.8e−01
2.0
1.5
1.0
0.5
D
H
Tetracosanoic acid
P=7.9e−01
4
3
2
1
D
H
Allantoic acid
P=8e−01
1.2
1.0
0.8
0.6
D
H
Dodecanoic acid
P=8.1e−01
0.45
0.40
0.35
0.30
0.25
D
H
UDP−N−acetylglucosamine
P=8.2e−01
7
6
5
4
D
H
Pantothenic acid
P=8.2e−01
2.0
1.5
1.0
D
H
Ribitol
P=8.3e−01
1.2
1.1
1.0
0.9
0.8
D
H

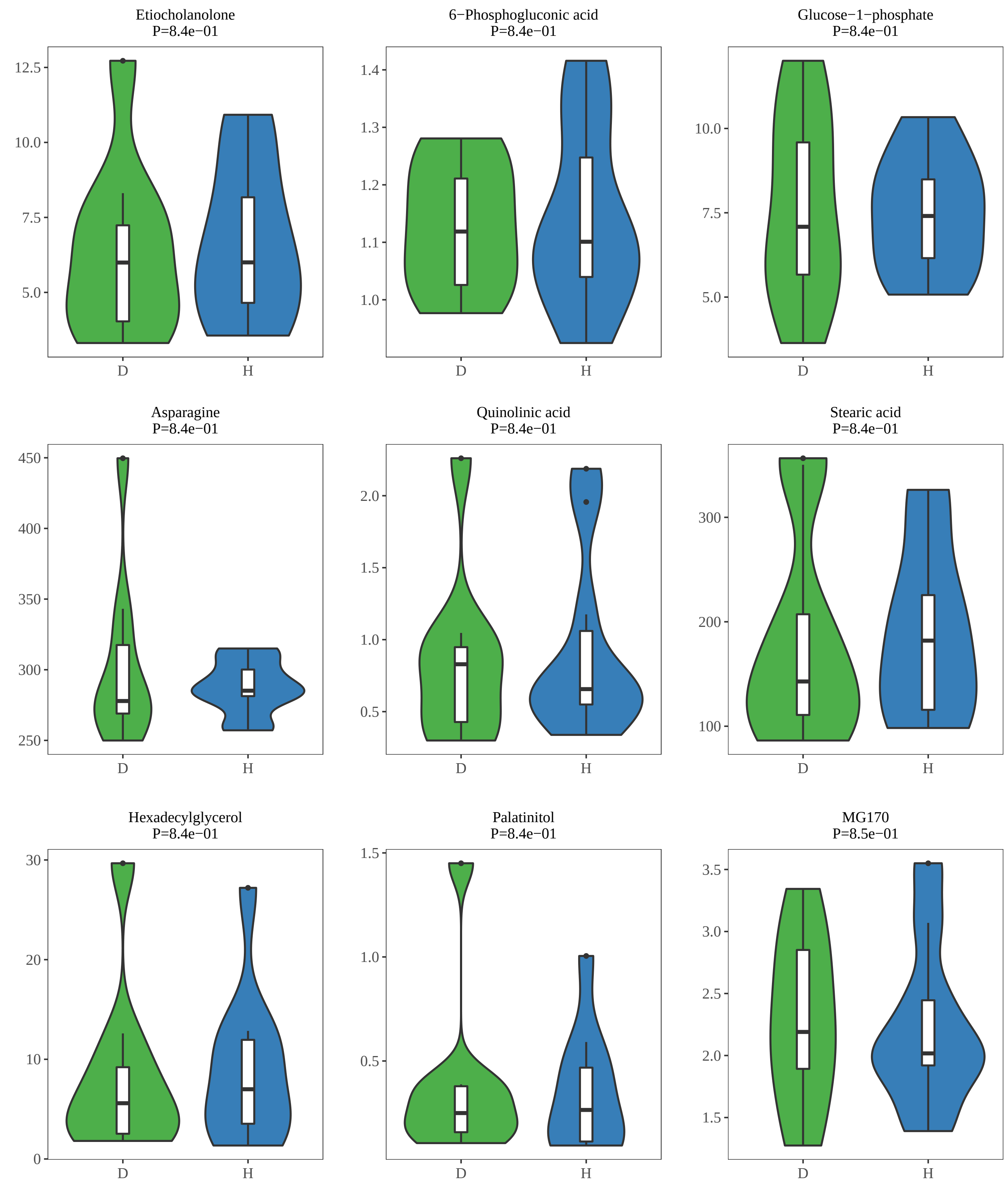

Etiocholanolone
P=8.4e−01
6−Phosphogluconic acid
P=8.4e−01
Glucose−1−phosphate
P=8.4e−01
Asparagine
P=8.4e−01
Quinolinic acid
P=8.4e−01
Stearic acid
P=8.4e−01
Hexadecylglycerol
P=8.4e−01
Palatinitol
P=8.4e−01
MG170
P=8.5e−01
D
H

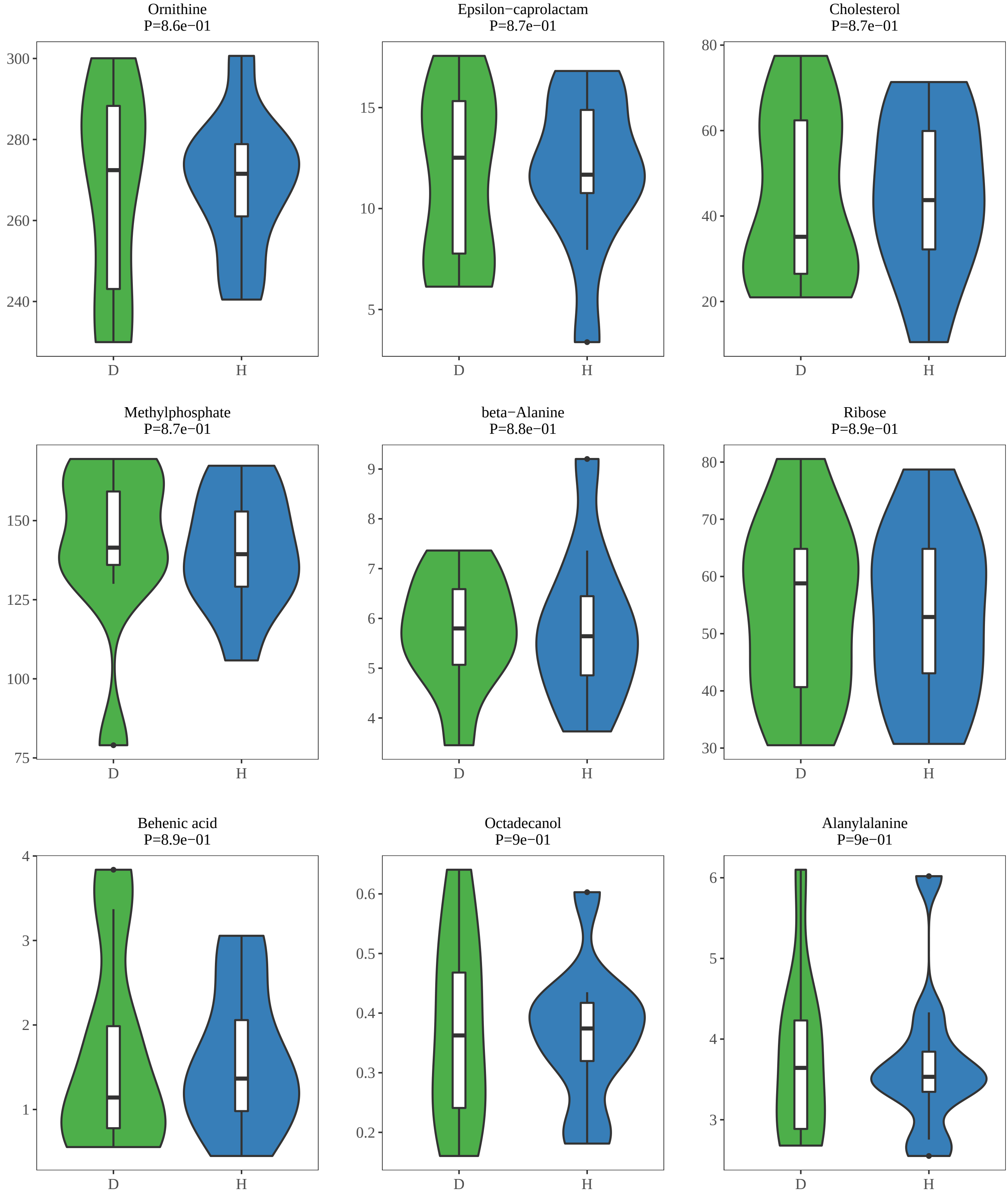
Ornithine
P=8.6e−01
300
280
260
240
D
H
Epsilon−caprolactam
P=8.7e−01
15
10
5
D
H
Cholesterol
P=8.7e−01
80
60
40
20
D
H
Methylphosphate
P=8.7e−01
150
125
100
75
D
H
beta−Alanine
P=8.8e−01
9
8
7
6
5
4
D
H
Ribose
P=8.9e−01
80
70
60
50
40
30
D
H
Behenic acid
P=8.9e−01
4
3
2
1
D
H
Octadecanol
P=9e−01
0.6
0.5
0.4
0.3
0.2
D
H
Alanylalanine
P=9e−01
6
5
4
3
D
H

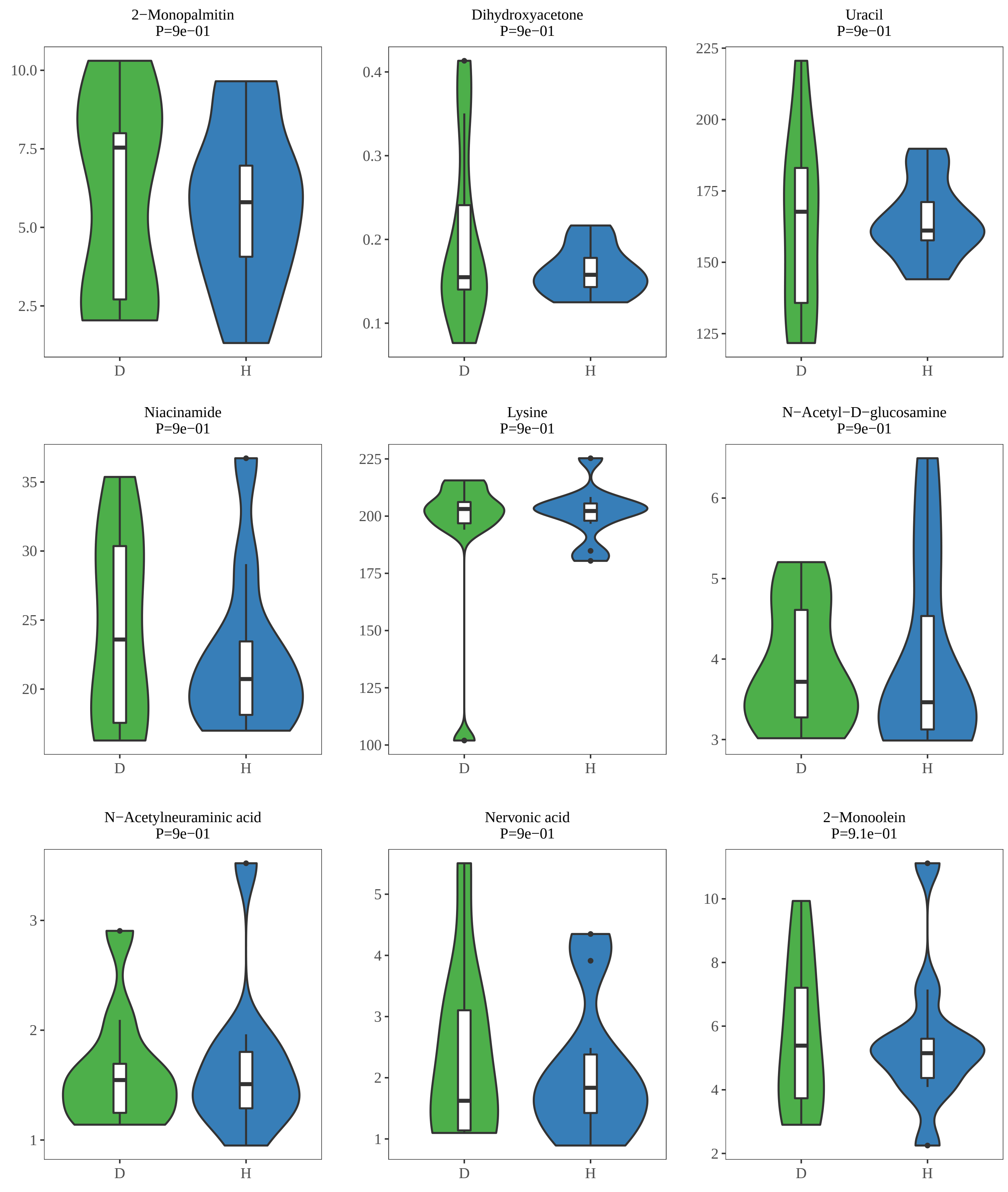
2−Monopalmitin
P=9e−01
10.0
7.5
5.0
2.5
D
H
Dihydroxyacetone
P=9e−01
0.4
0.3
0.2
0.1
D
H
Uracil
P=9e−01
225
200
175
150
125
D
H
Niacinamide
P=9e−01
35
30
25
20
D
H
Lysine
P=9e−01
225
200
175
150
125
100
D
H
N−Acetyl−D−glucosamine
P=9e−01
6
5
4
3
D
H
N−Acetylneuraminic acid
P=9e−01
3
2
1
D
H
Nervonic acid
P=9e−01
5
4
3
2
1
D
H
2−Monoolein
P=9.1e−01
10
8
6
4
2
D
H

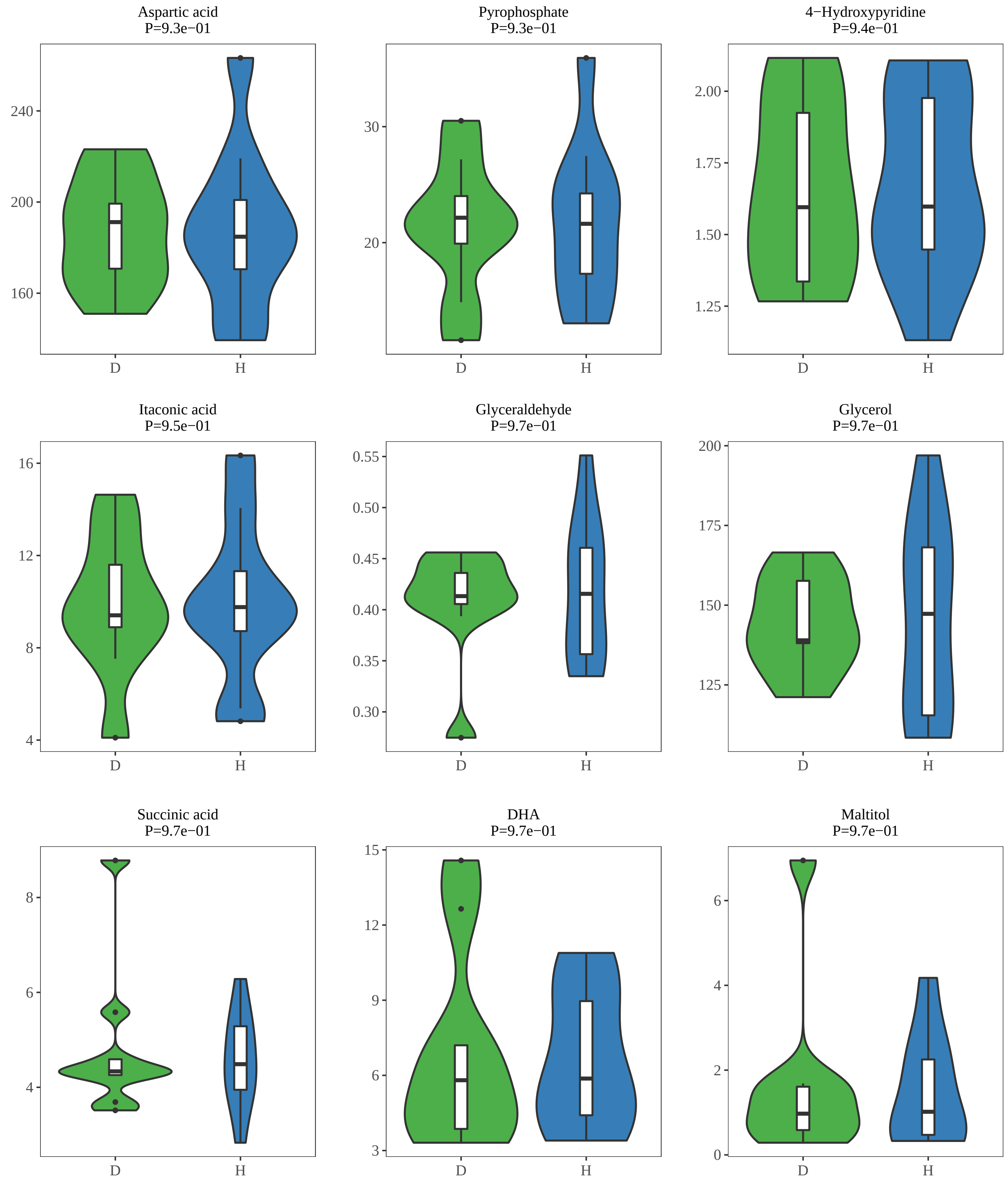

Aspartic acid
P=9.3e−01
240
200
160
D
H
Pyrophosphate
P=9.3e−01
30
20
D
H
4−Hydroxypyridine
P=9.4e−01
2.00
1.75
1.50
1.25
D
H
Itaconic acid
P=9.5e−01
16
12
8
4
D
H
Glyceraldehyde
P=9.7e−01
0.55
0.50
0.45
0.40
0.35
0.30
D
H
Glycerol
P=9.7e−01
200
175
150
125
D
H
Succinic acid
P=9.7e−01
8
6
4
D
H
DHA
P=9.7e−01
15
12
9
6
3
D
H
Maltitol
P=9.7e−01
6
4
2
0
D
H

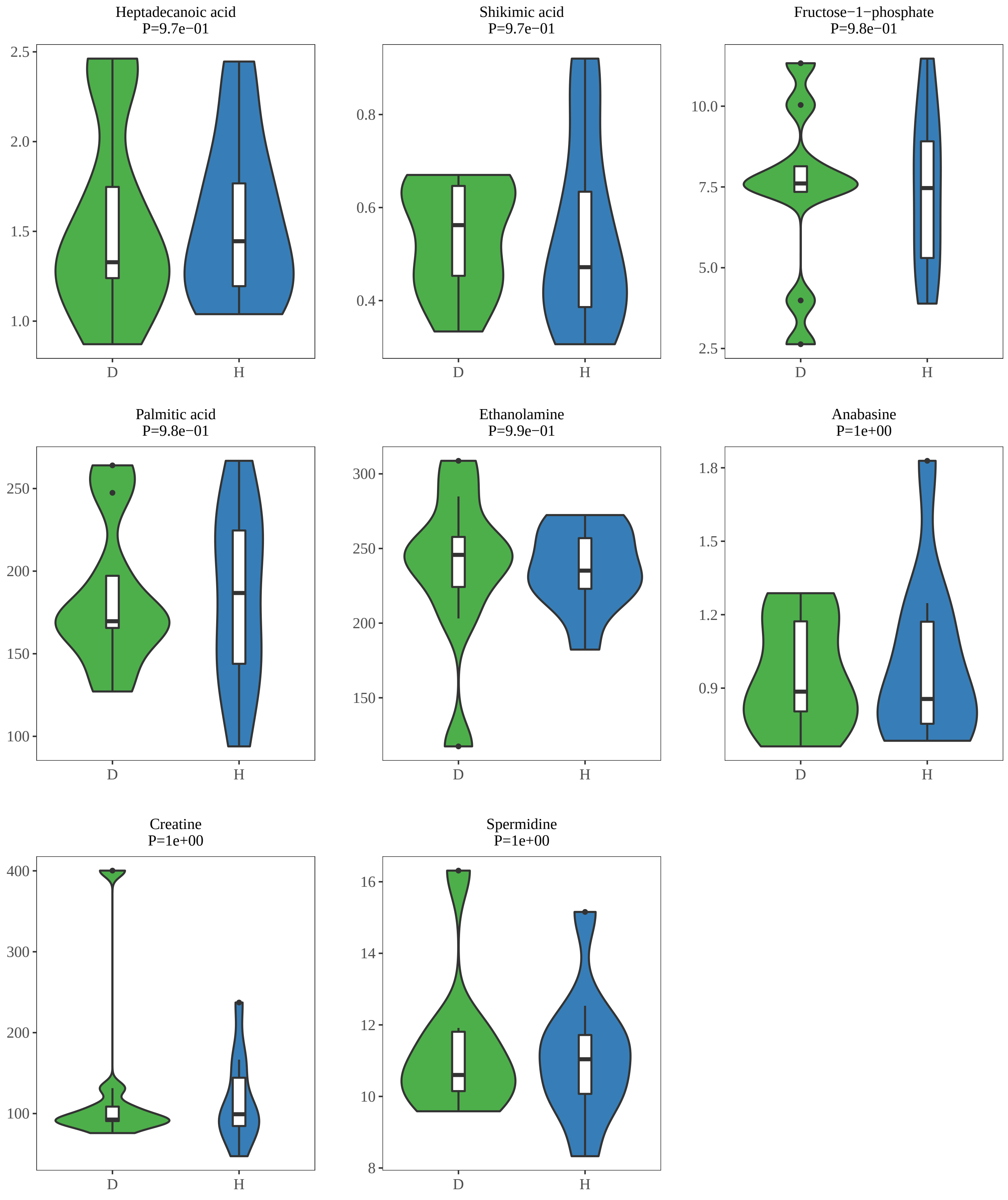
Heptadecanoic acid
P=9.7e−01
2.5
2.0
1.5
1.0
D
H
Shikimic acid
P=9.7e−01
0.8
0.6
0.4
D
H
Fructose−1−phosphate
P=9.8e−01
10.0
7.5
5.0
2.5
D
H
Palmitic acid
P=9.8e−01
250
200
150
100
D
H
Ethanolamine
P=9.9e−01
300
250
200
150
D
H
Anabasine
P=1e+00
1.8
1.5
1.2
0.9
D
H
Creatine
P=1e+00
400
300
200
100
D
H
Spermidine
P=1e+00
16
14
12
10
8
D
H
