# Supplementary material for: Yiai Fuzheng decoction inhibits triple-negative breast cancer by remodeling the immune microenvironment
Source: Front Immunol. 2025 Sep 30;16:1615631. doi: 10.3389/fimmu.2025.1615631 (PMC12518410; doi:10.3389/fimmu.2025.1615631)
Supplement: Supplementary file 2 [file DataSheet1.zip › Supplementary File 2/Treatment/C_vs_D_vs_H/04_Univariate_Analysis/AllMet_Boxplot_with_Points_and_Significant_labels_post_hoc.pdf]

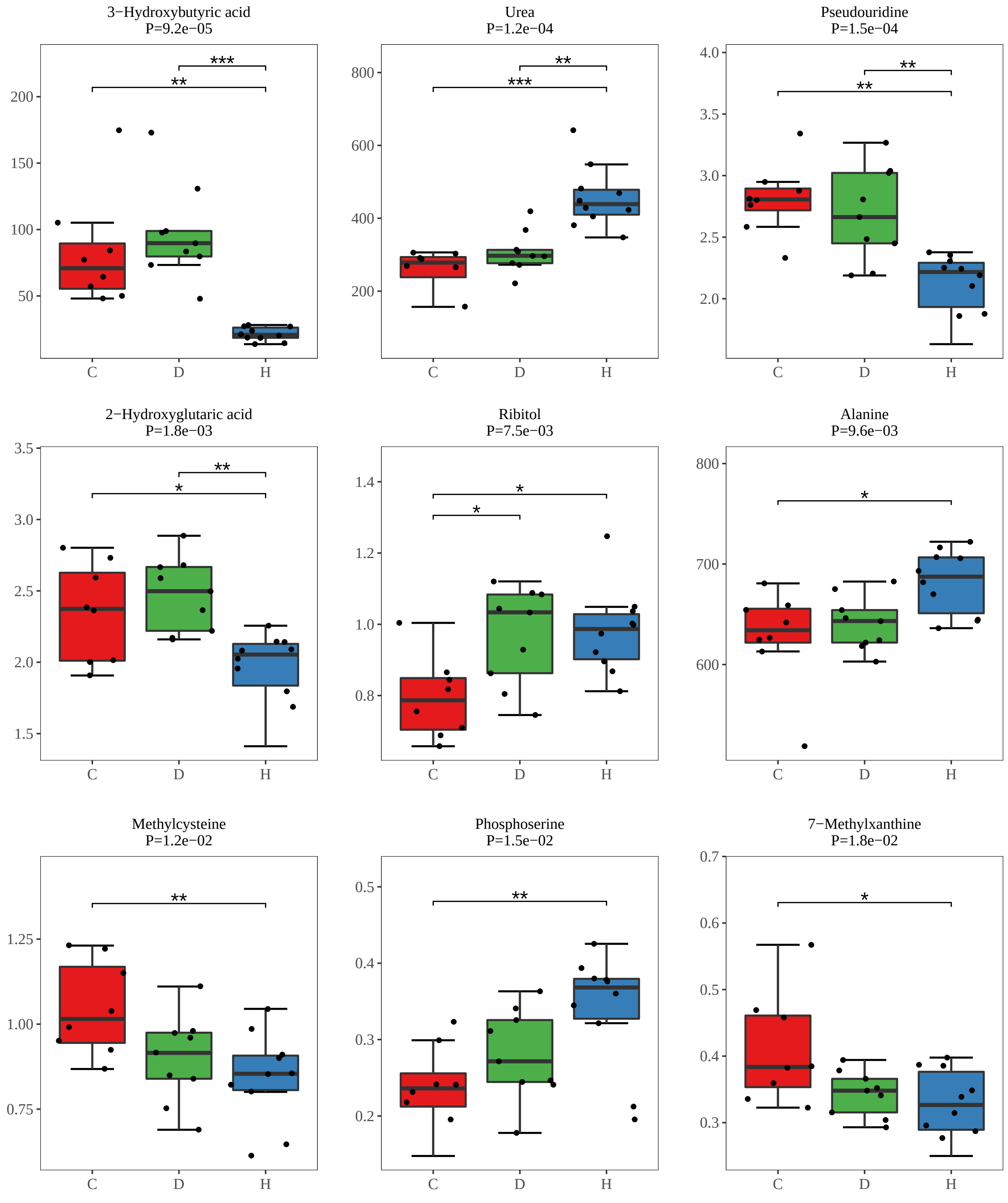
3−Hydroxybutyric acid
P=9.2e−05
Urea
P=1.2e−04
Pseudouridine
P=1.5e−04
2−Hydroxyglutaric acid
P=1.8e−03
Ribitol
P=7.5e−03
Alanine
P=9.6e−03
Methylcysteine
P=1.2e−02
Phosphoserine
P=1.5e−02
7−Methylxanthine
P=1.8e−02
C
D
H

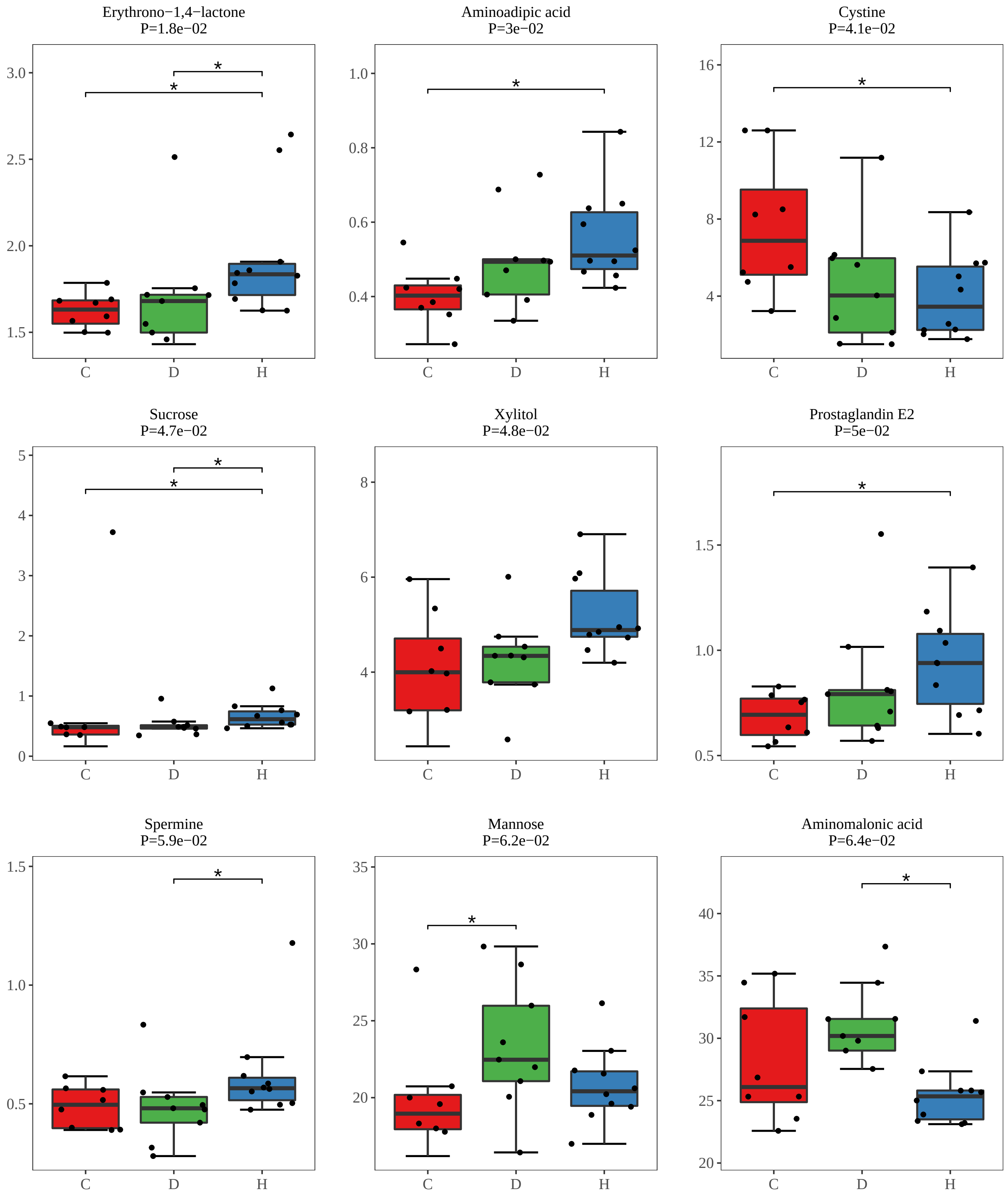

Erythrono−1,4−lactone
P=1.8e−02
Aminoadipic acid
P=3e−02
Cystine
P=4.1e−02
Sucrose
P=4.7e−02
Xylitol
P=4.8e−02
Prostaglandin E2
P=5e−02
Spermine
P=5.9e−02
Mannose
P=6.2e−02
Aminomalonic acid
P=6.4e−02
C
D
H

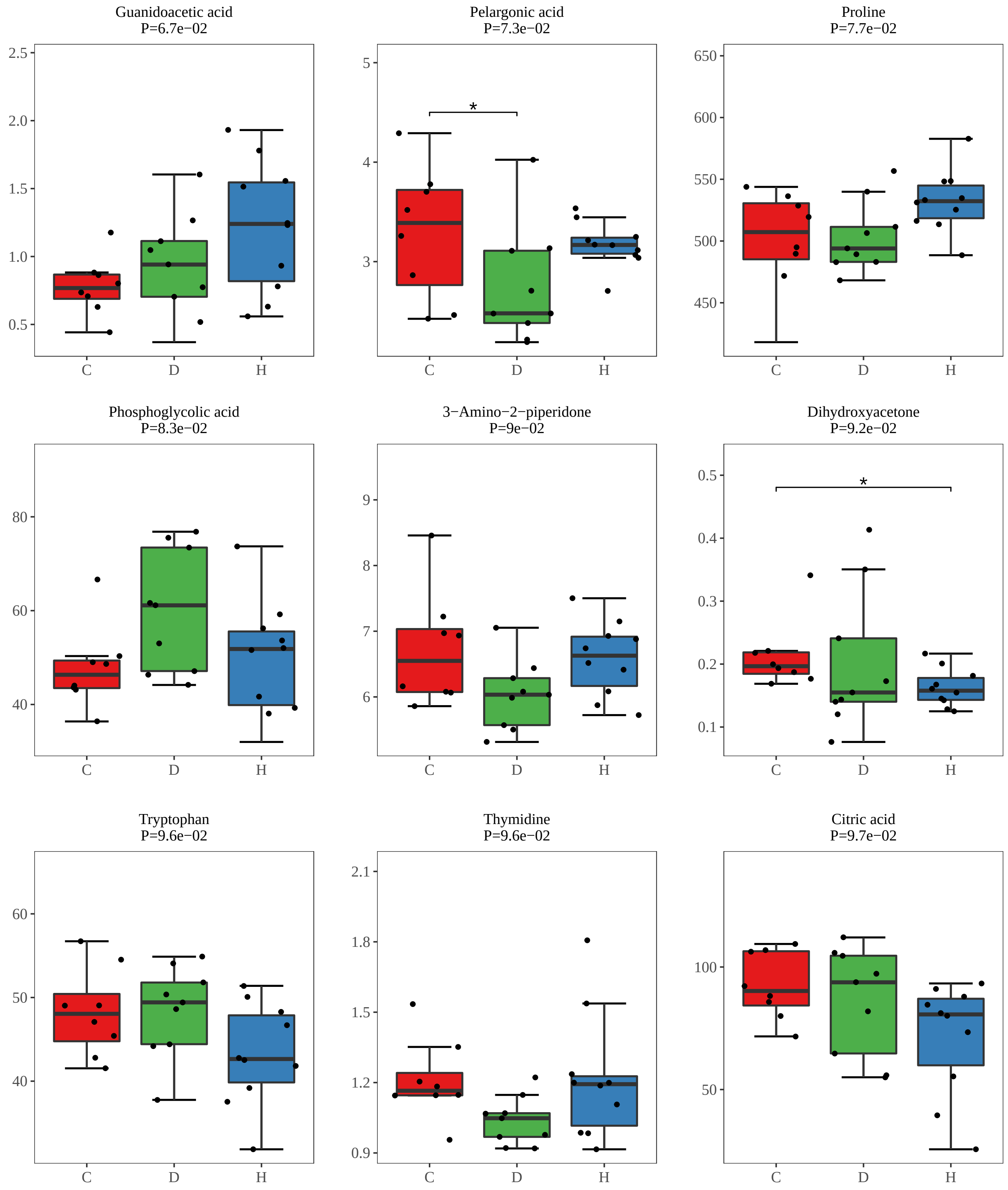

Guanidoacetic acid
P=6.7e−02
Pelargonic acid
P=7.3e−02
Proline
P=7.7e−02
Phosphoglycolic acid
P=8.3e−02
3−Amino−2−piperidone
P=9e−02
Dihydroxyacetone
P=9.2e−02
Tryptophan
P=9.6e−02
Thymidine
P=9.6e−02
Citric acid
P=9.7e−02
*
C
D
H

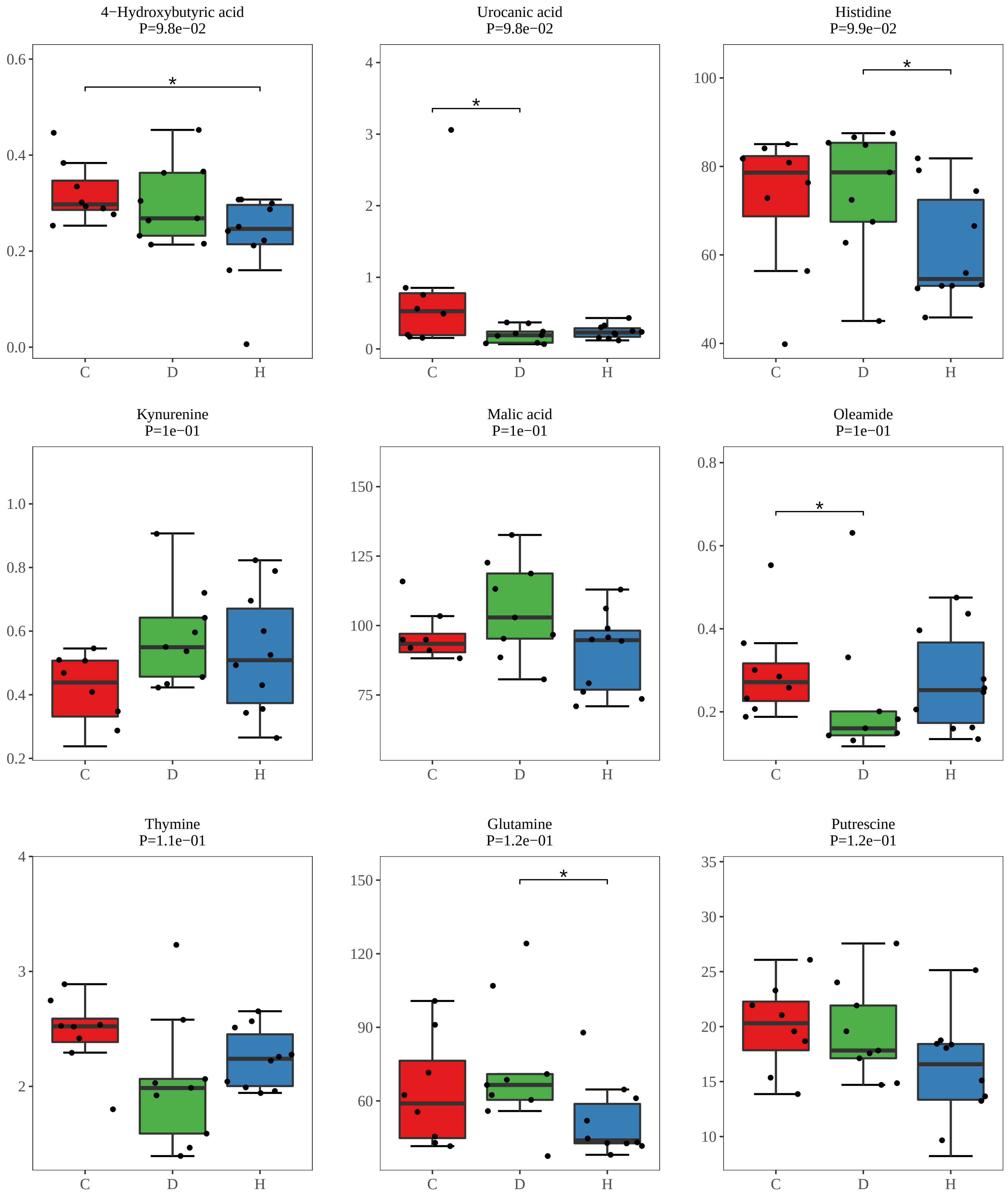
4−Hydroxybutyric acid
P=9.8e−02
Urocanic acid
P=9.8e−02
Histidine
P=9.9e−02
Kynurenine
P=1e−01
Malic acid
P=1e−01
Oleamide
P=1e−01
Thymine
P=1.1e−01
Glutamine
P=1.2e−01
Putrescine
P=1.2e−01
C
D
H
*

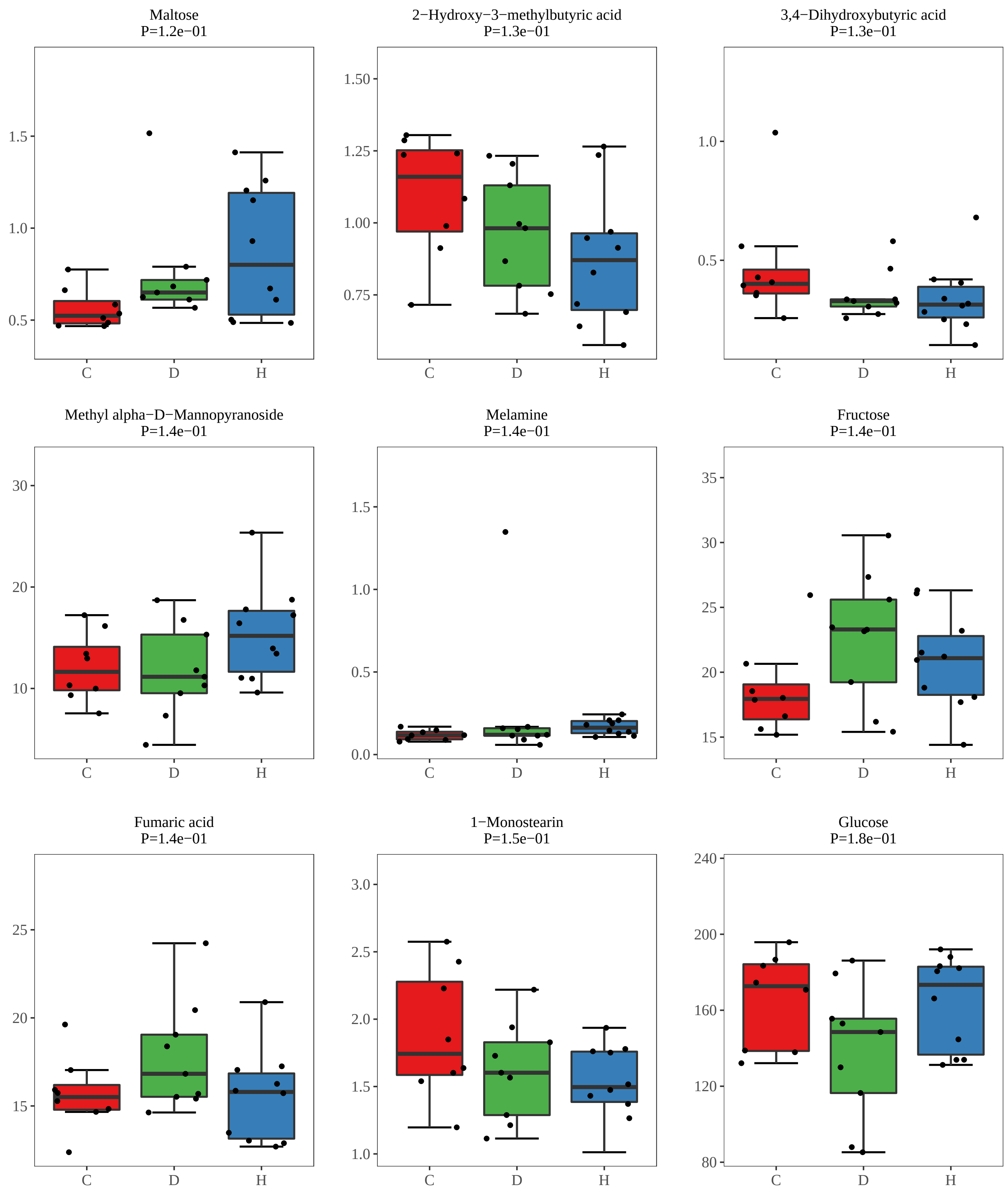

Maltose
P=1.2e−01
0.5
1.0
1.5
C
D
H
2−Hydroxy−3−methylbutyric acid
P=1.3e−01
0.75
1.00
1.25
1.50
C
D
H
3,4−Dihydroxybutyric acid
P=1.3e−01
0.5
1.0
C
D
H
Methyl alpha−D−Mannopyranoside
P=1.4e−01
10
20
30
C
D
H
Melamine
P=1.4e−01
0.0
0.5
1.0
1.5
C
D
H
Fructose
P=1.4e−01
15
20
25
30
35
C
D
H
Fumaric acid
P=1.4e−01
15
20
25
C
D
H
1−Monostearin
P=1.5e−01
1.0
1.5
2.0
2.5
3.0
C
D
H
Glucose
P=1.8e−01
80
120
160
200
240
C
D
H

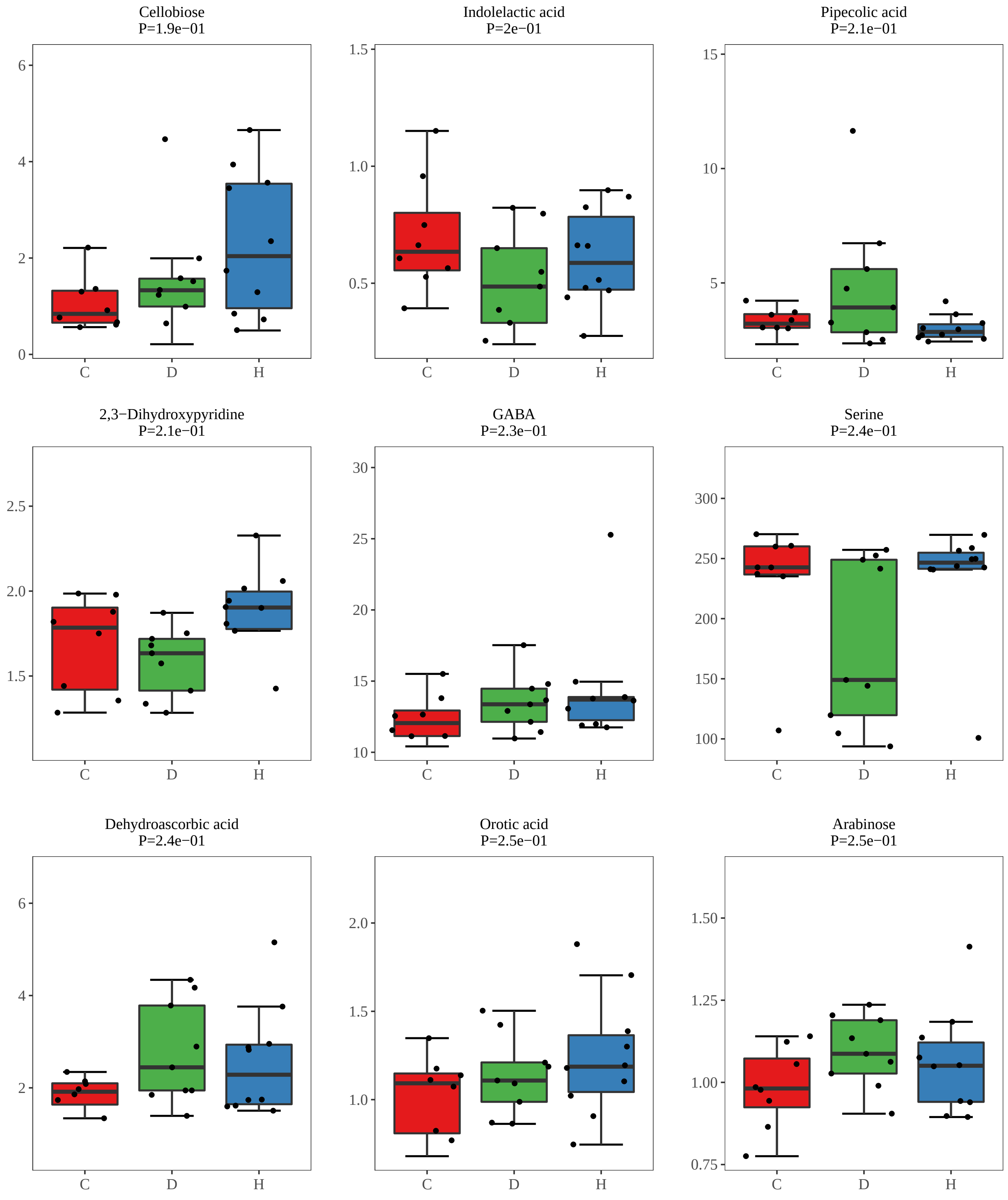

Cellobiose
P=1.9e−01
Indolelactic acid
P=2e−01
Pipecolic acid
P=2.1e−01
2,3−Dihydroxypyridine
P=2.1e−01
GABA
P=2.3e−01
Serine
P=2.4e−01
Dehydroascorbic acid
P=2.4e−01
Orotic acid
P=2.5e−01
Arabinose
P=2.5e−01
C
D
H

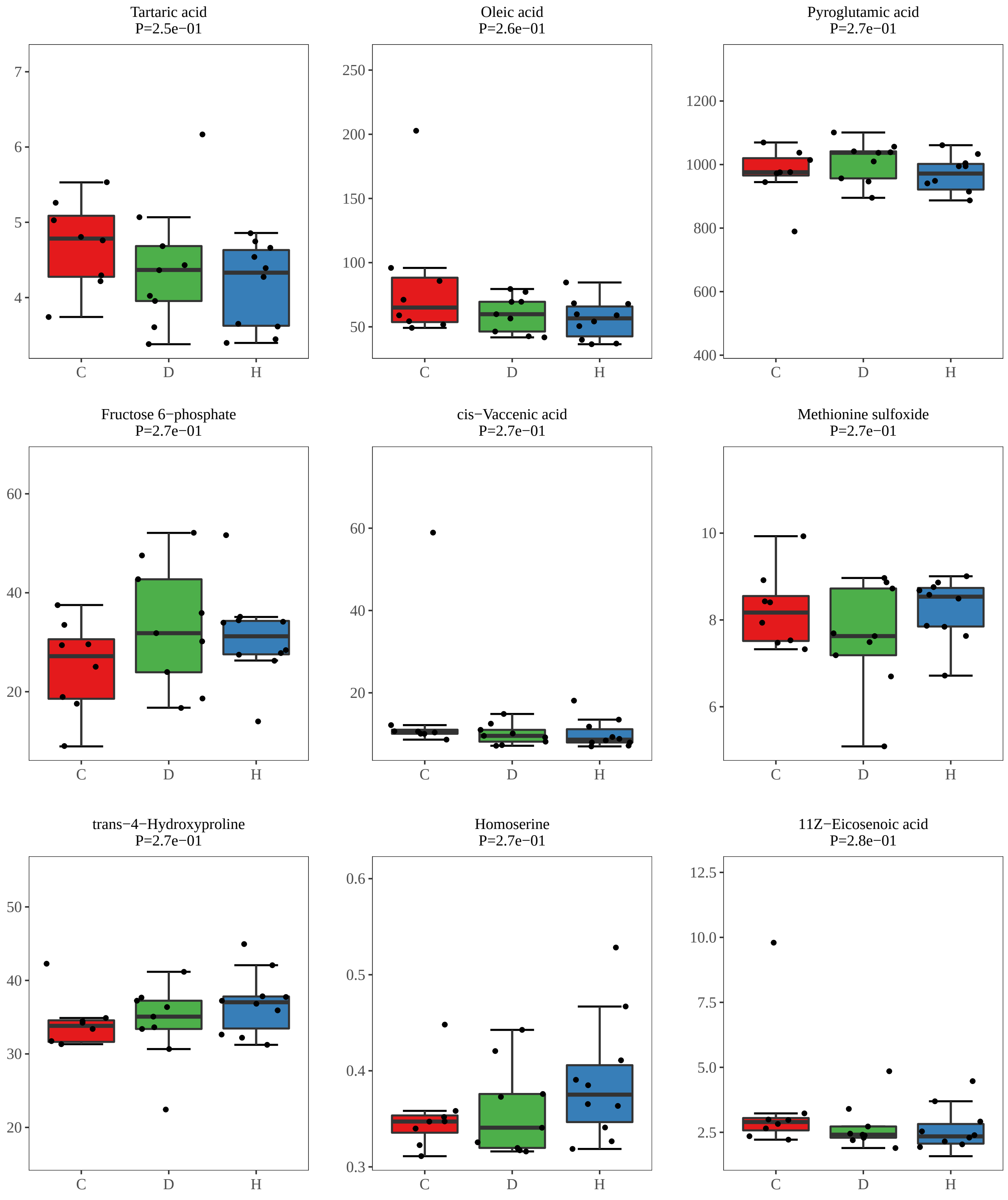
Tartaric acid
P=2.5e−01
Oleic acid
P=2.6e−01
Pyroglutamic acid
P=2.7e−01
Fructose 6−phosphate
P=2.7e−01
cis−Vaccenic acid
P=2.7e−01
Methionine sulfoxide
P=2.7e−01
trans−4−Hydroxyproline
P=2.7e−01
Homoserine
P=2.7e−01
11Z−Eicosenoic acid
P=2.8e−01
C
D
H

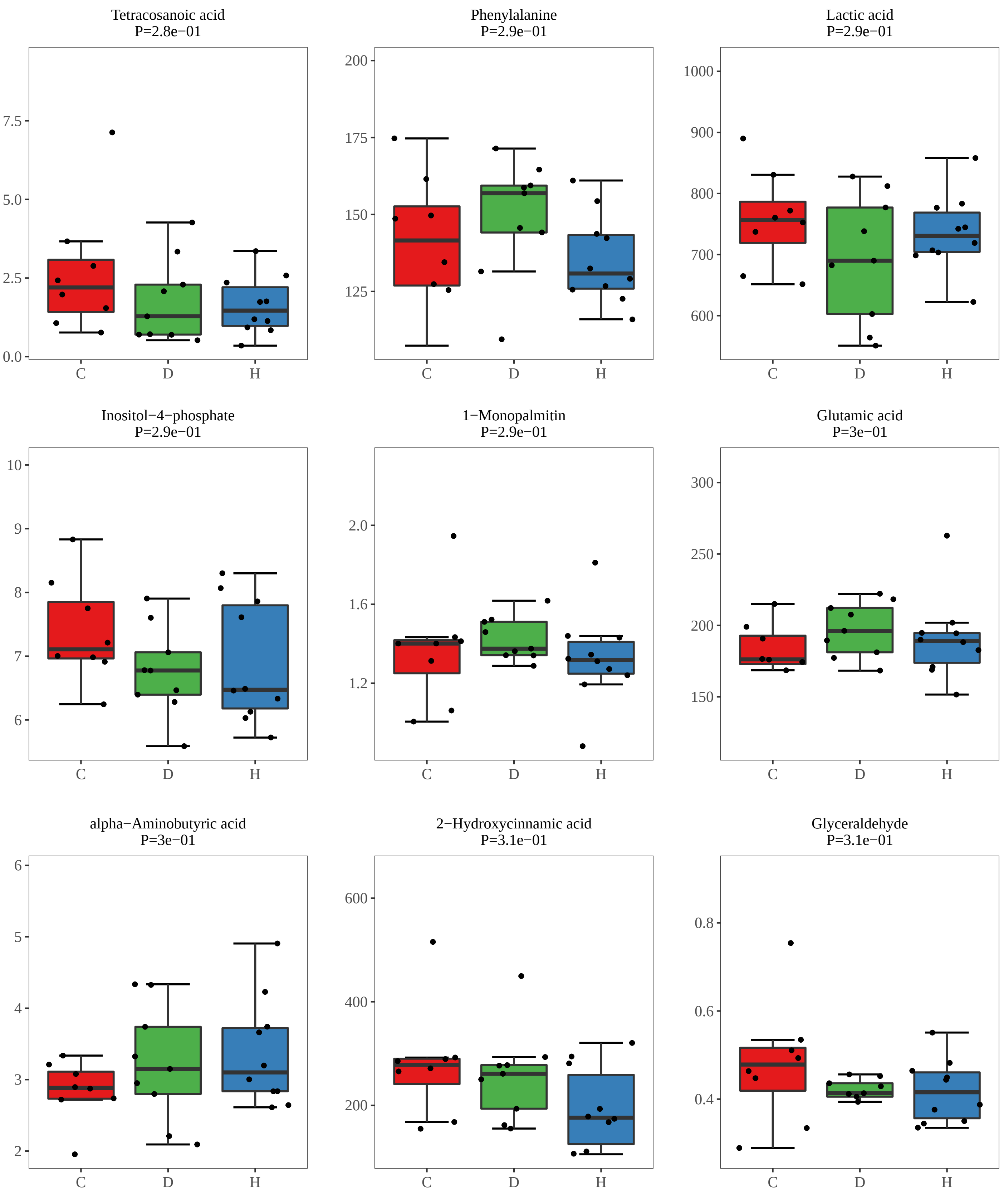
Tetracosanoic acid
P=2.8e−01
Phenylalanine
P=2.9e−01
Lactic acid
P=2.9e−01
Inositol−4−phosphate
P=2.9e−01
1−Monopalmitin
P=2.9e−01
Glutamic acid
P=3e−01
alpha−Aminobutyric acid
P=3e−01
2−Hydroxycinnamic acid
P=3.1e−01
Glyceraldehyde
P=3.1e−01
C
D
H

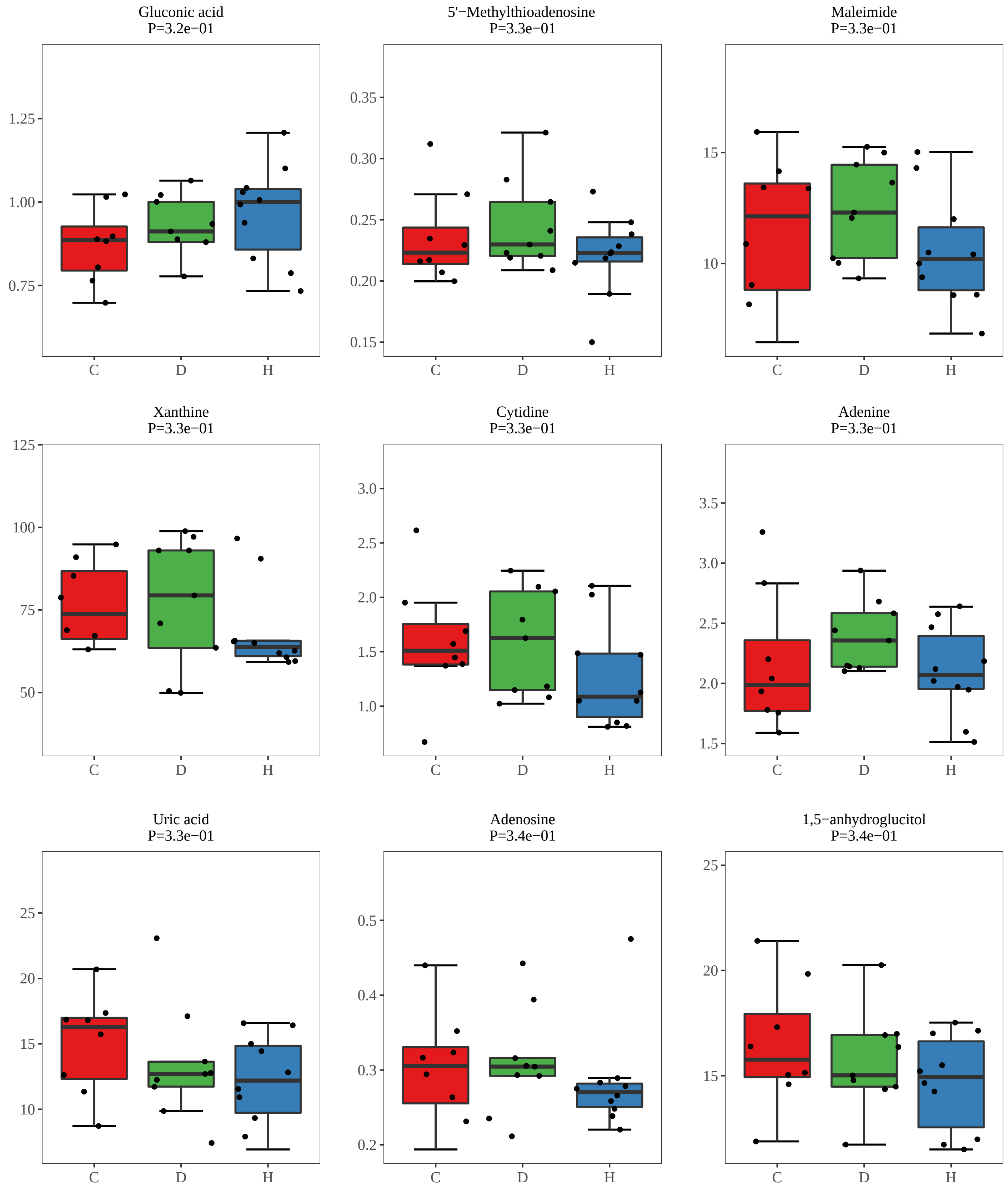
Gluconic acid
P=3.2e−01
1.25
1.00
0.75
C
D
H
5'−Methylthioadenosine
P=3.3e−01
0.35
0.30
0.25
0.20
0.15
C
D
H
Maleimide
P=3.3e−01
15
10
C
D
H
Xanthine
P=3.3e−01
125
100
75
50
C
D
H
Cytidine
P=3.3e−01
3.0
2.5
2.0
1.5
1.0
C
D
H
Adenine
P=3.3e−01
3.5
3.0
2.5
2.0
1.5
C
D
H
Uric acid
P=3.3e−01
25
20
15
10
C
D
H
Adenosine
P=3.4e−01
0.5
0.4
0.3
0.2
C
D
H
1,5−anhydroglucitol
P=3.4e−01
25
20
15
C
D
H

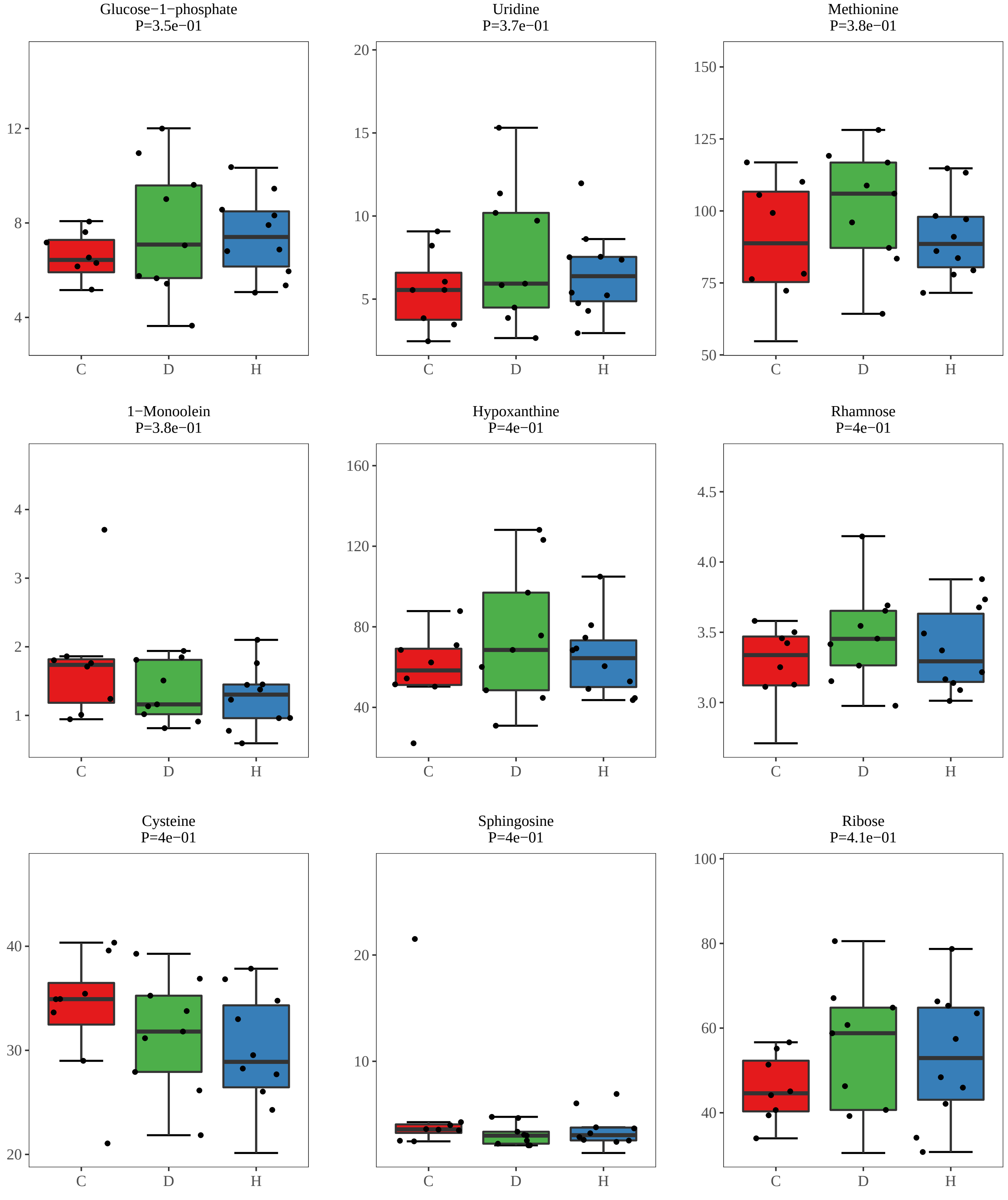

Glucose−1−phosphate
P=3.5e−01
4
8
12
Uridine
P=3.7e−01
5
10
15
20
Methionine
P=3.8e−01
50
75
100
125
150
1−Monoolein
P=3.8e−01
1
2
3
4
Hypoxanthine
P=4e−01
40
80
120
160
Rhamnose
P=4e−01
3.0
3.5
4.0
4.5
Cysteine
P=4e−01
20
30
40
Sphingosine
P=4e−01
10
20
Ribose
P=4.1e−01
40
60
80
100
C
D
H

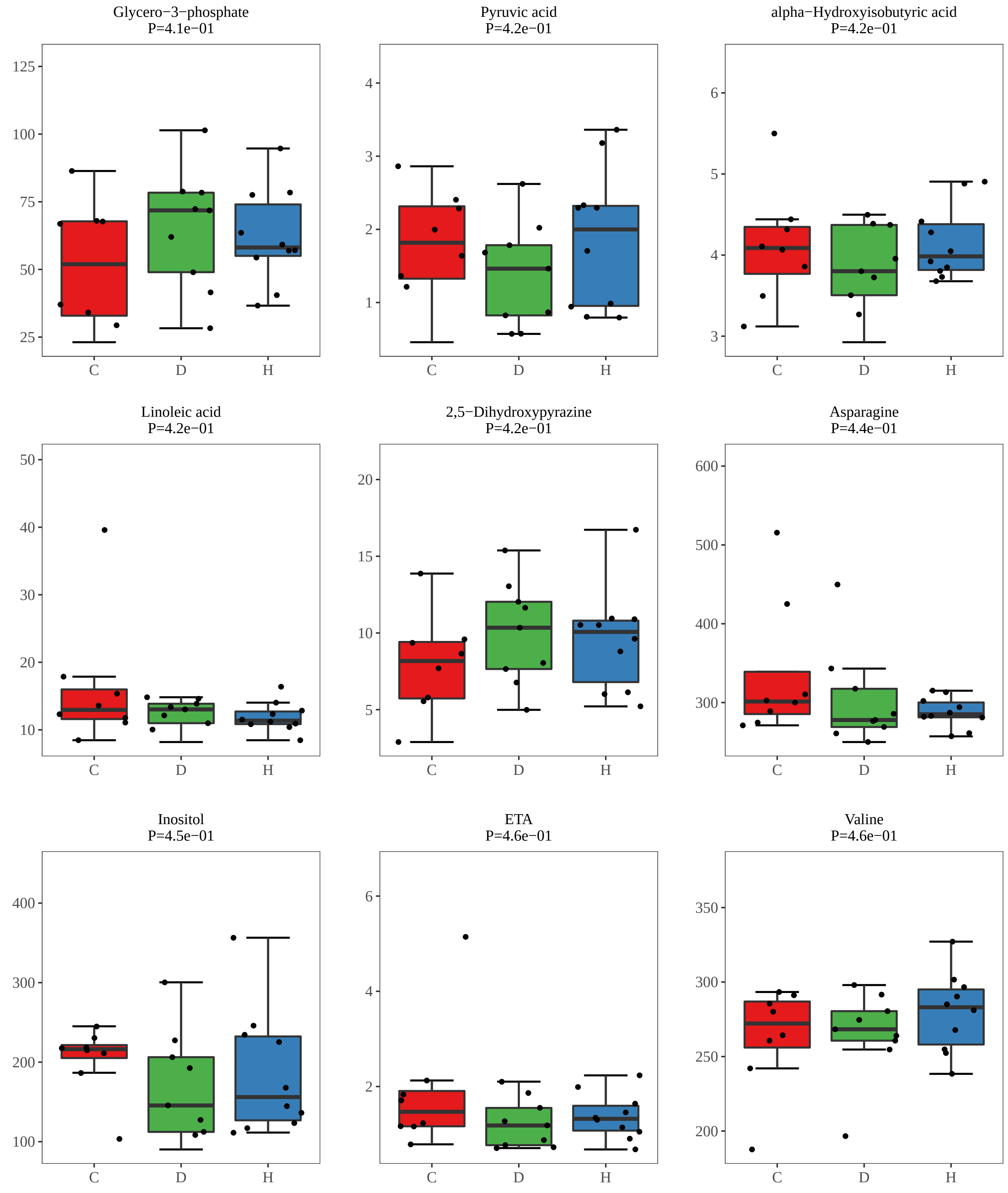
Glycero−3−phosphate
P=4.1e−01
Pyruvic acid
P=4.2e−01
alpha−Hydroxyisobutyric acid
P=4.2e−01
Linoleic acid
P=4.2e−01
2,5−Dihydroxypyrazine
P=4.2e−01
Asparagine
P=4.4e−01
Inositol
P=4.5e−01
ETA
P=4.6e−01
Valine
P=4.6e−01
C
D
H

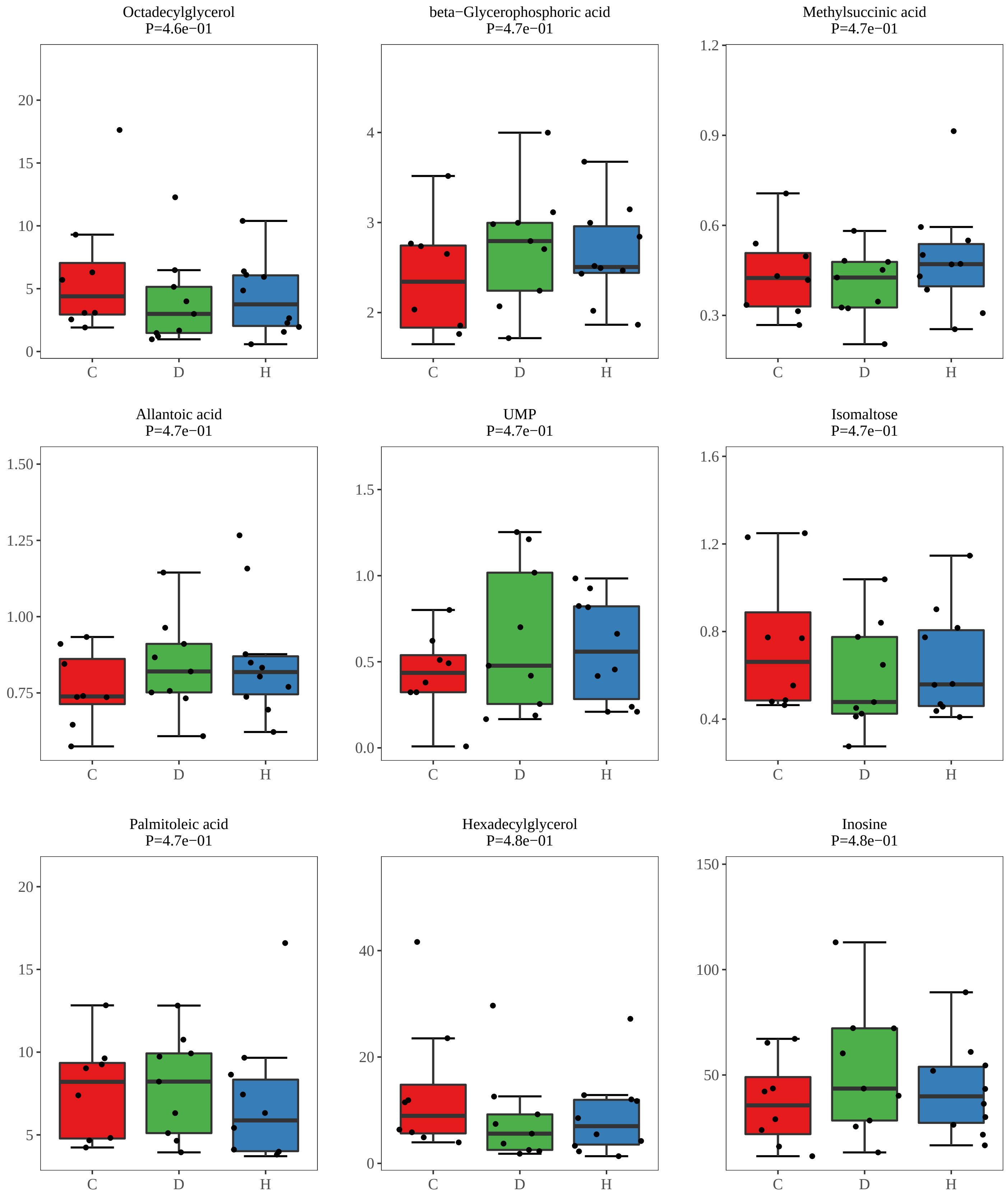
Octadecylglycerol
P=4.6e−01
0
5
10
15
20
C
D
H
beta−Glycerophosphoric acid
P=4.7e−01
2
3
4
C
D
H
Methylsuccinic acid
P=4.7e−01
0.3
0.6
0.9
1.2
C
D
H
Allantoic acid
P=4.7e−01
0.75
1.00
1.25
1.50
C
D
H
UMP
P=4.7e−01
0.0
0.5
1.0
1.5
C
D
H
Isomaltose
P=4.7e−01
0.4
0.8
1.2
1.6
C
D
H
Palmitoleic acid
P=4.7e−01
5
10
15
20
C
D
H
Hexadecylglycerol
P=4.8e−01
0
20
40
C
D
H
Inosine
P=4.8e−01
50
100
150
C
D
H

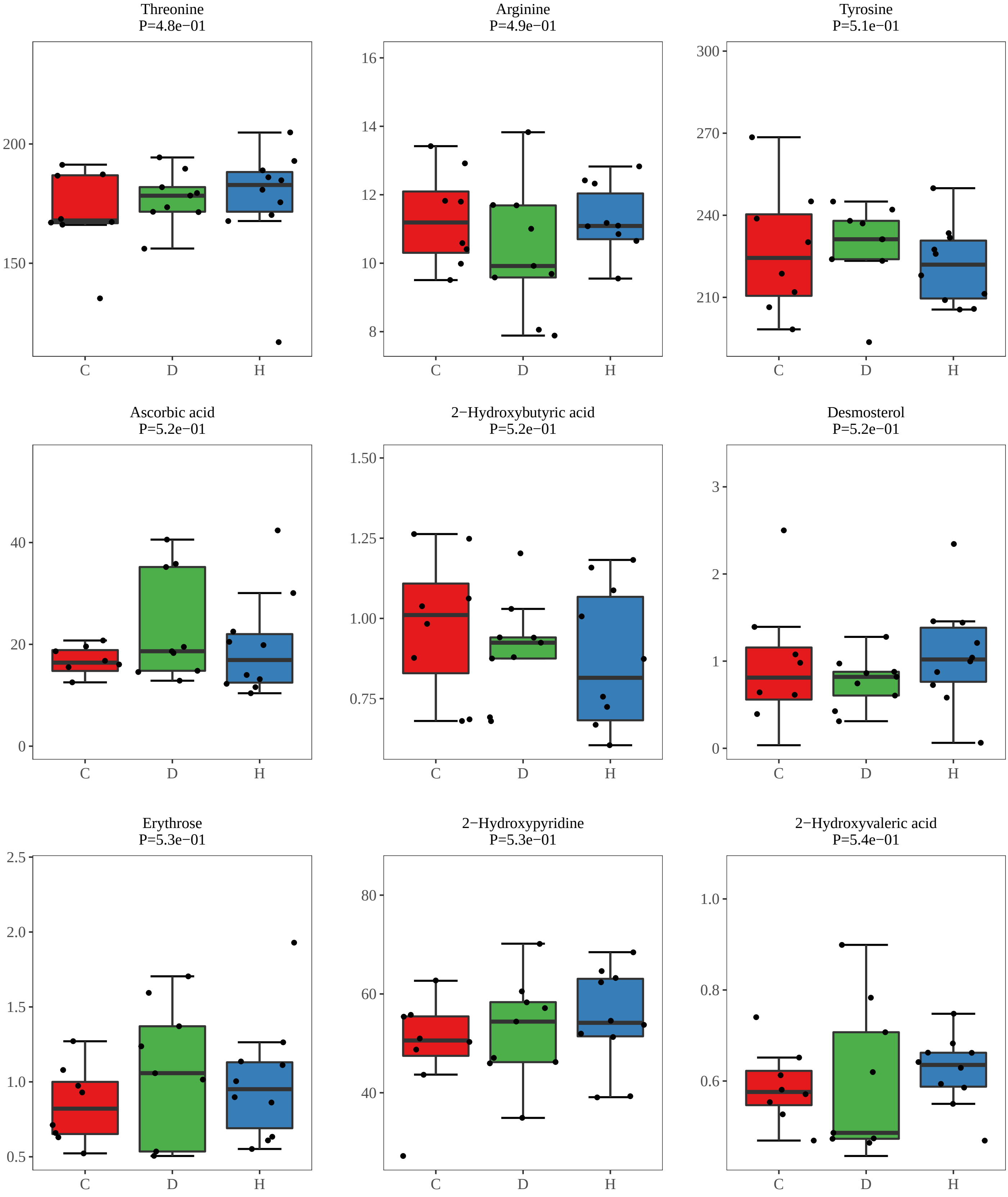

Threonine
P=4.8e−01
200
150
C
D
H
Arginine
P=4.9e−01
16
14
12
10
8
C
D
H
Tyrosine
P=5.1e−01
300
270
240
210
C
D
H
Ascorbic acid
P=5.2e−01
40
20
0
C
D
H
2−Hydroxybutyric acid
P=5.2e−01
1.50
1.25
1.00
0.75
C
D
H
Desmosterol
P=5.2e−01
3
2
1
0
C
D
H
Erythrose
P=5.3e−01
2.5
2.0
1.5
1.0
0.5
C
D
H
2−Hydroxypyridine
P=5.3e−01
80
60
40
C
D
H
2−Hydroxyvaleric acid
P=5.4e−01
1.0
0.8
0.6
C
D
H

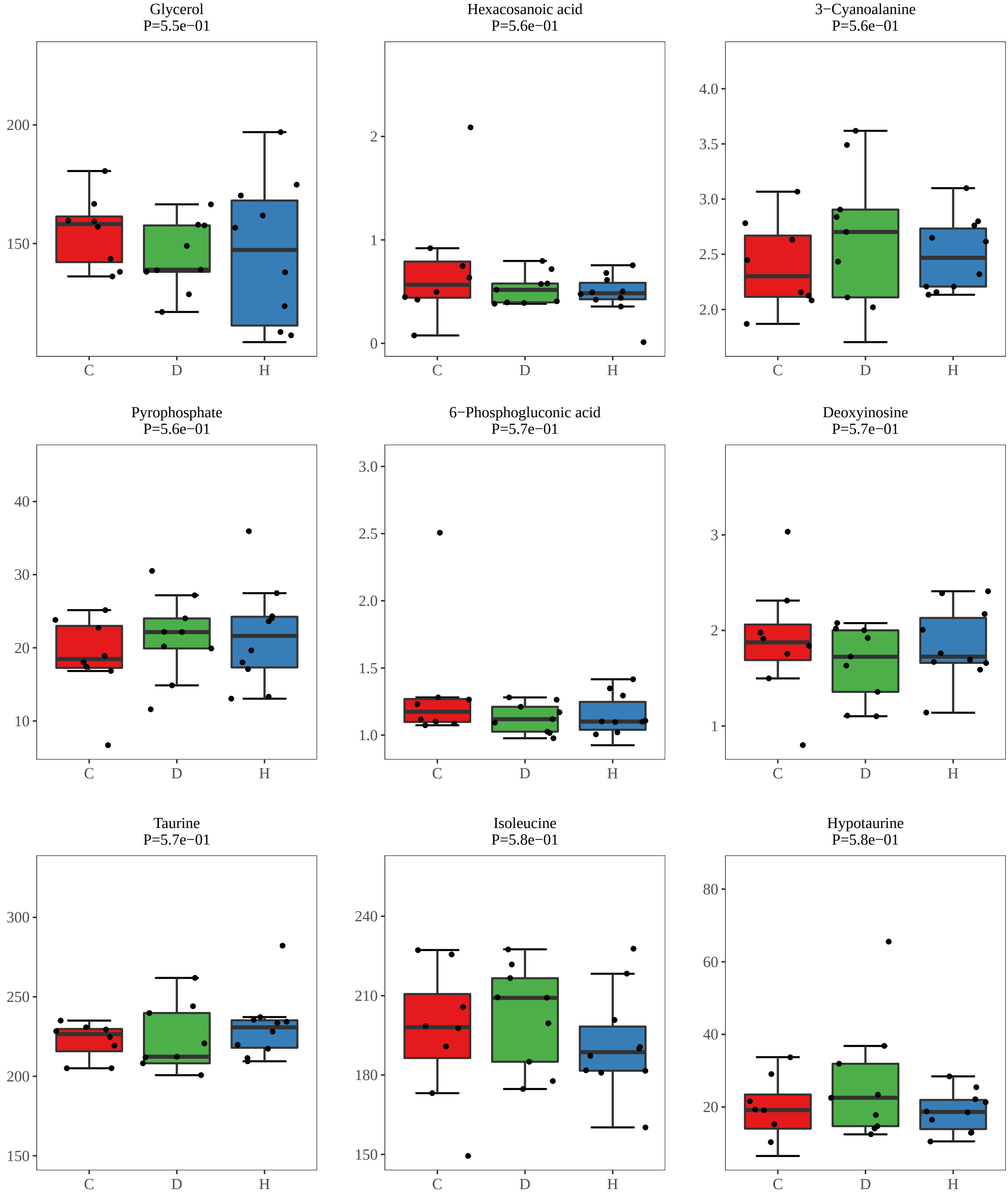
Glycerol
P=5.5e−01
Hexacosanoic acid
P=5.6e−01
3−Cyanoalanine
P=5.6e−01
Pyrophosphate
P=5.6e−01
6−Phosphogluconic acid
P=5.7e−01
Deoxyinosine
P=5.7e−01
Taurine
P=5.7e−01
Isoleucine
P=5.8e−01
Hypotaurine
P=5.8e−01
C
D
H

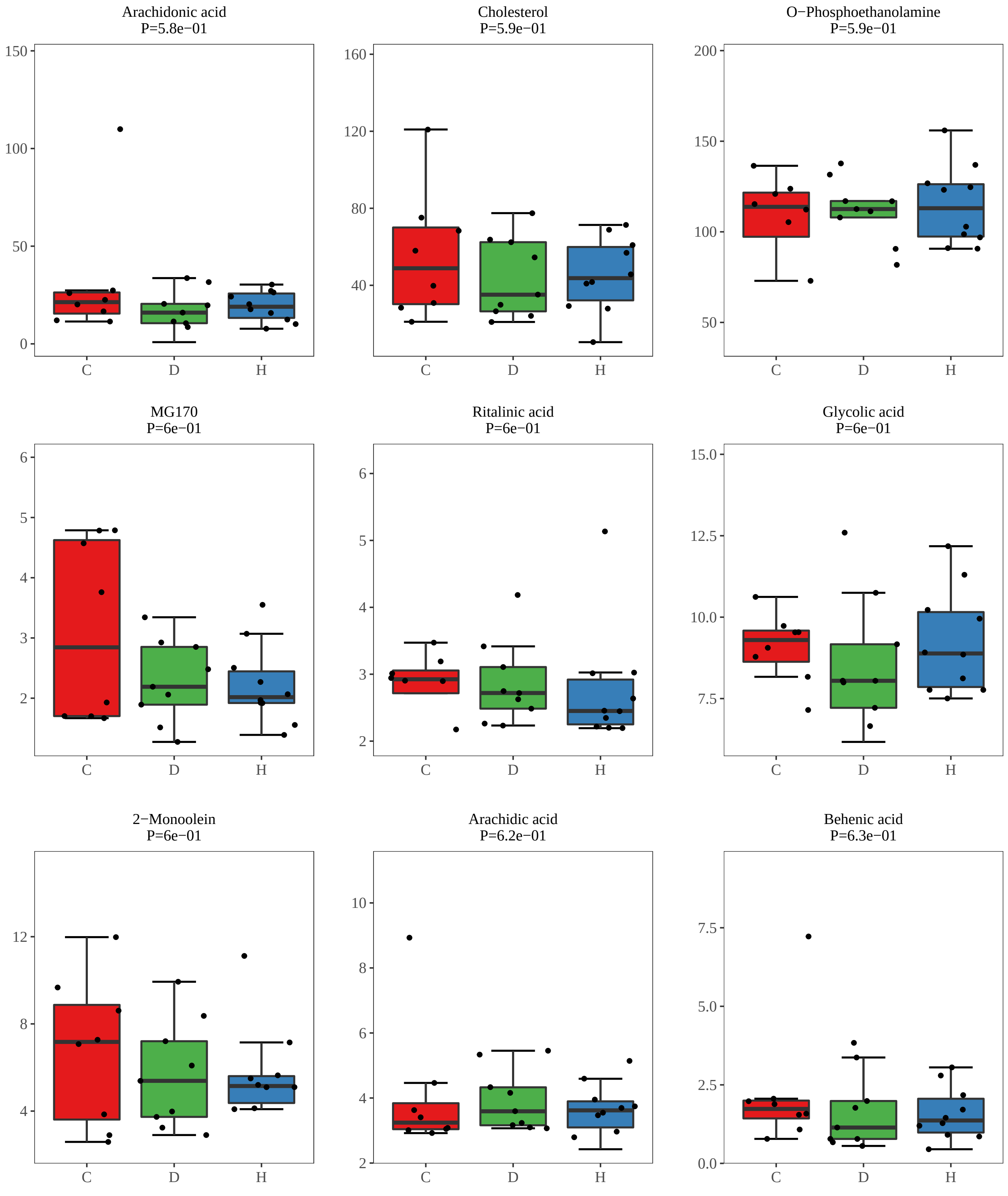
Arachidonic acid
P=5.8e−01
150
100
50
0
C
D
H
Cholesterol
P=5.9e−01
160
120
80
40
C
D
H
O−Phosphoethanolamine
P=5.9e−01
200
150
100
50
C
D
H
MG170
P=6e−01
6
5
4
3
2
C
D
H
Ritalinic acid
P=6e−01
6
5
4
3
2
C
D
H
Glycolic acid
P=6e−01
15.0
12.5
10.0
7.5
C
D
H
2−Monoolein
P=6e−01
12
8
4
C
D
H
Arachidic acid
P=6.2e−01
10
8
6
4
2
C
D
H
Behenic acid
P=6.3e−01
7.5
5.0
2.5
0.0
C
D
H

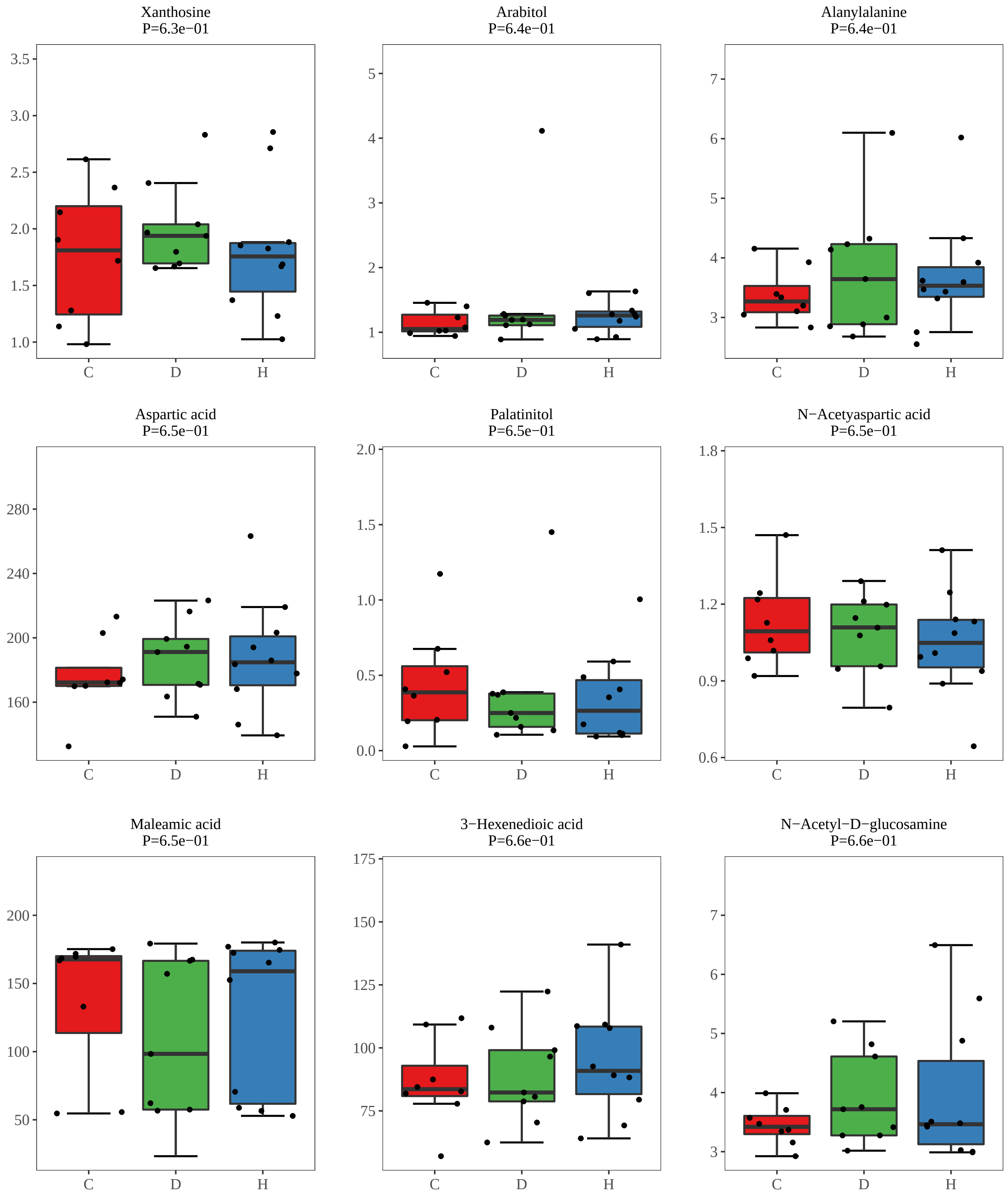
Xanthosine
P=6.3e−01
Arabitol
P=6.4e−01
Alanylalanine
P=6.4e−01
Aspartic acid
P=6.5e−01
Palatinitol
P=6.5e−01
N−Acetyaspartic acid
P=6.5e−01
Maleamic acid
P=6.5e−01
3−Hexenedioic acid
P=6.6e−01
N−Acetyl−D−glucosamine
P=6.6e−01
C
D
H

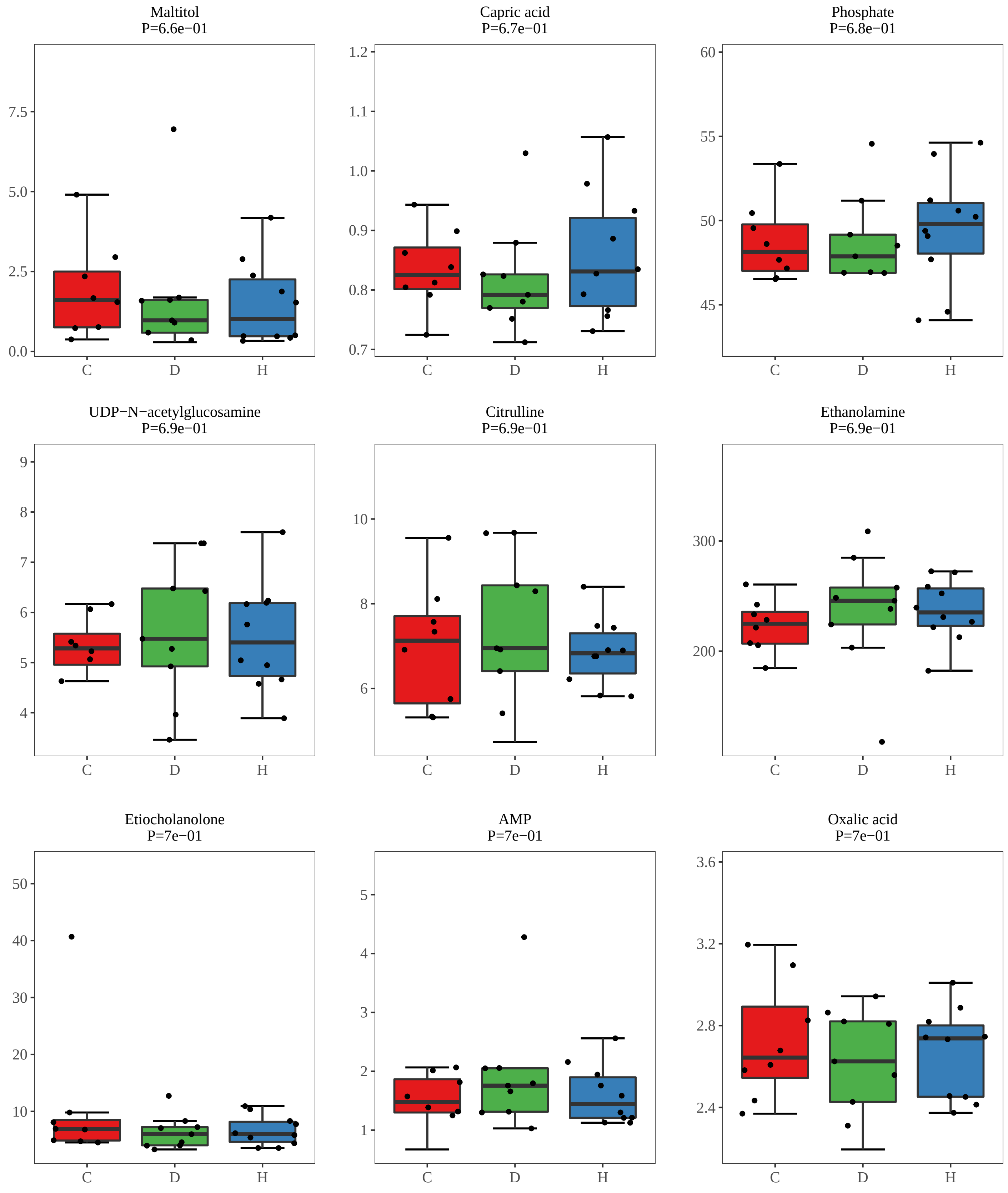
Maltitol
P=6.6e−01
0.0
2.5
5.0
7.5
Capric acid
P=6.7e−01
0.7
0.8
0.9
1.0
1.1
1.2
Phosphate
P=6.8e−01
45
50
55
60
UDP−N−acetylglucosamine
P=6.9e−01
4
5
6
7
8
9
Citrulline
P=6.9e−01
6
8
10
Ethanolamine
P=6.9e−01
200
300
Etiocholanolone
P=7e−01
10
20
30
40
50
AMP
P=7e−01
1
2
3
4
5
Oxalic acid
P=7e−01
2.4
2.8
3.2
3.6
C
D
H

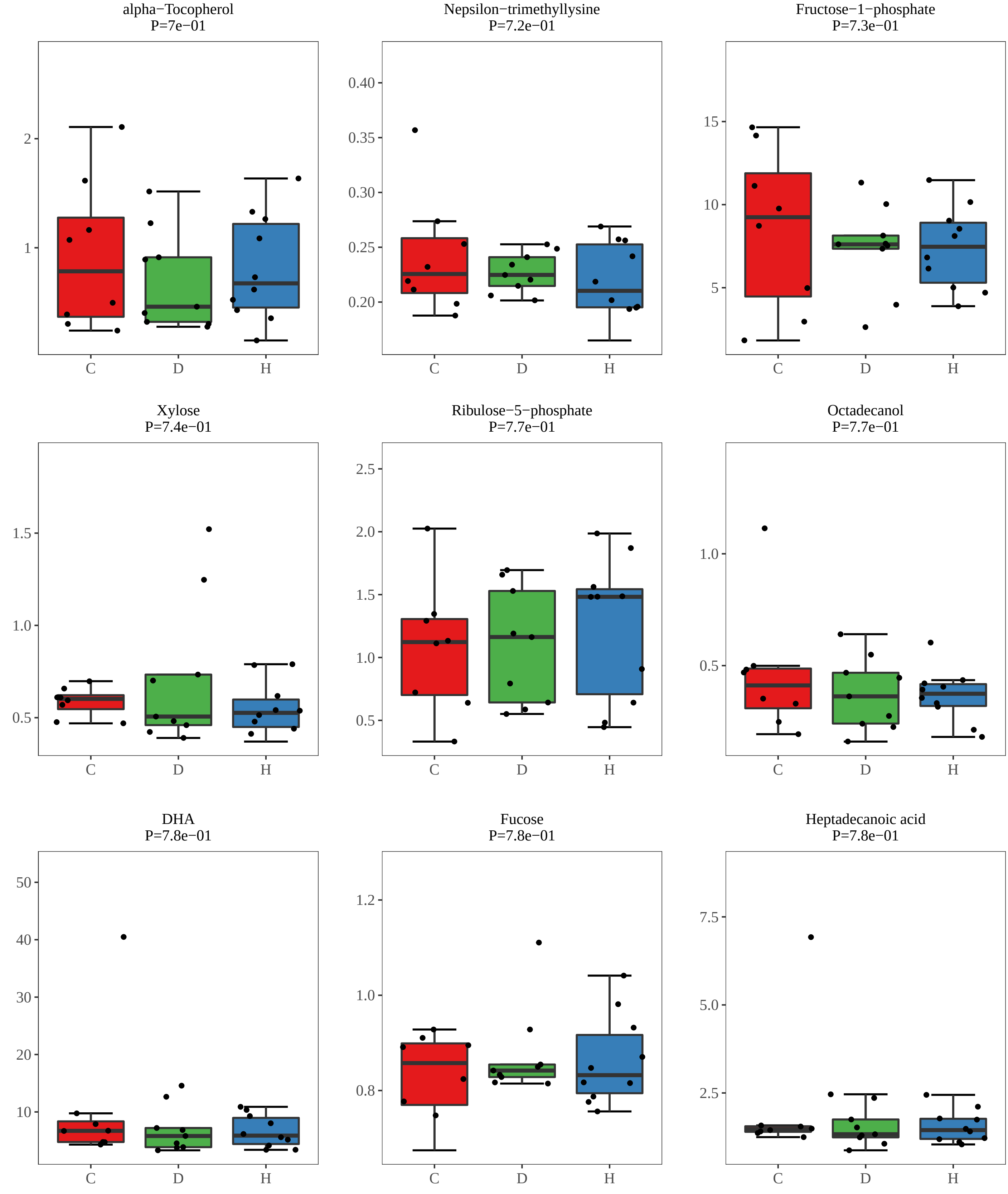
alpha−Tocopherol
P=7e−01
1
2
C
D
H
Nepsilon−trimethyllysine
P=7.2e−01
0.20
0.25
0.30
0.35
0.40
C
D
H
Fructose−1−phosphate
P=7.3e−01
5
10
15
C
D
H
Xylose
P=7.4e−01
0.5
1.0
1.5
C
D
H
Ribulose−5−phosphate
P=7.7e−01
0.5
1.0
1.5
2.0
2.5
C
D
H
Octadecanol
P=7.7e−01
0.5
1.0
C
D
H
DHA
P=7.8e−01
10
20
30
40
50
C
D
H
Fucose
P=7.8e−01
0.8
1.0
1.2
C
D
H
Heptadecanoic acid
P=7.8e−01
2.5
5.0
7.5
C
D
H

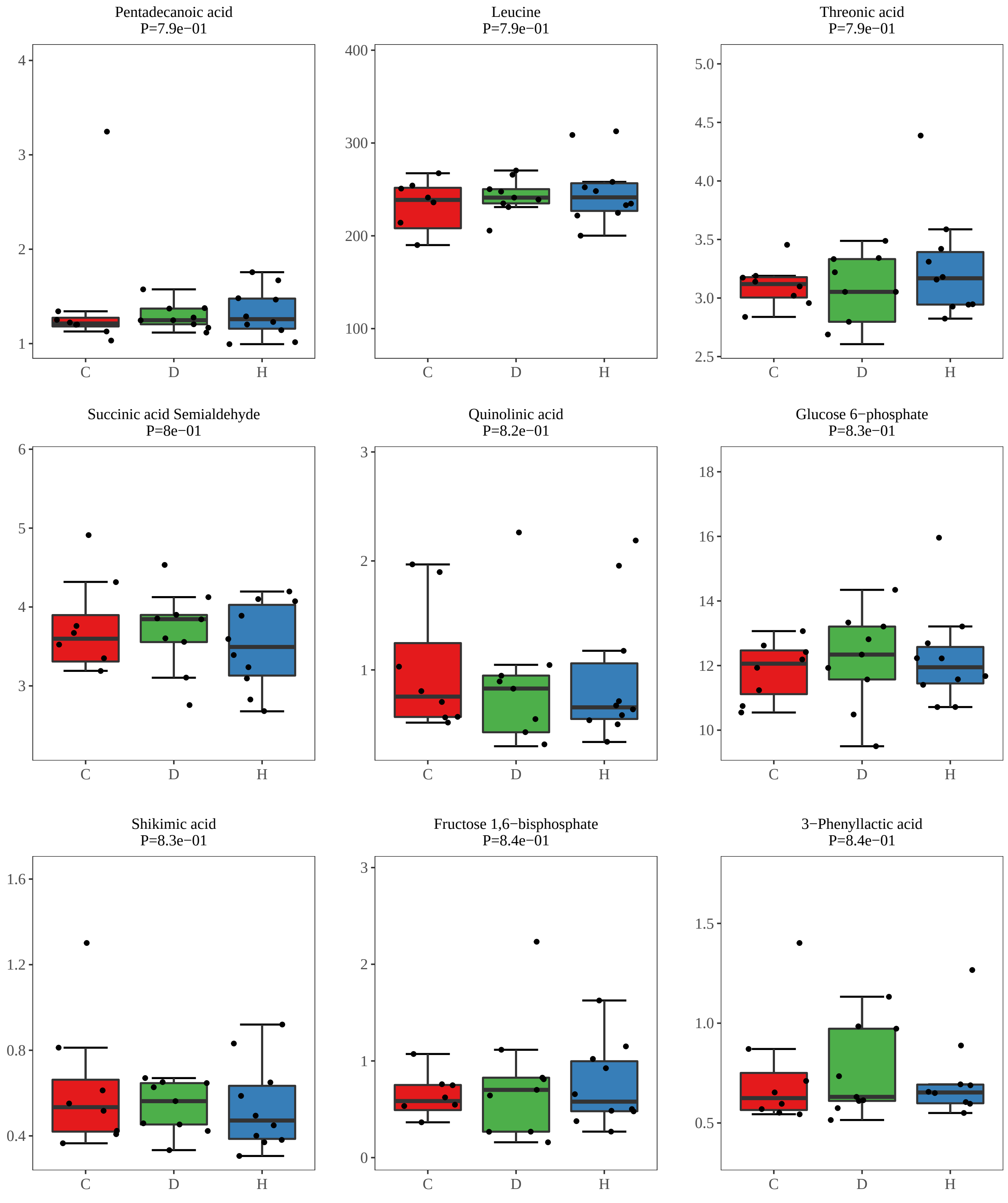
Pentadecanoic acid
P=7.9e−01
Leucine
P=7.9e−01
Threonic acid
P=7.9e−01
Succinic acid Semialdehyde
P=8e−01
Quinolinic acid
P=8.2e−01
Glucose 6−phosphate
P=8.3e−01
Shikimic acid
P=8.3e−01
Fructose 1,6−bisphosphate
P=8.4e−01
3−Phenyllactic acid
P=8.4e−01
C
D
H

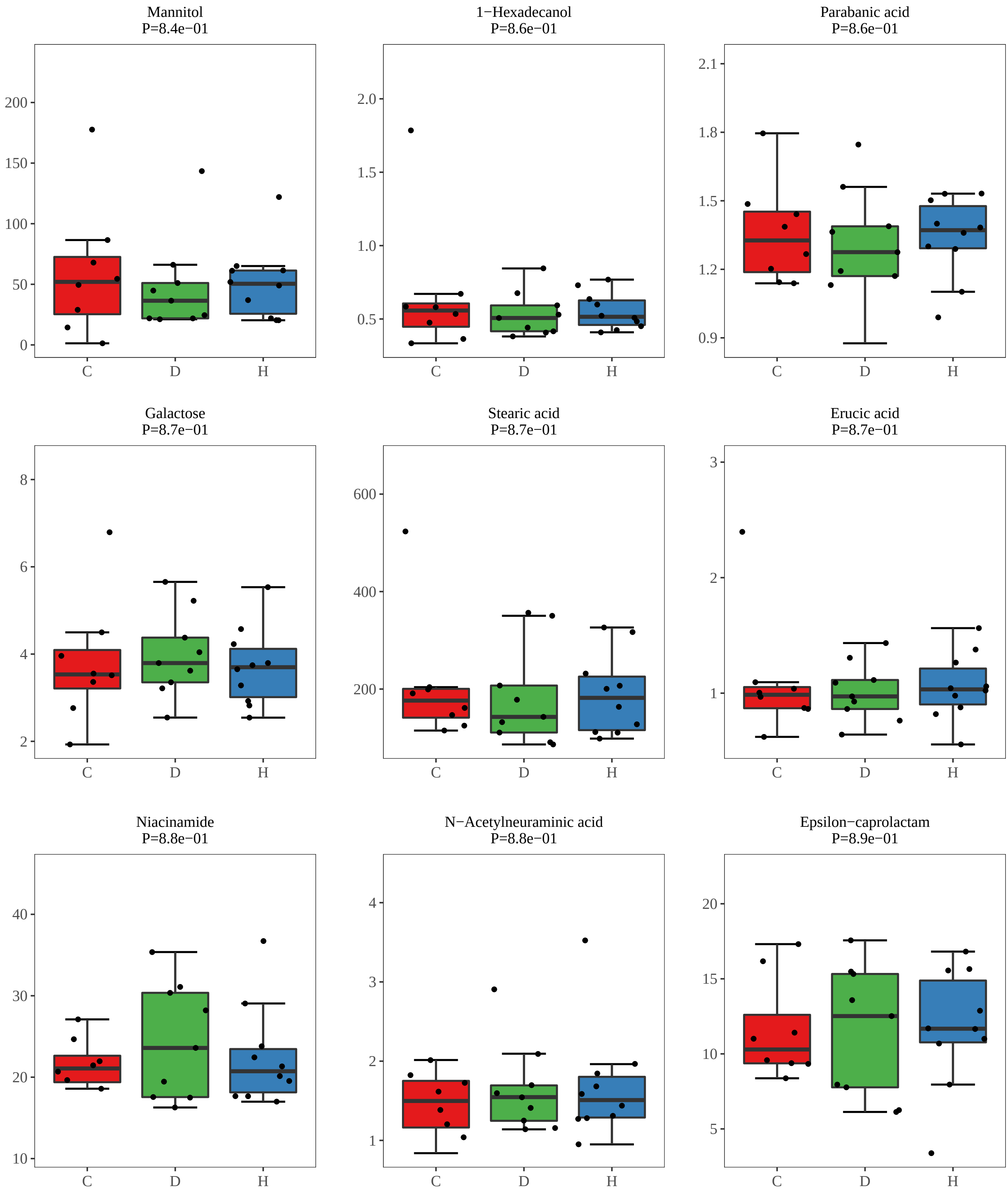

Mannitol
P=8.4e−01
1−Hexadecanol
P=8.6e−01
Parabanic acid
P=8.6e−01
Galactose
P=8.7e−01
Stearic acid
P=8.7e−01
Erucic acid
P=8.7e−01
Niacinamide
P=8.8e−01
N−Acetylneuraminic acid
P=8.8e−01
Epsilon−caprolactam
P=8.9e−01
C
D
H

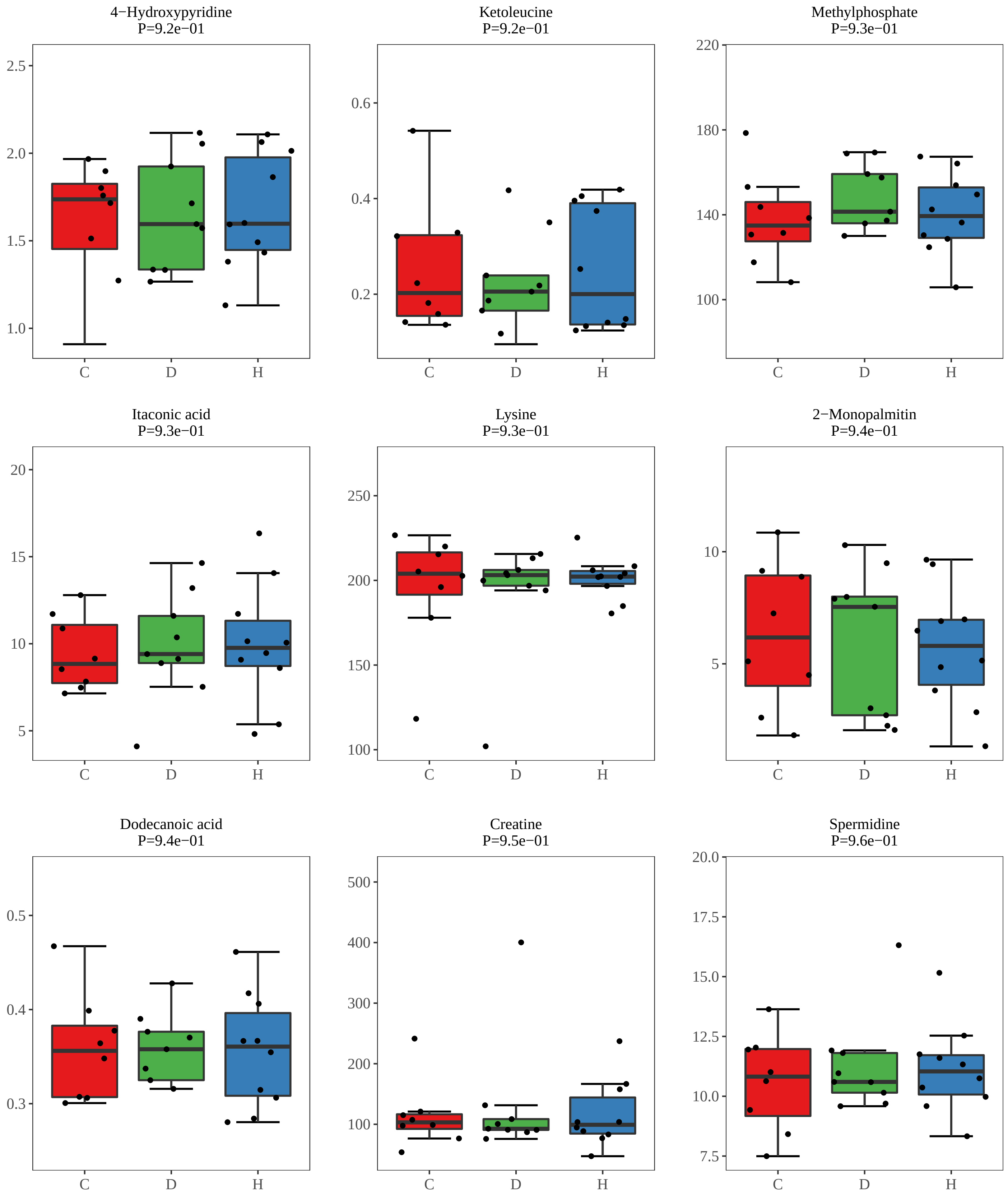

4−Hydroxypyridine
P=9.2e−01
Ketoleucine
P=9.2e−01
Methylphosphate
P=9.3e−01
Itaconic acid
P=9.3e−01
Lysine
P=9.3e−01
2−Monopalmitin
P=9.4e−01
Dodecanoic acid
P=9.4e−01
Creatine
P=9.5e−01
Spermidine
P=9.6e−01
C
D
H

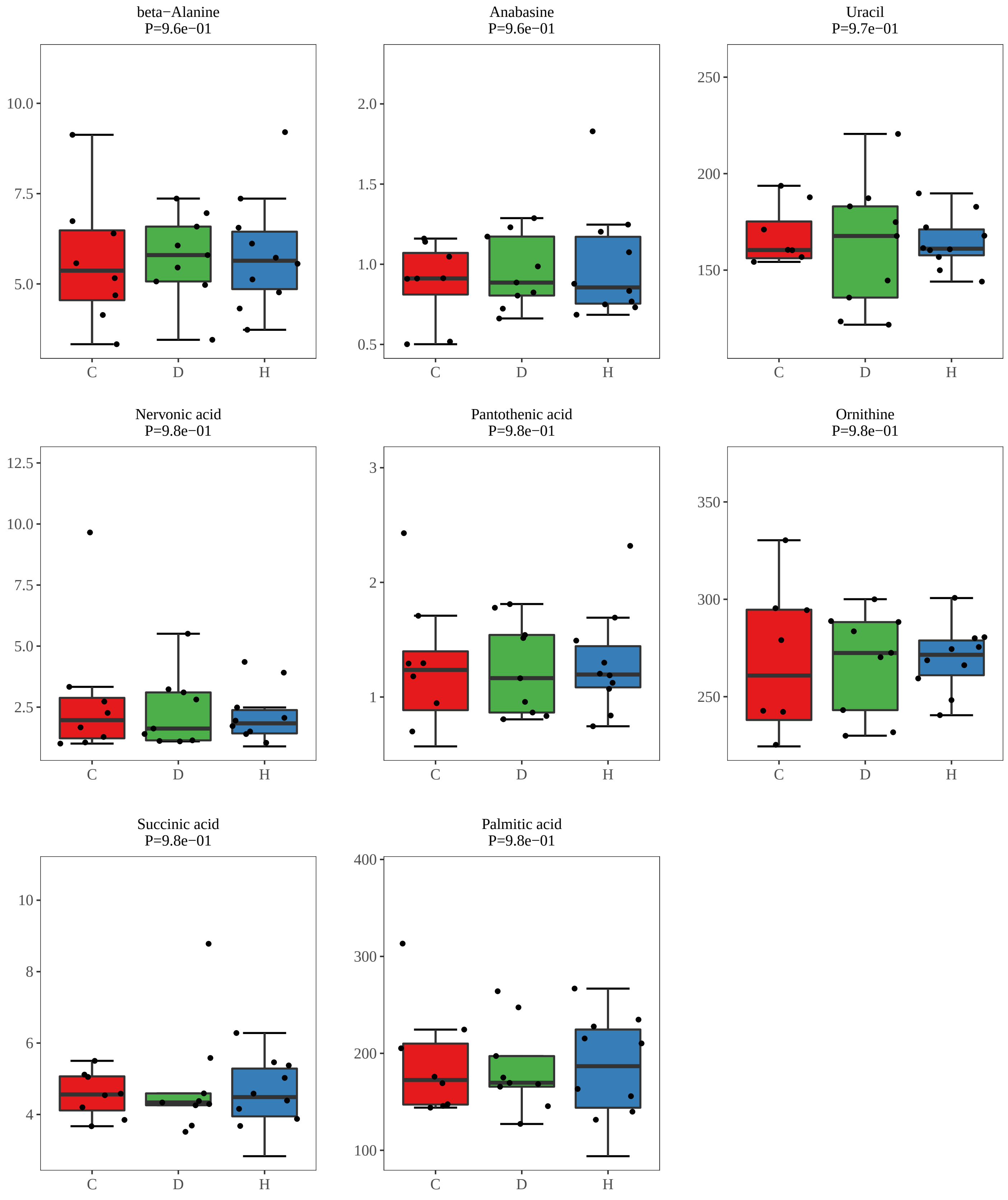
beta−Alanine
P=9.6e−01
10.0
7.5
5.0
C
D
H
Anabasine
P=9.6e−01
2.0
1.5
1.0
0.5
C
D
H
Uracil
P=9.7e−01
250
200
150
C
D
H
Nervonic acid
P=9.8e−01
12.5
10.0
7.5
5.0
2.5
C
D
H
Pantothenic acid
P=9.8e−01
3
2
1
C
D
H
Ornithine
P=9.8e−01
350
300
250
C
D
H
Succinic acid
P=9.8e−01
10
8
6
4
C
D
H
Palmitic acid
P=9.8e−01
400
300
200
100
C
D
H
