# Supplementary material for: Yiai Fuzheng decoction inhibits triple-negative breast cancer by remodeling the immune microenvironment
Source: Front Immunol. 2025 Sep 30;16:1615631. doi: 10.3389/fimmu.2025.1615631 (PMC12518410; doi:10.3389/fimmu.2025.1615631)
Supplement: Supplementary file 2 [file DataSheet1.zip › Supplementary File 2/Treatment/C_vs_D_vs_H/04_Univariate_Analysis/AllMet_Boxplot_with_Points.pdf]

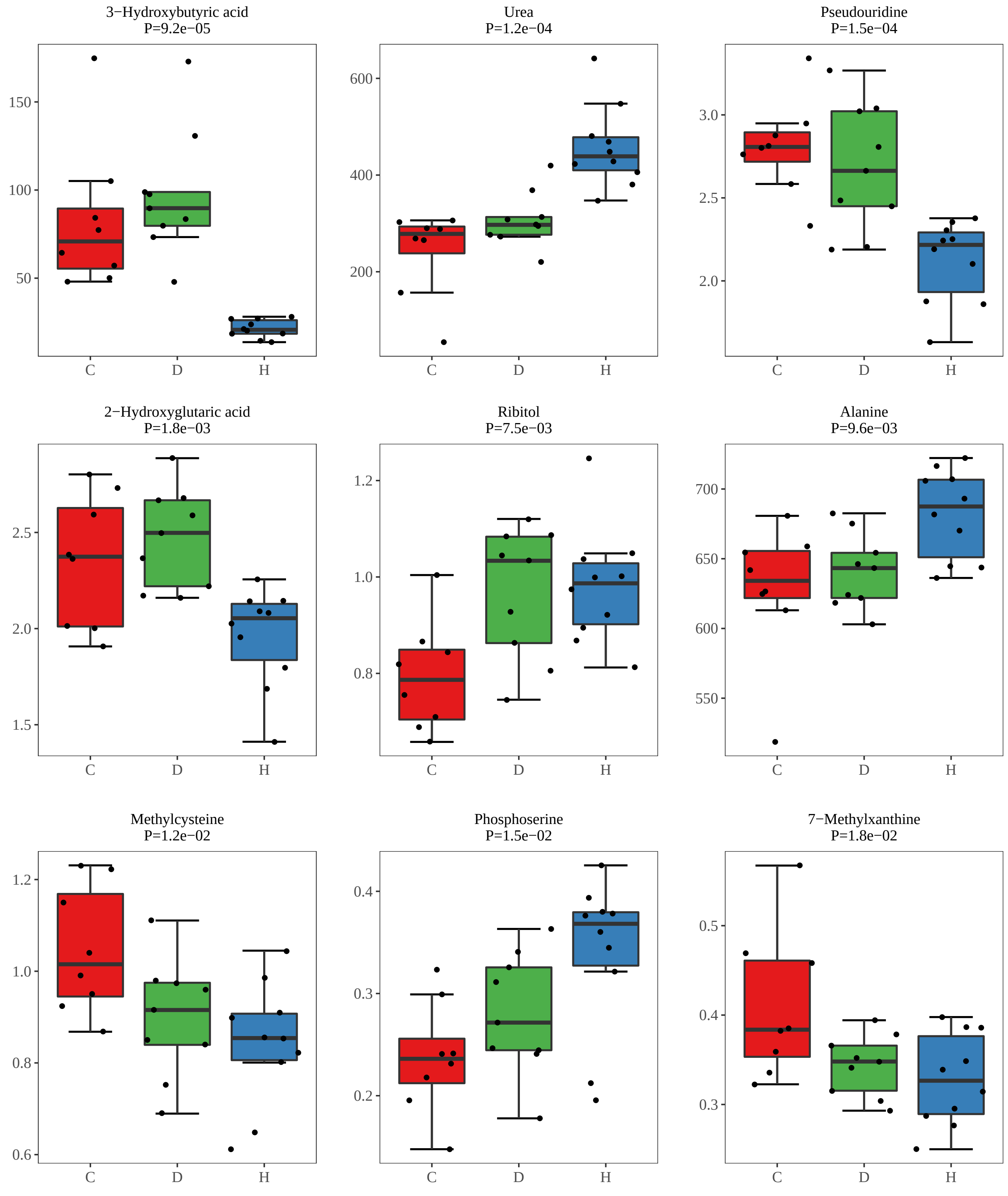
3−Hydroxybutyric acid
P=9.2e−05
150
100
50
C
D
H
Urea
P=1.2e−04
600
400
200
C
D
H
Pseudouridine
P=1.5e−04
3.0
2.5
2.0
C
D
H
2−Hydroxyglutaric acid
P=1.8e−03
2.5
2.0
1.5
C
D
H
Ribitol
P=7.5e−03
1.2
1.0
0.8
C
D
H
Alanine
P=9.6e−03
700
650
600
550
C
D
H
Methylcysteine
P=1.2e−02
1.2
1.0
0.8
0.6
C
D
H
Phosphoserine
P=1.5e−02
0.4
0.3
0.2
C
D
H
7−Methylxanthine
P=1.8e−02
0.5
0.4
0.3
C
D
H

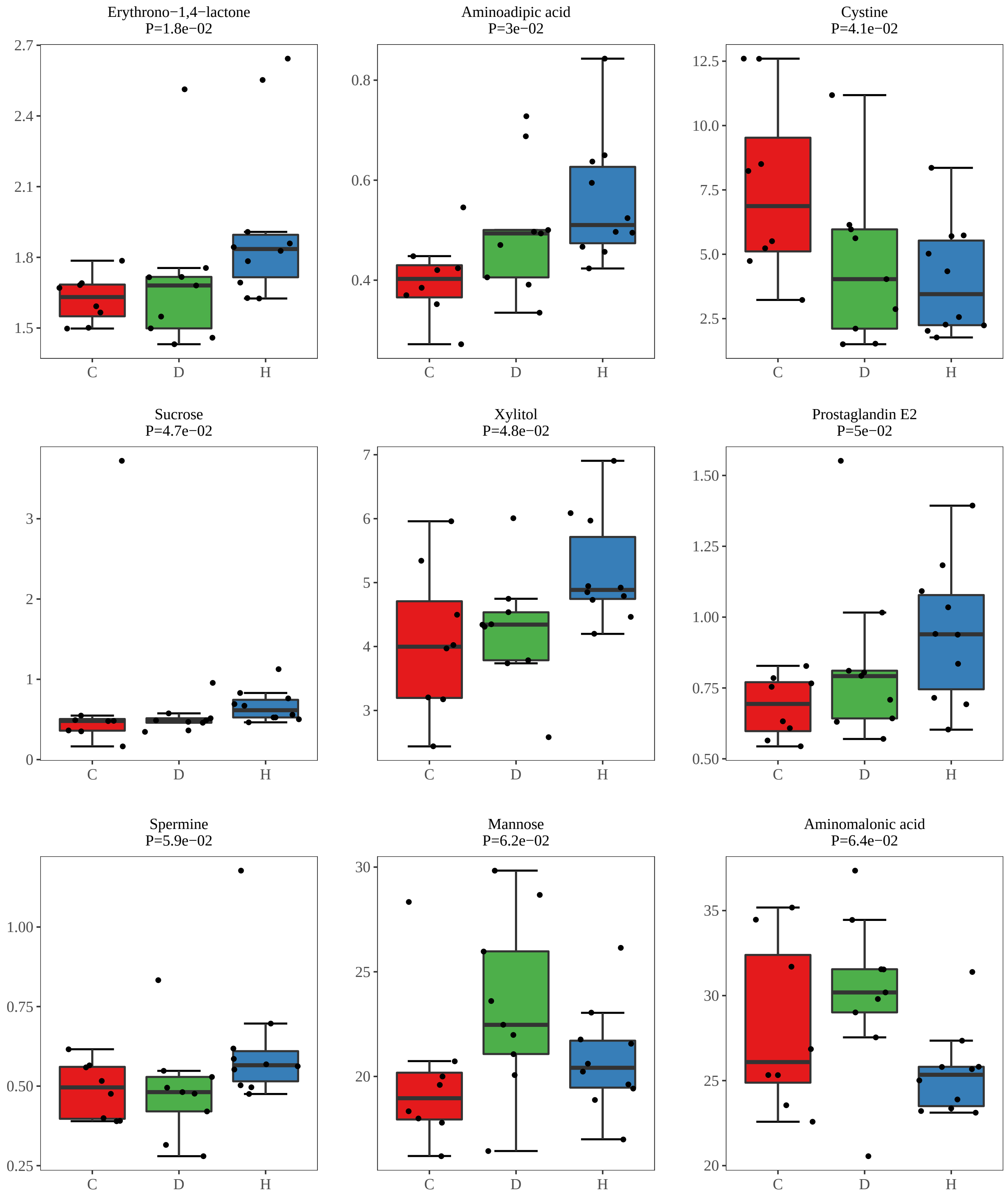

Erythrono−1,4−lactone
P=1.8e−02
2.7
2.4
2.1
1.8
1.5
C
D
H
Aminoadipic acid
P=3e−02
0.8
0.6
0.4
C
D
H
Cystine
P=4.1e−02
12.5
10.0
7.5
5.0
2.5
C
D
H
Sucrose
P=4.7e−02
3
2
1
0
C
D
H
Xylitol
P=4.8e−02
7
6
5
4
3
C
D
H
Prostaglandin E2
P=5e−02
1.50
1.25
1.00
0.75
0.50
C
D
H
Spermine
P=5.9e−02
1.00
0.75
0.50
0.25
C
D
H
Mannose
P=6.2e−02
30
25
20
C
D
H
Aminomalonic acid
P=6.4e−02
35
30
25
20
C
D
H

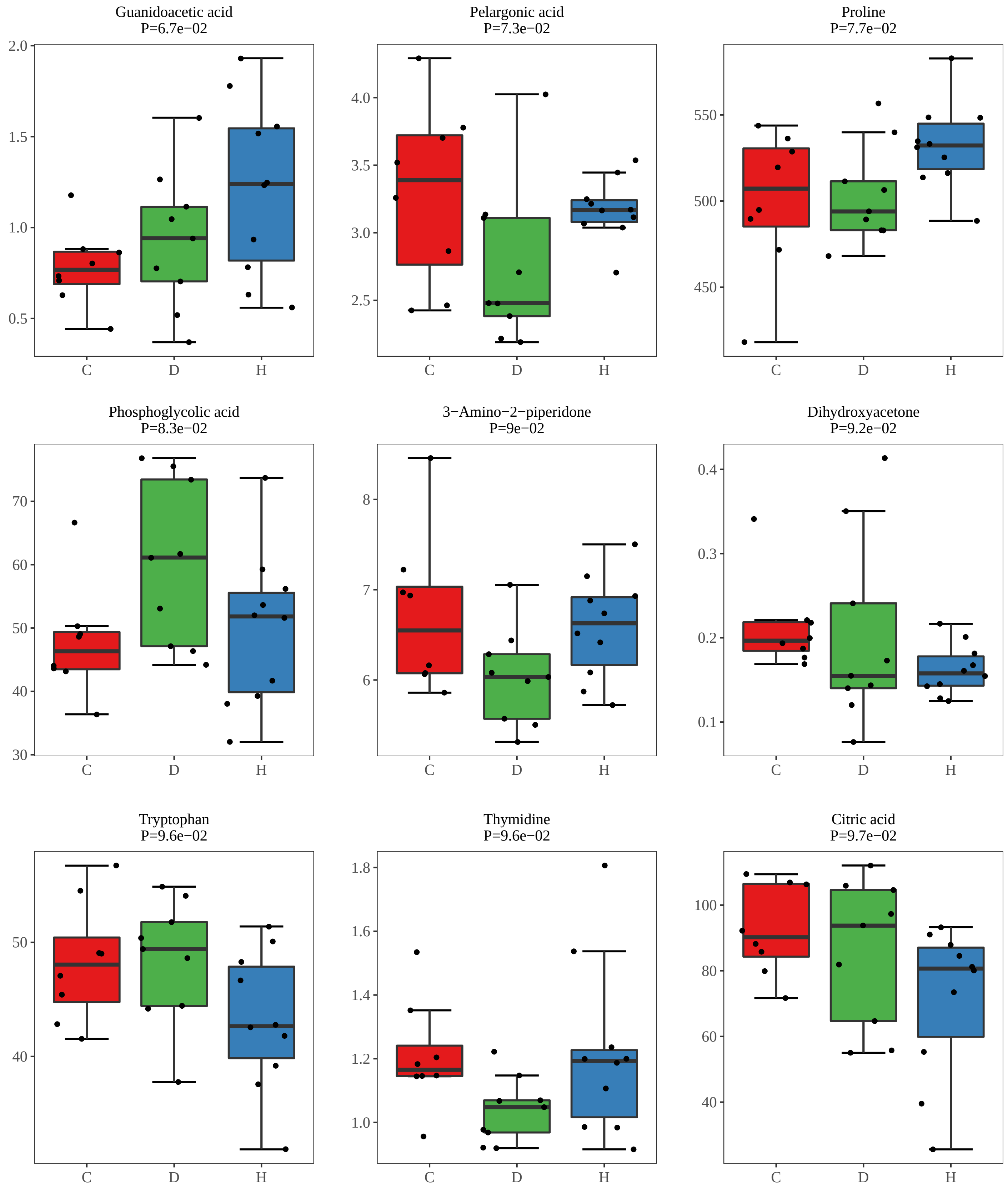

Guanidoacetic acid
P=6.7e−02
2.0
1.5
1.0
0.5
C
D
H
Pelargonic acid
P=7.3e−02
4.0
3.5
3.0
2.5
C
D
H
Proline
P=7.7e−02
550
500
450
C
D
H
Phosphoglycolic acid
P=8.3e−02
70
60
50
40
30
C
D
H
3−Amino−2−piperidone
P=9e−02
8
7
6
C
D
H
Dihydroxyacetone
P=9.2e−02
0.4
0.3
0.2
0.1
C
D
H
Tryptophan
P=9.6e−02
50
40
C
D
H
Thymidine
P=9.6e−02
1.8
1.6
1.4
1.2
1.0
C
D
H
Citric acid
P=9.7e−02
100
80
60
40
C
D
H

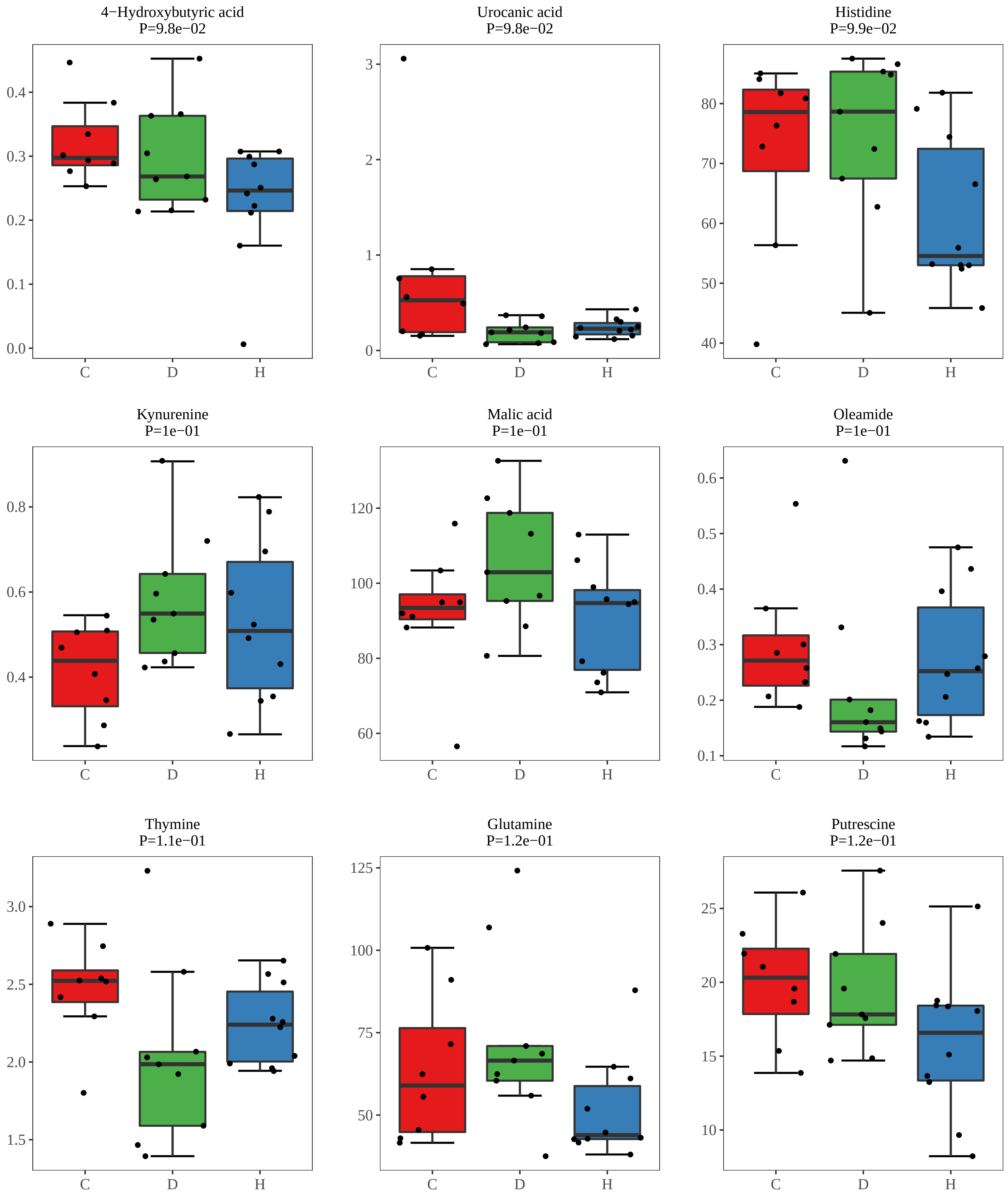
4−Hydroxybutyric acid
P=9.8e−02
Urocanic acid
P=9.8e−02
Histidine
P=9.9e−02
Kynurenine
P=1e−01
Malic acid
P=1e−01
Oleamide
P=1e−01
Thymine
P=1.1e−01
Glutamine
P=1.2e−01
Putrescine
P=1.2e−01
C
D
H

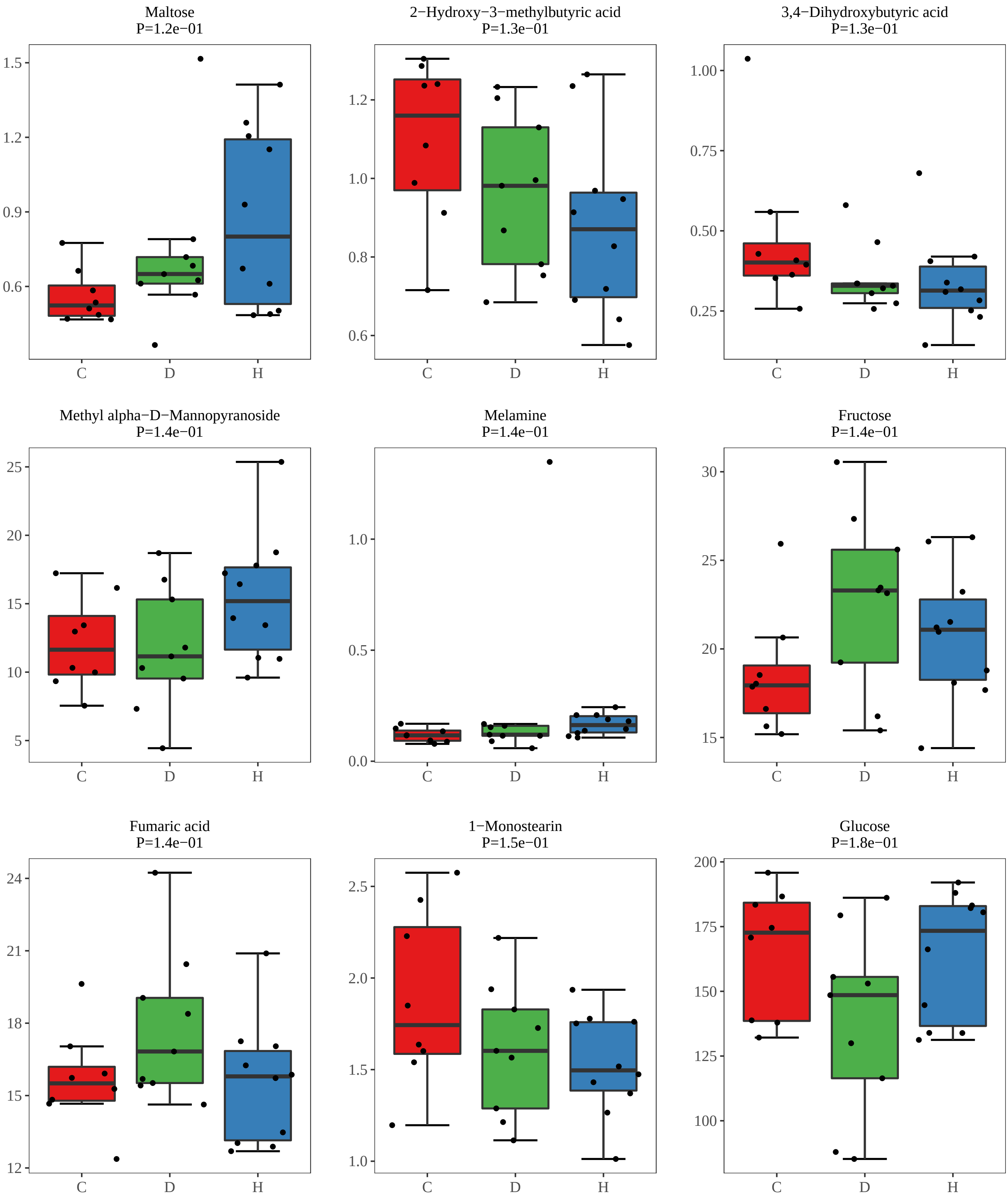
Maltose
P=1.2e−01
1.5
1.2
0.9
0.6
C
D
H
2−Hydroxy−3−methylbutyric acid
P=1.3e−01
1.2
1.0
0.8
0.6
C
D
H
3,4−Dihydroxybutyric acid
P=1.3e−01
1.00
0.75
0.50
0.25
C
D
H
Methyl alpha−D−Mannopyranoside
P=1.4e−01
25
20
15
10
5
C
D
H
Melamine
P=1.4e−01
1.0
0.5
0.0
C
D
H
Fructose
P=1.4e−01
30
25
20
15
C
D
H
Fumaric acid
P=1.4e−01
24
21
18
15
12
C
D
H
1−Monostearin
P=1.5e−01
2.5
2.0
1.5
1.0
C
D
H
Glucose
P=1.8e−01
200
175
150
125
100
C
D
H

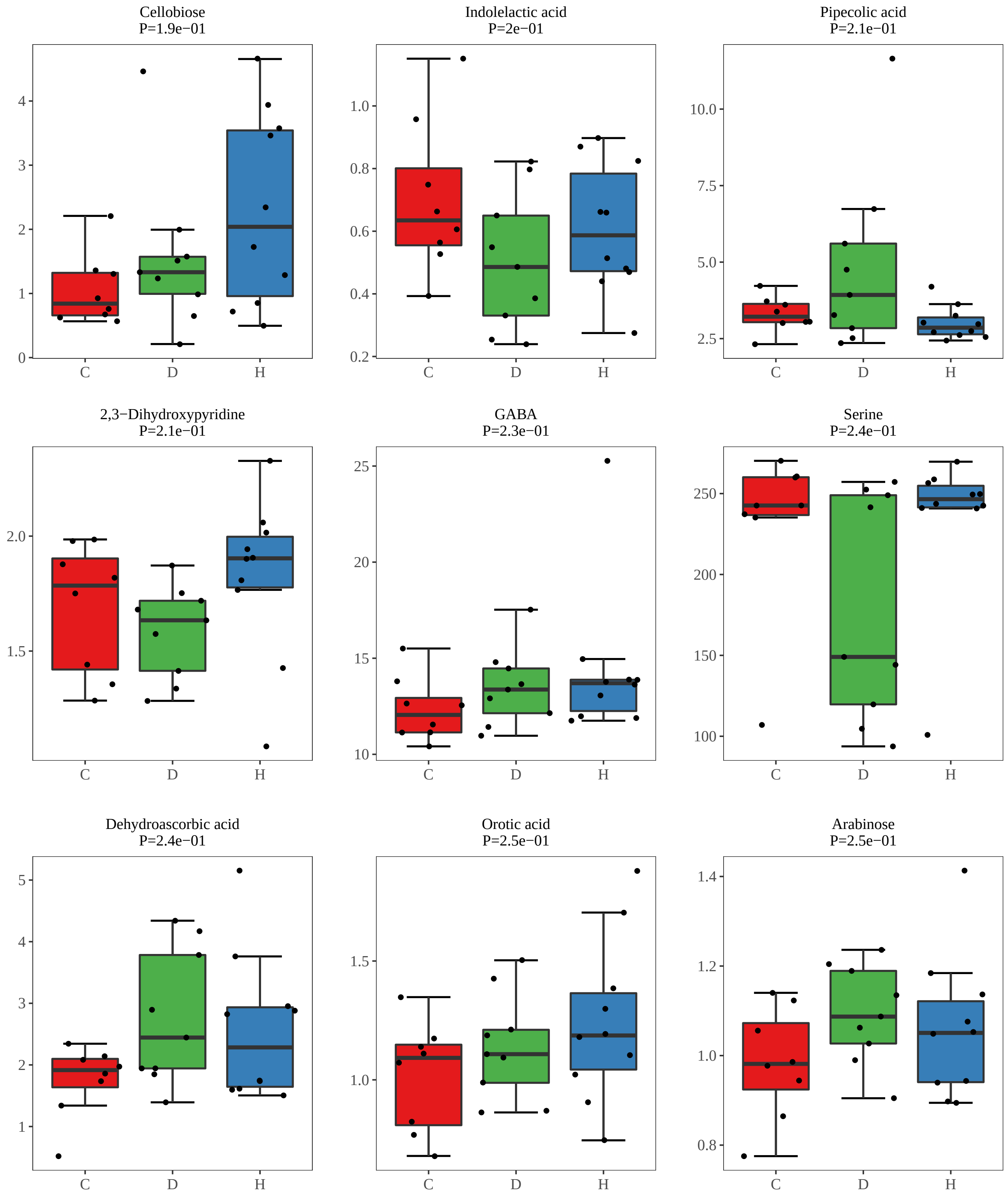
Cellobiose
P=1.9e−01
Indolelactic acid
P=2e−01
Pipecolic acid
P=2.1e−01
2,3−Dihydroxypyridine
P=2.1e−01
GABA
P=2.3e−01
Serine
P=2.4e−01
Dehydroascorbic acid
P=2.4e−01
Orotic acid
P=2.5e−01
Arabinose
P=2.5e−01
C
D
H

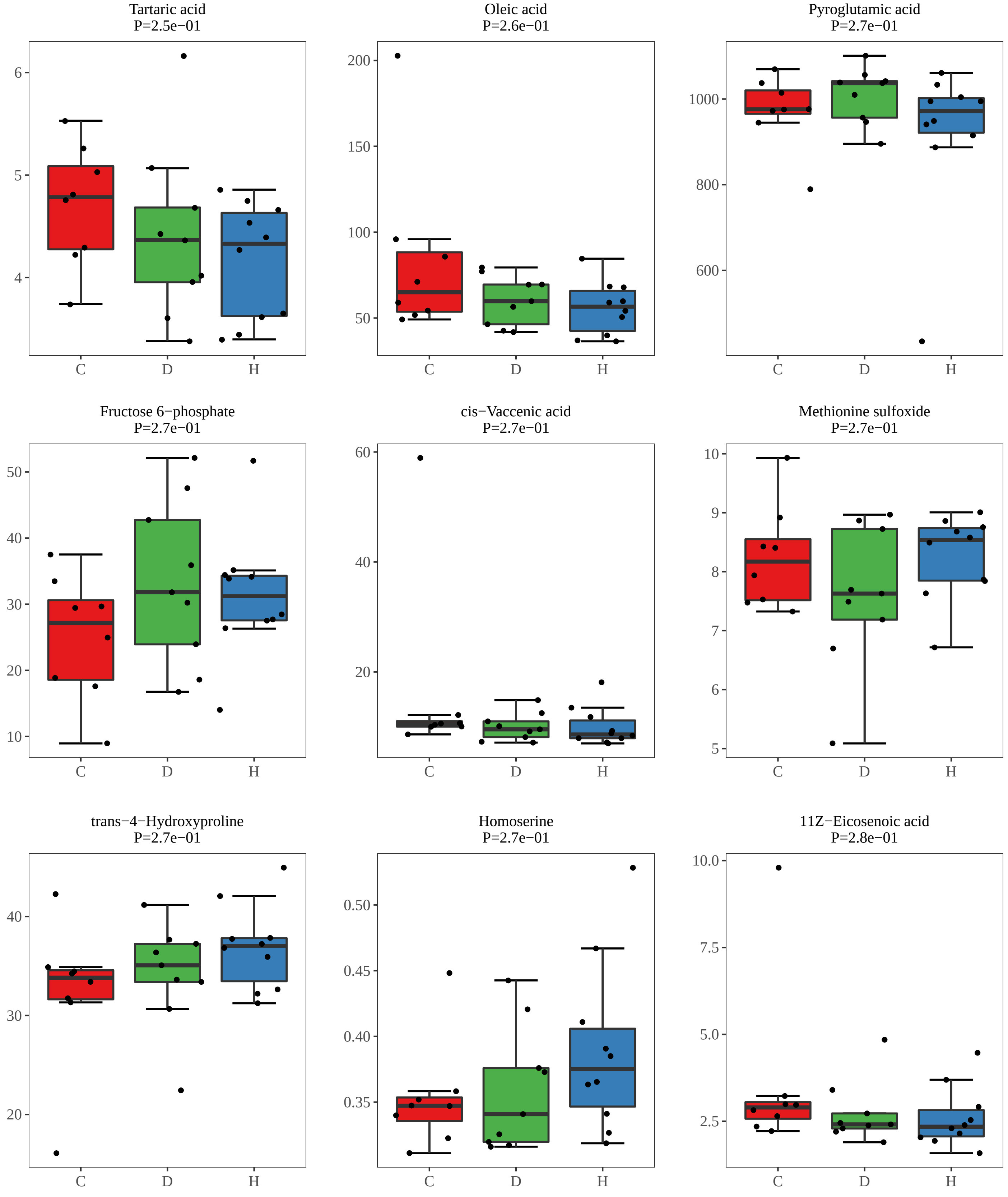
Tartaric acid
P=2.5e−01
Oleic acid
P=2.6e−01
Pyroglutamic acid
P=2.7e−01
Fructose 6−phosphate
P=2.7e−01
cis−Vaccenic acid
P=2.7e−01
Methionine sulfoxide
P=2.7e−01
trans−4−Hydroxyproline
P=2.7e−01
Homoserine
P=2.7e−01
11Z−Eicosenoic acid
P=2.8e−01
C
D
H

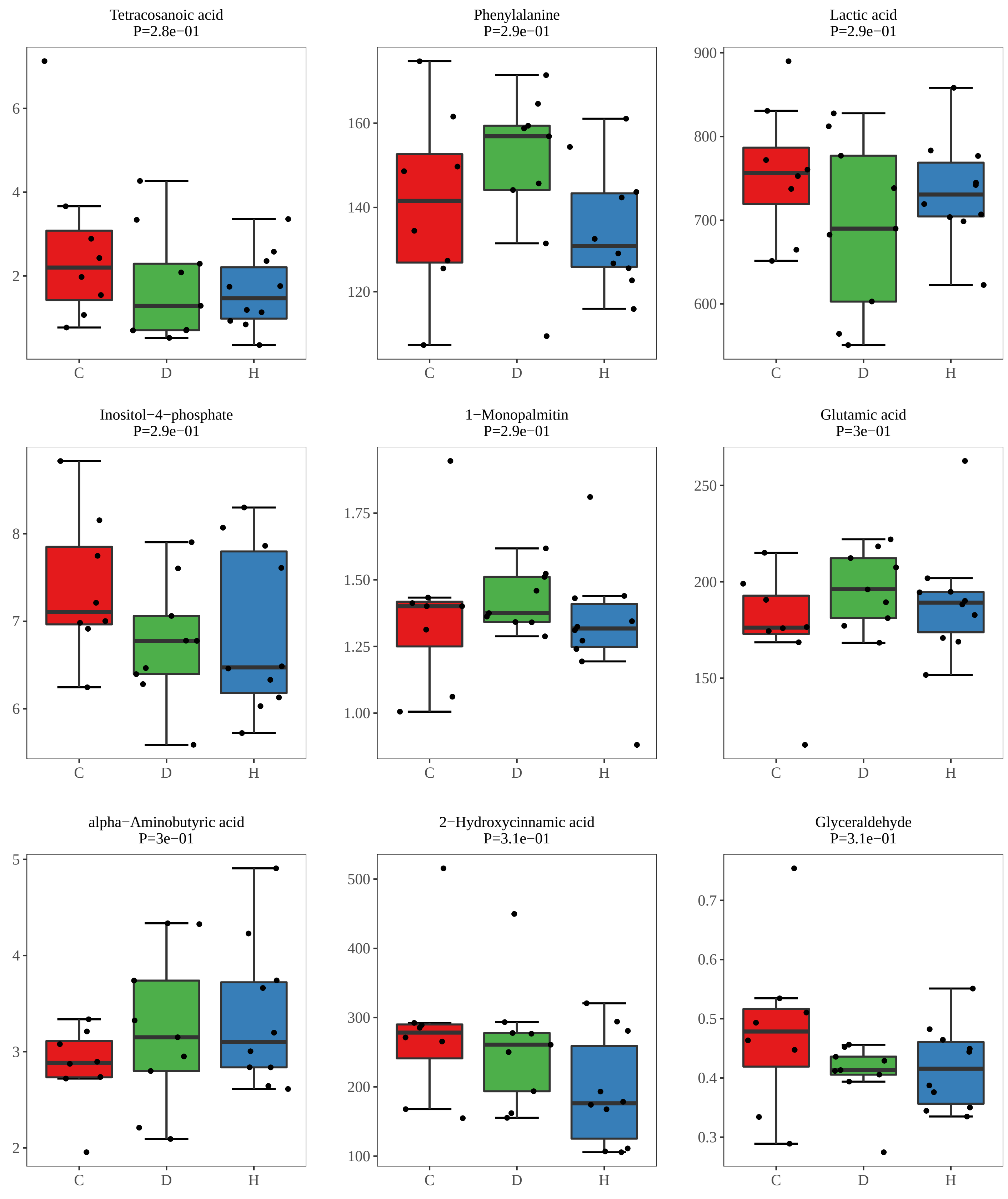

Tetracosanoic acid
P=2.8e−01
Phenylalanine
P=2.9e−01
Lactic acid
P=2.9e−01
Inositol−4−phosphate
P=2.9e−01
1−Monopalmitin
P=2.9e−01
Glutamic acid
P=3e−01
alpha−Aminobutyric acid
P=3e−01
2−Hydroxycinnamic acid
P=3.1e−01
Glyceraldehyde
P=3.1e−01
C
D
H

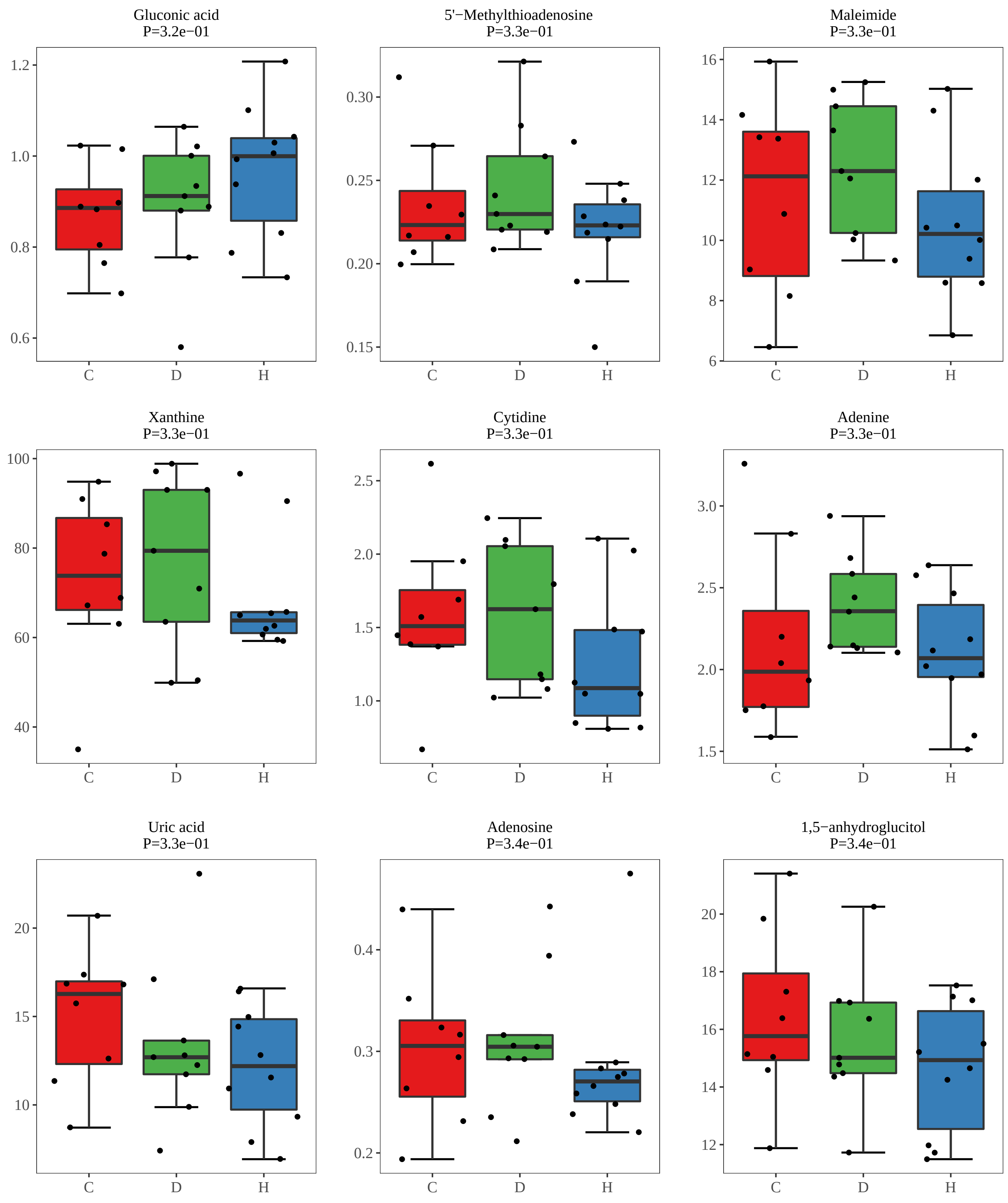
Gluconic acid
P=3.2e−01
1.2
1.0
0.8
0.6
C
D
H
5'−Methylthioadenosine
P=3.3e−01
0.30
0.25
0.20
0.15
C
D
H
Maleimide
P=3.3e−01
16
14
12
10
8
6
C
D
H
Xanthine
P=3.3e−01
100
80
60
40
C
D
H
Cytidine
P=3.3e−01
2.5
2.0
1.5
1.0
C
D
H
Adenine
P=3.3e−01
3.0
2.5
2.0
1.5
C
D
H
Uric acid
P=3.3e−01
20
15
10
C
D
H
Adenosine
P=3.4e−01
0.4
0.3
0.2
C
D
H
1,5−anhydroglucitol
P=3.4e−01
20
18
16
14
12
C
D
H

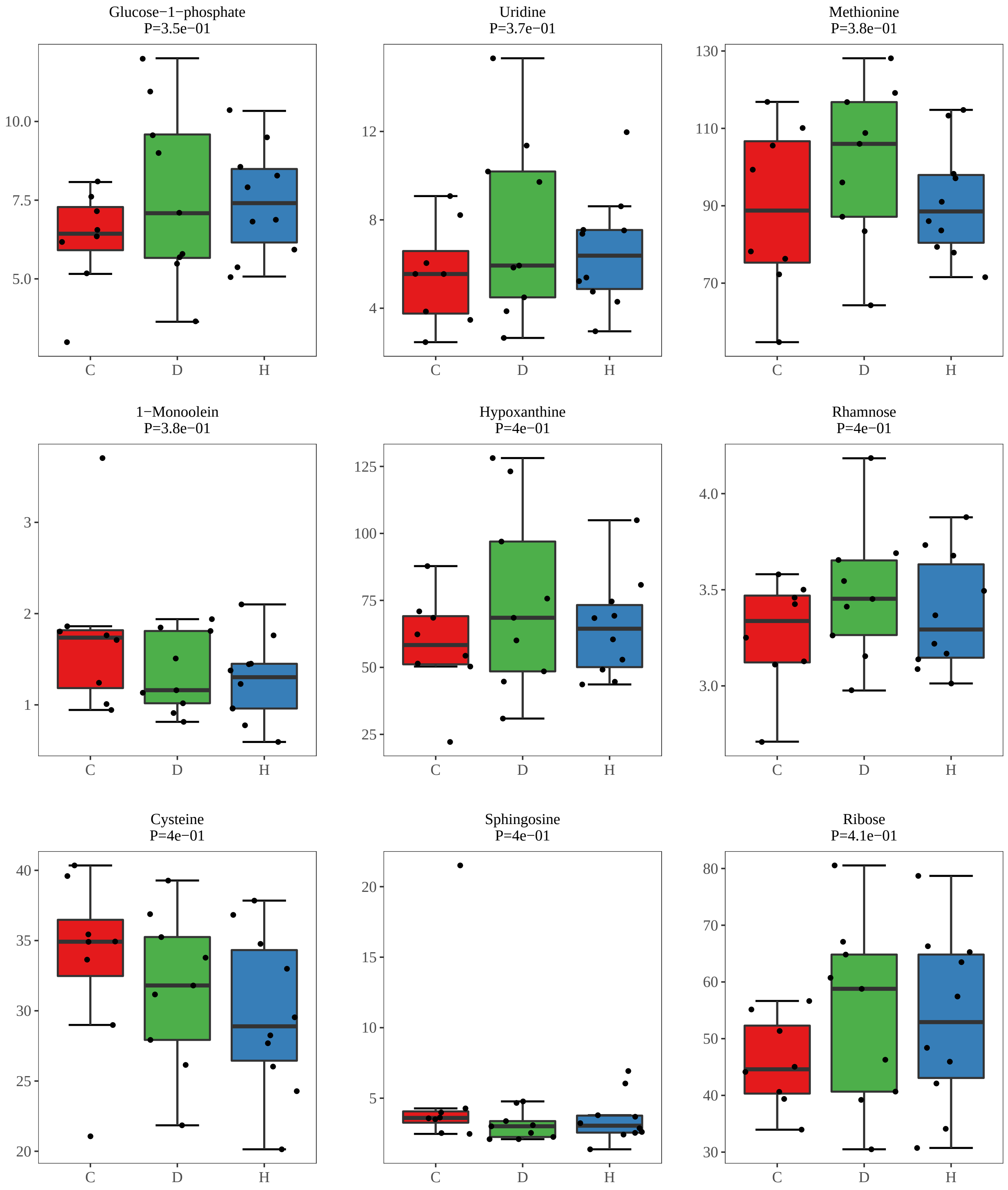
Glucose−1−phosphate
P=3.5e−01
Uridine
P=3.7e−01
Methionine
P=3.8e−01
1−Monoolein
P=3.8e−01
Hypoxanthine
P=4e−01
Rhamnose
P=4e−01
Cysteine
P=4e−01
Sphingosine
P=4e−01
Ribose
P=4.1e−01
C
D
H

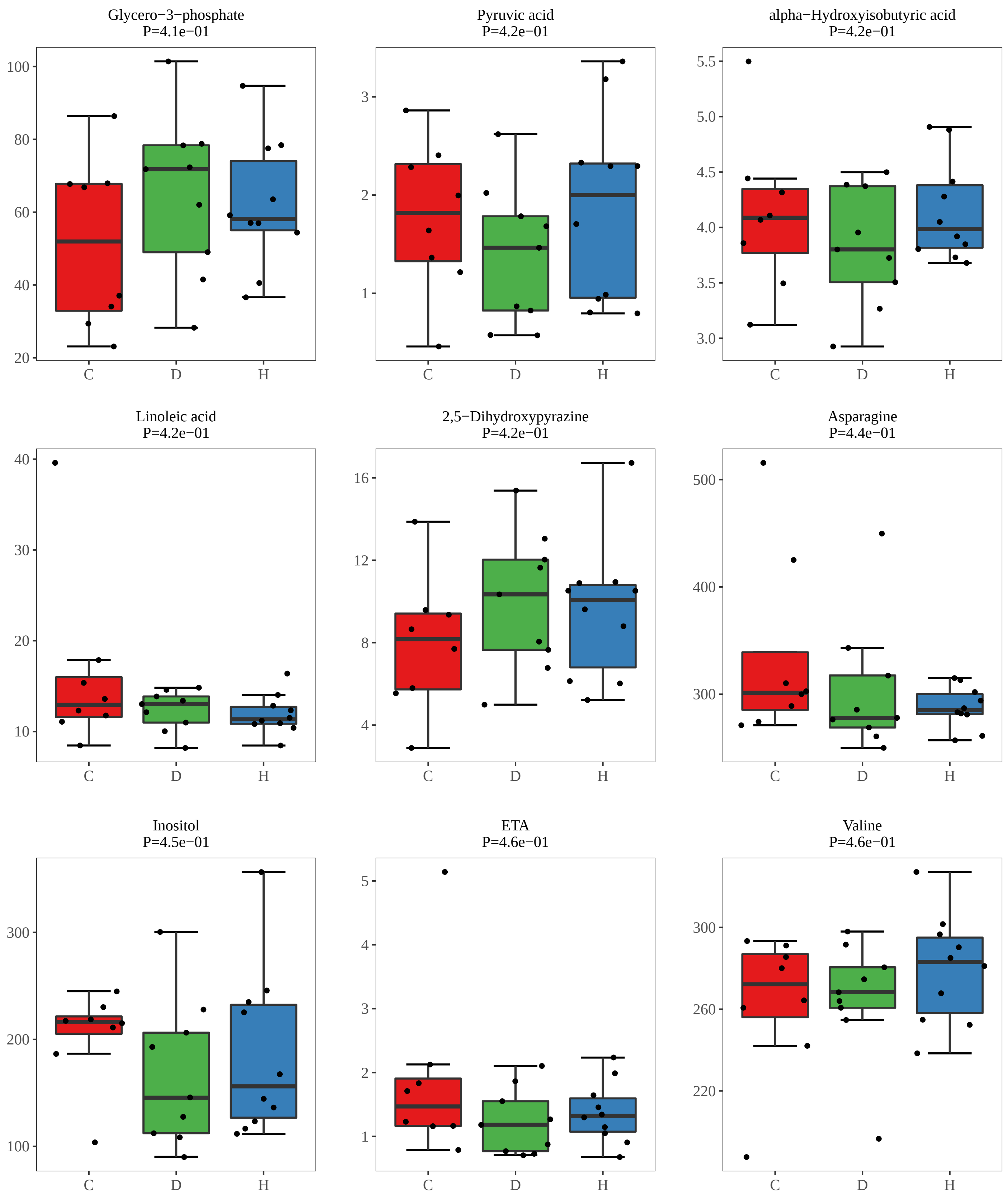

Glycero−3−phosphate
P=4.1e−01
Pyruvic acid
P=4.2e−01
alpha−Hydroxyisobutyric acid
P=4.2e−01
Linoleic acid
P=4.2e−01
2,5−Dihydroxypyrazine
P=4.2e−01
Asparagine
P=4.4e−01
Inositol
P=4.5e−01
ETA
P=4.6e−01
Valine
P=4.6e−01
C
D
H

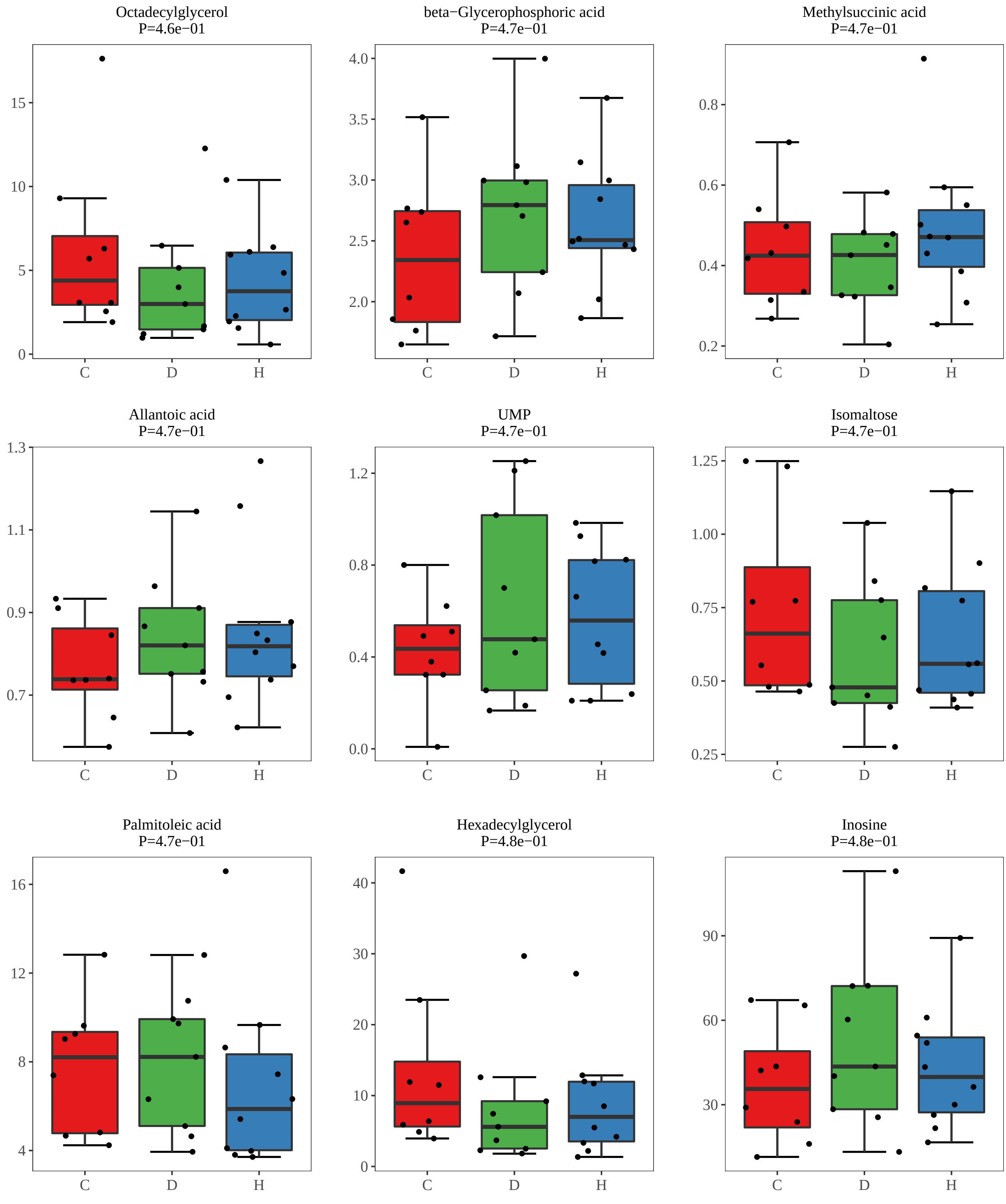

Octadecylglycerol
P=4.6e−01
0
5
10
15
C
D
H
beta−Glycerophosphoric acid
P=4.7e−01
2.0
2.5
3.0
3.5
4.0
C
D
H
Methylsuccinic acid
P=4.7e−01
0.2
0.4
0.6
0.8
C
D
H
Allantoic acid
P=4.7e−01
0.7
0.9
1.1
1.3
C
D
H
UMP
P=4.7e−01
0.0
0.4
0.8
1.2
C
D
H
Isomaltose
P=4.7e−01
0.25
0.50
0.75
1.00
1.25
C
D
H
Palmitoleic acid
P=4.7e−01
4
8
12
16
C
D
H
Hexadecylglycerol
P=4.8e−01
0
10
20
30
40
C
D
H
Inosine
P=4.8e−01
30
60
90
C
D
H

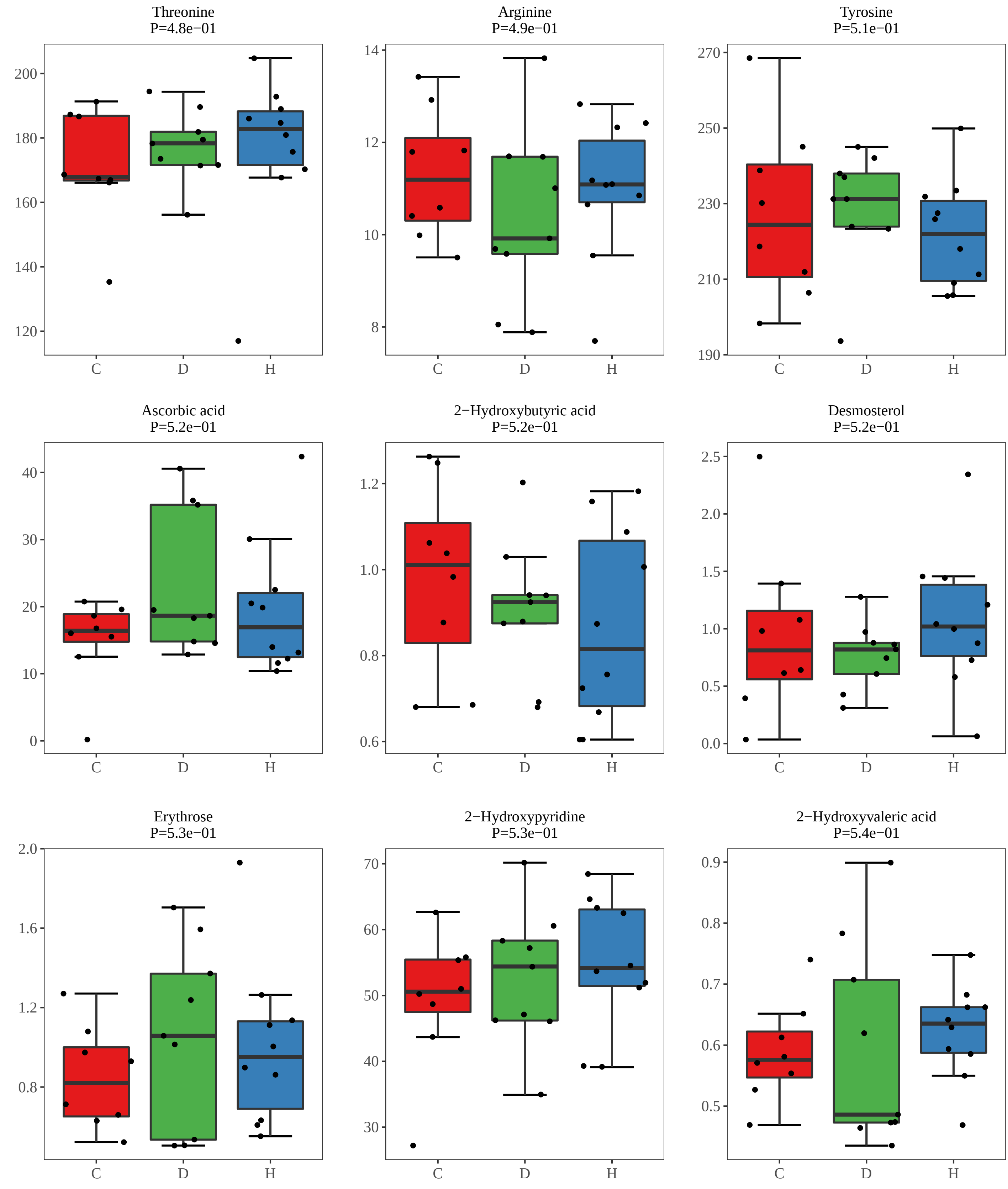
Threonine
P=4.8e−01
Arginine
P=4.9e−01
Tyrosine
P=5.1e−01
Ascorbic acid
P=5.2e−01
2−Hydroxybutyric acid
P=5.2e−01
Desmosterol
P=5.2e−01
Erythrose
P=5.3e−01
2−Hydroxypyridine
P=5.3e−01
2−Hydroxyvaleric acid
P=5.4e−01
C
D
H

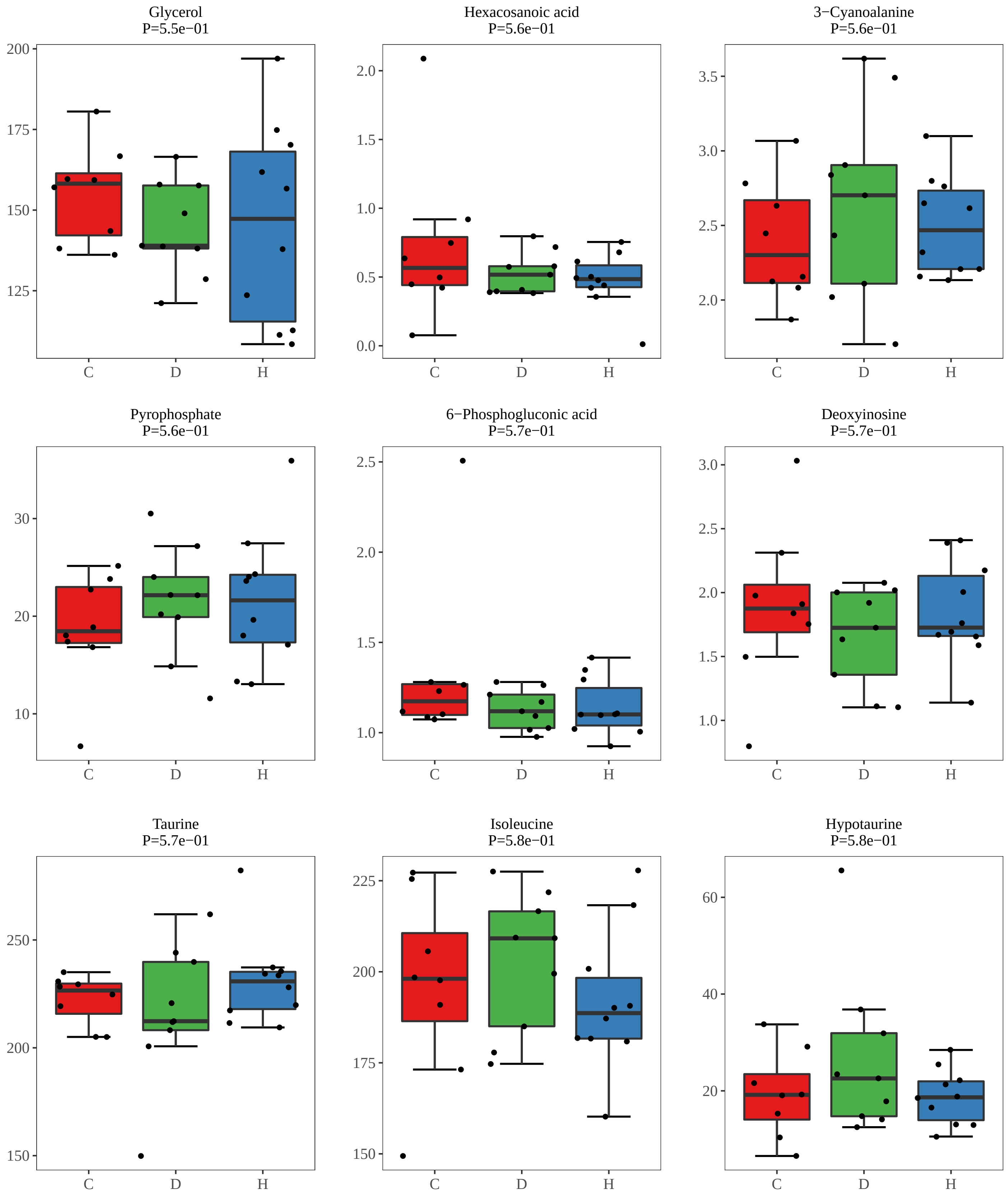
Glycerol
P=5.5e−01
Hexacosanoic acid
P=5.6e−01
3−Cyanoalanine
P=5.6e−01
Pyrophosphate
P=5.6e−01
6−Phosphogluconic acid
P=5.7e−01
Deoxyinosine
P=5.7e−01
Taurine
P=5.7e−01
Isoleucine
P=5.8e−01
Hypotaurine
P=5.8e−01
C
D
H

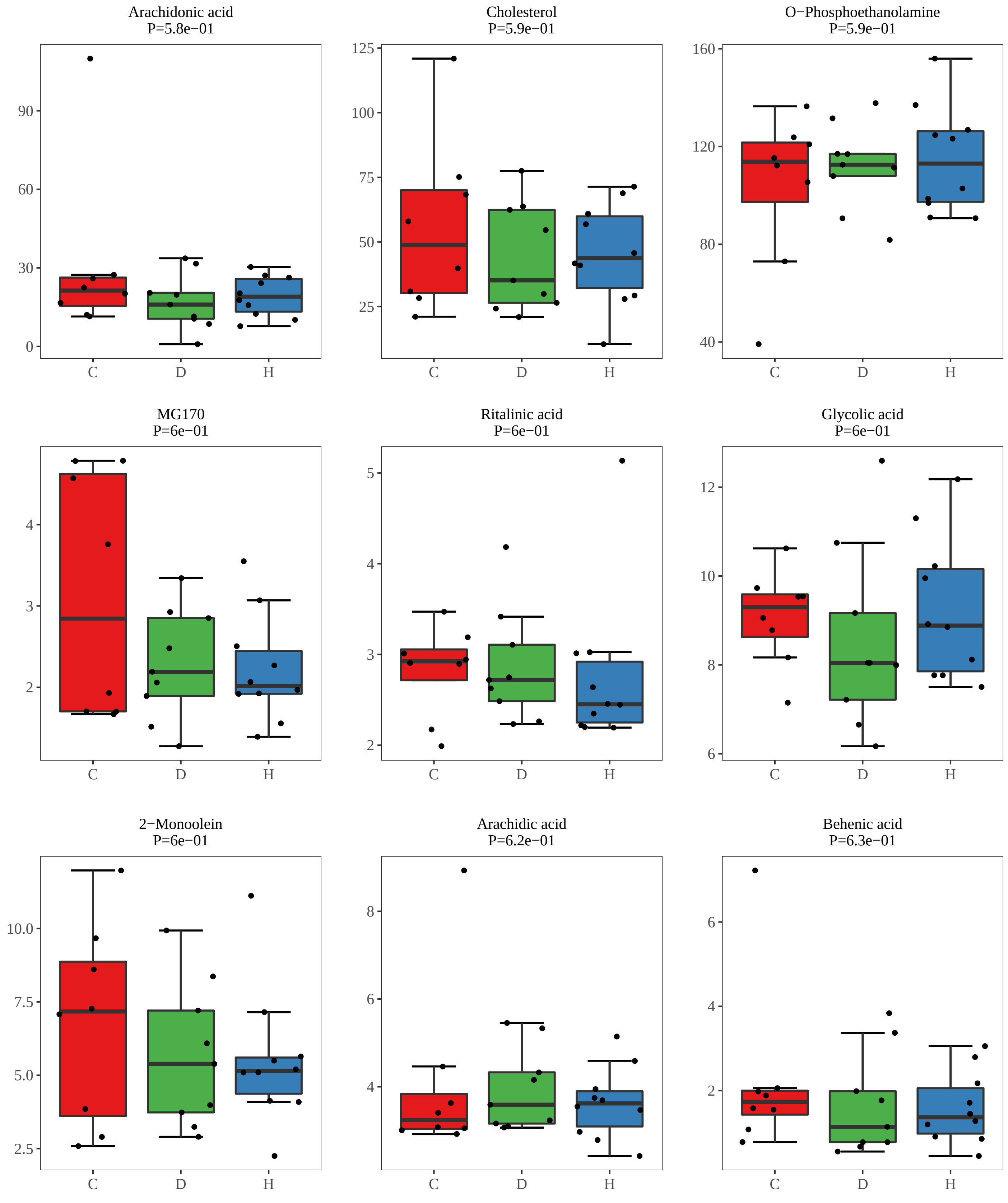

Arachidonic acid
P=5.8e−01
Cholesterol
P=5.9e−01
O−Phosphoethanolamine
P=5.9e−01
MG170
P=6e−01
Ritalinic acid
P=6e−01
Glycolic acid
P=6e−01
2−Monoolein
P=6e−01
Arachidic acid
P=6.2e−01
Behenic acid
P=6.3e−01
C
D
H

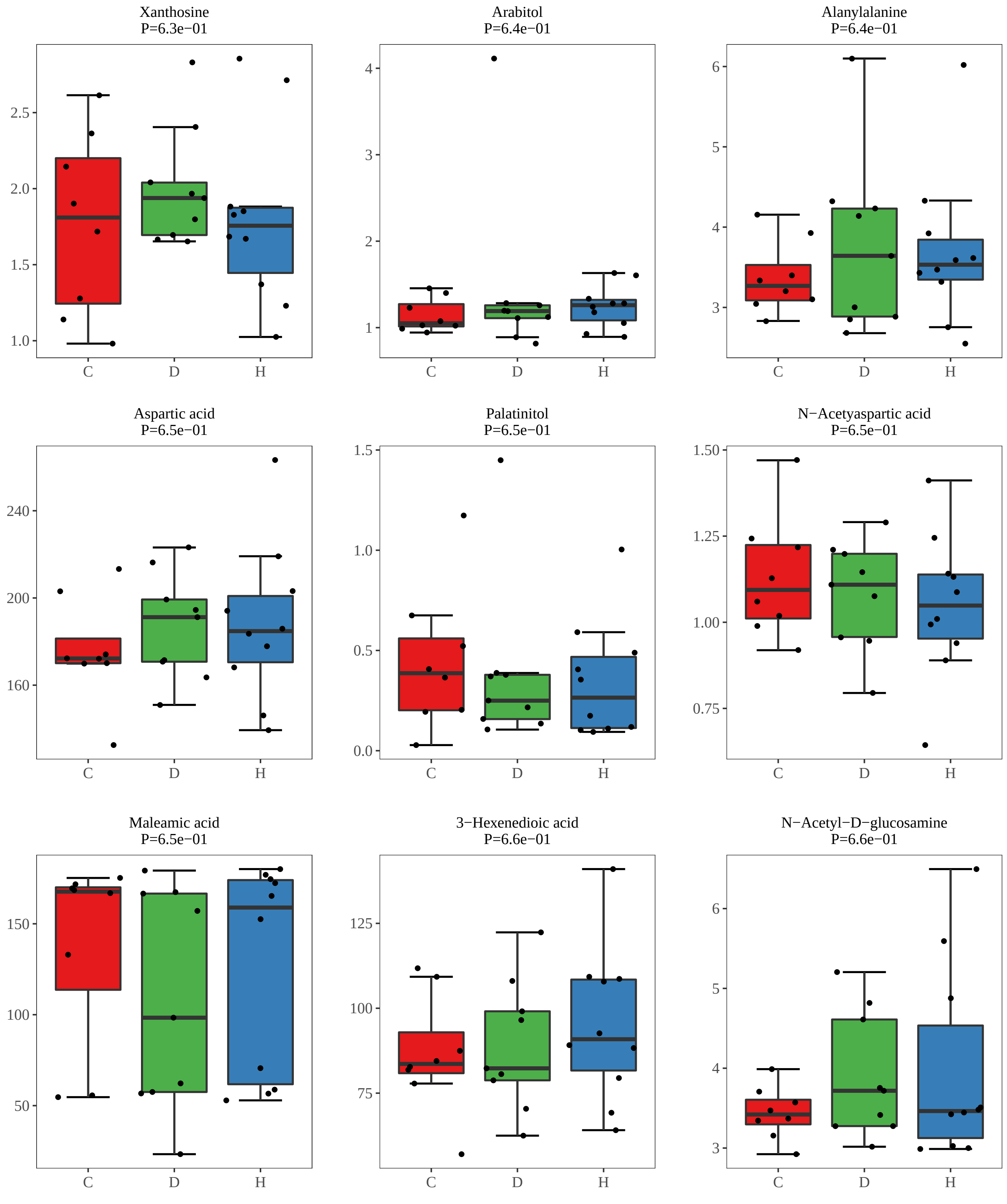

Xanthosine
P=6.3e−01
2.5
2.0
1.5
1.0
Arabitol
P=6.4e−01
4
3
2
1
Alanylalanine
P=6.4e−01
6
5
4
3
Aspartic acid
P=6.5e−01
240
200
160
Palatinitol
P=6.5e−01
1.5
1.0
0.5
0.0
N−Acetyaspartic acid
P=6.5e−01
1.50
1.25
1.00
0.75
Maleamic acid
P=6.5e−01
150
100
50
3−Hexenedioic acid
P=6.6e−01
125
100
75
N−Acetyl−D−glucosamine
P=6.6e−01
6
5
4
3
C
D
H

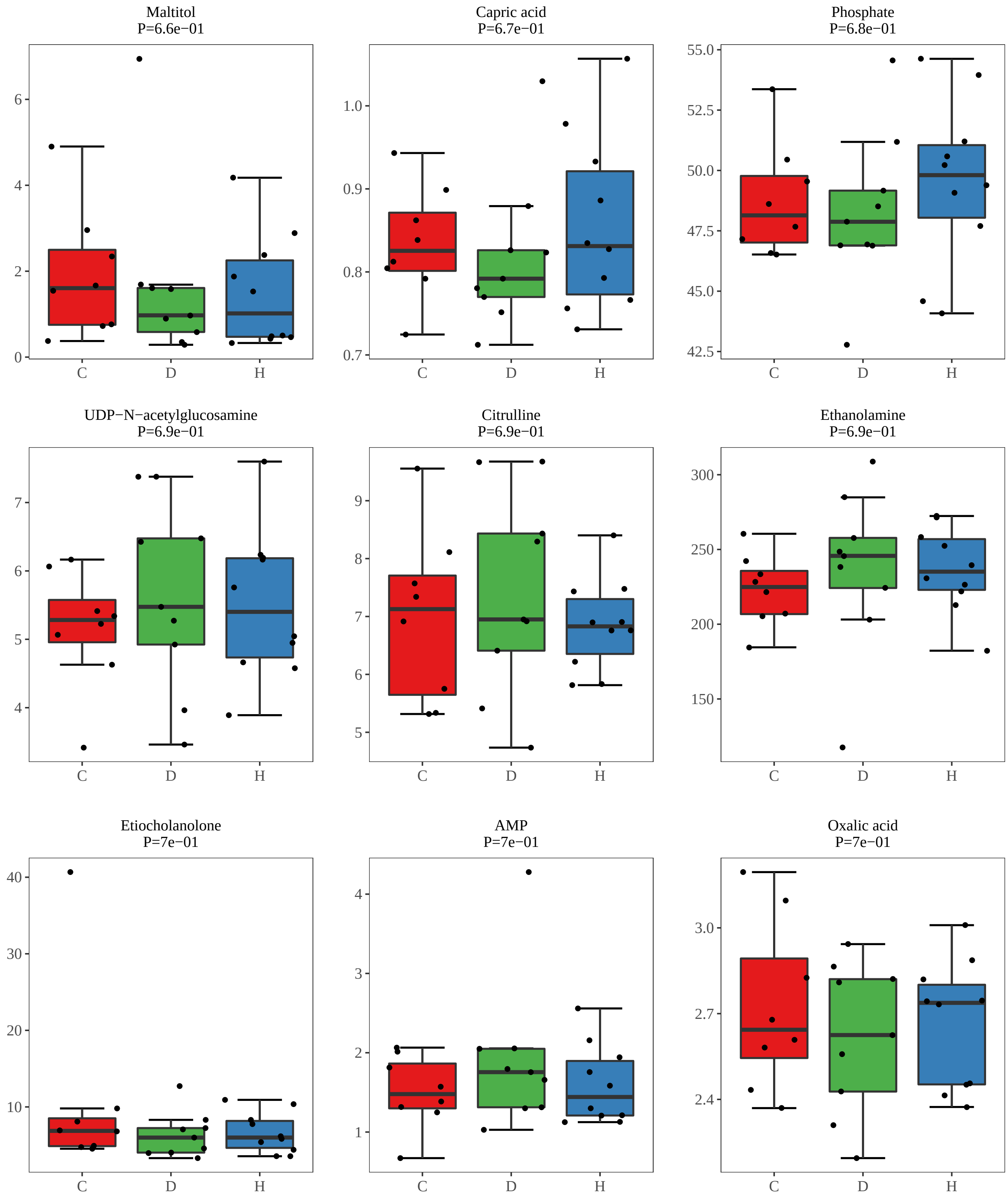
Maltitol
P=6.6e−01
0
2
4
6
C
D
H
Capric acid
P=6.7e−01
0.7
0.8
0.9
1.0
C
D
H
Phosphate
P=6.8e−01
42.5
45.0
47.5
50.0
52.5
55.0
C
D
H
UDP−N−acetylglucosamine
P=6.9e−01
4
5
6
7
C
D
H
Citrulline
P=6.9e−01
5
6
7
8
9
C
D
H
Ethanolamine
P=6.9e−01
150
200
250
300
C
D
H
Etiocholanolone
P=7e−01
10
20
30
40
C
D
H
AMP
P=7e−01
1
2
3
4
C
D
H
Oxalic acid
P=7e−01
2.4
2.7
3.0
C
D
H

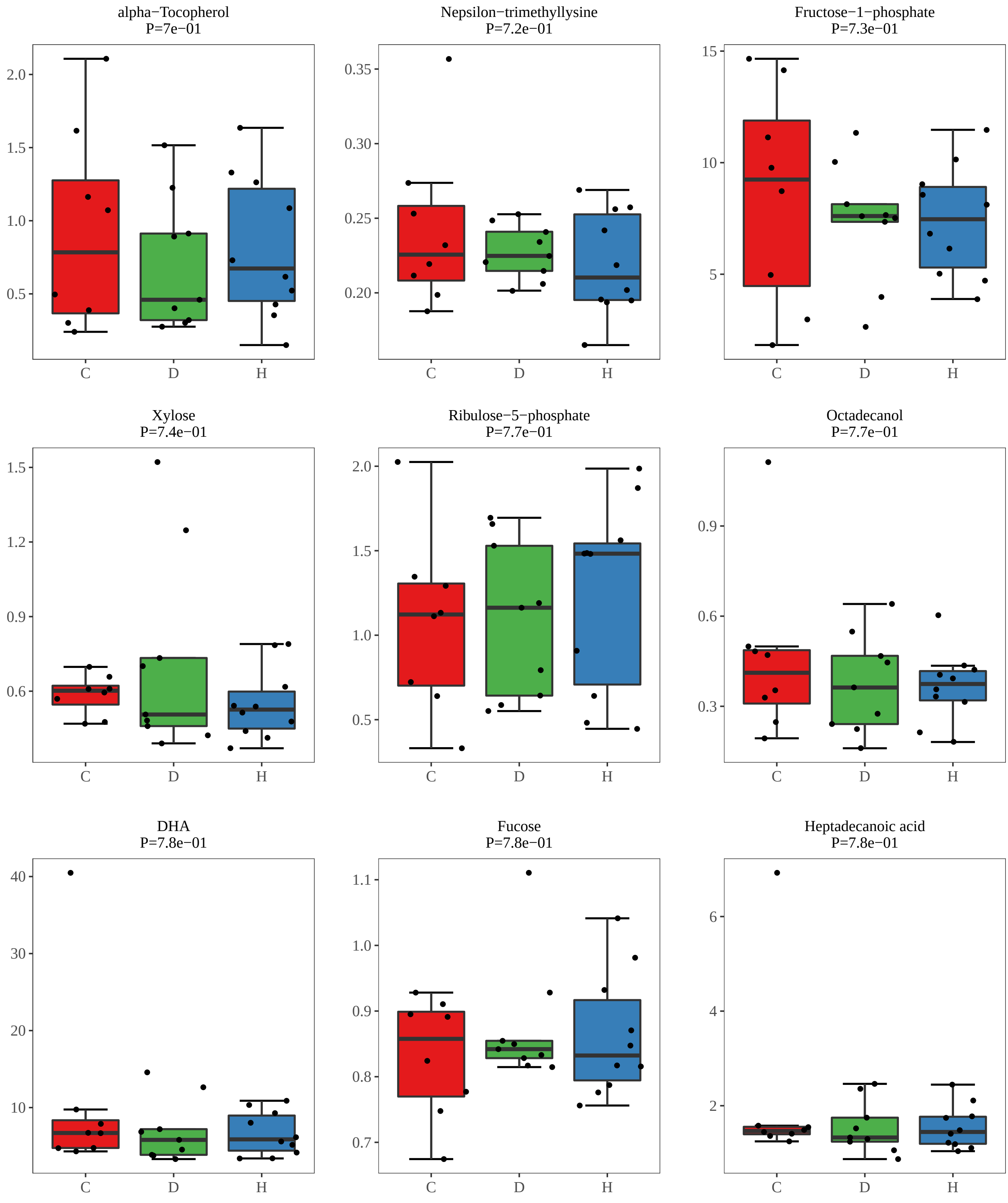

alpha−Tocopherol
P=7e−01
Nepsilon−trimethyllysine
P=7.2e−01
Fructose−1−phosphate
P=7.3e−01
Xylose
P=7.4e−01
Ribulose−5−phosphate
P=7.7e−01
Octadecanol
P=7.7e−01
DHA
P=7.8e−01
Fucose
P=7.8e−01
Heptadecanoic acid
P=7.8e−01
C
D
H

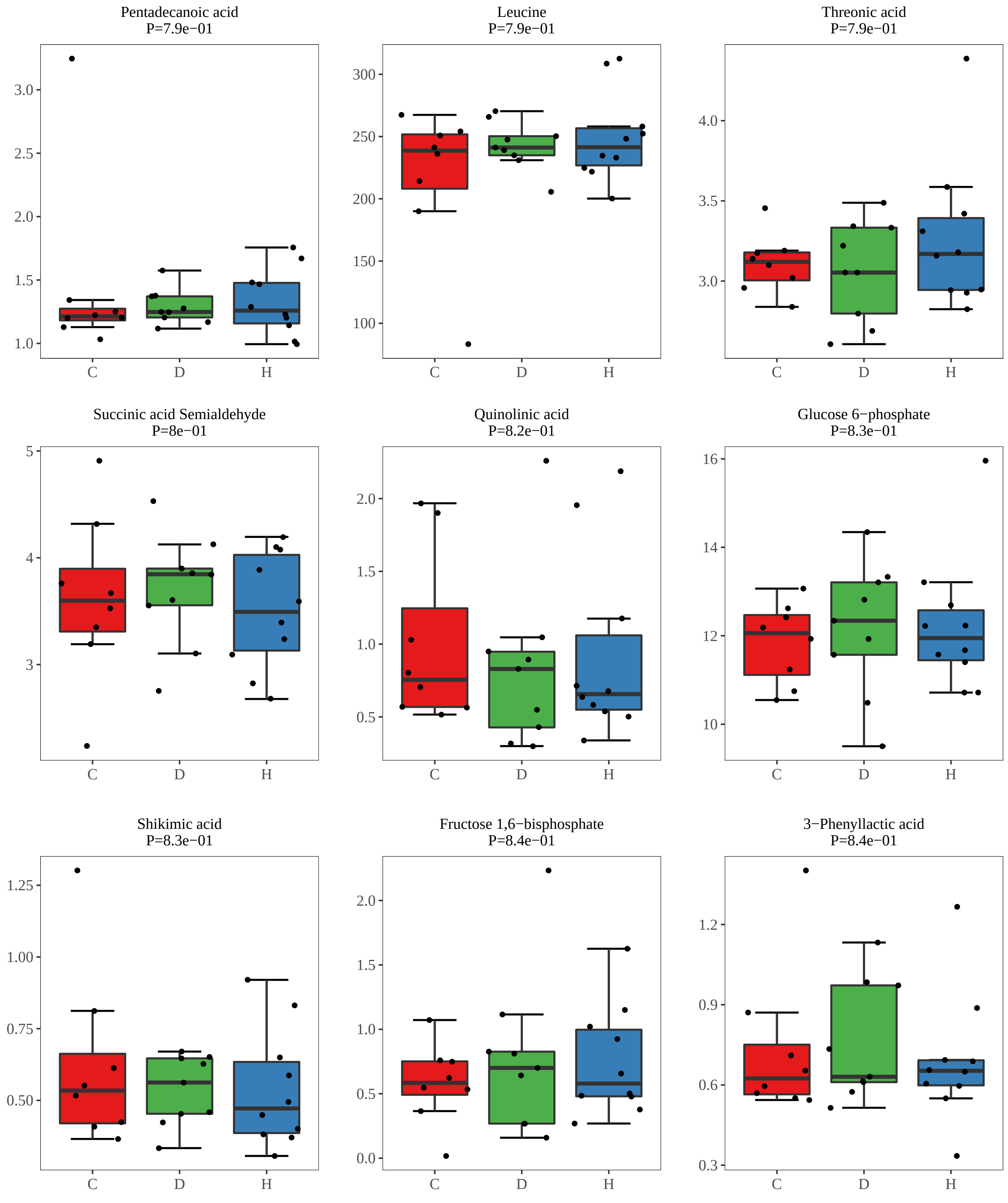
Pentadecanoic acid
P=7.9e−01
Leucine
P=7.9e−01
Threonic acid
P=7.9e−01
Succinic acid Semialdehyde
P=8e−01
Quinolinic acid
P=8.2e−01
Glucose 6−phosphate
P=8.3e−01
Shikimic acid
P=8.3e−01
Fructose 1,6−bisphosphate
P=8.4e−01
3−Phenyllactic acid
P=8.4e−01
C
D
H

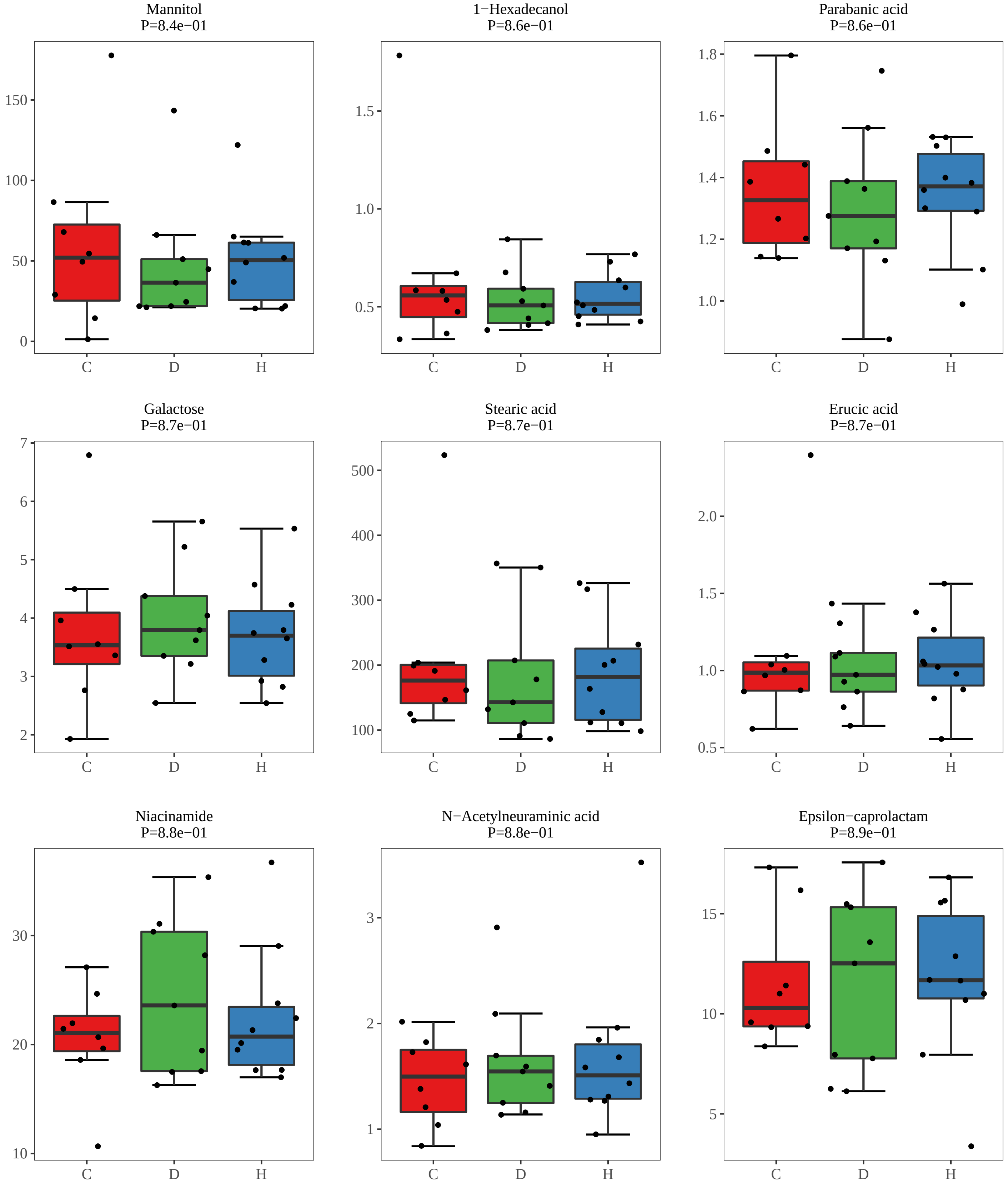

Mannitol
P=8.4e−01
1−Hexadecanol
P=8.6e−01
Parabanic acid
P=8.6e−01
Galactose
P=8.7e−01
Stearic acid
P=8.7e−01
Erucic acid
P=8.7e−01
Niacinamide
P=8.8e−01
N−Acetylneuraminic acid
P=8.8e−01
Epsilon−caprolactam
P=8.9e−01
C
D
H

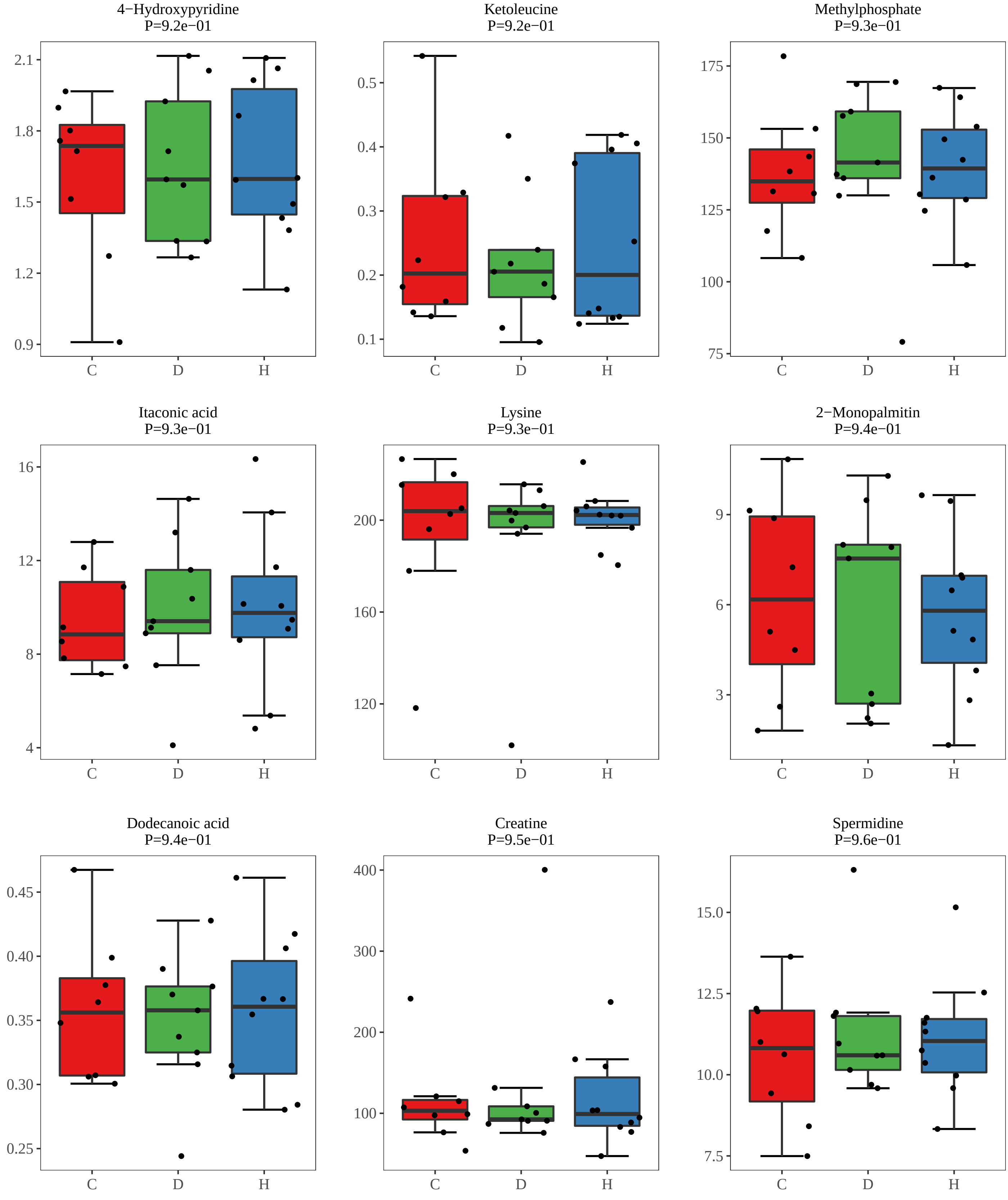
4−Hydroxypyridine
P=9.2e−01
Ketoleucine
P=9.2e−01
Methylphosphate
P=9.3e−01
Itaconic acid
P=9.3e−01
Lysine
P=9.3e−01
2−Monopalmitin
P=9.4e−01
Dodecanoic acid
P=9.4e−01
Creatine
P=9.5e−01
Spermidine
P=9.6e−01
0.9 1.2 1.5 1.8 2.1
0.1 0.2 0.3 0.4 0.5
75 100 125 150 175
4 8 12 16
120 160 200
3 6 9
0.25 0.30 0.35 0.40 0.45
100 200 300 400
7.5 10.0 12.5 15.0
C D H

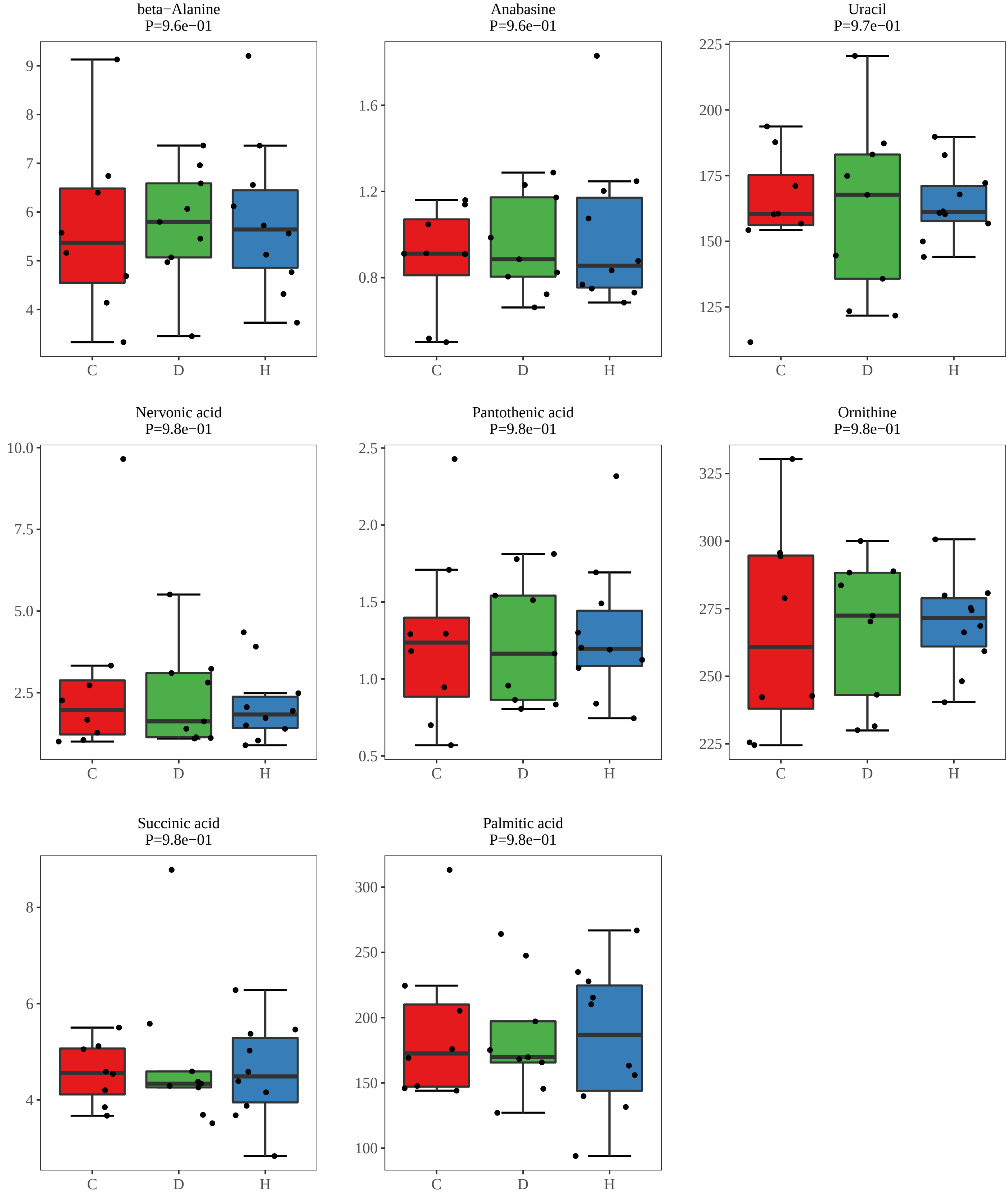
beta−Alanine
P=9.6e−01
Anabasine
P=9.6e−01
Uracil
P=9.7e−01
Nervonic acid
P=9.8e−01
Pantothenic acid
P=9.8e−01
Ornithine
P=9.8e−01
Succinic acid
P=9.8e−01
Palmitic acid
P=9.8e−01
C
D
H
