# Supplementary material for: Yiai Fuzheng decoction inhibits triple-negative breast cancer by remodeling the immune microenvironment
Source: Front Immunol. 2025 Sep 30;16:1615631. doi: 10.3389/fimmu.2025.1615631 (PMC12518410; doi:10.3389/fimmu.2025.1615631)
Supplement: Supplementary file 2 [file DataSheet1.zip › Supplementary File 2/Treatment/C_vs_D_vs_H/05_Potential_Biomarkers/Markers_Boxplot_with_Points.pdf]

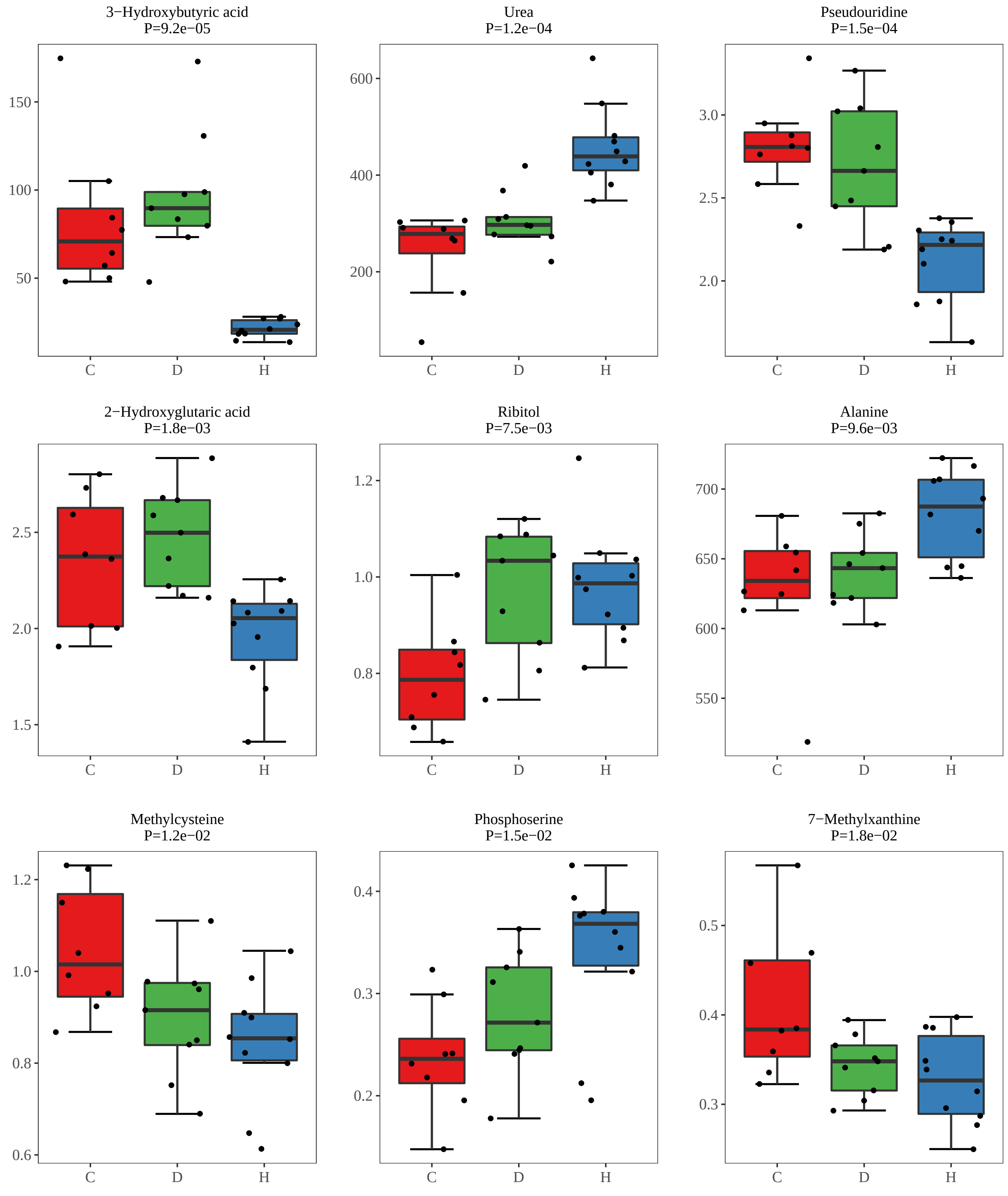

3−Hydroxybutyric acid
P=9.2e−05
Urea
P=1.2e−04
Pseudouridine
P=1.5e−04
2−Hydroxyglutaric acid
P=1.8e−03
Ribitol
P=7.5e−03
Alanine
P=9.6e−03
Methylcysteine
P=1.2e−02
Phosphoserine
P=1.5e−02
7−Methylxanthine
P=1.8e−02
C
D
H

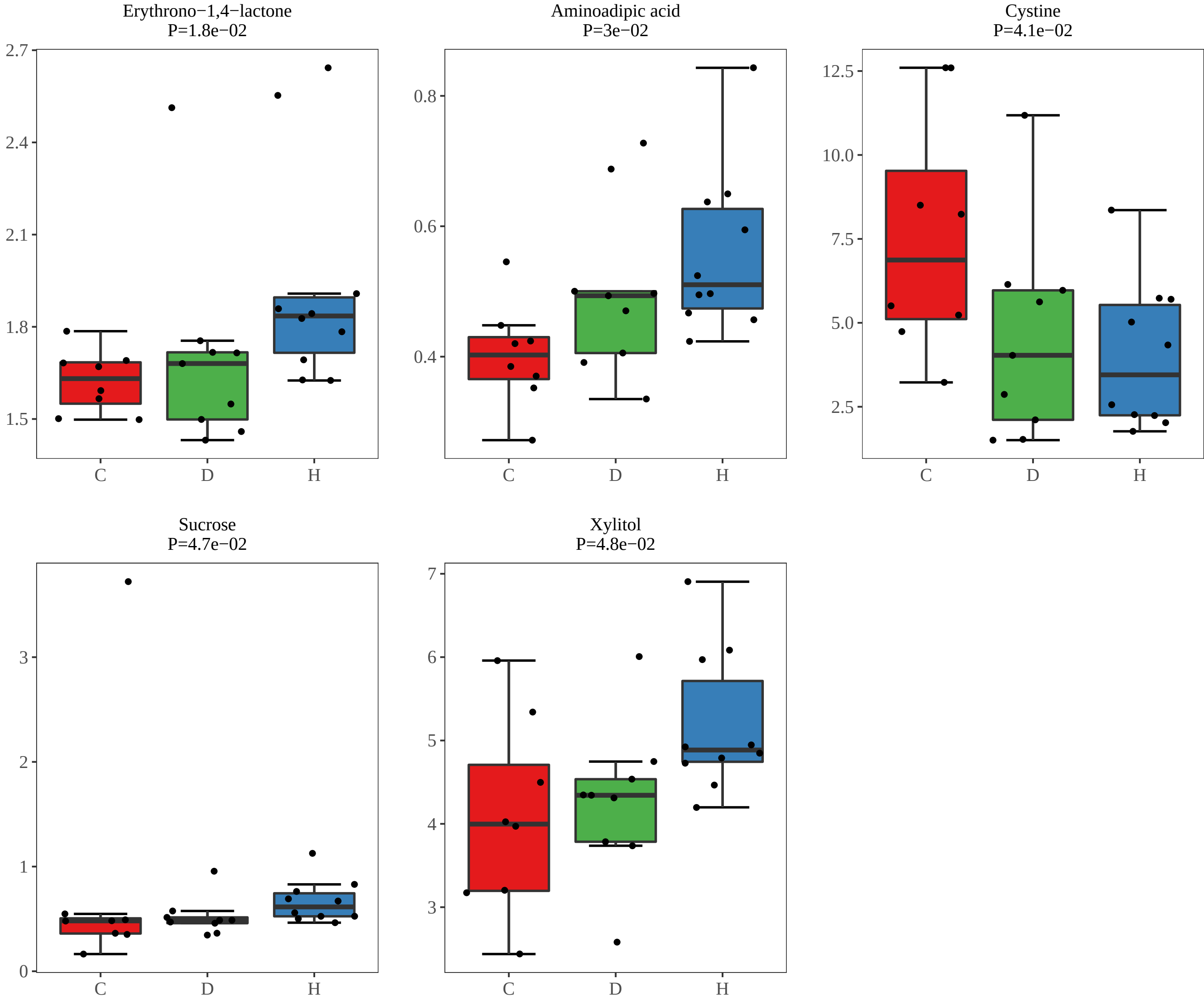
Erythrono−1,4−lactone
P=1.8e−02
2.7
2.4
2.1
1.8
1.5
C
D
H
Aminoadipic acid
P=3e−02
0.8
0.6
0.4
C
D
H
Cystine
P=4.1e−02
12.5
10.0
7.5
5.0
2.5
C
D
H
Sucrose
P=4.7e−02
3
2
1
0
C
D
H
Xylitol
P=4.8e−02
7
6
5
4
3
C
D
H
