# Supplementary material for: Yiai Fuzheng decoction inhibits triple-negative breast cancer by remodeling the immune microenvironment
Source: Front Immunol. 2025 Sep 30;16:1615631. doi: 10.3389/fimmu.2025.1615631 (PMC12518410; doi:10.3389/fimmu.2025.1615631)
Supplement: Supplementary file 2 [file DataSheet1.zip › Supplementary File 2/Treatment/C_vs_H/06_Potential_Biomarkers/Markers_Boxplot.pdf]

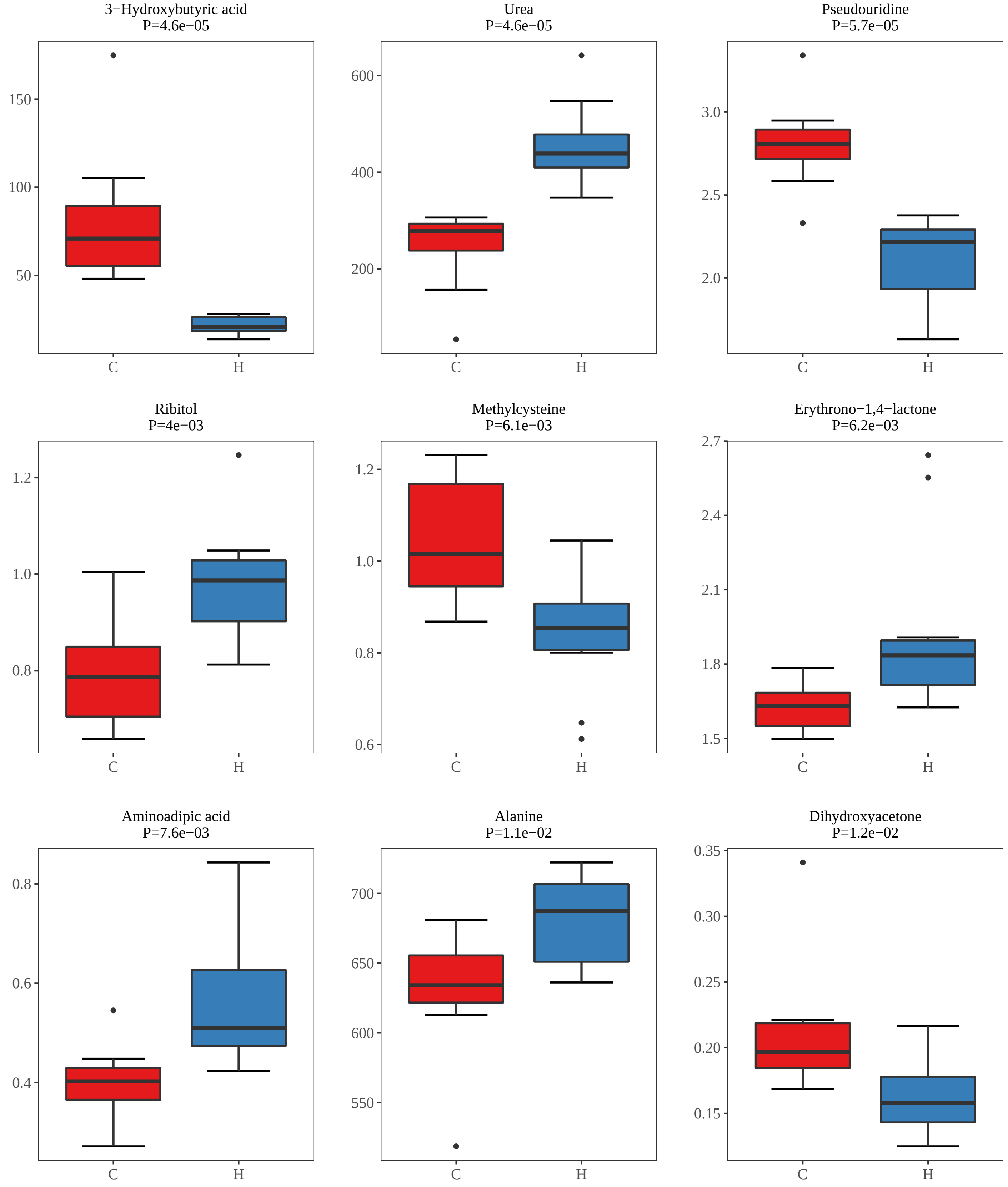
3−Hydroxybutyric acid
P=4.6e−05
150
100
50
C
H
Urea
P=4.6e−05
600
400
200
C
H
Pseudouridine
P=5.7e−05
3.0
2.5
2.0
C
H
Ribitol
P=4e−03
1.2
1.0
0.8
C
H
Methylcysteine
P=6.1e−03
1.2
1.0
0.8
0.6
C
H
Erythrono−1,4−lactone
P=6.2e−03
2.7
2.4
2.1
1.8
1.5
C
H
Aminoadipic acid
P=7.6e−03
0.8
0.6
0.4
C
H
Alanine
P=1.1e−02
700
650
600
550
C
H
Dihydroxyacetone
P=1.2e−02
0.35
0.30
0.25
0.20
0.15
C
H

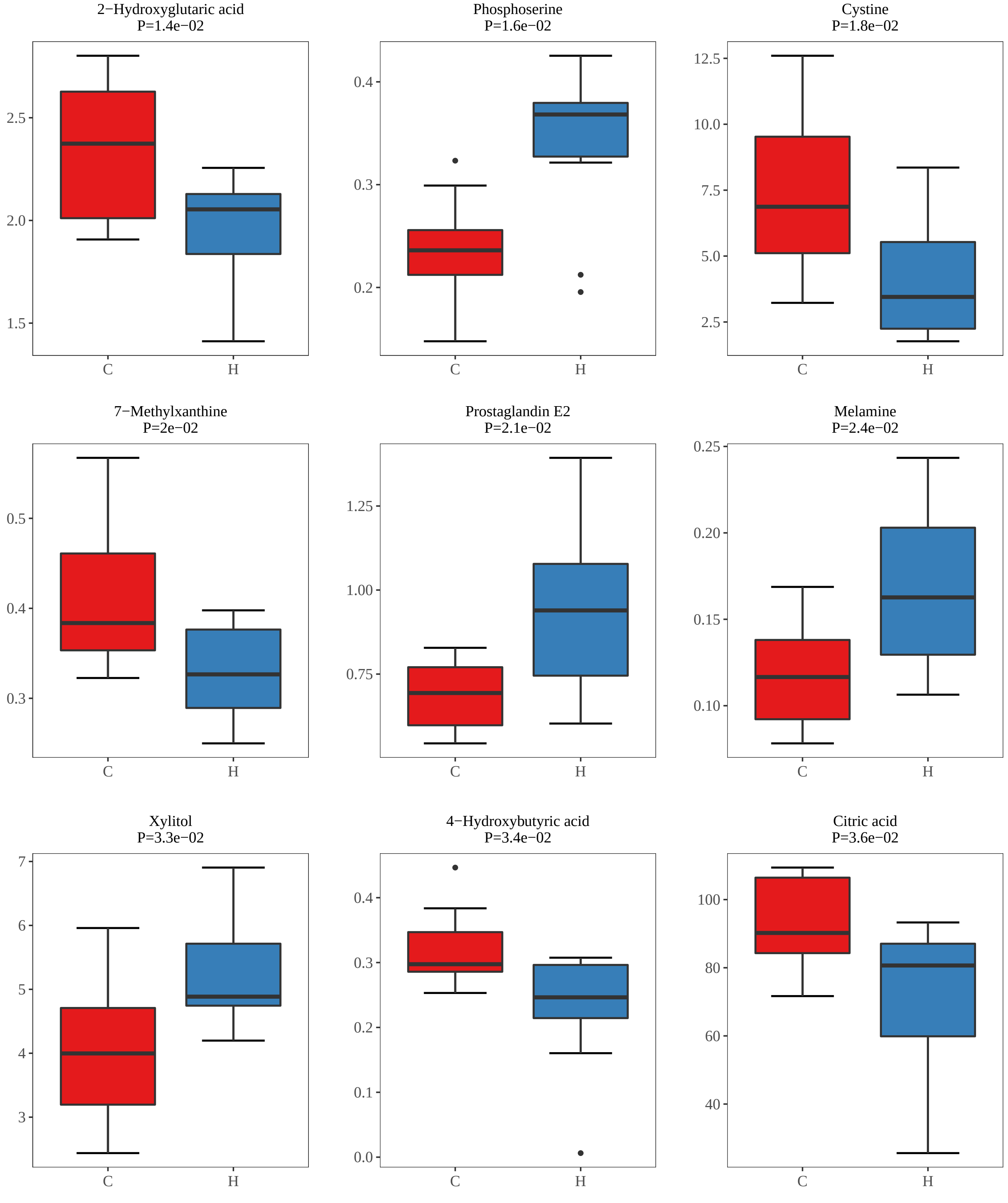
2−Hydroxyglutaric acid
P=1.4e−02
Phosphoserine
P=1.6e−02
Cystine
P=1.8e−02
7−Methylxanthine
P=2e−02
Prostaglandin E2
P=2.1e−02
Melamine
P=2.4e−02
Xylitol
P=3.3e−02
4−Hydroxybutyric acid
P=3.4e−02
Citric acid
P=3.6e−02
C
H
