# Supplementary material for: Yiai Fuzheng decoction inhibits triple-negative breast cancer by remodeling the immune microenvironment
Source: Front Immunol. 2025 Sep 30;16:1615631. doi: 10.3389/fimmu.2025.1615631 (PMC12518410; doi:10.3389/fimmu.2025.1615631)
Supplement: Supplementary file 2 [file DataSheet1.zip › Supplementary File 2/Treatment/C_vs_H/05_Univariate_Analysis/AllMet_Boxplot_with_Points.pdf]

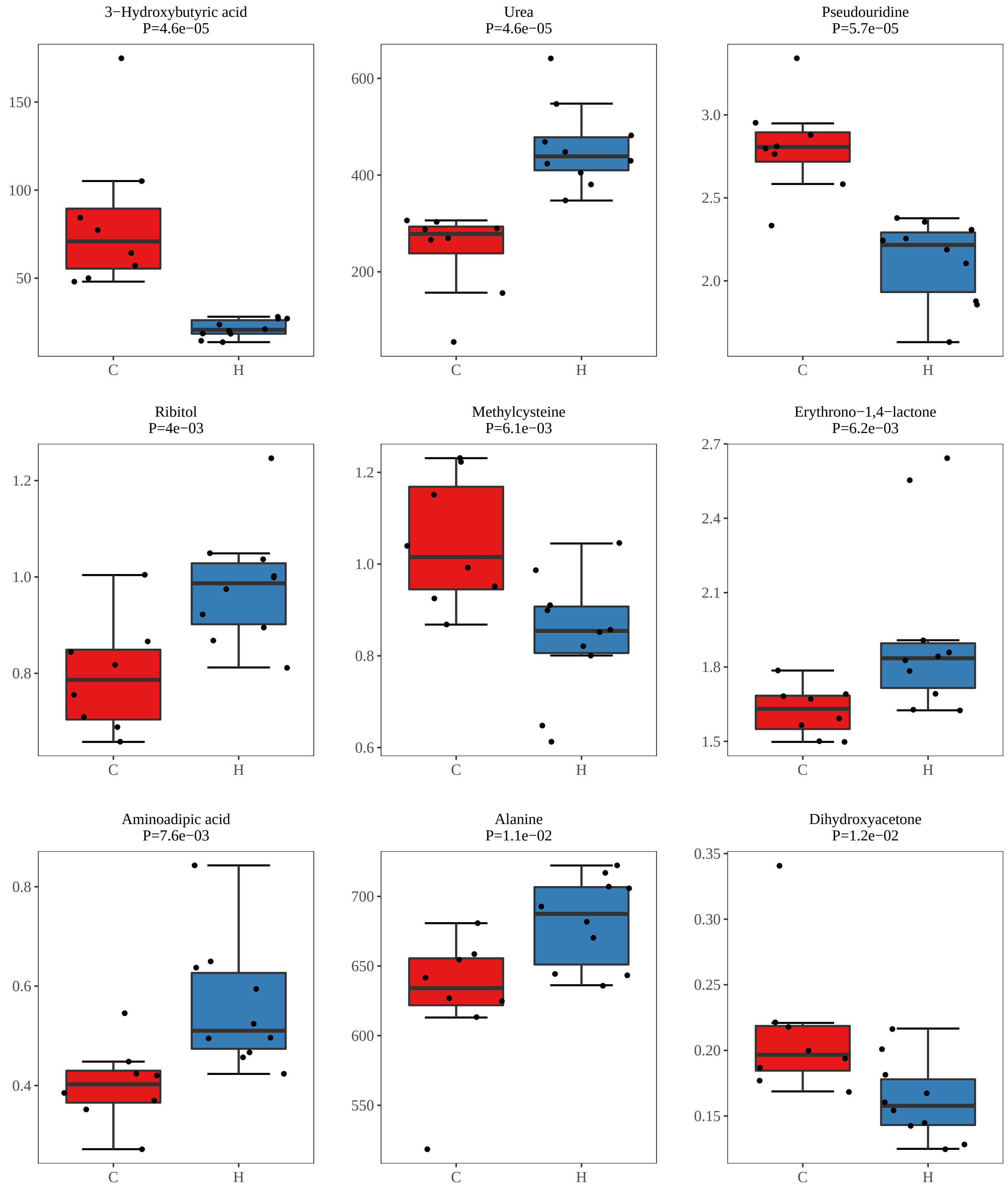
3−Hydroxybutyric acid
P=4.6e−05
150
100
50
C
H
Urea
P=4.6e−05
600
400
200
C
H
Pseudouridine
P=5.7e−05
3.0
2.5
2.0
C
H
Ribitol
P=4e−03
1.2
1.0
0.8
C
H
Methylcysteine
P=6.1e−03
1.2
1.0
0.8
0.6
C
H
Erythrono−1,4−lactone
P=6.2e−03
2.7
2.4
2.1
1.8
1.5
C
H
Aminoadipic acid
P=7.6e−03
0.8
0.6
0.4
C
H
Alanine
P=1.1e−02
700
650
600
550
C
H
Dihydroxyacetone
P=1.2e−02
0.35
0.30
0.25
0.20
0.15
C
H

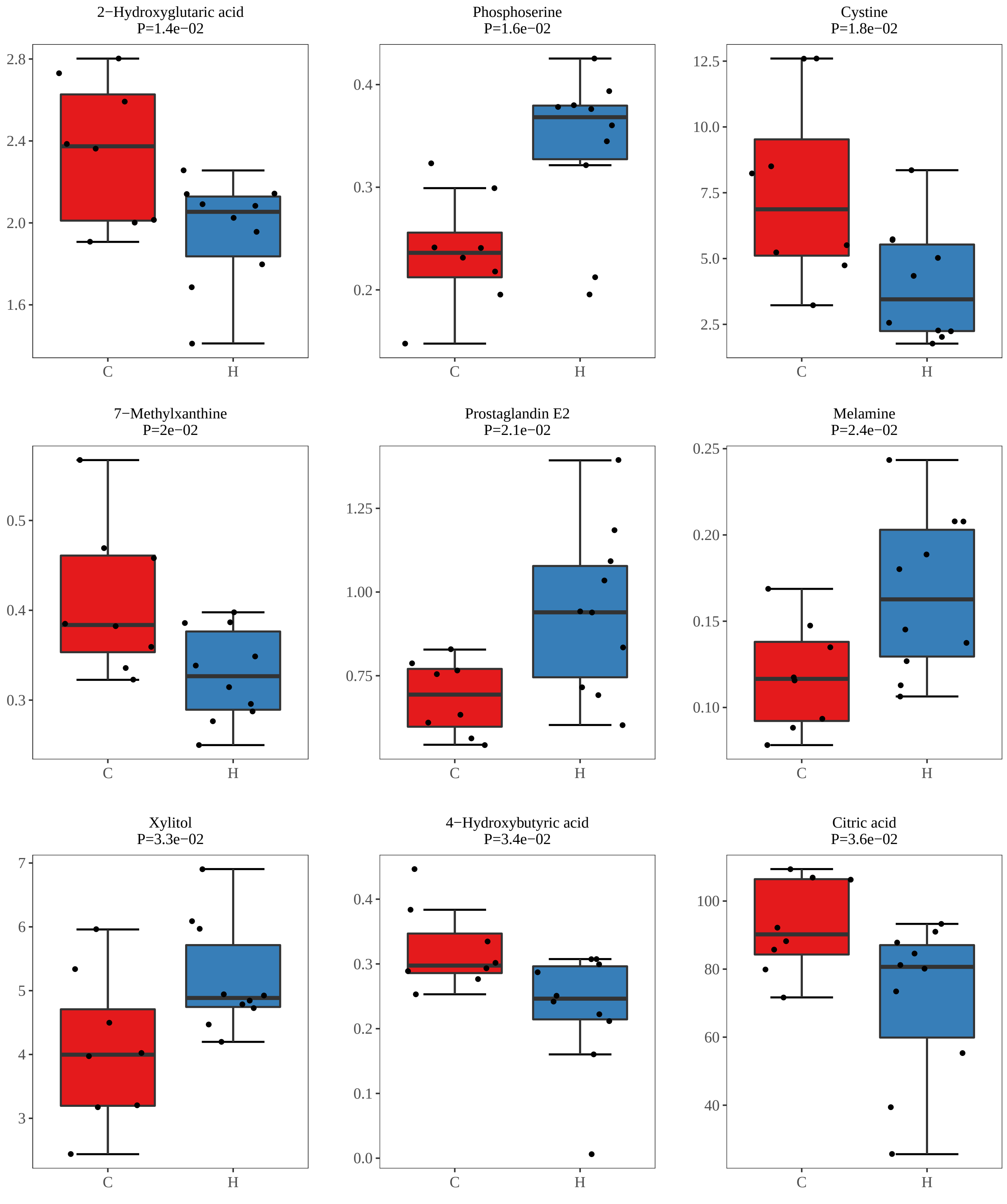
2−Hydroxyglutaric acid
P=1.4e−02
2.8
2.4
2.0
1.6
C
H
Phosphoserine
P=1.6e−02
0.4
0.3
0.2
C
H
Cystine
P=1.8e−02
12.5
10.0
7.5
5.0
2.5
C
H
7−Methylxanthine
P=2e−02
0.5
0.4
0.3
C
H
Prostaglandin E2
P=2.1e−02
1.25
1.00
0.75
C
H
Melamine
P=2.4e−02
0.25
0.20
0.15
0.10
C
H
Xylitol
P=3.3e−02
7
6
5
4
3
C
H
4−Hydroxybutyric acid
P=3.4e−02
0.4
0.3
0.2
0.1
0.0
C
H
Citric acid
P=3.6e−02
100
80
60
40
C
H

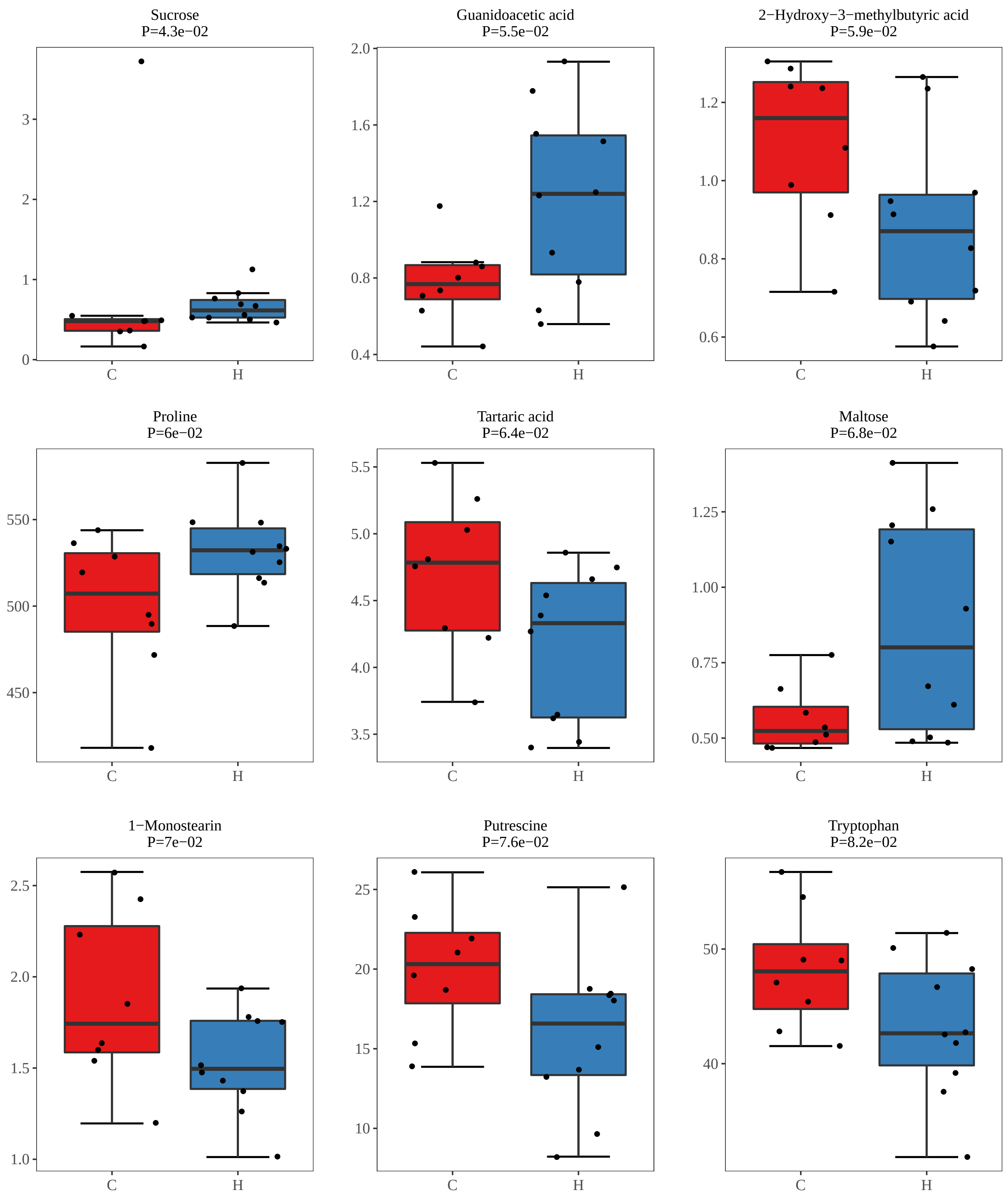

Sucrose
P=4.3e−02
Guanidoacetic acid
P=5.5e−02
2−Hydroxy−3−methylbutyric acid
P=5.9e−02
Proline
P=6e−02
Tartaric acid
P=6.4e−02
Maltose
P=6.8e−02
1−Monostearin
P=7e−02
Putrescine
P=7.6e−02
Tryptophan
P=8.2e−02
C
H

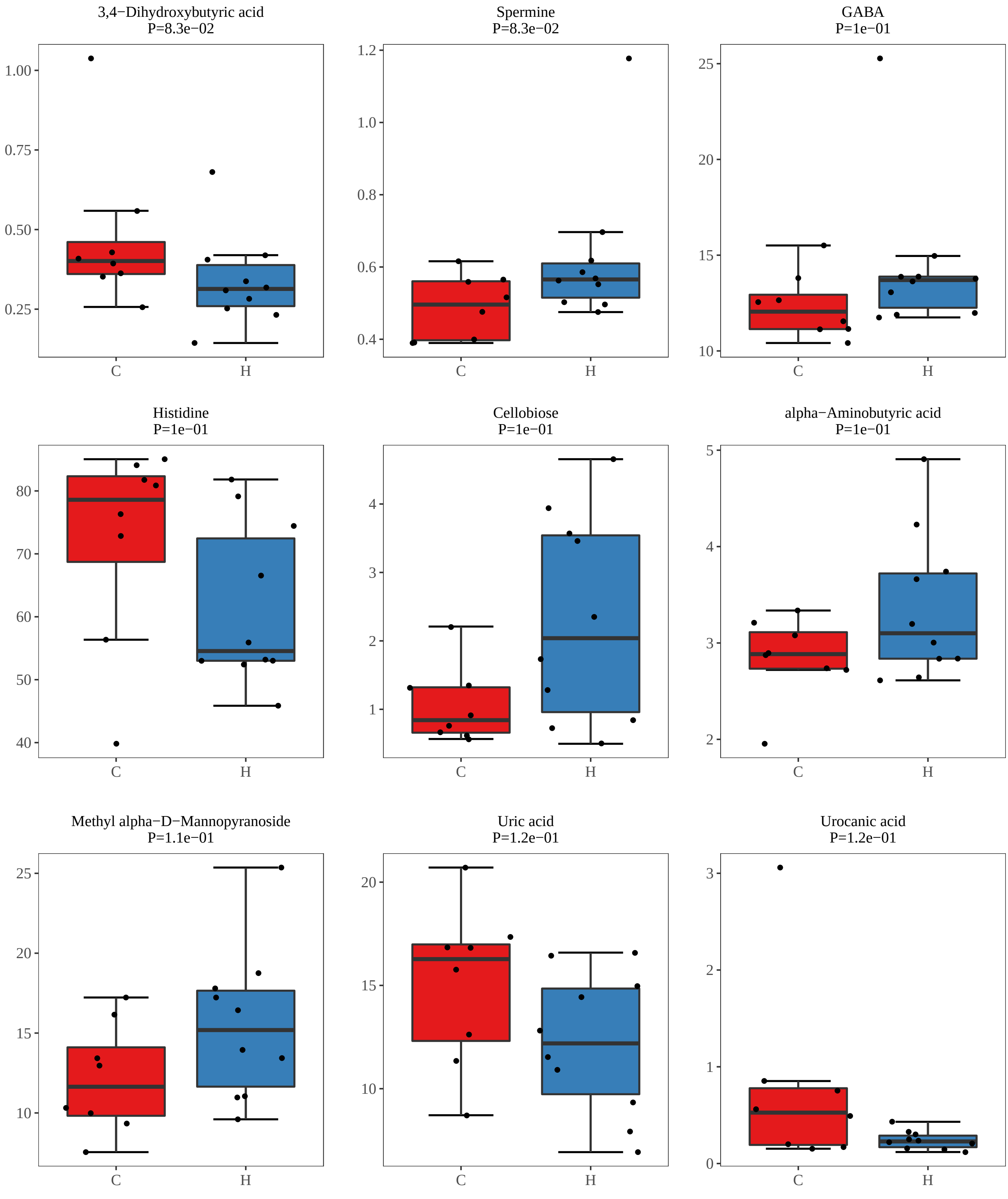

3,4−Dihydroxybutyric acid
P=8.3e−02
1.00
0.75
0.50
0.25
C
H
Spermine
P=8.3e−02
1.2
1.0
0.8
0.6
0.4
C
H
GABA
P=1e−01
25
20
15
10
C
H
Histidine
P=1e−01
80
70
60
50
40
C
H
Cellobiose
P=1e−01
4
3
2
1
C
H
alpha−Aminobutyric acid
P=1e−01
5
4
3
2
C
H
Methyl alpha−D−Mannopyranoside
P=1.1e−01
25
20
15
10
C
H
Uric acid
P=1.2e−01
20
15
10
C
H
Urocanic acid
P=1.2e−01
3
2
1
0
C
H

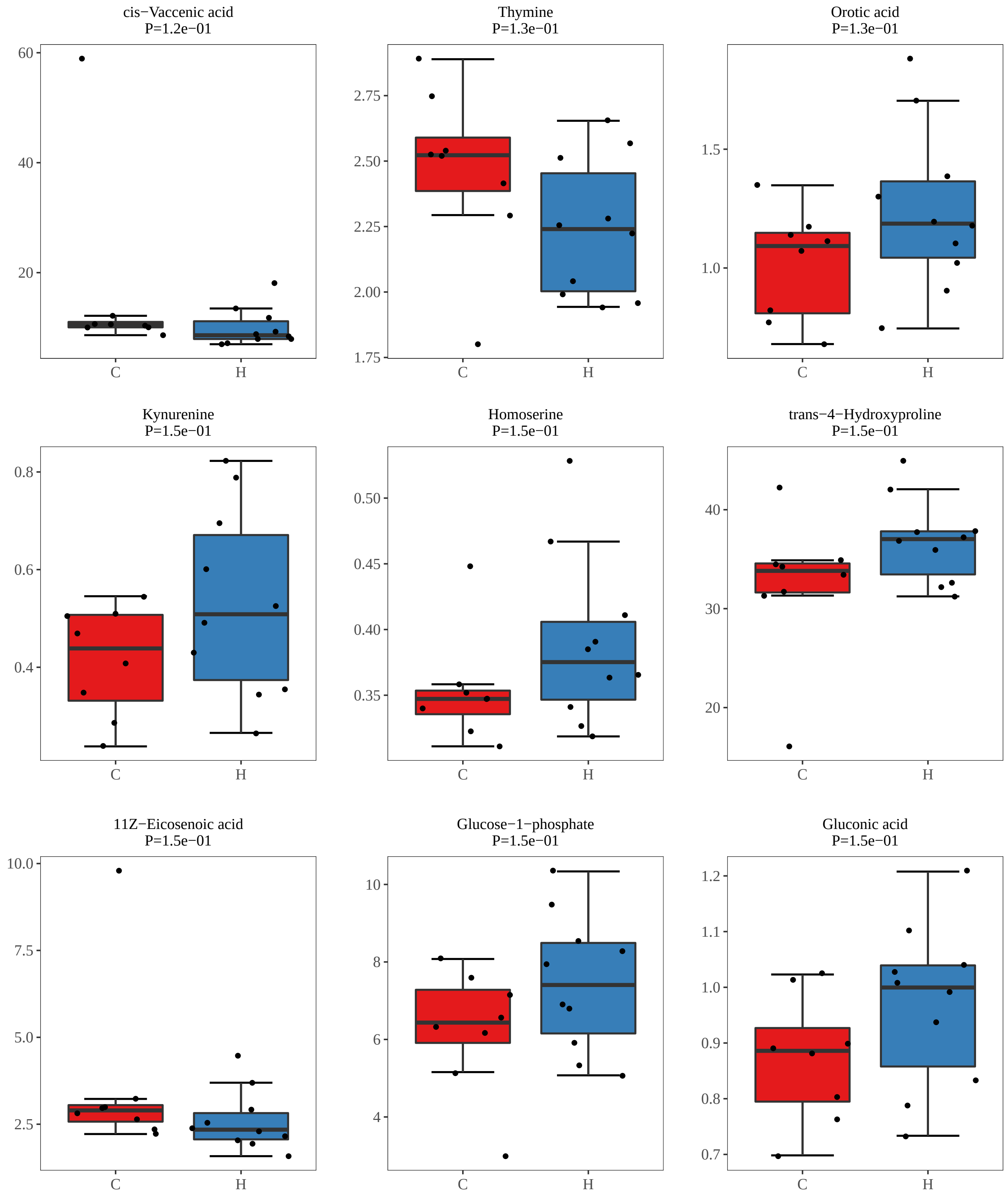

cis−Vaccenic acid
P=1.2e−01
Thymine
P=1.3e−01
Orotic acid
P=1.3e−01
Kynurenine
P=1.5e−01
Homoserine
P=1.5e−01
trans−4−Hydroxyproline
P=1.5e−01
11Z−Eicosenoic acid
P=1.5e−01
Glucose−1−phosphate
P=1.5e−01
Gluconic acid
P=1.5e−01
C
H

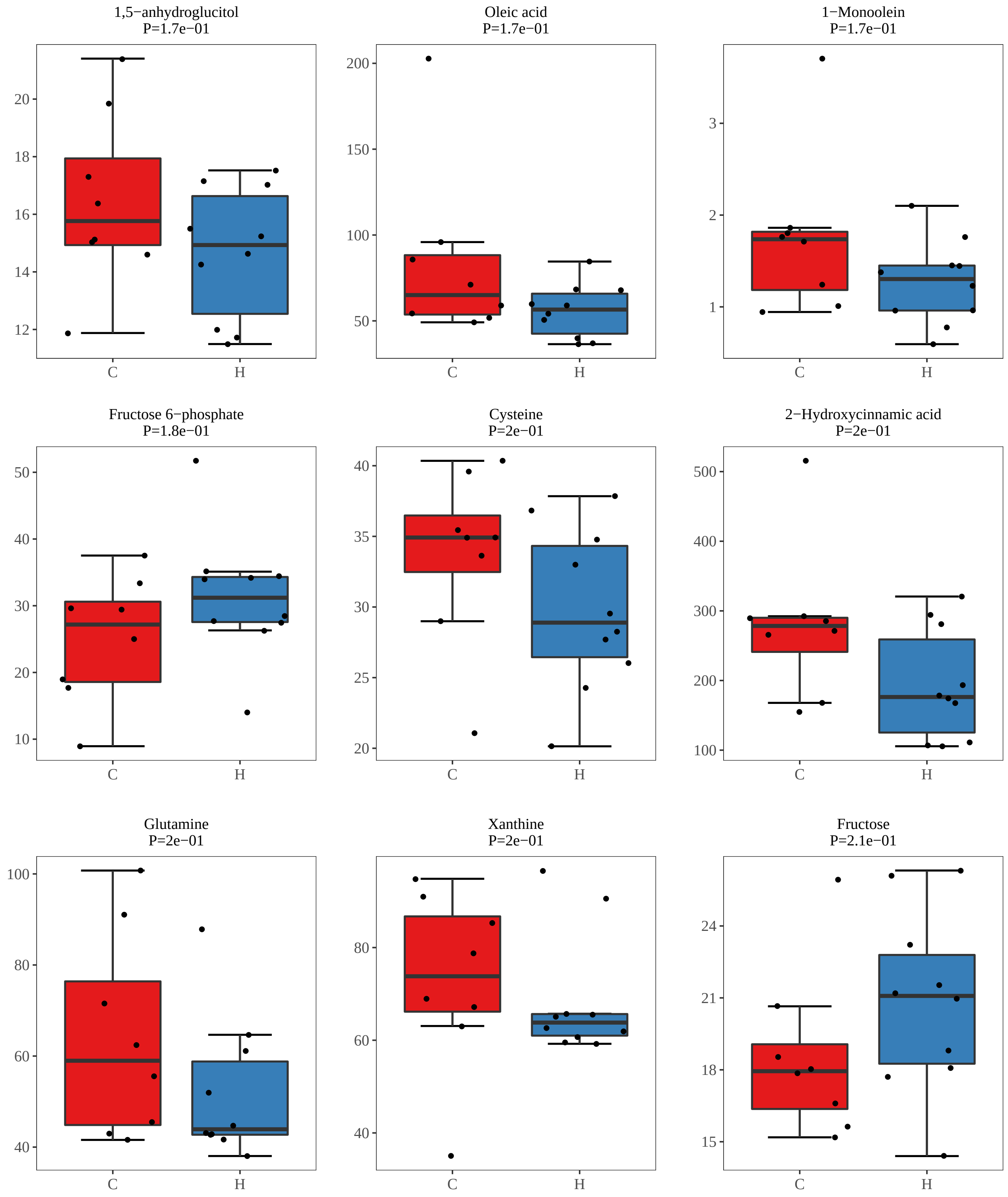

1,5−anhydroglucitol
P=1.7e−01
Oleic acid
P=1.7e−01
1−Monoolein
P=1.7e−01
Fructose 6−phosphate
P=1.8e−01
Cysteine
P=2e−01
2−Hydroxycinnamic acid
P=2e−01
Glutamine
P=2e−01
Xanthine
P=2e−01
Fructose
P=2.1e−01
C
H

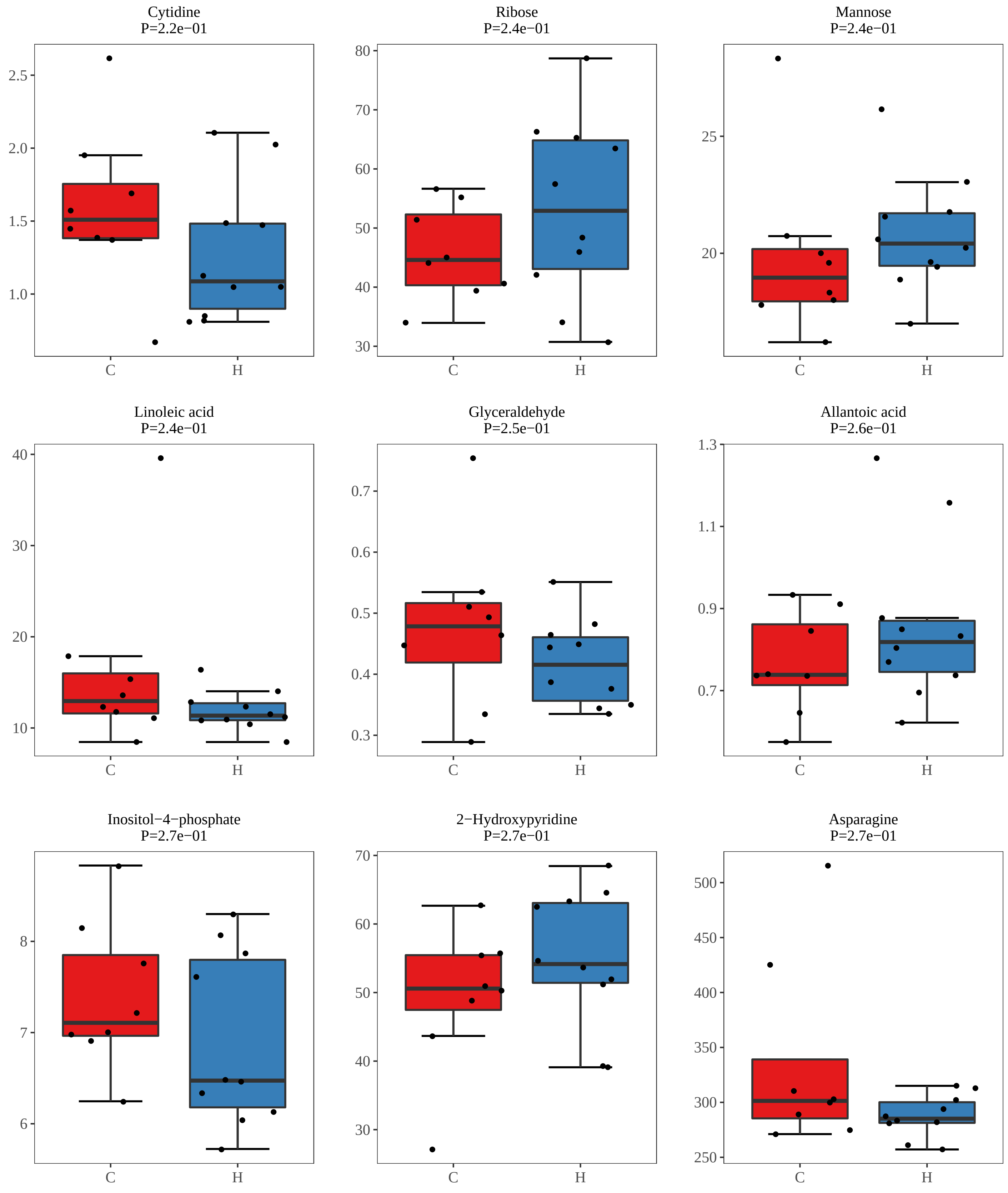
Cytidine
P=2.2e−01
Ribose
P=2.4e−01
Mannose
P=2.4e−01
Linoleic acid
P=2.4e−01
Glyceraldehyde
P=2.5e−01
Allantoic acid
P=2.6e−01
Inositol−4−phosphate
P=2.7e−01
2−Hydroxypyridine
P=2.7e−01
Asparagine
P=2.7e−01
C
H

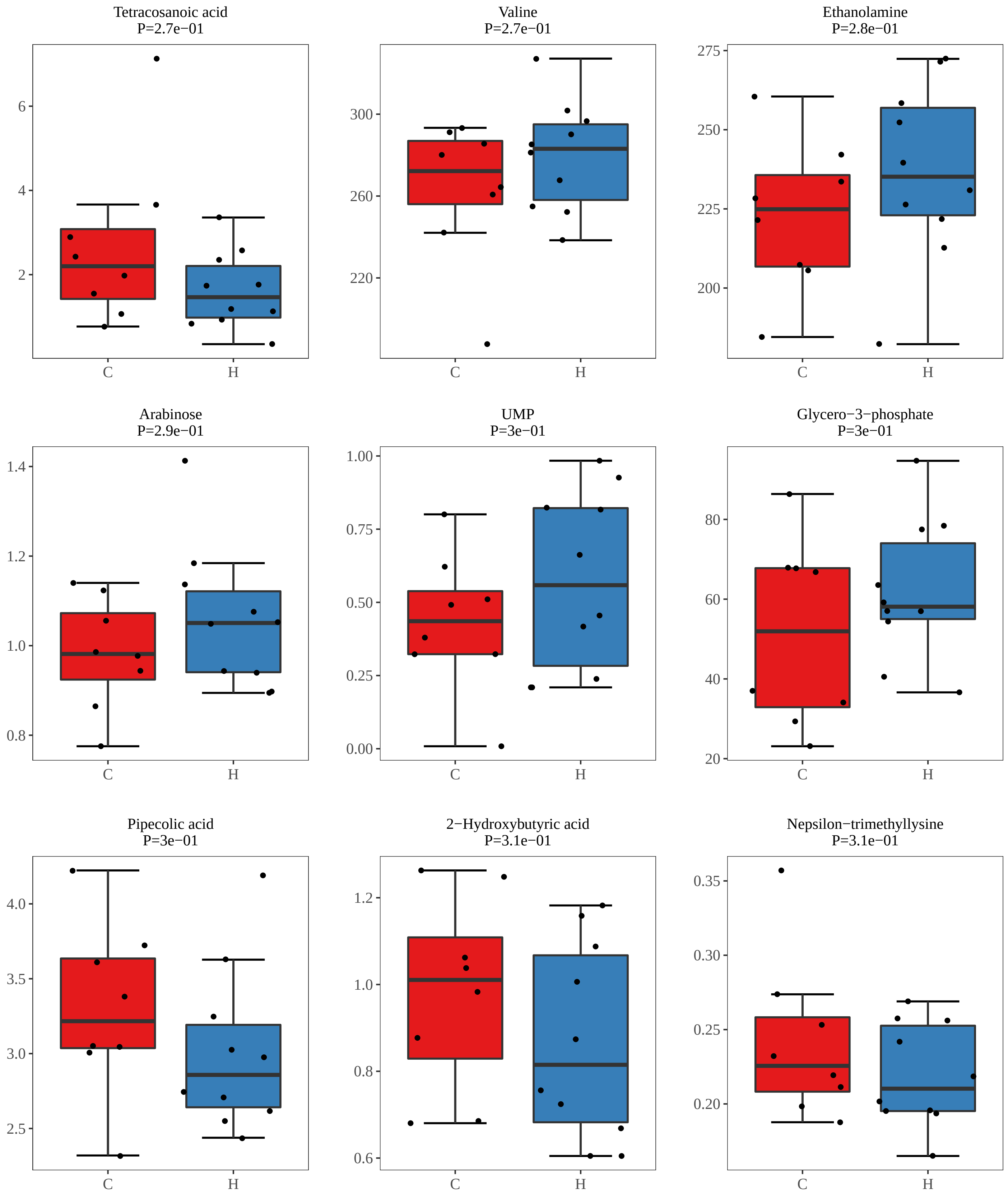

Tetracosanoic acid
P=2.7e−01
2
4
6
C
H
Valine
P=2.7e−01
220
260
300
C
H
Ethanolamine
P=2.8e−01
200
225
250
275
C
H
Arabinose
P=2.9e−01
0.8
1.0
1.2
1.4
C
H
UMP
P=3e−01
0.00
0.25
0.50
0.75
1.00
C
H
Glycero−3−phosphate
P=3e−01
20
40
60
80
C
H
Pipecolic acid
P=3e−01
2.5
3.0
3.5
4.0
C
H
2−Hydroxybutyric acid
P=3.1e−01
0.6
0.8
1.0
1.2
C
H
Nepsilon−trimethyllysine
P=3.1e−01
0.20
0.25
0.30
0.35
C
H

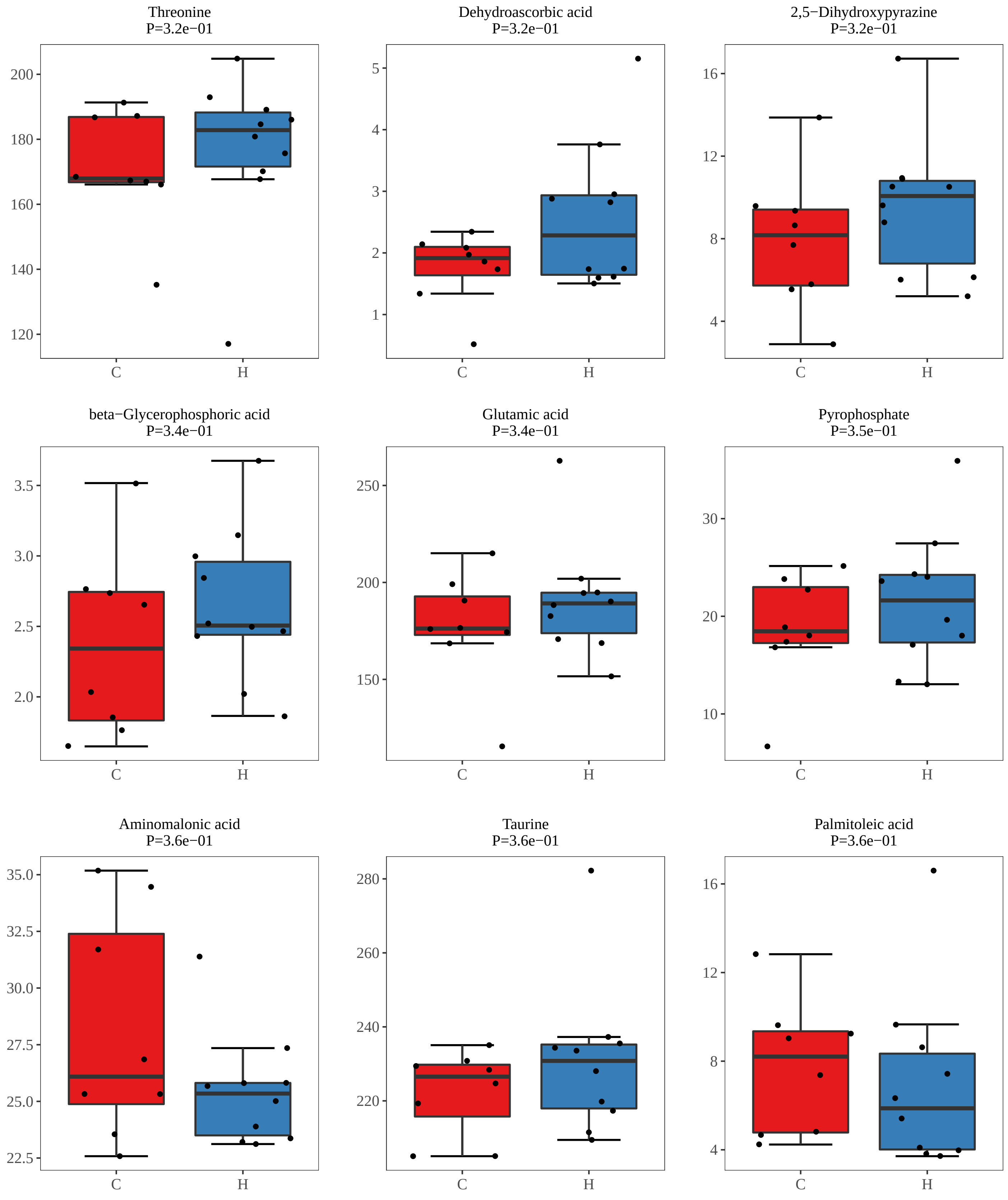
Threonine
P=3.2e−01
Dehydroascorbic acid
P=3.2e−01
2,5−Dihydroxypyrazine
P=3.2e−01
beta−Glycerophosphoric acid
P=3.4e−01
Glutamic acid
P=3.4e−01
Pyrophosphate
P=3.5e−01
Aminomalonic acid
P=3.6e−01
Taurine
P=3.6e−01
Palmitoleic acid
P=3.6e−01
C
H

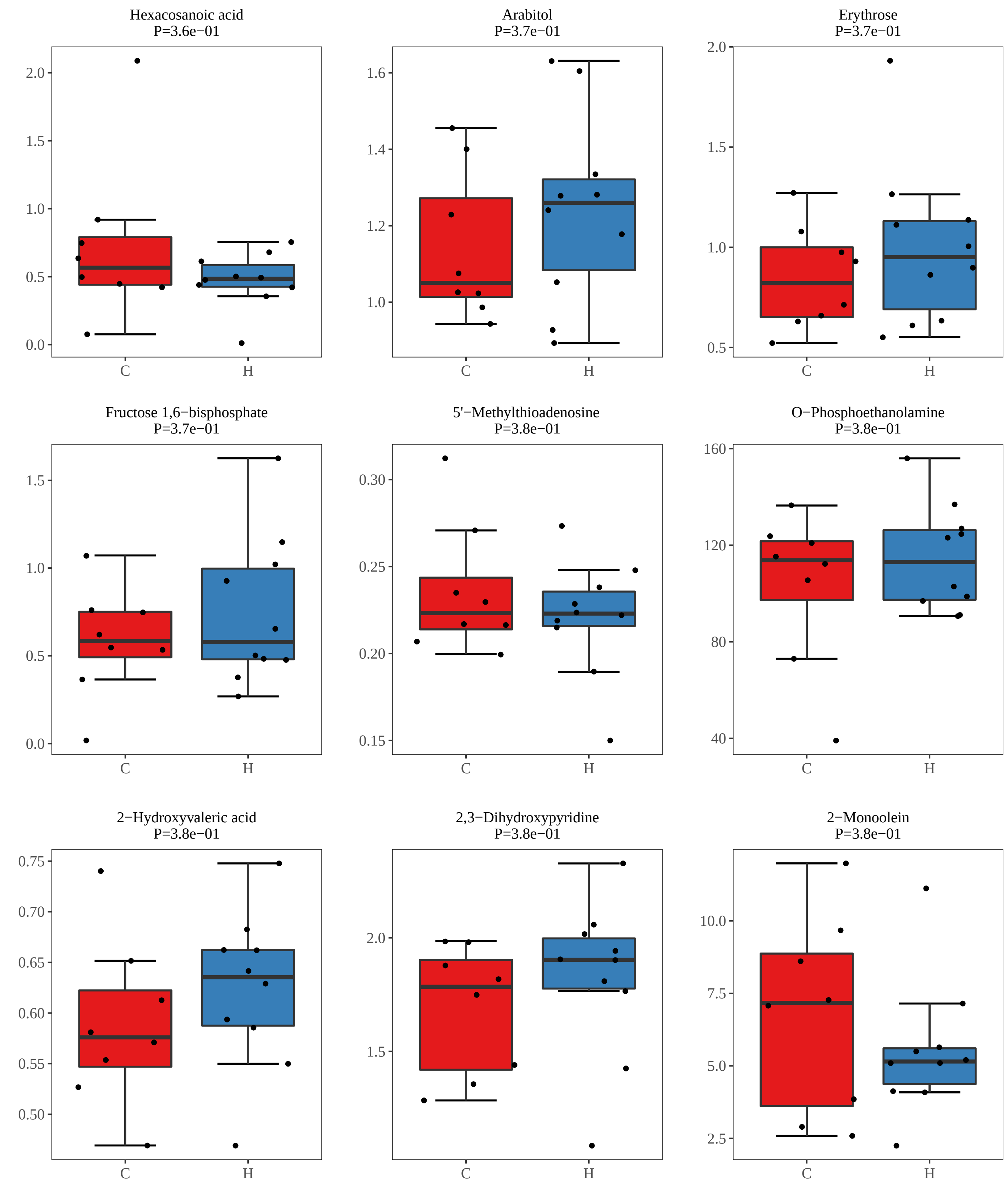
Hexacosanoic acid
P=3.6e−01
0.0
0.5
1.0
1.5
2.0
C
H
Arabitol
P=3.7e−01
1.0
1.2
1.4
1.6
C
H
Erythrose
P=3.7e−01
0.5
1.0
1.5
2.0
C
H
Fructose 1,6−bisphosphate
P=3.7e−01
0.0
0.5
1.0
1.5
C
H
5'−Methylthioadenosine
P=3.8e−01
0.15
0.20
0.25
0.30
C
H
O−Phosphoethanolamine
P=3.8e−01
40
80
120
160
C
H
2−Hydroxyvaleric acid
P=3.8e−01
0.50
0.55
0.60
0.65
0.70
0.75
C
H
2,3−Dihydroxypyridine
P=3.8e−01
1.5
2.0
C
H
2−Monoolein
P=3.8e−01
2.5
5.0
7.5
10.0
C
H

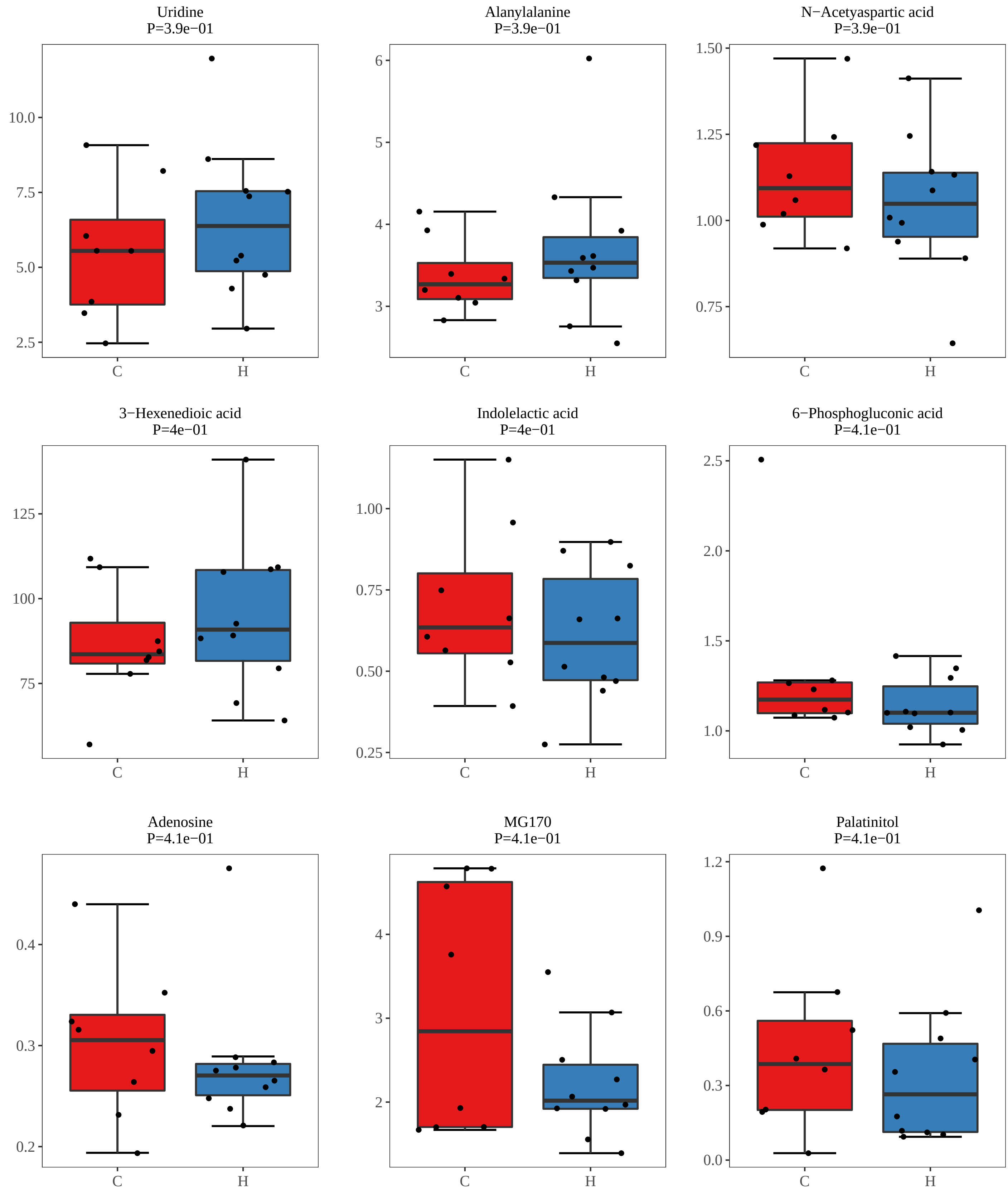
Uridine
P=3.9e−01
10.0
7.5
5.0
2.5
C
H
Alanylalanine
P=3.9e−01
6
5
4
3
C
H
N−Acetyaspartic acid
P=3.9e−01
1.50
1.25
1.00
0.75
C
H
3−Hexenedioic acid
P=4e−01
125
100
75
C
H
Indolelactic acid
P=4e−01
1.00
0.75
0.50
0.25
C
H
6−Phosphogluconic acid
P=4.1e−01
2.5
2.0
1.5
1.0
C
H
Adenosine
P=4.1e−01
0.4
0.3
0.2
C
H
MG170
P=4.1e−01
4
3
2
C
H
Palatinitol
P=4.1e−01
1.2
0.9
0.6
0.3
0.0
C
H

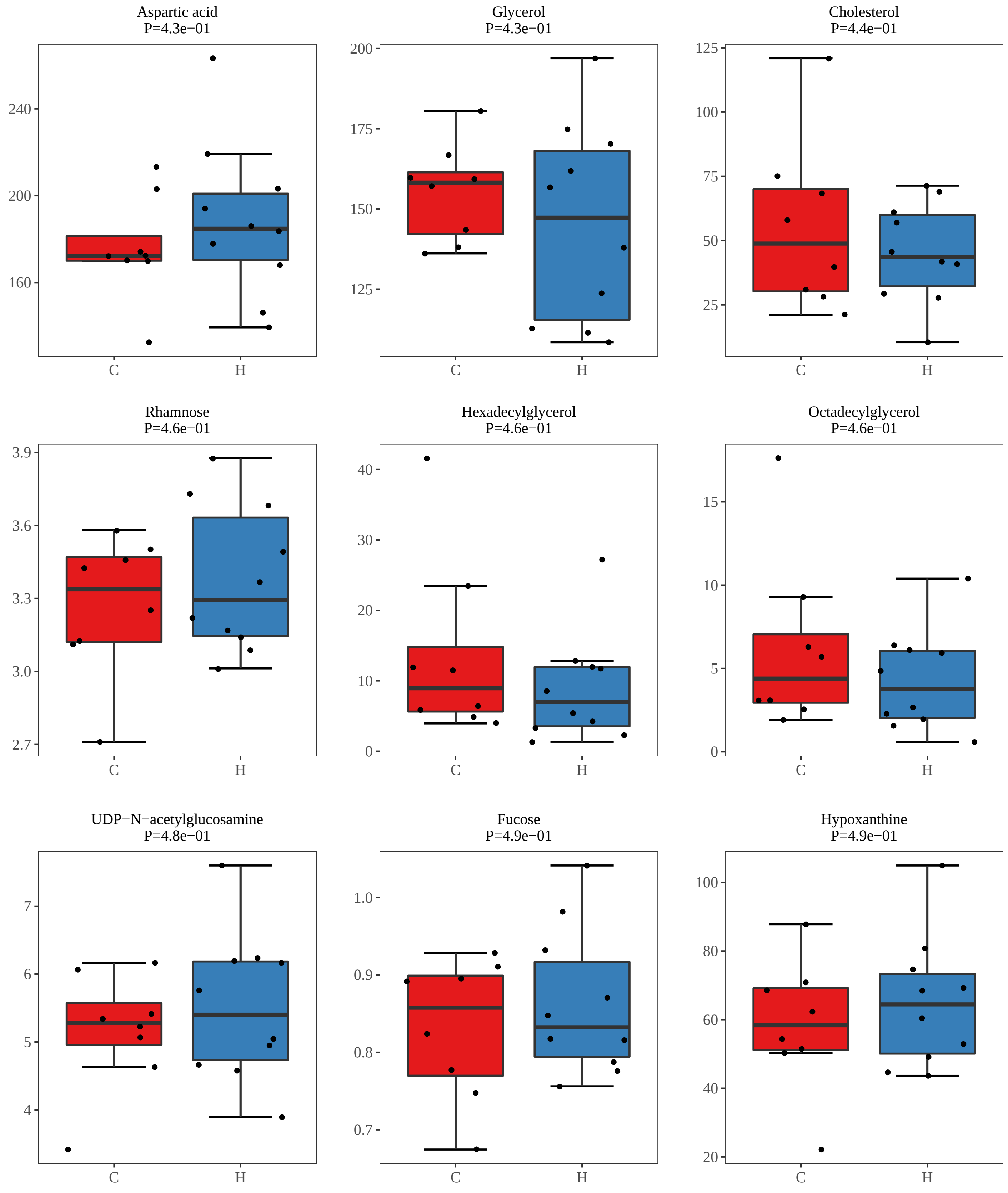
Aspartic acid
P=4.3e−01
Glycerol
P=4.3e−01
Cholesterol
P=4.4e−01
Rhamnose
P=4.6e−01
Hexadecylglycerol
P=4.6e−01
Octadecylglycerol
P=4.6e−01
UDP−N−acetylglucosamine
P=4.8e−01
Fucose
P=4.9e−01
Hypoxanthine
P=4.9e−01
C
H

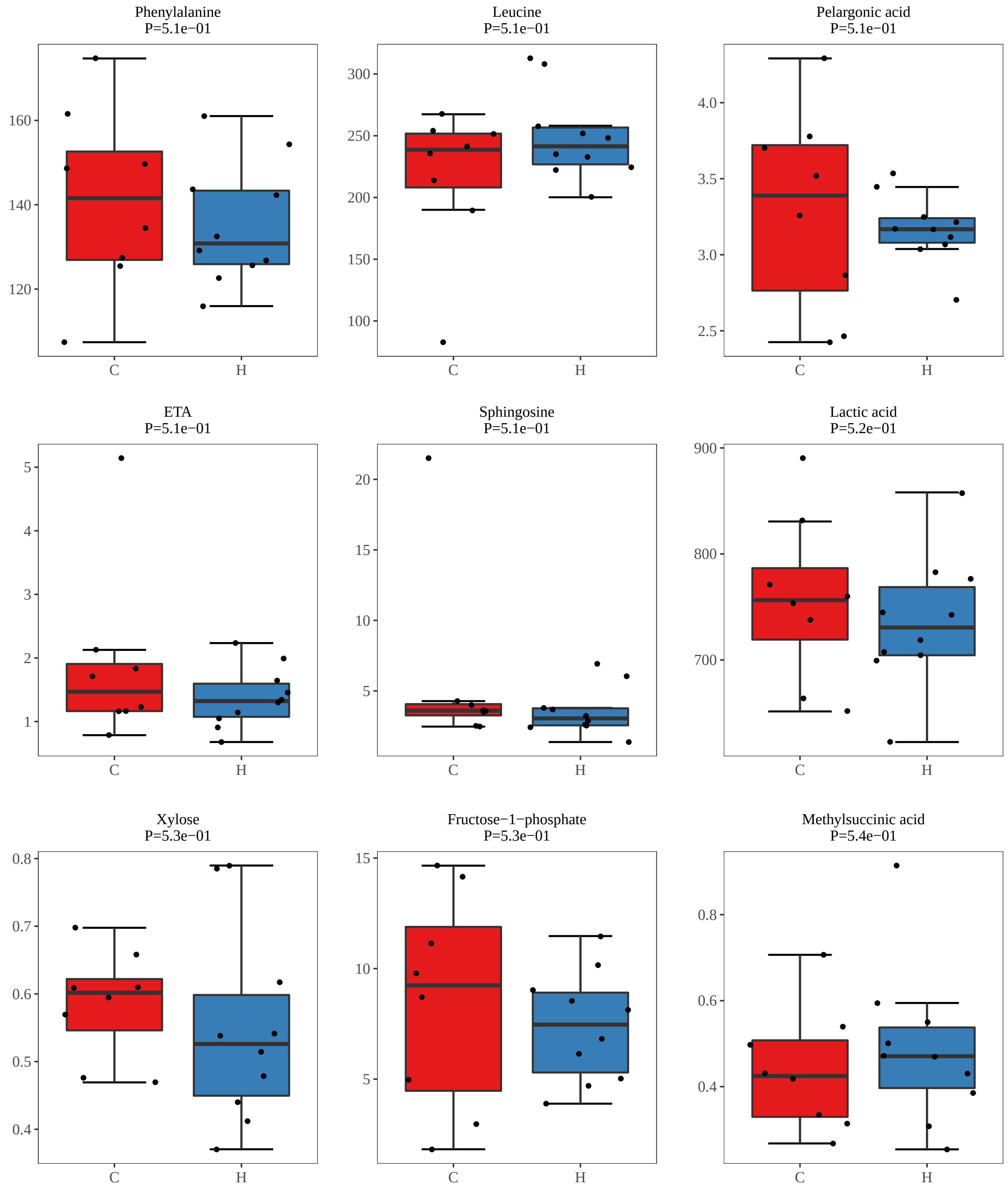
Phenylalanine
P=5.1e−01
120
140
160
C
H
Leucine
P=5.1e−01
100
150
200
250
300
C
H
Pelargonic acid
P=5.1e−01
2.5
3.0
3.5
4.0
C
H
ETA
P=5.1e−01
1
2
3
4
5
C
H
Sphingosine
P=5.1e−01
5
10
15
20
C
H
Lactic acid
P=5.2e−01
700
800
900
C
H
Xylose
P=5.3e−01
0.4
0.5
0.6
0.7
0.8
C
H
Fructose−1−phosphate
P=5.3e−01
5
10
15
C
H
Methylsuccinic acid
P=5.4e−01
0.4
0.6
0.8
C
H

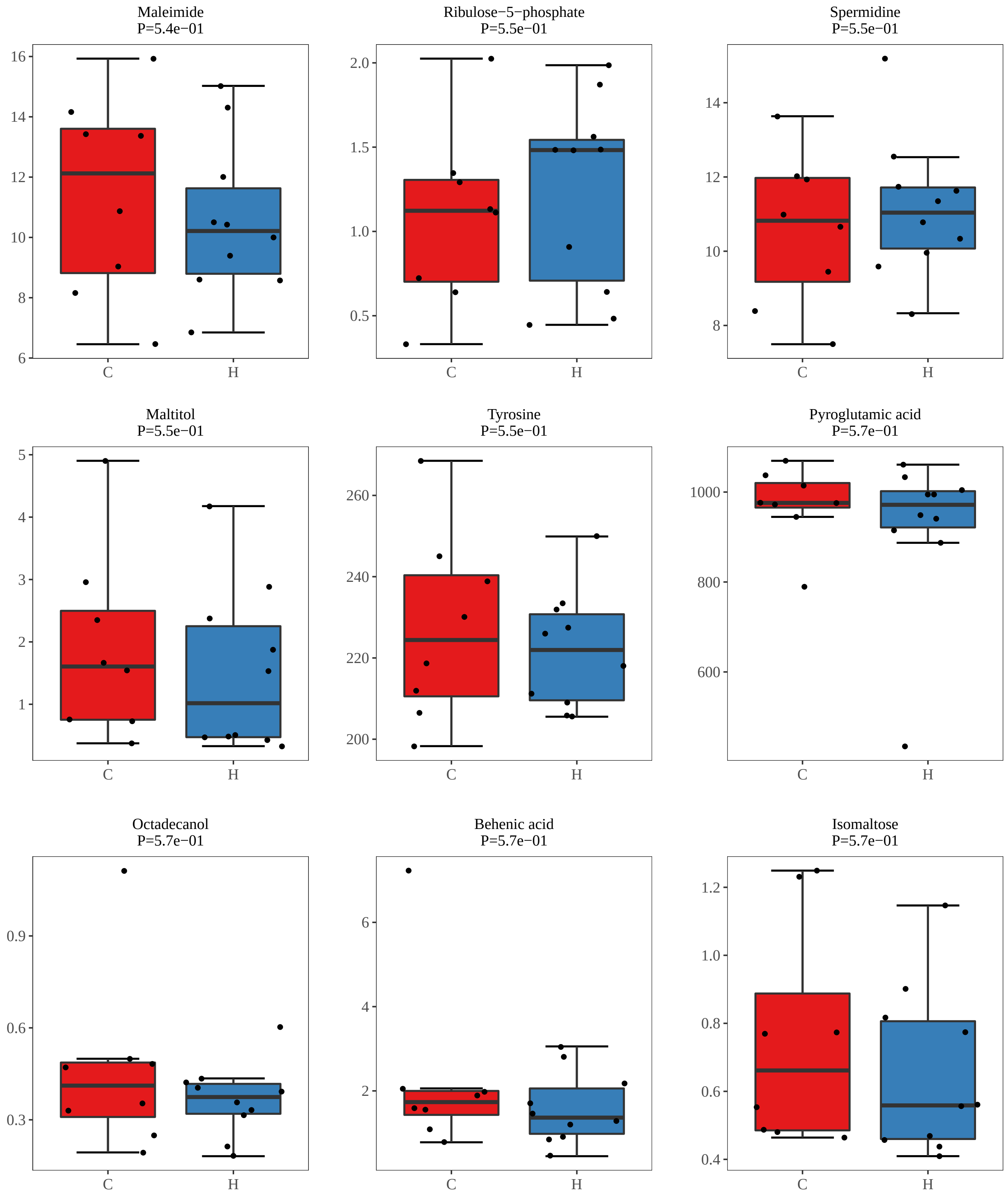

Maleimide
P=5.4e−01
16
14
12
10
8
6
C
H
Ribulose−5−phosphate
P=5.5e−01
2.0
1.5
1.0
0.5
C
H
Spermidine
P=5.5e−01
14
12
10
8
C
H
Maltitol
P=5.5e−01
5
4
3
2
1
C
H
Tyrosine
P=5.5e−01
260
240
220
200
C
H
Pyroglutamic acid
P=5.7e−01
1000
800
600
C
H
Octadecanol
P=5.7e−01
0.9
0.6
0.3
C
H
Behenic acid
P=5.7e−01
6
4
2
C
H
Isomaltose
P=5.7e−01
1.2
1.0
0.8
0.6
0.4
C
H

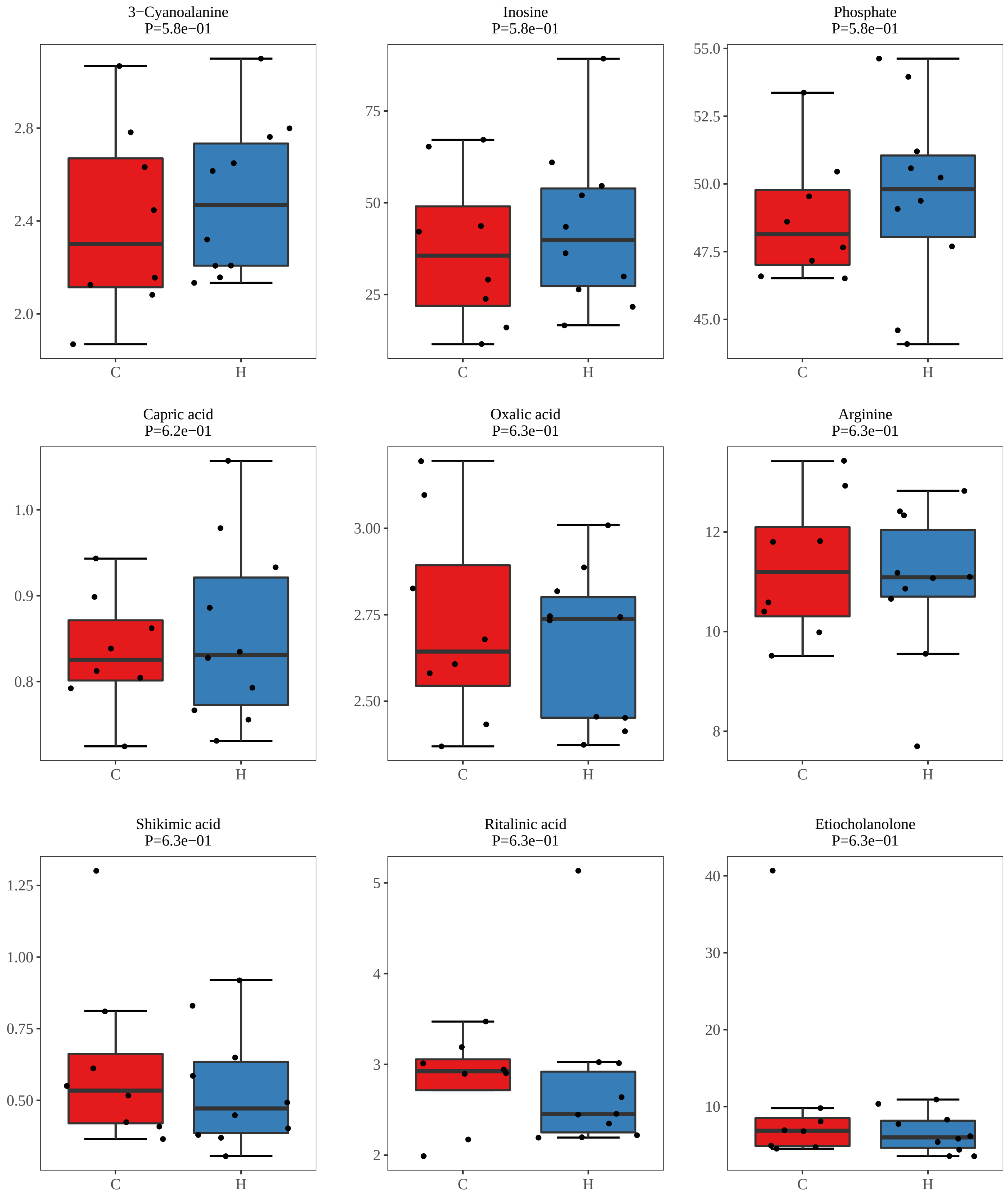
3−Cyanoalanine
P=5.8e−01
2.0
2.4
2.8
C
H
Inosine
P=5.8e−01
25
50
75
C
H
Phosphate
P=5.8e−01
45.0
47.5
50.0
52.5
55.0
C
H
Capric acid
P=6.2e−01
0.8
0.9
1.0
C
H
Oxalic acid
P=6.3e−01
2.50
2.75
3.00
C
H
Arginine
P=6.3e−01
8
10
12
C
H
Shikimic acid
P=6.3e−01
0.50
0.75
1.00
1.25
C
H
Ritalinic acid
P=6.3e−01
2
3
4
5
C
H
Etiocholanolone
P=6.3e−01
10
20
30
40
C
H

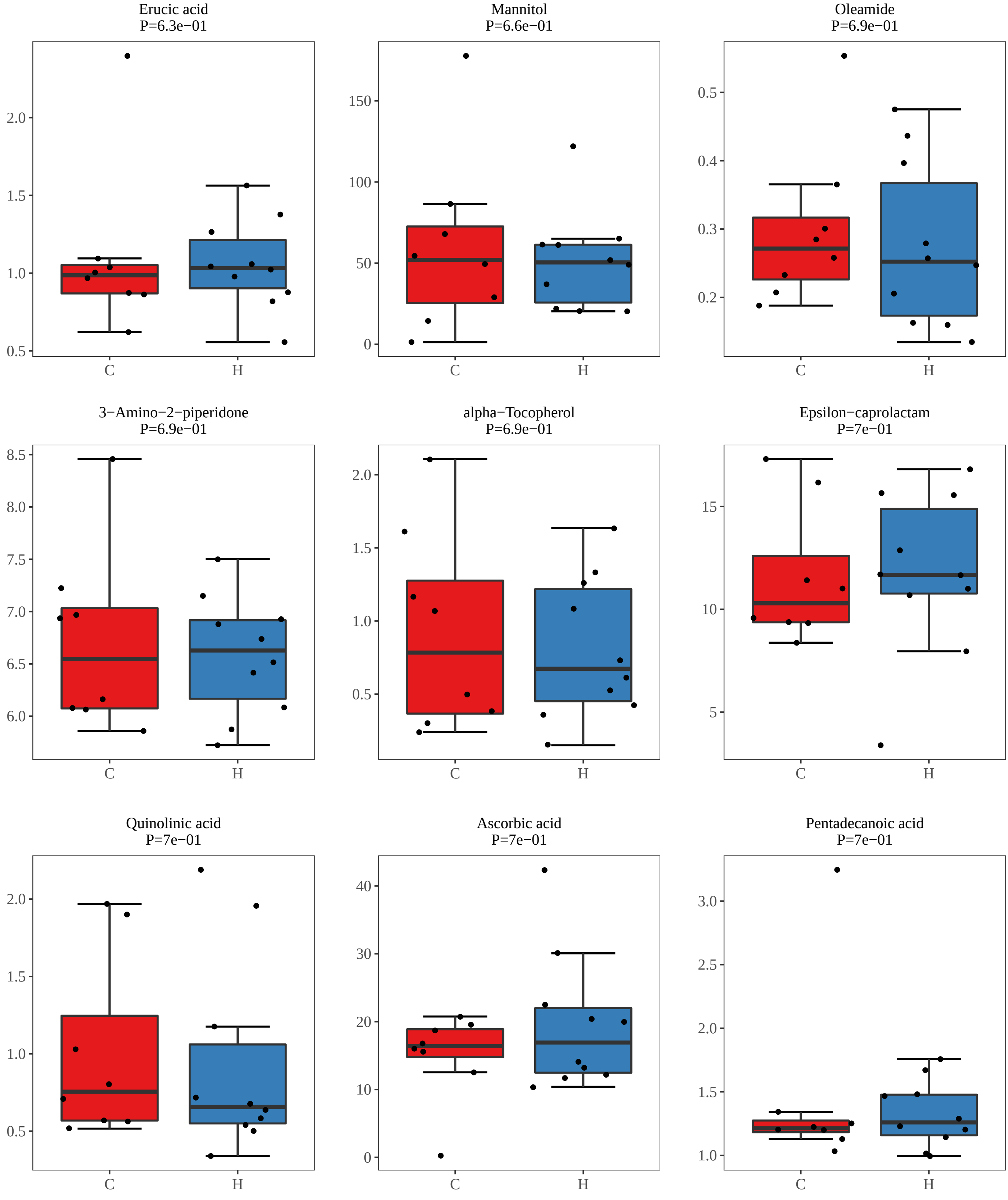
Erucic acid
P=6.3e−01
Mannitol
P=6.6e−01
Oleamide
P=6.9e−01
3−Amino−2−piperidone
P=6.9e−01
alpha−Tocopherol
P=6.9e−01
Epsilon−caprolactam
P=7e−01
Quinolinic acid
P=7e−01
Ascorbic acid
P=7e−01
Pentadecanoic acid
P=7e−01
C
H

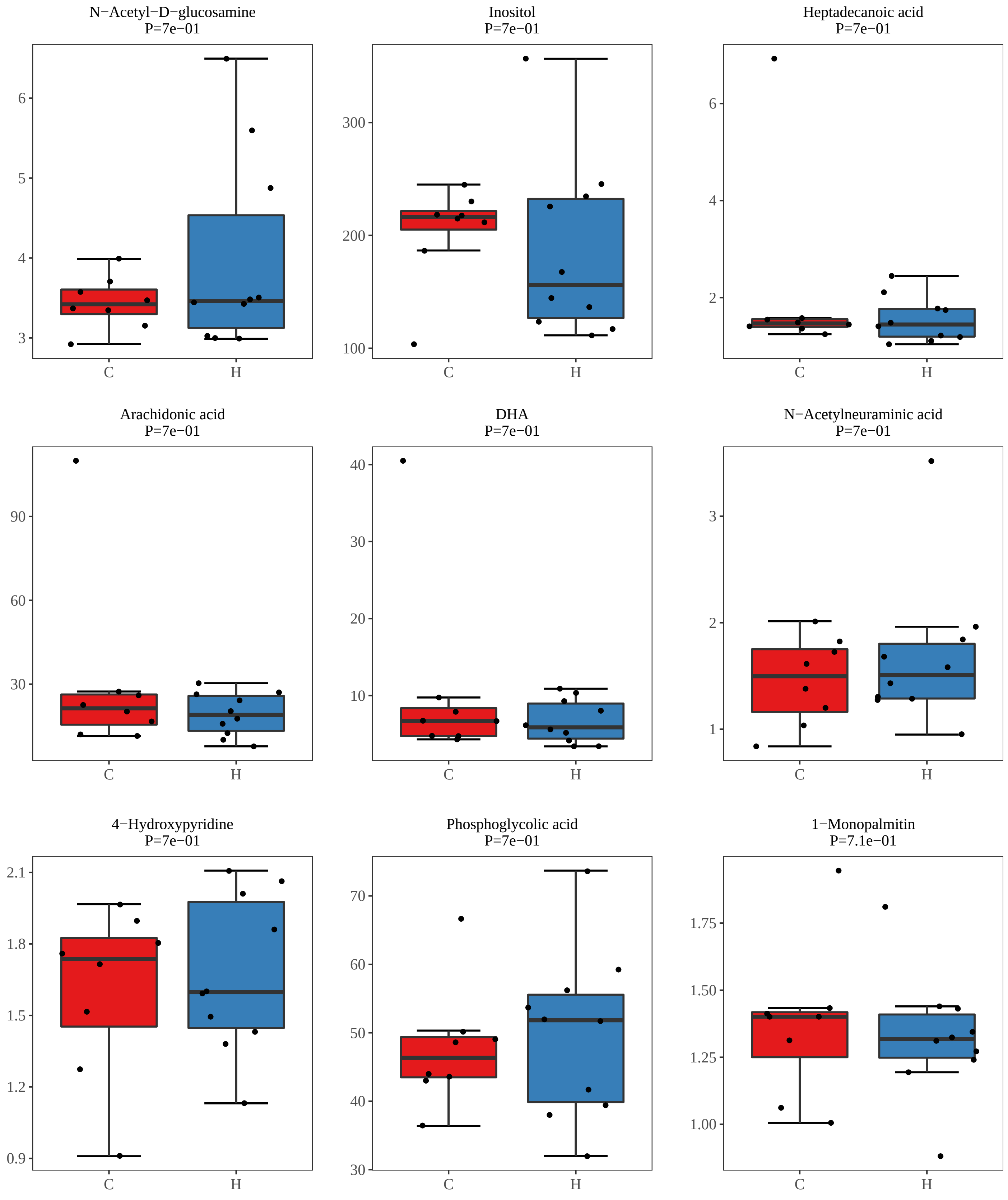
N−Acetyl−D−glucosamine
P=7e−01
6
5
4
3
C
H
Inositol
P=7e−01
300
200
100
C
H
Heptadecanoic acid
P=7e−01
6
4
2
C
H
Arachidonic acid
P=7e−01
90
60
30
C
H
DHA
P=7e−01
40
30
20
10
C
H
N−Acetylneuraminic acid
P=7e−01
3
2
1
C
H
4−Hydroxypyridine
P=7e−01
2.1
1.8
1.5
1.2
0.9
C
H
Phosphoglycolic acid
P=7e−01
70
60
50
40
30
C
H
1−Monopalmitin
P=7.1e−01
1.75
1.50
1.25
1.00
C
H

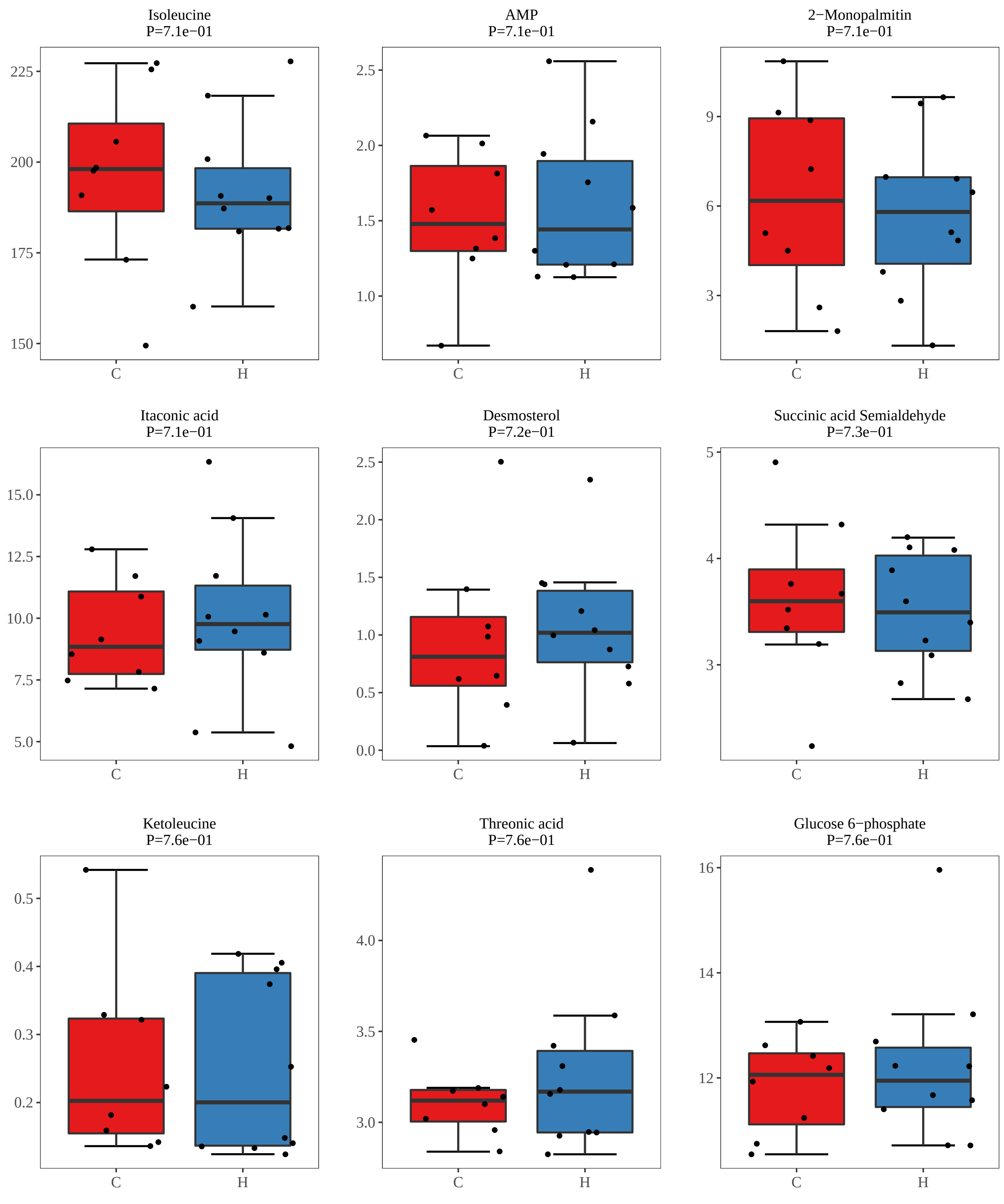
Isoleucine
P=7.1e−01
150
175
200
225
C
H
AMP
P=7.1e−01
1.0
1.5
2.0
2.5
C
H
2−Monopalmitin
P=7.1e−01
3
6
9
C
H
Itaconic acid
P=7.1e−01
5.0
7.5
10.0
12.5
15.0
C
H
Desmosterol
P=7.2e−01
0.0
0.5
1.0
1.5
2.0
2.5
C
H
Succinic acid Semialdehyde
P=7.3e−01
3
4
5
C
H
Ketoleucine
P=7.6e−01
0.2
0.3
0.4
0.5
C
H
Threonic acid
P=7.6e−01
3.0
3.5
4.0
C
H
Glucose 6−phosphate
P=7.6e−01
12
14
16
C
H

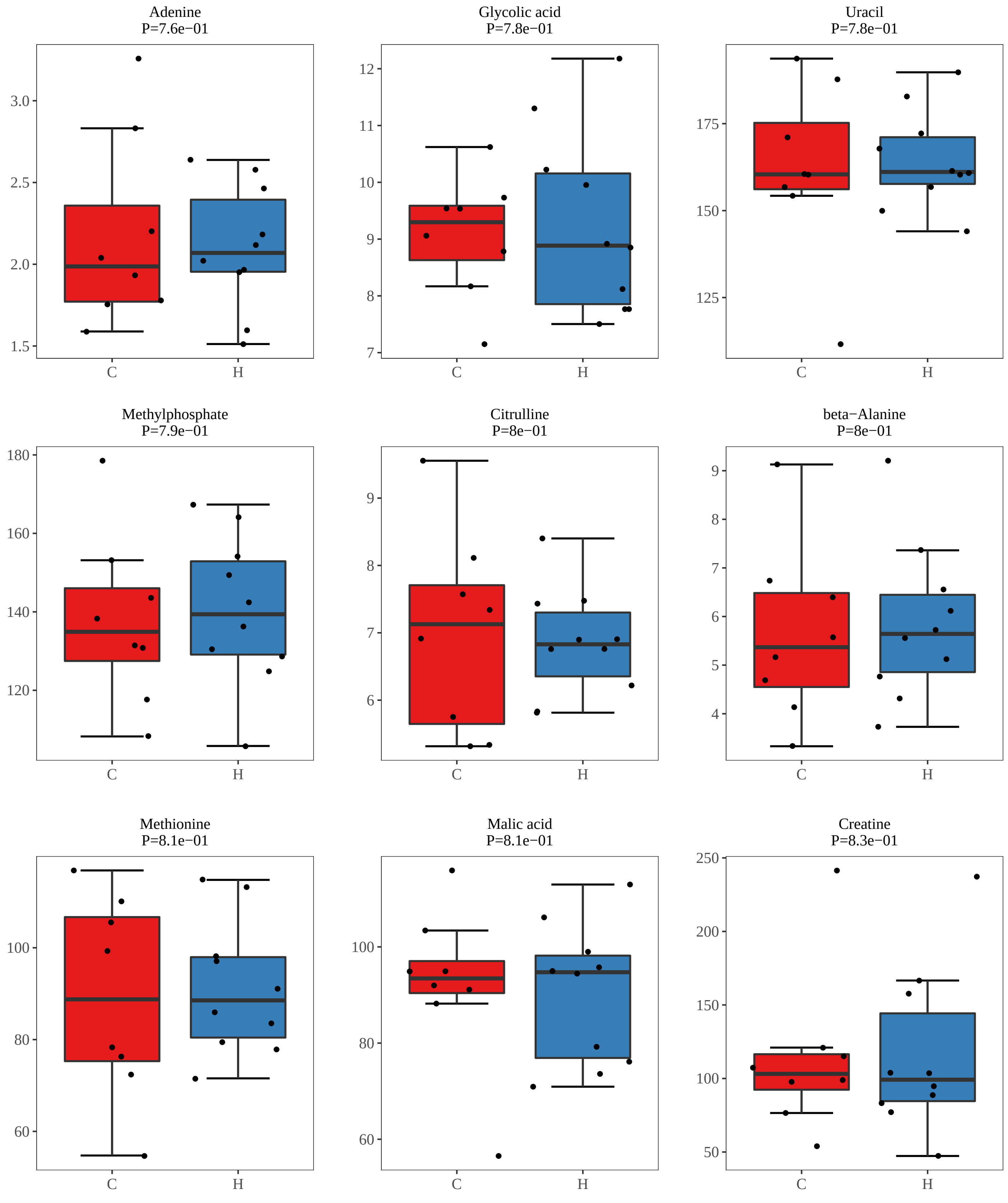
Adenine
P=7.6e−01
Glycolic acid
P=7.8e−01
Uracil
P=7.8e−01
Methylphosphate
P=7.9e−01
Citrulline
P=8e−01
beta−Alanine
P=8e−01
Methionine
P=8.1e−01
Malic acid
P=8.1e−01
Creatine
P=8.3e−01
C
H

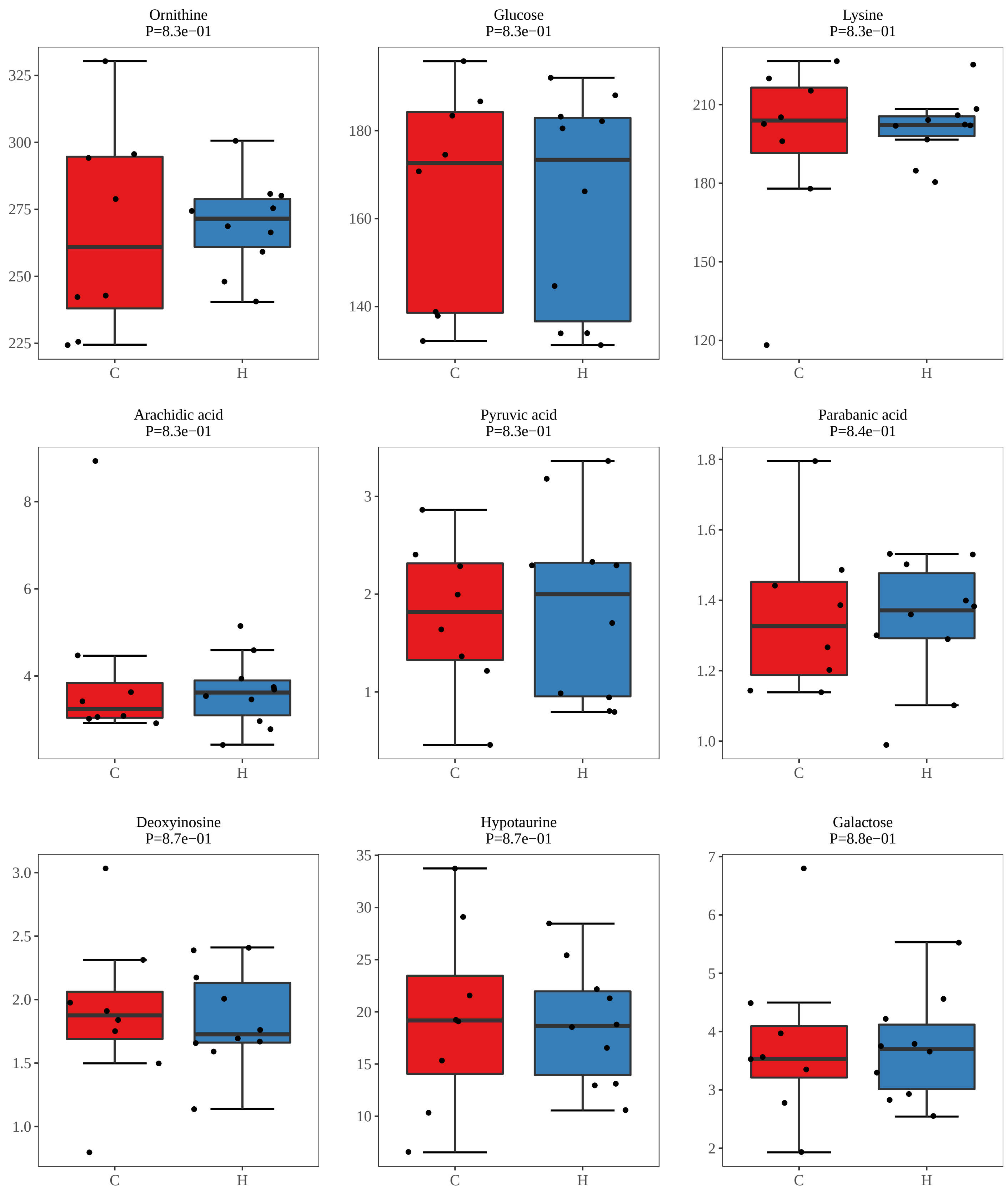

Ornithine
P=8.3e−01
Glucose
P=8.3e−01
Lysine
P=8.3e−01
Arachidic acid
P=8.3e−01
Pyruvic acid
P=8.3e−01
Parabanic acid
P=8.4e−01
Deoxyinosine
P=8.7e−01
Hypotaurine
P=8.7e−01
Galactose
P=8.8e−01
C
H

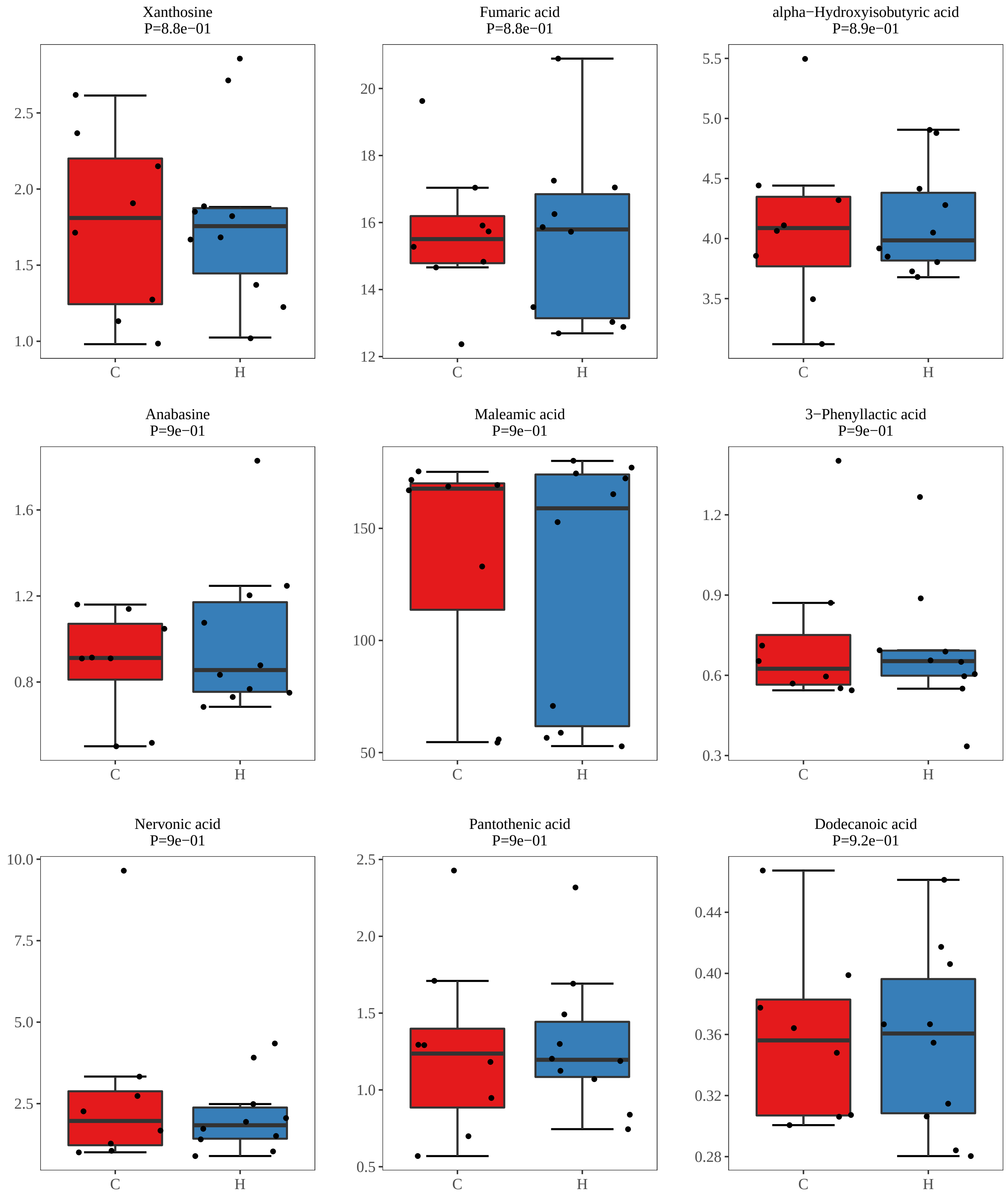

Xanthosine
P=8.8e−01
1.0
1.5
2.0
2.5
Fumaric acid
P=8.8e−01
12
14
16
18
20
alpha−Hydroxyisobutyric acid
P=8.9e−01
3.5
4.0
4.5
5.0
5.5
Anabasine
P=9e−01
0.8
1.2
1.6
Maleamic acid
P=9e−01
50
100
150
3−Phenyllactic acid
P=9e−01
0.3
0.6
0.9
1.2
Nervonic acid
P=9e−01
2.5
5.0
7.5
10.0
Pantothenic acid
P=9e−01
0.5
1.0
1.5
2.0
2.5
Dodecanoic acid
P=9.2e−01
0.28
0.32
0.36
0.40
0.44
C
H

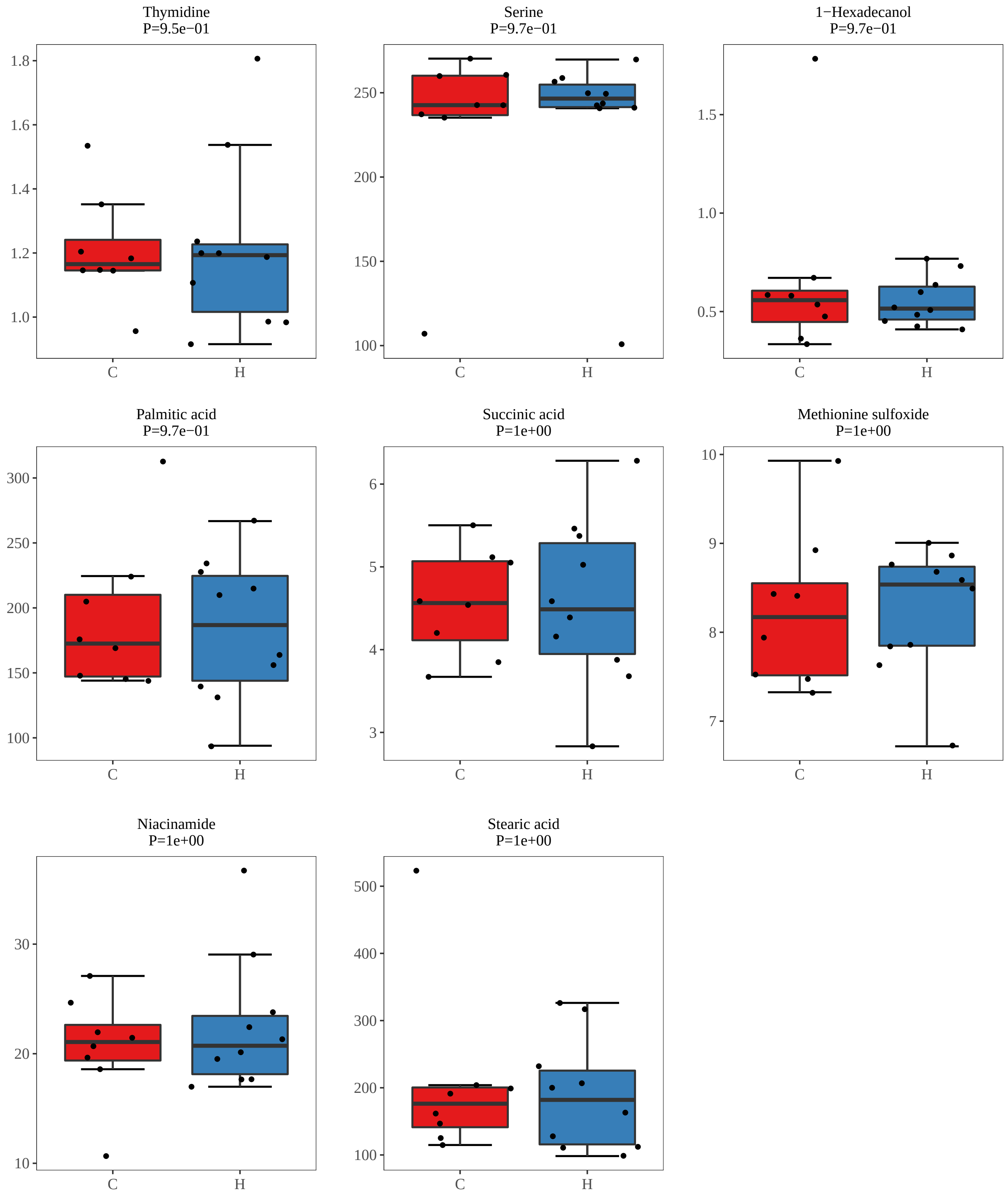

Thymidine
P=9.5e−01
1.8
1.6
1.4
1.2
1.0
C
H
Serine
P=9.7e−01
250
200
150
100
C
H
1−Hexadecanol
P=9.7e−01
1.5
1.0
0.5
C
H
Palmitic acid
P=9.7e−01
300
250
200
150
100
C
H
Succinic acid
P=1e+00
6
5
4
3
C
H
Methionine sulfoxide
P=1e+00
10
9
8
7
C
H
Niacinamide
P=1e+00
30
20
10
C
H
Stearic acid
P=1e+00
500
400
300
200
100
C
H
