# Supplementary material for: Yiai Fuzheng decoction inhibits triple-negative breast cancer by remodeling the immune microenvironment
Source: Front Immunol. 2025 Sep 30;16:1615631. doi: 10.3389/fimmu.2025.1615631 (PMC12518410; doi:10.3389/fimmu.2025.1615631)
Supplement: Supplementary file 2 [file DataSheet1.zip › Supplementary File 2/Treatment/C_vs_H/05_Univariate_Analysis/AllMet_Vioplot.pdf]

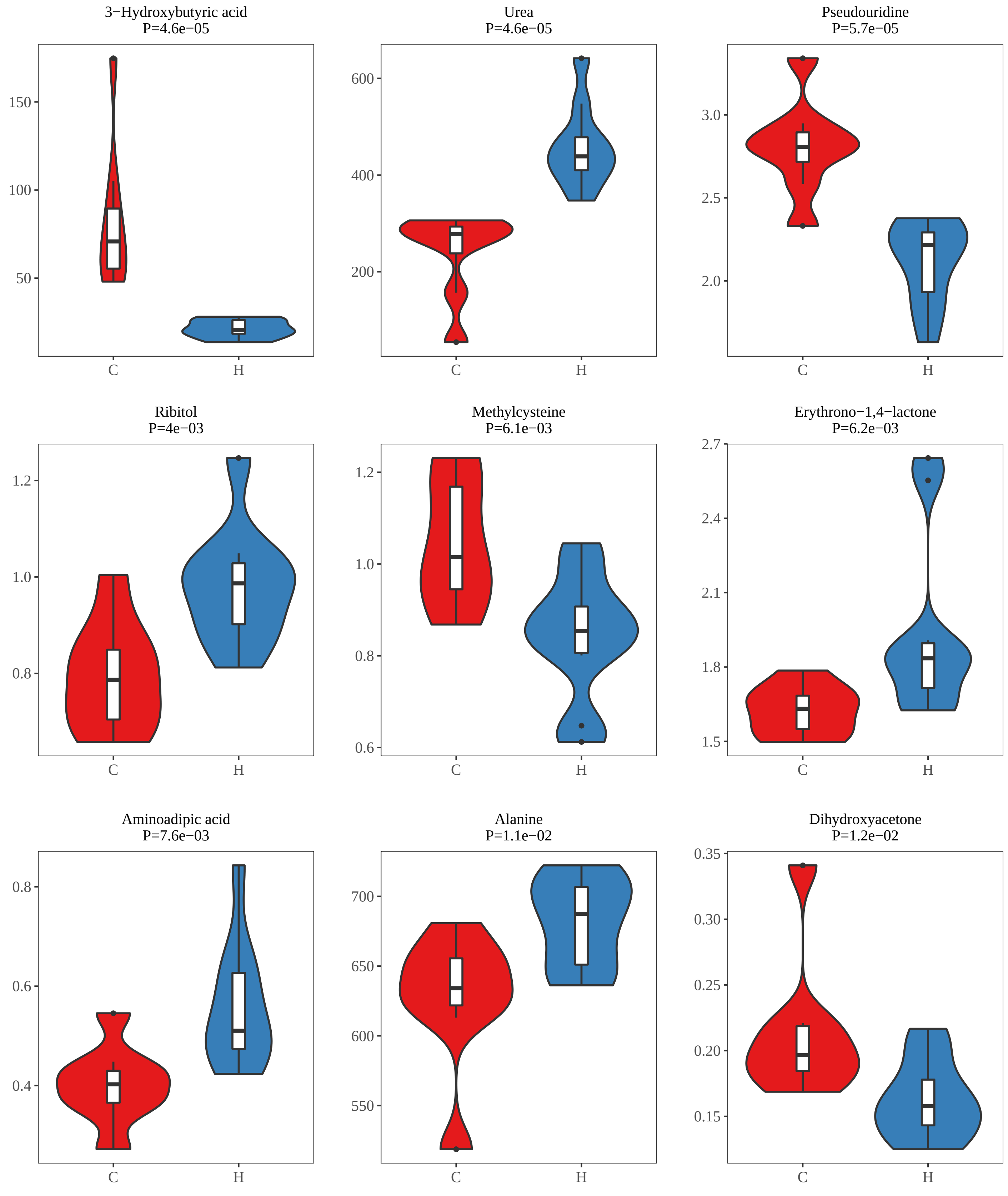
3−Hydroxybutyric acid
P=4.6e−05
150
100
50
C
H
Urea
P=4.6e−05
600
400
200
C
H
Pseudouridine
P=5.7e−05
3.0
2.5
2.0
C
H
Ribitol
P=4e−03
1.2
1.0
0.8
C
H
Methylcysteine
P=6.1e−03
1.2
1.0
0.8
0.6
C
H
Erythrono−1,4−lactone
P=6.2e−03
2.7
2.4
2.1
1.8
1.5
C
H
Aminoadipic acid
P=7.6e−03
0.8
0.6
0.4
C
H
Alanine
P=1.1e−02
700
650
600
550
C
H
Dihydroxyacetone
P=1.2e−02
0.35
0.30
0.25
0.20
0.15
C
H

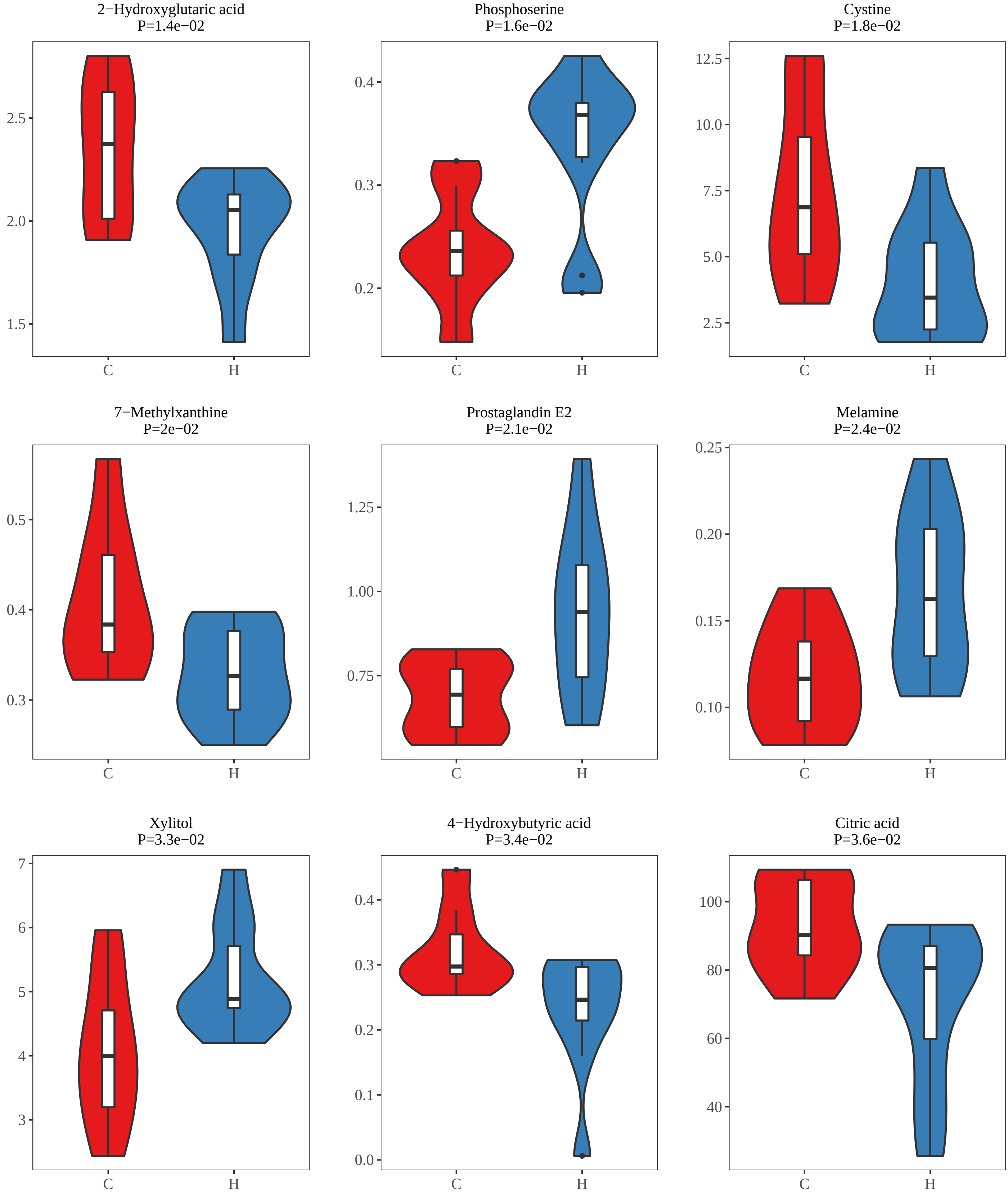
2−Hydroxyglutaric acid
P=1.4e−02
Phosphoserine
P=1.6e−02
Cystine
P=1.8e−02
7−Methylxanthine
P=2e−02
Prostaglandin E2
P=2.1e−02
Melamine
P=2.4e−02
Xylitol
P=3.3e−02
4−Hydroxybutyric acid
P=3.4e−02
Citric acid
P=3.6e−02
C
H

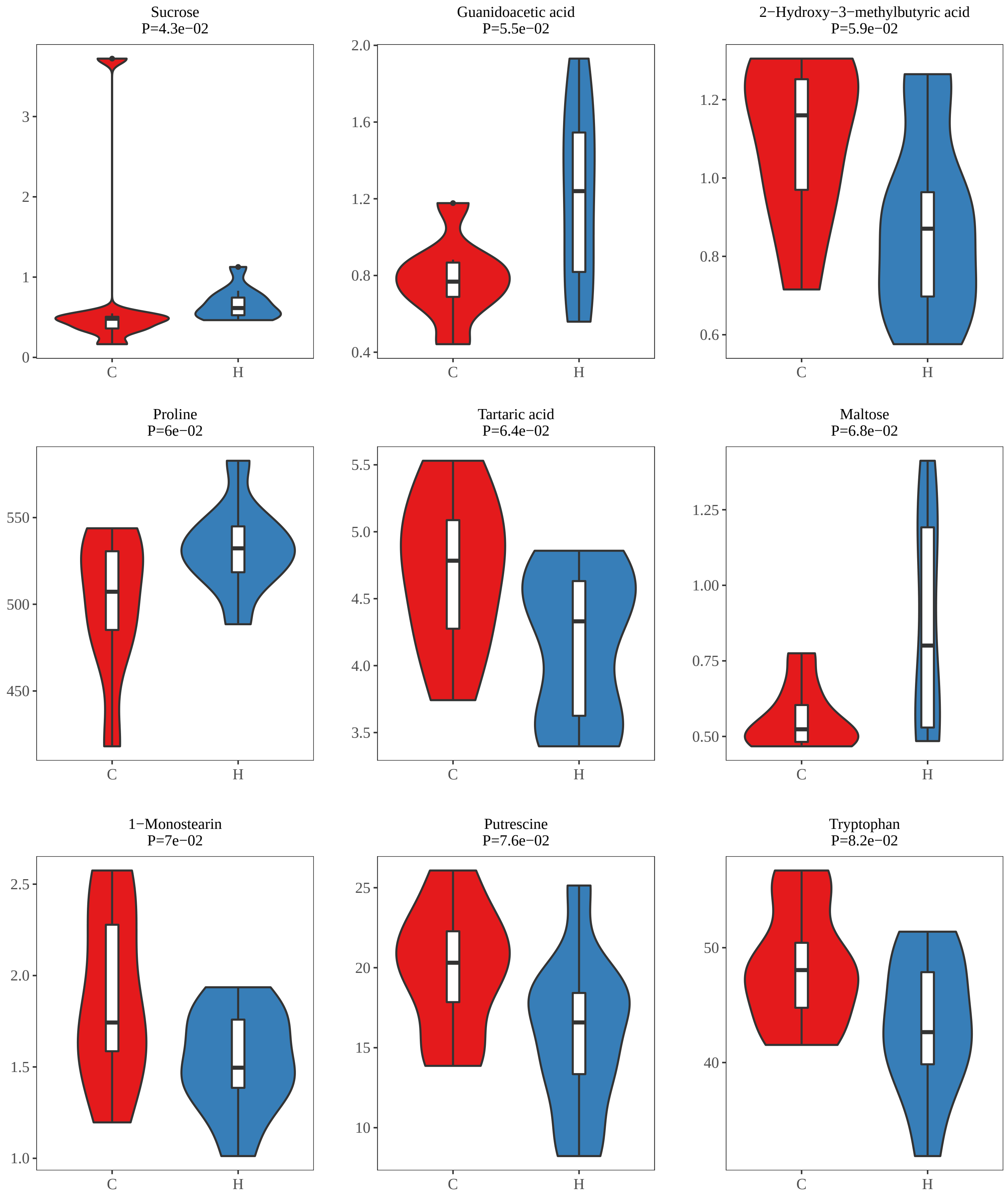

Sucrose
P=4.3e−02
Guanidoacetic acid
P=5.5e−02
2−Hydroxy−3−methylbutyric acid
P=5.9e−02
Proline
P=6e−02
Tartaric acid
P=6.4e−02
Maltose
P=6.8e−02
1−Monostearin
P=7e−02
Putrescine
P=7.6e−02
Tryptophan
P=8.2e−02
C
H

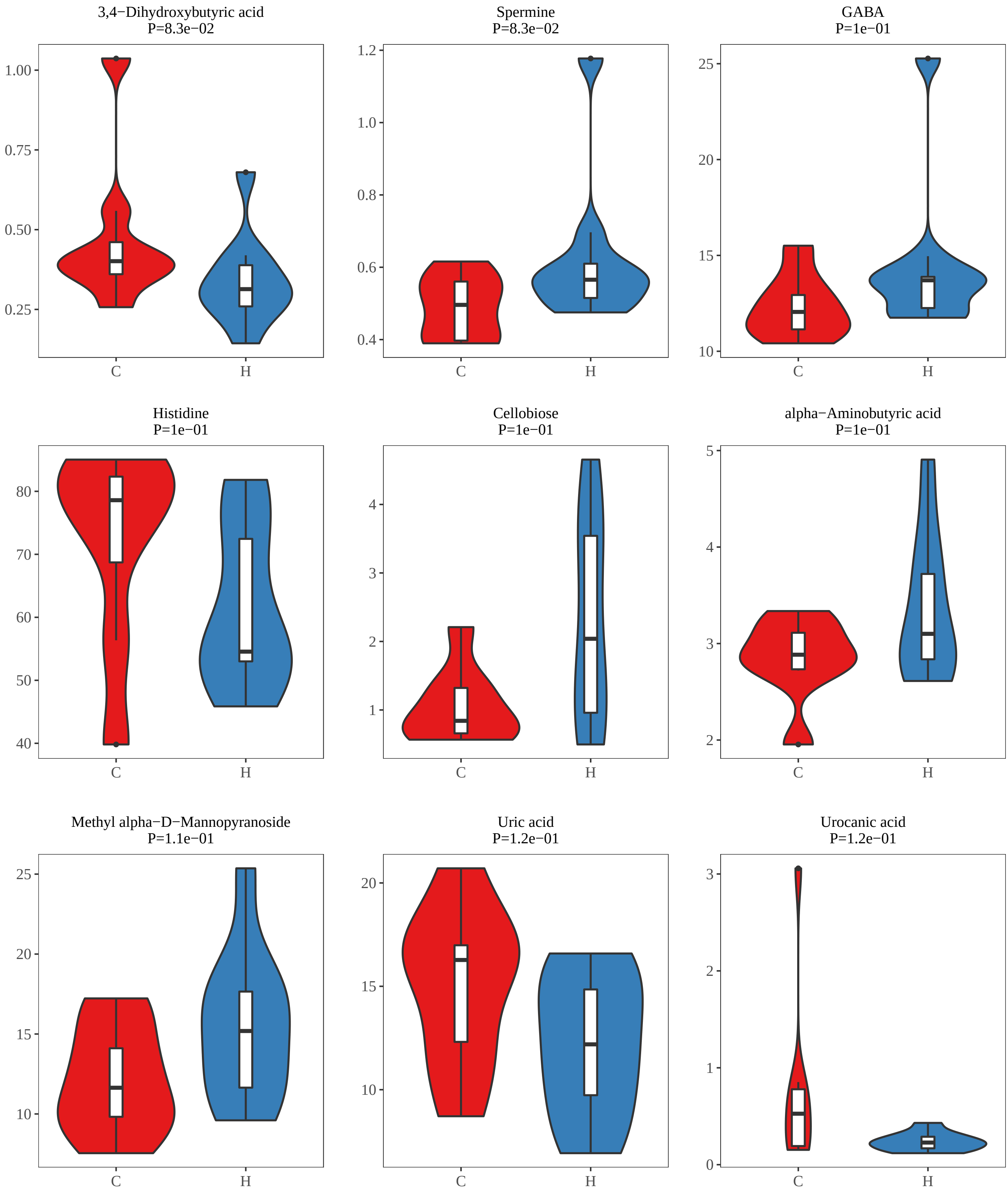

3,4−Dihydroxybutyric acid
P=8.3e−02
1.00
0.75
0.50
0.25
C
H
Spermine
P=8.3e−02
1.2
1.0
0.8
0.6
0.4
C
H
GABA
P=1e−01
25
20
15
10
C
H
Histidine
P=1e−01
80
70
60
50
40
C
H
Cellobiose
P=1e−01
4
3
2
1
C
H
alpha−Aminobutyric acid
P=1e−01
5
4
3
2
C
H
Methyl alpha−D−Mannopyranoside
P=1.1e−01
25
20
15
10
C
H
Uric acid
P=1.2e−01
20
15
10
C
H
Urocanic acid
P=1.2e−01
3
2
1
0
C
H

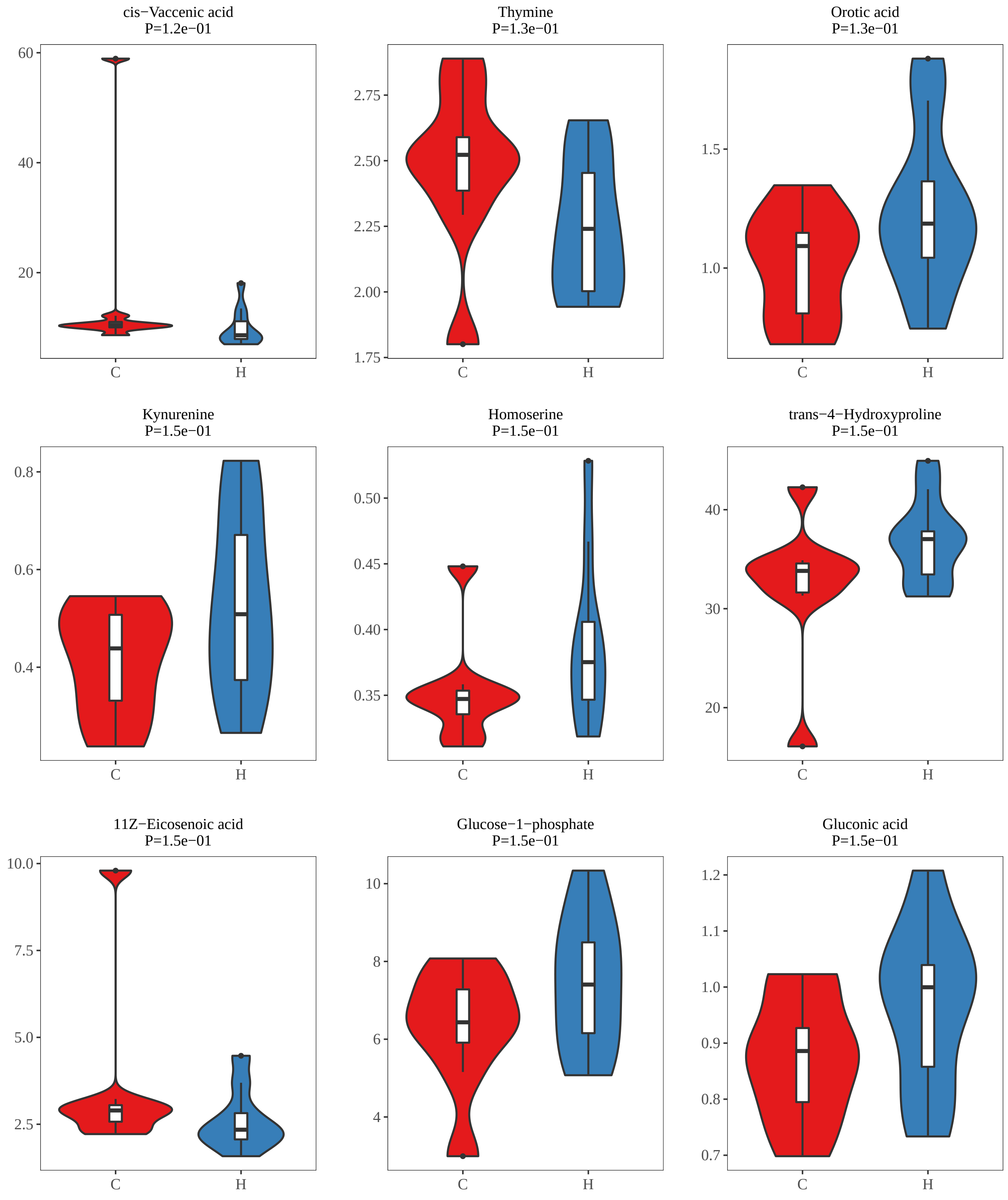
cis−Vaccenic acid
P=1.2e−01
Thymine
P=1.3e−01
Orotic acid
P=1.3e−01
Kynurenine
P=1.5e−01
Homoserine
P=1.5e−01
trans−4−Hydroxyproline
P=1.5e−01
11Z−Eicosenoic acid
P=1.5e−01
Glucose−1−phosphate
P=1.5e−01
Gluconic acid
P=1.5e−01
C
H

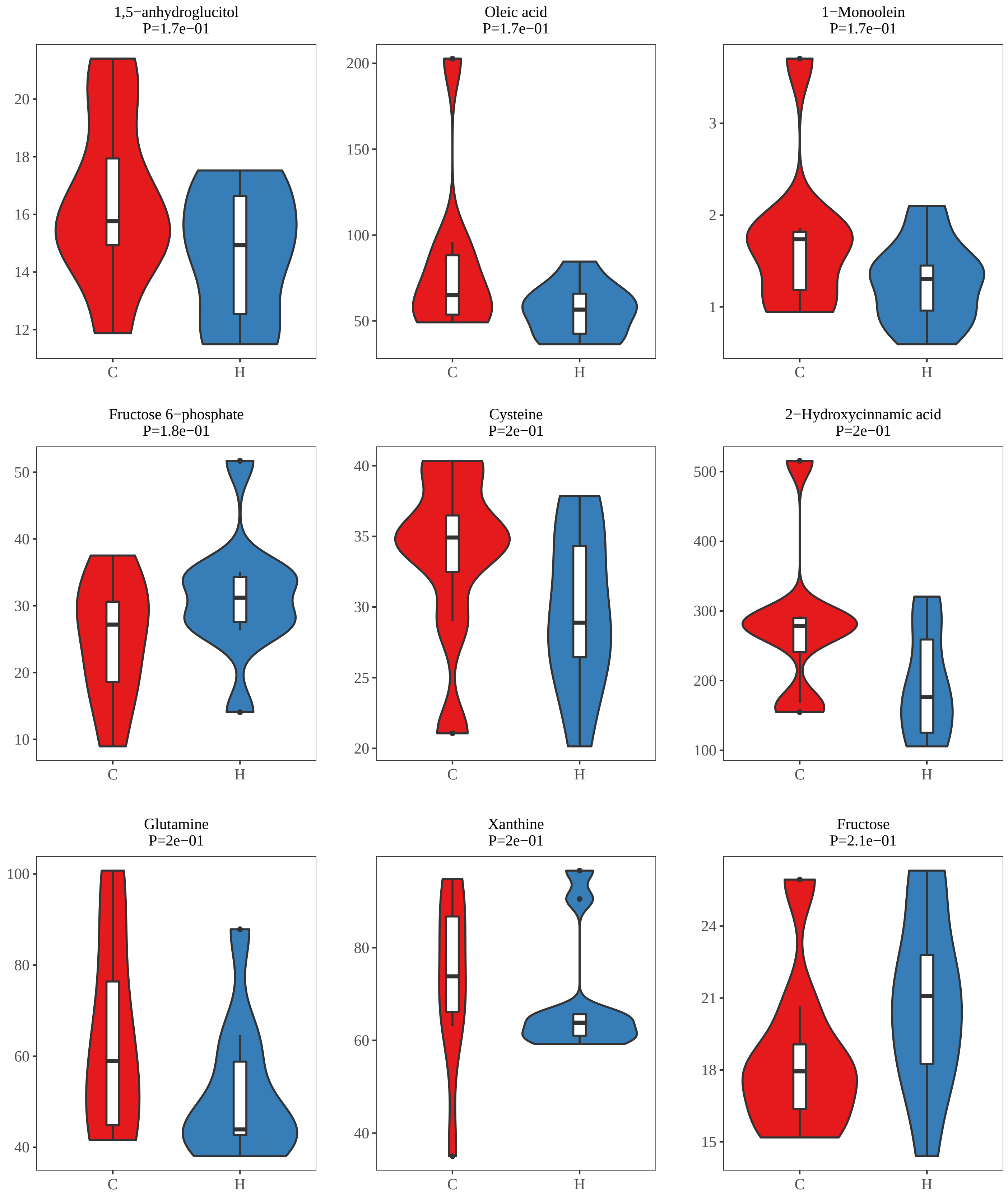
1,5−anhydroglucitol
P=1.7e−01
Oleic acid
P=1.7e−01
1−Monoolein
P=1.7e−01
Fructose 6−phosphate
P=1.8e−01
Cysteine
P=2e−01
2−Hydroxycinnamic acid
P=2e−01
Glutamine
P=2e−01
Xanthine
P=2e−01
Fructose
P=2.1e−01
C
H

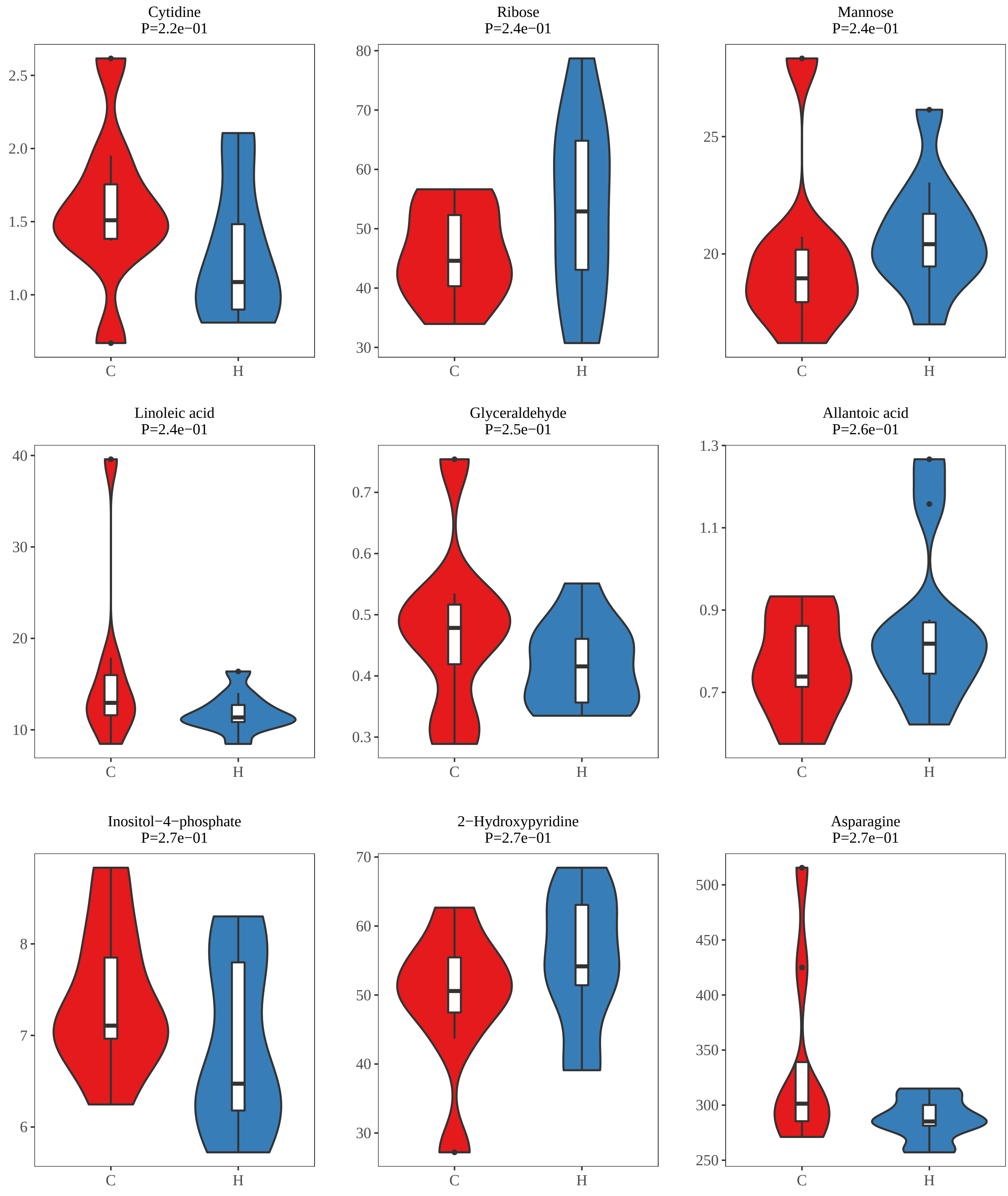

Cytidine
P=2.2e−01
Ribose
P=2.4e−01
Mannose
P=2.4e−01
Linoleic acid
P=2.4e−01
Glyceraldehyde
P=2.5e−01
Allantoic acid
P=2.6e−01
Inositol−4−phosphate
P=2.7e−01
2−Hydroxypyridine
P=2.7e−01
Asparagine
P=2.7e−01
C
H

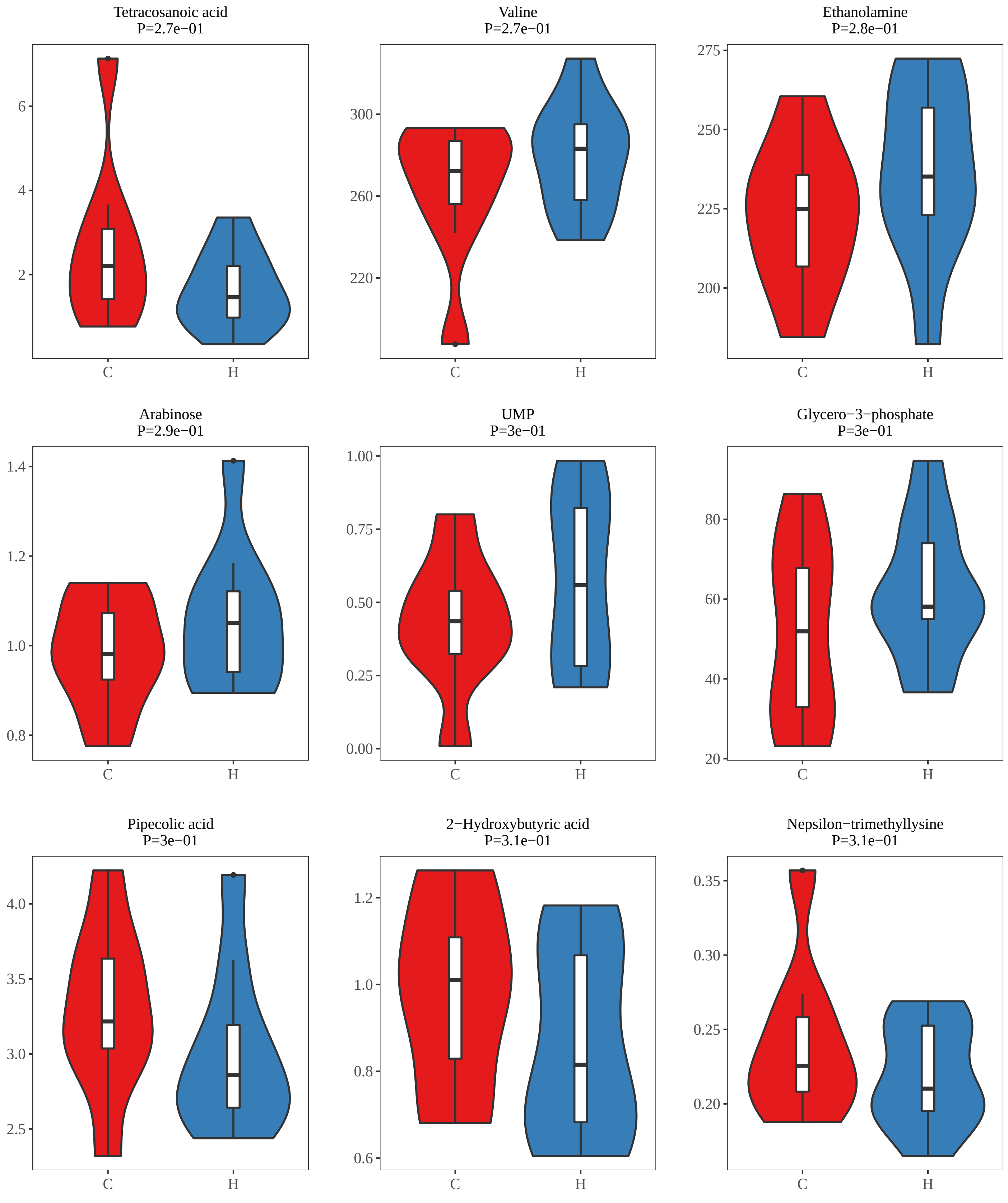
Tetracosanoic acid
P=2.7e−01
Valine
P=2.7e−01
Ethanolamine
P=2.8e−01
Arabinose
P=2.9e−01
UMP
P=3e−01
Glycero−3−phosphate
P=3e−01
Pipecolic acid
P=3e−01
2−Hydroxybutyric acid
P=3.1e−01
Nepsilon−trimethyllysine
P=3.1e−01
C
H

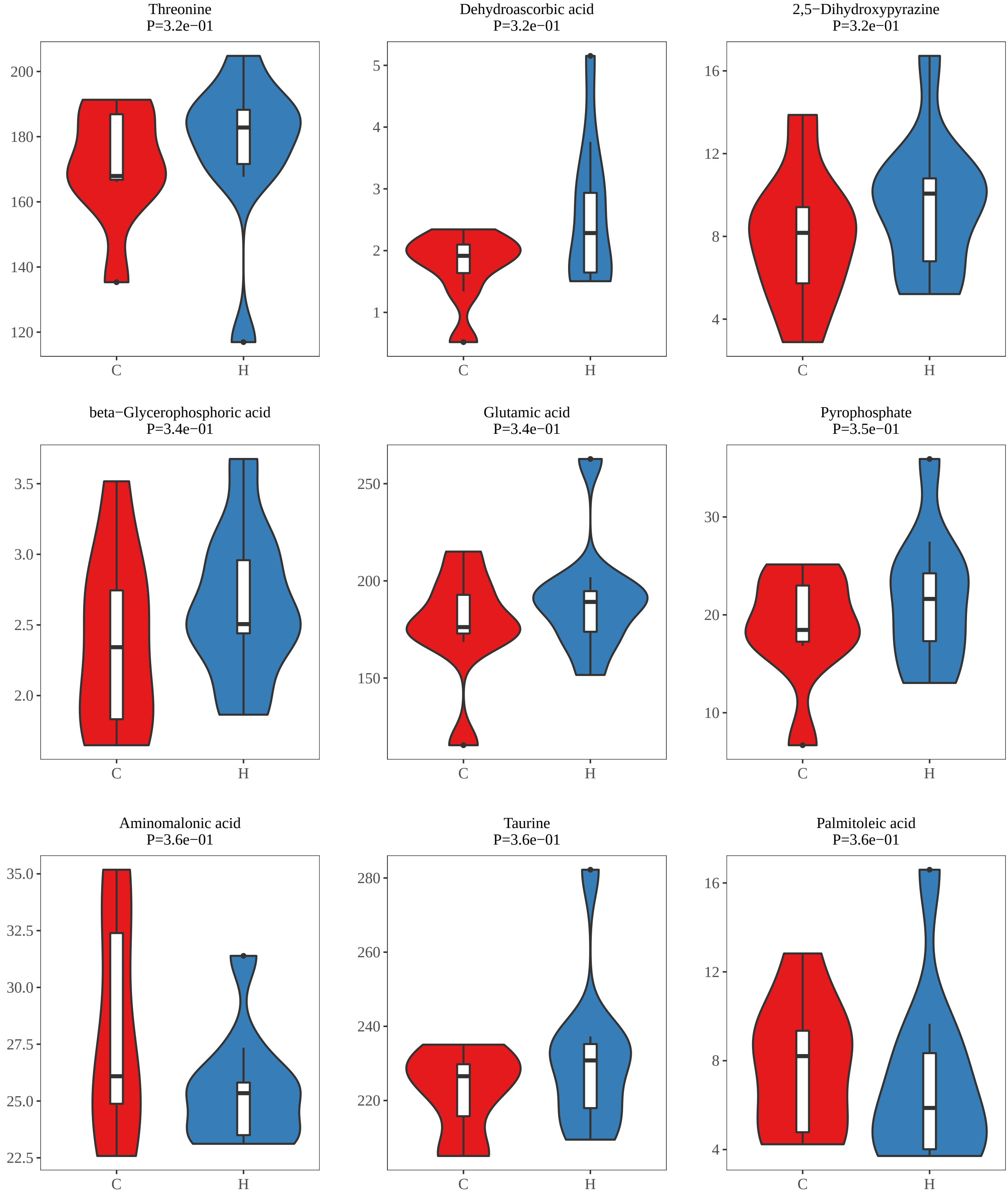
Threonine
P=3.2e−01
Dehydroascorbic acid
P=3.2e−01
2,5−Dihydroxypyrazine
P=3.2e−01
beta−Glycerophosphoric acid
P=3.4e−01
Glutamic acid
P=3.4e−01
Pyrophosphate
P=3.5e−01
Aminomalonic acid
P=3.6e−01
Taurine
P=3.6e−01
Palmitoleic acid
P=3.6e−01
C
H

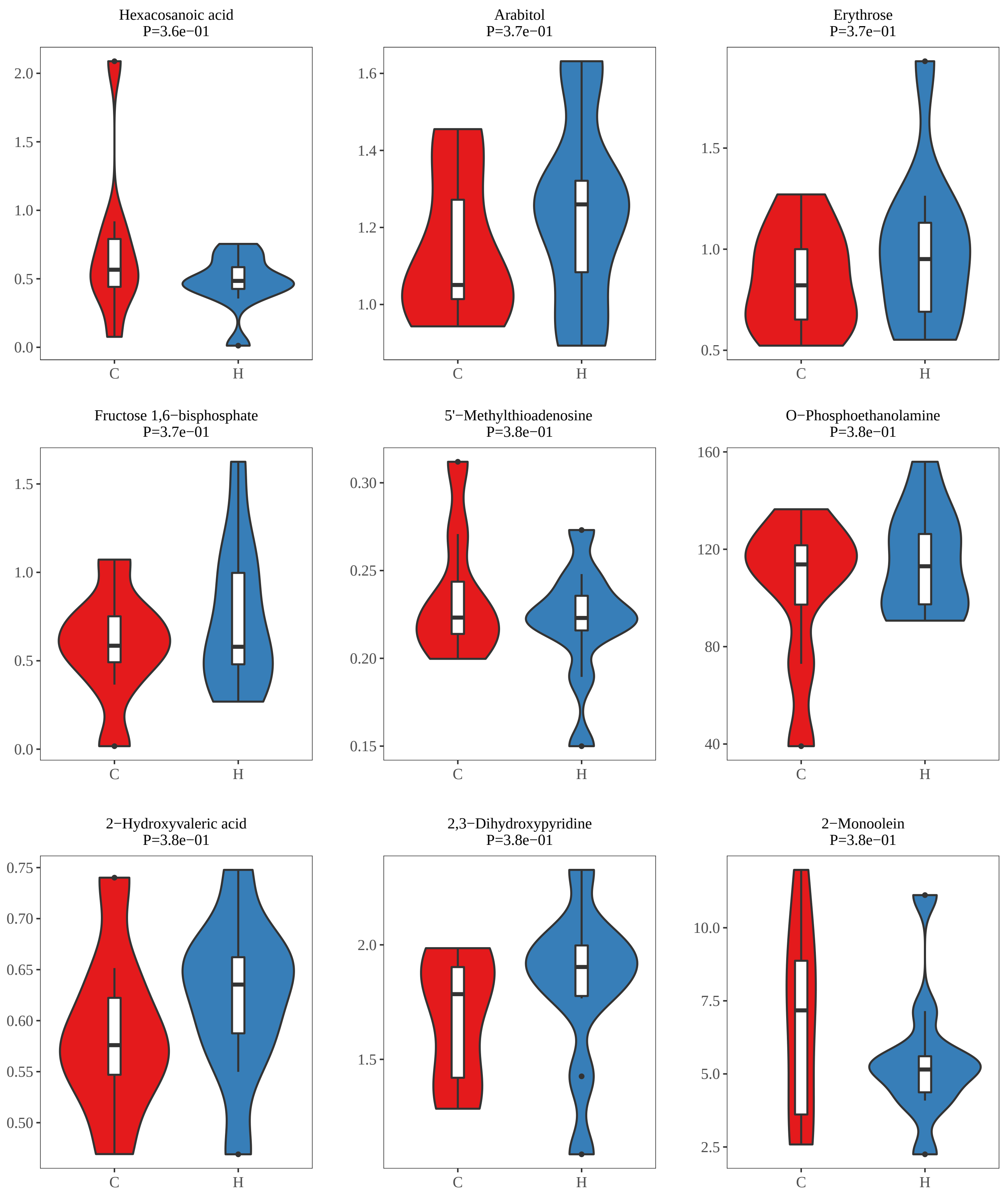

Hexacosanoic acid
P=3.6e−01
Arabitol
P=3.7e−01
Erythrose
P=3.7e−01
Fructose 1,6−bisphosphate
P=3.7e−01
5'−Methylthioadenosine
P=3.8e−01
O−Phosphoethanolamine
P=3.8e−01
2−Hydroxyvaleric acid
P=3.8e−01
2,3−Dihydroxypyridine
P=3.8e−01
2−Monoolein
P=3.8e−01
C
H

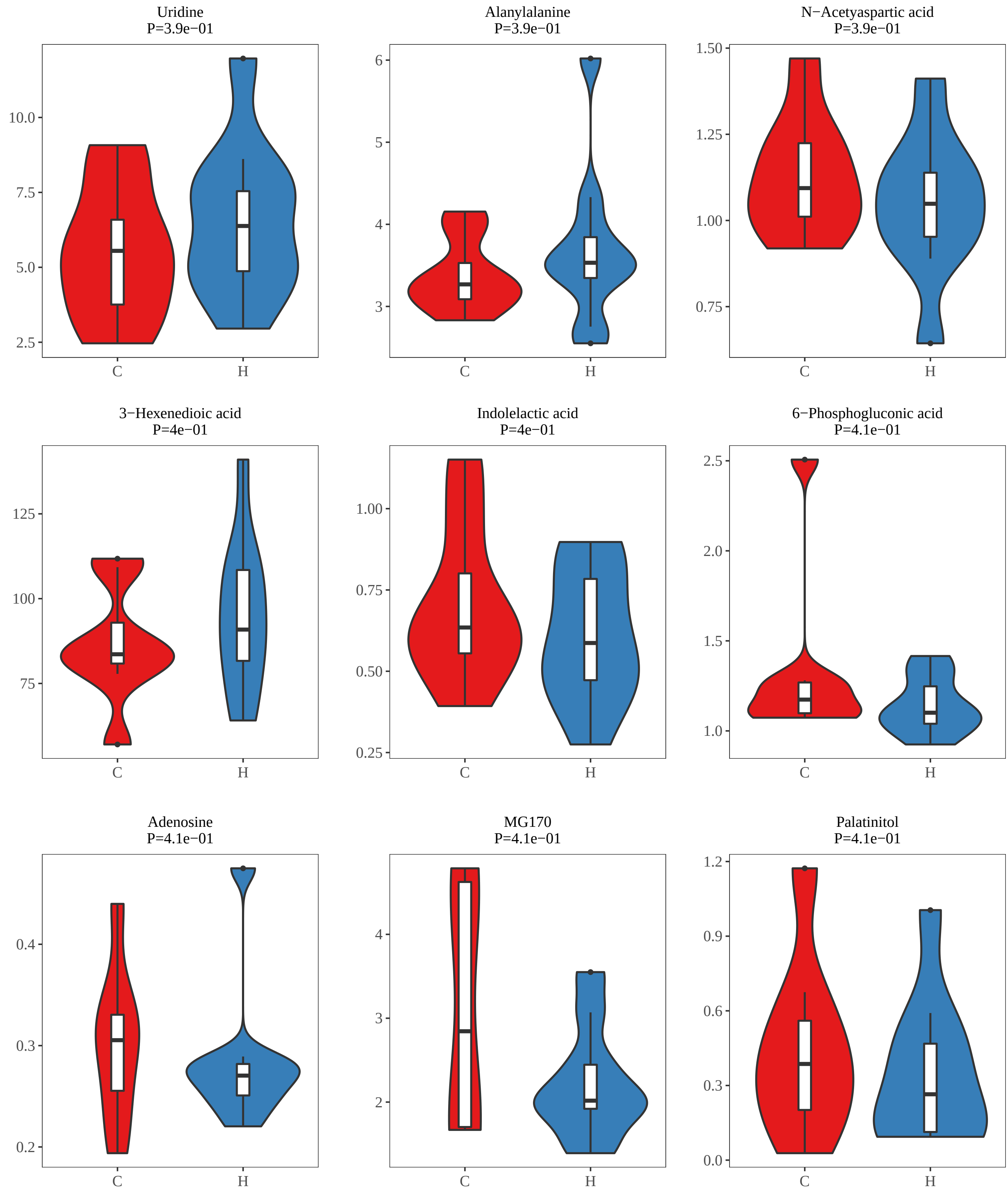

Uridine
P=3.9e−01
Alanylalanine
P=3.9e−01
N−Acetyaspartic acid
P=3.9e−01
3−Hexenedioic acid
P=4e−01
Indolelactic acid
P=4e−01
6−Phosphogluconic acid
P=4.1e−01
Adenosine
P=4.1e−01
MG170
P=4.1e−01
Palatinitol
P=4.1e−01
C
H

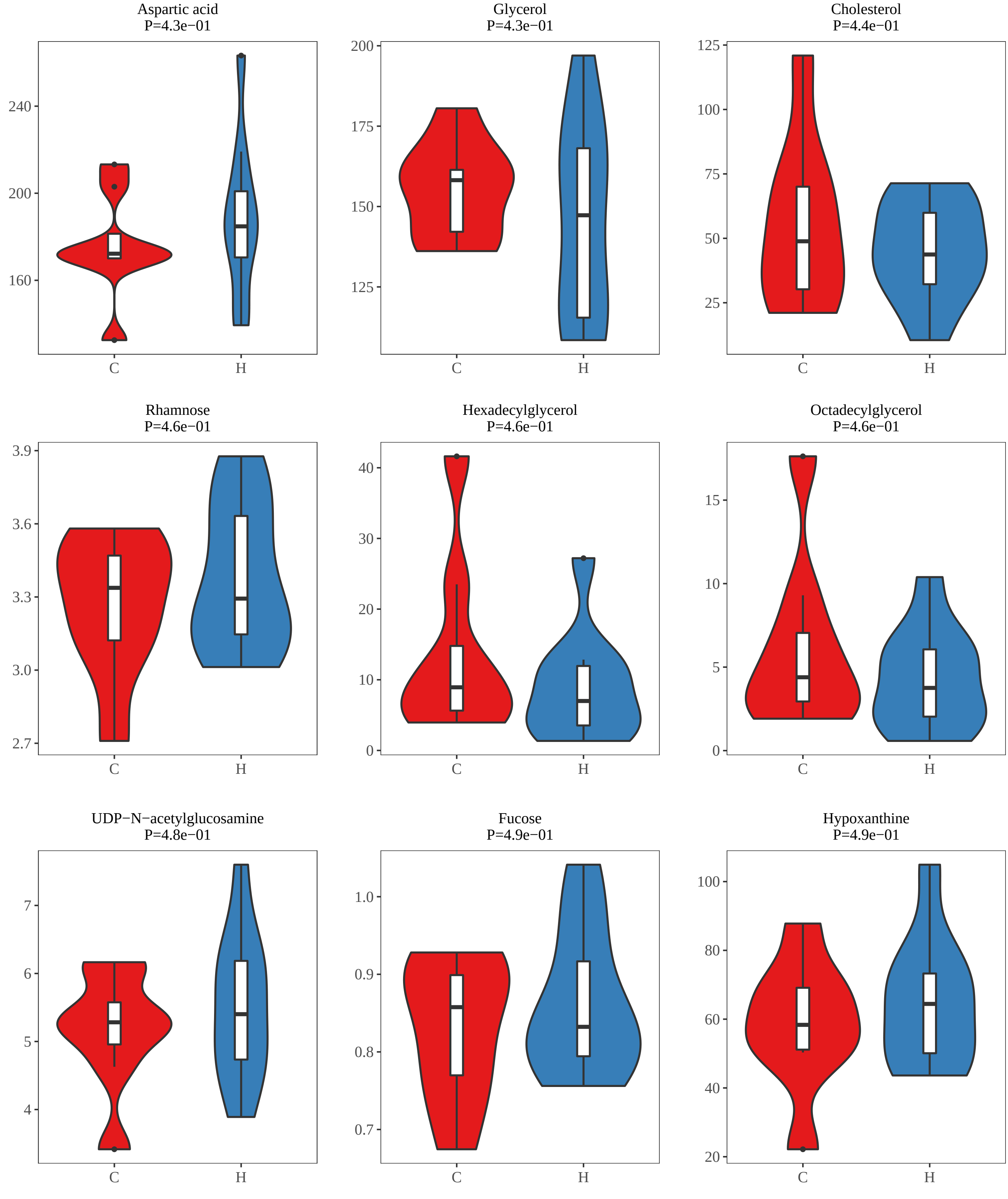
Aspartic acid
P=4.3e−01
240
200
160
C
H
Glycerol
P=4.3e−01
200
175
150
125
C
H
Cholesterol
P=4.4e−01
125
100
75
50
25
C
H
Rhamnose
P=4.6e−01
3.9
3.6
3.3
3.0
2.7
C
H
Hexadecylglycerol
P=4.6e−01
40
30
20
10
0
C
H
Octadecylglycerol
P=4.6e−01
15
10
5
0
C
H
UDP−N−acetylglucosamine
P=4.8e−01
7
6
5
4
C
H
Fucose
P=4.9e−01
1.0
0.9
0.8
0.7
C
H
Hypoxanthine
P=4.9e−01
100
80
60
40
20
C
H

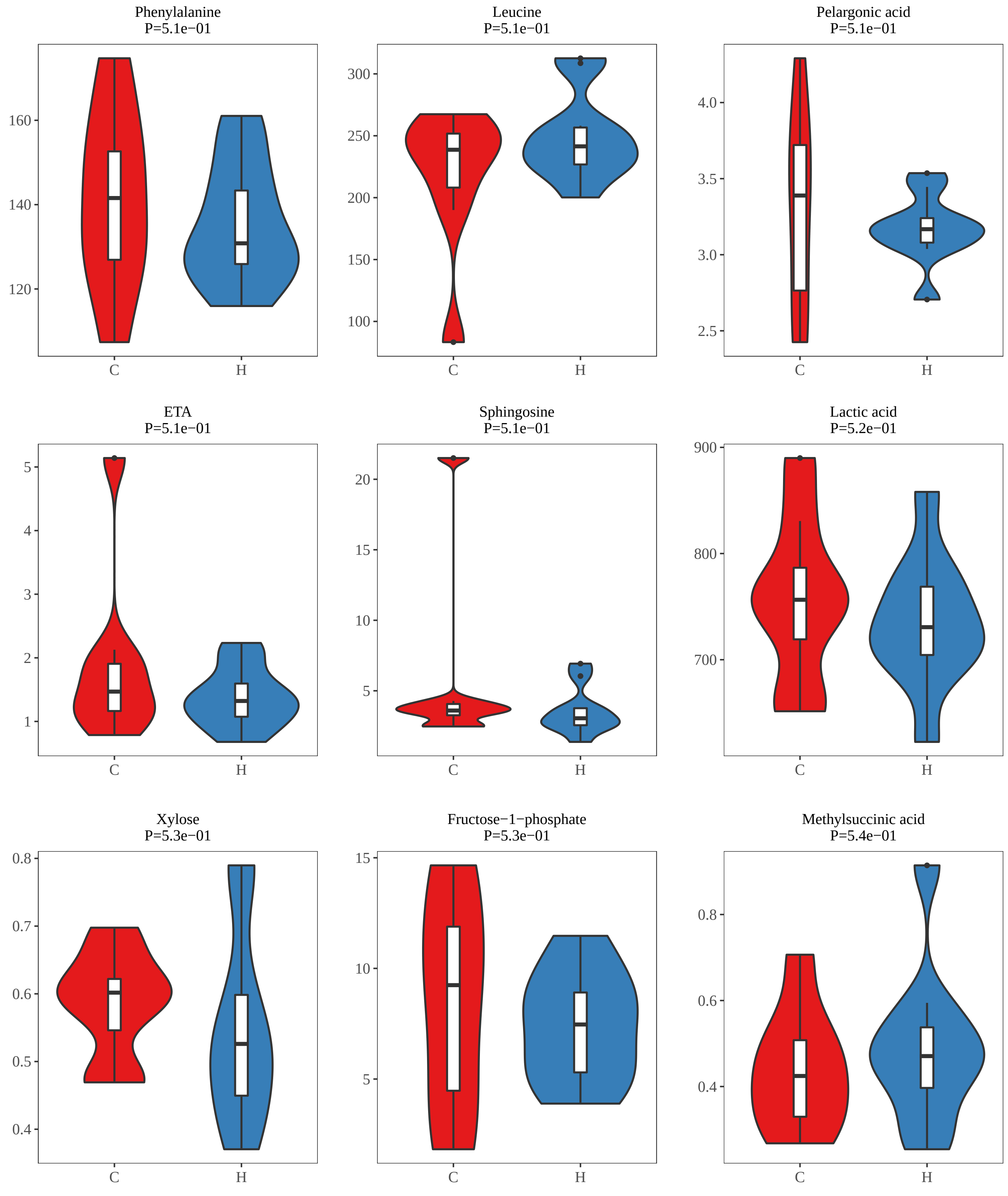
Phenylalanine
P=5.1e−01
Leucine
P=5.1e−01
Pelargonic acid
P=5.1e−01
ETA
P=5.1e−01
Sphingosine
P=5.1e−01
Lactic acid
P=5.2e−01
Xylose
P=5.3e−01
Fructose−1−phosphate
P=5.3e−01
Methylsuccinic acid
P=5.4e−01
C
H

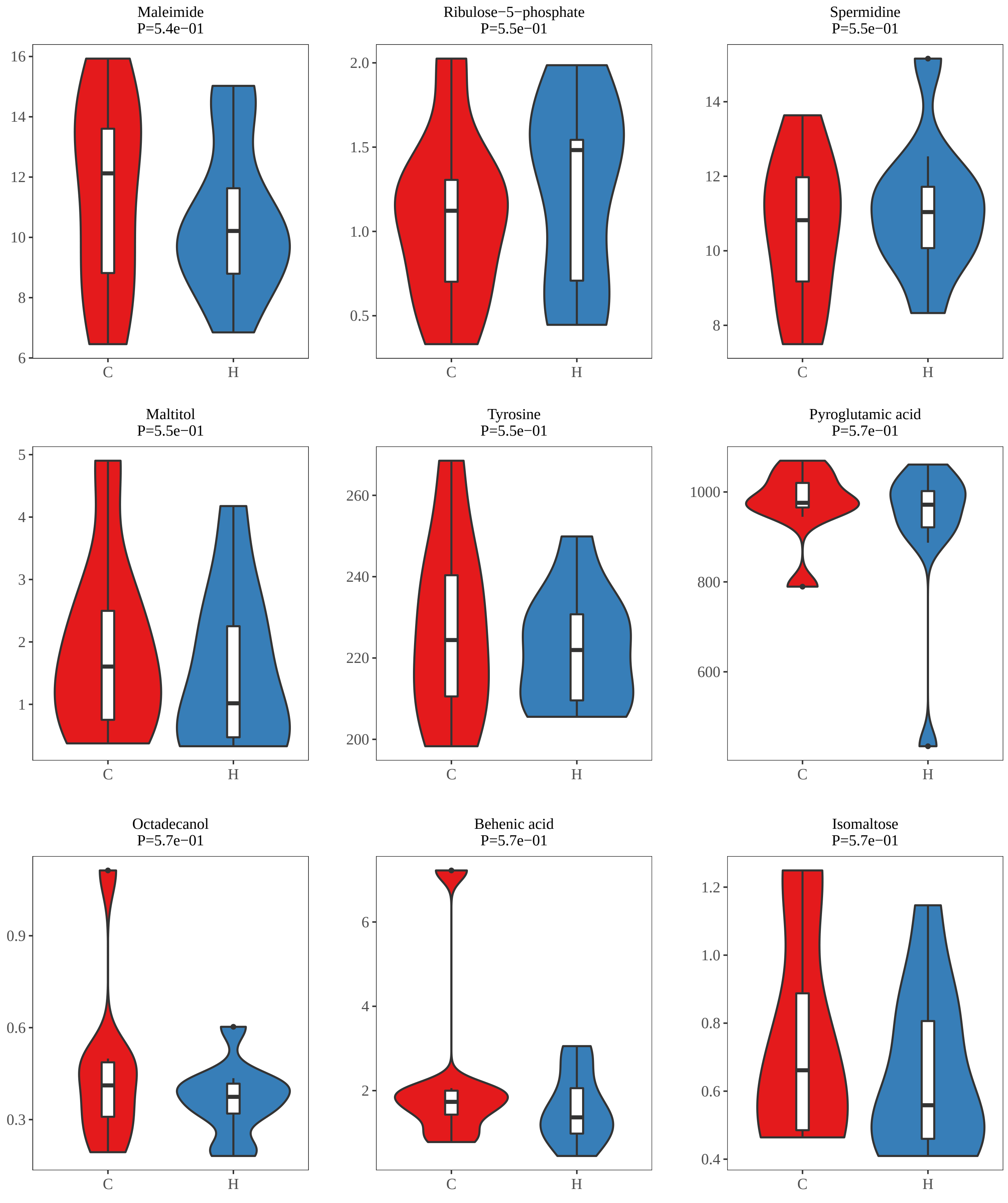

Maleimide
P=5.4e−01
Ribulose−5−phosphate
P=5.5e−01
Spermidine
P=5.5e−01
Maltitol
P=5.5e−01
Tyrosine
P=5.5e−01
Pyroglutamic acid
P=5.7e−01
Octadecanol
P=5.7e−01
Behenic acid
P=5.7e−01
Isomaltose
P=5.7e−01
C
H

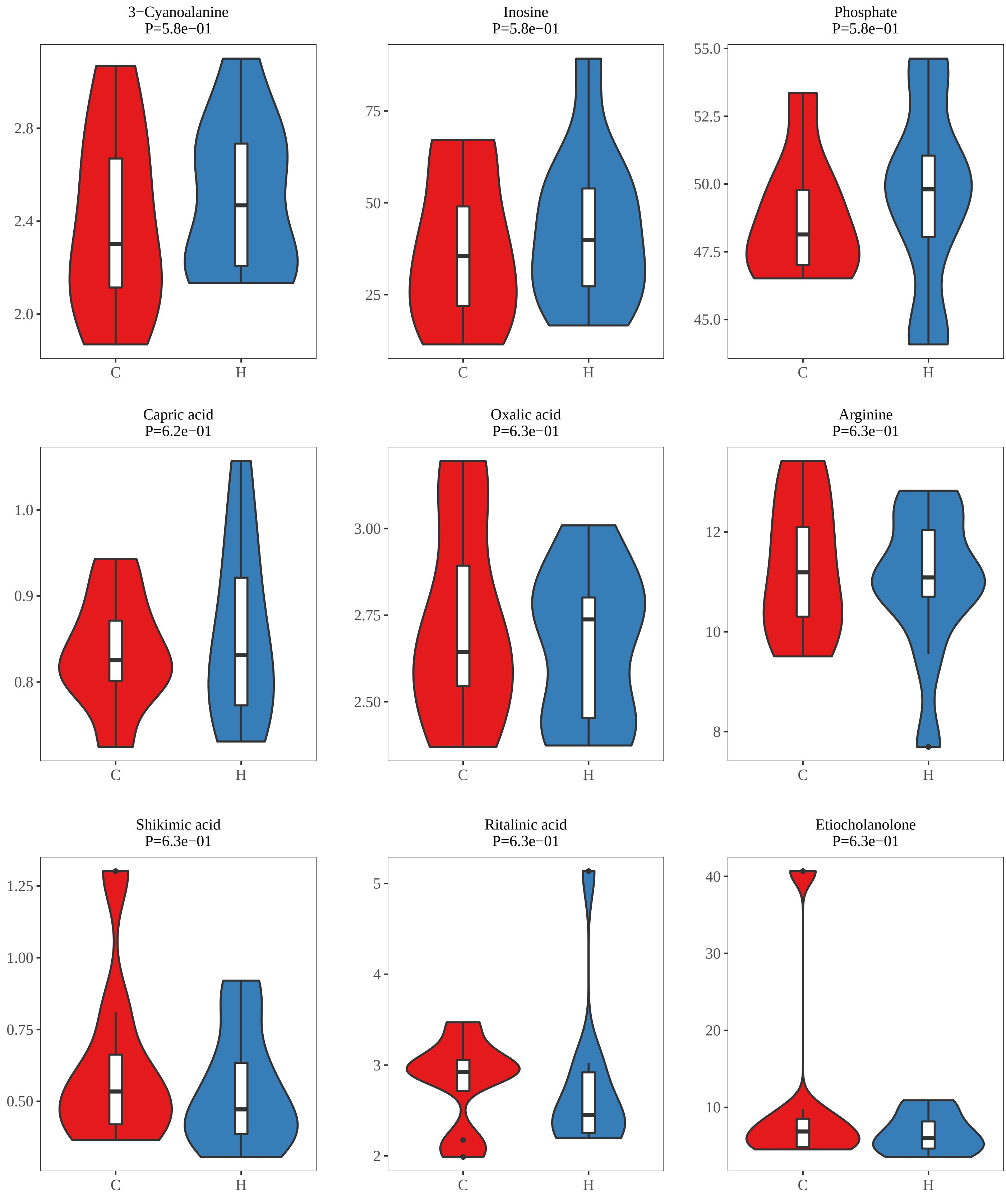
3−Cyanoalanine
P=5.8e−01
Inosine
P=5.8e−01
Phosphate
P=5.8e−01
Capric acid
P=6.2e−01
Oxalic acid
P=6.3e−01
Arginine
P=6.3e−01
Shikimic acid
P=6.3e−01
Ritalinic acid
P=6.3e−01
Etiocholanolone
P=6.3e−01
C
H

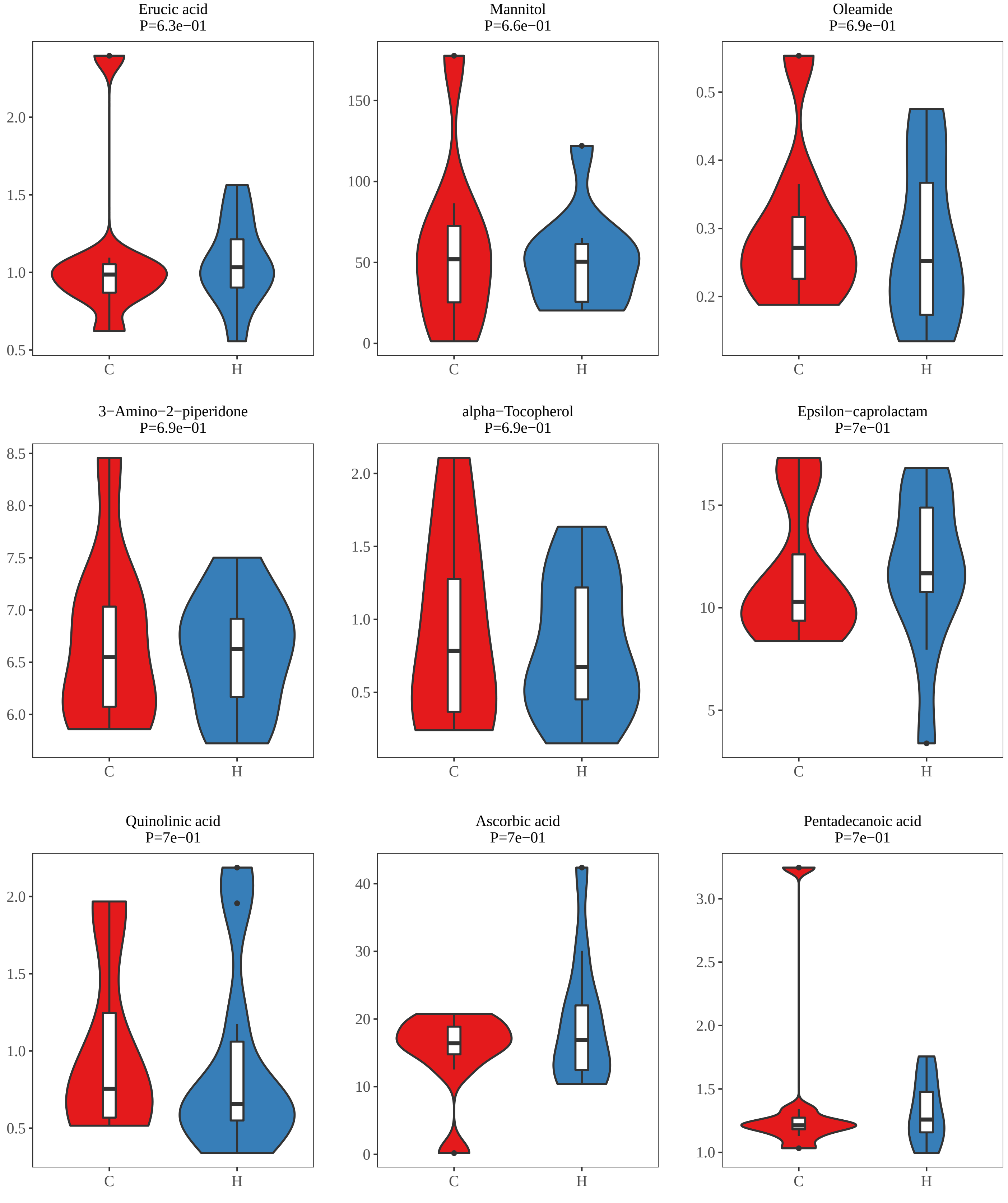
Erucic acid
P=6.3e−01
Mannitol
P=6.6e−01
Oleamide
P=6.9e−01
3−Amino−2−piperidone
P=6.9e−01
alpha−Tocopherol
P=6.9e−01
Epsilon−caprolactam
P=7e−01
Quinolinic acid
P=7e−01
Ascorbic acid
P=7e−01
Pentadecanoic acid
P=7e−01
C
H

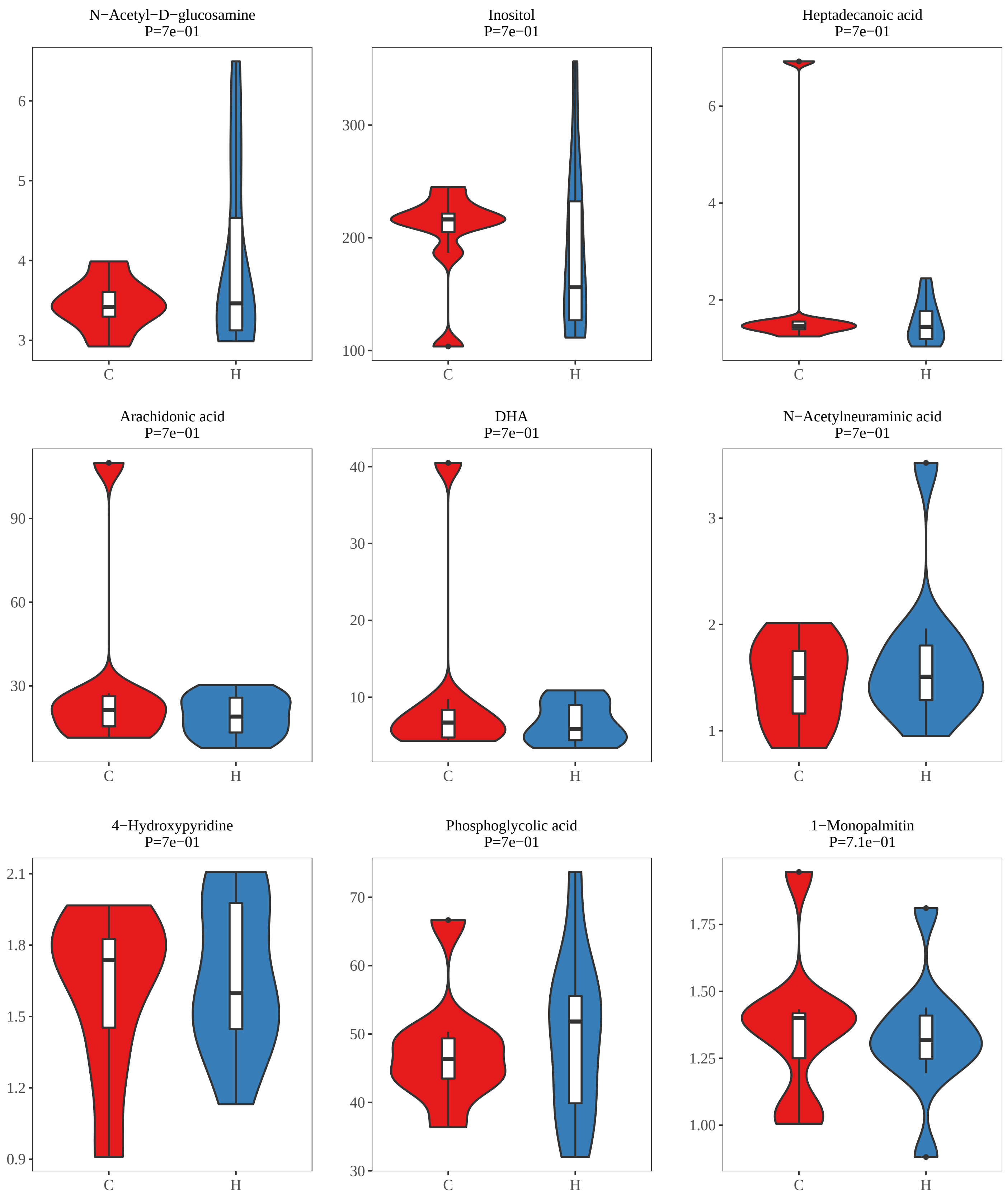
N−Acetyl−D−glucosamine
P=7e−01
Inositol
P=7e−01
Heptadecanoic acid
P=7e−01
Arachidonic acid
P=7e−01
DHA
P=7e−01
N−Acetylneuraminic acid
P=7e−01
4−Hydroxypyridine
P=7e−01
Phosphoglycolic acid
P=7e−01
1−Monopalmitin
P=7.1e−01
C
H

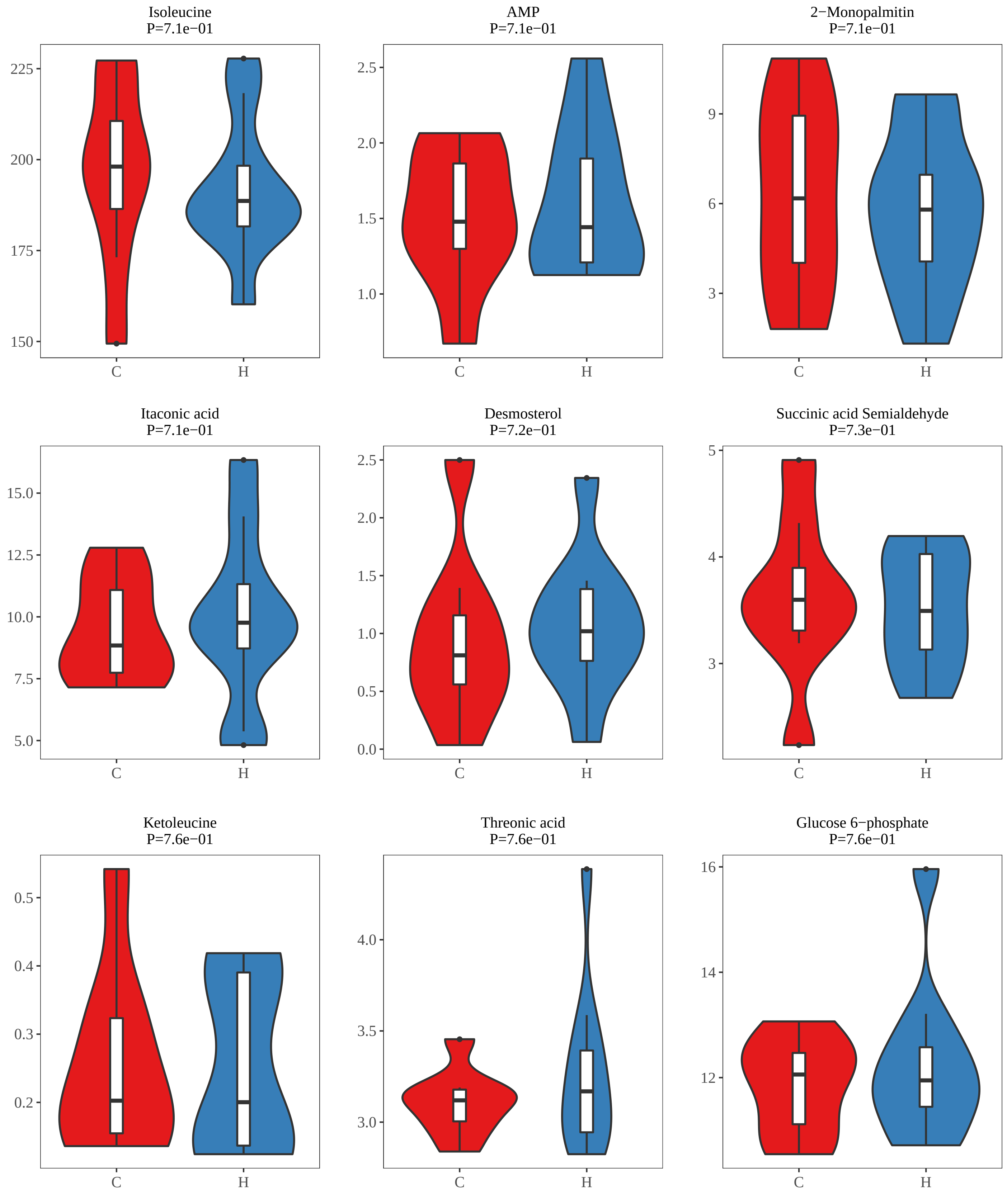

Isoleucine
P=7.1e−01
225
200
175
150
C
H
AMP
P=7.1e−01
2.5
2.0
1.5
1.0
C
H
2−Monopalmitin
P=7.1e−01
9
6
3
C
H
Itaconic acid
P=7.1e−01
15.0
12.5
10.0
7.5
5.0
C
H
Desmosterol
P=7.2e−01
2.5
2.0
1.5
1.0
0.5
0.0
C
H
Succinic acid Semialdehyde
P=7.3e−01
5
4
3
C
H
Ketoleucine
P=7.6e−01
0.5
0.4
0.3
0.2
C
H
Threonic acid
P=7.6e−01
4.0
3.5
3.0
C
H
Glucose 6−phosphate
P=7.6e−01
16
14
12
C
H

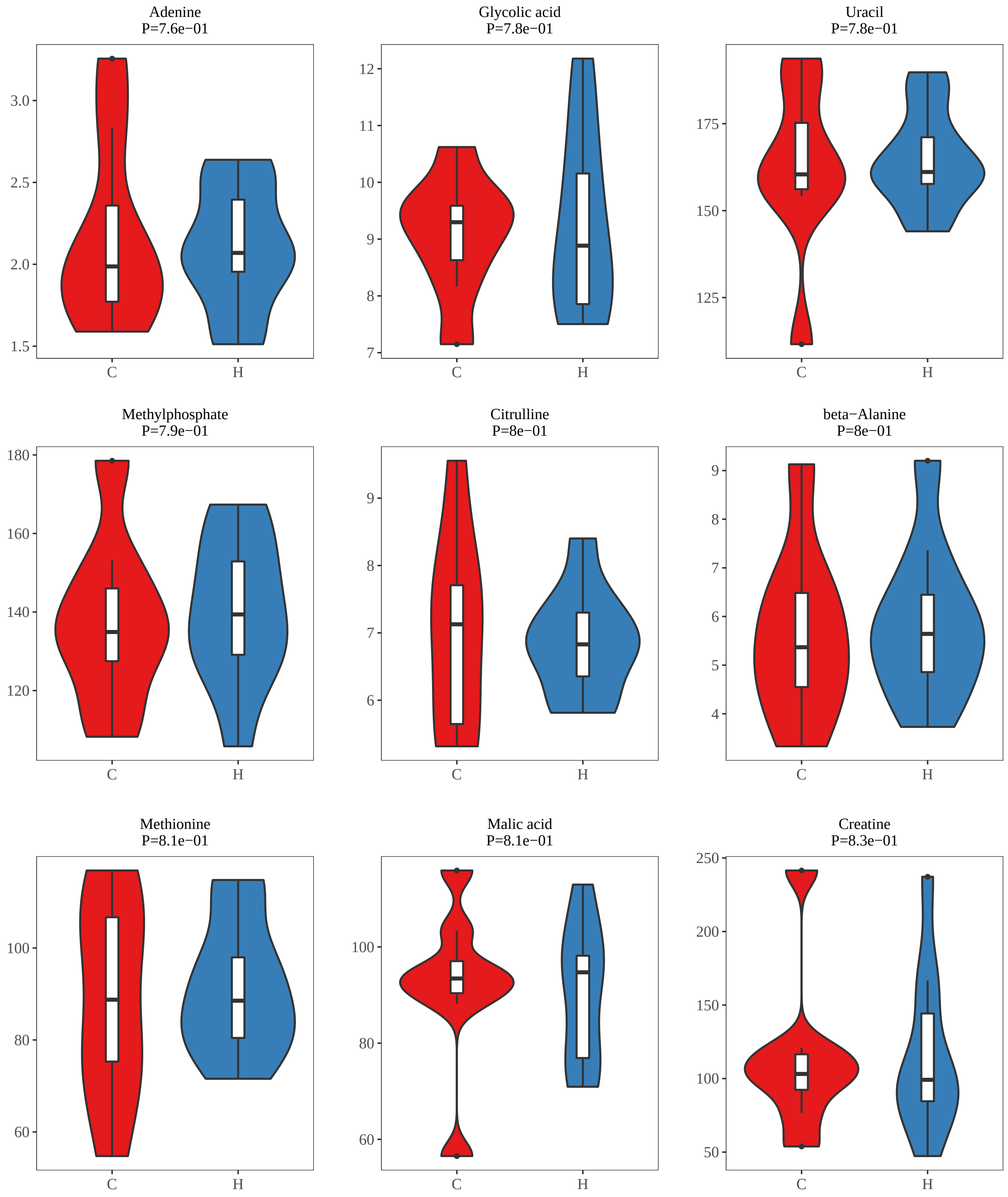

Adenine
P=7.6e−01
Glycolic acid
P=7.8e−01
Uracil
P=7.8e−01
Methylphosphate
P=7.9e−01
Citrulline
P=8e−01
beta−Alanine
P=8e−01
Methionine
P=8.1e−01
Malic acid
P=8.1e−01
Creatine
P=8.3e−01
C
H

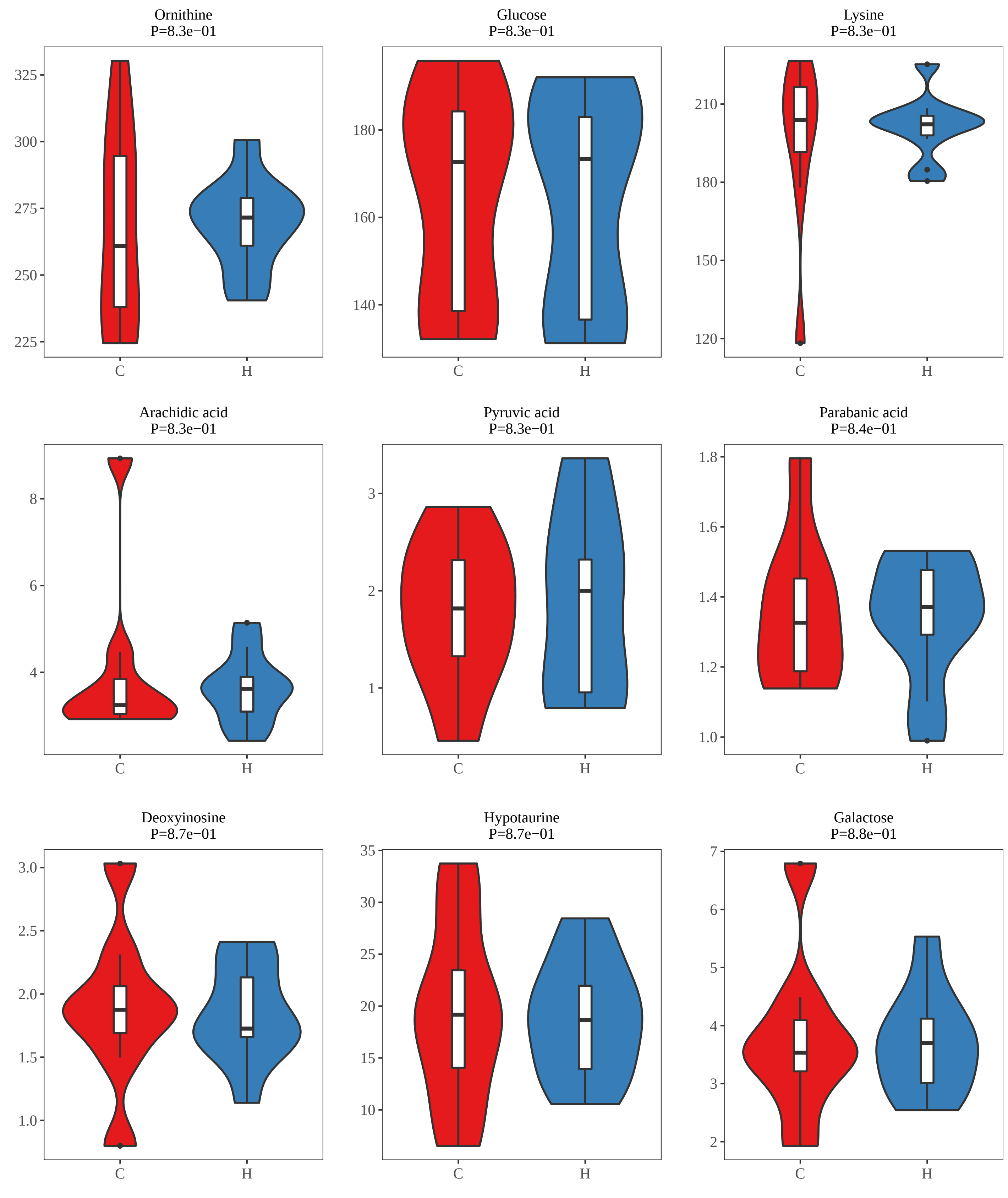

Ornithine
P=8.3e−01
325
300
275
250
225
C
H
Glucose
P=8.3e−01
180
160
140
C
H
Lysine
P=8.3e−01
210
180
150
120
C
H
Arachidic acid
P=8.3e−01
8
6
4
C
H
Pyruvic acid
P=8.3e−01
3
2
1
C
H
Parabanic acid
P=8.4e−01
1.8
1.6
1.4
1.2
1.0
C
H
Deoxyinosine
P=8.7e−01
3.0
2.5
2.0
1.5
1.0
C
H
Hypotaurine
P=8.7e−01
35
30
25
20
15
10
C
H
Galactose
P=8.8e−01
7
6
5
4
3
2
C
H

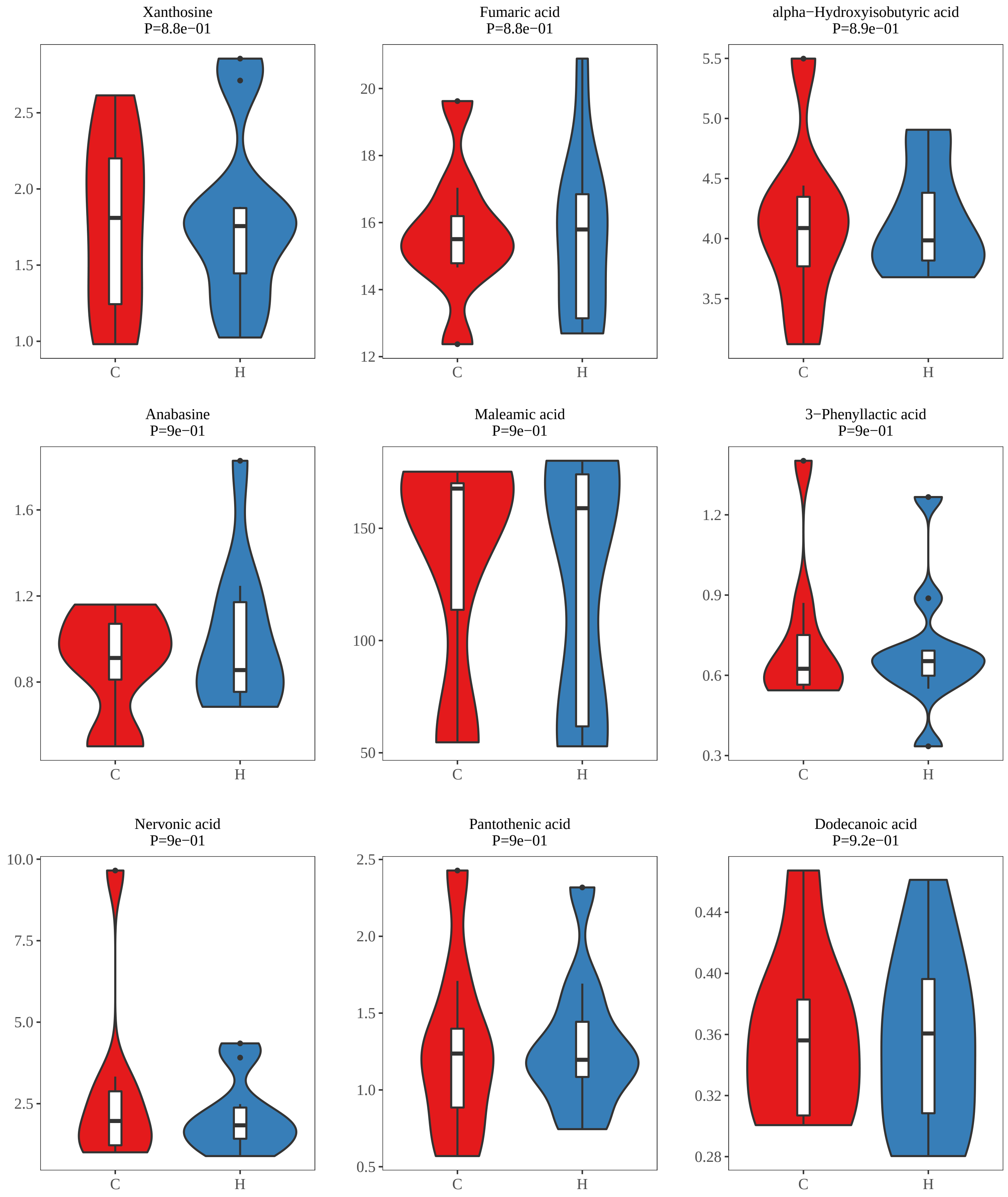
Xanthosine
P=8.8e−01
Fumaric acid
P=8.8e−01
alpha−Hydroxyisobutyric acid
P=8.9e−01
Anabasine
P=9e−01
Maleamic acid
P=9e−01
3−Phenyllactic acid
P=9e−01
Nervonic acid
P=9e−01
Pantothenic acid
P=9e−01
Dodecanoic acid
P=9.2e−01
C
H

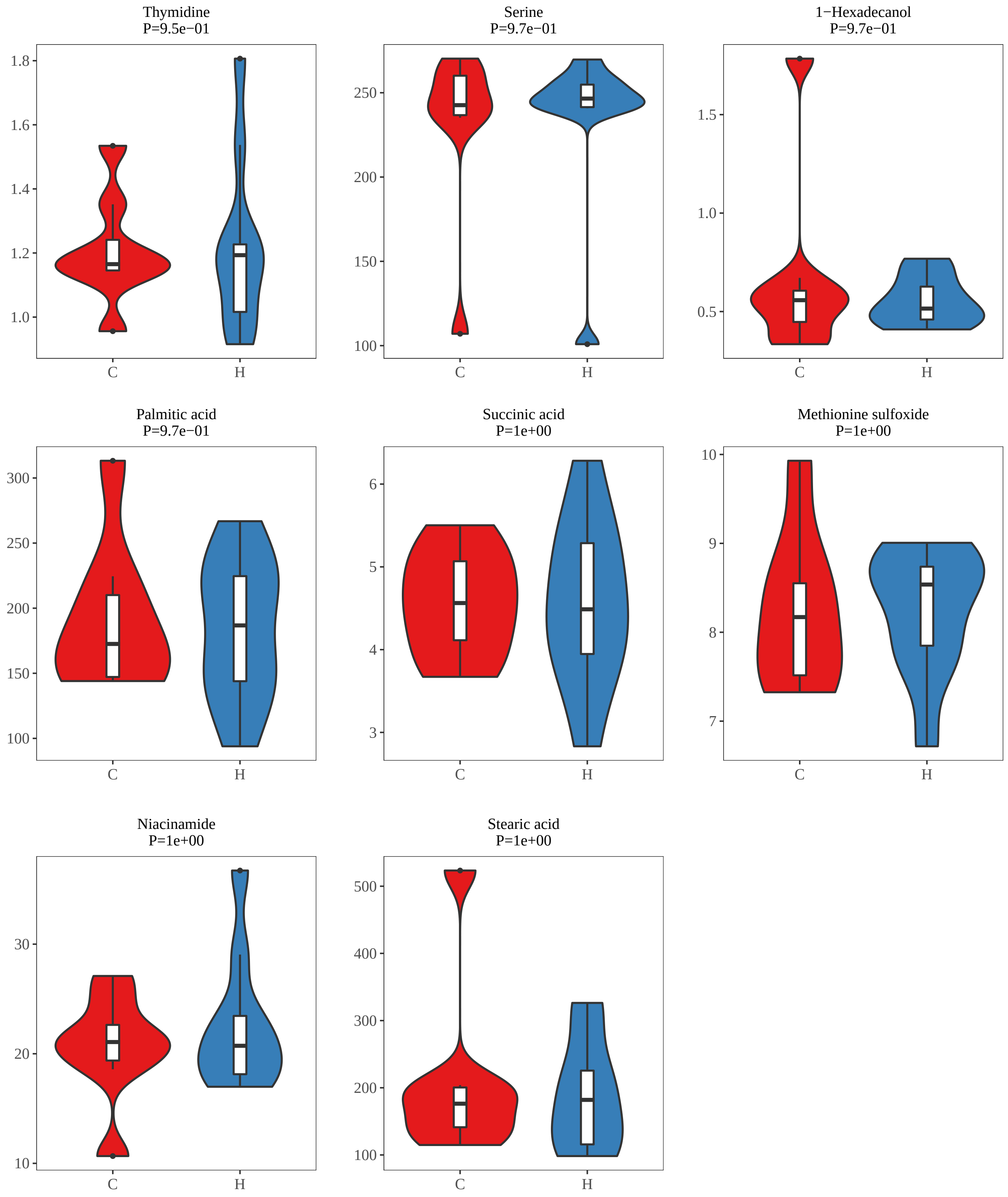
Thymidine
P=9.5e−01
1.8
1.6
1.4
1.2
1.0
C
H
Serine
P=9.7e−01
250
200
150
100
C
H
1−Hexadecanol
P=9.7e−01
1.5
1.0
0.5
C
H
Palmitic acid
P=9.7e−01
300
250
200
150
100
C
H
Succinic acid
P=1e+00
6
5
4
3
C
H
Methionine sulfoxide
P=1e+00
10
9
8
7
C
H
Niacinamide
P=1e+00
30
20
10
C
H
Stearic acid
P=1e+00
500
400
300
200
100
C
H
