# Supplementary material for: Yiai Fuzheng decoction inhibits triple-negative breast cancer by remodeling the immune microenvironment
Source: Front Immunol. 2025 Sep 30;16:1615631. doi: 10.3389/fimmu.2025.1615631 (PMC12518410; doi:10.3389/fimmu.2025.1615631)
Supplement: Supplementary file 2 [file DataSheet1.zip › Supplementary File 2/Treatment/C_vs_D/06_Potential_Biomarkers/Markers_Vioplot.pdf]

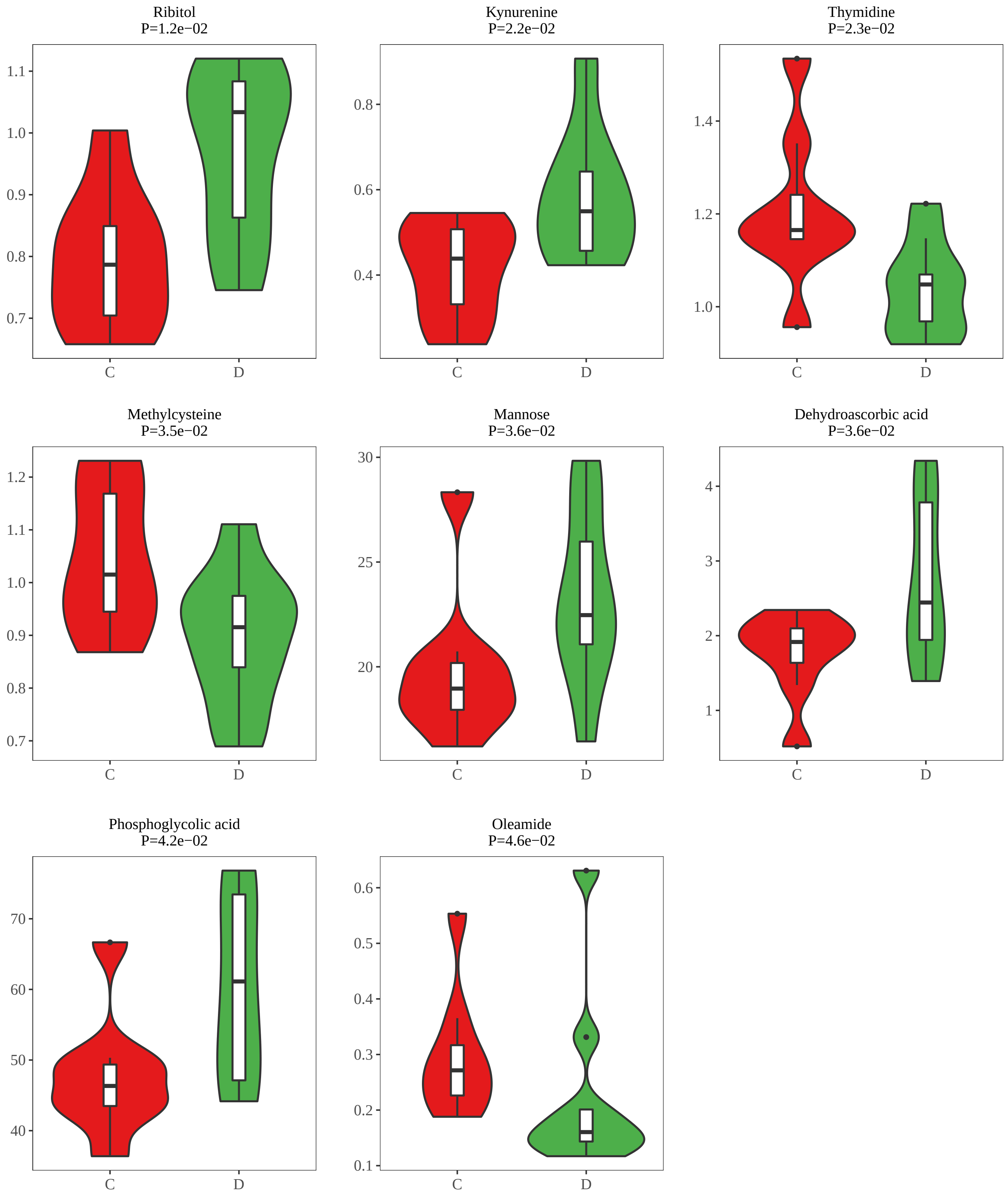
Ribitol
P=1.2e−02
Kynurenine
P=2.2e−02
Thymidine
P=2.3e−02
Methylcysteine
P=3.5e−02
Mannose
P=3.6e−02
Dehydroascorbic acid
P=3.6e−02
Phosphoglycolic acid
P=4.2e−02
Oleamide
P=4.6e−02
C
D
