# Supplementary material for: Yiai Fuzheng decoction inhibits triple-negative breast cancer by remodeling the immune microenvironment
Source: Front Immunol. 2025 Sep 30;16:1615631. doi: 10.3389/fimmu.2025.1615631 (PMC12518410; doi:10.3389/fimmu.2025.1615631)
Supplement: Supplementary file 2 [file DataSheet1.zip › Supplementary File 2/Treatment/C_vs_D/06_Potential_Biomarkers/Markers_Boxplot_with_Points.pdf]

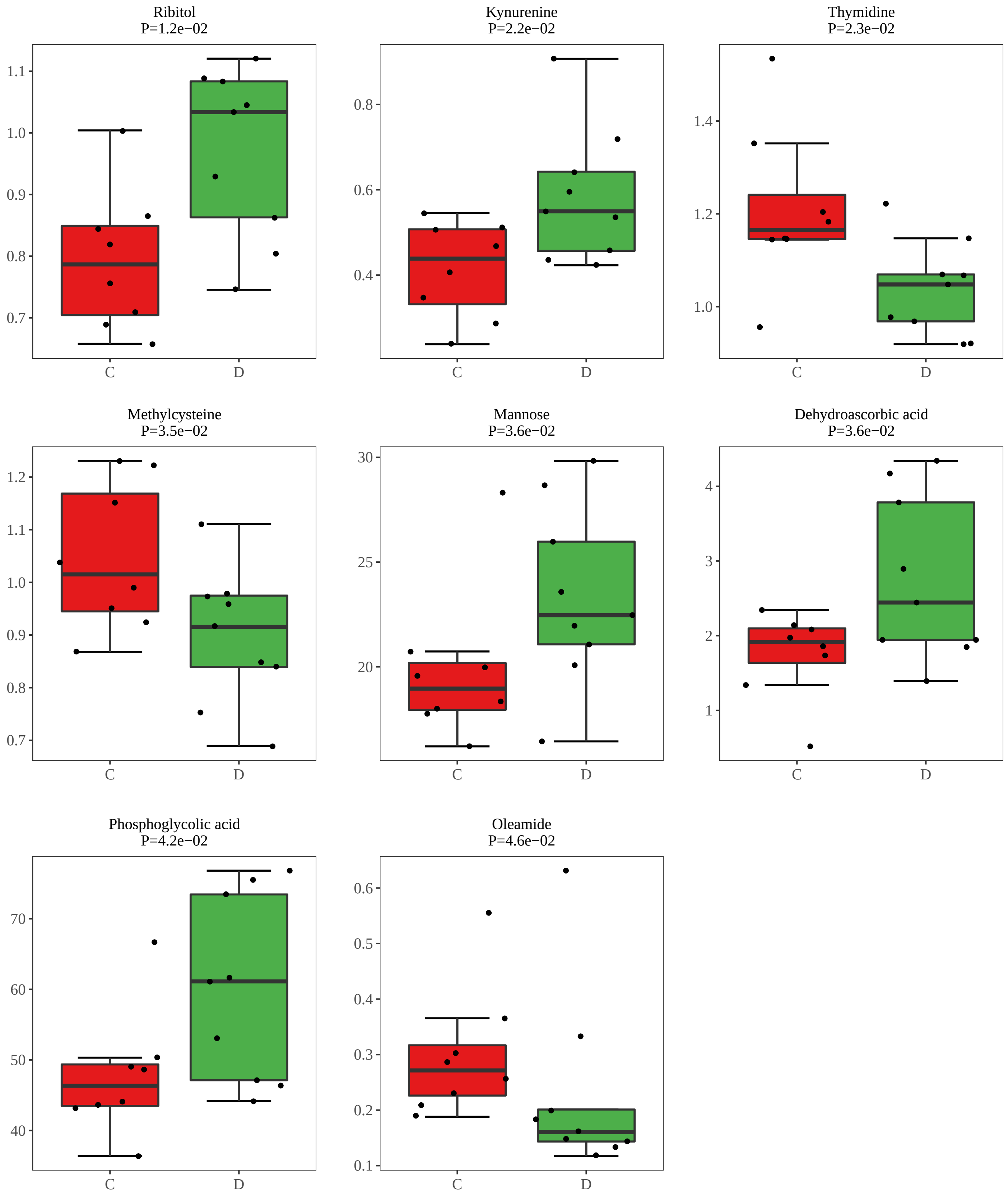

Ribitol
P=1.2e−02
1.1
1.0
0.9
0.8
0.7
C
D
Kynurenine
P=2.2e−02
0.8
0.6
0.4
C
D
Thymidine
P=2.3e−02
1.4
1.2
1.0
C
D
Methylcysteine
P=3.5e−02
1.2
1.1
1.0
0.9
0.8
0.7
C
D
Mannose
P=3.6e−02
30
25
20
C
D
Dehydroascorbic acid
P=3.6e−02
4
3
2
1
C
D
Phosphoglycolic acid
P=4.2e−02
70
60
50
40
C
D
Oleamide
P=4.6e−02
0.6
0.5
0.4
0.3
0.2
0.1
C
D
