# Supplementary material for: Yiai Fuzheng decoction inhibits triple-negative breast cancer by remodeling the immune microenvironment
Source: Front Immunol. 2025 Sep 30;16:1615631. doi: 10.3389/fimmu.2025.1615631 (PMC12518410; doi:10.3389/fimmu.2025.1615631)
Supplement: Supplementary file 2 [file DataSheet1.zip › Supplementary File 2/Treatment/C_vs_D/05_Univariate_Analysis/AllMet_Boxplot.pdf]

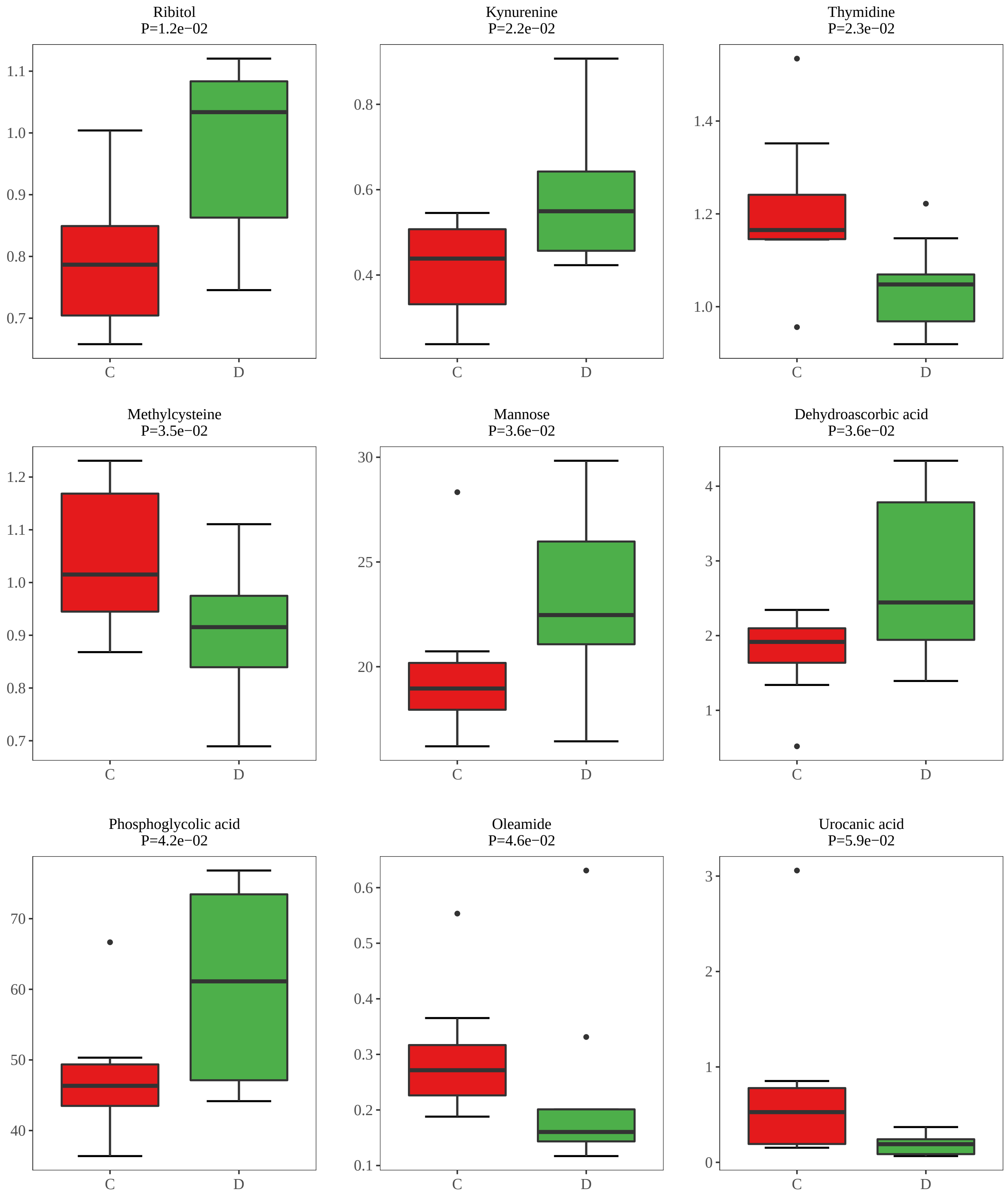
Ribitol
P=1.2e−02
1.1
1.0
0.9
0.8
0.7
C
D
Kynurenine
P=2.2e−02
0.8
0.6
0.4
C
D
Thymidine
P=2.3e−02
1.4
1.2
1.0
C
D
Methylcysteine
P=3.5e−02
1.2
1.1
1.0
0.9
0.8
0.7
C
D
Mannose
P=3.6e−02
30
25
20
C
D
Dehydroascorbic acid
P=3.6e−02
4
3
2
1
C
D
Phosphoglycolic acid
P=4.2e−02
70
60
50
40
C
D
Oleamide
P=4.6e−02
0.6
0.5
0.4
0.3
0.2
0.1
C
D
Urocanic acid
P=5.9e−02
3
2
1
0
C
D

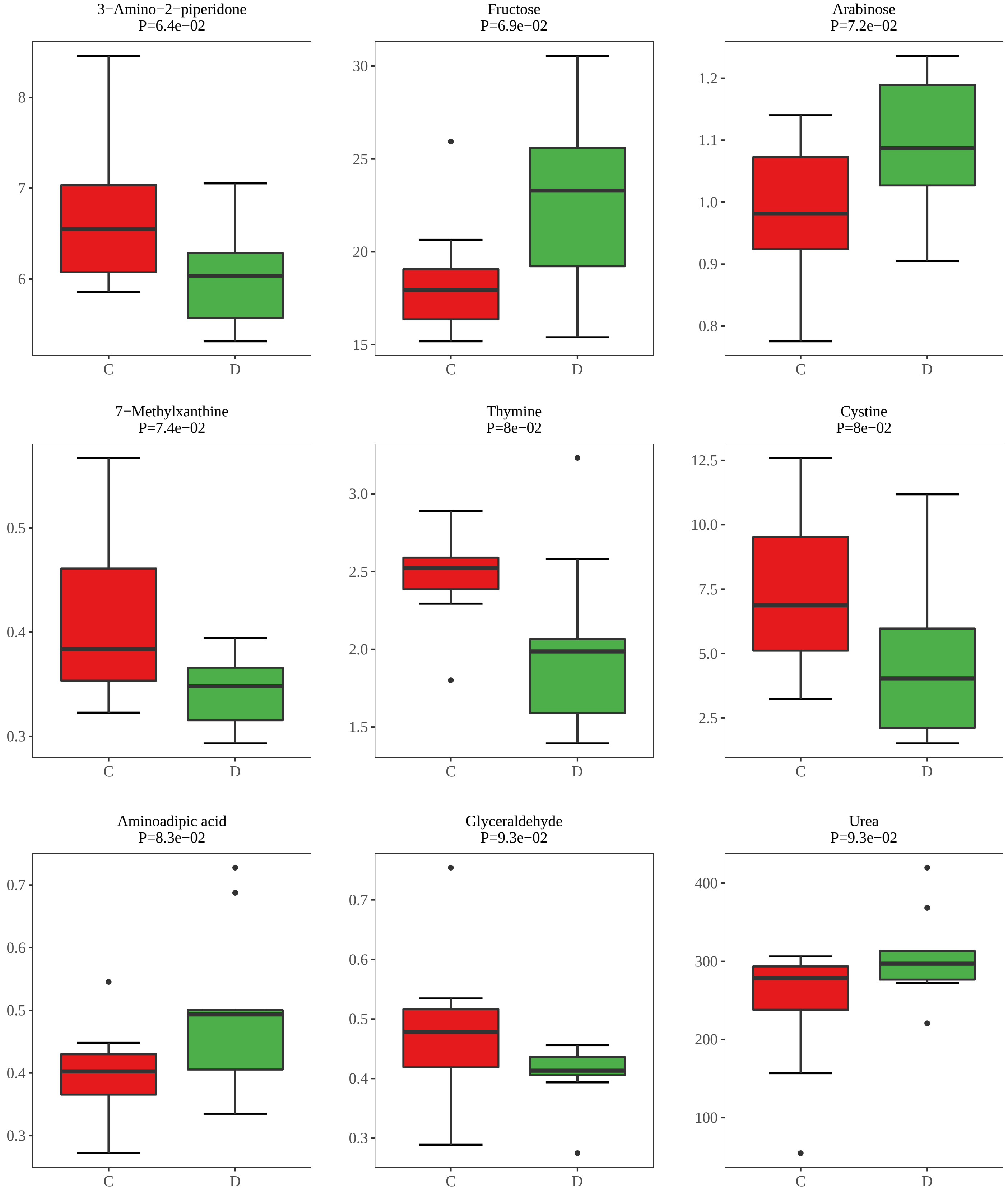
3−Amino−2−piperidone
P=6.4e−02
Fructose
P=6.9e−02
Arabinose
P=7.2e−02
7−Methylxanthine
P=7.4e−02
Thymine
P=8e−02
Cystine
P=8e−02
Aminoadipic acid
P=8.3e−02
Glyceraldehyde
P=9.3e−02
Urea
P=9.3e−02
C
D

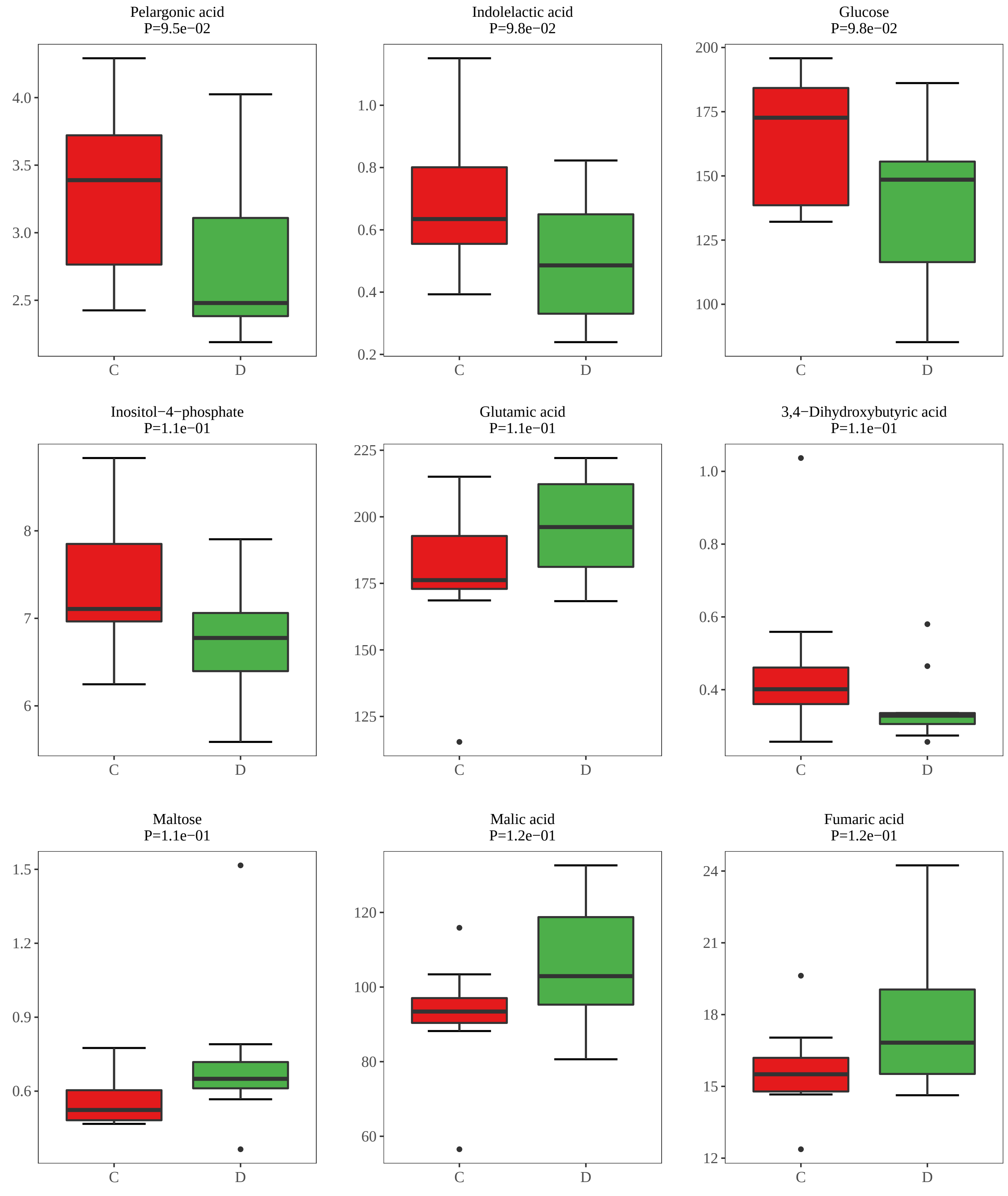
Pelargonic acid
P=9.5e−02
Indolelactic acid
P=9.8e−02
Glucose
P=9.8e−02
Inositol−4−phosphate
P=1.1e−01
Glutamic acid
P=1.1e−01
3,4−Dihydroxybutyric acid
P=1.1e−01
Maltose
P=1.1e−01
Malic acid
P=1.2e−01
Fumaric acid
P=1.2e−01
C
D

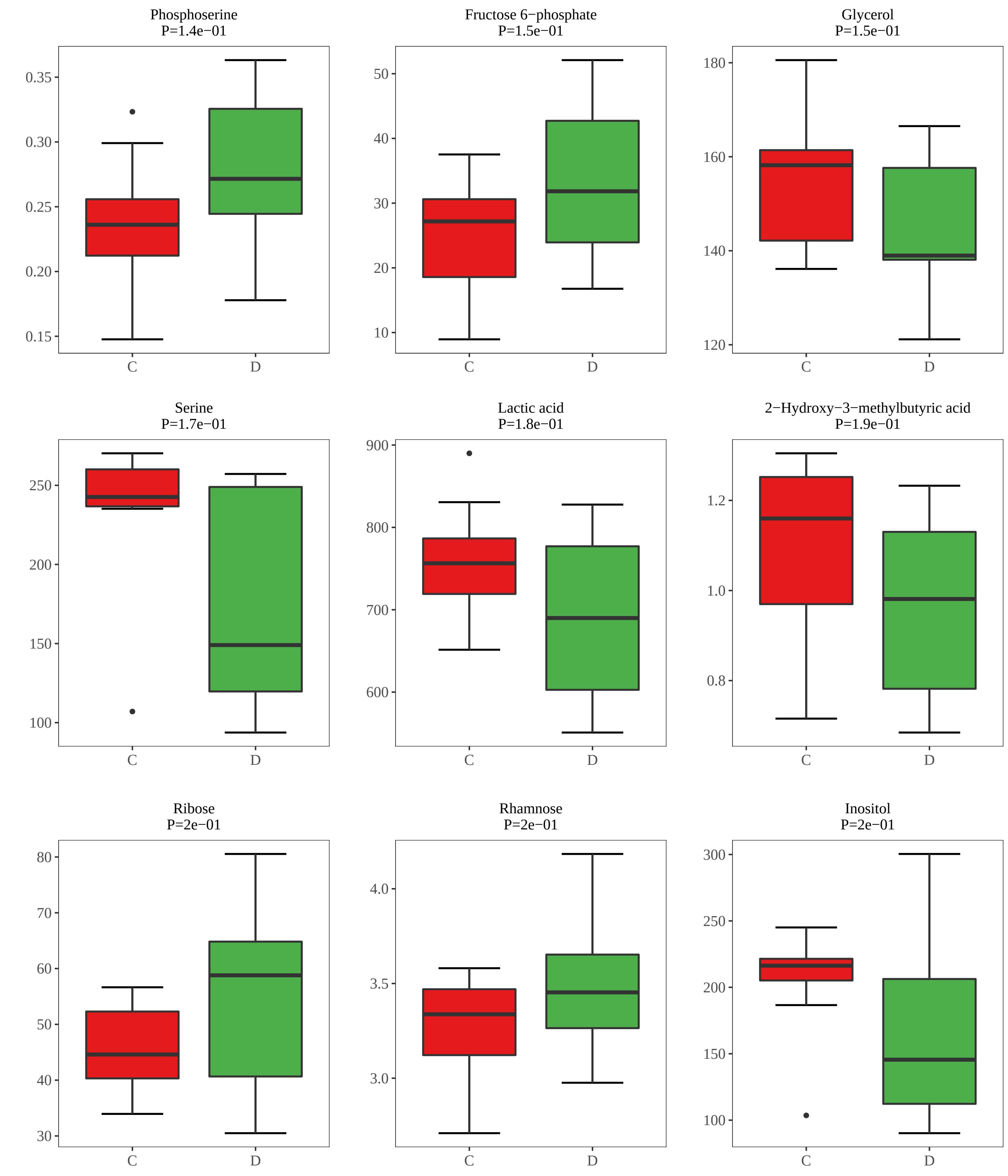
Phosphoserine
P=1.4e−01
0.35
0.30
0.25
0.20
0.15
C
D
Fructose 6−phosphate
P=1.5e−01
50
40
30
20
10
C
D
Glycerol
P=1.5e−01
180
160
140
120
C
D
Serine
P=1.7e−01
250
200
150
100
C
D
Lactic acid
P=1.8e−01
900
800
700
600
C
D
2−Hydroxy−3−methylbutyric acid
P=1.9e−01
1.2
1.0
0.8
C
D
Ribose
P=2e−01
80
70
60
50
40
30
C
D
Rhamnose
P=2e−01
4.0
3.5
3.0
C
D
Inositol
P=2e−01
300
250
200
150
100
C
D

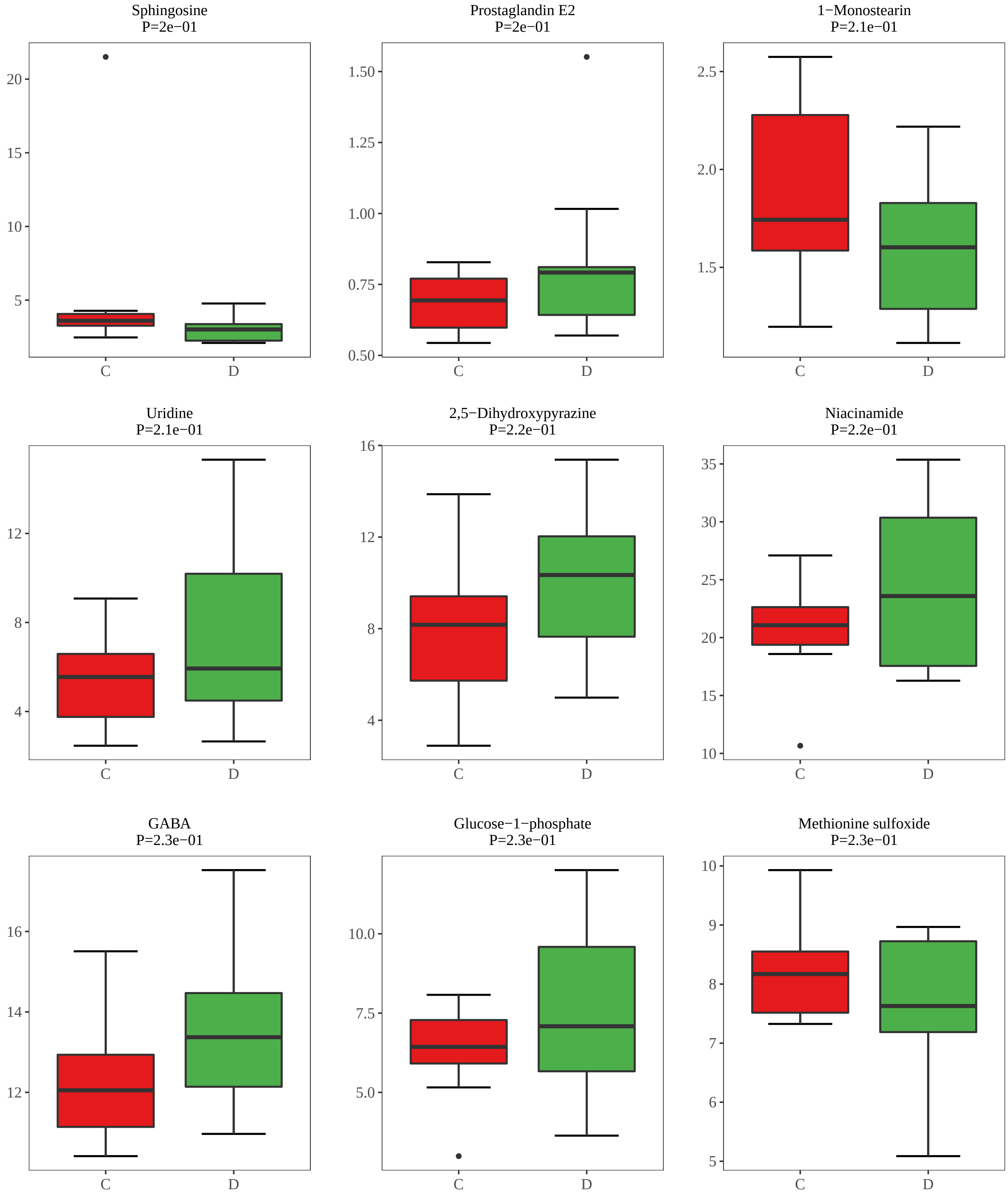

Sphingosine
P=2e−01
20
15
10
5
C
D
Prostaglandin E2
P=2e−01
1.50
1.25
1.00
0.75
0.50
C
D
1−Monostearin
P=2.1e−01
2.5
2.0
1.5
C
D
Uridine
P=2.1e−01
12
8
4
C
D
2,5−Dihydroxypyrazine
P=2.2e−01
16
12
8
4
C
D
Niacinamide
P=2.2e−01
35
30
25
20
15
10
C
D
GABA
P=2.3e−01
16
14
12
C
D
Glucose−1−phosphate
P=2.3e−01
10.0
7.5
5.0
C
D
Methionine sulfoxide
P=2.3e−01
10
9
8
7
6
5
C
D

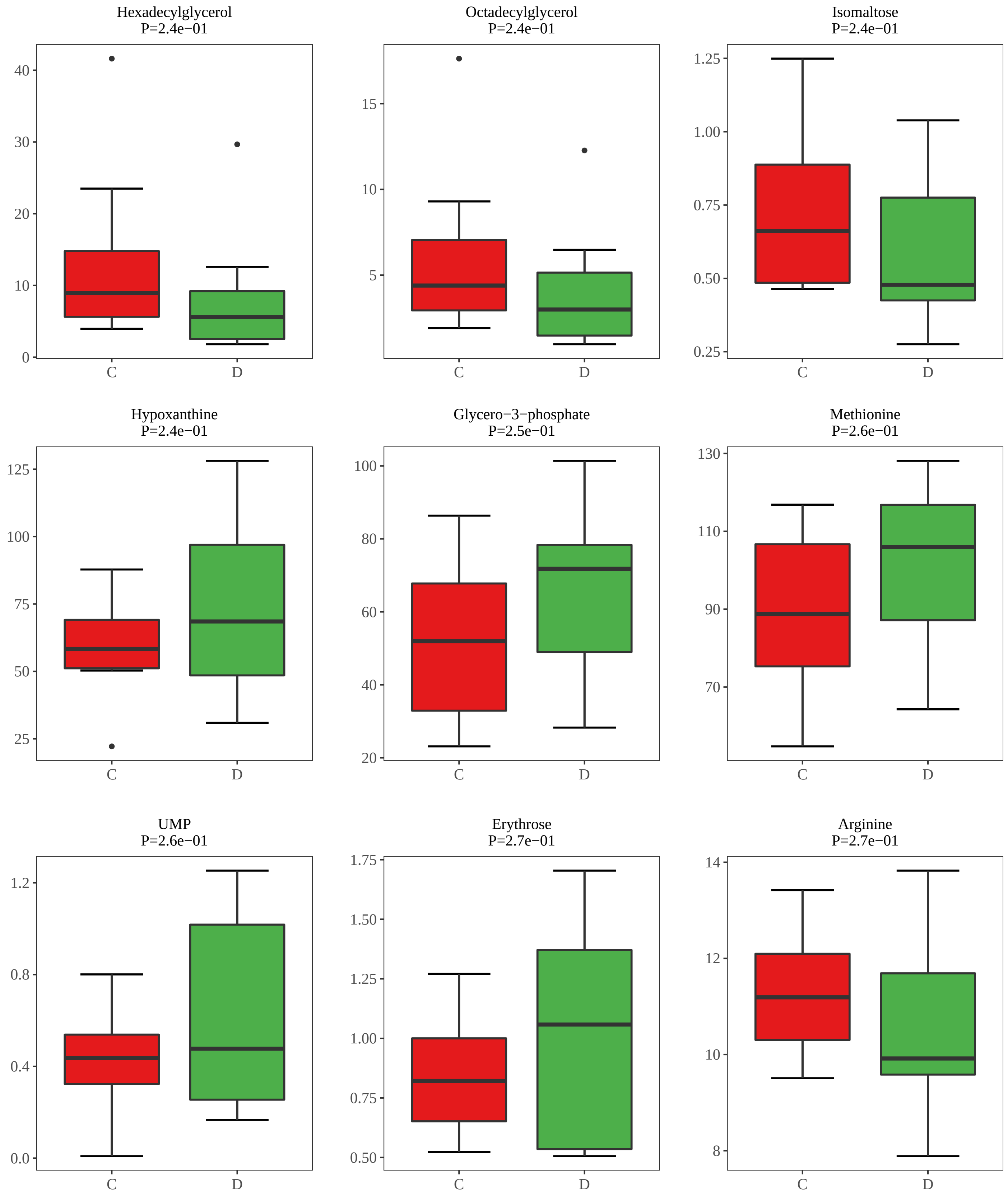
Hexadecylglycerol
P=2.4e−01
0
10
20
30
40
C
D
Octadecylglycerol
P=2.4e−01
5
10
15
C
D
Isomaltose
P=2.4e−01
0.25
0.50
0.75
1.00
1.25
C
D
Hypoxanthine
P=2.4e−01
25
50
75
100
125
C
D
Glycero−3−phosphate
P=2.5e−01
20
40
60
80
100
C
D
Methionine
P=2.6e−01
70
90
110
130
C
D
UMP
P=2.6e−01
0.0
0.4
0.8
1.2
C
D
Erythrose
P=2.7e−01
0.50
0.75
1.00
1.25
1.50
1.75
C
D
Arginine
P=2.7e−01
8
10
12
14
C
D

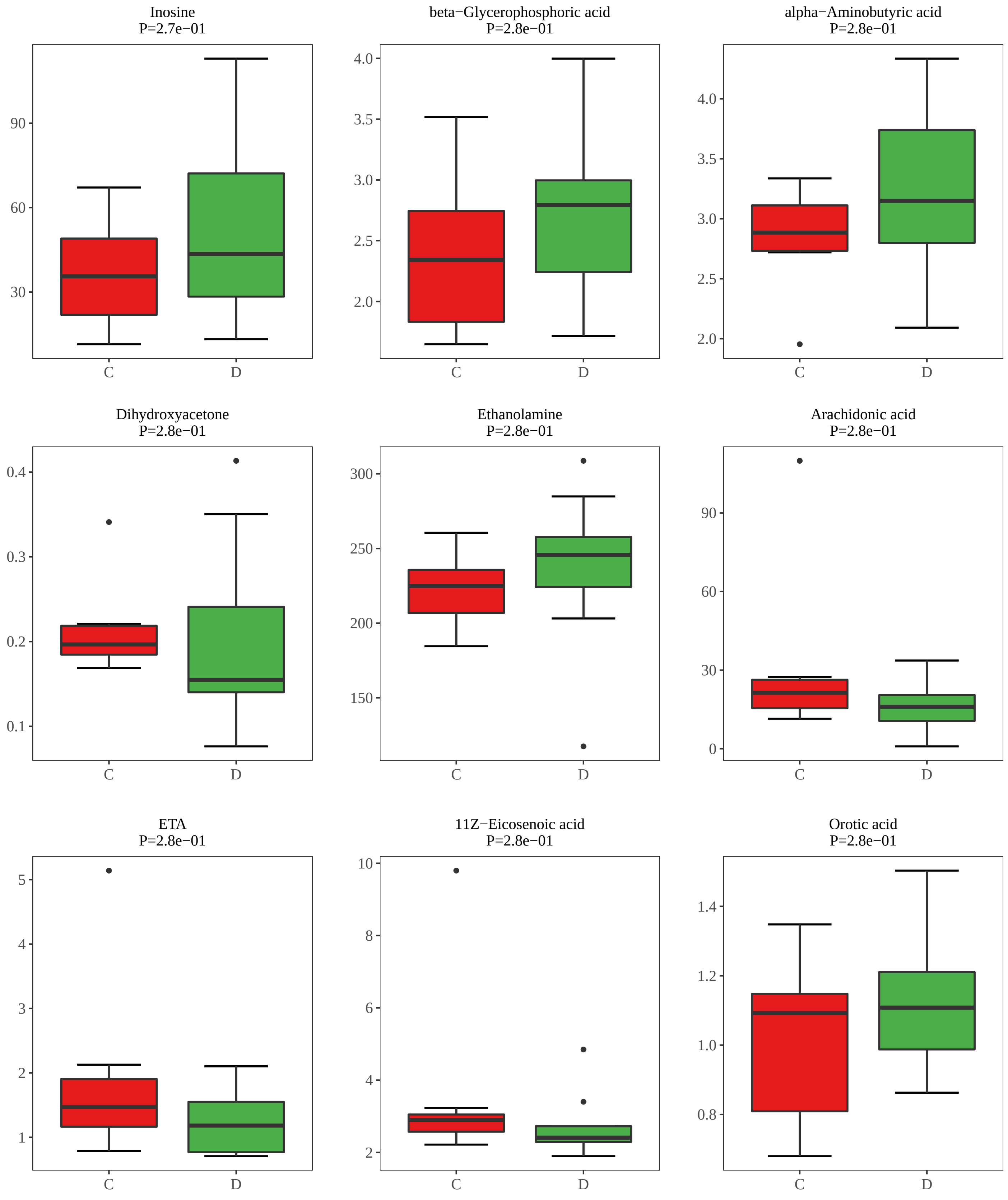
Inosine
P=2.7e−01
beta−Glycerophosphoric acid
P=2.8e−01
alpha−Aminobutyric acid
P=2.8e−01
Dihydroxyacetone
P=2.8e−01
Ethanolamine
P=2.8e−01
Arachidonic acid
P=2.8e−01
ETA
P=2.8e−01
11Z−Eicosenoic acid
P=2.8e−01
Orotic acid
P=2.8e−01
C
D

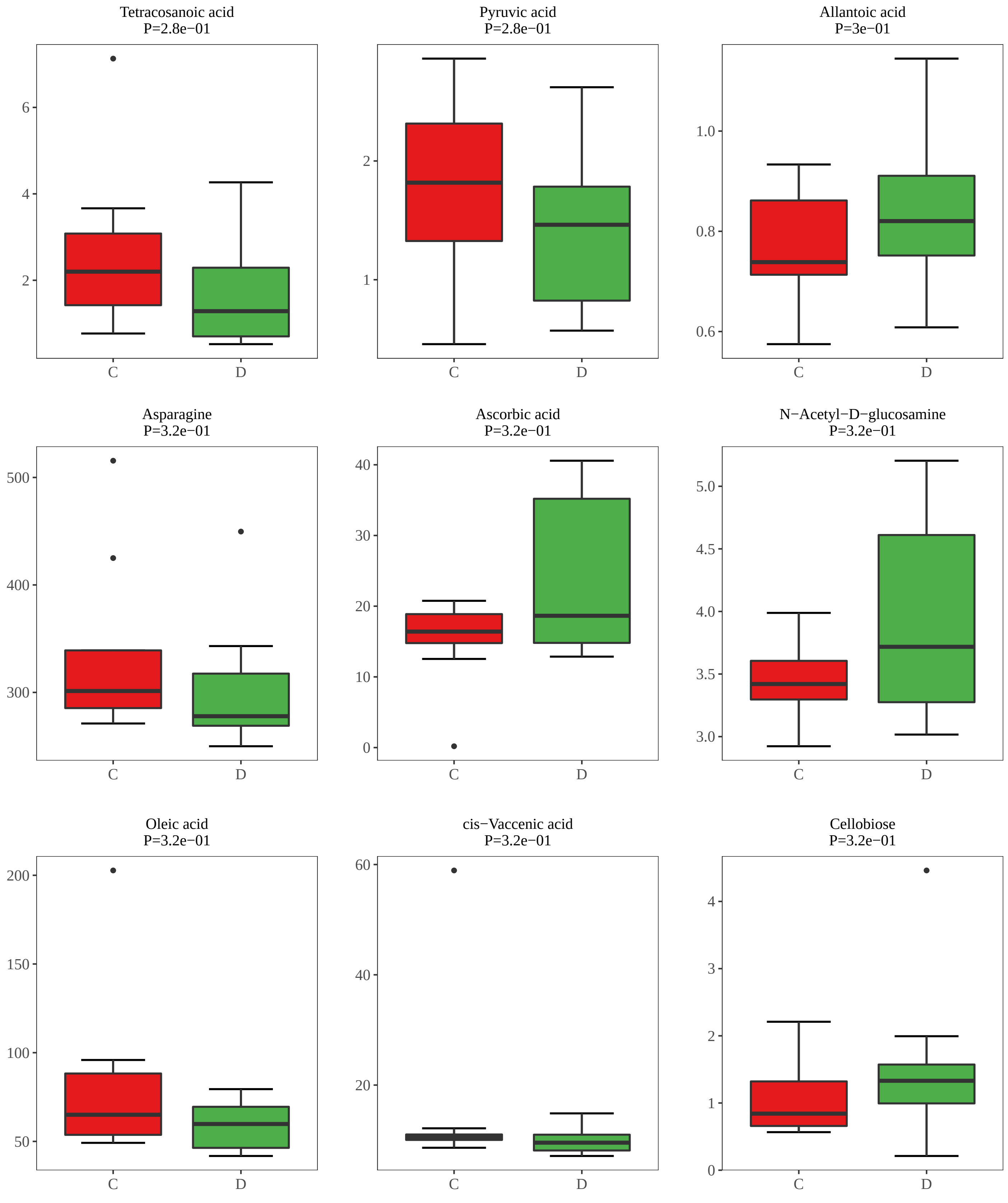

Tetracosanoic acid
P=2.8e−01
Pyruvic acid
P=2.8e−01
Allantoic acid
P=3e−01
Asparagine
P=3.2e−01
Ascorbic acid
P=3.2e−01
N−Acetyl−D−glucosamine
P=3.2e−01
Oleic acid
P=3.2e−01
cis−Vaccenic acid
P=3.2e−01
Cellobiose
P=3.2e−01
C
D

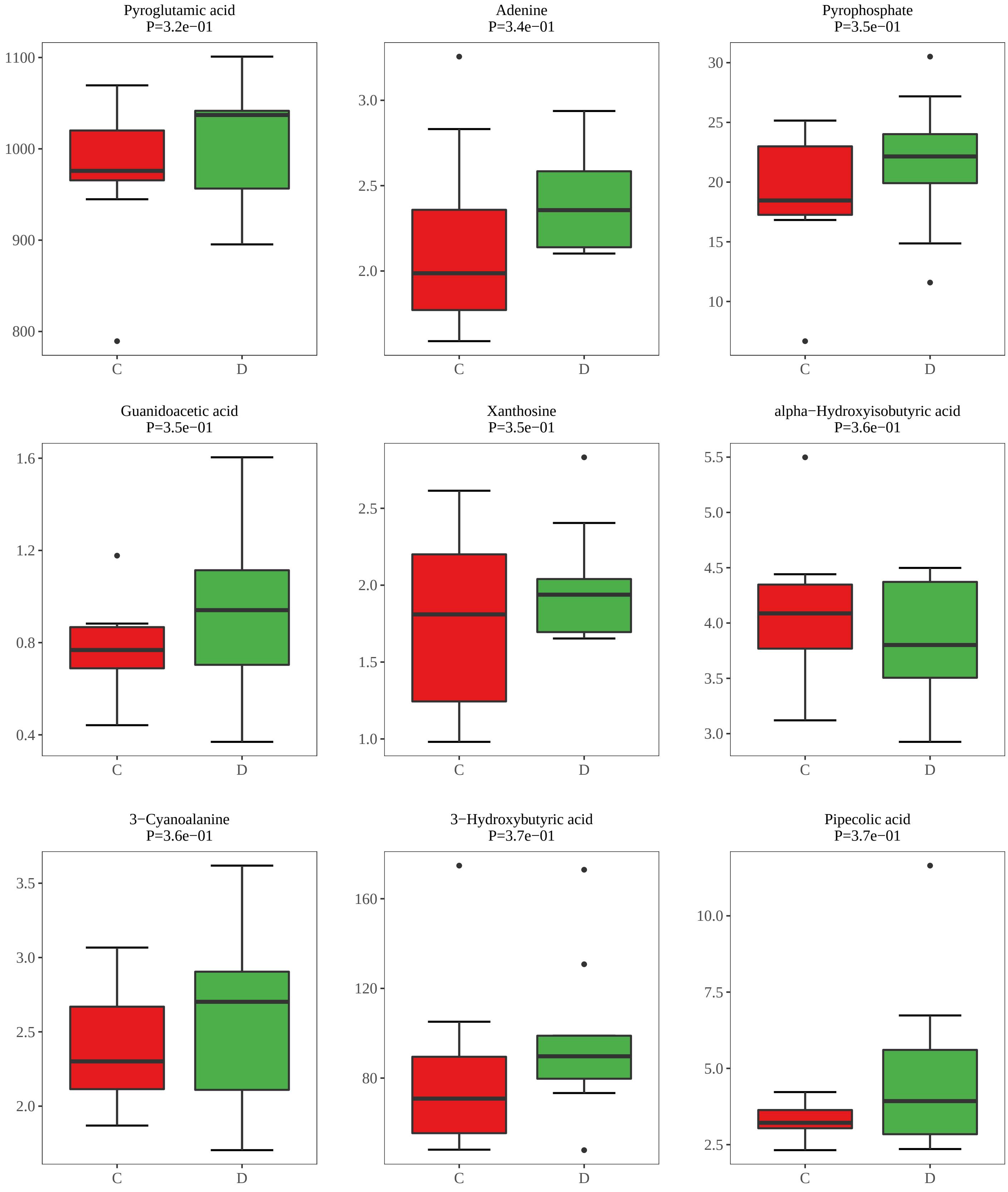
Pyroglutamic acid
P=3.2e−01
1100
1000
900
800
C
D
Adenine
P=3.4e−01
3.0
2.5
2.0
C
D
Pyrophosphate
P=3.5e−01
30
25
20
15
10
C
D
Guanidoacetic acid
P=3.5e−01
1.6
1.2
0.8
0.4
C
D
Xanthosine
P=3.5e−01
2.5
2.0
1.5
1.0
C
D
alpha−Hydroxyisobutyric acid
P=3.6e−01
5.5
5.0
4.5
4.0
3.5
3.0
C
D
3−Cyanoalanine
P=3.6e−01
3.5
3.0
2.5
2.0
C
D
3−Hydroxybutyric acid
P=3.7e−01
160
120
80
C
D
Pipecolic acid
P=3.7e−01
10.0
7.5
5.0
2.5
C
D

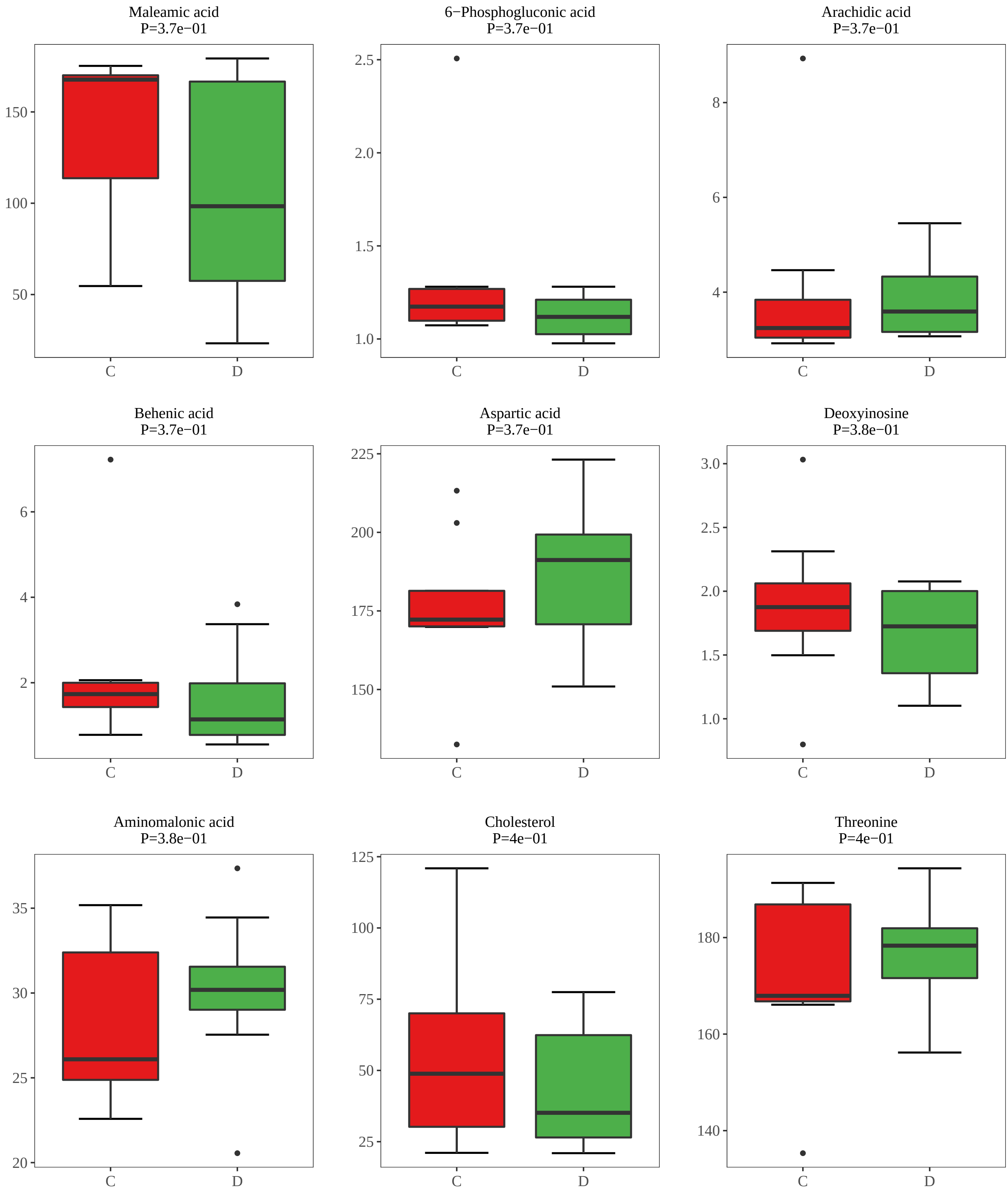

Maleamic acid
P=3.7e−01
6−Phosphogluconic acid
P=3.7e−01
Arachidic acid
P=3.7e−01
Behenic acid
P=3.7e−01
Aspartic acid
P=3.7e−01
Deoxyinosine
P=3.8e−01
Aminomalonic acid
P=3.8e−01
Cholesterol
P=4e−01
Threonine
P=4e−01
C
D

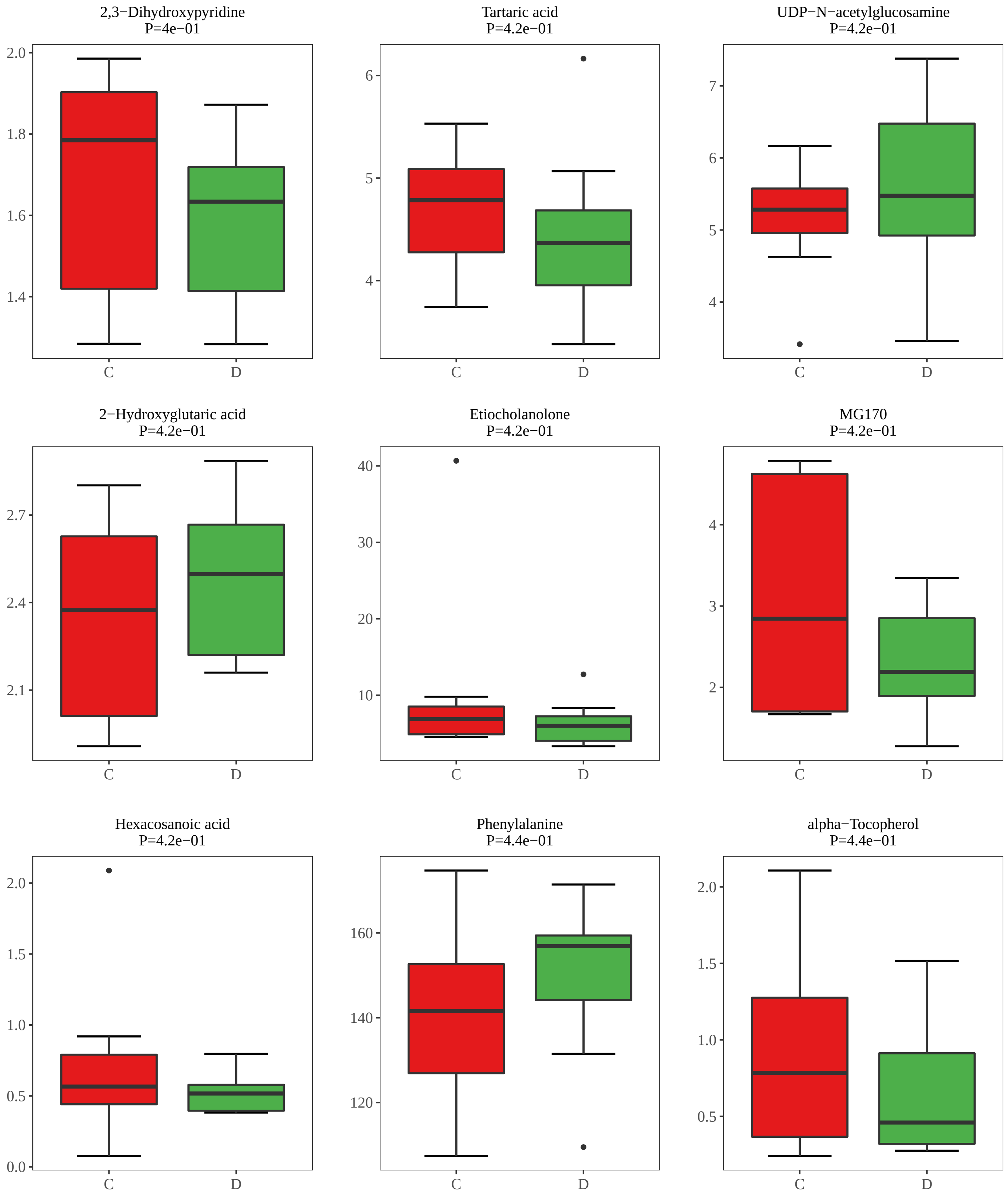

2,3−Dihydroxypyridine
P=4e−01
2.0
1.8
1.6
1.4
C
D
Tartaric acid
P=4.2e−01
6
5
4
C
D
UDP−N−acetylglucosamine
P=4.2e−01
7
6
5
4
C
D
2−Hydroxyglutaric acid
P=4.2e−01
2.7
2.4
2.1
C
D
Etiocholanolone
P=4.2e−01
40
30
20
10
C
D
MG170
P=4.2e−01
4
3
2
C
D
Hexacosanoic acid
P=4.2e−01
2.0
1.5
1.0
0.5
0.0
C
D
Phenylalanine
P=4.4e−01
160
140
120
C
D
alpha−Tocopherol
P=4.4e−01
2.0
1.5
1.0
0.5
C
D

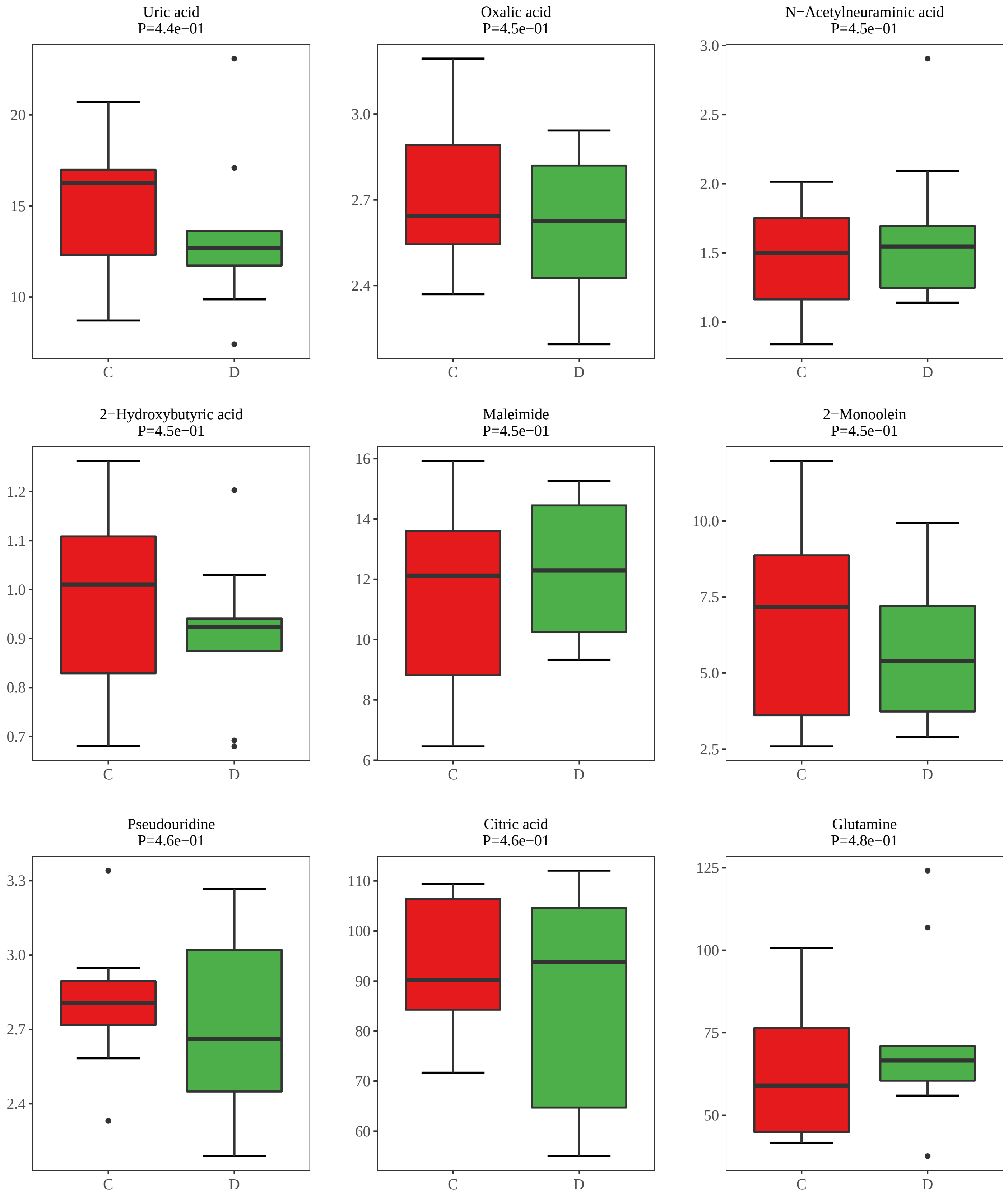

Uric acid
P=4.4e−01
Oxalic acid
P=4.5e−01
N−Acetylneuraminic acid
P=4.5e−01
2−Hydroxybutyric acid
P=4.5e−01
Maleimide
P=4.5e−01
2−Monoolein
P=4.5e−01
Pseudouridine
P=4.6e−01
Citric acid
P=4.6e−01
Glutamine
P=4.8e−01
C
D

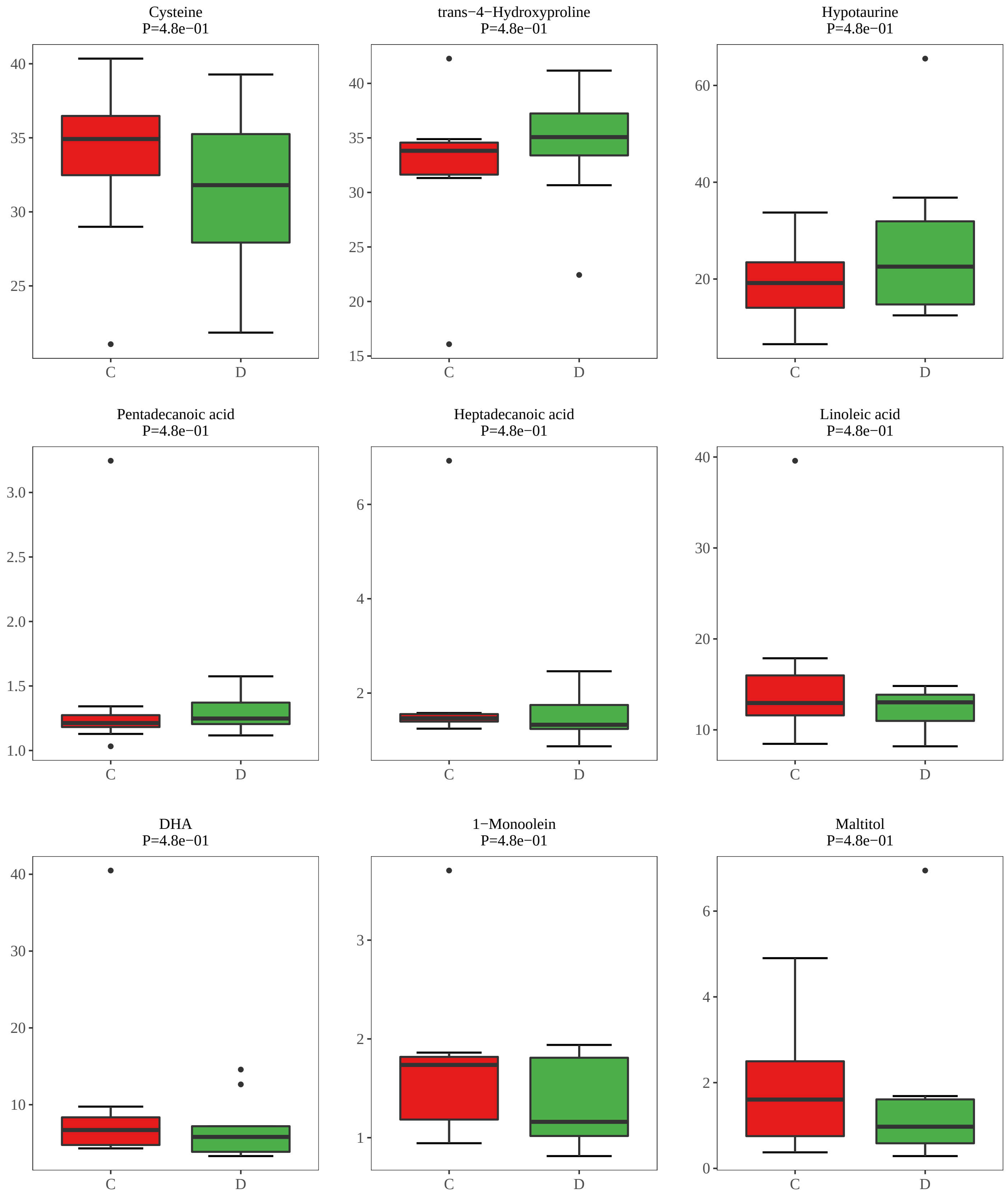

Cysteine
P=4.8e−01
trans−4−Hydroxyproline
P=4.8e−01
Hypotaurine
P=4.8e−01
Pentadecanoic acid
P=4.8e−01
Heptadecanoic acid
P=4.8e−01
Linoleic acid
P=4.8e−01
DHA
P=4.8e−01
1−Monoolein
P=4.8e−01
Maltitol
P=4.8e−01
C
D

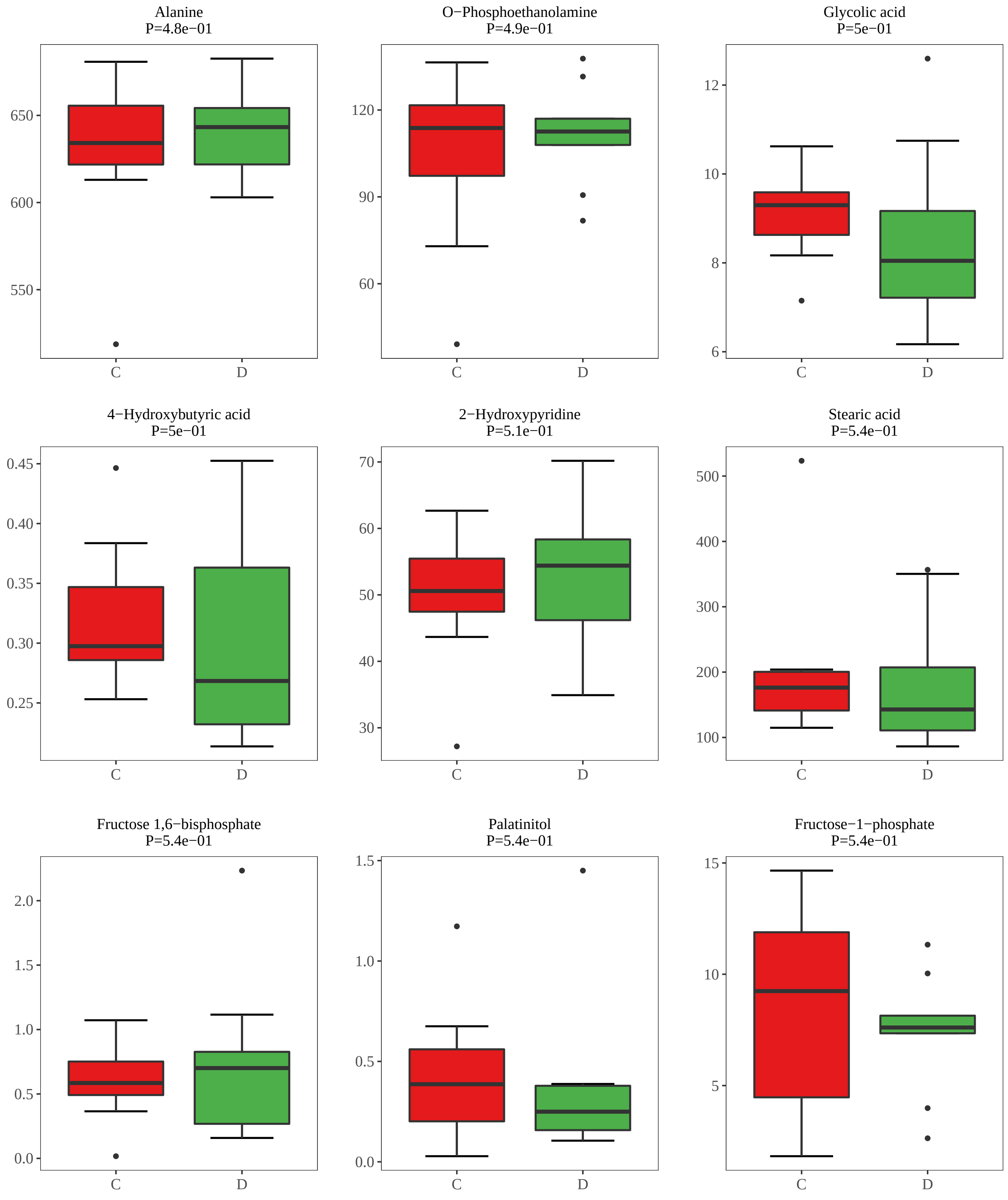

Alanine
P=4.8e−01
O−Phosphoethanolamine
P=4.9e−01
Glycolic acid
P=5e−01
4−Hydroxybutyric acid
P=5e−01
2−Hydroxypyridine
P=5.1e−01
Stearic acid
P=5.4e−01
Fructose 1,6−bisphosphate
P=5.4e−01
Palatinitol
P=5.4e−01
Fructose−1−phosphate
P=5.4e−01
C
D

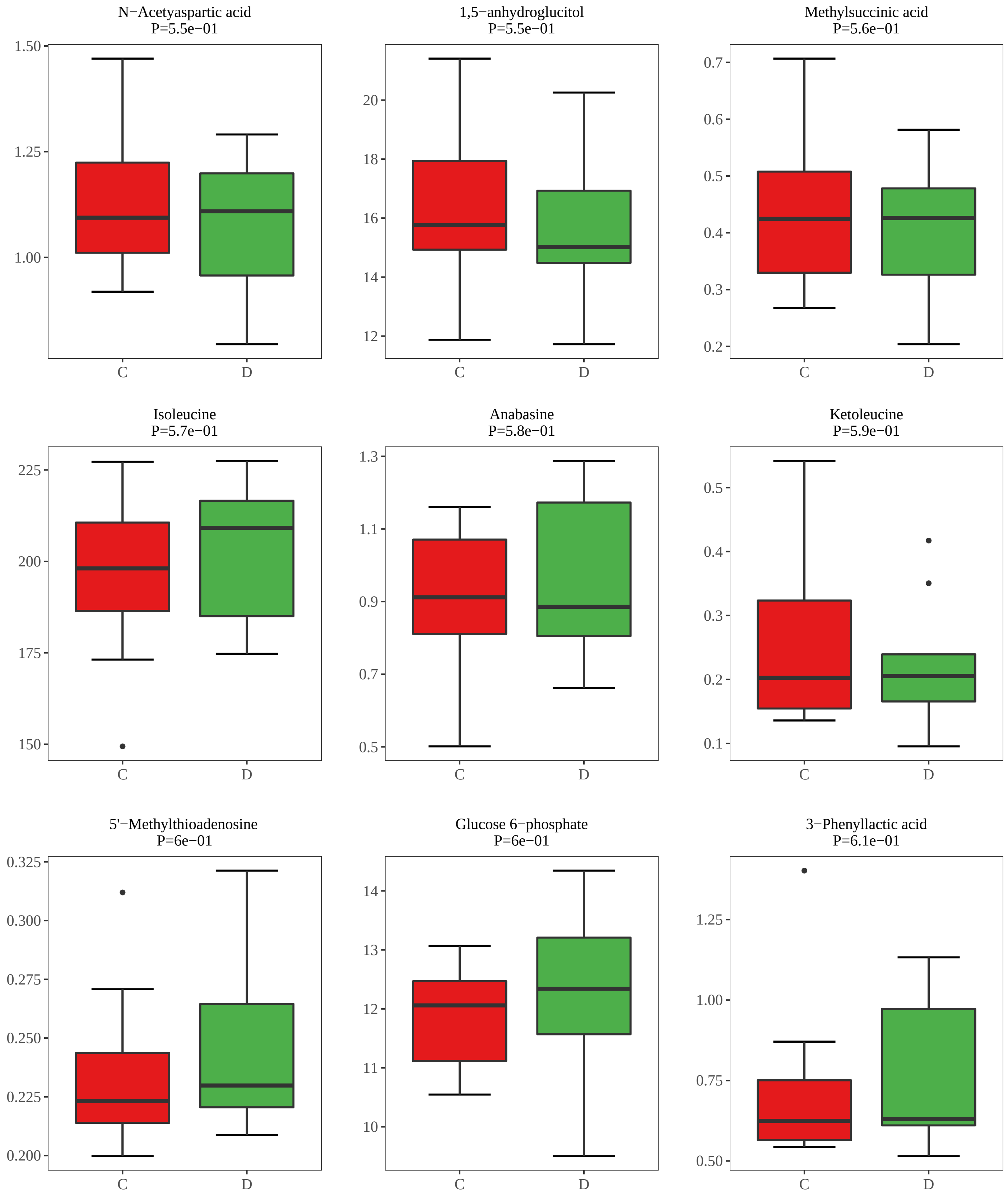
N−Acetyaspartic acid
P=5.5e−01
1.50
1.25
1.00
C
D
1,5−anhydroglucitol
P=5.5e−01
20
18
16
14
12
C
D
Methylsuccinic acid
P=5.6e−01
0.7
0.6
0.5
0.4
0.3
0.2
C
D
Isoleucine
P=5.7e−01
225
200
175
150
C
D
Anabasine
P=5.8e−01
1.3
1.1
0.9
0.7
0.5
C
D
Ketoleucine
P=5.9e−01
0.5
0.4
0.3
0.2
0.1
C
D
5'−Methylthioadenosine
P=6e−01
0.325
0.300
0.275
0.250
0.225
0.200
C
D
Glucose 6−phosphate
P=6e−01
14
13
12
11
10
C
D
3−Phenyllactic acid
P=6.1e−01
1.25
1.00
0.75
0.50
C
D

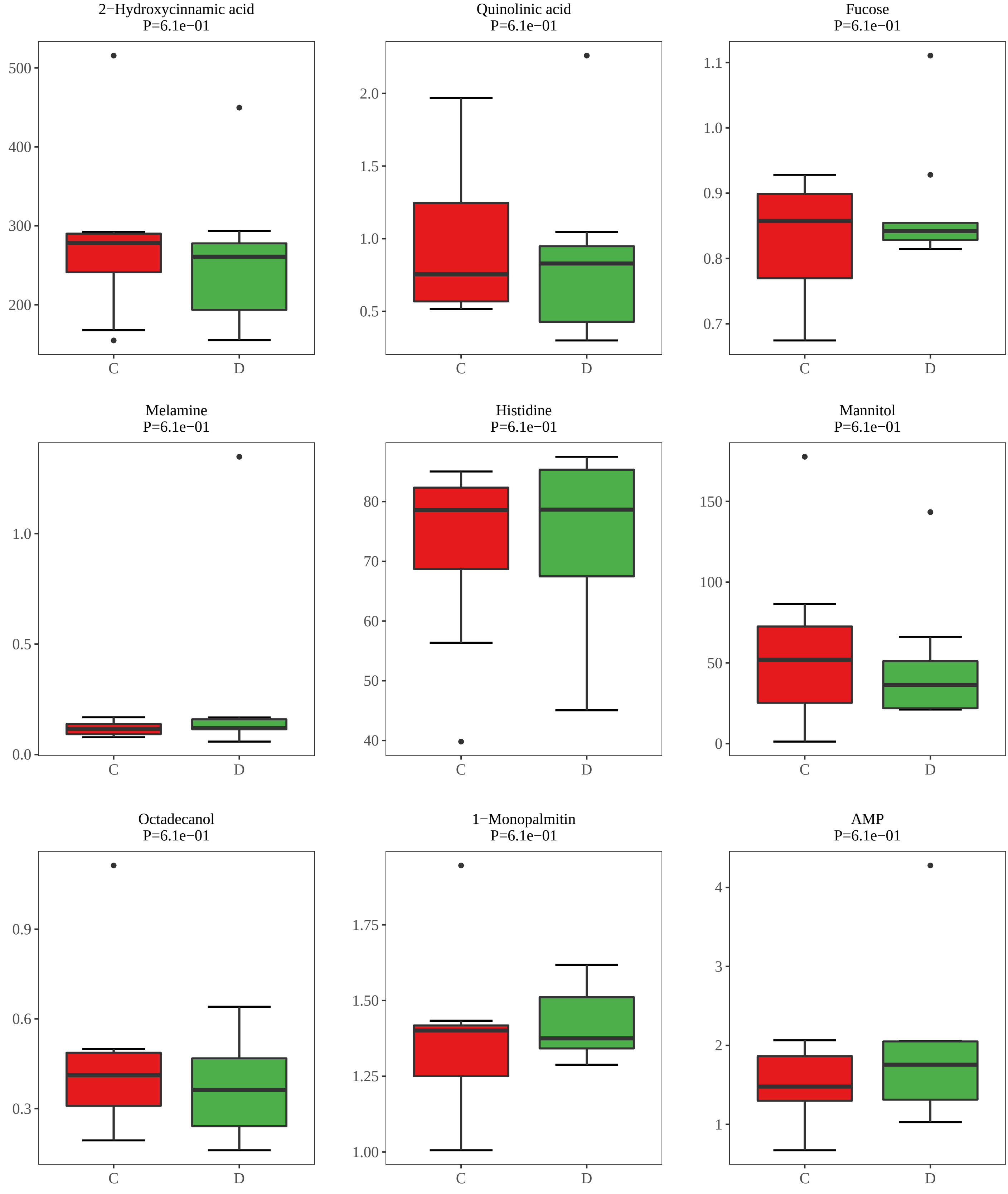
2−Hydroxycinnamic acid
P=6.1e−01
500
400
300
200
C
D
Quinolinic acid
P=6.1e−01
2.0
1.5
1.0
0.5
C
D
Fucose
P=6.1e−01
1.1
1.0
0.9
0.8
0.7
C
D
Melamine
P=6.1e−01
1.0
0.5
0.0
C
D
Histidine
P=6.1e−01
80
70
60
50
40
C
D
Mannitol
P=6.1e−01
150
100
50
0
C
D
Octadecanol
P=6.1e−01
0.9
0.6
0.3
C
D
1−Monopalmitin
P=6.1e−01
1.75
1.50
1.25
1.00
C
D
AMP
P=6.1e−01
4
3
2
1
C
D

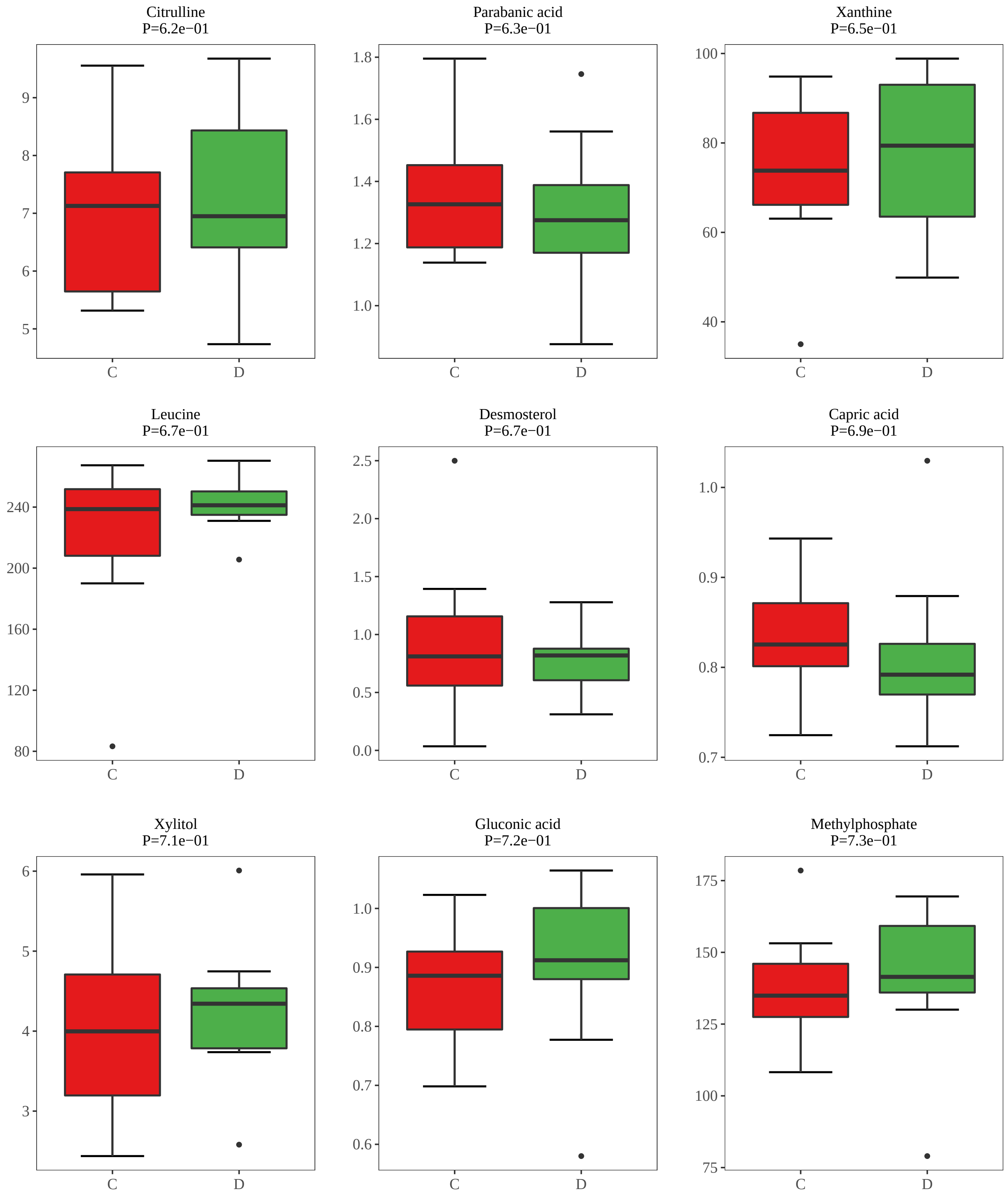
Citrulline
P=6.2e−01
Parabanic acid
P=6.3e−01
Xanthine
P=6.5e−01
Leucine
P=6.7e−01
Desmosterol
P=6.7e−01
Capric acid
P=6.9e−01
Xylitol
P=7.1e−01
Gluconic acid
P=7.2e−01
Methylphosphate
P=7.3e−01
C
D

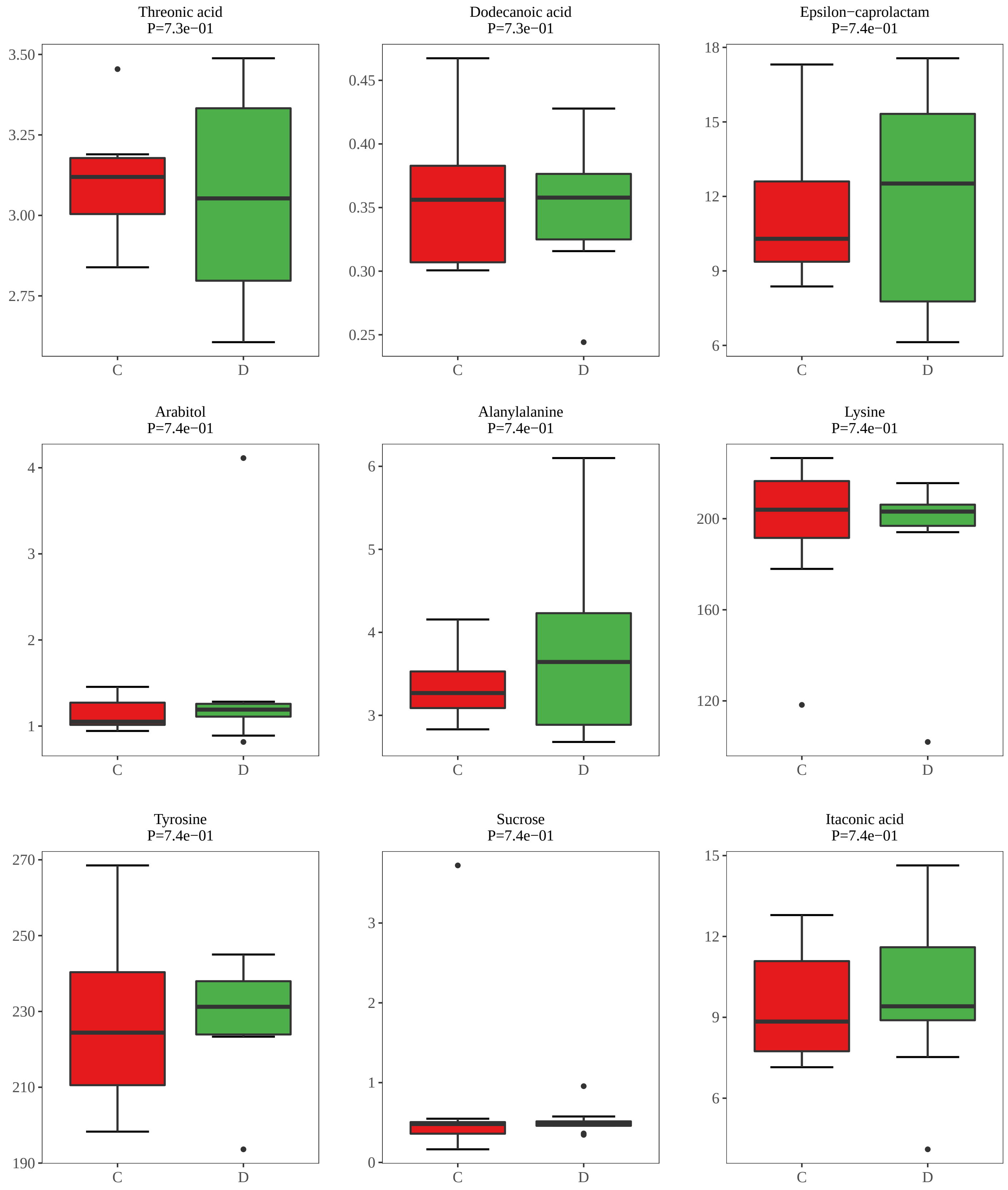

Threonic acid
P=7.3e−01
3.50
3.25
3.00
2.75
C
D
Dodecanoic acid
P=7.3e−01
0.45
0.40
0.35
0.30
0.25
C
D
Epsilon−caprolactam
P=7.4e−01
18
15
12
9
6
C
D
Arabitol
P=7.4e−01
4
3
2
1
C
D
Alanylalanine
P=7.4e−01
6
5
4
3
C
D
Lysine
P=7.4e−01
200
160
120
C
D
Tyrosine
P=7.4e−01
270
250
230
210
190
C
D
Sucrose
P=7.4e−01
3
2
1
0
C
D
Itaconic acid
P=7.4e−01
15
12
9
6
C
D

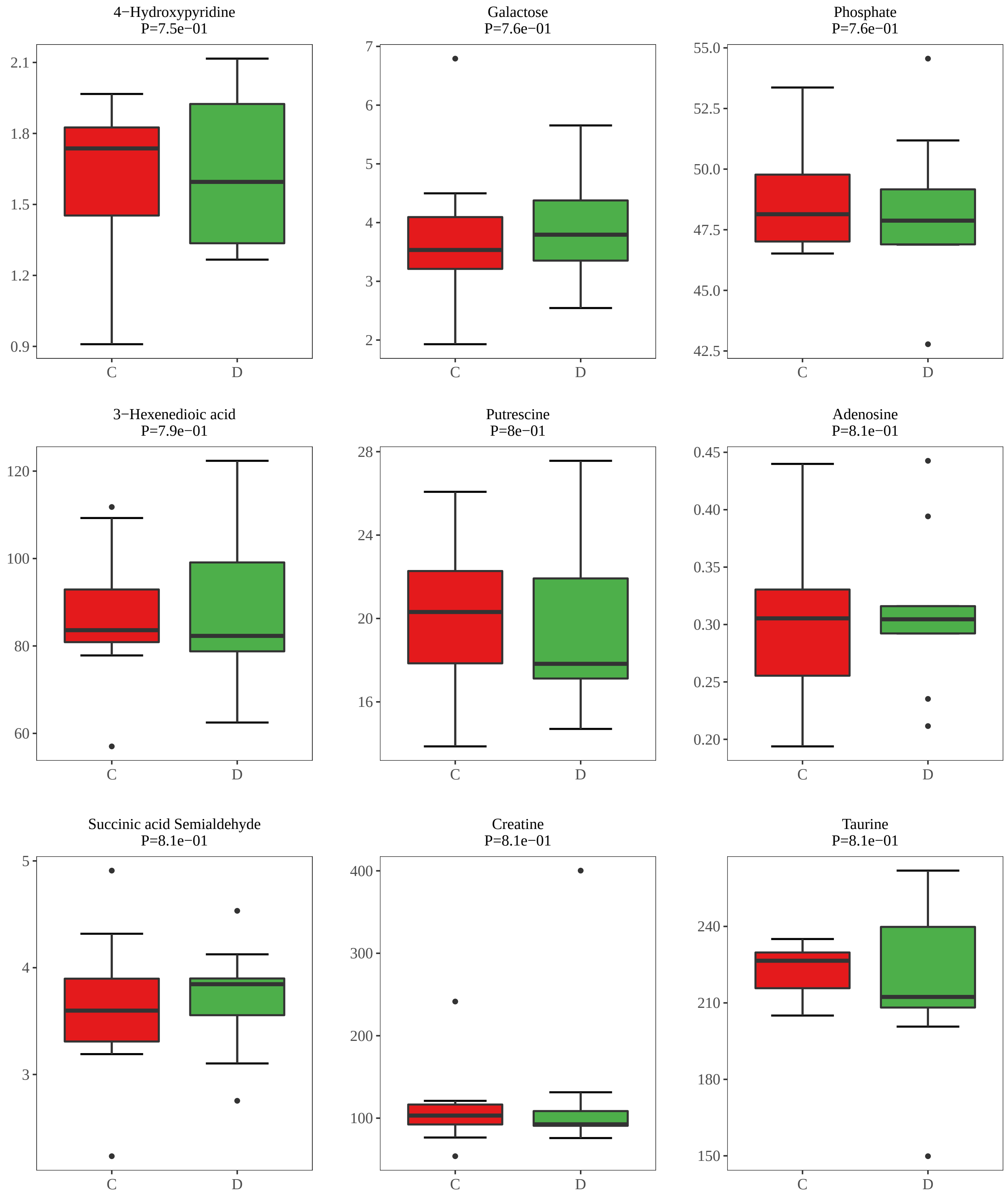

4−Hydroxypyridine
P=7.5e−01
2.1
1.8
1.5
1.2
0.9
C
D
Galactose
P=7.6e−01
7
6
5
4
3
2
C
D
Phosphate
P=7.6e−01
55.0
52.5
50.0
47.5
45.0
42.5
C
D
3−Hexenedioic acid
P=7.9e−01
120
100
80
60
C
D
Putrescine
P=8e−01
28
24
20
16
C
D
Adenosine
P=8.1e−01
0.45
0.40
0.35
0.30
0.25
0.20
C
D
Succinic acid Semialdehyde
P=8.1e−01
5
4
3
C
D
Creatine
P=8.1e−01
400
300
200
100
C
D
Taurine
P=8.1e−01
240
210
180
150
C
D

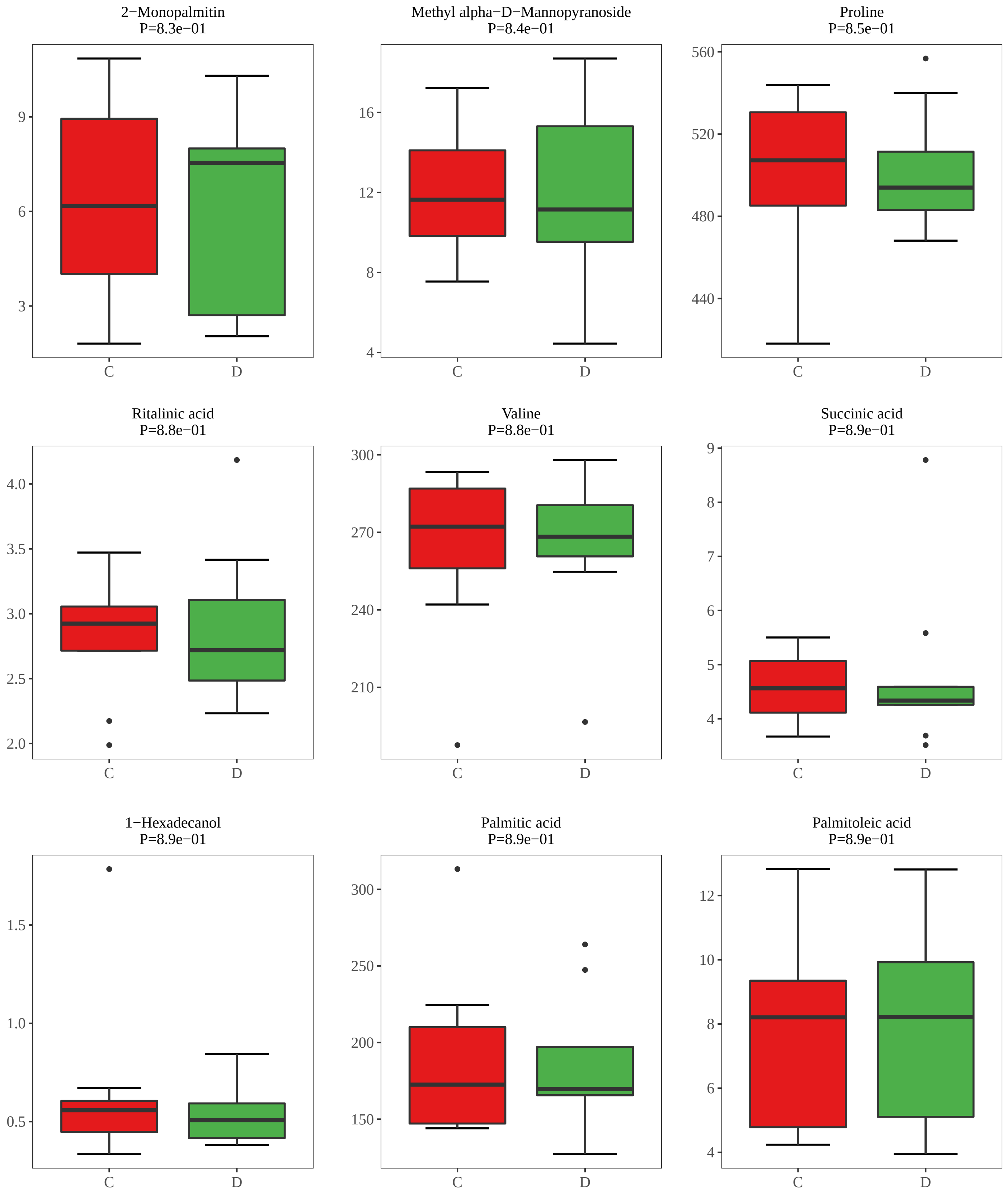
2−Monopalmitin
P=8.3e−01
Methyl alpha−D−Mannopyranoside
P=8.4e−01
Proline
P=8.5e−01
Ritalinic acid
P=8.8e−01
Valine
P=8.8e−01
Succinic acid
P=8.9e−01
1−Hexadecanol
P=8.9e−01
Palmitic acid
P=8.9e−01
Palmitoleic acid
P=8.9e−01
C
D

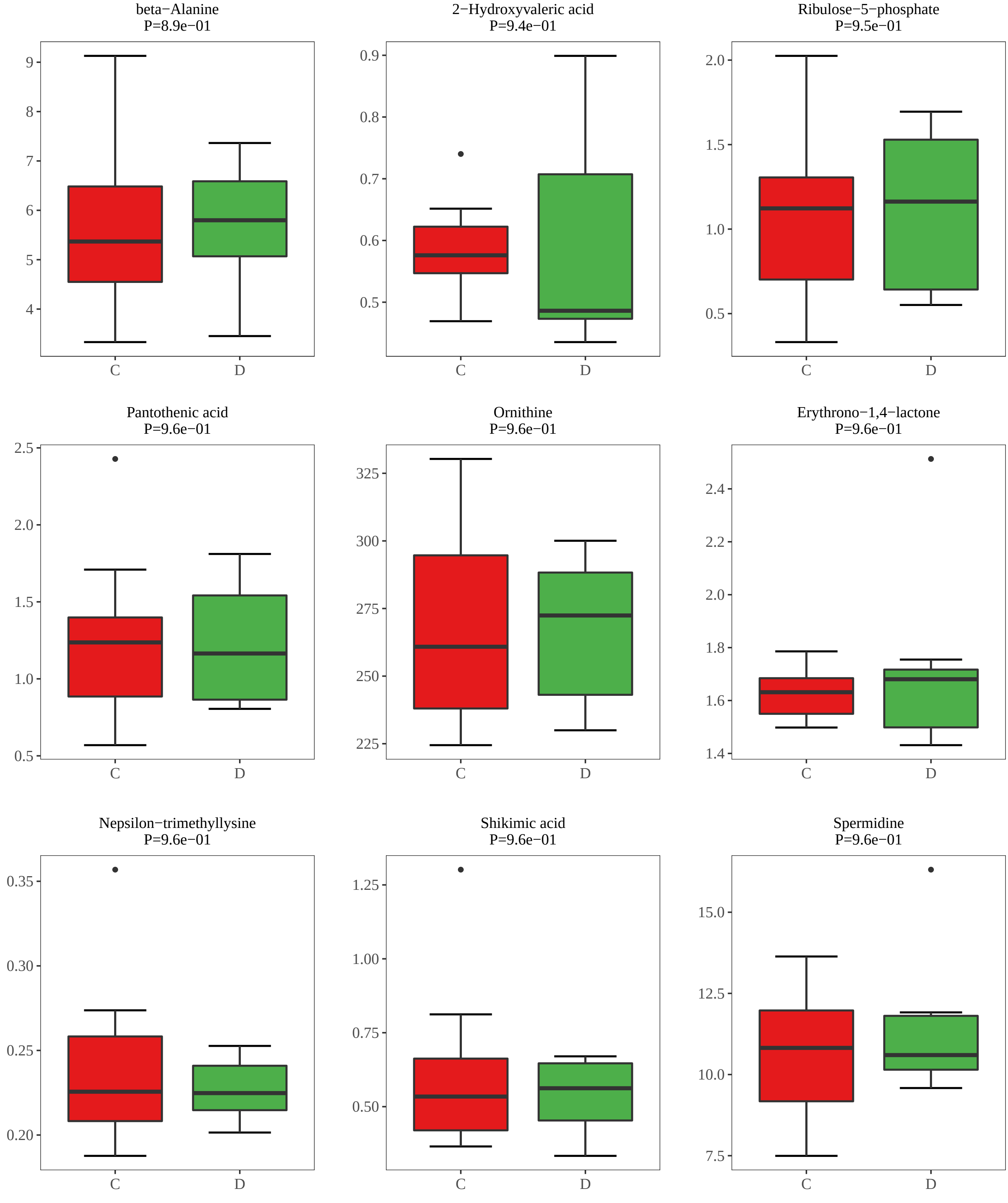

beta−Alanine
P=8.9e−01
2−Hydroxyvaleric acid
P=9.4e−01
Ribulose−5−phosphate
P=9.5e−01
Pantothenic acid
P=9.6e−01
Ornithine
P=9.6e−01
Erythrono−1,4−lactone
P=9.6e−01
Nepsilon−trimethyllysine
P=9.6e−01
Shikimic acid
P=9.6e−01
Spermidine
P=9.6e−01
C
D

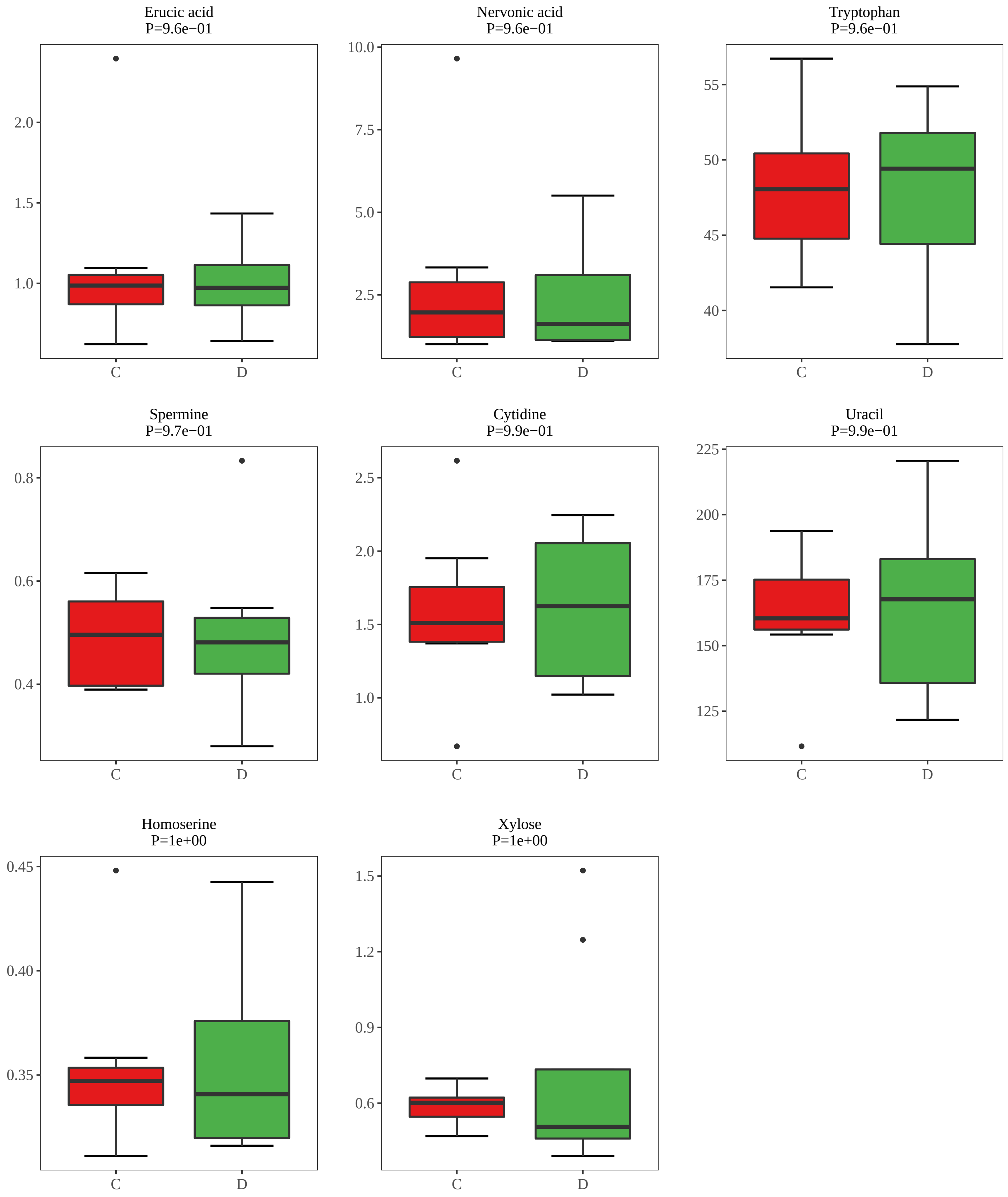
Erucic acid
P=9.6e−01
2.0
1.5
1.0
C
D
Nervonic acid
P=9.6e−01
10.0
7.5
5.0
2.5
C
D
Tryptophan
P=9.6e−01
55
50
45
40
C
D
Spermine
P=9.7e−01
0.8
0.6
0.4
C
D
Cytidine
P=9.9e−01
2.5
2.0
1.5
1.0
C
D
Uracil
P=9.9e−01
225
200
175
150
125
C
D
Homoserine
P=1e+00
0.45
0.40
0.35
C
D
Xylose
P=1e+00
1.5
1.2
0.9
0.6
C
D
