# Supplementary material for: Yiai Fuzheng decoction inhibits triple-negative breast cancer by remodeling the immune microenvironment
Source: Front Immunol. 2025 Sep 30;16:1615631. doi: 10.3389/fimmu.2025.1615631 (PMC12518410; doi:10.3389/fimmu.2025.1615631)
Supplement: Supplementary file 2 [file DataSheet1.zip › Supplementary File 2/Treatment/C_vs_D/05_Univariate_Analysis/AllMet_Vioplot.pdf]

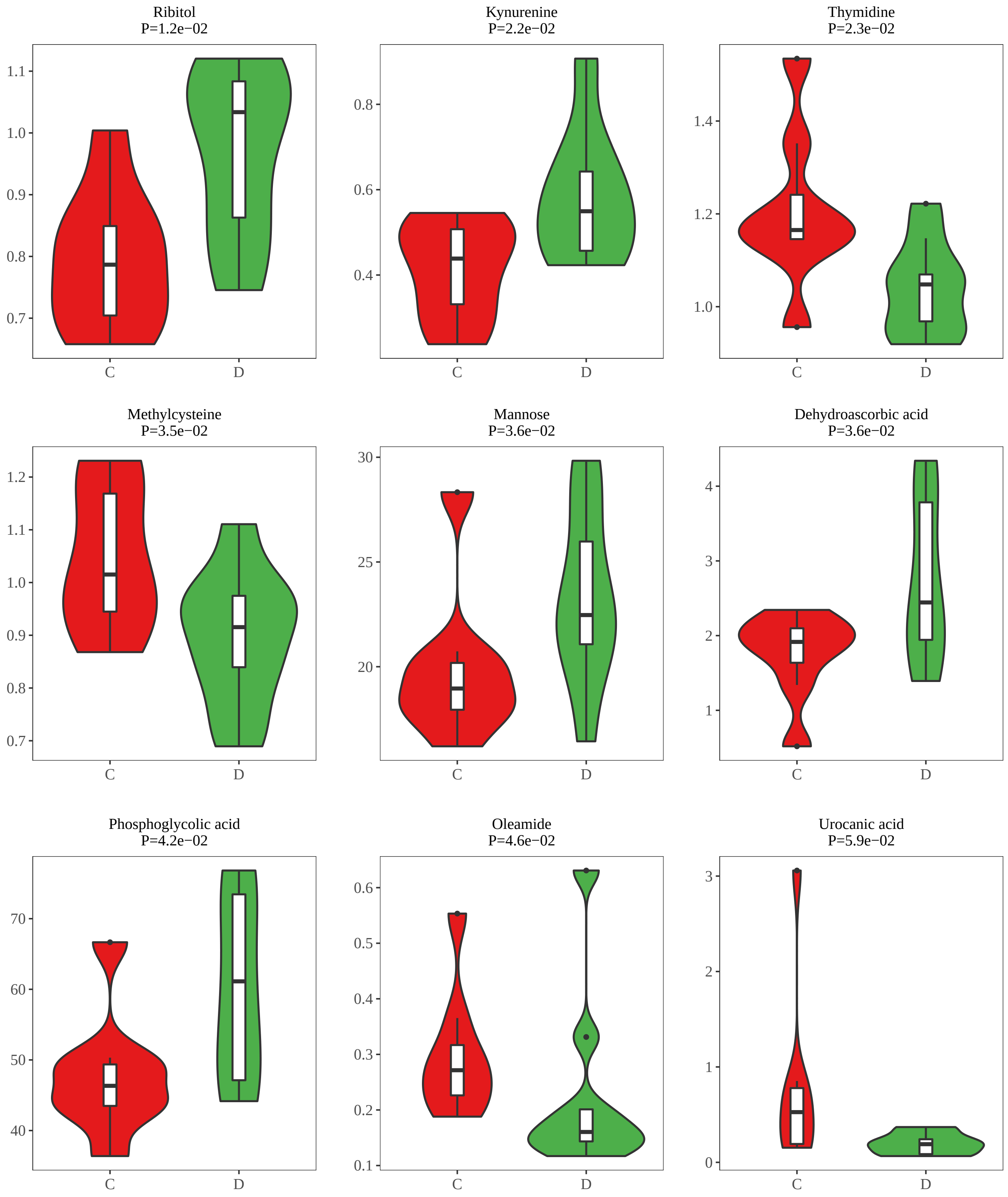
Ribitol
P=1.2e−02
Kynurenine
P=2.2e−02
Thymidine
P=2.3e−02
Methylcysteine
P=3.5e−02
Mannose
P=3.6e−02
Dehydroascorbic acid
P=3.6e−02
Phosphoglycolic acid
P=4.2e−02
Oleamide
P=4.6e−02
Urocanic acid
P=5.9e−02
C
D

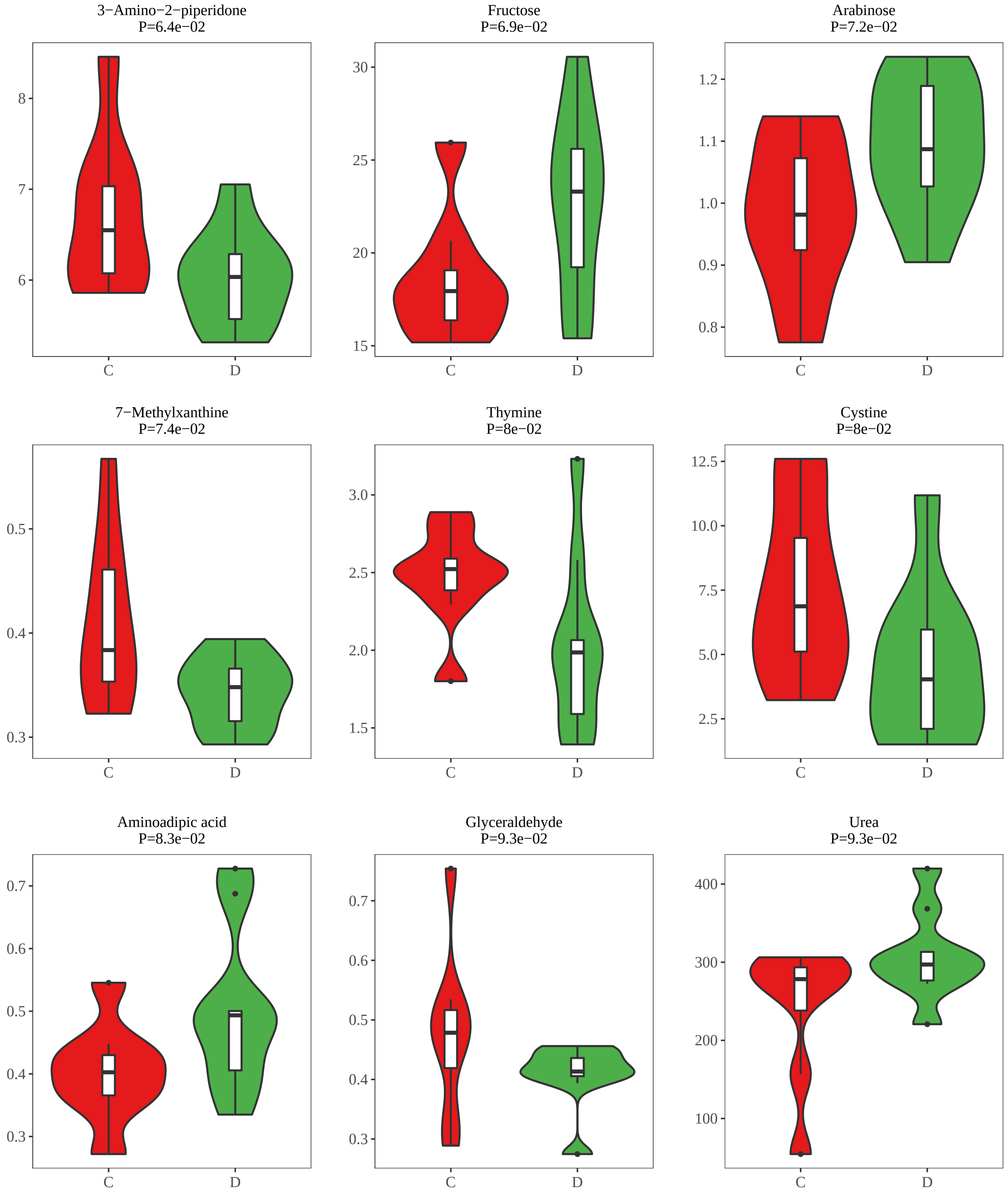
3−Amino−2−piperidone
P=6.4e−02
Fructose
P=6.9e−02
Arabinose
P=7.2e−02
7−Methylxanthine
P=7.4e−02
Thymine
P=8e−02
Cystine
P=8e−02
Aminoadipic acid
P=8.3e−02
Glyceraldehyde
P=9.3e−02
Urea
P=9.3e−02
C
D
8
7
6
30
25
20
15
1.2
1.1
1.0
0.9
0.8
0.5
0.4
0.3
3.0
2.5
2.0
1.5
12.5
10.0
7.5
5.0
2.5
0.7
0.6
0.5
0.4
0.3
400
300
200
100

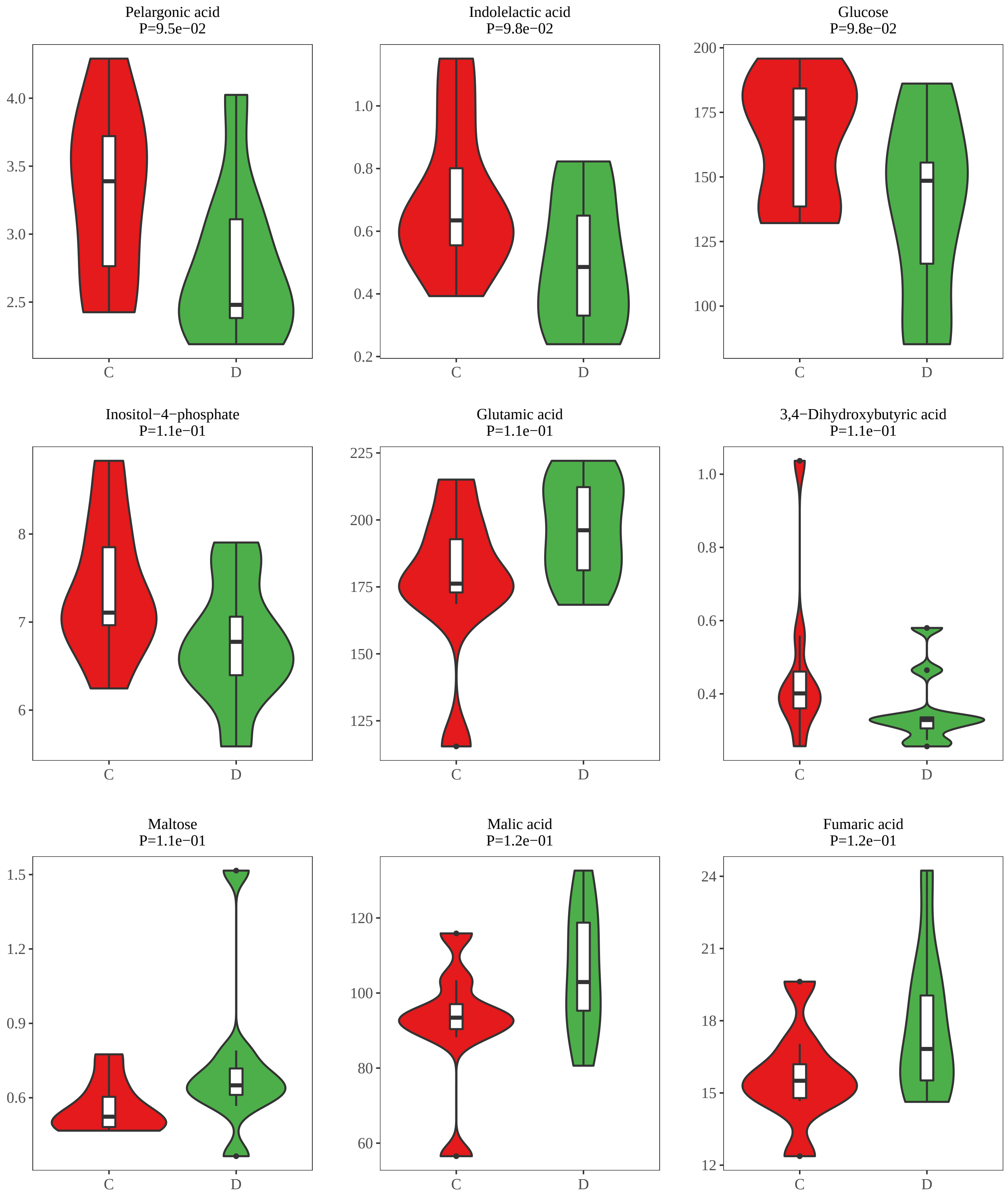

Pelargonic acid
P=9.5e−02
Indolelactic acid
P=9.8e−02
Glucose
P=9.8e−02
Inositol−4−phosphate
P=1.1e−01
Glutamic acid
P=1.1e−01
3,4−Dihydroxybutyric acid
P=1.1e−01
Maltose
P=1.1e−01
Malic acid
P=1.2e−01
Fumaric acid
P=1.2e−01
C
D

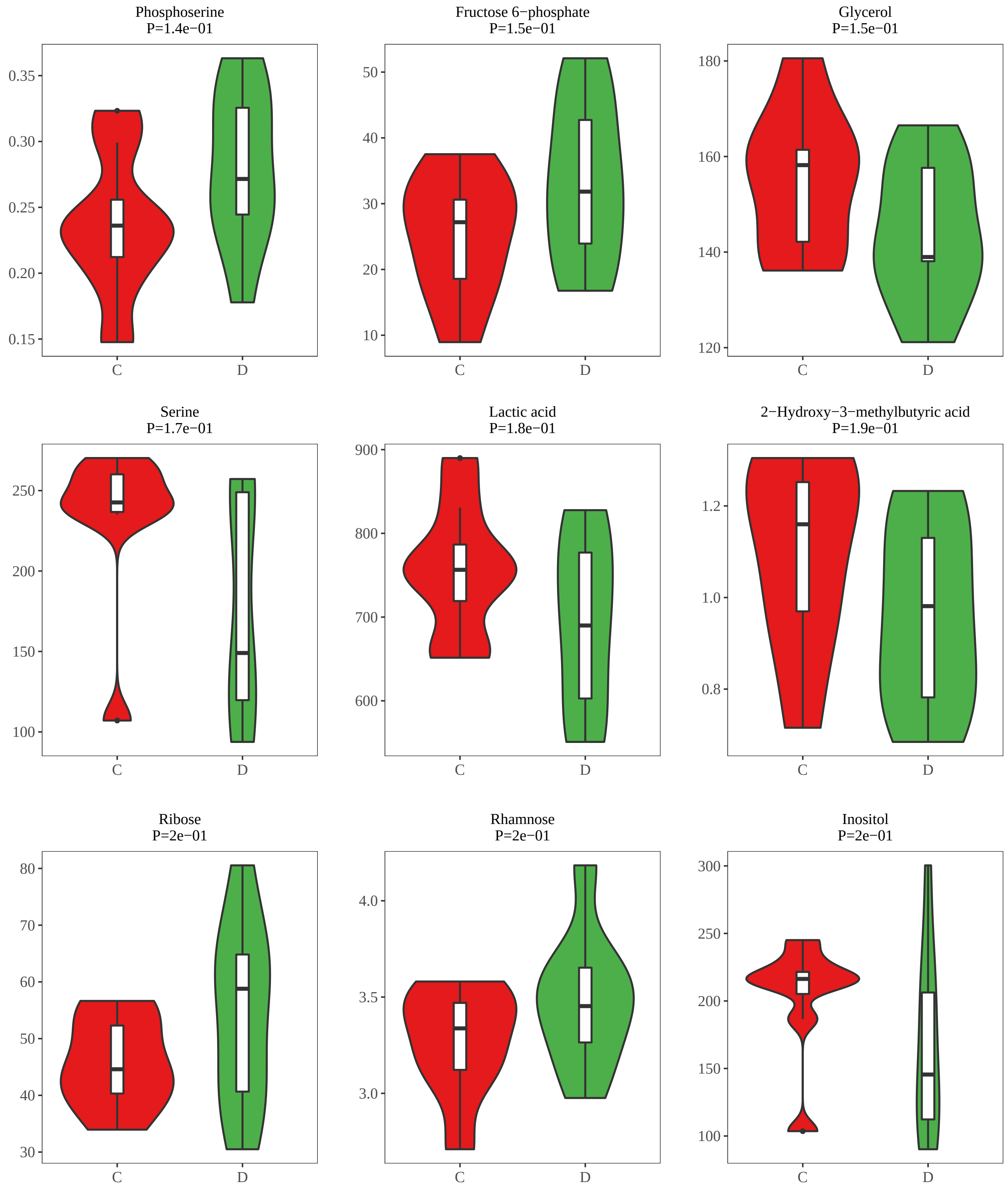

Phosphoserine
P=1.4e−01
Fructose 6−phosphate
P=1.5e−01
Glycerol
P=1.5e−01
Serine
P=1.7e−01
Lactic acid
P=1.8e−01
2−Hydroxy−3−methylbutyric acid
P=1.9e−01
Ribose
P=2e−01
Rhamnose
P=2e−01
Inositol
P=2e−01
C
D

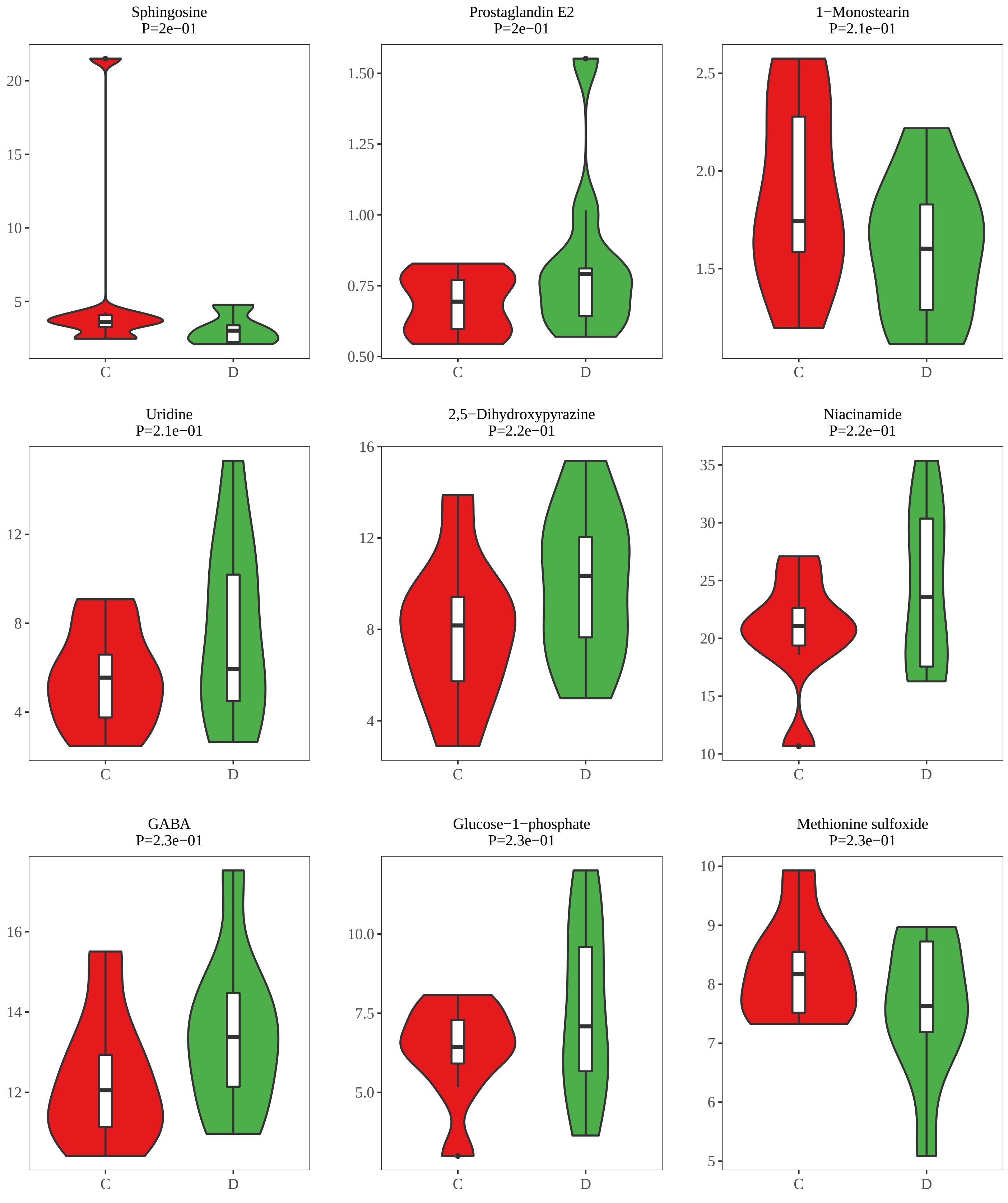
Sphingosine
P=2e−01
20
15
10
5
C
D
Prostaglandin E2
P=2e−01
1.50
1.25
1.00
0.75
0.50
C
D
1−Monostearin
P=2.1e−01
2.5
2.0
1.5
C
D
Uridine
P=2.1e−01
12
8
4
C
D
2,5−Dihydroxypyrazine
P=2.2e−01
16
12
8
4
C
D
Niacinamide
P=2.2e−01
35
30
25
20
15
10
C
D
GABA
P=2.3e−01
16
14
12
C
D
Glucose−1−phosphate
P=2.3e−01
10.0
7.5
5.0
C
D
Methionine sulfoxide
P=2.3e−01
10
9
8
7
6
5
C
D

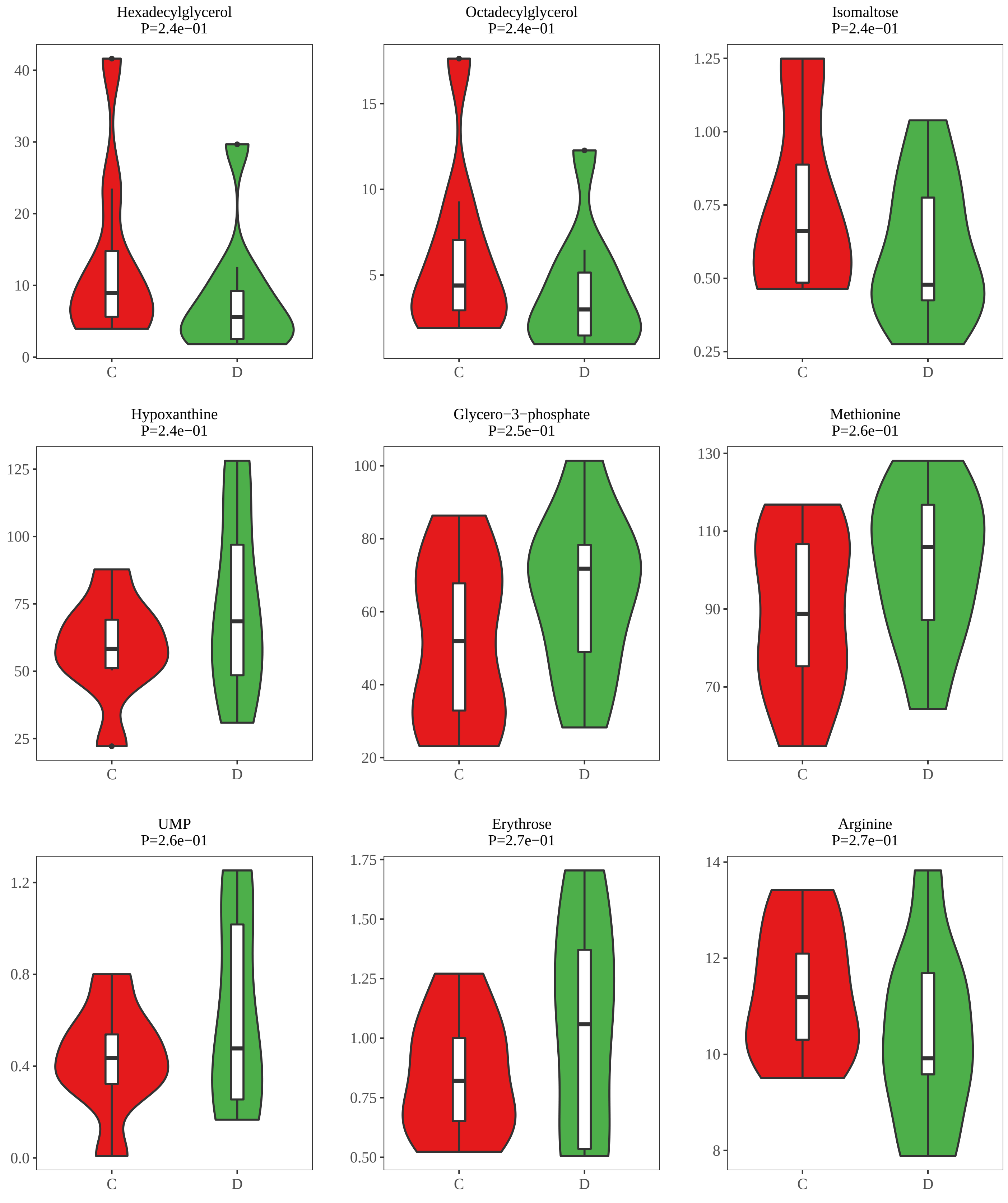
Hexadecylglycerol
P=2.4e−01
Octadecylglycerol
P=2.4e−01
Isomaltose
P=2.4e−01
Hypoxanthine
P=2.4e−01
Glycero−3−phosphate
P=2.5e−01
Methionine
P=2.6e−01
UMP
P=2.6e−01
Erythrose
P=2.7e−01
Arginine
P=2.7e−01
C
D

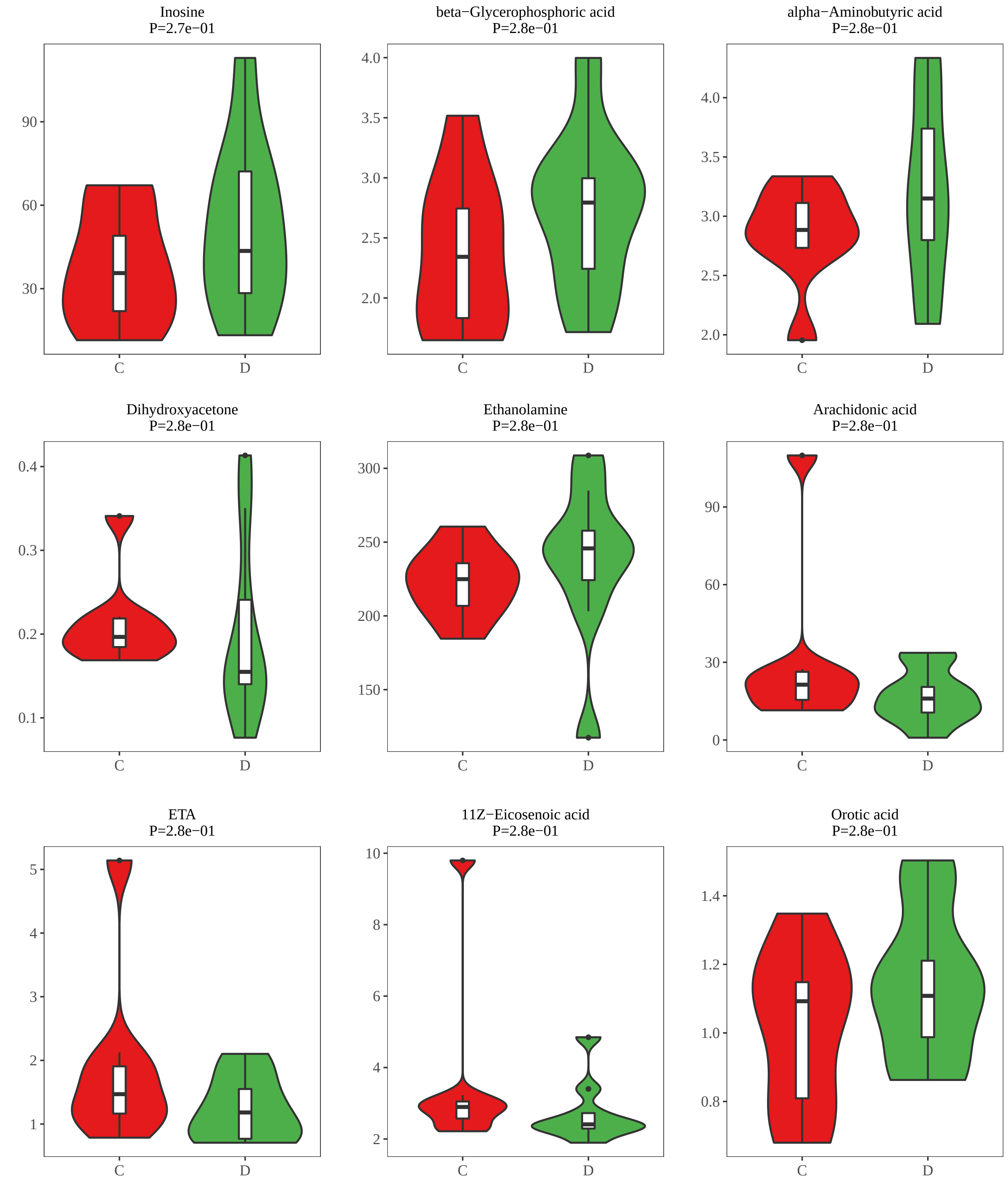
Inosine
P=2.7e−01
30
60
90
C
D
beta−Glycerophosphoric acid
P=2.8e−01
2.0
2.5
3.0
3.5
4.0
C
D
alpha−Aminobutyric acid
P=2.8e−01
2.0
2.5
3.0
3.5
4.0
C
D
Dihydroxyacetone
P=2.8e−01
0.1
0.2
0.3
0.4
C
D
Ethanolamine
P=2.8e−01
150
200
250
300
C
D
Arachidonic acid
P=2.8e−01
0
30
60
90
C
D
ETA
P=2.8e−01
1
2
3
4
5
C
D
11Z−Eicosenoic acid
P=2.8e−01
2
4
6
8
10
C
D
Orotic acid
P=2.8e−01
0.8
1.0
1.2
1.4
C
D

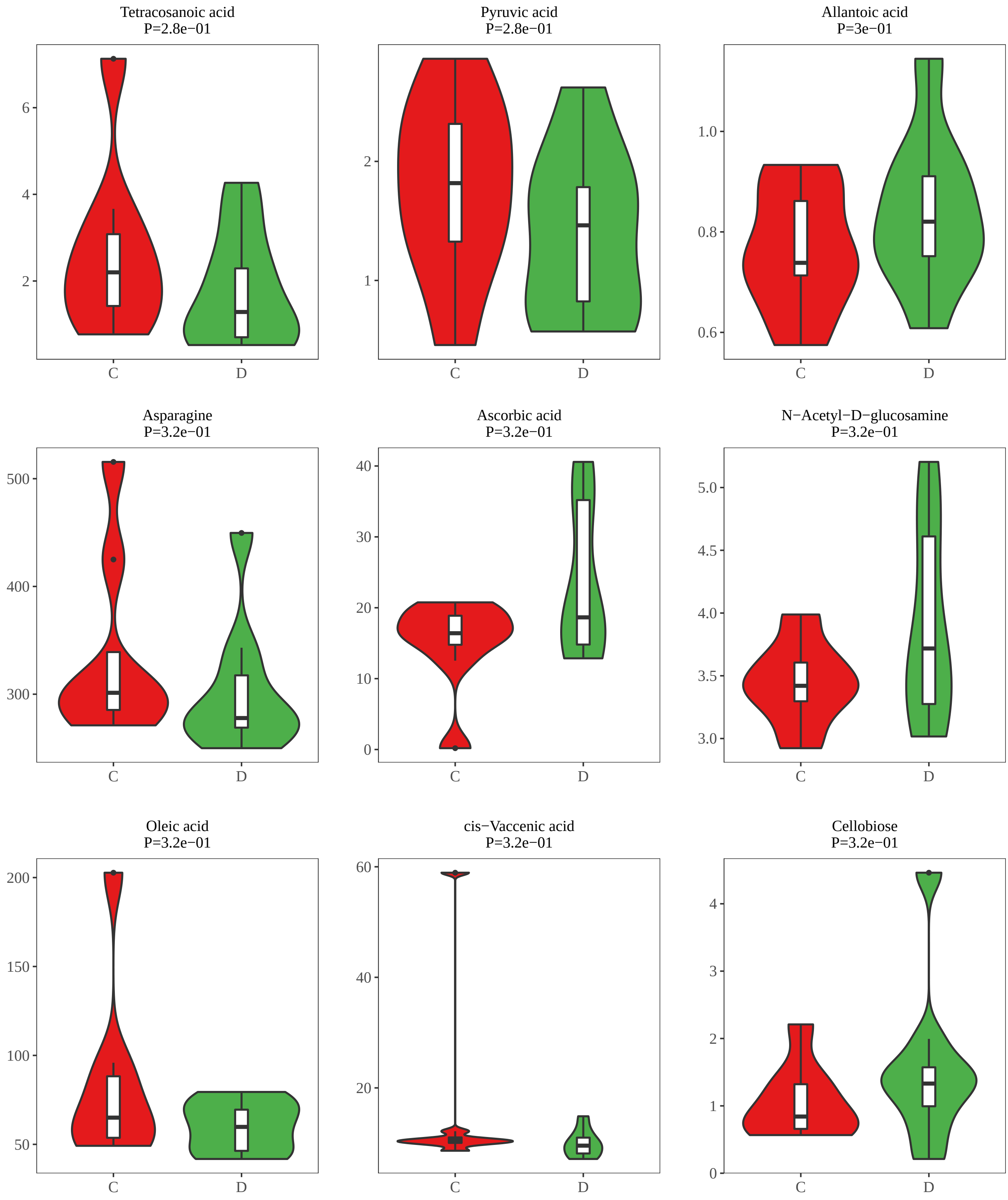

Tetracosanoic acid
P=2.8e−01
6
4
2
C
D
Pyruvic acid
P=2.8e−01
2
1
C
D
Allantoic acid
P=3e−01
1.0
0.8
0.6
C
D
Asparagine
P=3.2e−01
500
400
300
C
D
Ascorbic acid
P=3.2e−01
40
30
20
10
0
C
D
N−Acetyl−D−glucosamine
P=3.2e−01
5.0
4.5
4.0
3.5
3.0
C
D
Oleic acid
P=3.2e−01
200
150
100
50
C
D
cis−Vaccenic acid
P=3.2e−01
60
40
20
C
D
Cellobiose
P=3.2e−01
4
3
2
1
0
C
D

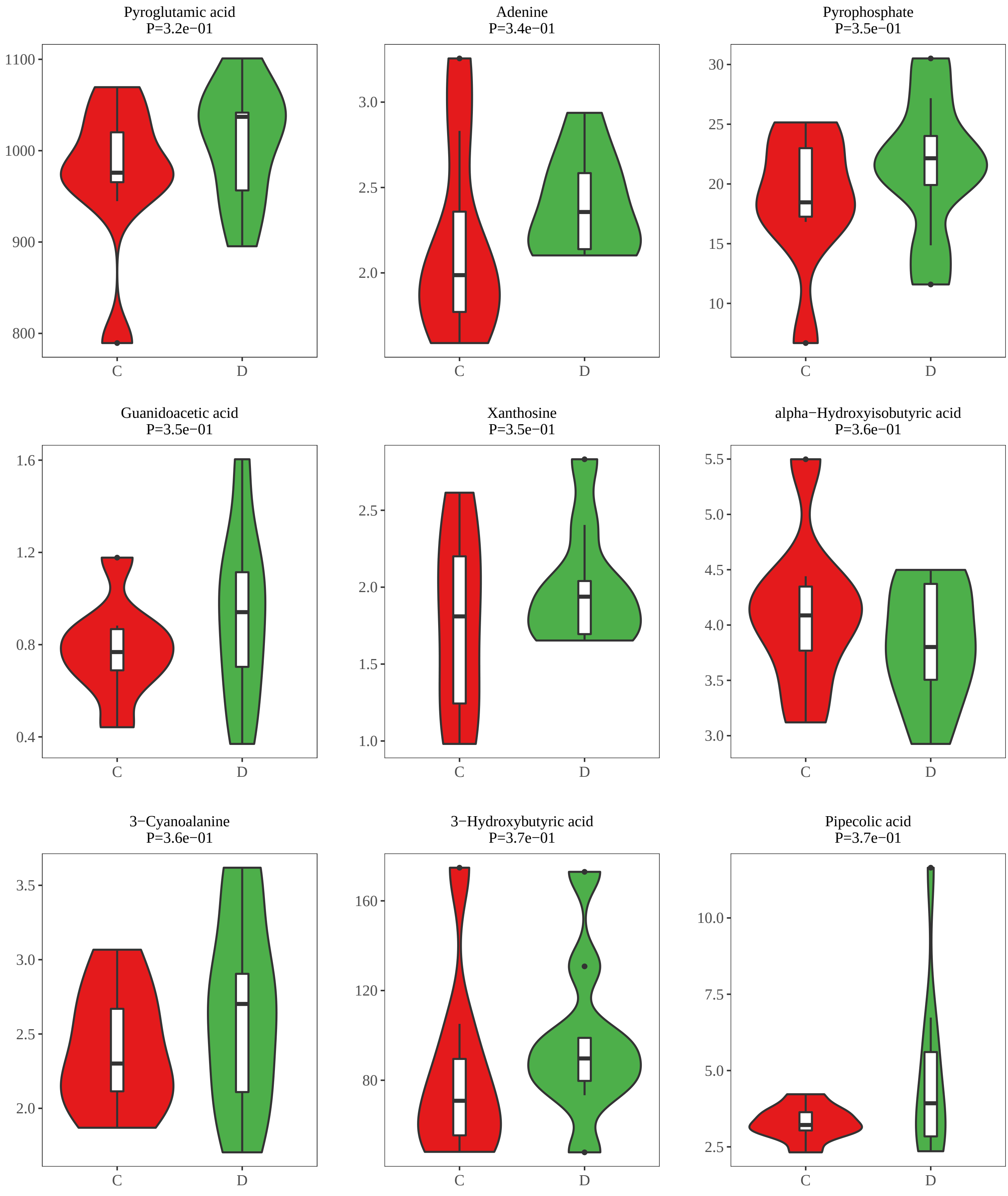
Pyroglutamic acid
P=3.2e−01
1100
1000
900
800
C
D
Adenine
P=3.4e−01
3.0
2.5
2.0
C
D
Pyrophosphate
P=3.5e−01
30
25
20
15
10
C
D
Guanidoacetic acid
P=3.5e−01
1.6
1.2
0.8
0.4
C
D
Xanthosine
P=3.5e−01
2.5
2.0
1.5
1.0
C
D
alpha−Hydroxyisobutyric acid
P=3.6e−01
5.5
5.0
4.5
4.0
3.5
3.0
C
D
3−Cyanoalanine
P=3.6e−01
3.5
3.0
2.5
2.0
C
D
3−Hydroxybutyric acid
P=3.7e−01
160
120
80
C
D
Pipecolic acid
P=3.7e−01
10.0
7.5
5.0
2.5
C
D

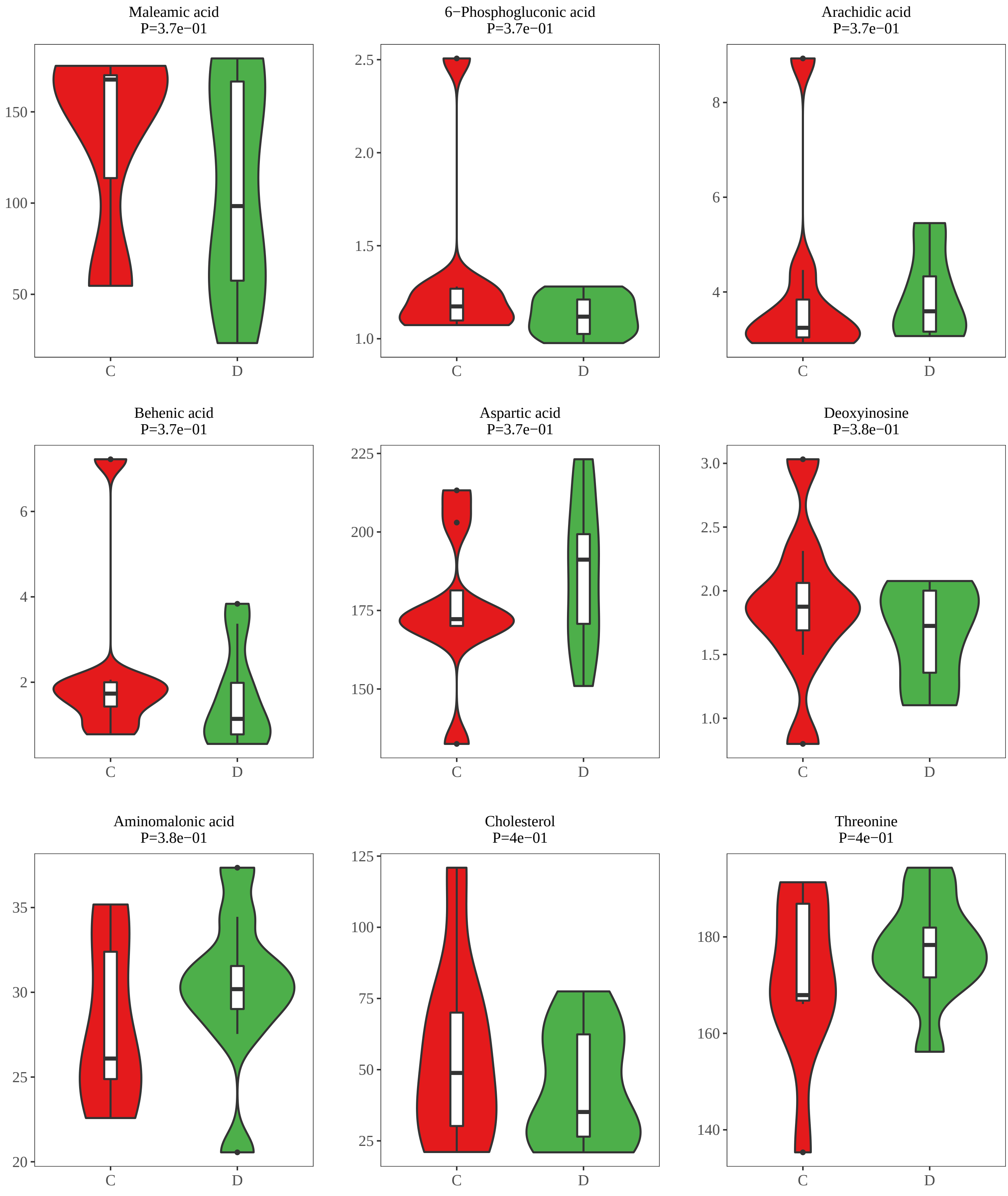

Maleamic acid
P=3.7e−01
150
100
50
C
D
6−Phosphogluconic acid
P=3.7e−01
2.5
2.0
1.5
1.0
C
D
Arachidic acid
P=3.7e−01
8
6
4
C
D
Behenic acid
P=3.7e−01
6
4
2
C
D
Aspartic acid
P=3.7e−01
225
200
175
150
C
D
Deoxyinosine
P=3.8e−01
3.0
2.5
2.0
1.5
1.0
C
D
Aminomalonic acid
P=3.8e−01
35
30
25
20
C
D
Cholesterol
P=4e−01
125
100
75
50
25
C
D
Threonine
P=4e−01
180
160
140
C
D

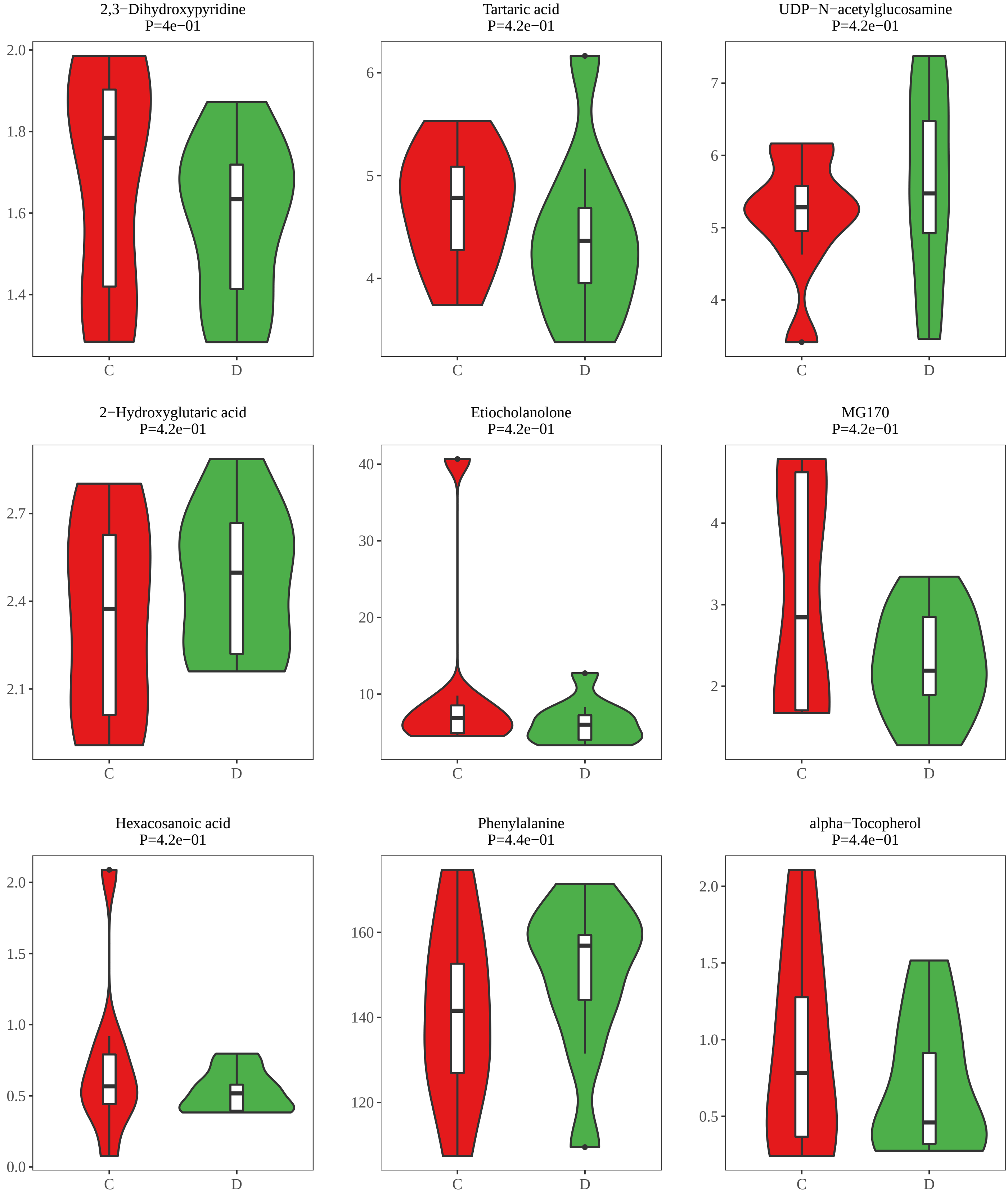
2,3−Dihydroxypyridine
P=4e−01
Tartaric acid
P=4.2e−01
UDP−N−acetylglucosamine
P=4.2e−01
2−Hydroxyglutaric acid
P=4.2e−01
Etiocholanolone
P=4.2e−01
MG170
P=4.2e−01
Hexacosanoic acid
P=4.2e−01
Phenylalanine
P=4.4e−01
alpha−Tocopherol
P=4.4e−01
C
D

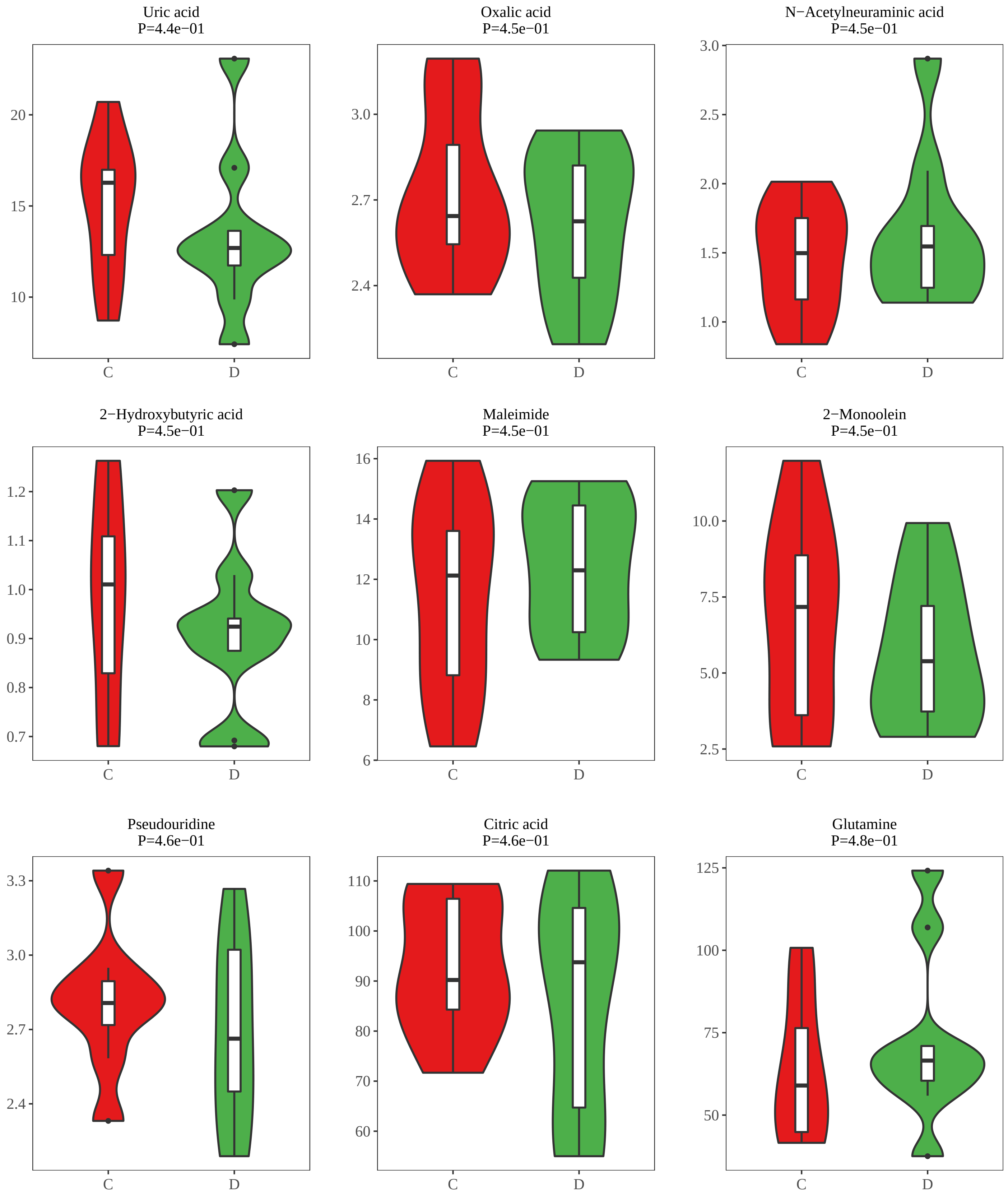
Uric acid
P=4.4e−01
20
15
10
C
D
Oxalic acid
P=4.5e−01
3.0
2.7
2.4
C
D
N−Acetylneuraminic acid
P=4.5e−01
3.0
2.5
2.0
1.5
1.0
C
D
2−Hydroxybutyric acid
P=4.5e−01
1.2
1.1
1.0
0.9
0.8
0.7
C
D
Maleimide
P=4.5e−01
16
14
12
10
8
6
C
D
2−Monoolein
P=4.5e−01
10.0
7.5
5.0
2.5
C
D
Pseudouridine
P=4.6e−01
3.3
3.0
2.7
2.4
C
D
Citric acid
P=4.6e−01
110
100
90
80
70
60
C
D
Glutamine
P=4.8e−01
125
100
75
50
C
D

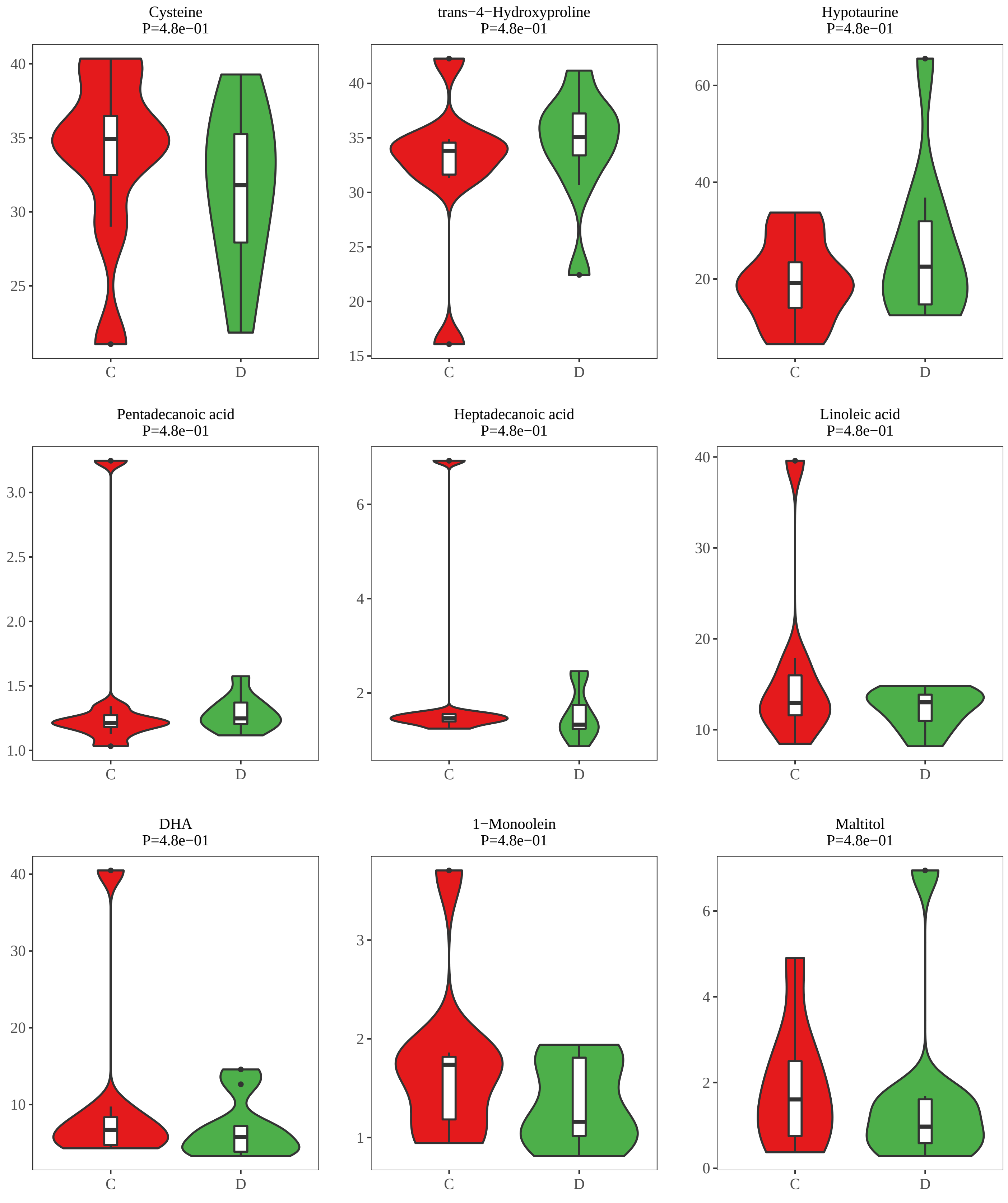
Cysteine
P=4.8e−01
trans−4−Hydroxyproline
P=4.8e−01
Hypotaurine
P=4.8e−01
Pentadecanoic acid
P=4.8e−01
Heptadecanoic acid
P=4.8e−01
Linoleic acid
P=4.8e−01
DHA
P=4.8e−01
1−Monoolein
P=4.8e−01
Maltitol
P=4.8e−01
C
D

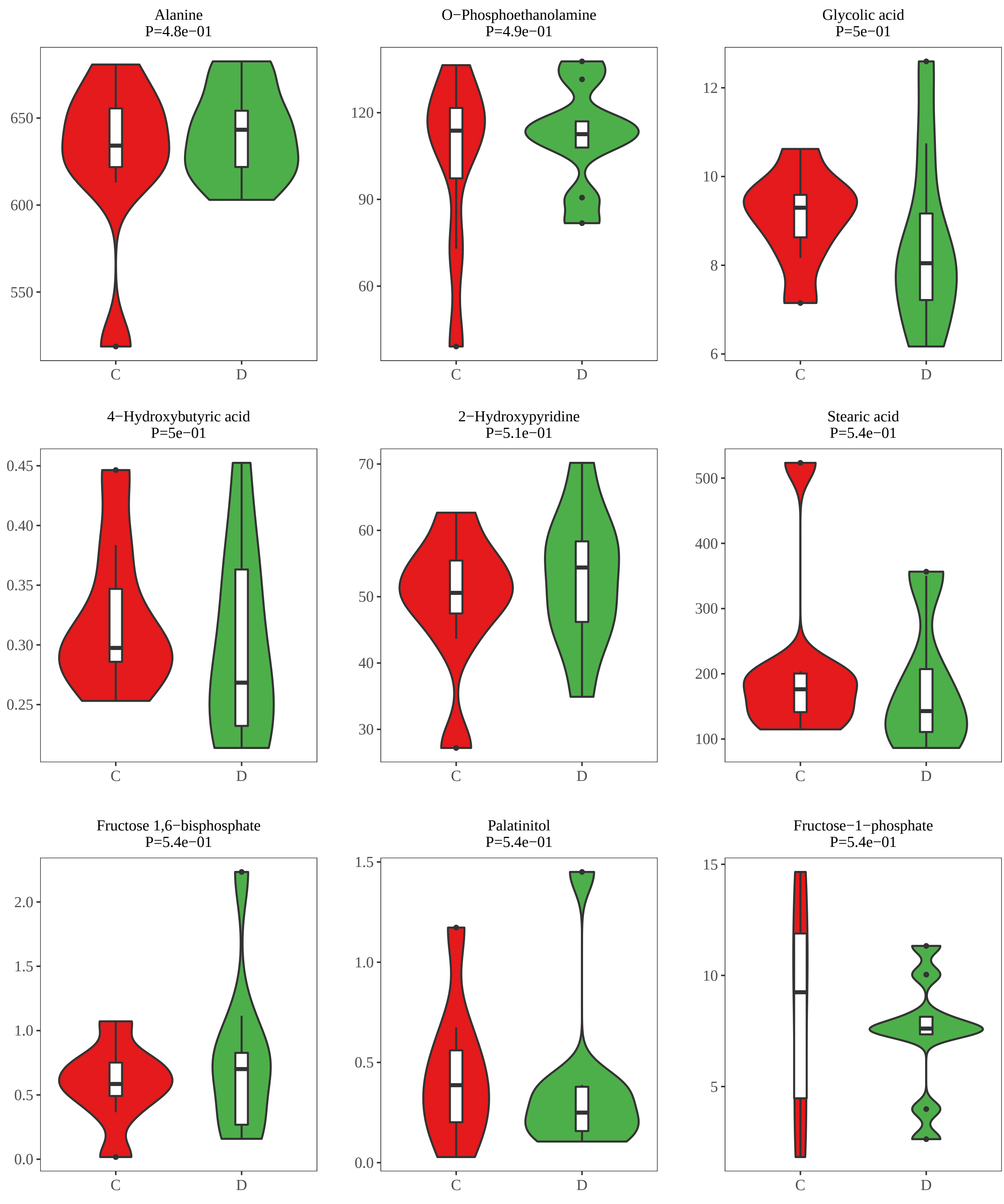

Alanine
P=4.8e−01
O−Phosphoethanolamine
P=4.9e−01
Glycolic acid
P=5e−01
4−Hydroxybutyric acid
P=5e−01
2−Hydroxypyridine
P=5.1e−01
Stearic acid
P=5.4e−01
Fructose 1,6−bisphosphate
P=5.4e−01
Palatinitol
P=5.4e−01
Fructose−1−phosphate
P=5.4e−01
C
D

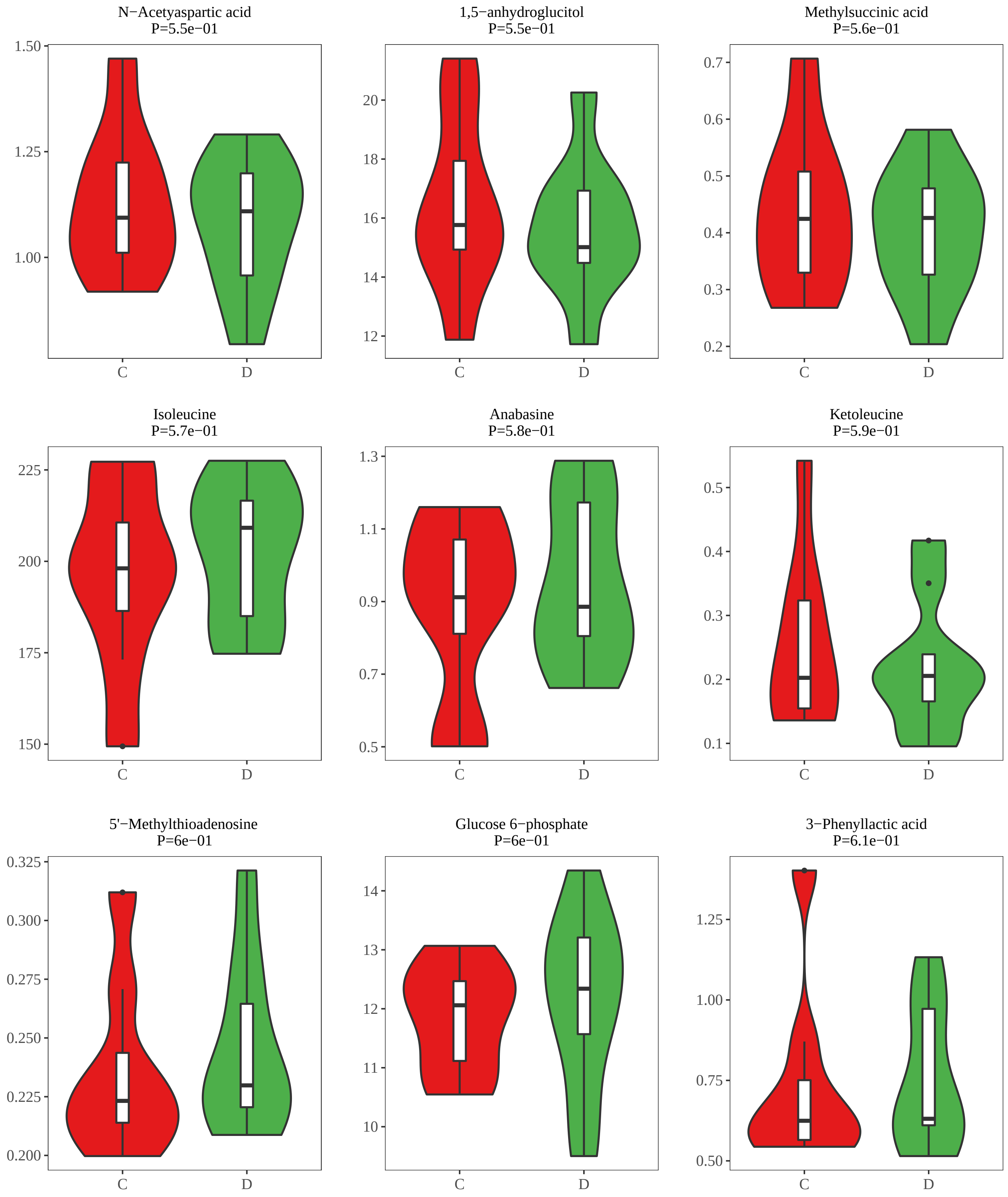
N−Acetyaspartic acid
P=5.5e−01
1,5−anhydroglucitol
P=5.5e−01
Methylsuccinic acid
P=5.6e−01
Isoleucine
P=5.7e−01
Anabasine
P=5.8e−01
Ketoleucine
P=5.9e−01
5'−Methylthioadenosine
P=6e−01
Glucose 6−phosphate
P=6e−01
3−Phenyllactic acid
P=6.1e−01
C
D

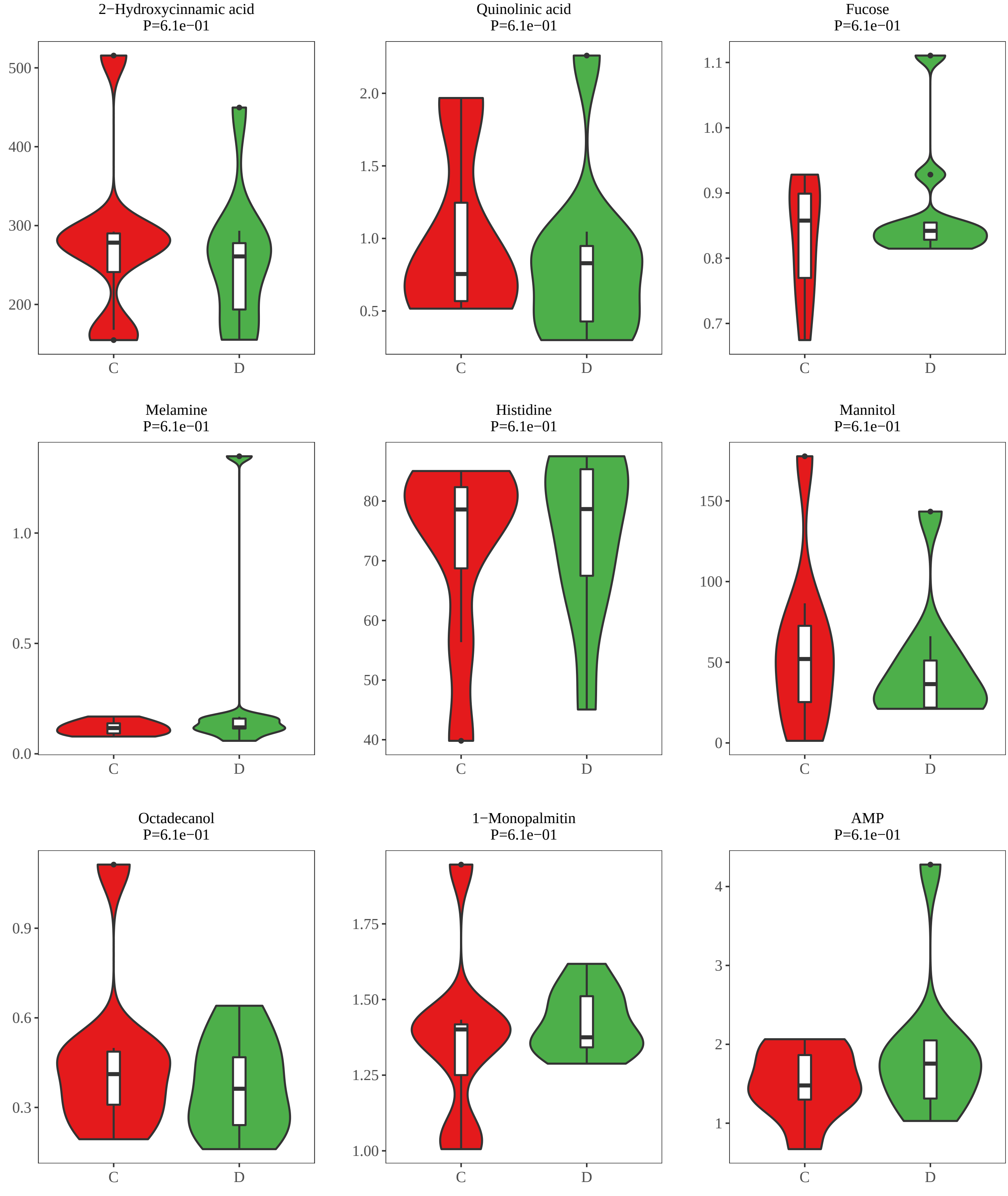
2−Hydroxycinnamic acid
P=6.1e−01
Quinolinic acid
P=6.1e−01
Fucose
P=6.1e−01
Melamine
P=6.1e−01
Histidine
P=6.1e−01
Mannitol
P=6.1e−01
Octadecanol
P=6.1e−01
1−Monopalmitin
P=6.1e−01
AMP
P=6.1e−01
C
D

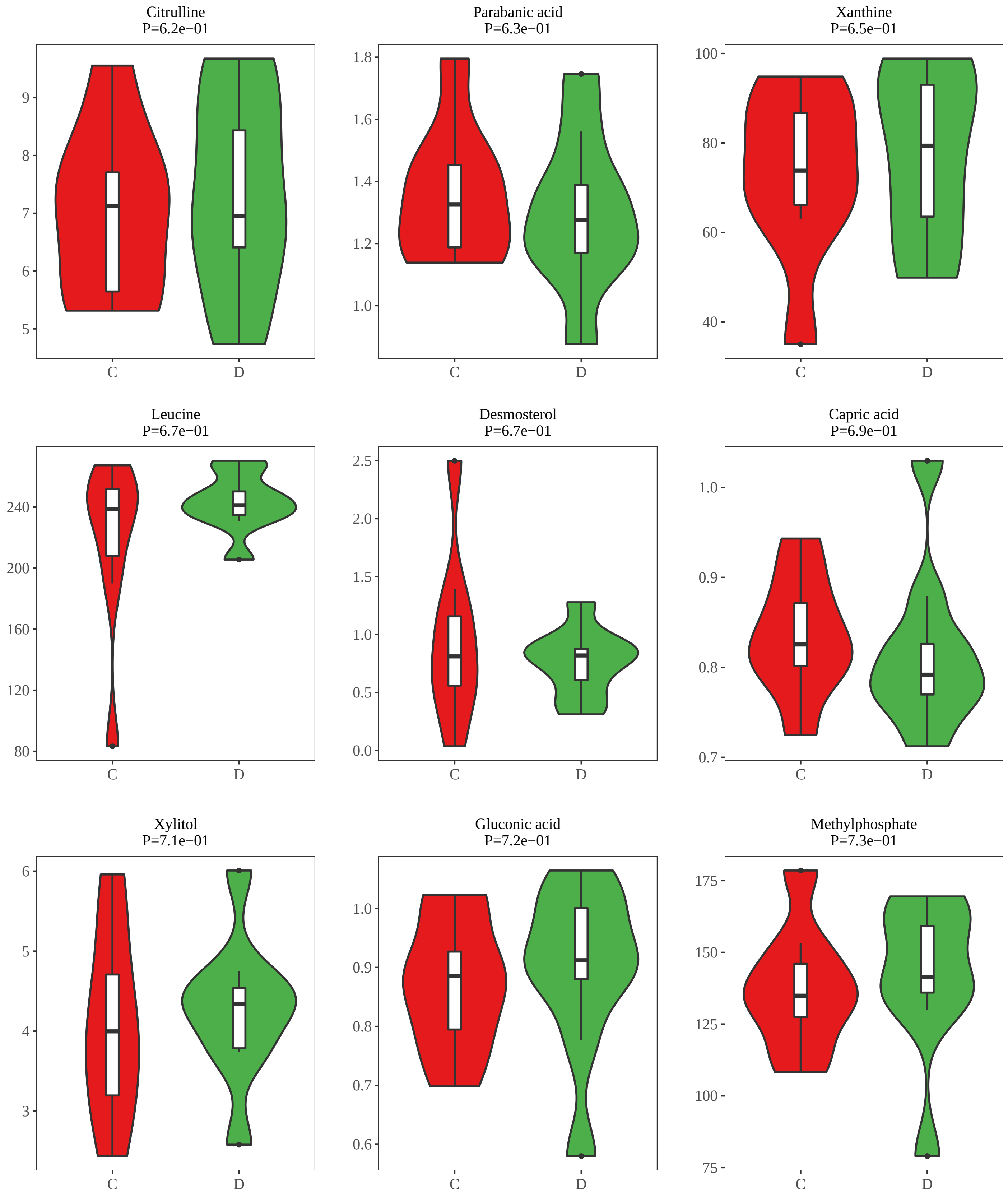
Citrulline
P=6.2e−01
Parabanic acid
P=6.3e−01
Xanthine
P=6.5e−01
Leucine
P=6.7e−01
Desmosterol
P=6.7e−01
Capric acid
P=6.9e−01
Xylitol
P=7.1e−01
Gluconic acid
P=7.2e−01
Methylphosphate
P=7.3e−01
C
D

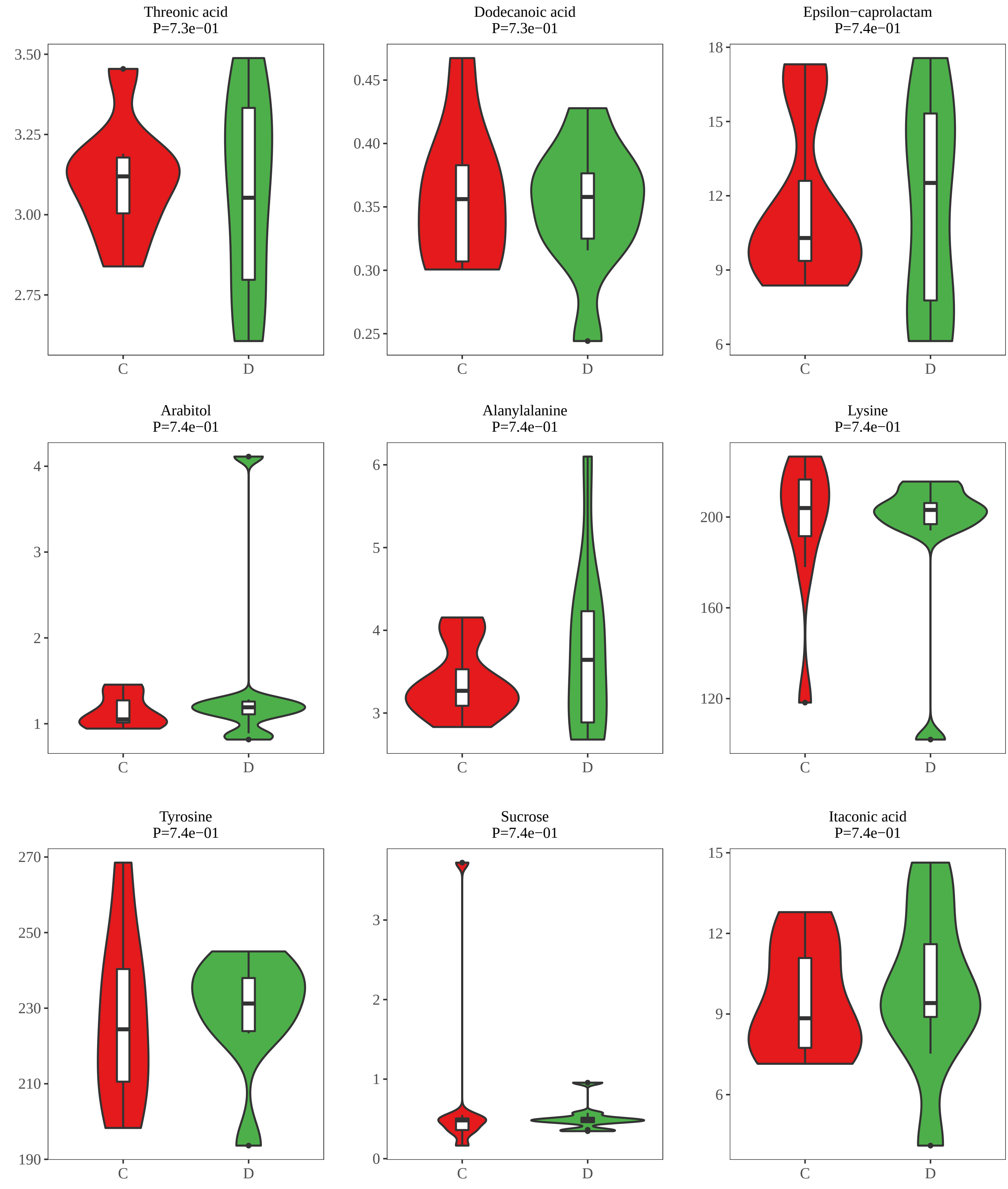
Threonic acid
P=7.3e−01
3.50
3.25
3.00
2.75
C
D
Dodecanoic acid
P=7.3e−01
0.45
0.40
0.35
0.30
0.25
C
D
Epsilon−caprolactam
P=7.4e−01
18
15
12
9
6
C
D
Arabitol
P=7.4e−01
4
3
2
1
C
D
Alanylalanine
P=7.4e−01
6
5
4
3
C
D
Lysine
P=7.4e−01
200
160
120
C
D
Tyrosine
P=7.4e−01
270
250
230
210
190
C
D
Sucrose
P=7.4e−01
3
2
1
0
C
D
Itaconic acid
P=7.4e−01
15
12
9
6
C
D

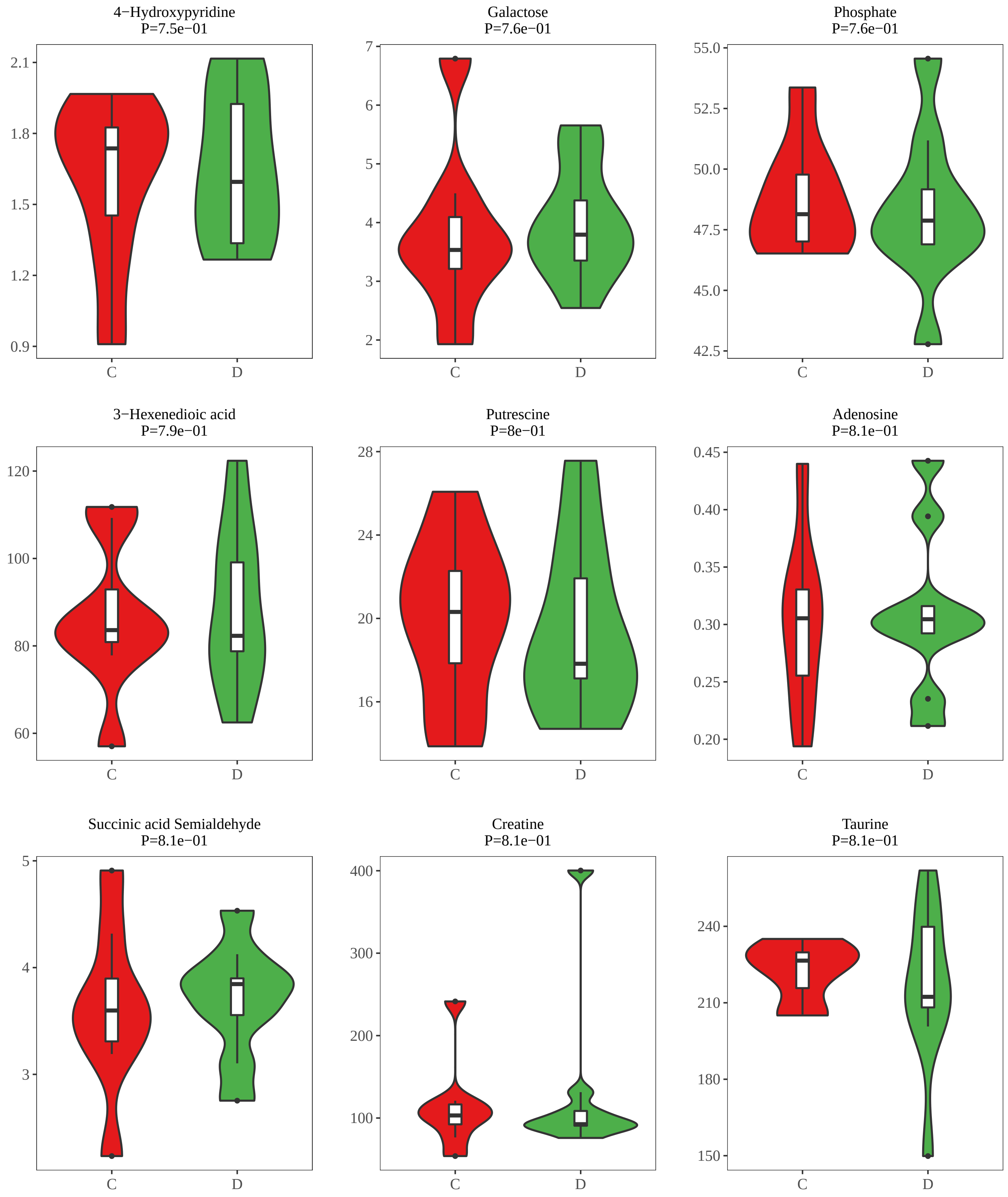
4−Hydroxypyridine
P=7.5e−01
Galactose
P=7.6e−01
Phosphate
P=7.6e−01
3−Hexenedioic acid
P=7.9e−01
Putrescine
P=8e−01
Adenosine
P=8.1e−01
Succinic acid Semialdehyde
P=8.1e−01
Creatine
P=8.1e−01
Taurine
P=8.1e−01
C
D

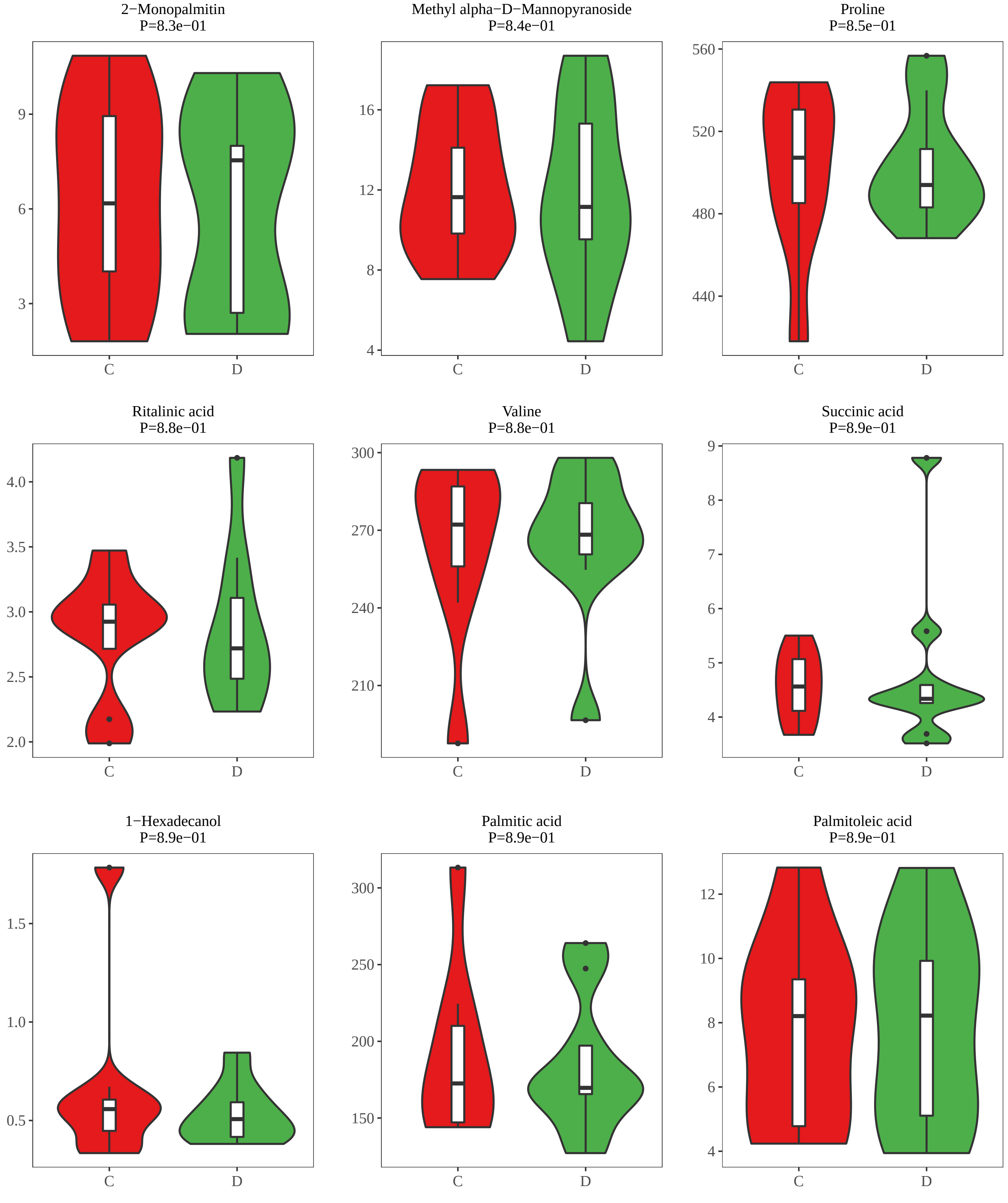

2−Monopalmitin
P=8.3e−01
3
6
9
C
D
Methyl alpha−D−Mannopyranoside
P=8.4e−01
4
8
12
16
C
D
Proline
P=8.5e−01
440
480
520
560
C
D
Ritalinic acid
P=8.8e−01
2.0
2.5
3.0
3.5
4.0
C
D
Valine
P=8.8e−01
210
240
270
300
C
D
Succinic acid
P=8.9e−01
4
5
6
7
8
9
C
D
1−Hexadecanol
P=8.9e−01
0.5
1.0
1.5
C
D
Palmitic acid
P=8.9e−01
150
200
250
300
C
D
Palmitoleic acid
P=8.9e−01
4
6
8
10
12
C
D

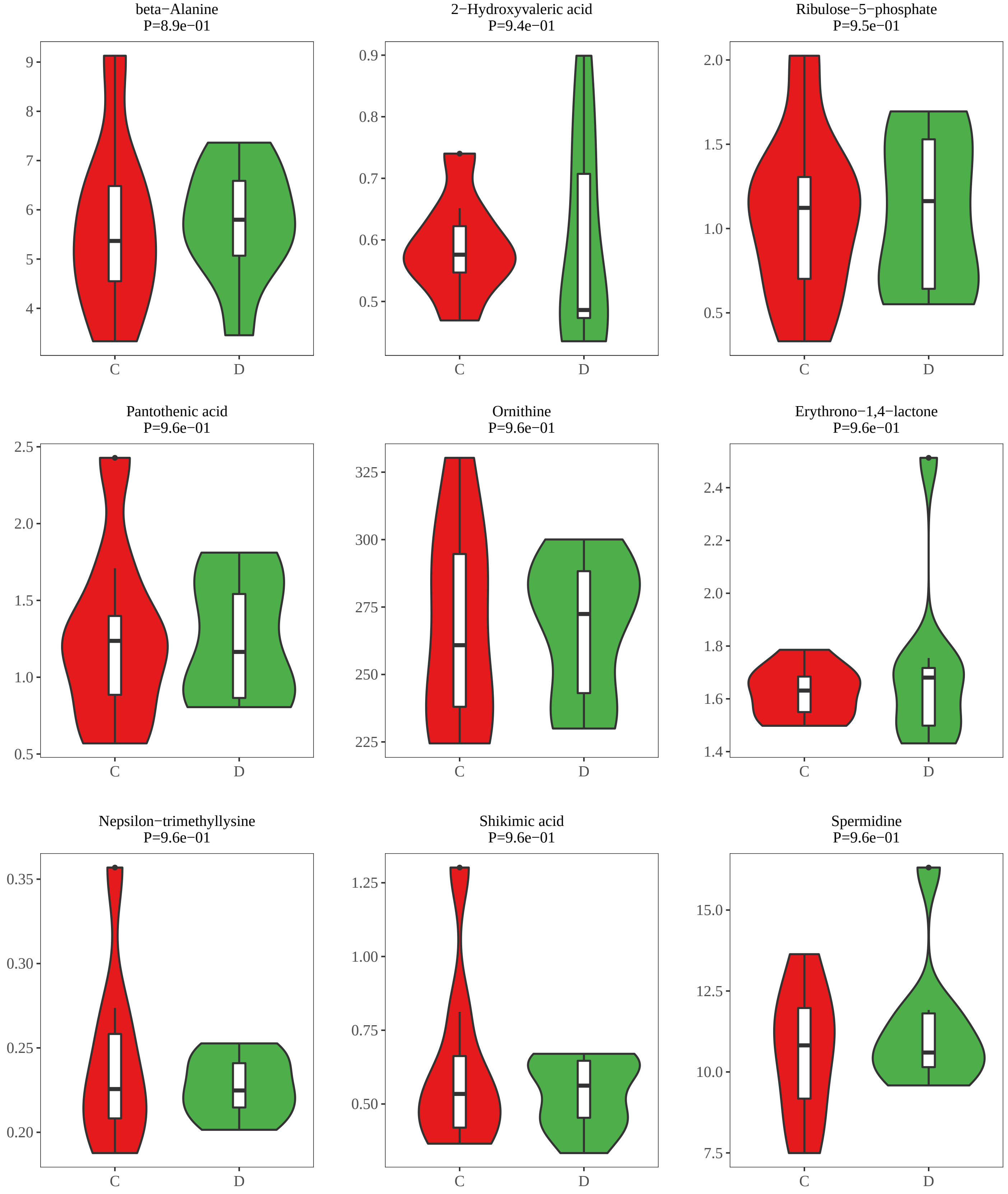
beta−Alanine
P=8.9e−01
9
8
7
6
5
4
C
D
2−Hydroxyvaleric acid
P=9.4e−01
0.9
0.8
0.7
0.6
0.5
C
D
Ribulose−5−phosphate
P=9.5e−01
2.0
1.5
1.0
0.5
C
D
Pantothenic acid
P=9.6e−01
2.5
2.0
1.5
1.0
0.5
C
D
Ornithine
P=9.6e−01
325
300
275
250
225
C
D
Erythrono−1,4−lactone
P=9.6e−01
2.4
2.2
2.0
1.8
1.6
1.4
C
D
Nepsilon−trimethyllysine
P=9.6e−01
0.35
0.30
0.25
0.20
C
D
Shikimic acid
P=9.6e−01
1.25
1.00
0.75
0.50
C
D
Spermidine
P=9.6e−01
15.0
12.5
10.0
7.5
C
D

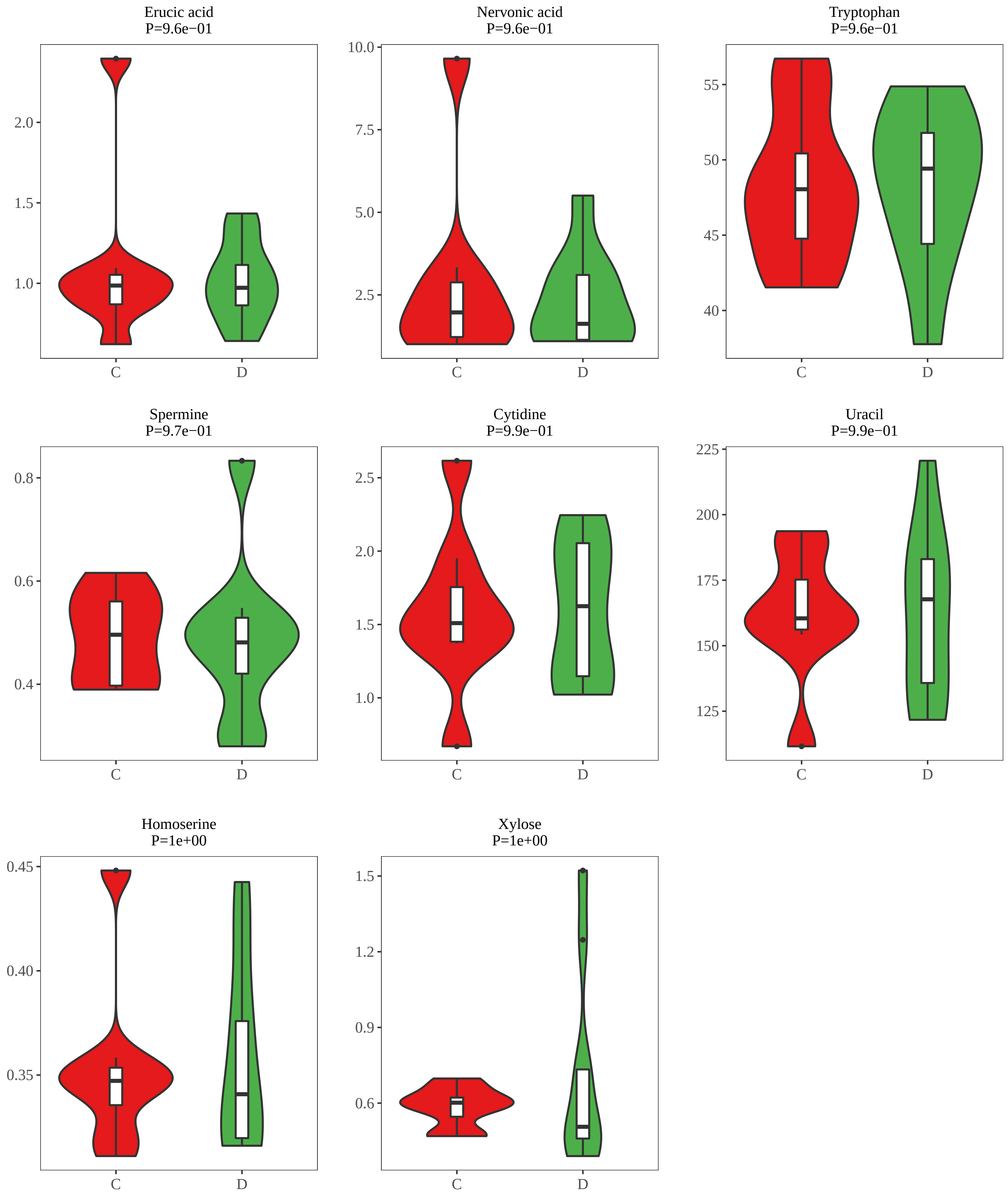

Erucic acid
P=9.6e−01
2.0
1.5
1.0
C
D
Nervonic acid
P=9.6e−01
10.0
7.5
5.0
2.5
C
D
Tryptophan
P=9.6e−01
55
50
45
40
C
D
Spermine
P=9.7e−01
0.8
0.6
0.4
C
D
Cytidine
P=9.9e−01
2.5
2.0
1.5
1.0
C
D
Uracil
P=9.9e−01
225
200
175
150
125
C
D
Homoserine
P=1e+00
0.45
0.40
0.35
C
D
Xylose
P=1e+00
1.5
1.2
0.9
0.6
C
D
