# Supplementary figures and images for: Yiai Fuzheng decoction inhibits triple-negative breast cancer by remodeling the immune microenvironment
Source: Front Immunol. 2025 Sep 30;16:1615631. doi: 10.3389/fimmu.2025.1615631 (PMC12518410; doi:10.3389/fimmu.2025.1615631)

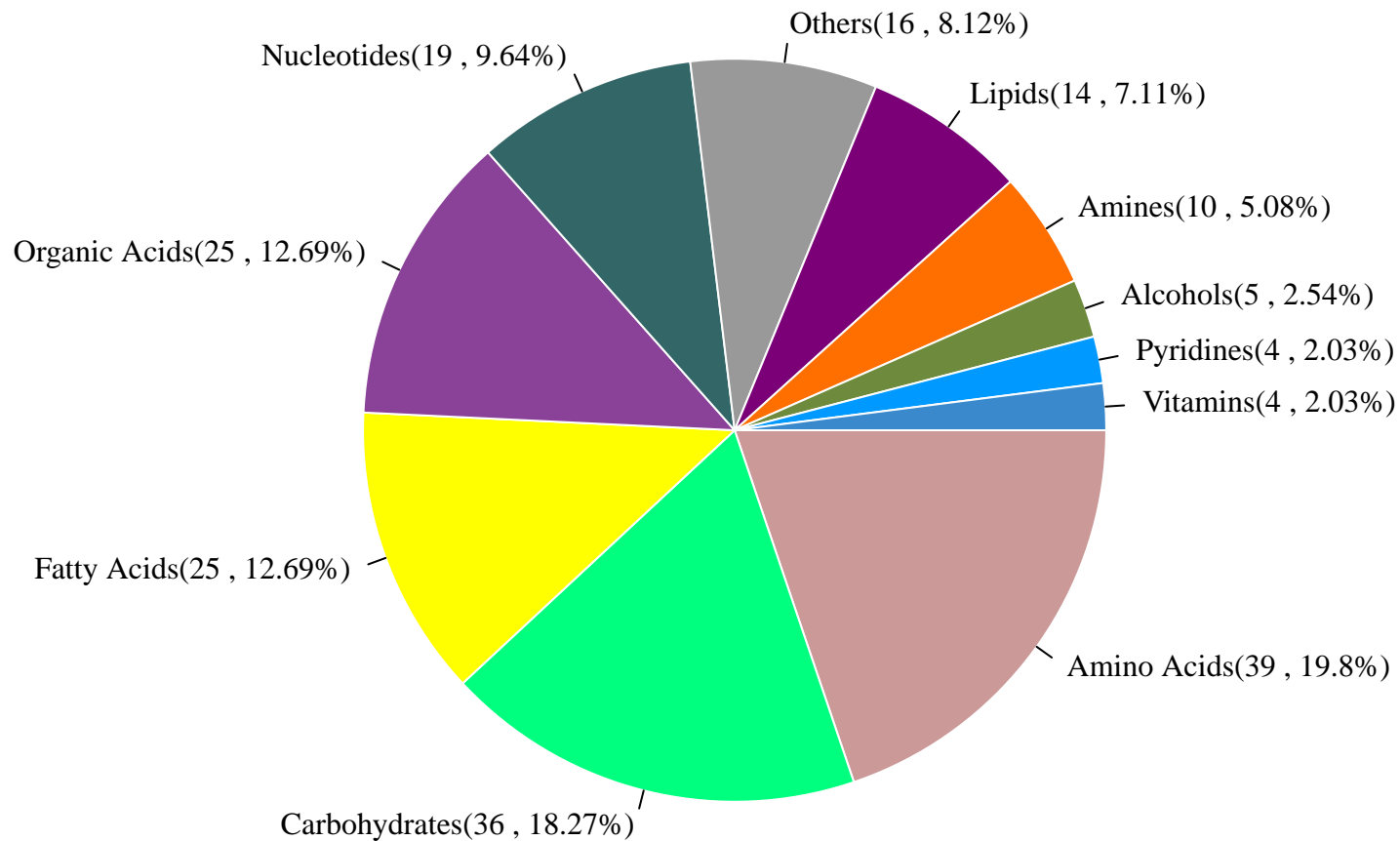

Supplement: Supplementary file 2 [file DataSheet1.zip › Supplementary File 2/Quality_Control/Class_Count_Pieplot.pdf]

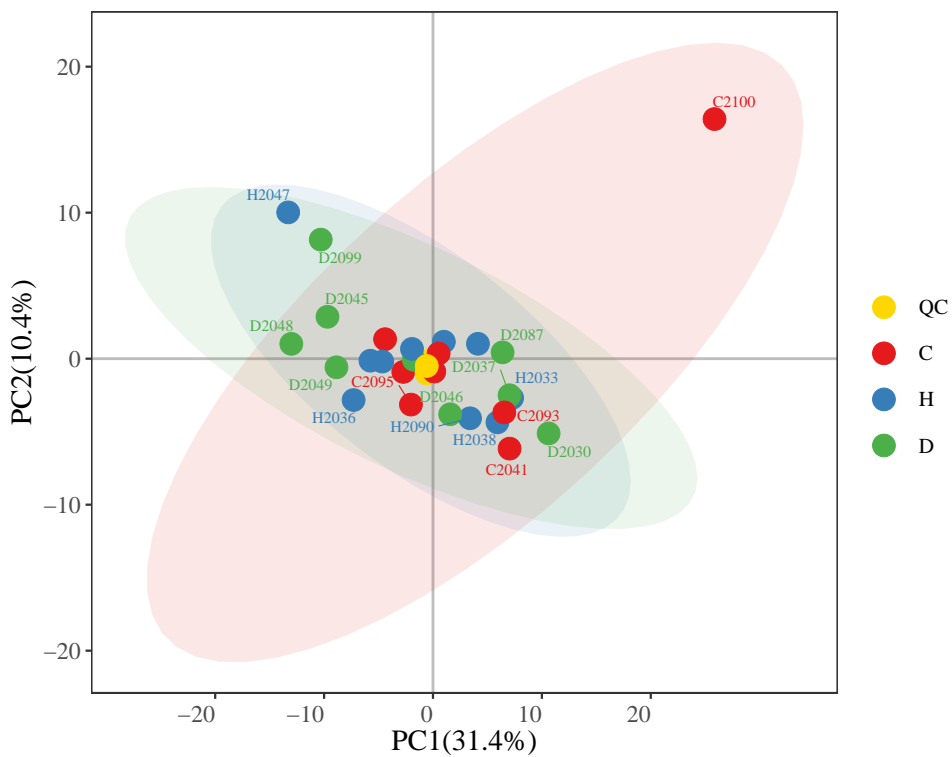

Supplement: Supplementary file 2 [file DataSheet1.zip › Supplementary File 2/Quality_Control/01_PC1_with_All_Samples/PCA_with_All_Samples_First2PCs_with_Labels.pdf]

PC1 scores/Outlier Detection

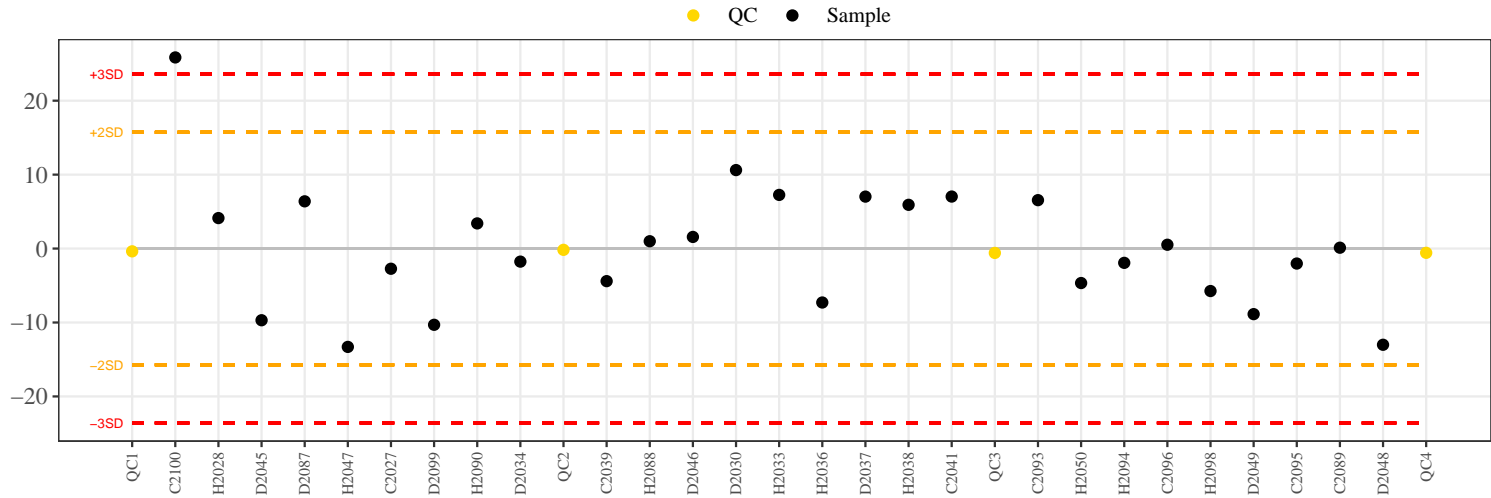

Supplement: Supplementary file 2 [file DataSheet1.zip › Supplementary File 2/Quality_Control/01_PC1_with_All_Samples/PC1_Dotplot.pdf]

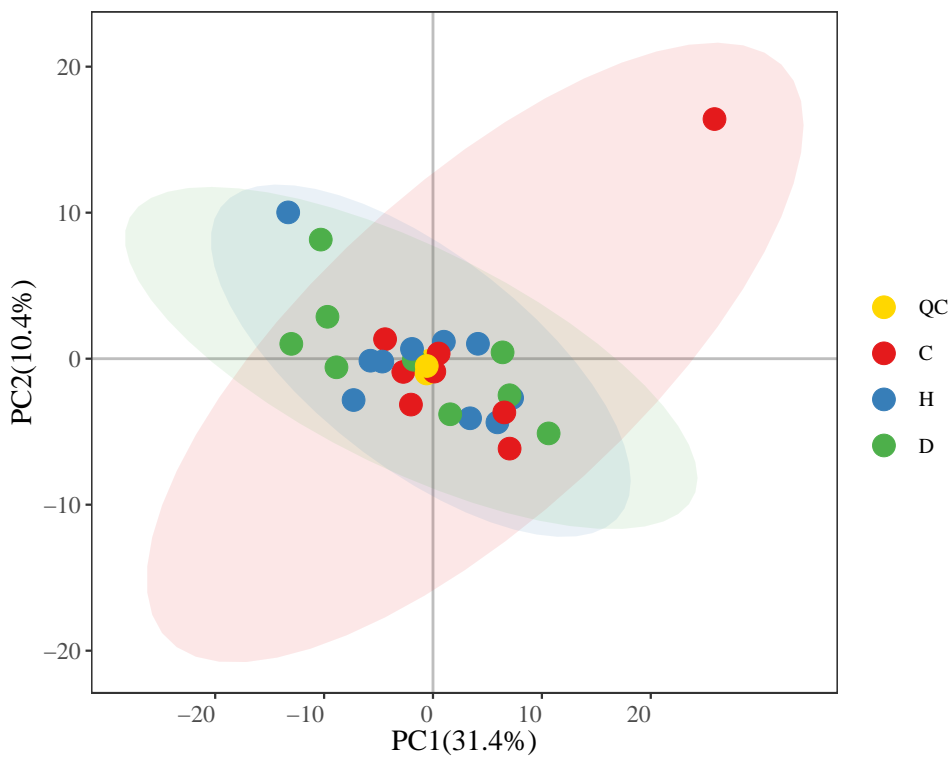

Supplement: Supplementary file 2 [file DataSheet1.zip › Supplementary File 2/Quality_Control/01_PC1_with_All_Samples/PCA_with_All_Samples_First2PCs.pdf]

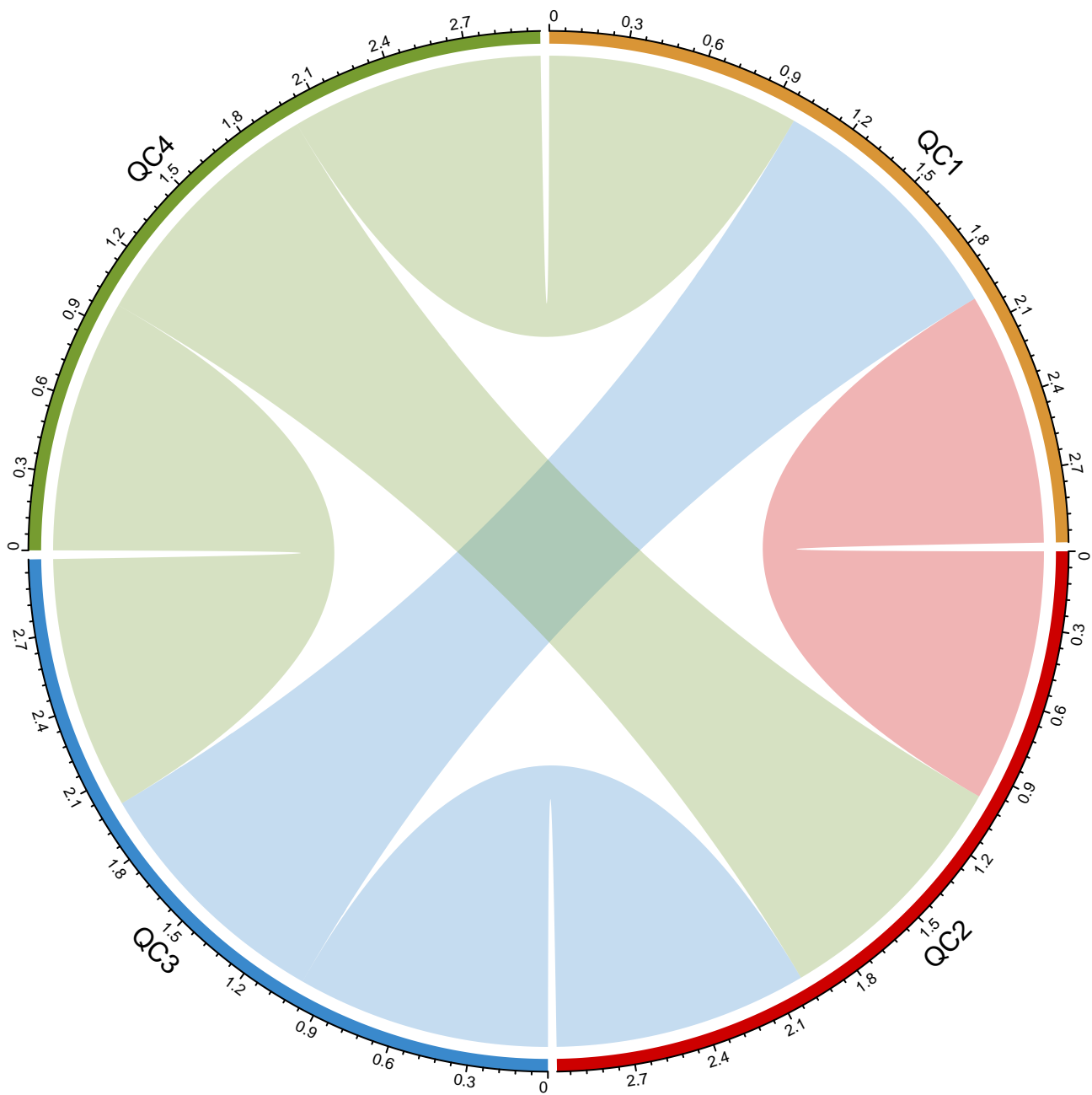

Supplement: Supplementary file 2 [file DataSheet1.zip › Supplementary File 2/Quality_Control/02_QC_Correlation_Pearson/QC_Pearson_Chord.pdf]

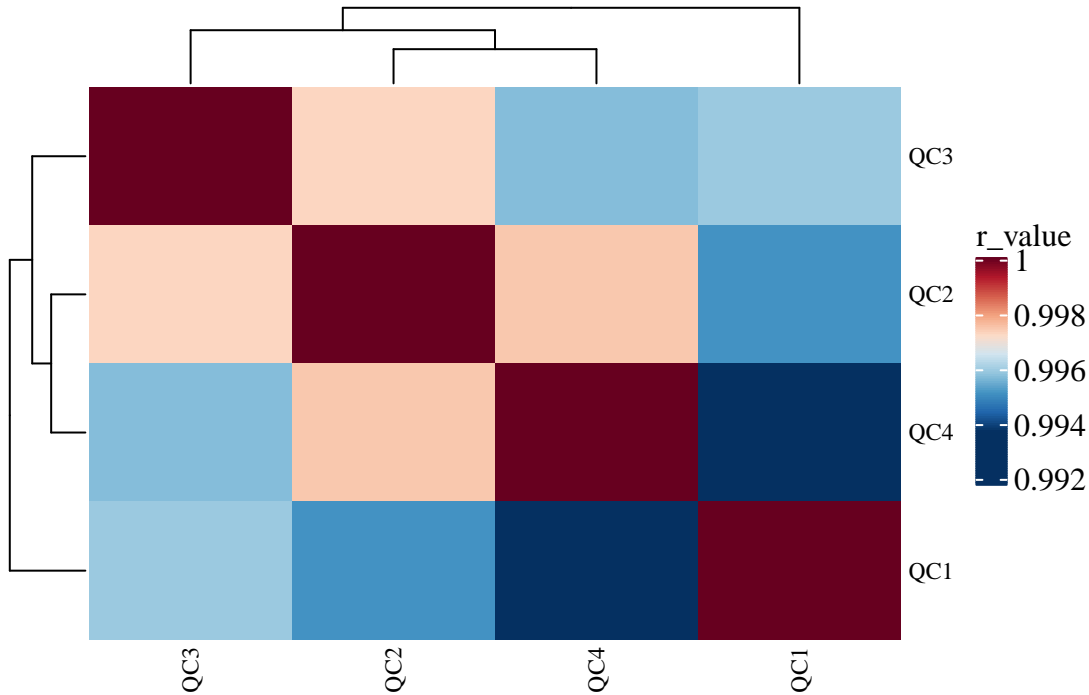

Supplement: Supplementary file 2 [file DataSheet1.zip › Supplementary File 2/Quality_Control/02_QC_Correlation_Pearson/QC_Pearson_Correlation_Heatmap.pdf]

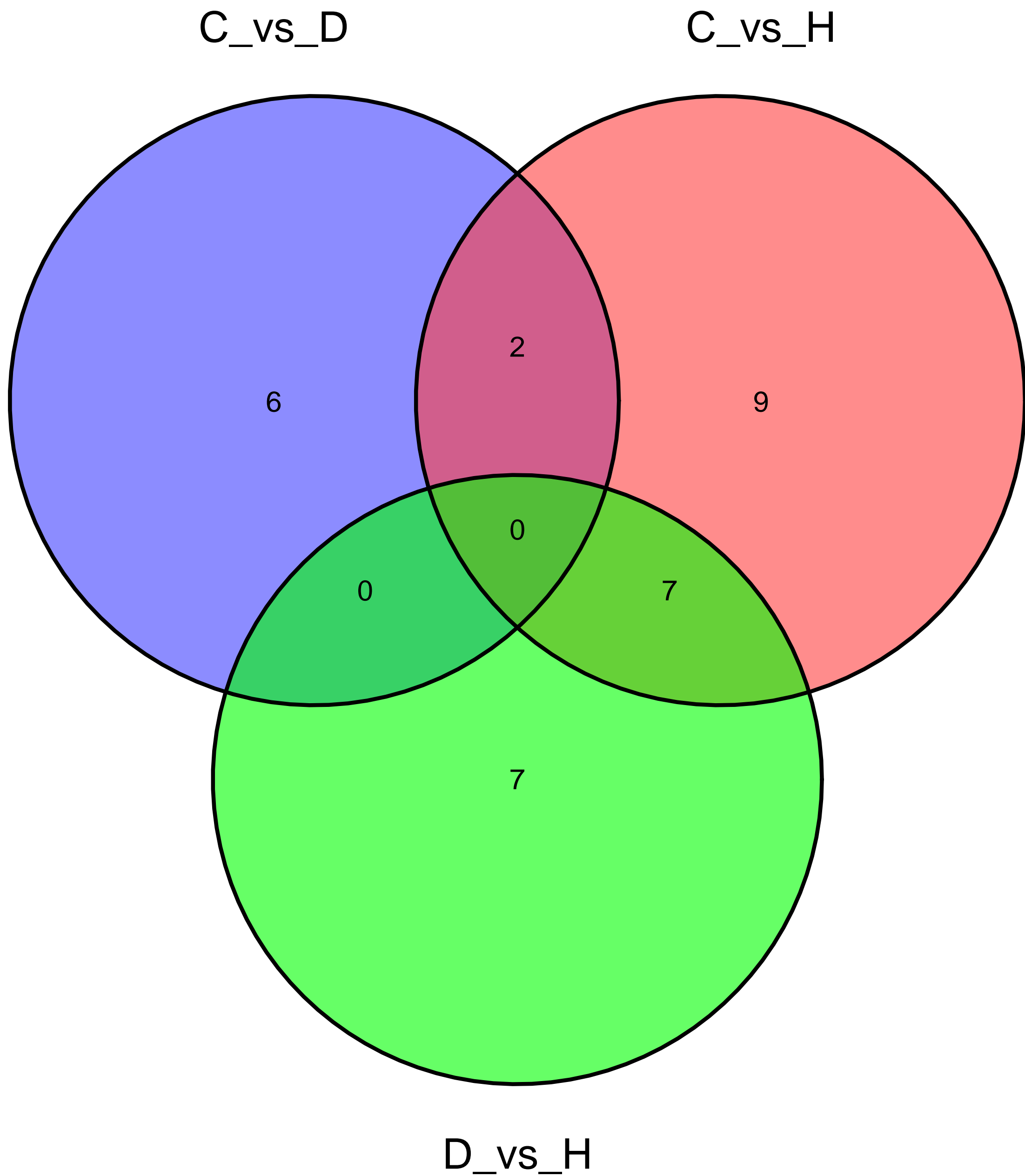

Supplement: Supplementary file 2 [file DataSheet1.zip › Supplementary File 2/Treatment/Sig_Met_Summary/Sig_Met_Venn_Plot_By_TwoGroup_Compares.pdf]

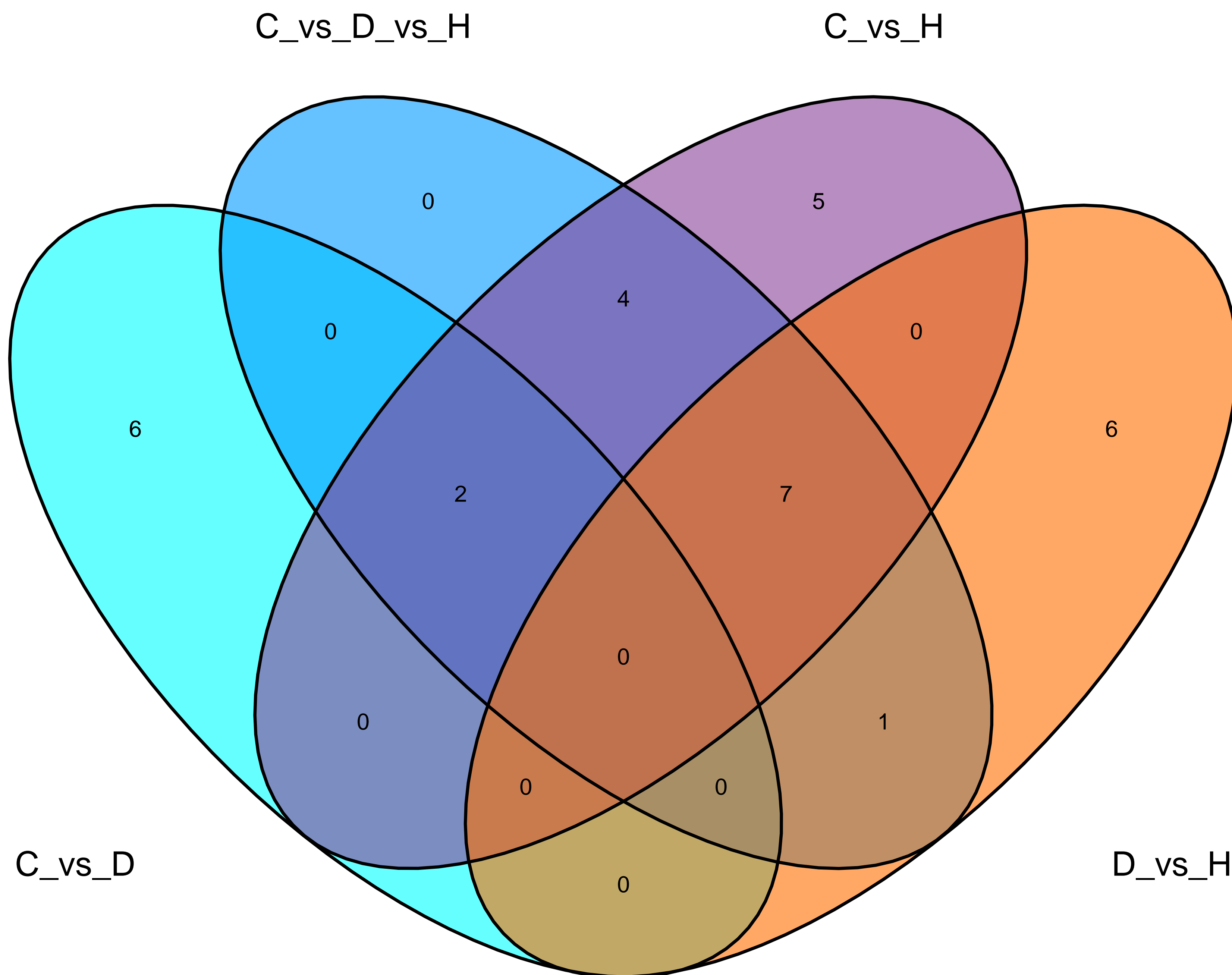

Supplement: Supplementary file 2 [file DataSheet1.zip › Supplementary File 2/Treatment/Sig_Met_Summary/Sig_Met_Venn_Plot.pdf]

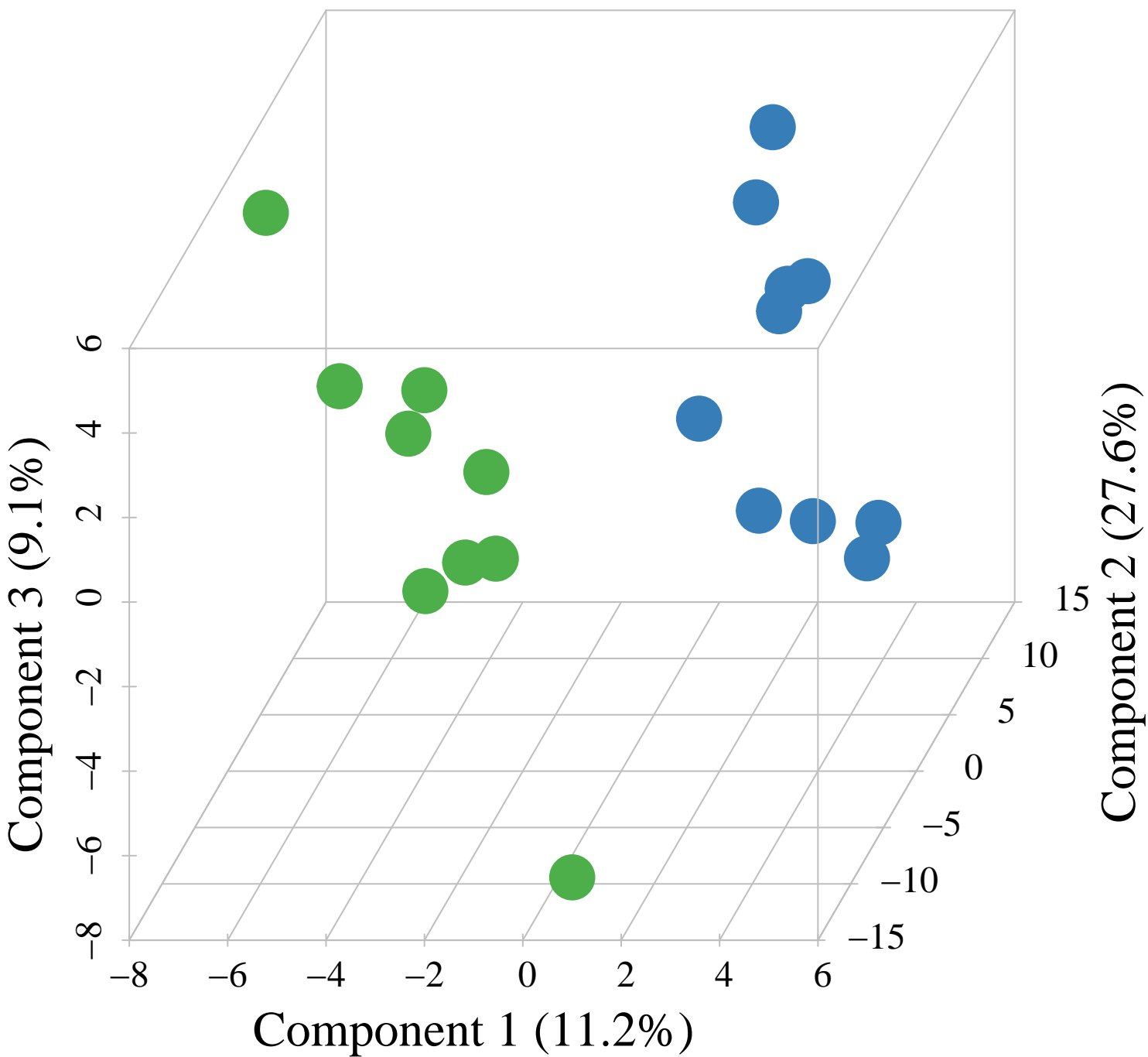

Supplement: Supplementary file 2 [file DataSheet1.zip › Supplementary File 2/Treatment/D_vs_H/03_PLS_DA/PC123_Score_3D.pdf]

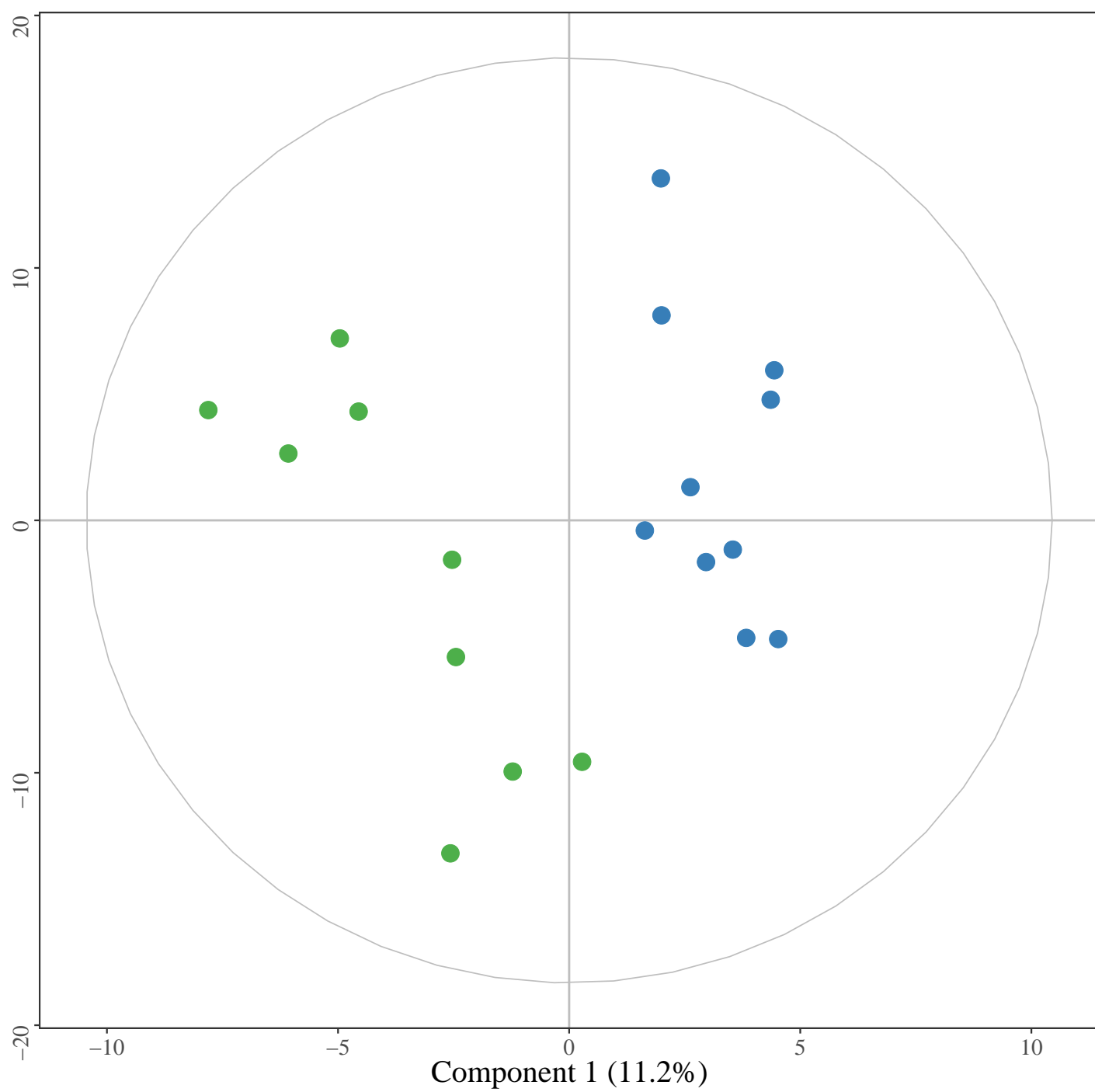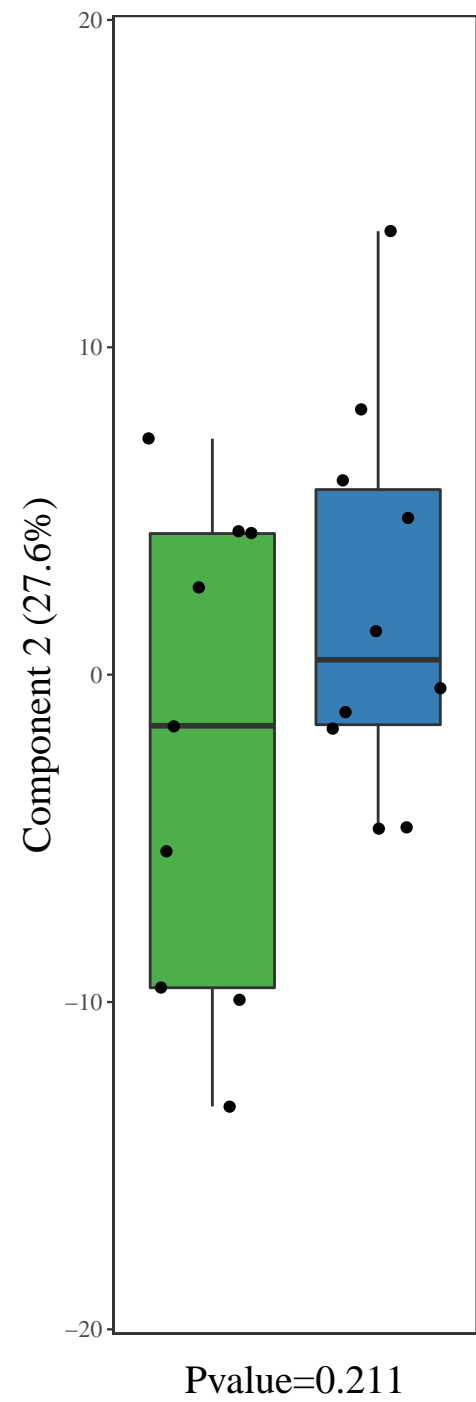

Pvalue=2.17e-05

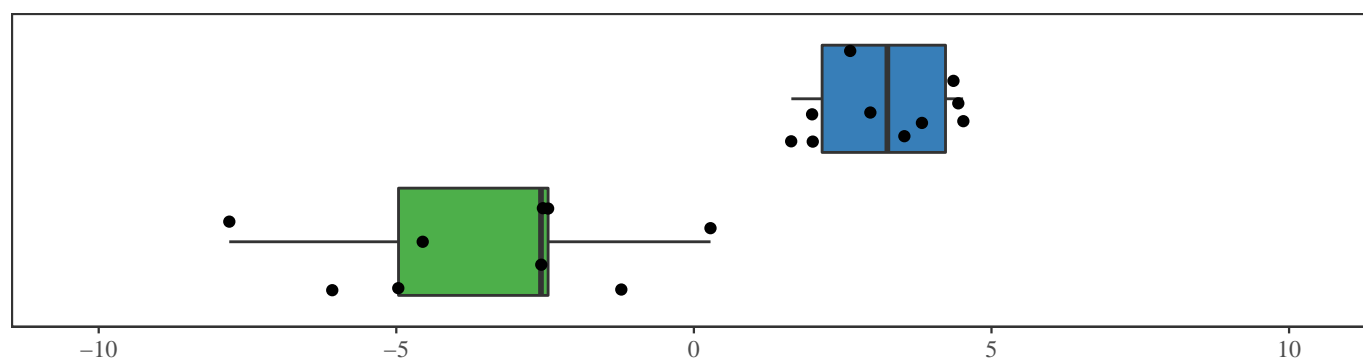

Supplement: Supplementary file 2 [file DataSheet1.zip › Supplementary File 2/Treatment/D_vs_H/03_PLS_DA/PLSDA_Score_with_Boxplot_with_Points.pdf]

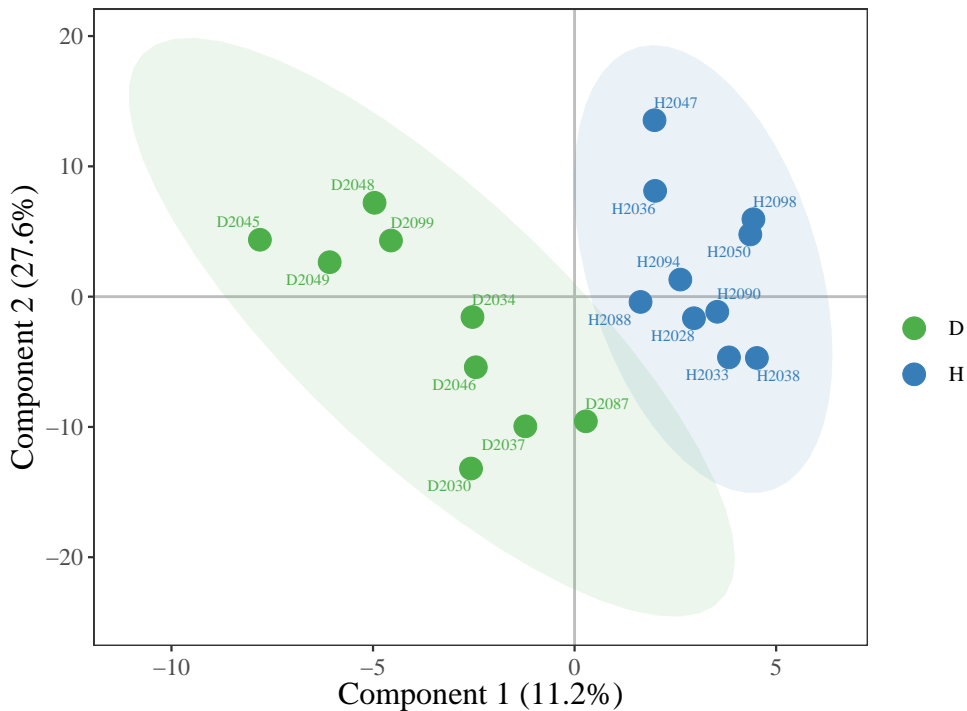

Supplement: Supplementary file 2 [file DataSheet1.zip › Supplementary File 2/Treatment/D_vs_H/03_PLS_DA/PLSDA_Score_2D_Label.pdf]

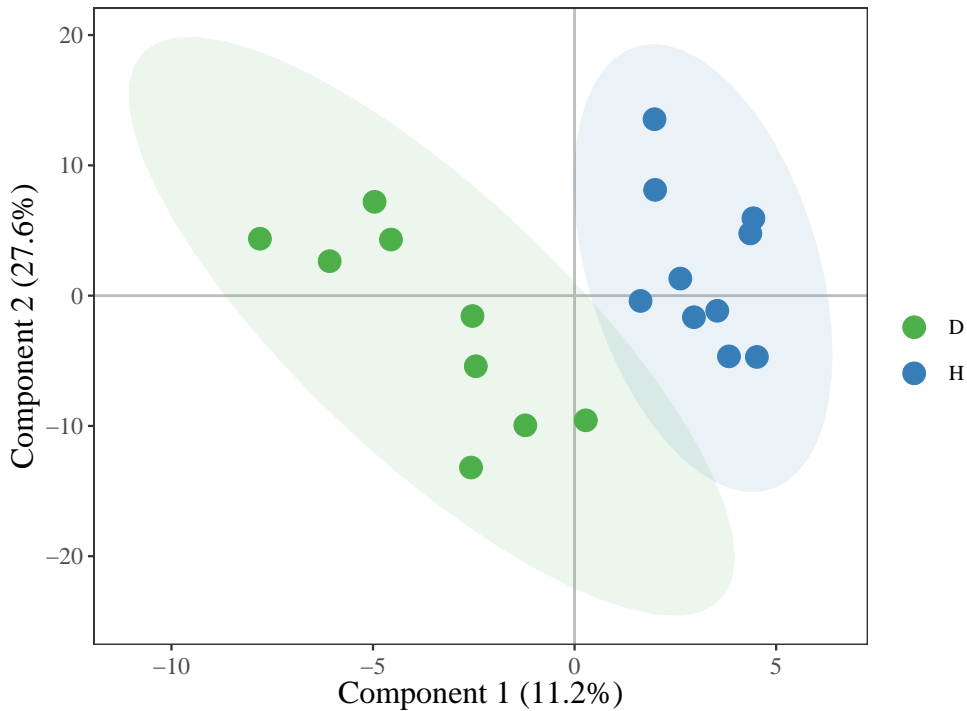

Supplement: Supplementary file 2 [file DataSheet1.zip › Supplementary File 2/Treatment/D_vs_H/03_PLS_DA/PLSDA_Score_2D.pdf]

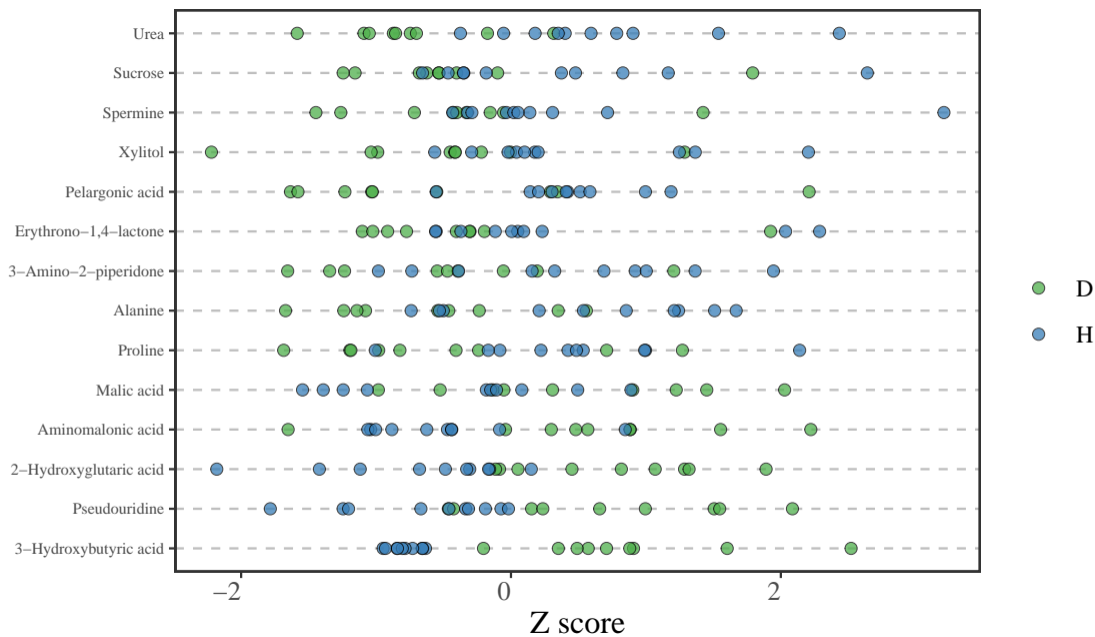

Supplement: Supplementary file 2 [file DataSheet1.zip › Supplementary File 2/Treatment/D_vs_H/06_Potential_Biomarkers/Markers_Z_Score_Plot.pdf]

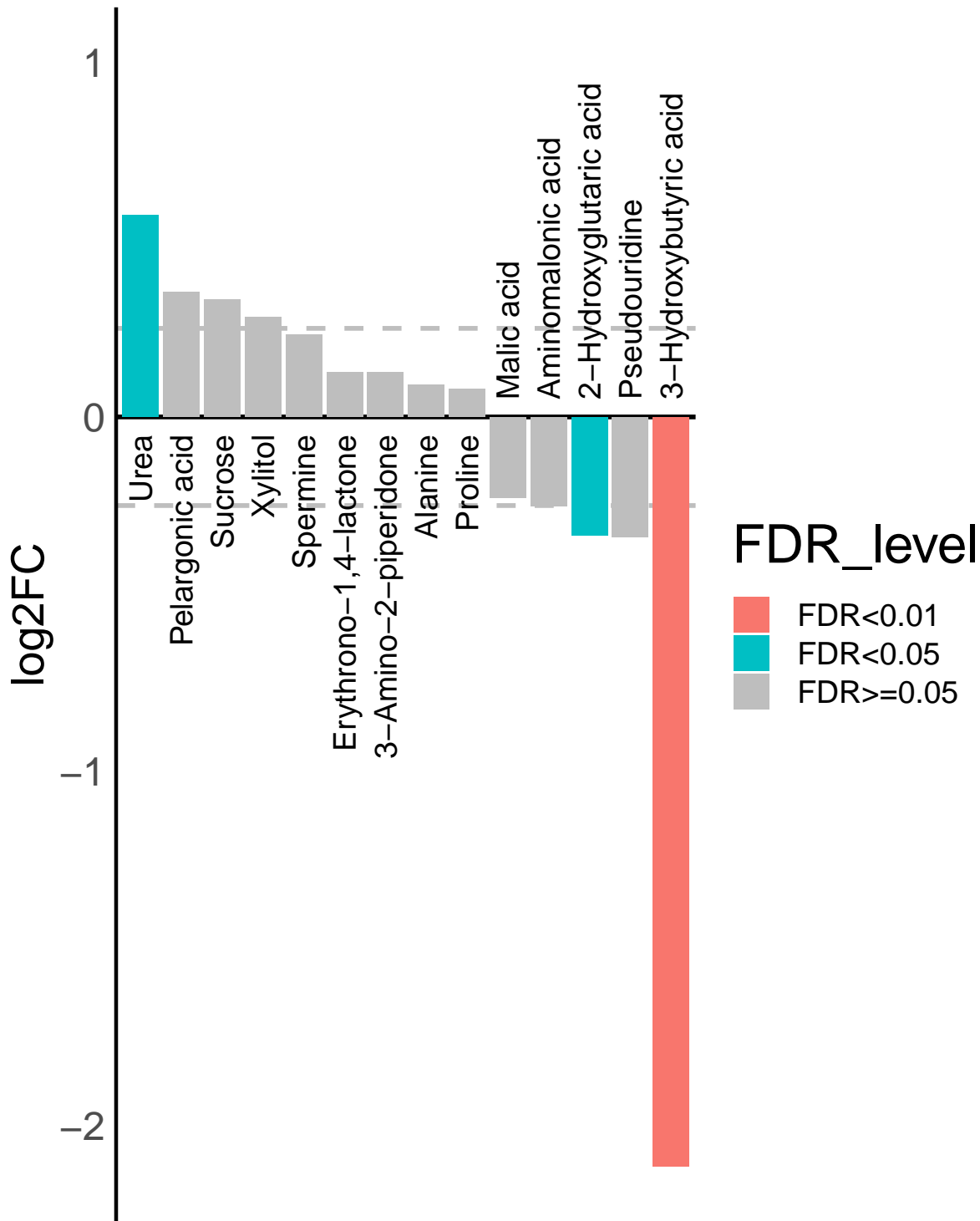

Supplement: Supplementary file 2 [file DataSheet1.zip › Supplementary File 2/Treatment/D_vs_H/06_Potential_Biomarkers/Markers_log2FC_FDR_Bar_Plot.pdf]

Univariate

OPLS-DA

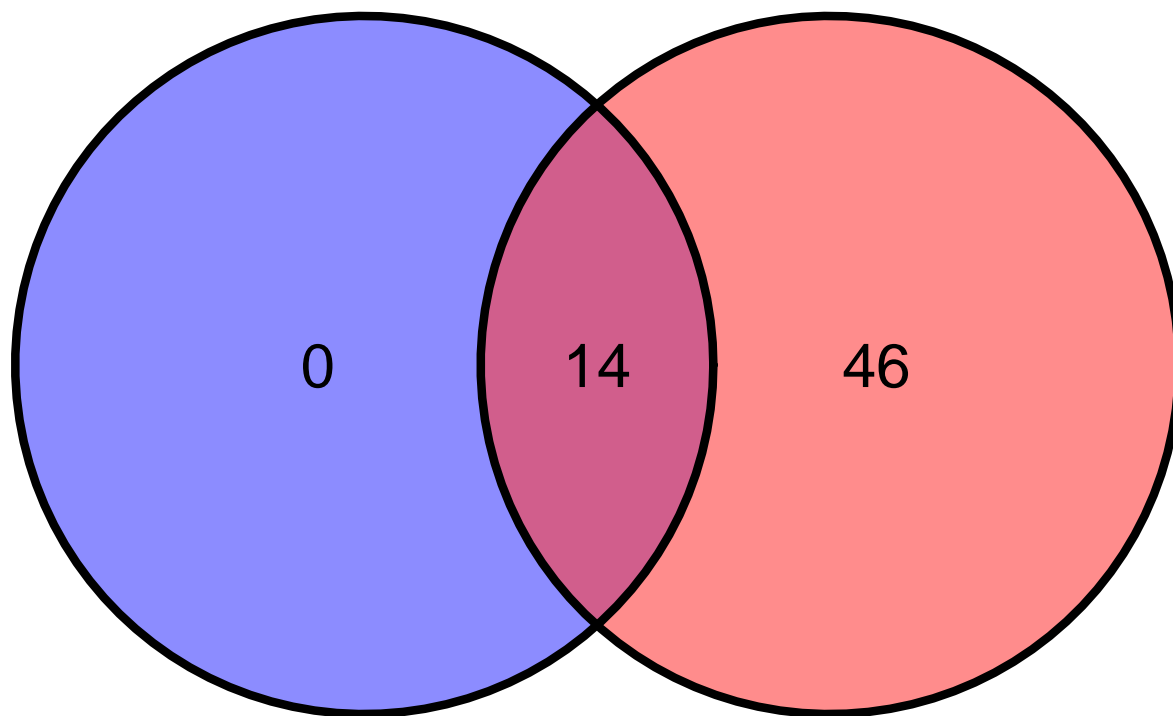

Supplement: Supplementary file 2 [file DataSheet1.zip › Supplementary File 2/Treatment/D_vs_H/06_Potential_Biomarkers/Venn_Plot.pdf]

D H

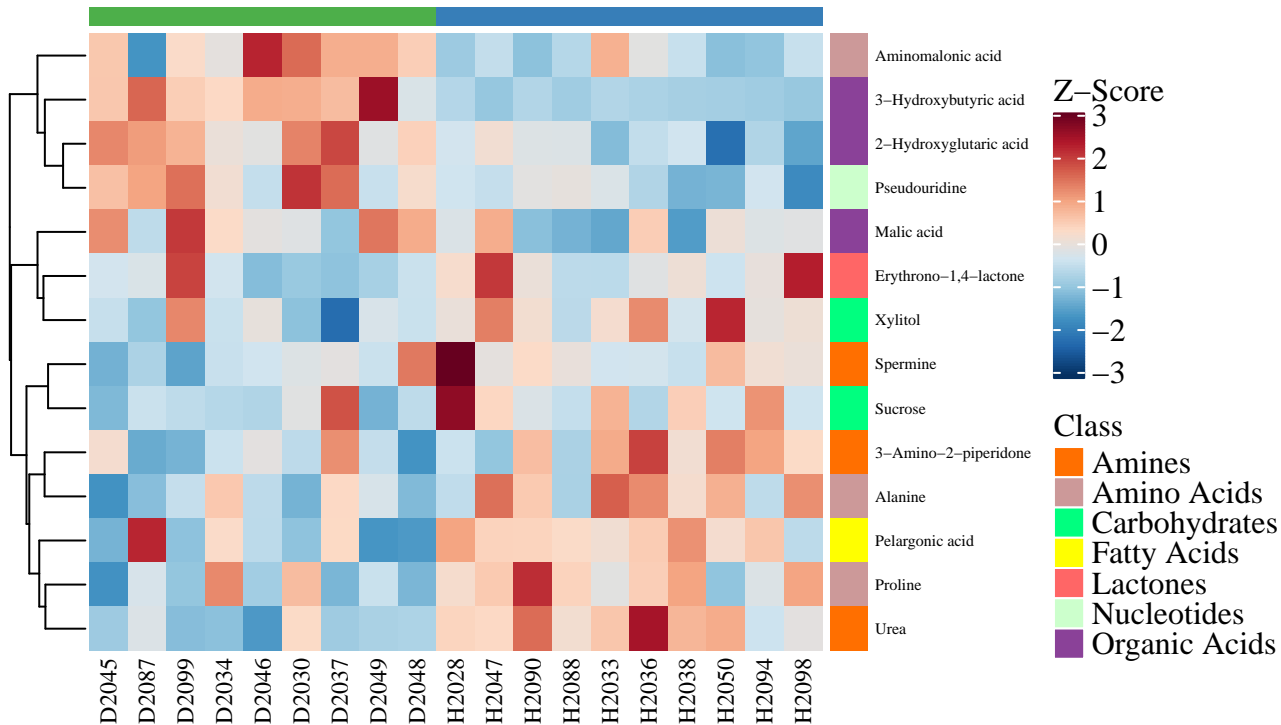

Supplement: Supplementary file 2 [file DataSheet1.zip › Supplementary File 2/Treatment/D_vs_H/06_Potential_Biomarkers/Markers_Z_Score_Heatmap_with_Name.pdf]

D H

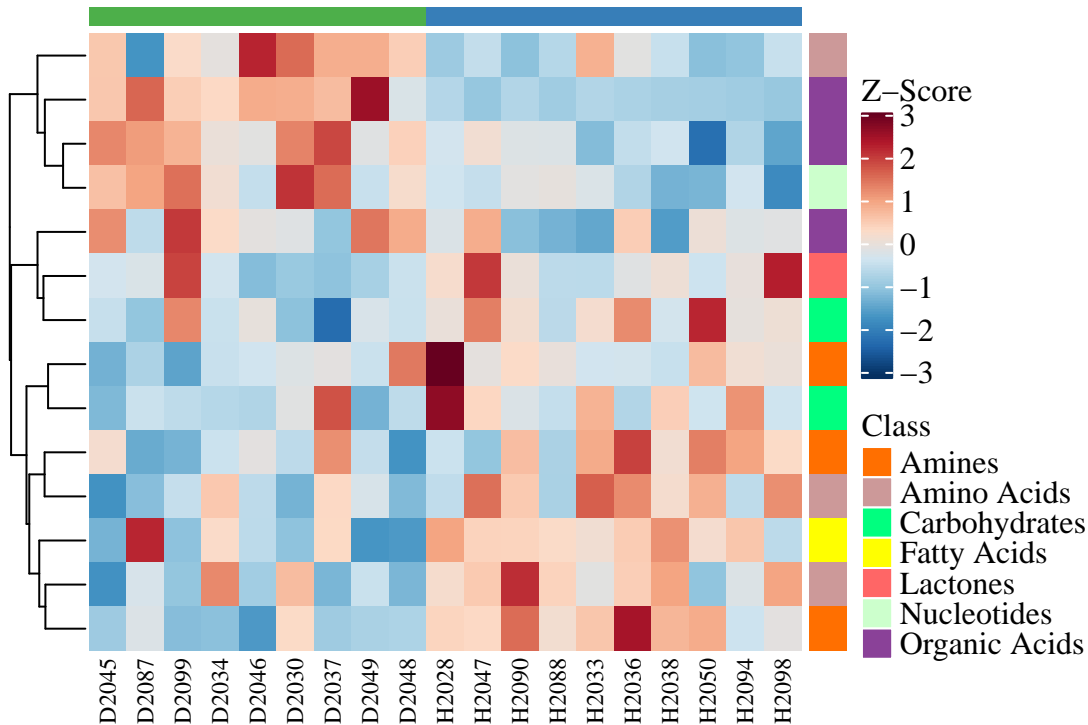

Supplement: Supplementary file 2 [file DataSheet1.zip › Supplementary File 2/Treatment/D_vs_H/06_Potential_Biomarkers/Markers_Z_Score_Heatmap.pdf]

D H

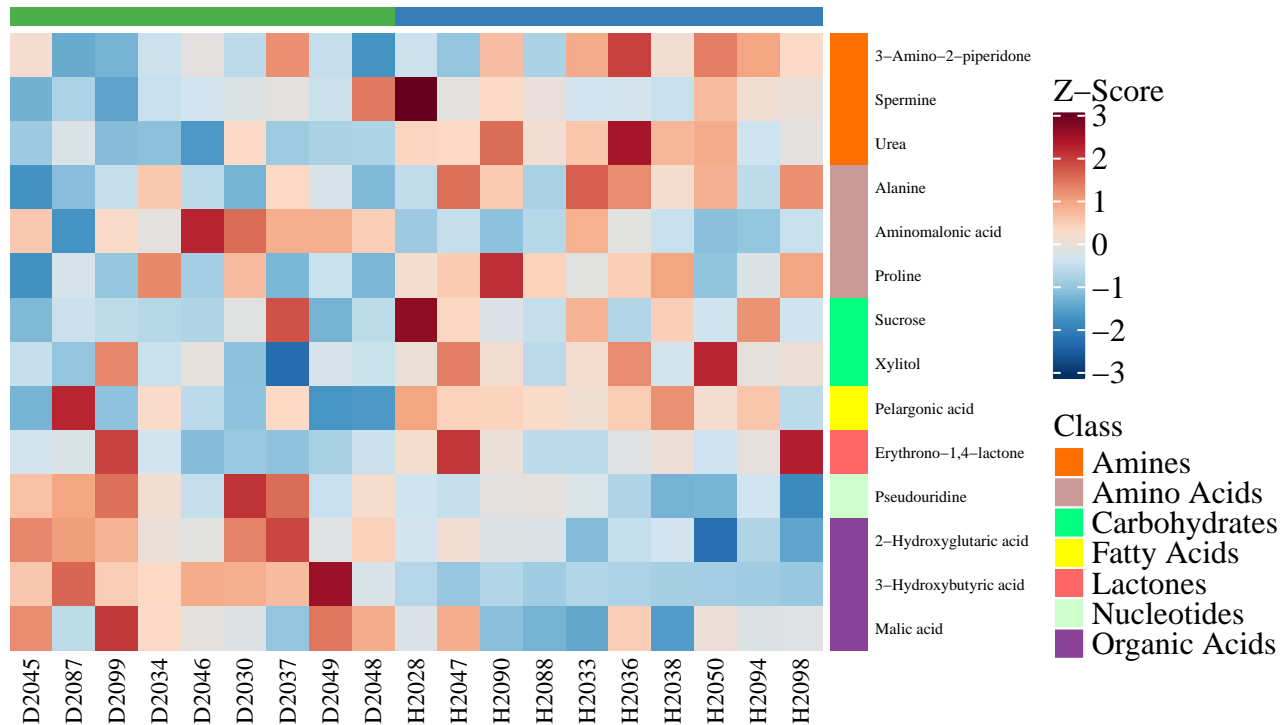

Supplement: Supplementary file 2 [file DataSheet1.zip › Supplementary File 2/Treatment/D_vs_H/06_Potential_Biomarkers/Markers_Z_Score_Heatmap_ordered_by_Class_with_Name.pdf]

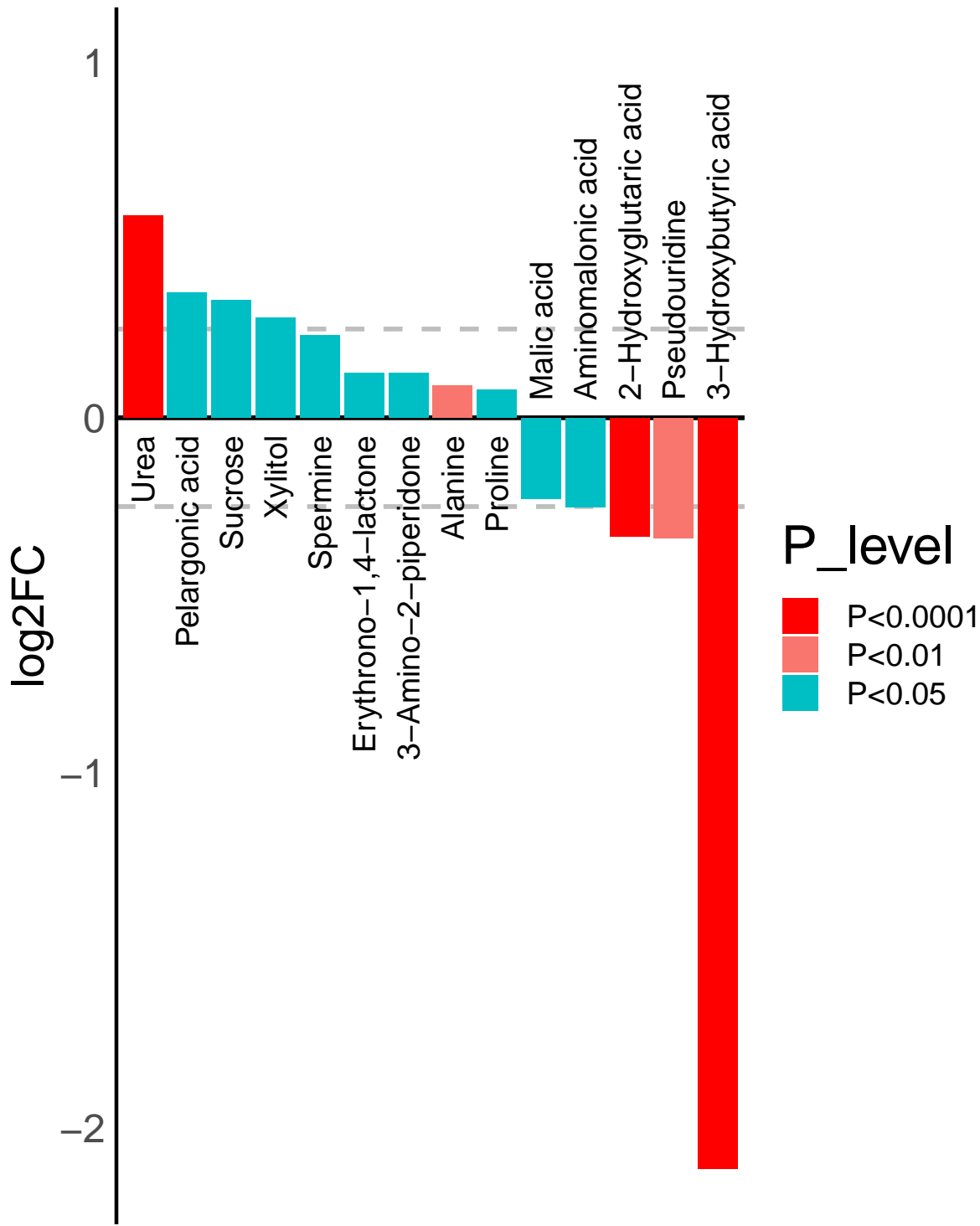

Supplement: Supplementary file 2 [file DataSheet1.zip › Supplementary File 2/Treatment/D_vs_H/06_Potential_Biomarkers/Markers_log2FC_P_Bar_Plot.pdf]

O1 (31.6%)

20  
10  
0  
-10  
-20

-8

-4

0

4

P1 (7.28%)

D2030  
D2037  
D2087  
D2046  
D2034  
D2045  
D2049  
D2099  
D2048

H2038  
H2033  
H2028  
H2088  
H2090  
H2094  
H2036  
H2050  
H2047  
H2098

● D  
● H

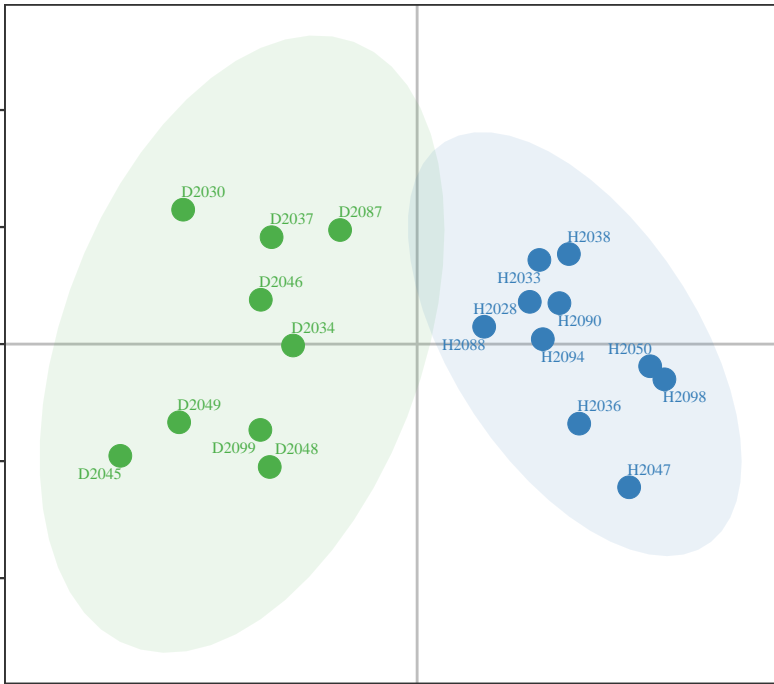

Supplement: Supplementary file 2 [file DataSheet1.zip › Supplementary File 2/Treatment/D_vs_H/04_OPLS_DA/OPLSDA_Score_2D_Label.pdf]

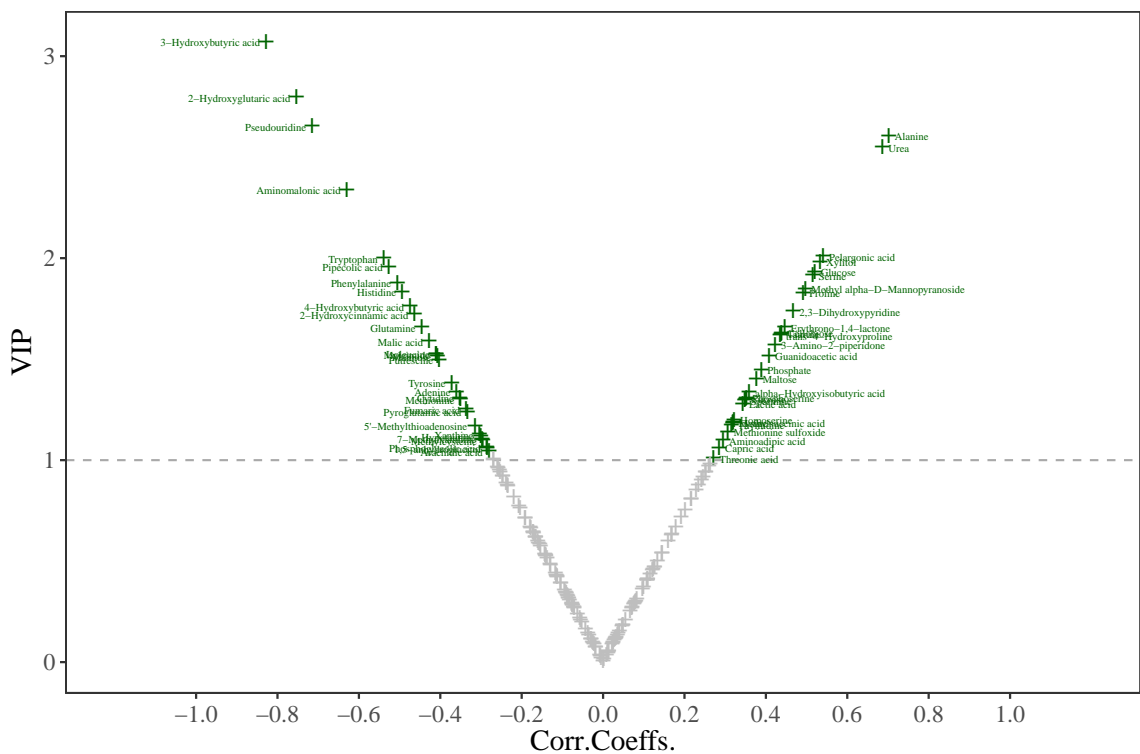

Supplement: Supplementary file 2 [file DataSheet1.zip › Supplementary File 2/Treatment/D_vs_H/04_OPLS_DA/OPLSDA_VPlot.pdf]

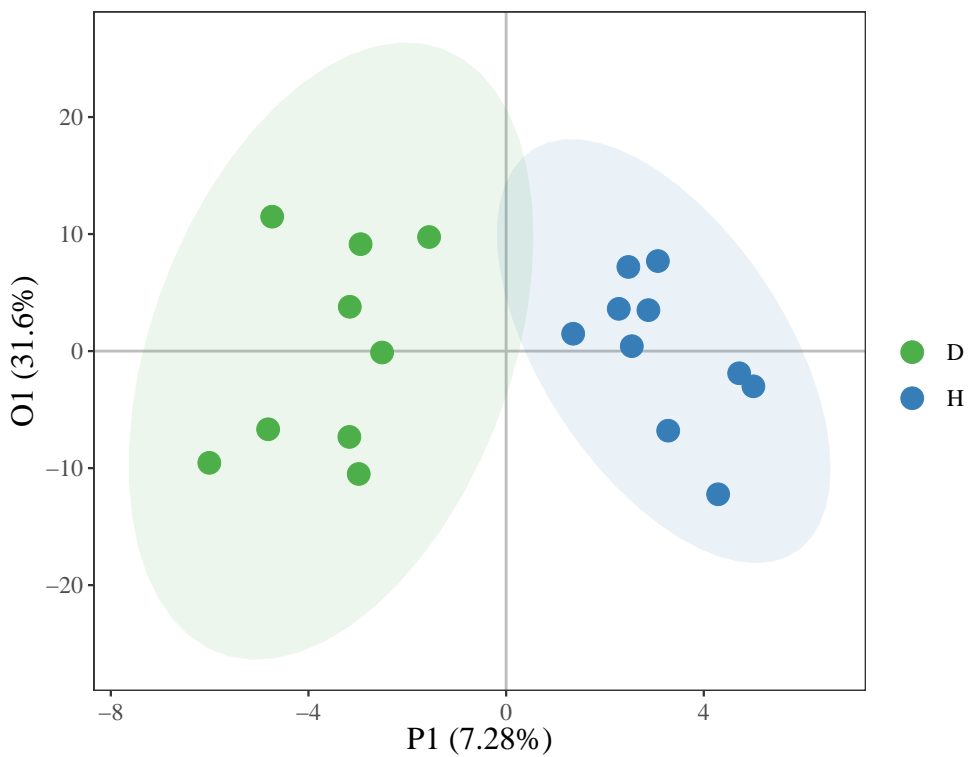

Supplement: Supplementary file 2 [file DataSheet1.zip › Supplementary File 2/Treatment/D_vs_H/04_OPLS_DA/OPLSDA_Score_2D.pdf]

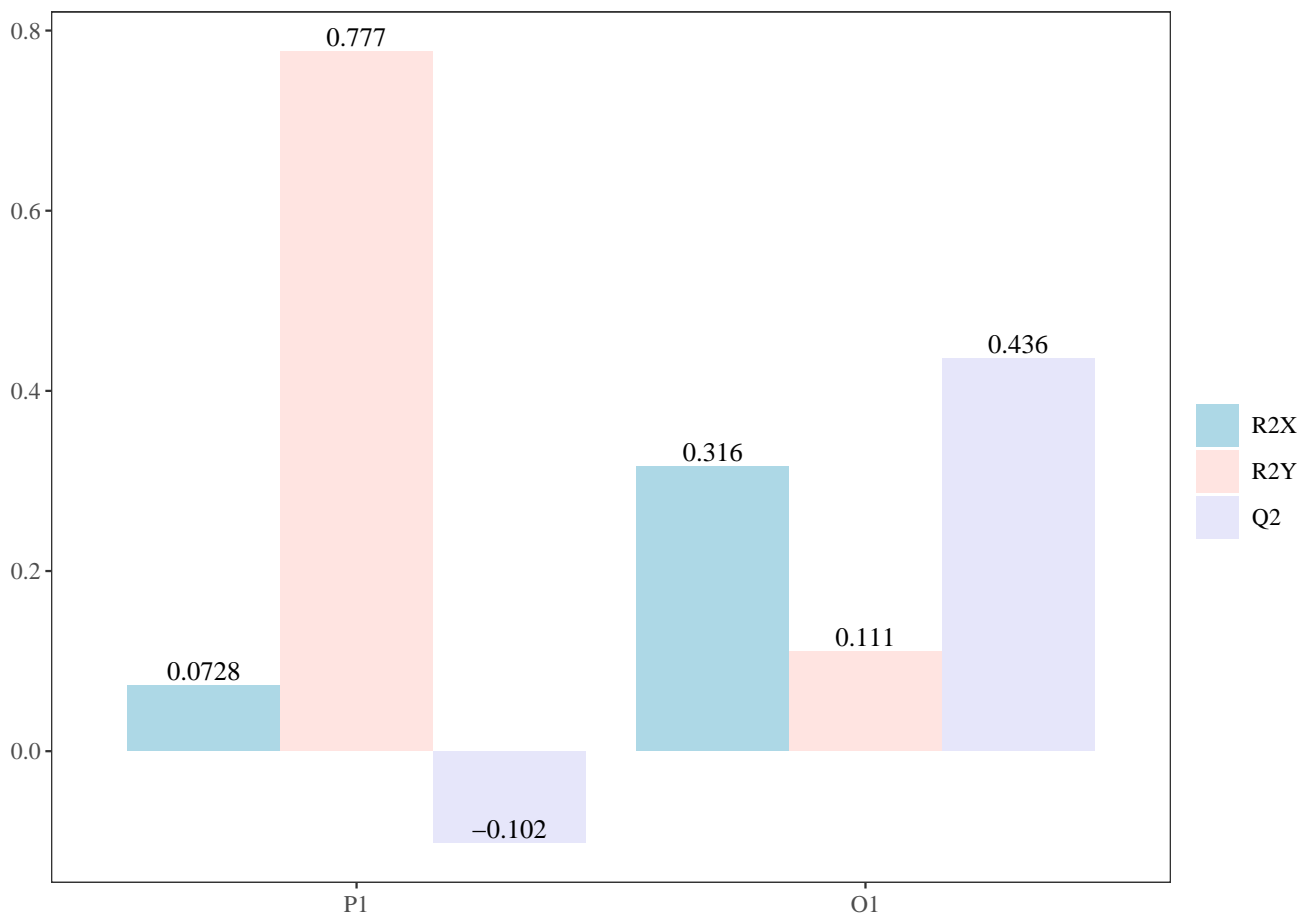

Supplement: Supplementary file 2 [file DataSheet1.zip › Supplementary File 2/Treatment/D_vs_H/04_OPLS_DA/OPLSDA_R2X_R2Y_Q2.pdf]

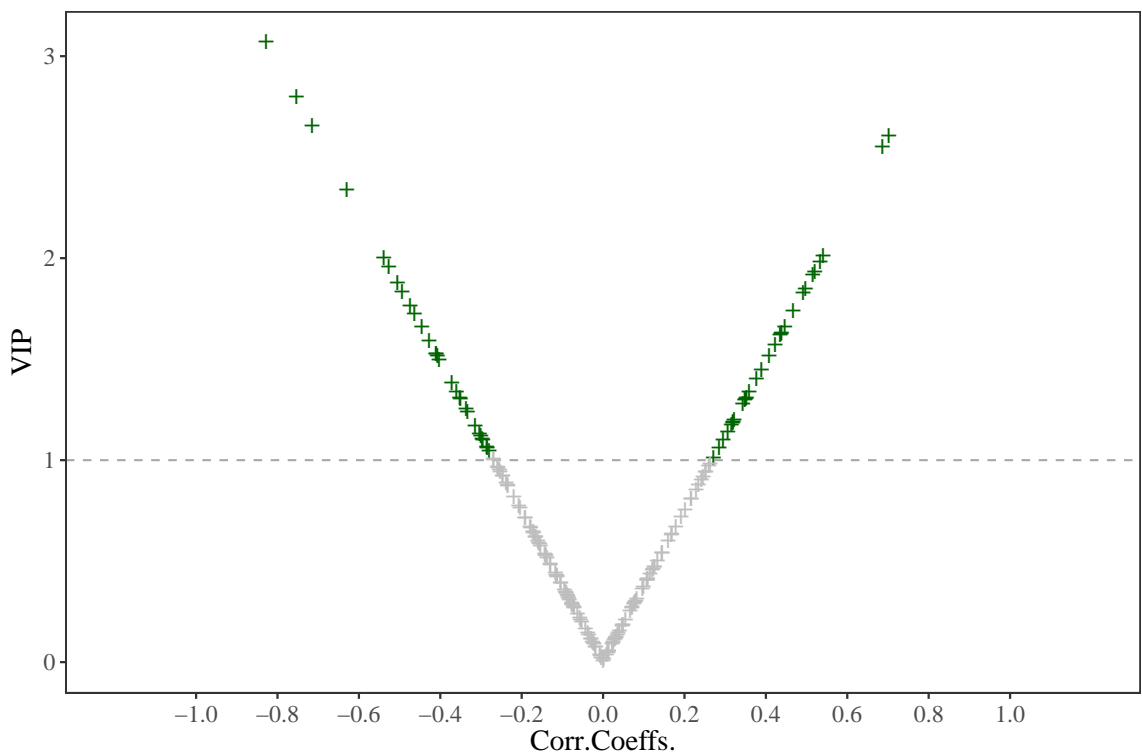

Supplement: Supplementary file 2 [file DataSheet1.zip › Supplementary File 2/Treatment/D_vs_H/04_OPLS_DA/OPLSDA_VPlot_without_Label.pdf]

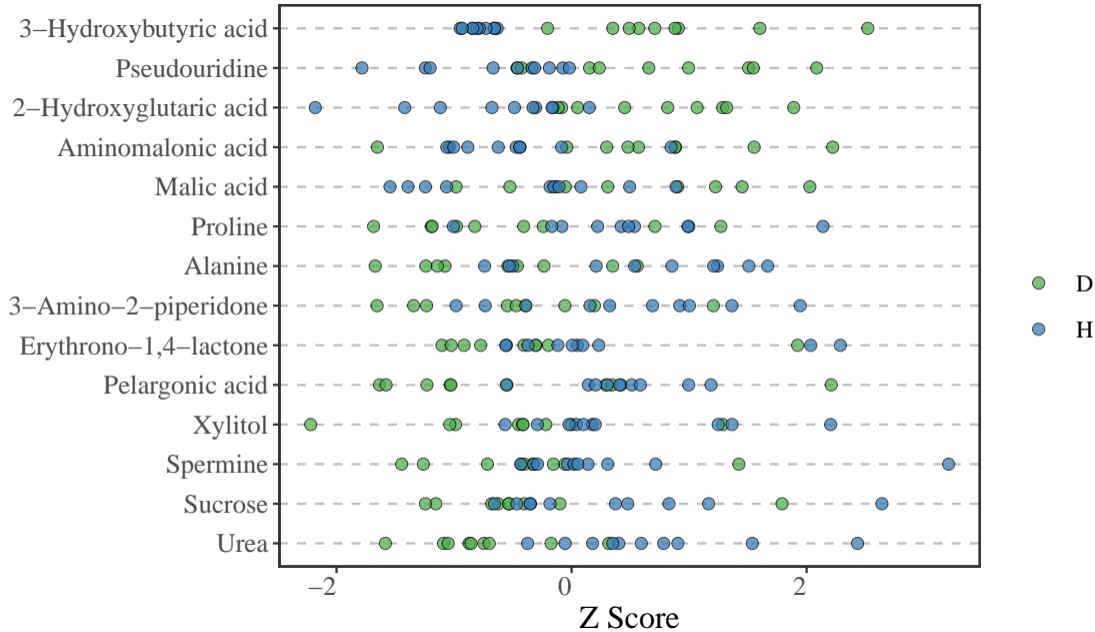

Supplement: Supplementary file 2 [file DataSheet1.zip › Supplementary File 2/Treatment/D_vs_H/05_Univariate_Analysis/Z_Score_Plot.pdf]

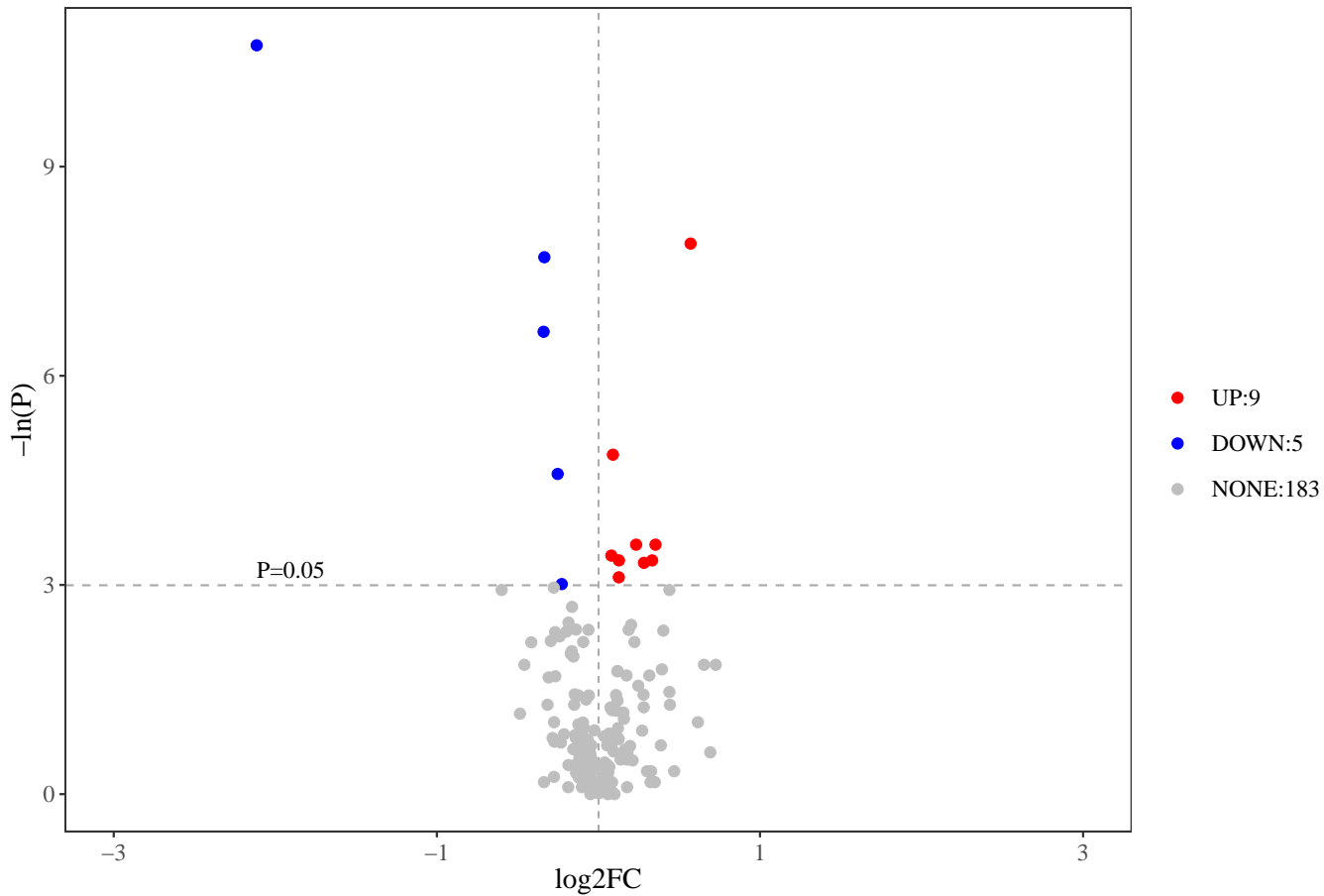

Supplement: Supplementary file 2 [file DataSheet1.zip › Supplementary File 2/Treatment/D_vs_H/05_Univariate_Analysis/Volcano_Plot.pdf]

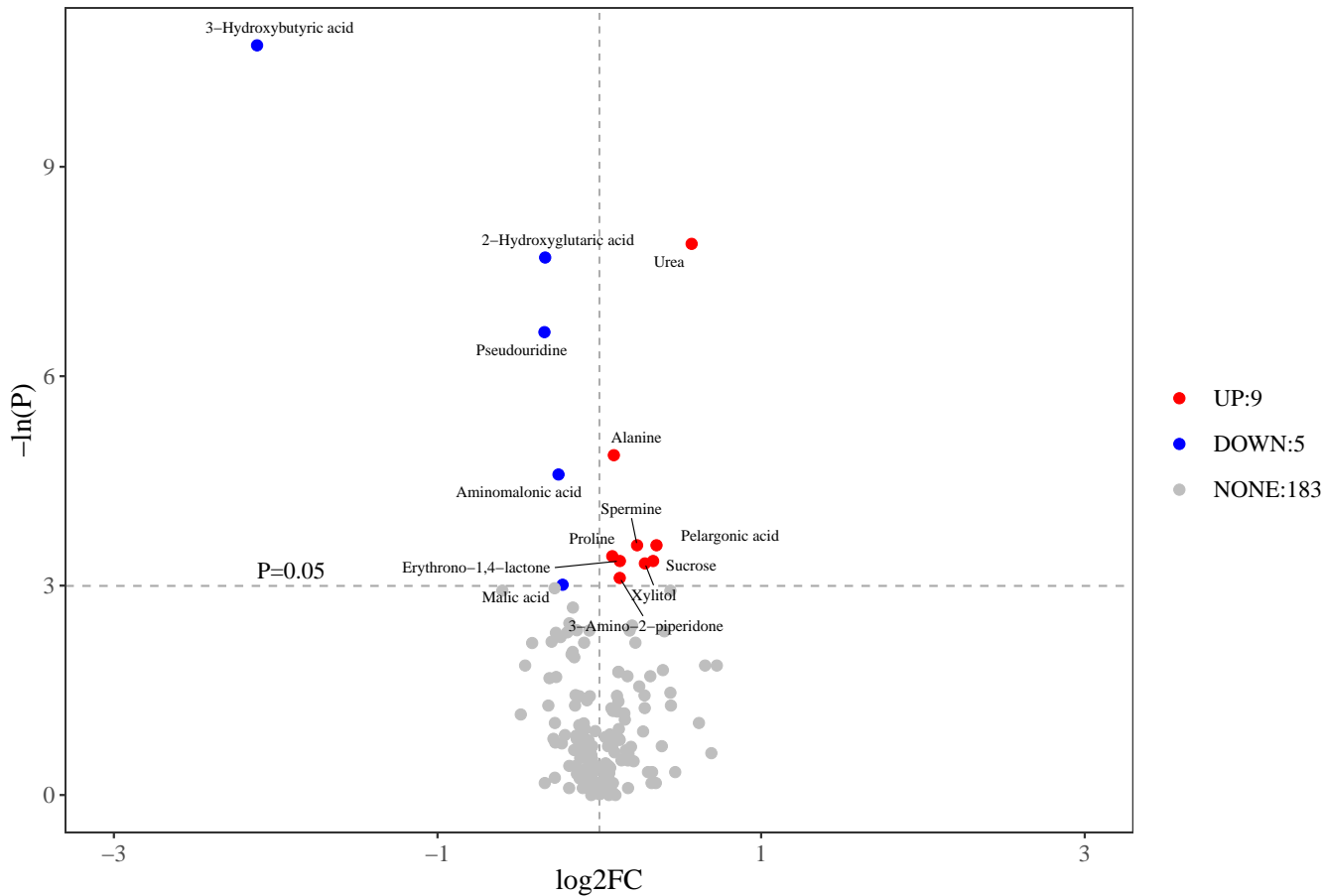

Supplement: Supplementary file 2 [file DataSheet1.zip › Supplementary File 2/Treatment/D_vs_H/05_Univariate_Analysis/Volcano_Plot_with_Label.pdf]

D H

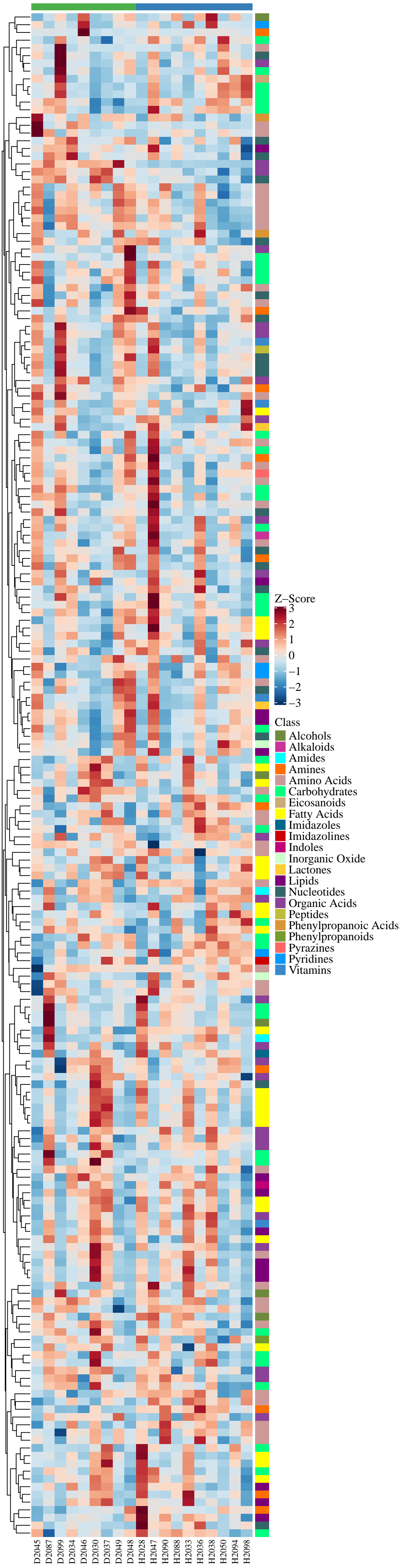

Supplement: Supplementary file 2 [file DataSheet1.zip › Supplementary File 2/Treatment/D_vs_H/01_Basic_Statistics/Z_Score_Heatmap.pdf]

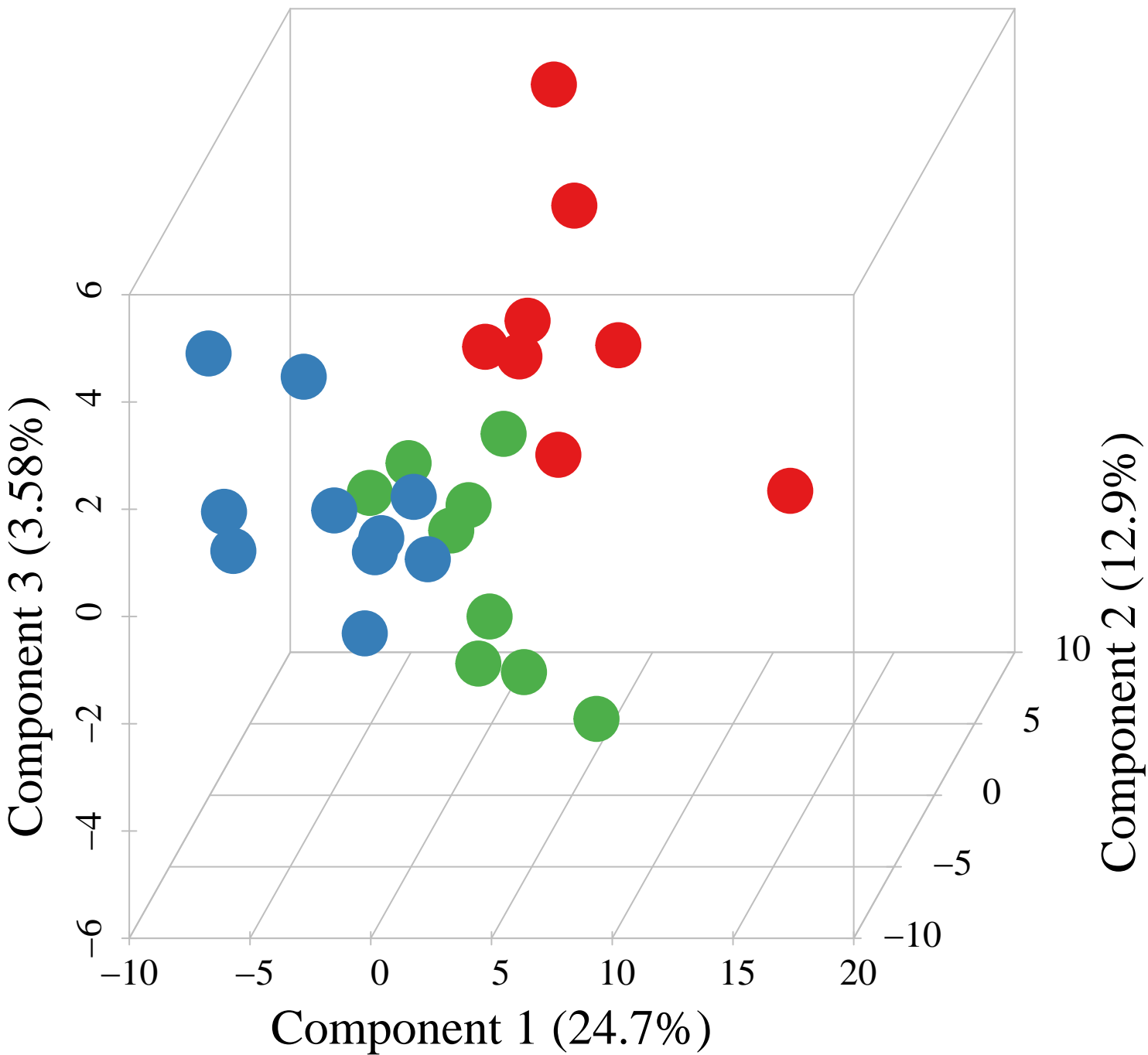

Supplement: Supplementary file 2 [file DataSheet1.zip › Supplementary File 2/Treatment/C_vs_D_vs_H/03_PLS_DA/PC123_Score_3D.pdf]

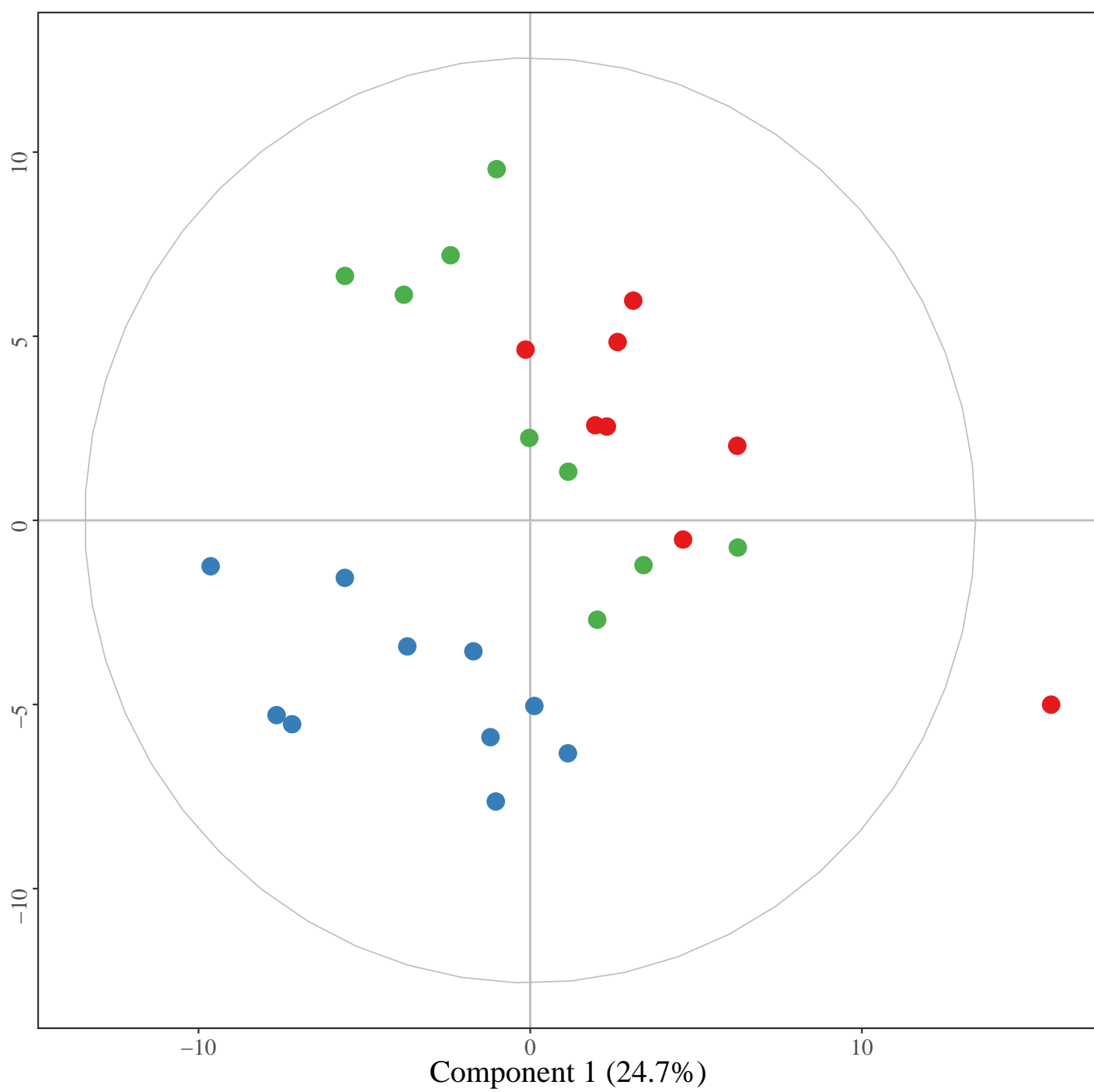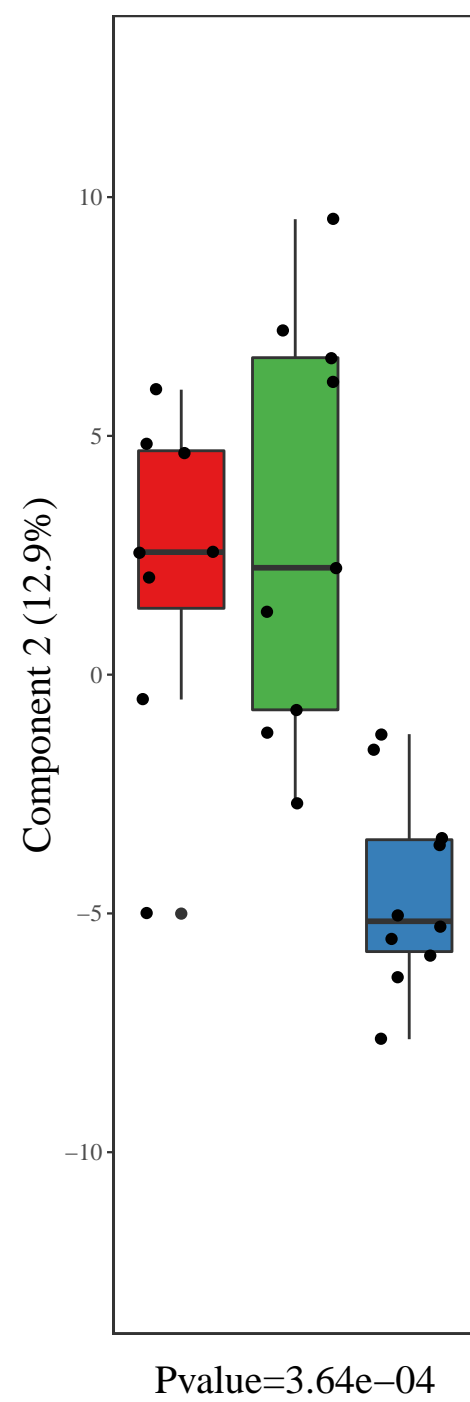

Pvalue=0.002

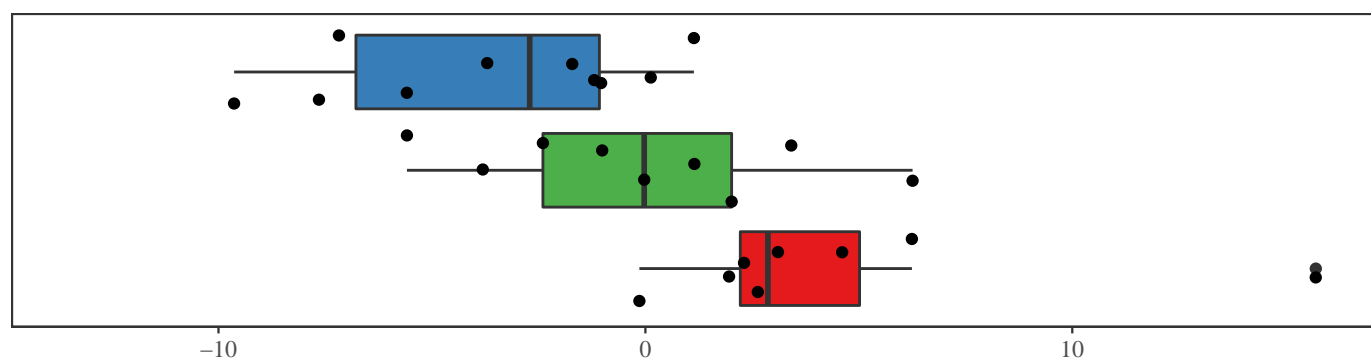

C  
D  
H

Supplement: Supplementary file 2 [file DataSheet1.zip › Supplementary File 2/Treatment/C_vs_D_vs_H/03_PLS_DA/PLSDA_Score_with_Boxplot_with_Points.pdf]

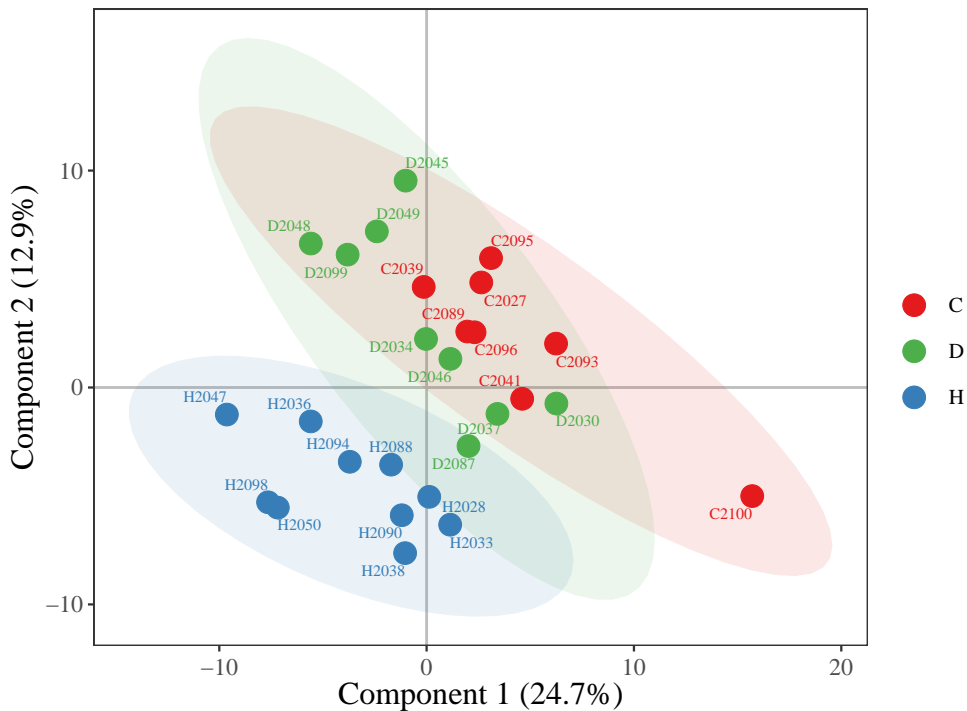

Supplement: Supplementary file 2 [file DataSheet1.zip › Supplementary File 2/Treatment/C_vs_D_vs_H/03_PLS_DA/PLSDA_Score_2D_Label.pdf]

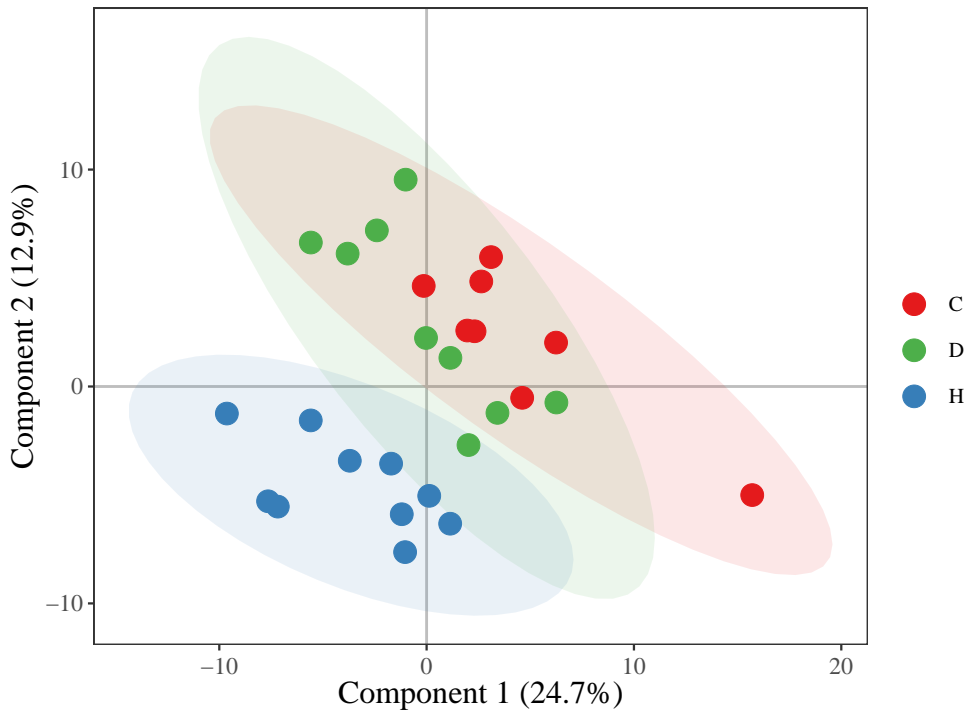

Supplement: Supplementary file 2 [file DataSheet1.zip › Supplementary File 2/Treatment/C_vs_D_vs_H/03_PLS_DA/PLSDA_Score_2D.pdf]

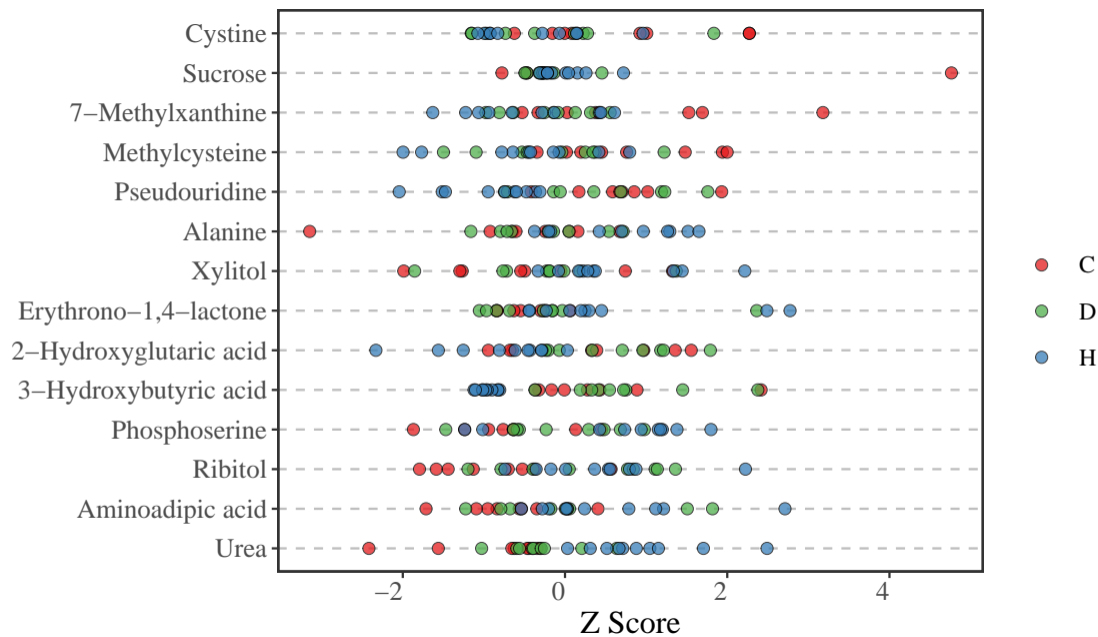

Supplement: Supplementary file 2 [file DataSheet1.zip › Supplementary File 2/Treatment/C_vs_D_vs_H/04_Univariate_Analysis/Z_Score_Plot.pdf]

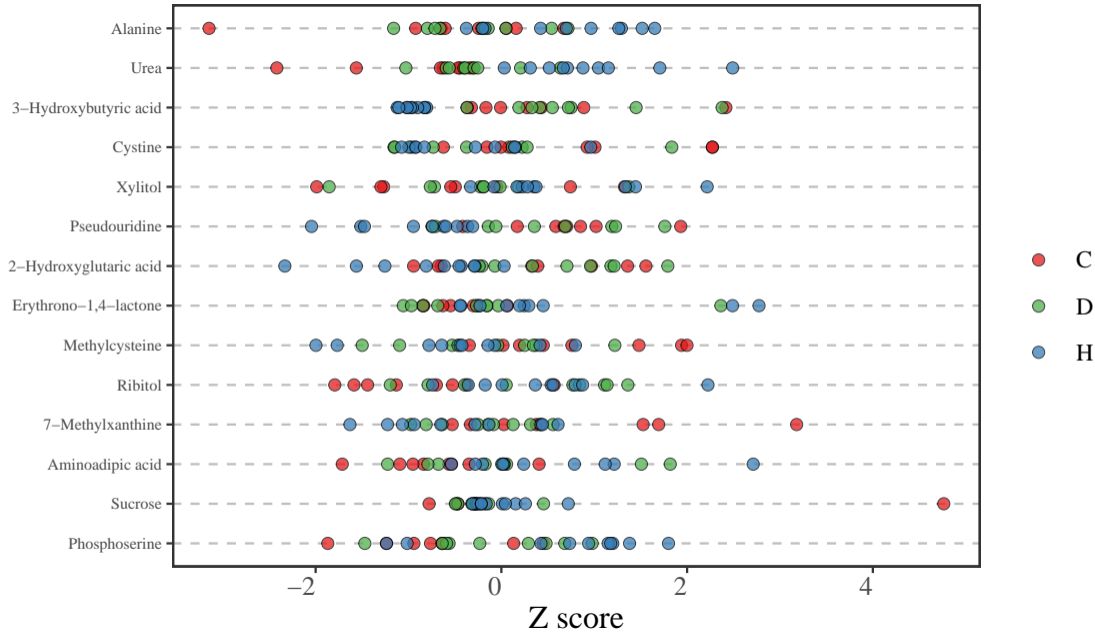

Supplement: Supplementary file 2 [file DataSheet1.zip › Supplementary File 2/Treatment/C_vs_D_vs_H/05_Potential_Biomarkers/Markers_Z_Score_Plot.pdf]

C D H

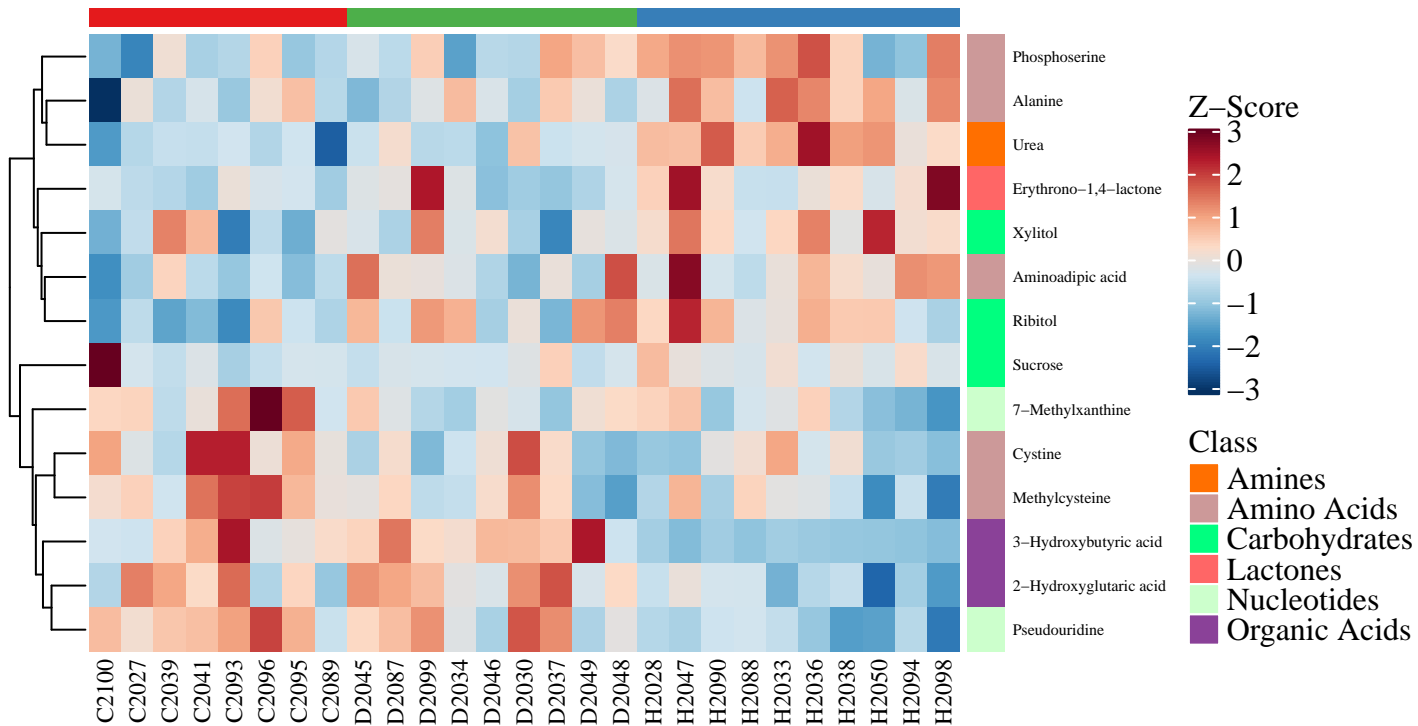

Supplement: Supplementary file 2 [file DataSheet1.zip › Supplementary File 2/Treatment/C_vs_D_vs_H/05_Potential_Biomarkers/Markers_Z_Score_Heatmap_with_Name.pdf]

C D H

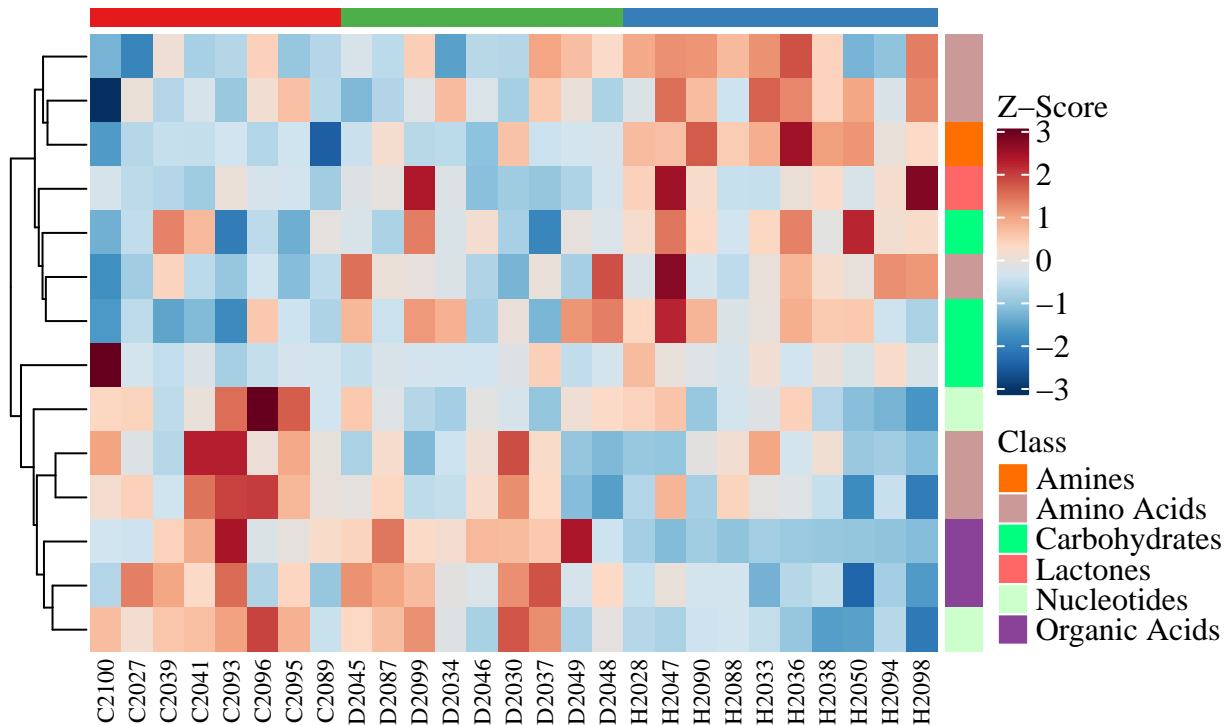

Supplement: Supplementary file 2 [file DataSheet1.zip › Supplementary File 2/Treatment/C_vs_D_vs_H/05_Potential_Biomarkers/Markers_Z_Score_Heatmap.pdf]

C D H

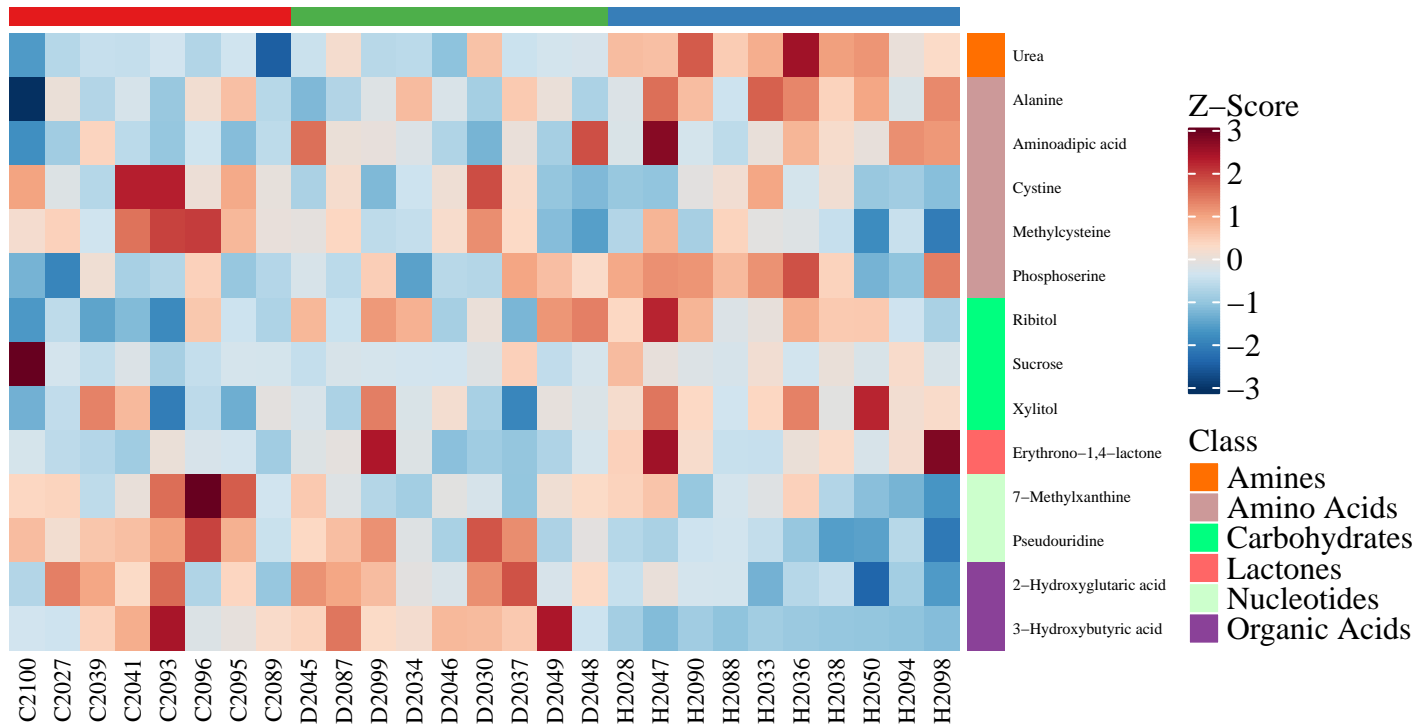

Supplement: Supplementary file 2 [file DataSheet1.zip › Supplementary File 2/Treatment/C_vs_D_vs_H/05_Potential_Biomarkers/Markers_Z_Score_Heatmap_ordered_by_Class_with_Name.pdf]

C D H

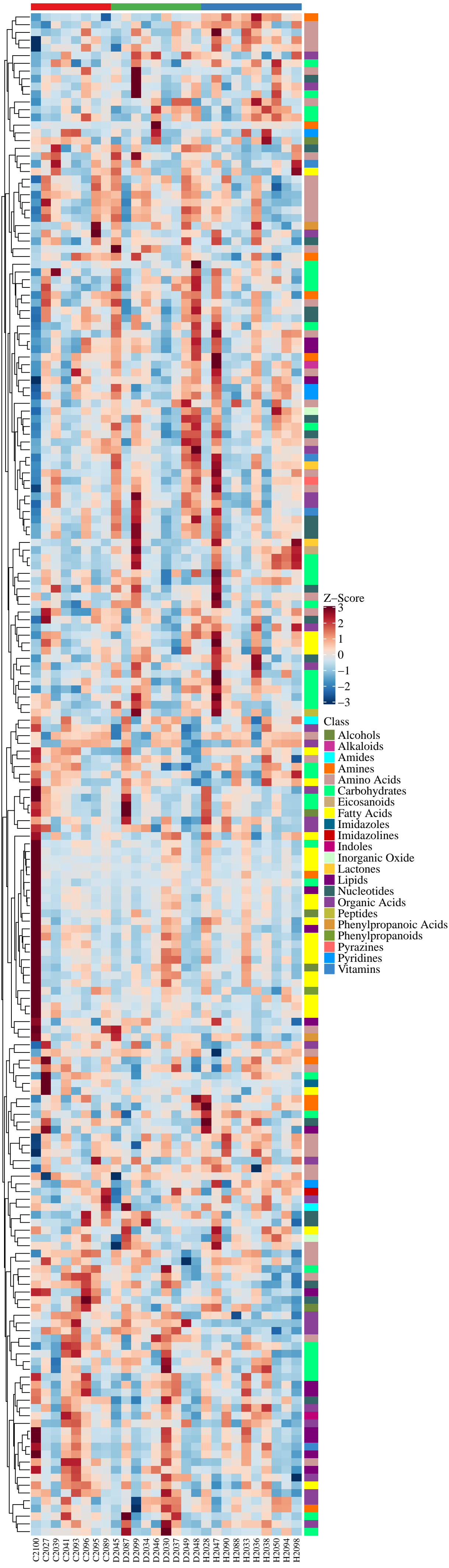

Supplement: Supplementary file 2 [file DataSheet1.zip › Supplementary File 2/Treatment/C_vs_D_vs_H/01_Basic_Statistics/Z_Score_Heatmap.pdf]

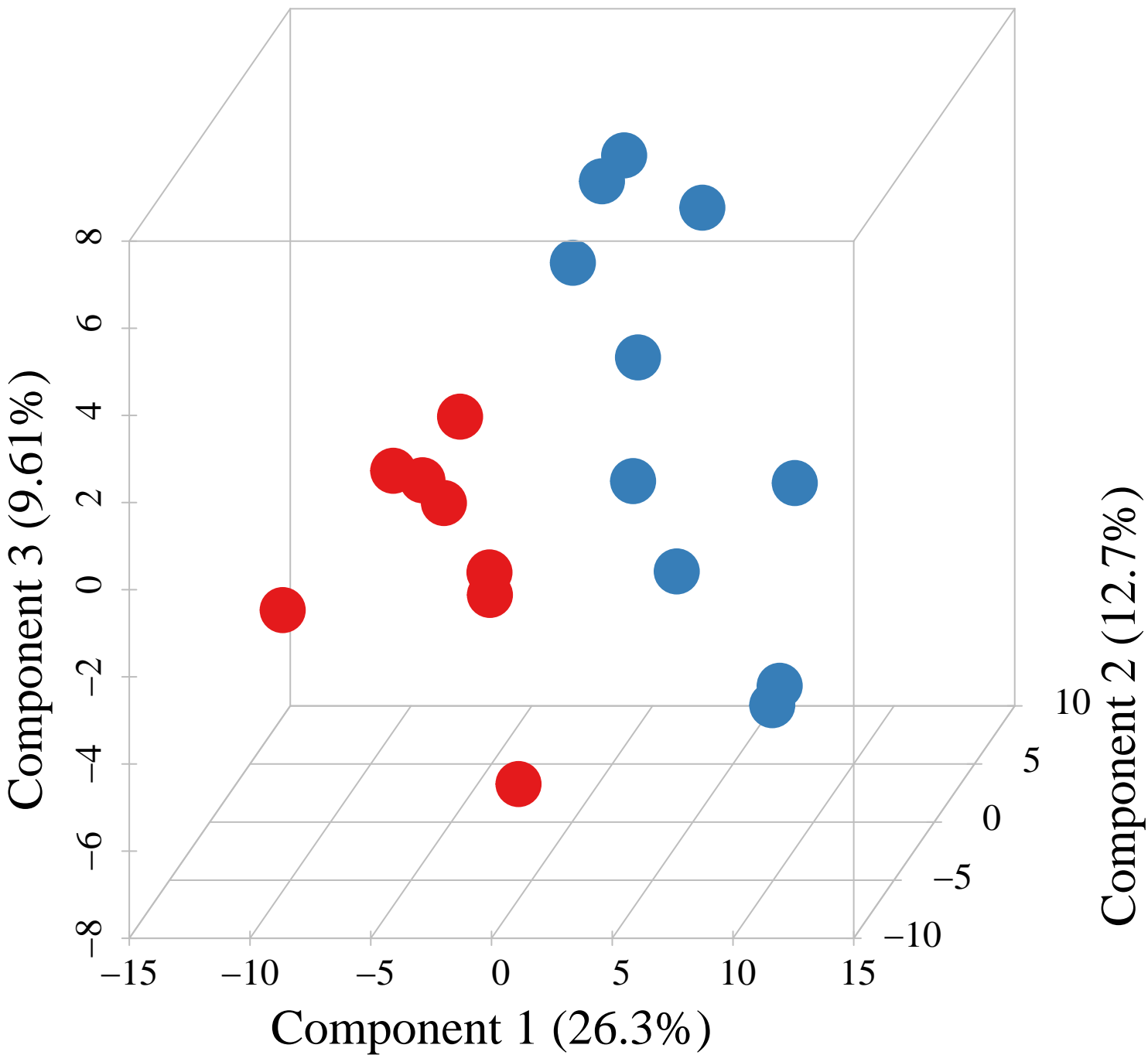

Supplement: Supplementary file 2 [file DataSheet1.zip › Supplementary File 2/Treatment/C_vs_H/03_PLS_DA/PC123_Score_3D.pdf]

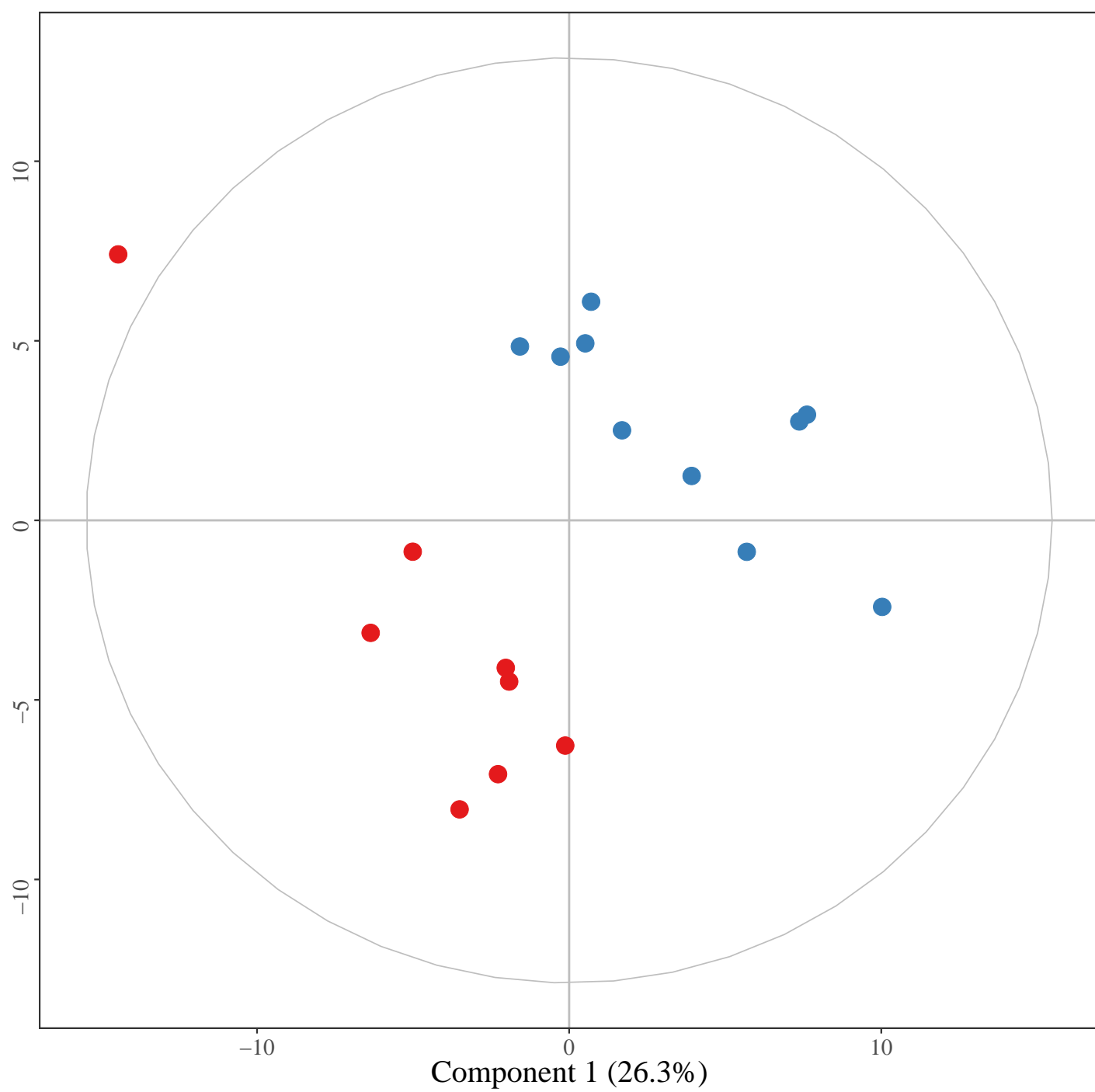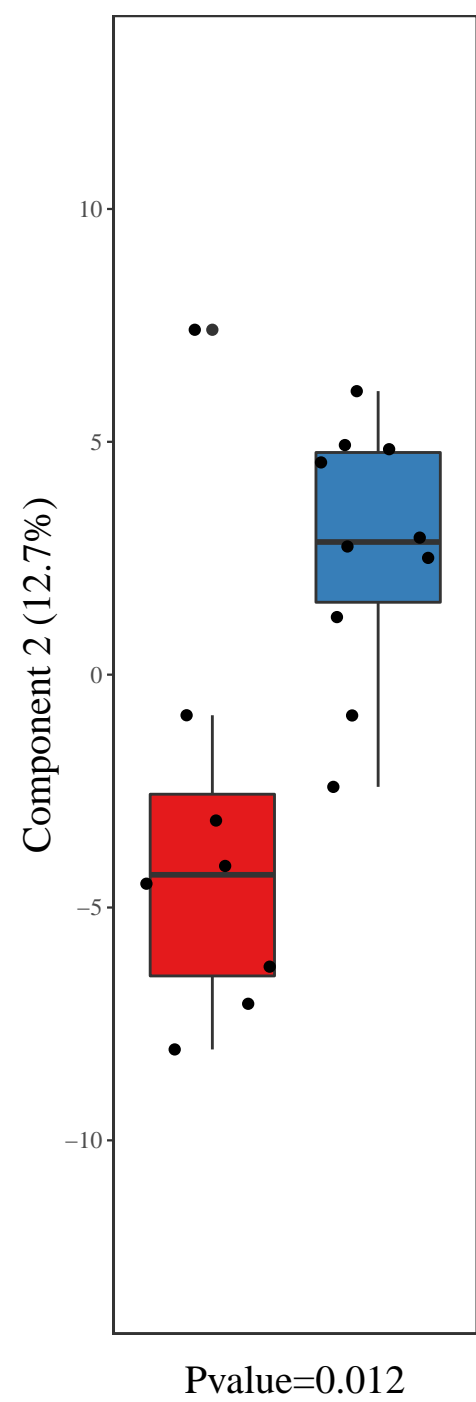

Pvalue=1.83e-04

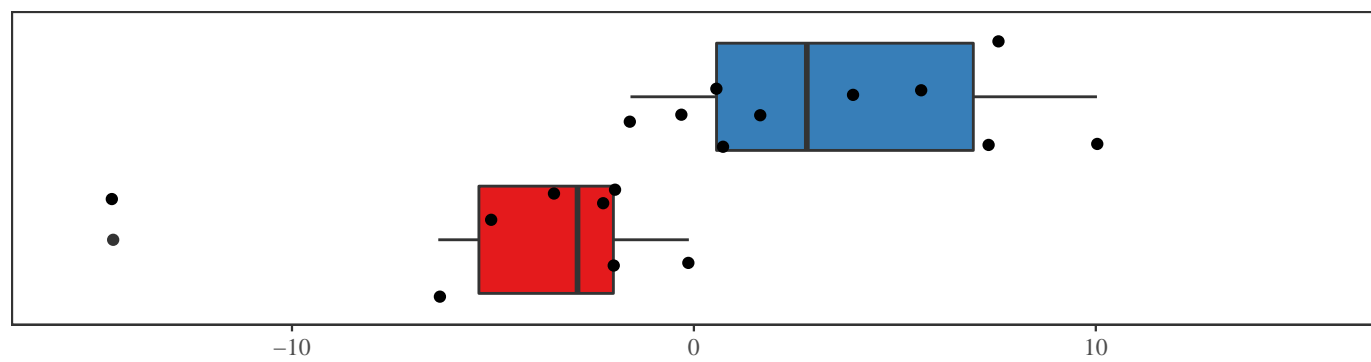

C  
H

Supplement: Supplementary file 2 [file DataSheet1.zip › Supplementary File 2/Treatment/C_vs_H/03_PLS_DA/PLSDA_Score_with_Boxplot_with_Points.pdf]

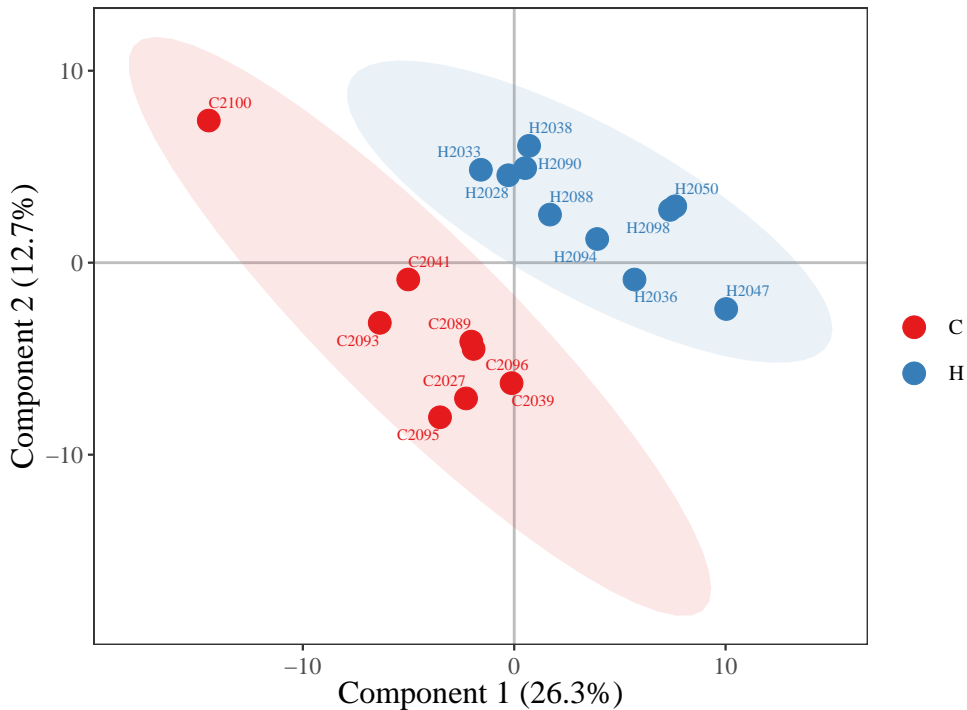

Supplement: Supplementary file 2 [file DataSheet1.zip › Supplementary File 2/Treatment/C_vs_H/03_PLS_DA/PLSDA_Score_2D_Label.pdf]

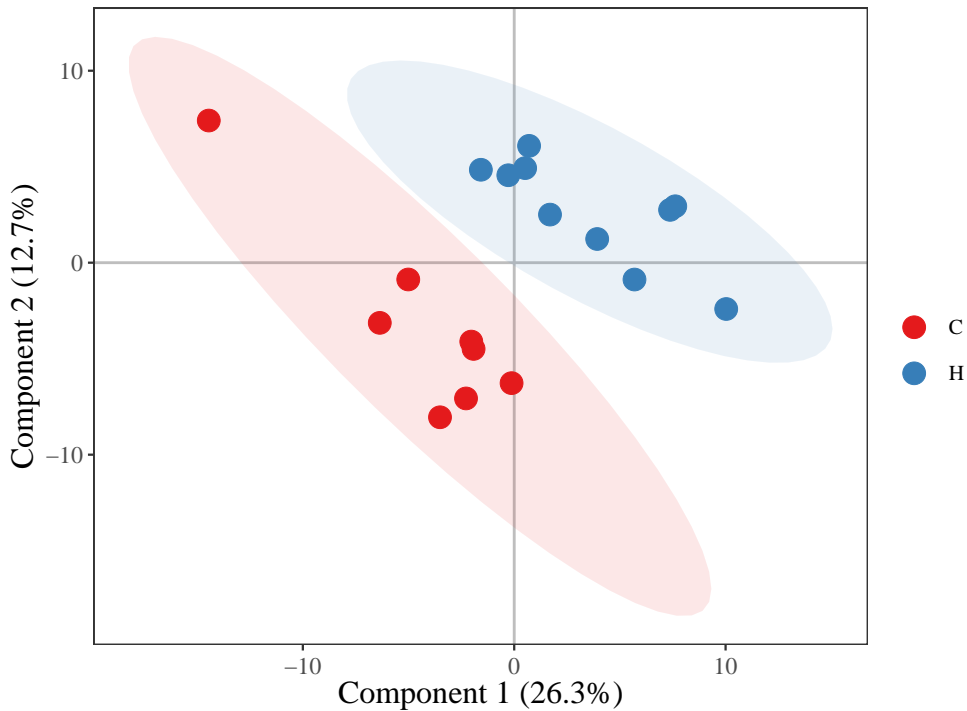

Supplement: Supplementary file 2 [file DataSheet1.zip › Supplementary File 2/Treatment/C_vs_H/03_PLS_DA/PLSDA_Score_2D.pdf]

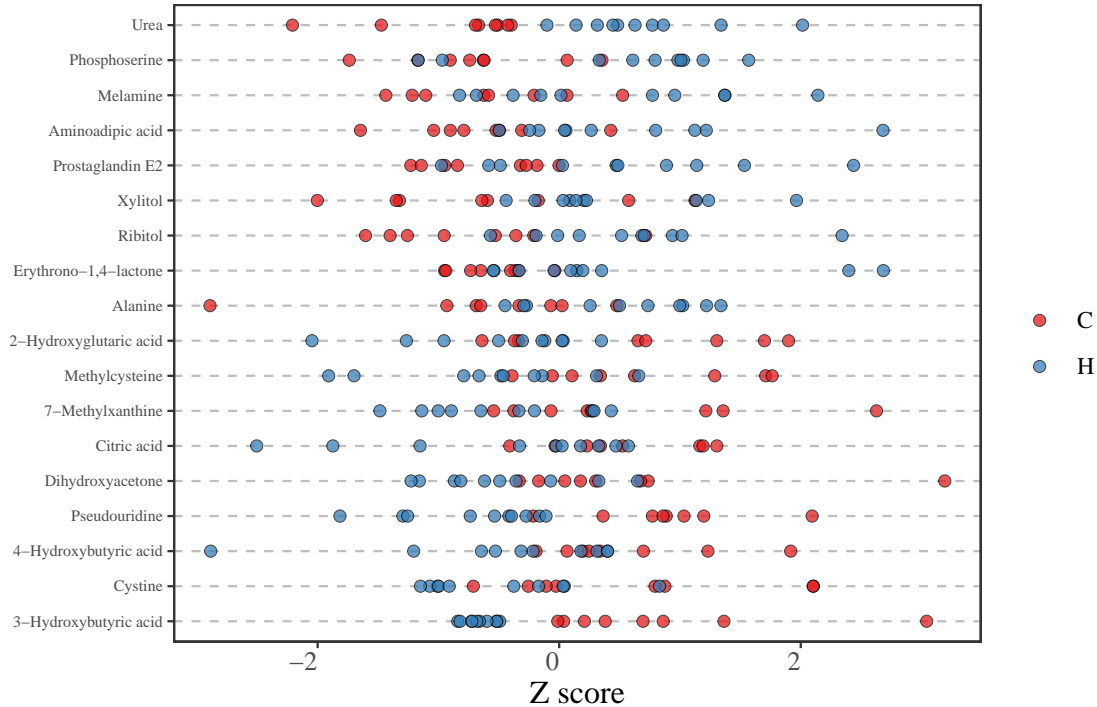

Supplement: Supplementary file 2 [file DataSheet1.zip › Supplementary File 2/Treatment/C_vs_H/06_Potential_Biomarkers/Markers_Z_Score_Plot.pdf]

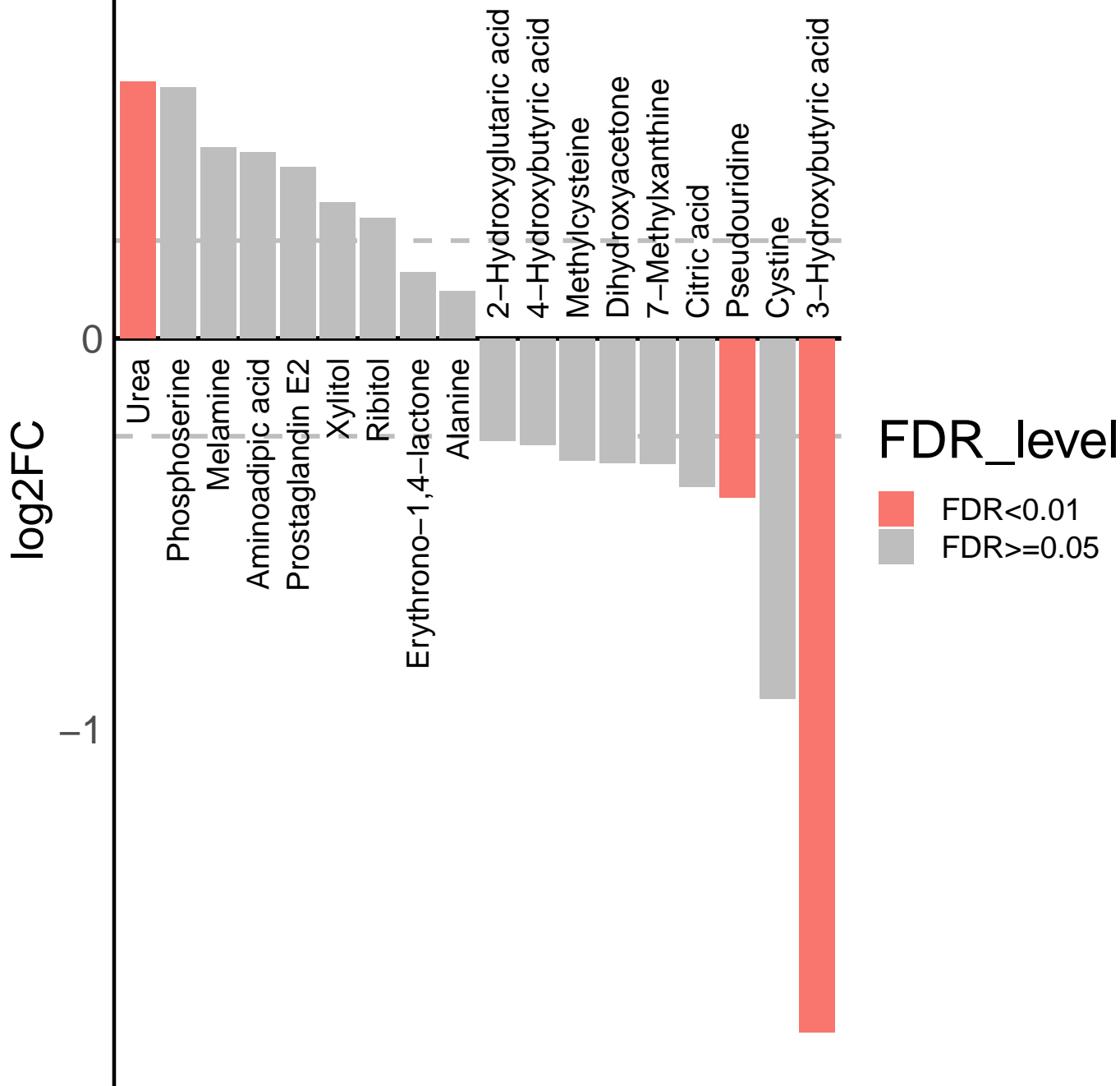

Supplement: Supplementary file 2 [file DataSheet1.zip › Supplementary File 2/Treatment/C_vs_H/06_Potential_Biomarkers/Markers_log2FC_FDR_Bar_Plot.pdf]

Univariate

OPLS-DA

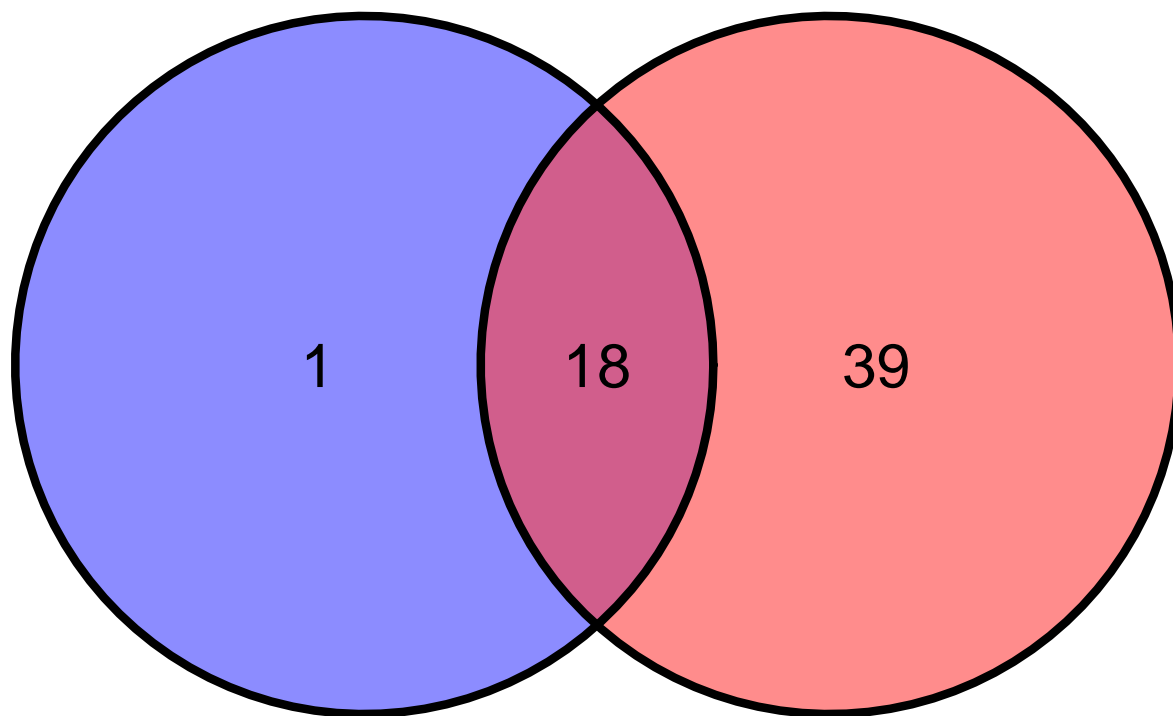

Supplement: Supplementary file 2 [file DataSheet1.zip › Supplementary File 2/Treatment/C_vs_H/06_Potential_Biomarkers/Venn_Plot.pdf]

C H

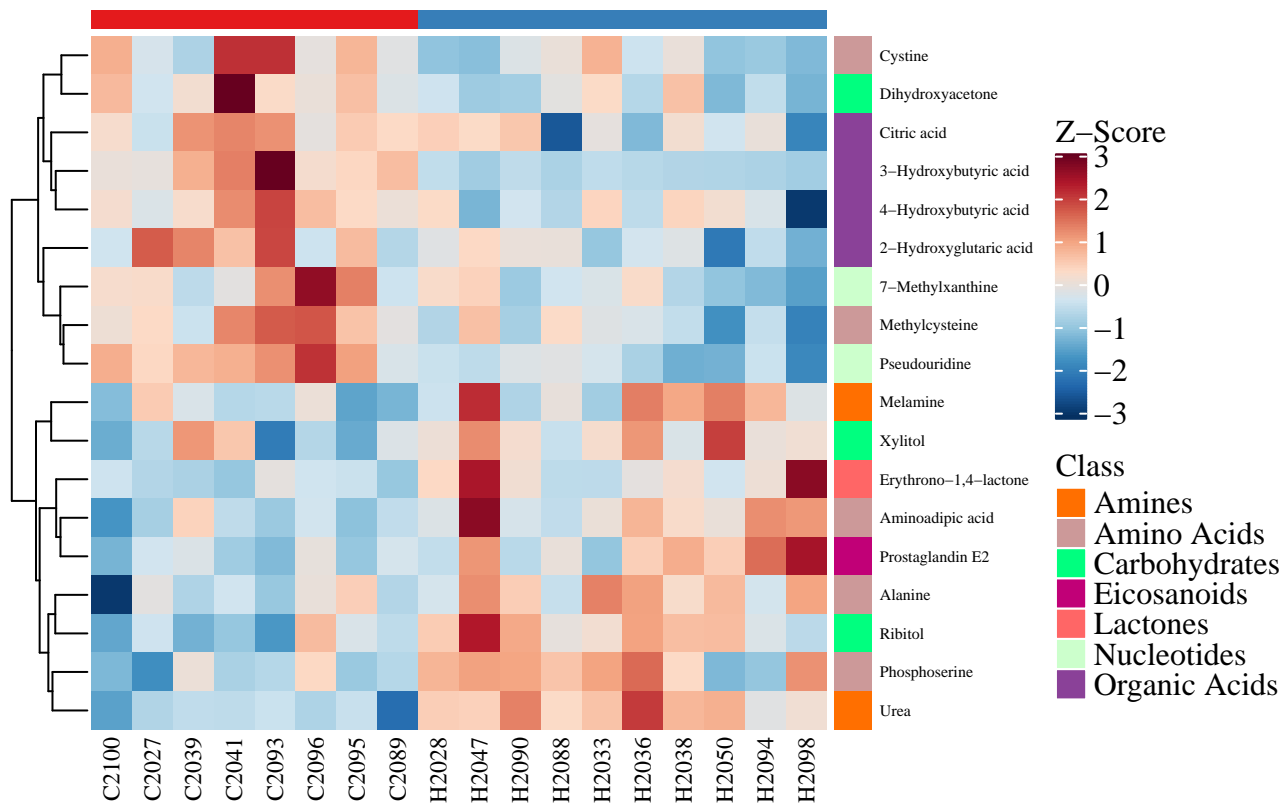

Supplement: Supplementary file 2 [file DataSheet1.zip › Supplementary File 2/Treatment/C_vs_H/06_Potential_Biomarkers/Markers_Z_Score_Heatmap_with_Name.pdf]

C H

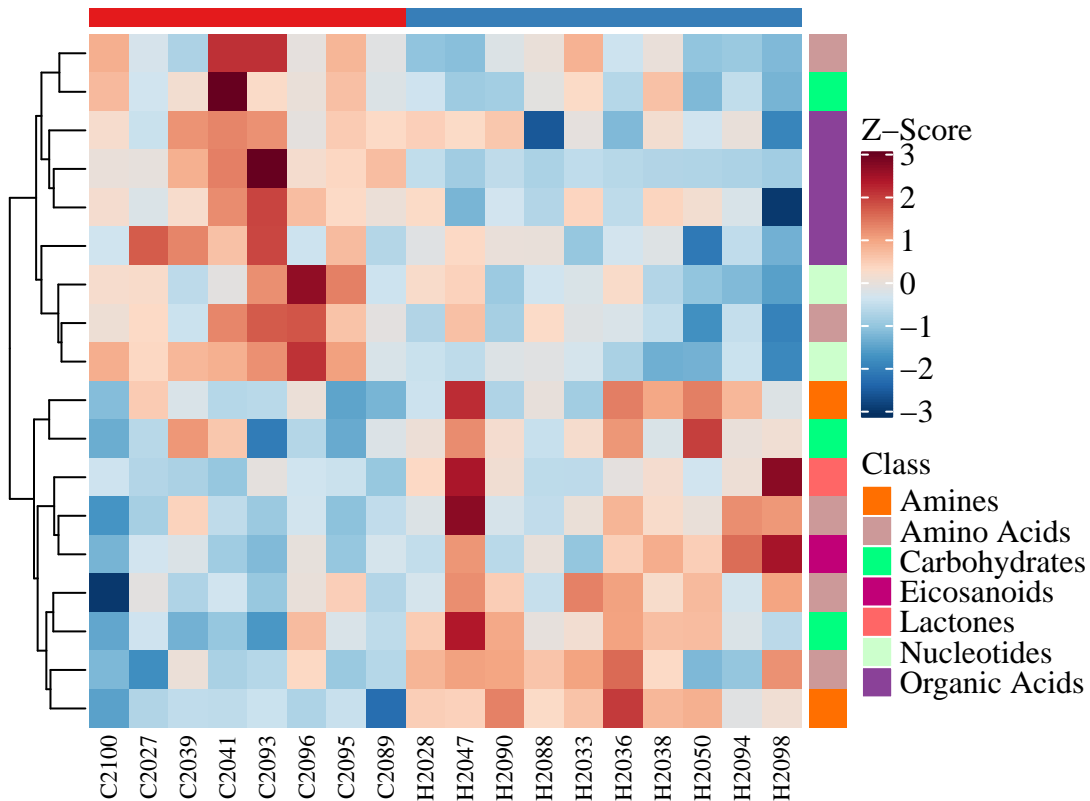

Supplement: Supplementary file 2 [file DataSheet1.zip › Supplementary File 2/Treatment/C_vs_H/06_Potential_Biomarkers/Markers_Z_Score_Heatmap.pdf]

■ C ■ H

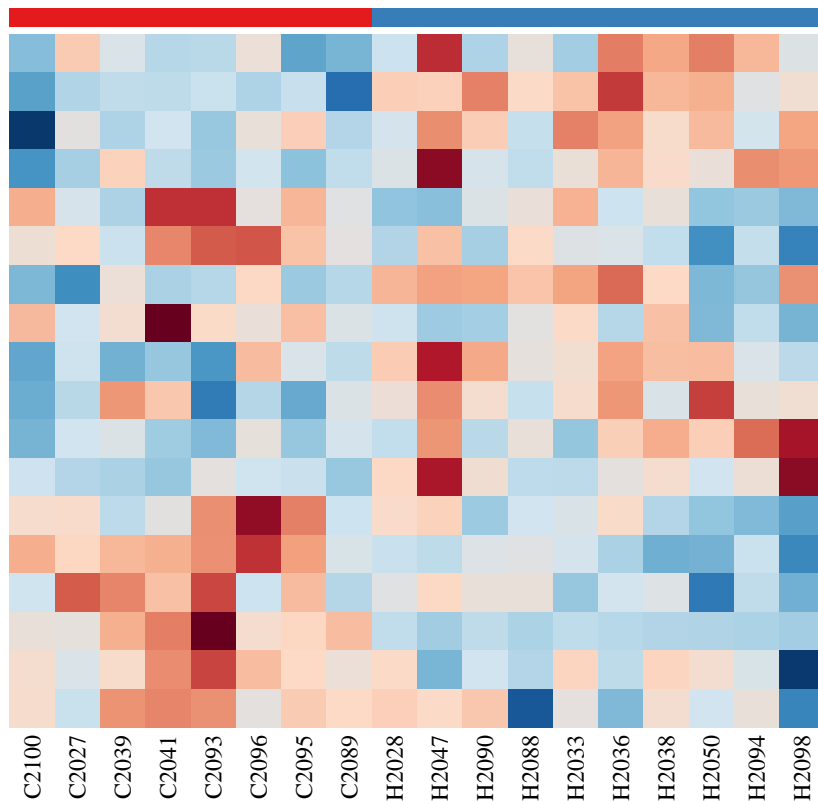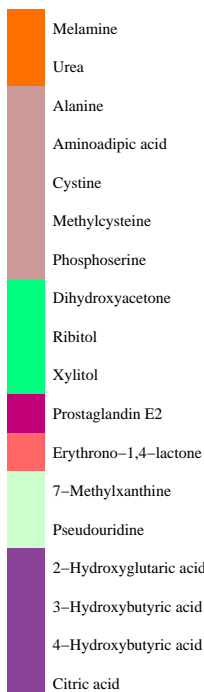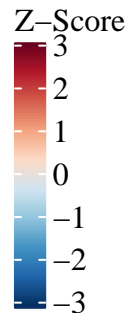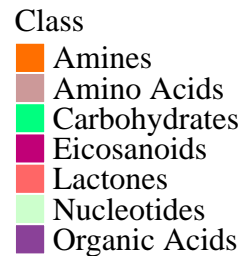

Supplement: Supplementary file 2 [file DataSheet1.zip › Supplementary File 2/Treatment/C_vs_H/06_Potential_Biomarkers/Markers_Z_Score_Heatmap_ordered_by_Class_with_Name.pdf]

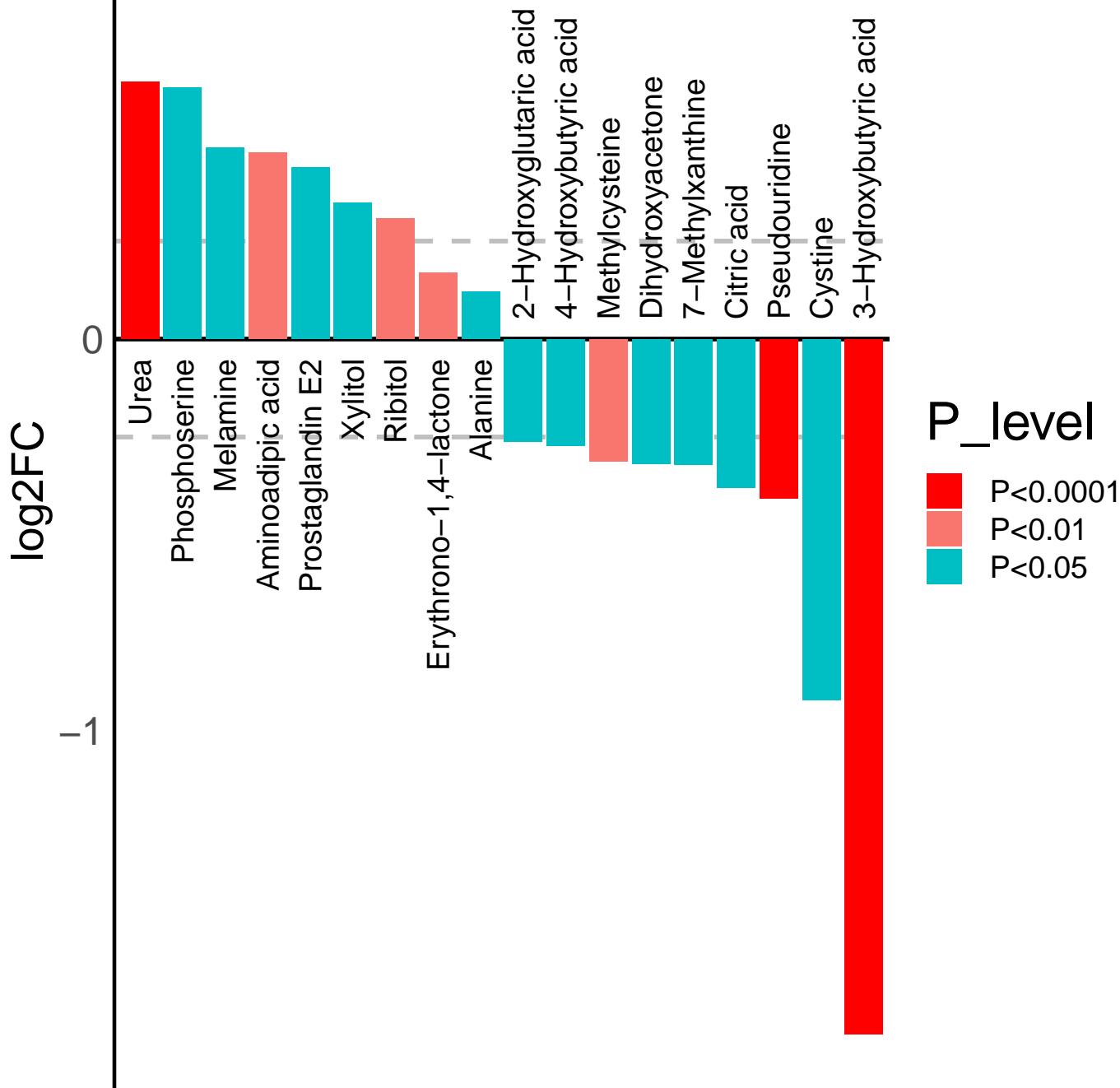

Supplement: Supplementary file 2 [file DataSheet1.zip › Supplementary File 2/Treatment/C_vs_H/06_Potential_Biomarkers/Markers_log2FC_P_Bar_Plot.pdf]

O1 (29.2%)

20

10

0

-10

-20

-5

0

5

P1 (9.84%)

C2095

C2027

C2096

C2039

C2093

C2089

C2041

C2100

H2036

H2047

H2094

H2098

H2050

H2088

H2028

H2090

H2033

H2038

C

H

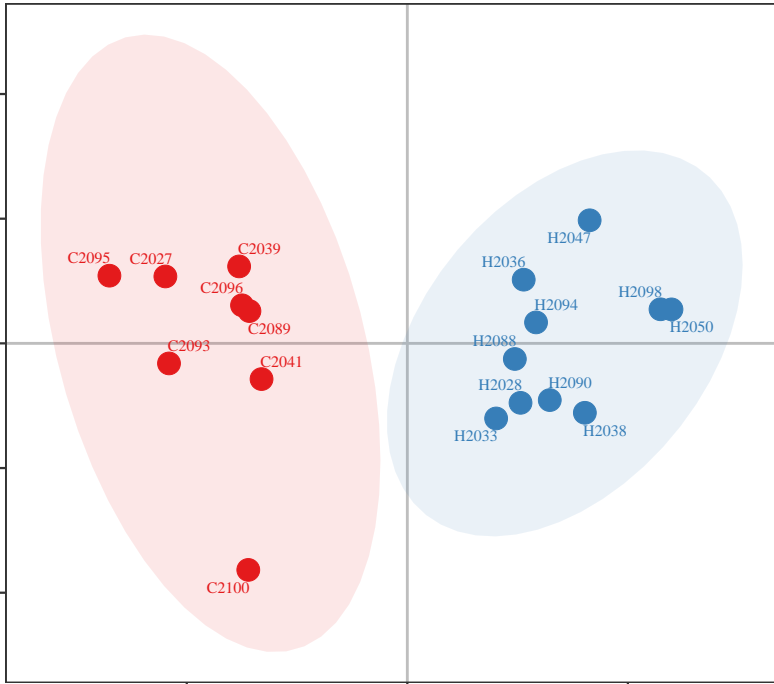

Supplement: Supplementary file 2 [file DataSheet1.zip › Supplementary File 2/Treatment/C_vs_H/04_OPLS_DA/OPLSDA_Score_2D_Label.pdf]

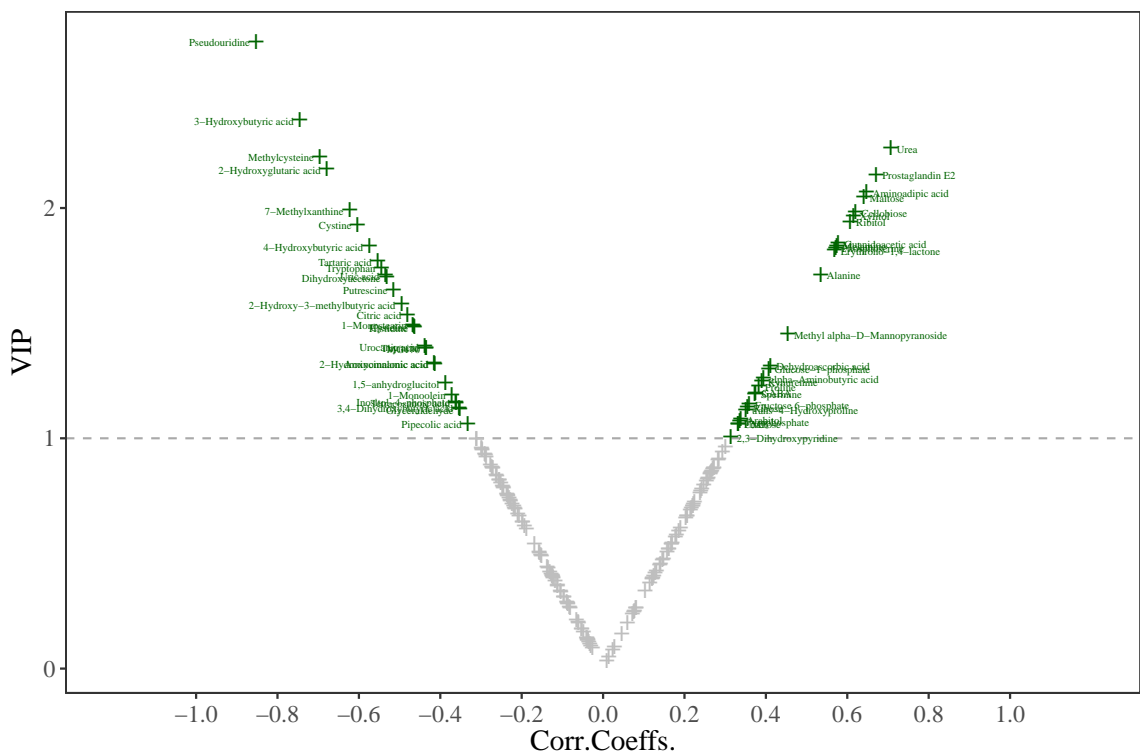

Supplement: Supplementary file 2 [file DataSheet1.zip › Supplementary File 2/Treatment/C_vs_H/04_OPLS_DA/OPLSDA_VPlot.pdf]

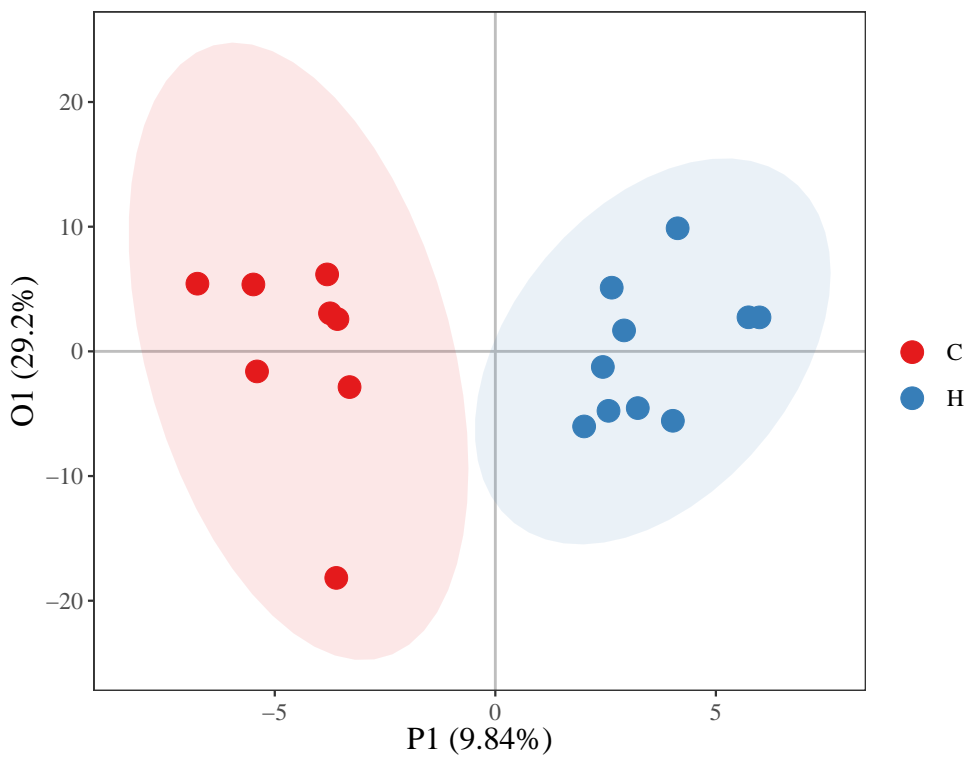

Supplement: Supplementary file 2 [file DataSheet1.zip › Supplementary File 2/Treatment/C_vs_H/04_OPLS_DA/OPLSDA_Score_2D.pdf]

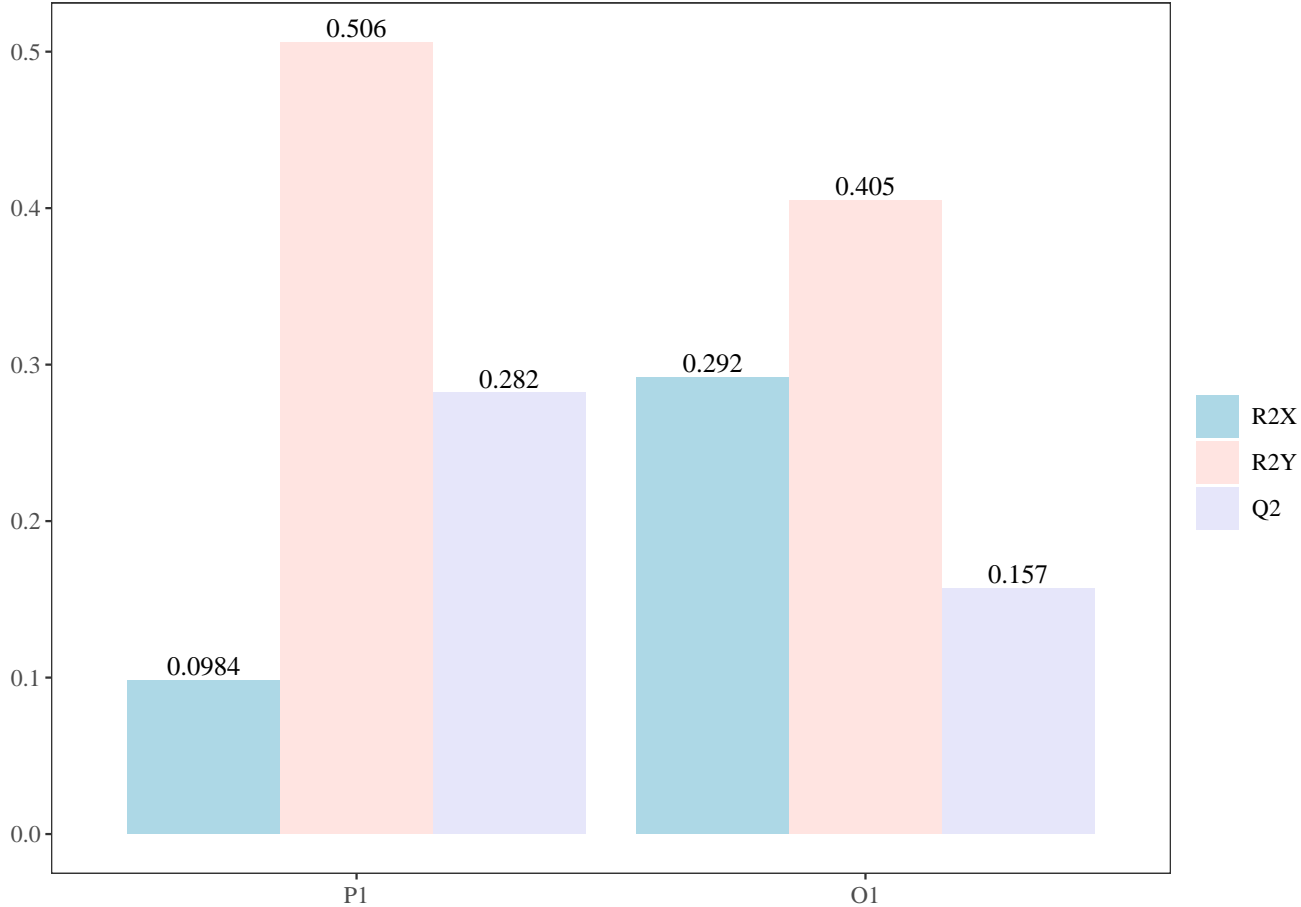

Supplement: Supplementary file 2 [file DataSheet1.zip › Supplementary File 2/Treatment/C_vs_H/04_OPLS_DA/OPLSDA_R2X_R2Y_Q2.pdf]

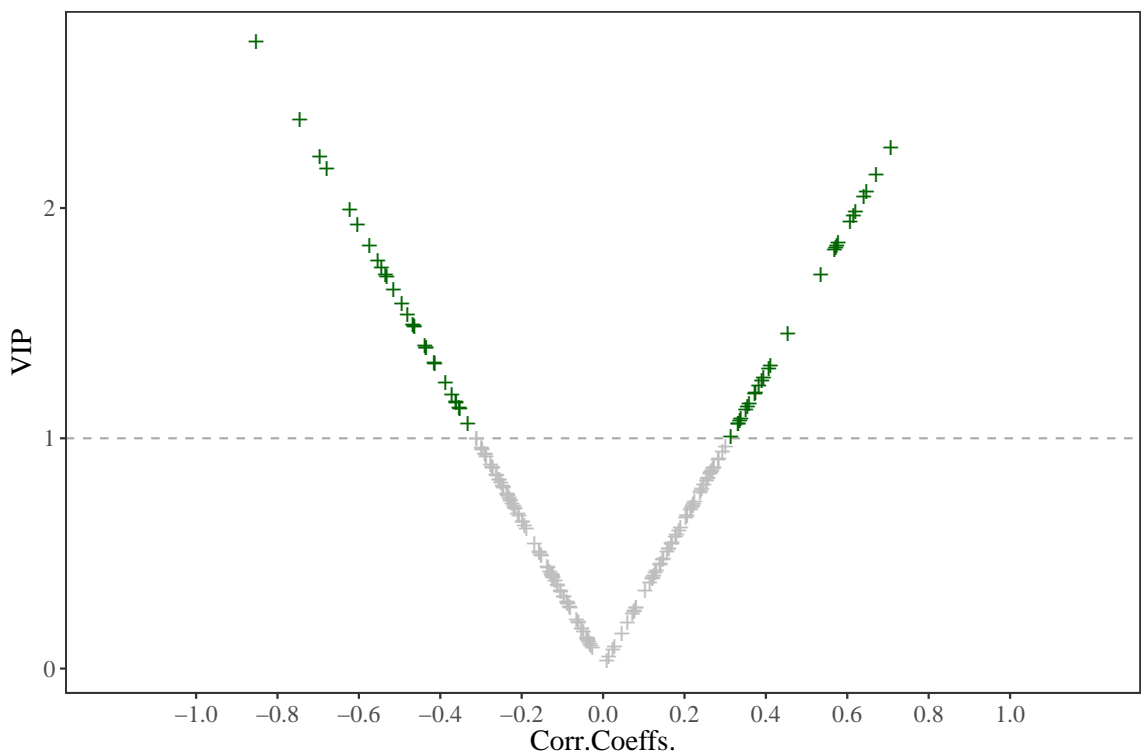

Supplement: Supplementary file 2 [file DataSheet1.zip › Supplementary File 2/Treatment/C_vs_H/04_OPLS_DA/OPLSDA_VPlot_without_Label.pdf]

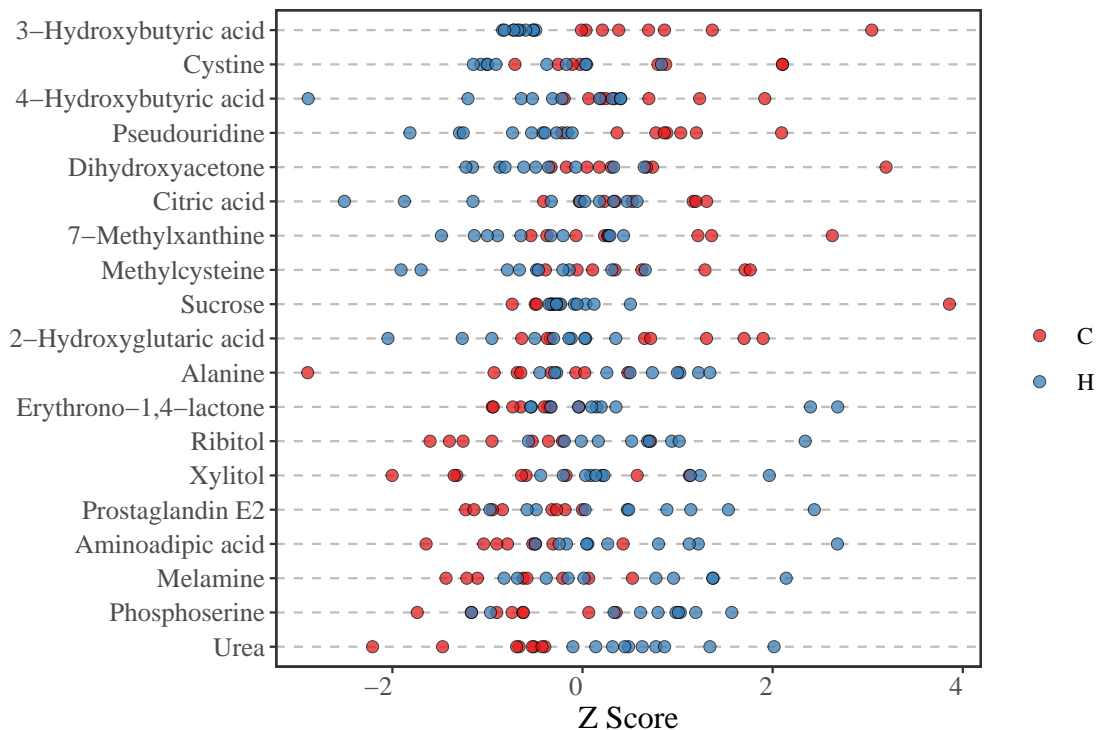

Supplement: Supplementary file 2 [file DataSheet1.zip › Supplementary File 2/Treatment/C_vs_H/05_Univariate_Analysis/Z_Score_Plot.pdf]

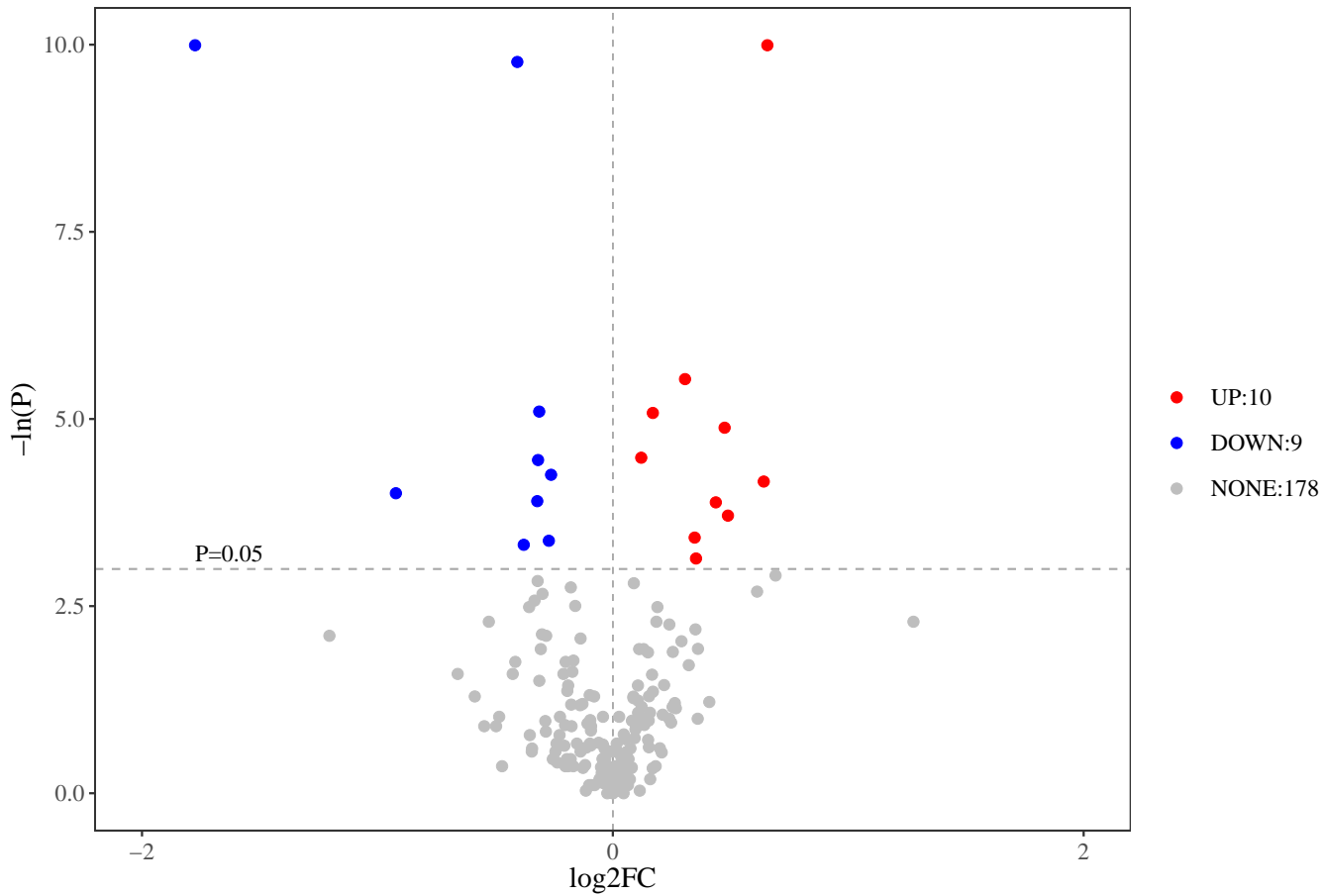

Supplement: Supplementary file 2 [file DataSheet1.zip › Supplementary File 2/Treatment/C_vs_H/05_Univariate_Analysis/Volcano_Plot.pdf]

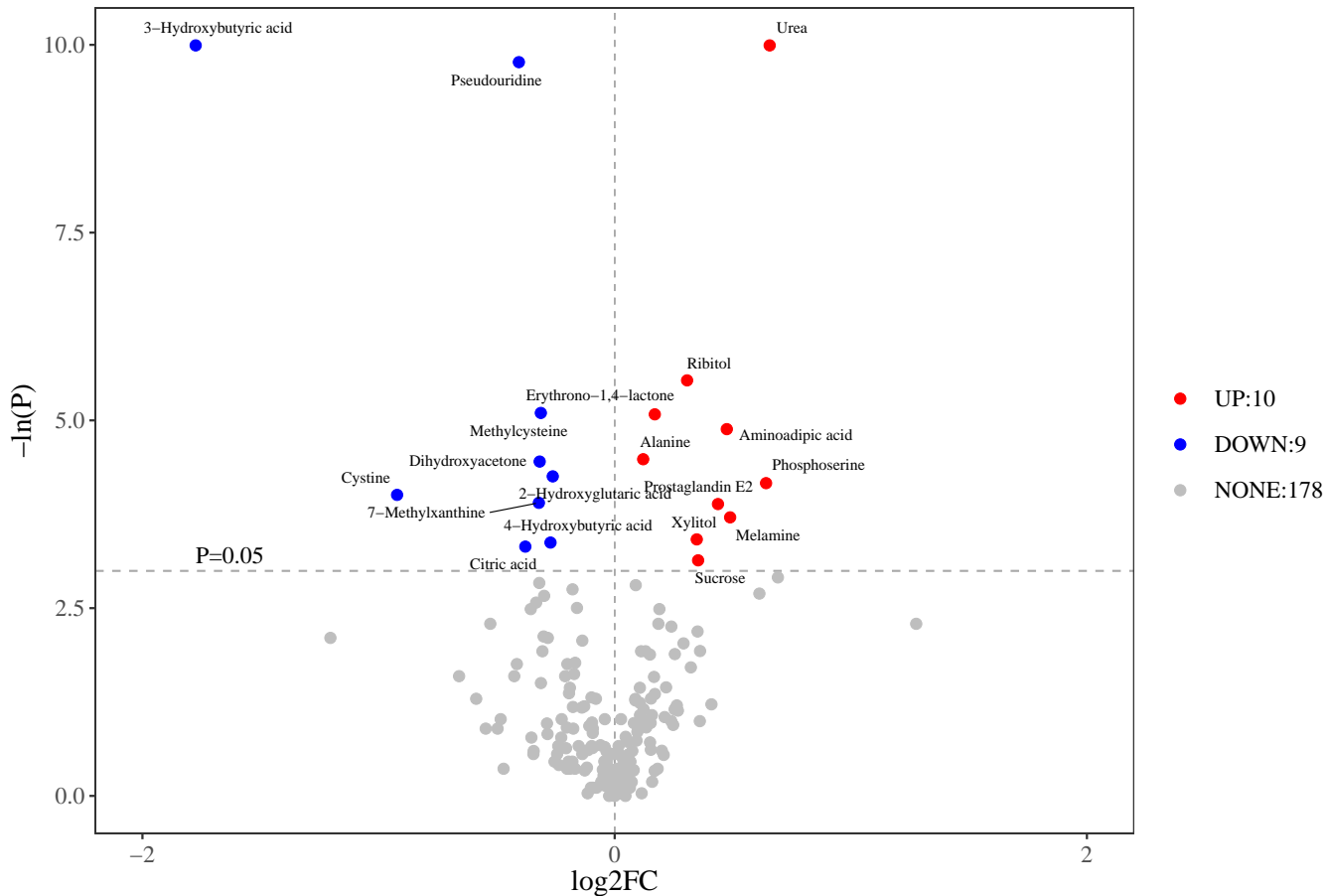

Supplement: Supplementary file 2 [file DataSheet1.zip › Supplementary File 2/Treatment/C_vs_H/05_Univariate_Analysis/Volcano_Plot_with_Label.pdf]

C H

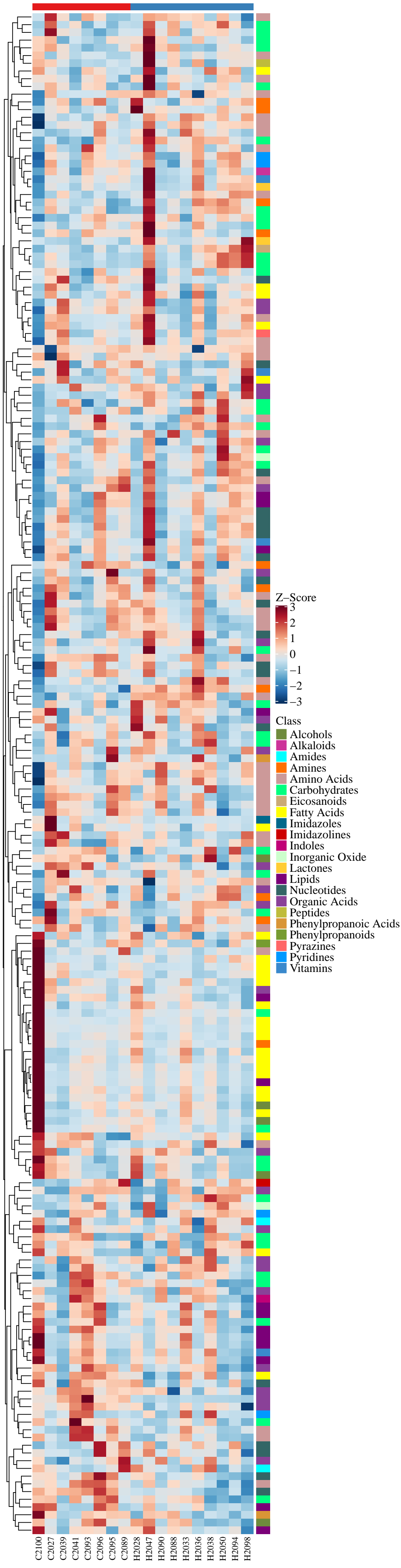

Supplement: Supplementary file 2 [file DataSheet1.zip › Supplementary File 2/Treatment/C_vs_H/01_Basic_Statistics/Z_Score_Heatmap.pdf]

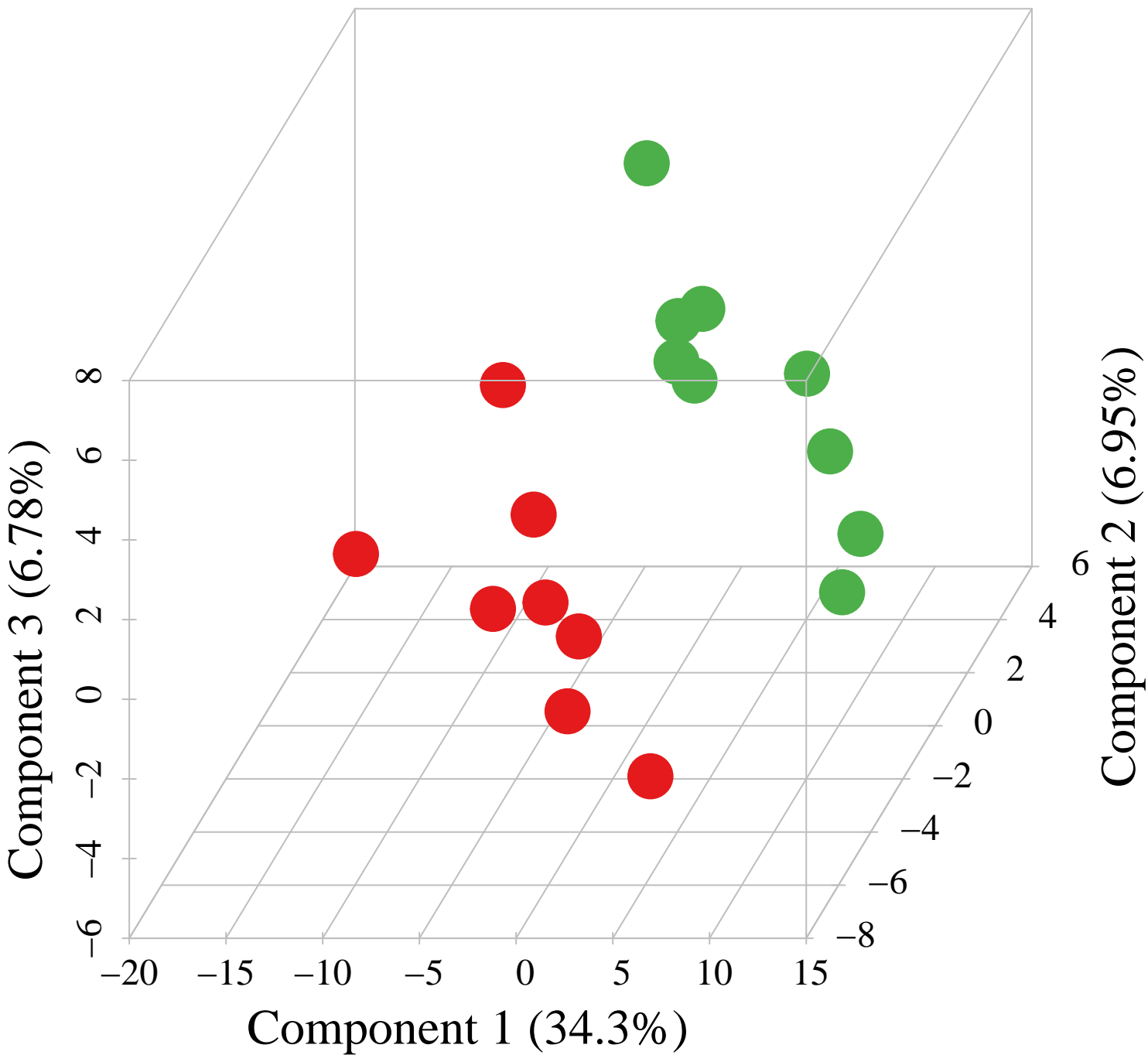

Supplement: Supplementary file 2 [file DataSheet1.zip › Supplementary File 2/Treatment/C_vs_D/03_PLS_DA/PC123_Score_3D.pdf]

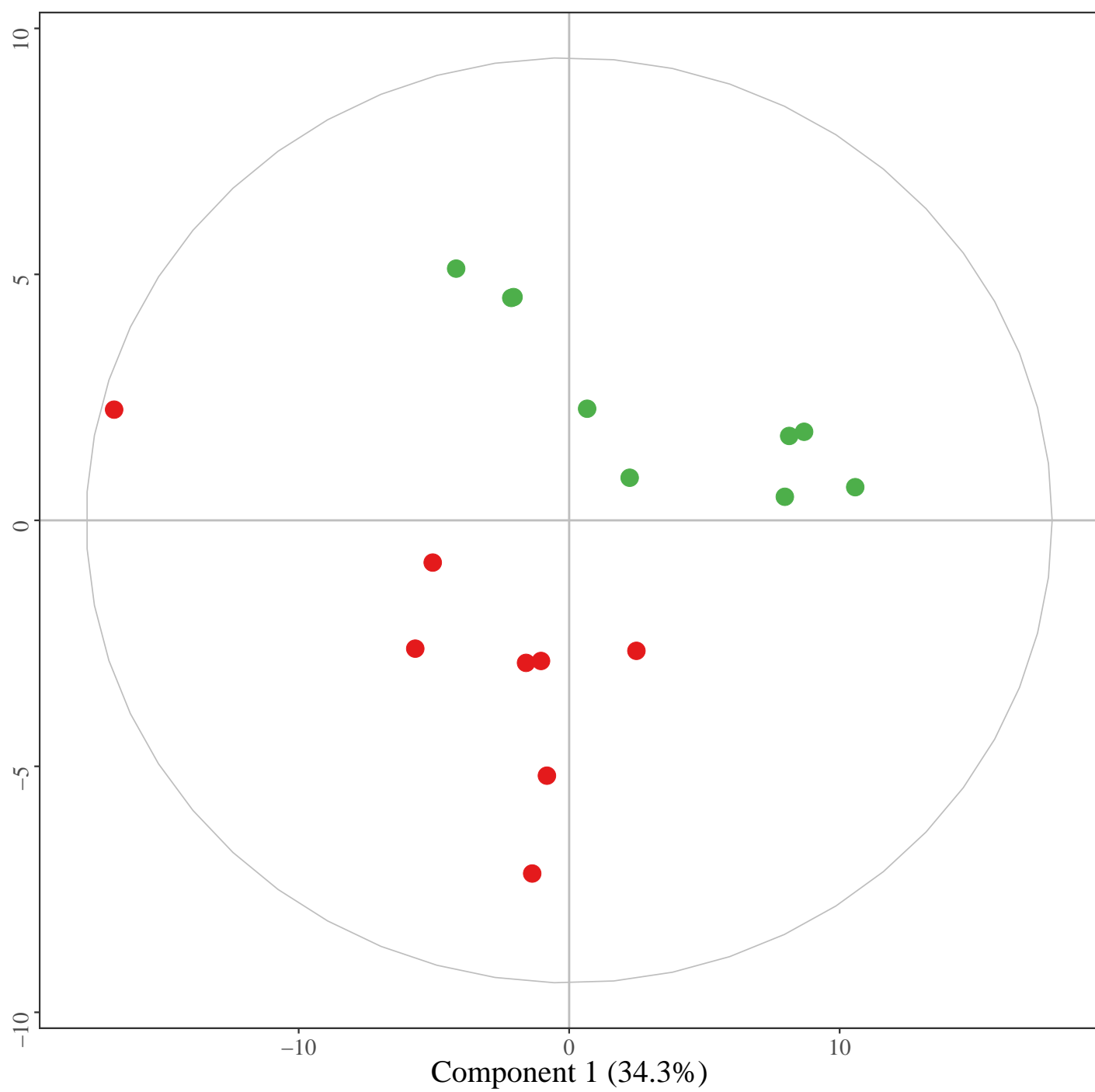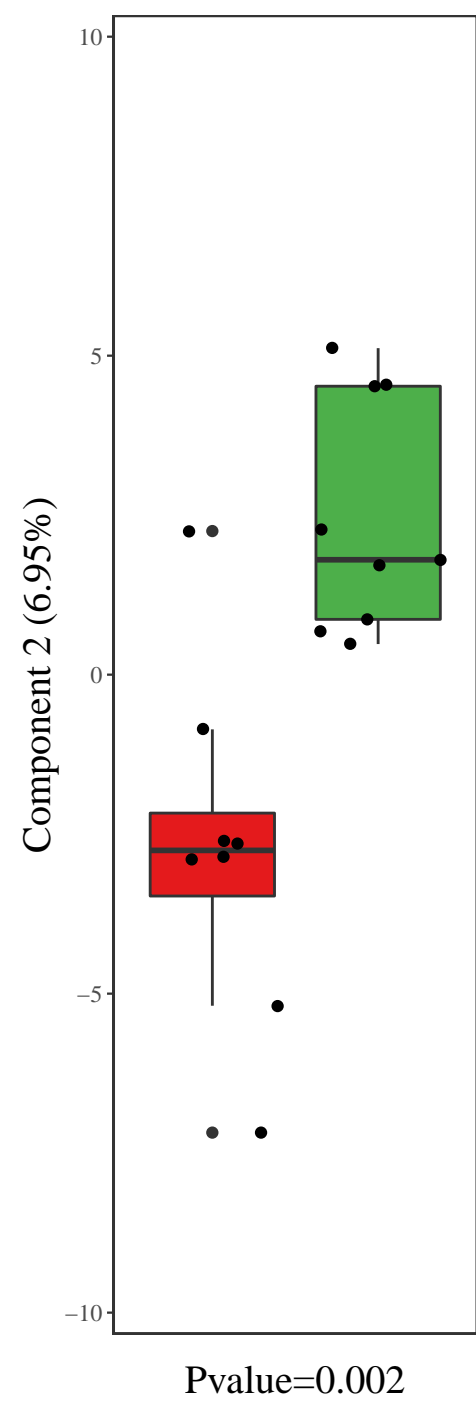

Pvalue=0.074

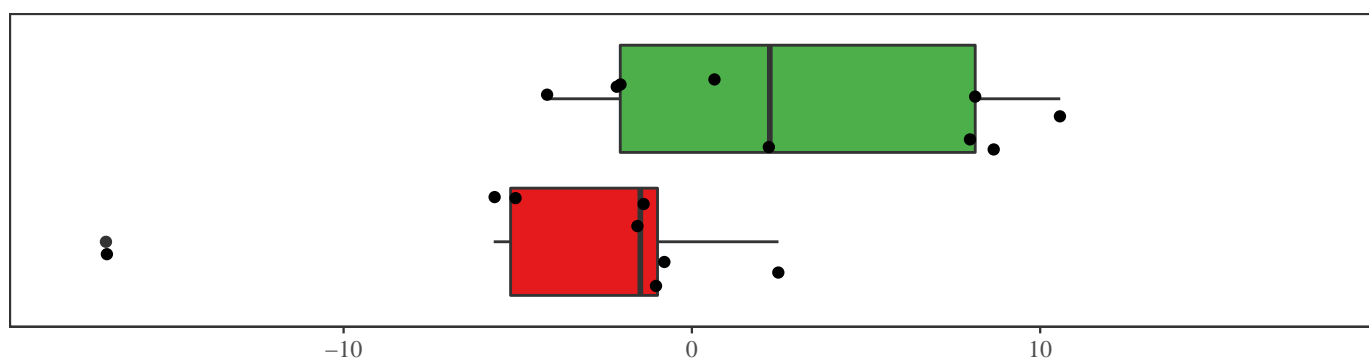

C  
D

Supplement: Supplementary file 2 [file DataSheet1.zip › Supplementary File 2/Treatment/C_vs_D/03_PLS_DA/PLSDA_Score_with_Boxplot_with_Points.pdf]

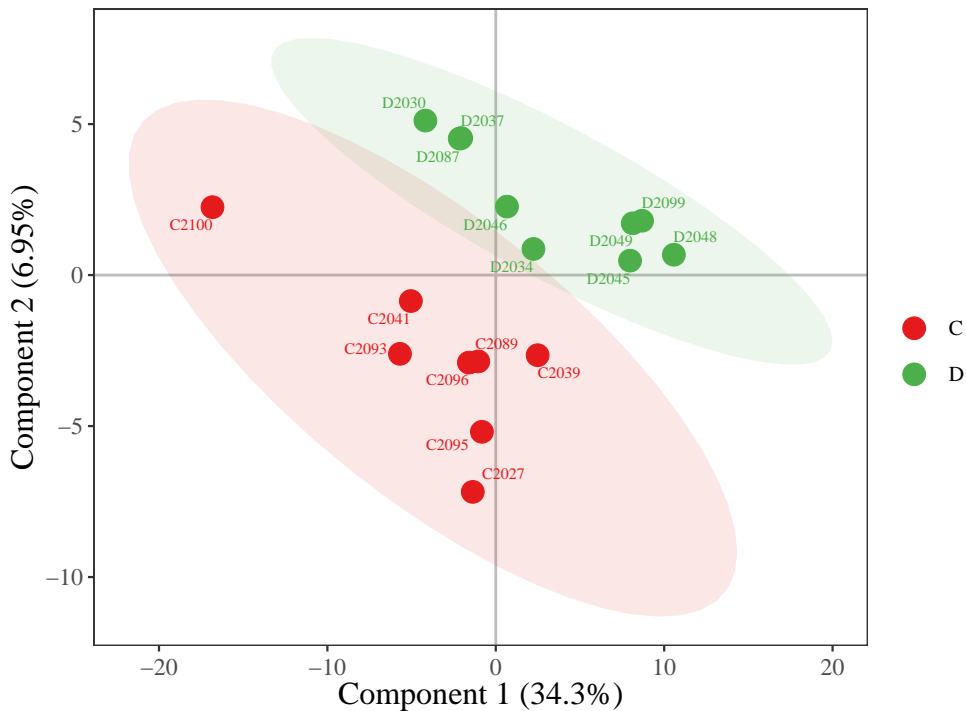

Supplement: Supplementary file 2 [file DataSheet1.zip › Supplementary File 2/Treatment/C_vs_D/03_PLS_DA/PLSDA_Score_2D_Label.pdf]

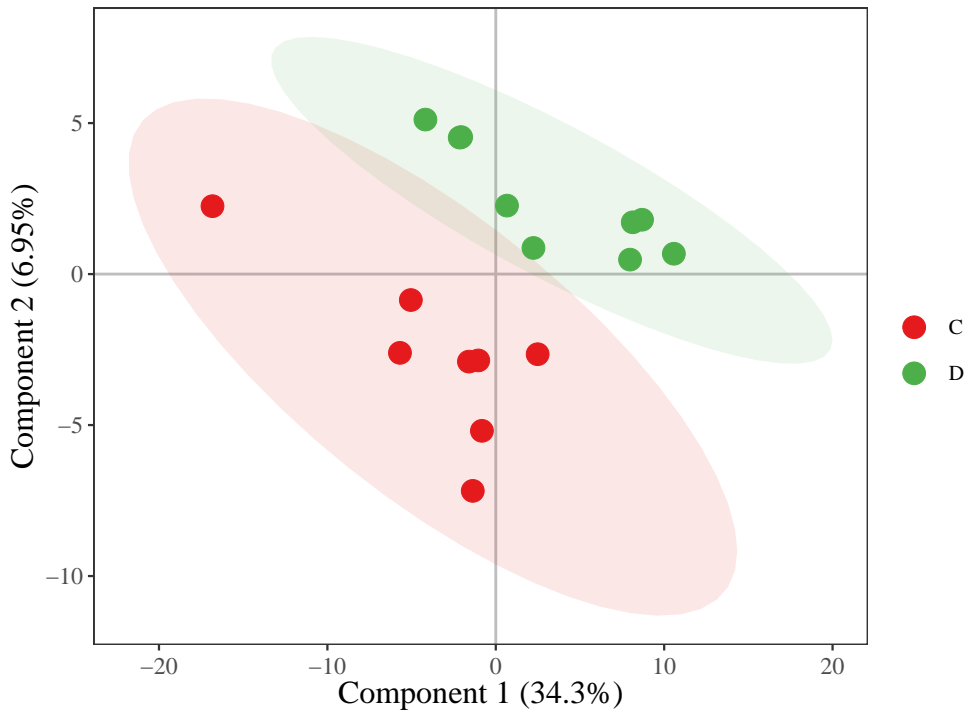

Supplement: Supplementary file 2 [file DataSheet1.zip › Supplementary File 2/Treatment/C_vs_D/03_PLS_DA/PLSDA_Score_2D.pdf]

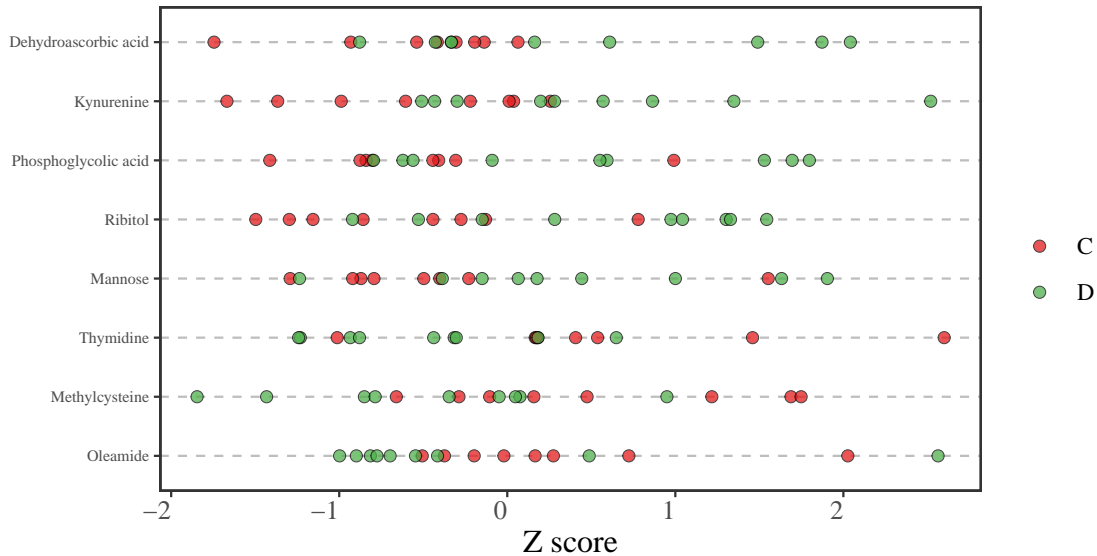

Supplement: Supplementary file 2 [file DataSheet1.zip › Supplementary File 2/Treatment/C_vs_D/06_Potential_Biomarkers/Markers_Z_Score_Plot.pdf]

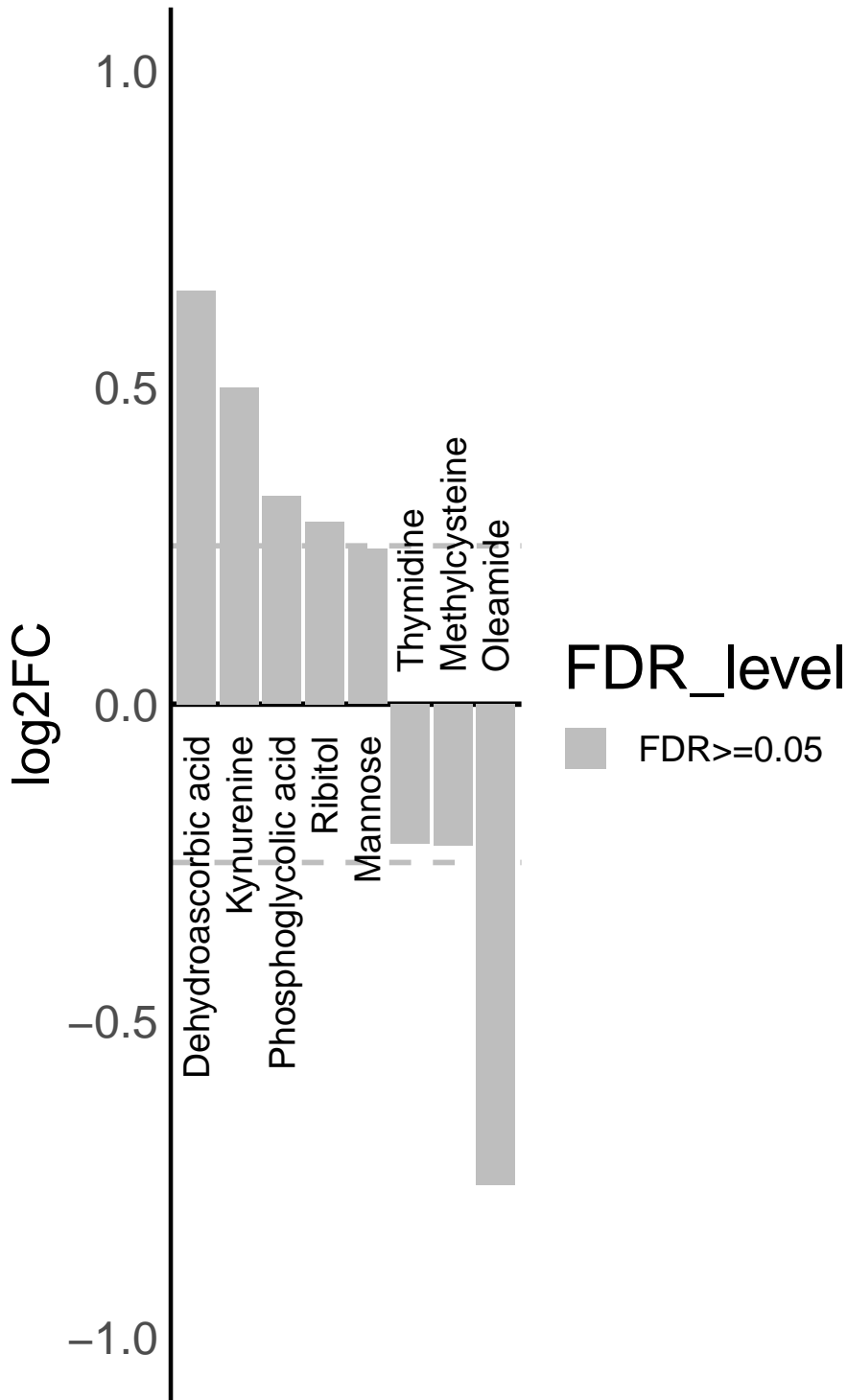

Supplement: Supplementary file 2 [file DataSheet1.zip › Supplementary File 2/Treatment/C_vs_D/06_Potential_Biomarkers/Markers_log2FC_FDR_Bar_Plot.pdf]

Univariate

OPLS-DA

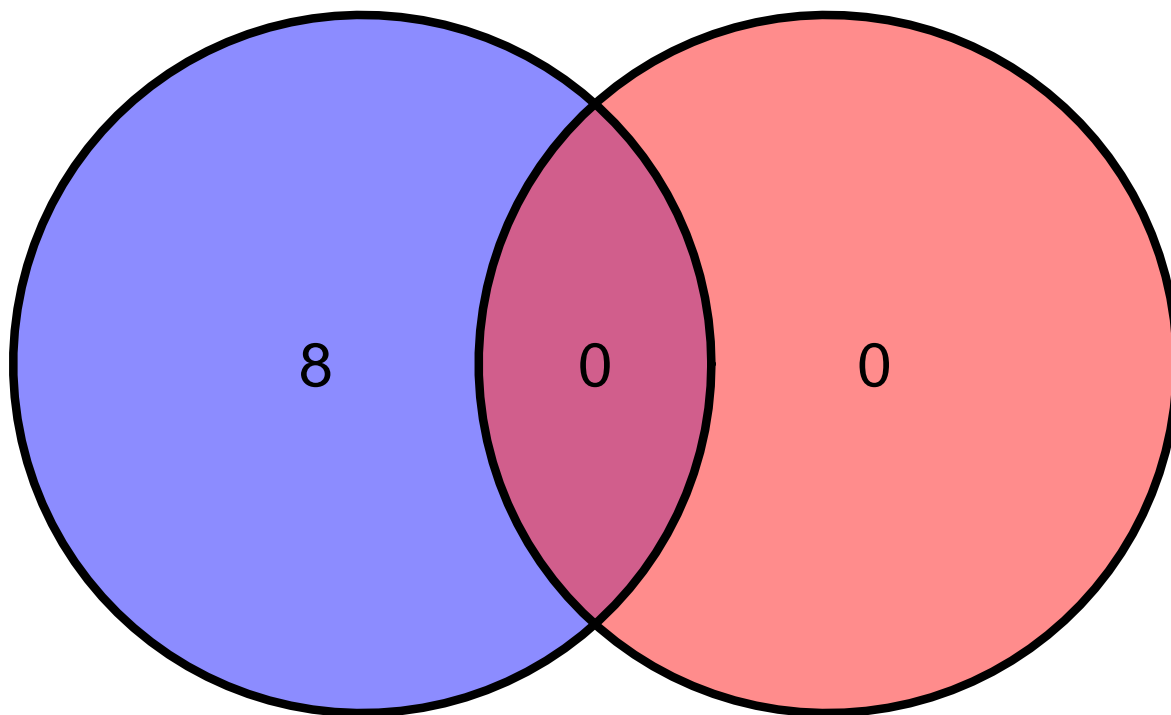

Supplement: Supplementary file 2 [file DataSheet1.zip › Supplementary File 2/Treatment/C_vs_D/06_Potential_Biomarkers/Venn_Plot.pdf]

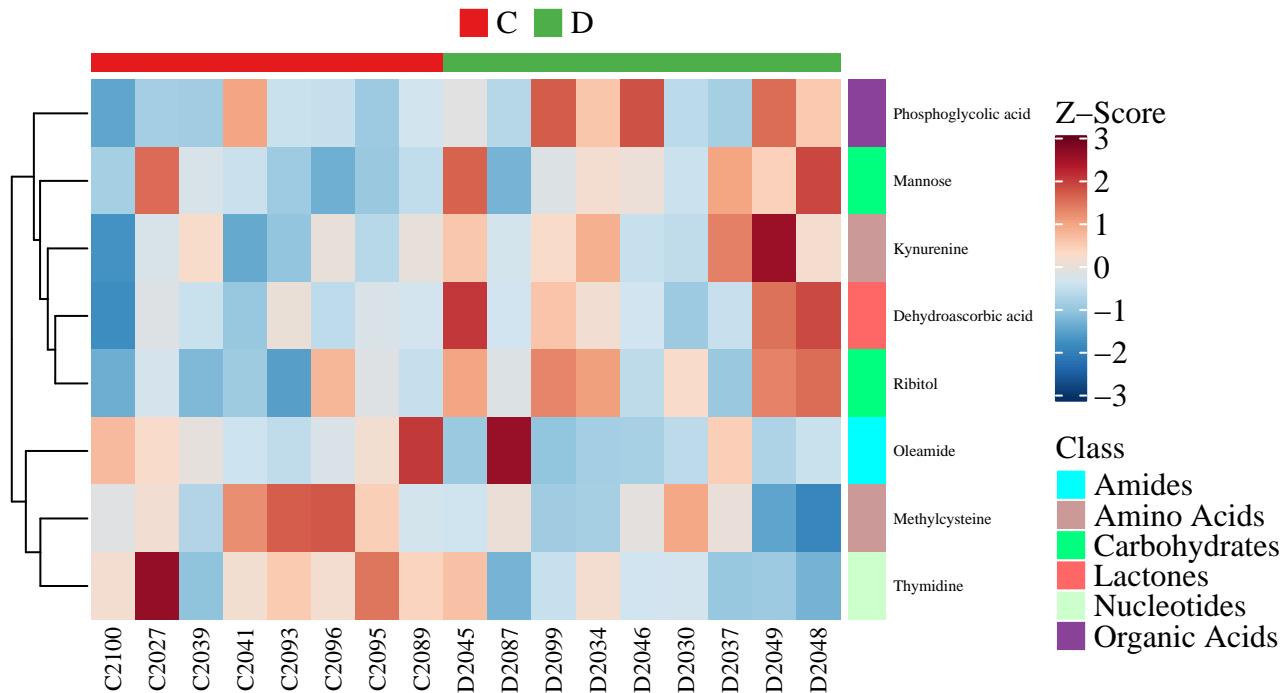

Supplement: Supplementary file 2 [file DataSheet1.zip › Supplementary File 2/Treatment/C_vs_D/06_Potential_Biomarkers/Markers_Z_Score_Heatmap_with_Name.pdf]

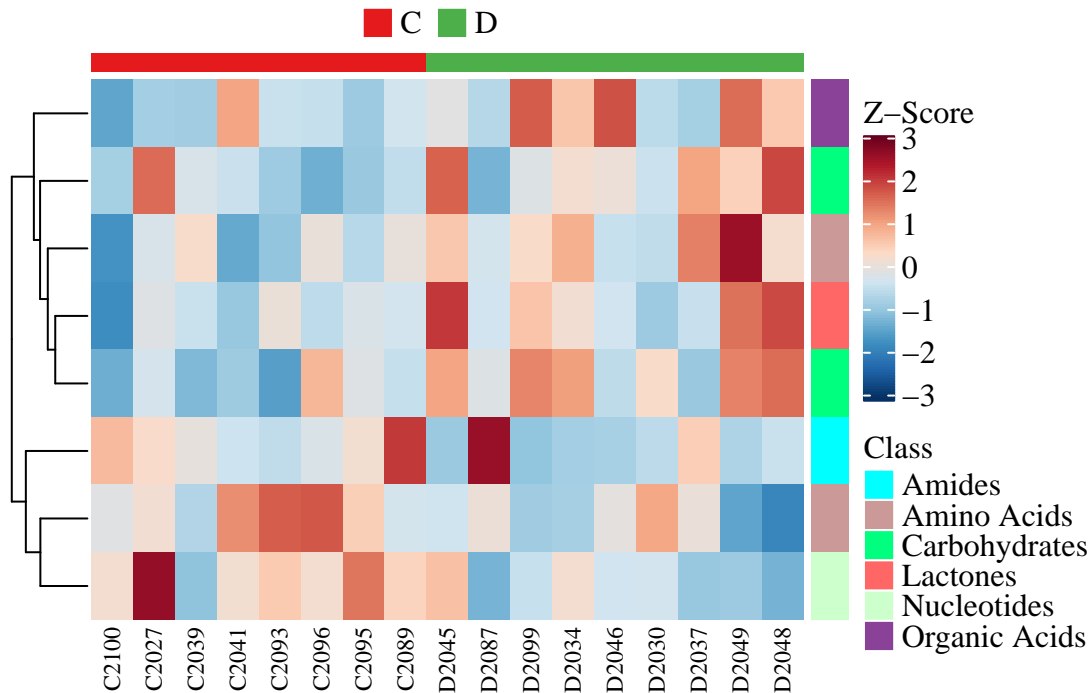

Supplement: Supplementary file 2 [file DataSheet1.zip › Supplementary File 2/Treatment/C_vs_D/06_Potential_Biomarkers/Markers_Z_Score_Heatmap.pdf]

C D

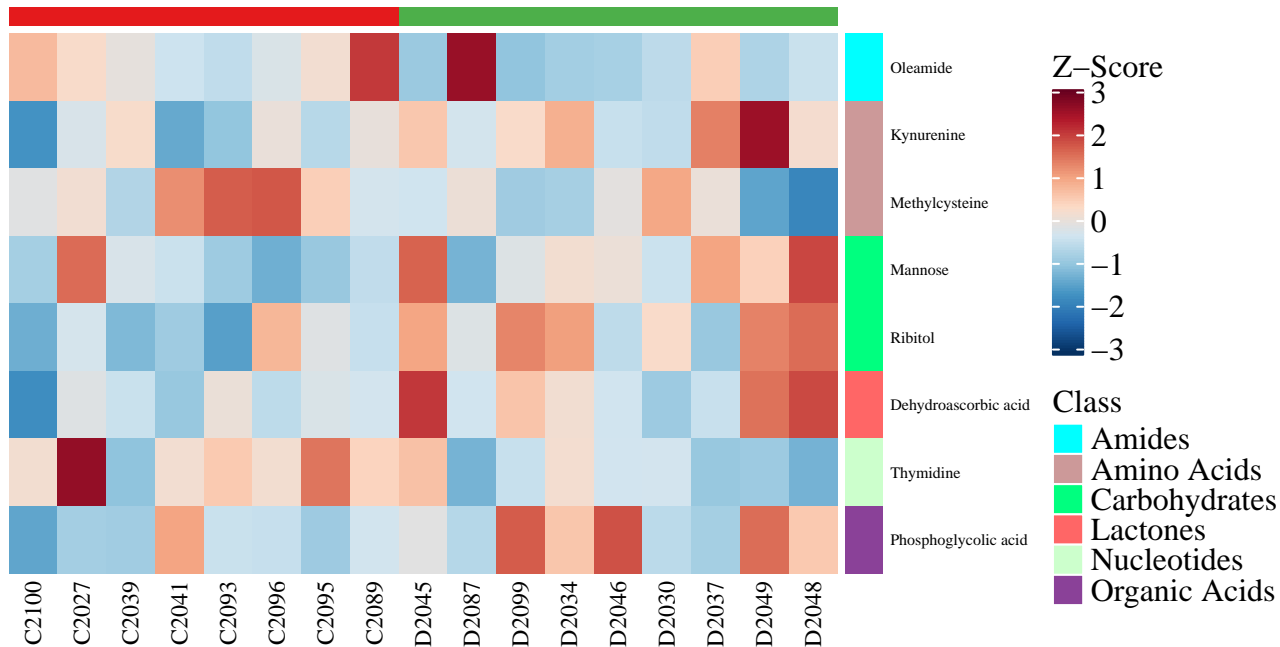

Supplement: Supplementary file 2 [file DataSheet1.zip › Supplementary File 2/Treatment/C_vs_D/06_Potential_Biomarkers/Markers_Z_Score_Heatmap_ordered_by_Class_with_Name.pdf]

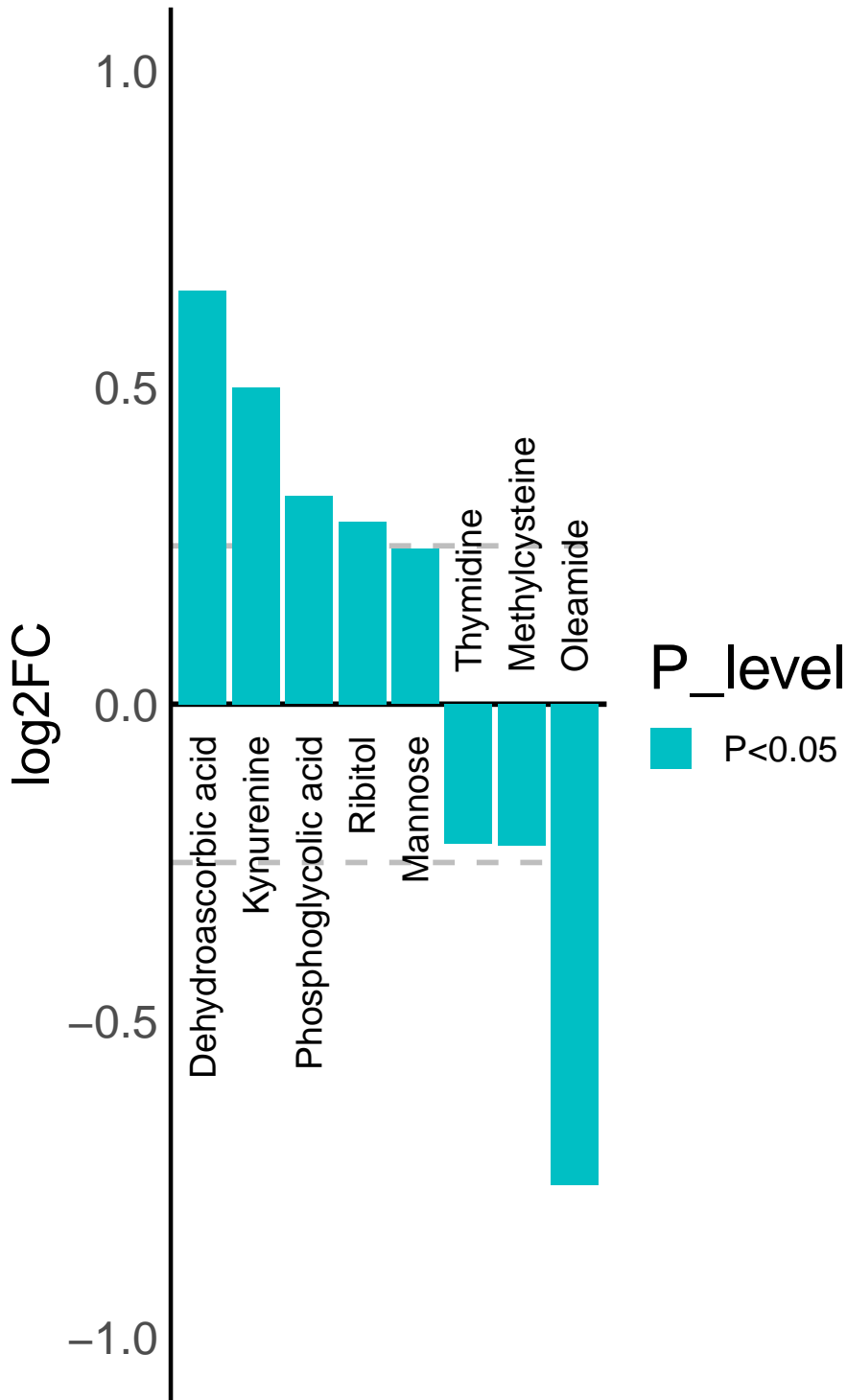

Supplement: Supplementary file 2 [file DataSheet1.zip › Supplementary File 2/Treatment/C_vs_D/06_Potential_Biomarkers/Markers_log2FC_P_Bar_Plot.pdf]

O1 (33.2%)

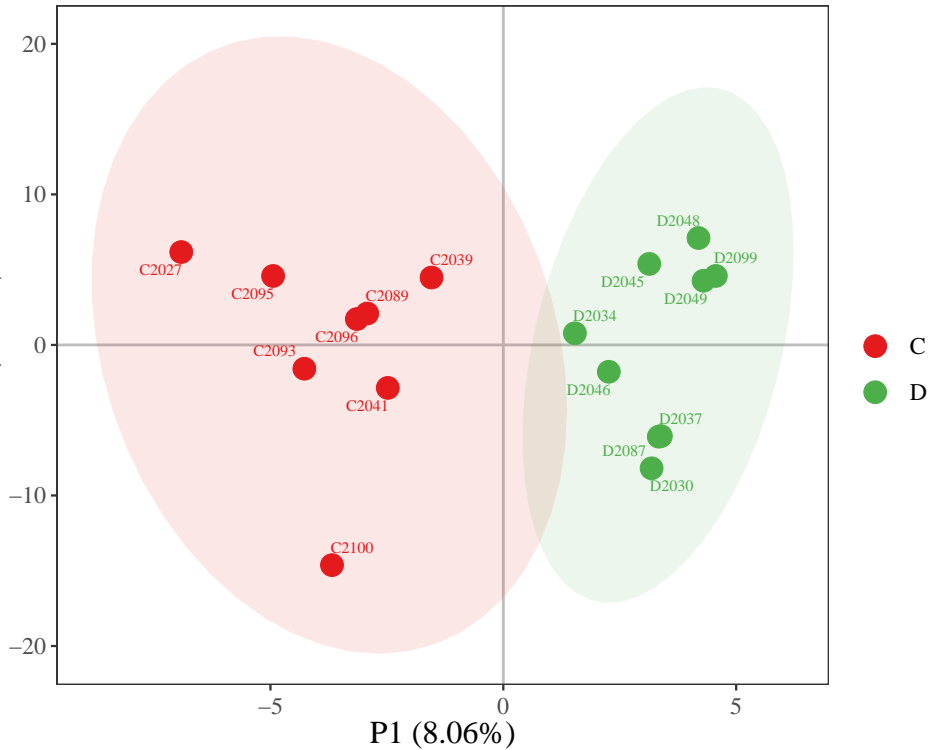

Supplement: Supplementary file 2 [file DataSheet1.zip › Supplementary File 2/Treatment/C_vs_D/04_OPLS_DA/OPLSDA_Score_2D_Label.pdf]

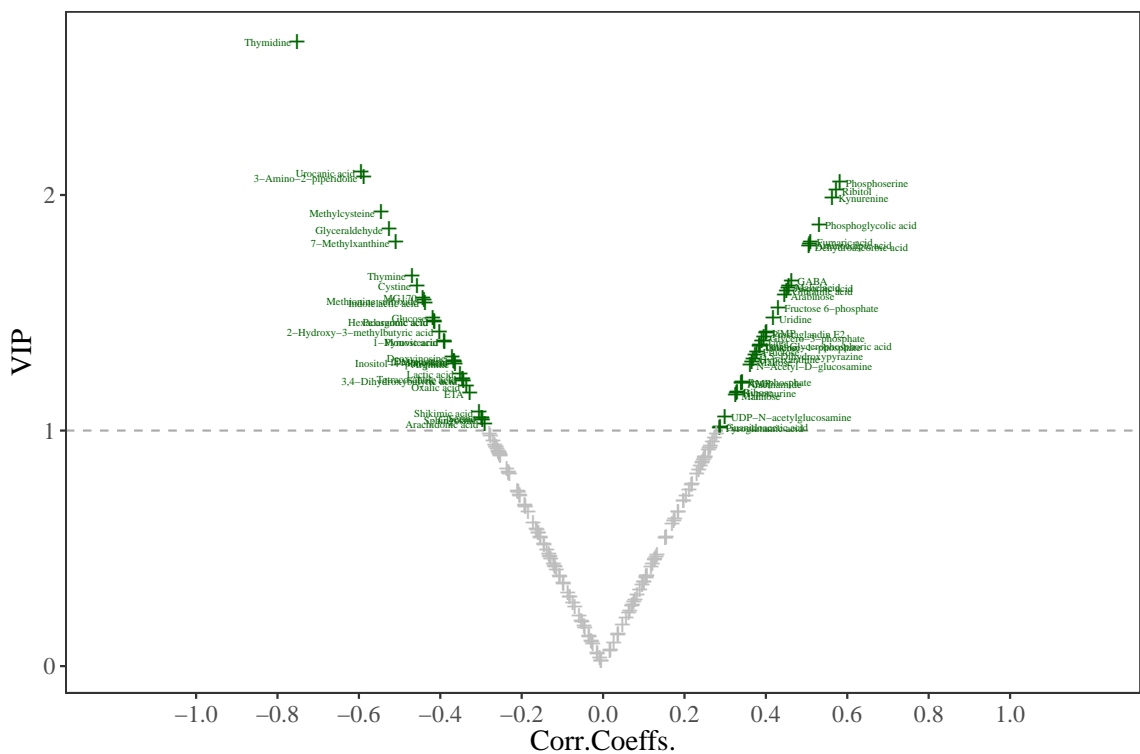

Supplement: Supplementary file 2 [file DataSheet1.zip › Supplementary File 2/Treatment/C_vs_D/04_OPLS_DA/OPLSDA_VPlot.pdf]

O1 (33.2%)

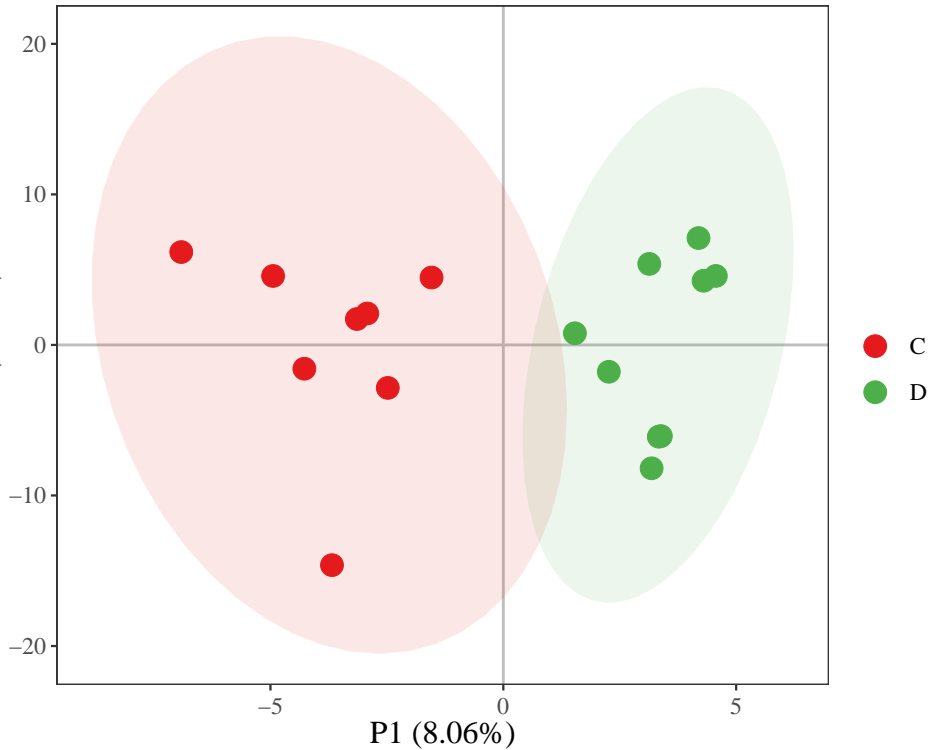

Supplement: Supplementary file 2 [file DataSheet1.zip › Supplementary File 2/Treatment/C_vs_D/04_OPLS_DA/OPLSDA_Score_2D.pdf]

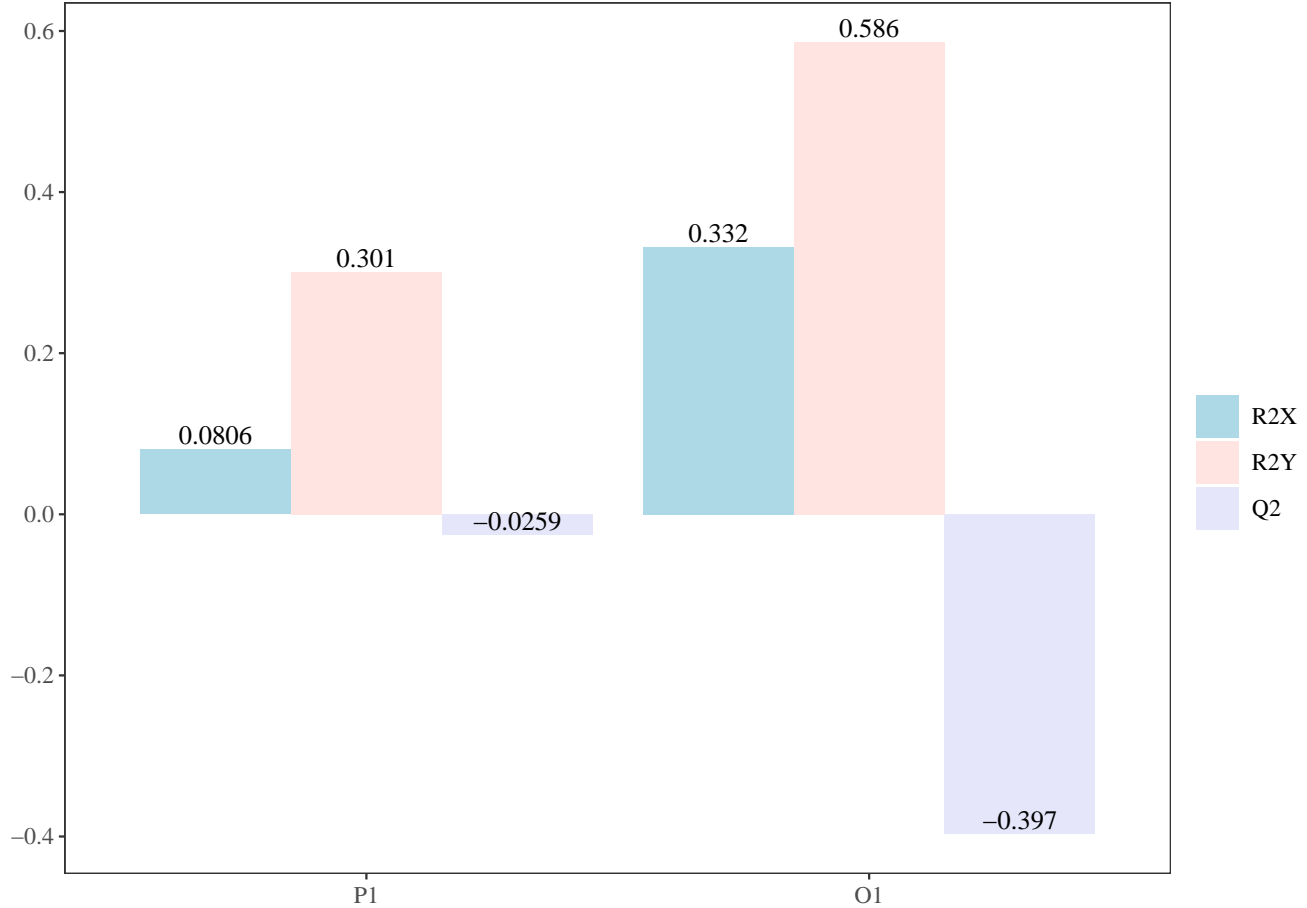

Supplement: Supplementary file 2 [file DataSheet1.zip › Supplementary File 2/Treatment/C_vs_D/04_OPLS_DA/OPLSDA_R2X_R2Y_Q2.pdf]

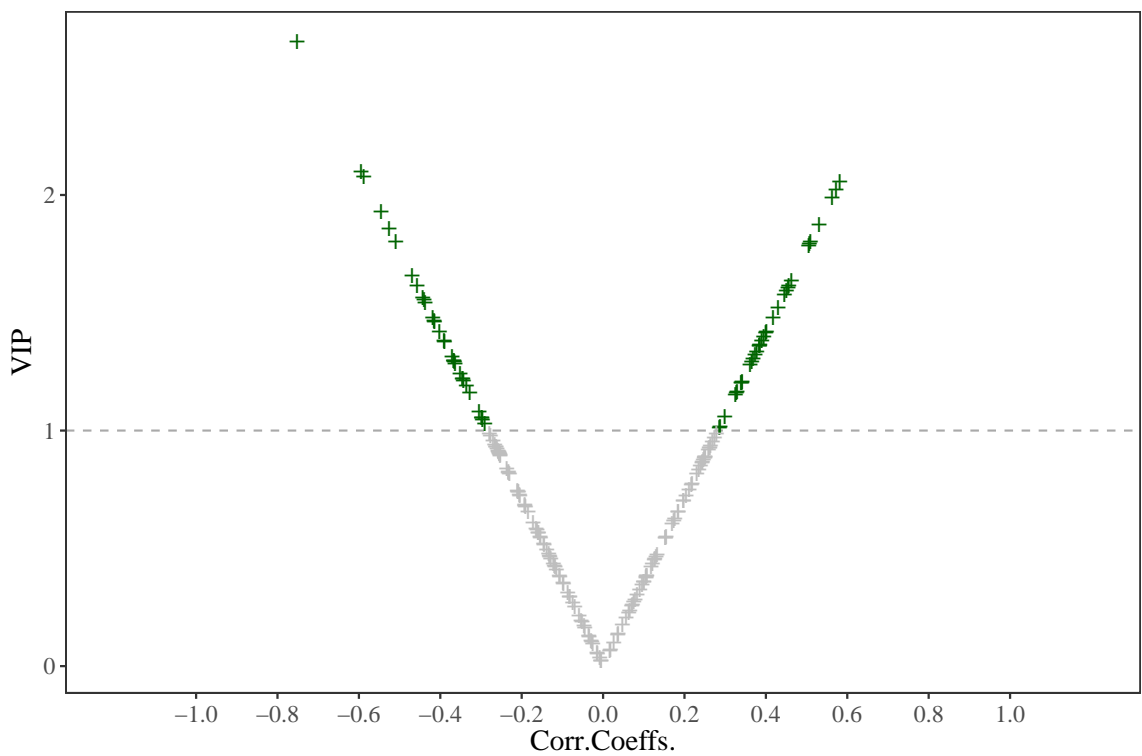

Supplement: Supplementary file 2 [file DataSheet1.zip › Supplementary File 2/Treatment/C_vs_D/04_OPLS_DA/OPLSDA_VPlot_without_Label.pdf]

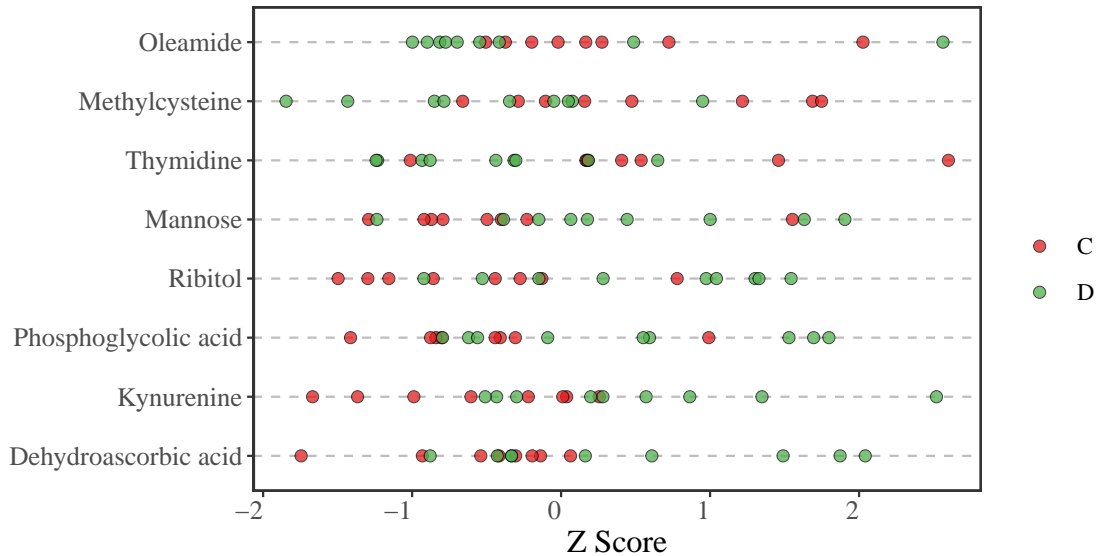

Supplement: Supplementary file 2 [file DataSheet1.zip › Supplementary File 2/Treatment/C_vs_D/05_Univariate_Analysis/Z_Score_Plot.pdf]

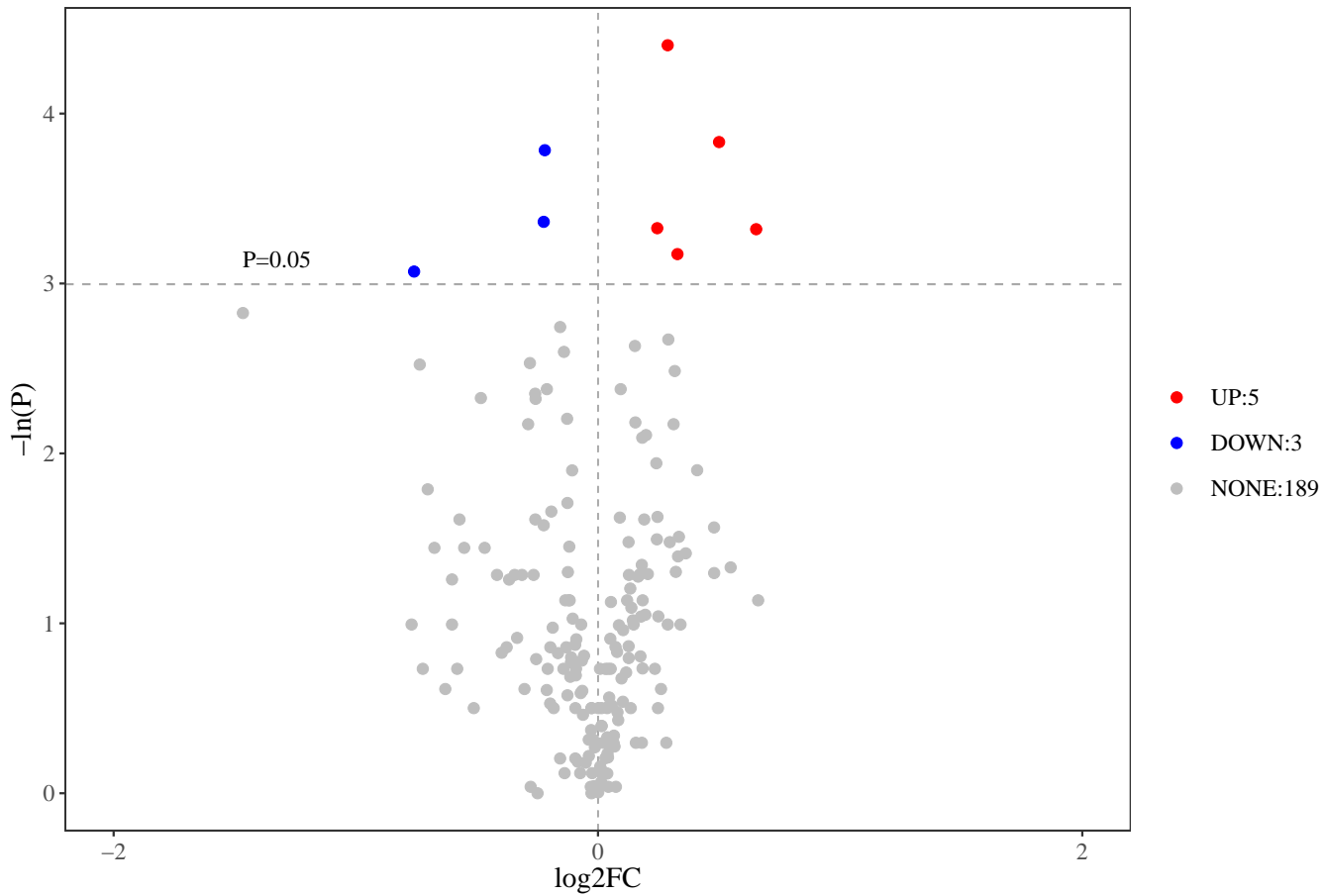

Supplement: Supplementary file 2 [file DataSheet1.zip › Supplementary File 2/Treatment/C_vs_D/05_Univariate_Analysis/Volcano_Plot.pdf]

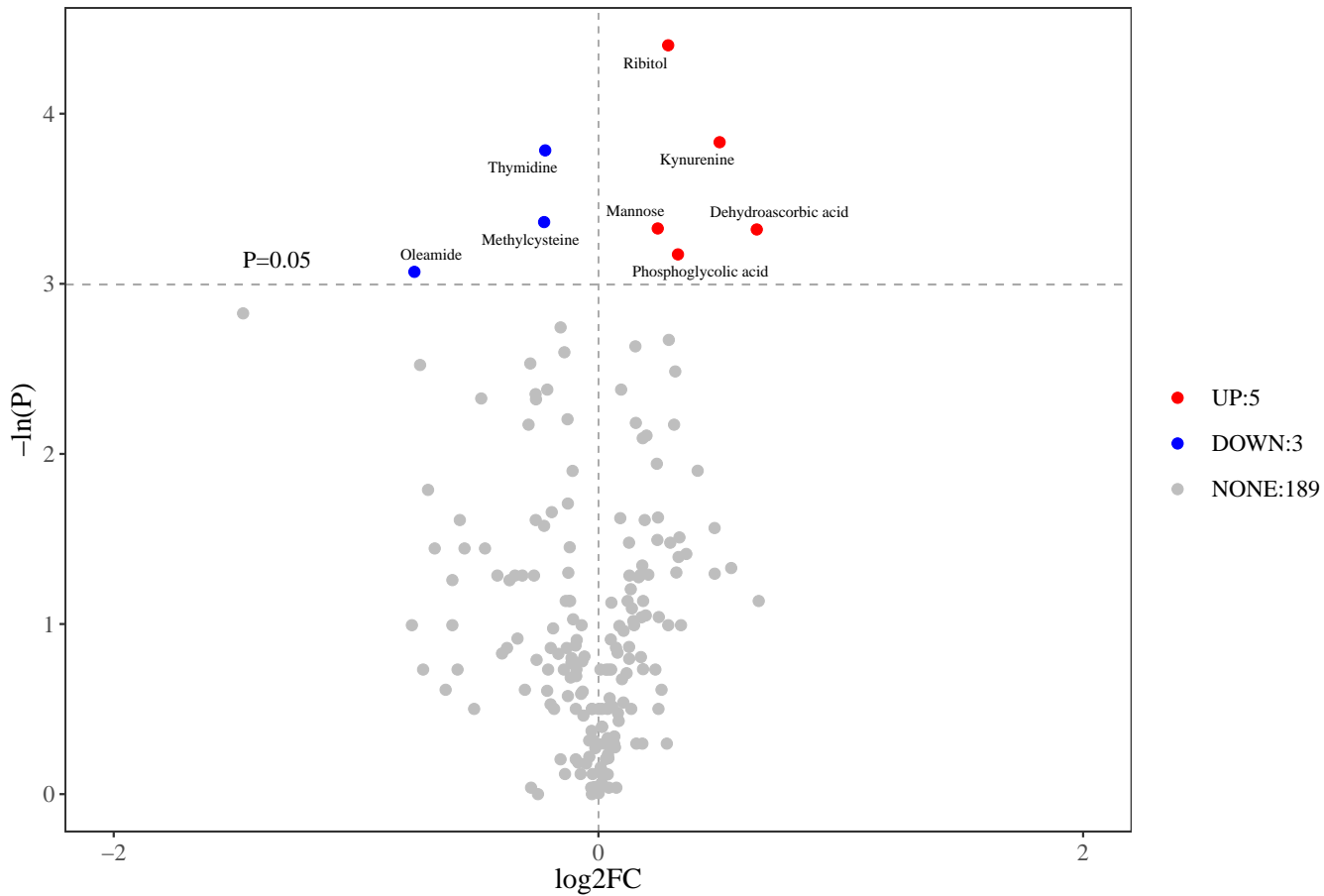

Supplement: Supplementary file 2 [file DataSheet1.zip › Supplementary File 2/Treatment/C_vs_D/05_Univariate_Analysis/Volcano_Plot_with_Label.pdf]

■ C ■ D

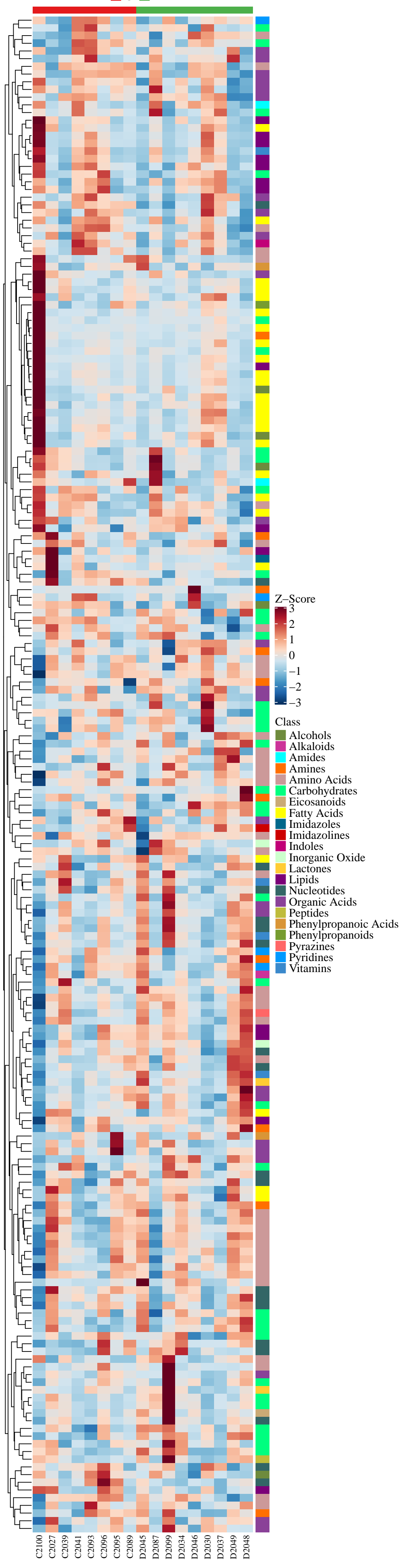

Supplement: Supplementary file 2 [file DataSheet1.zip › Supplementary File 2/Treatment/C_vs_D/01_Basic_Statistics/Z_Score_Heatmap.pdf]

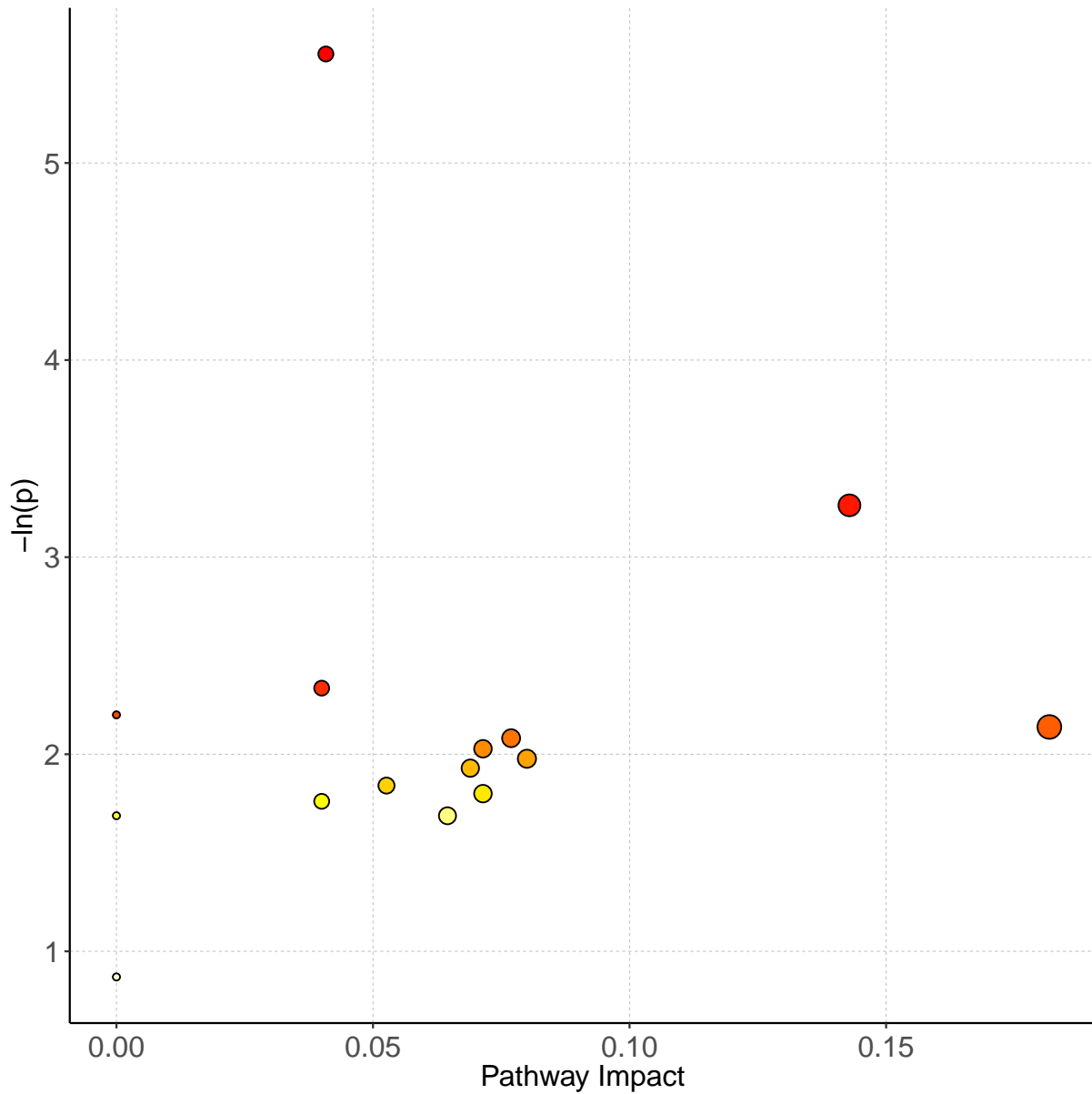

Supplement: Supplementary file 2 [file DataSheet1.zip › Supplementary File 2/Treatment/D_vs_H/07_Pathway_Analysis/Pathway_Analysis/Pathway_Bubbleplot_without_label.pdf]

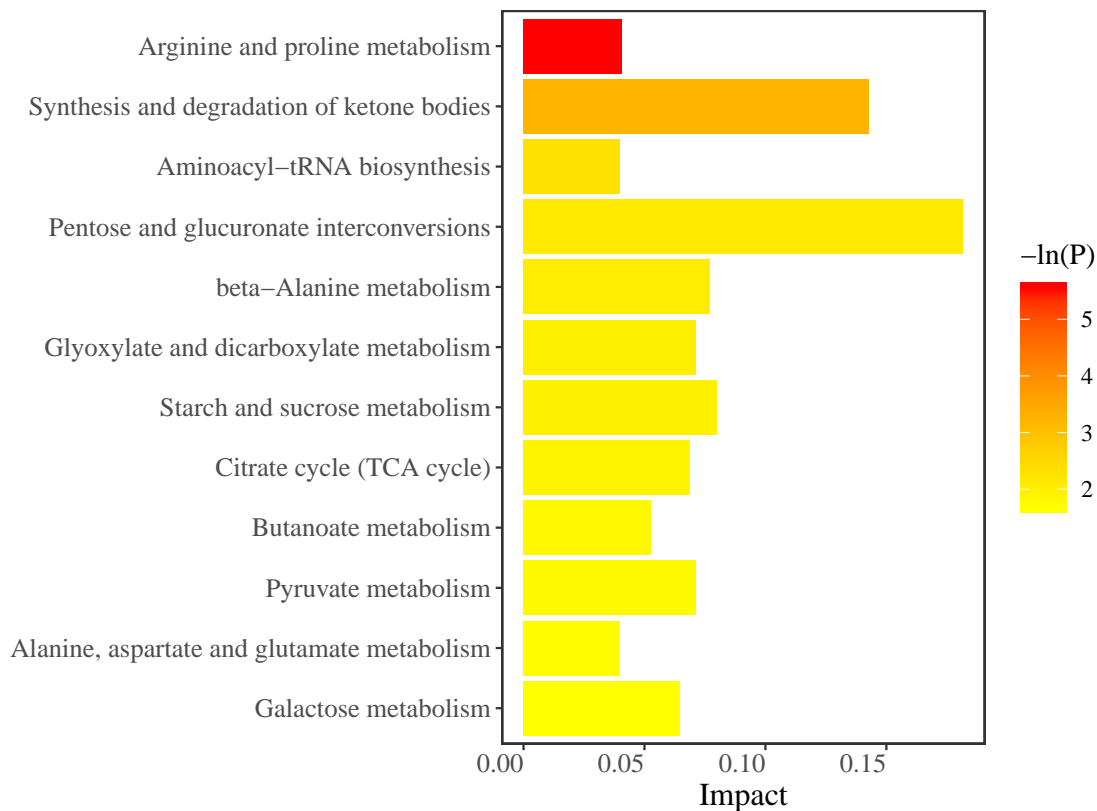

Supplement: Supplementary file 2 [file DataSheet1.zip › Supplementary File 2/Treatment/D_vs_H/07_Pathway_Analysis/Pathway_Analysis/Pathway_Barplot.pdf]

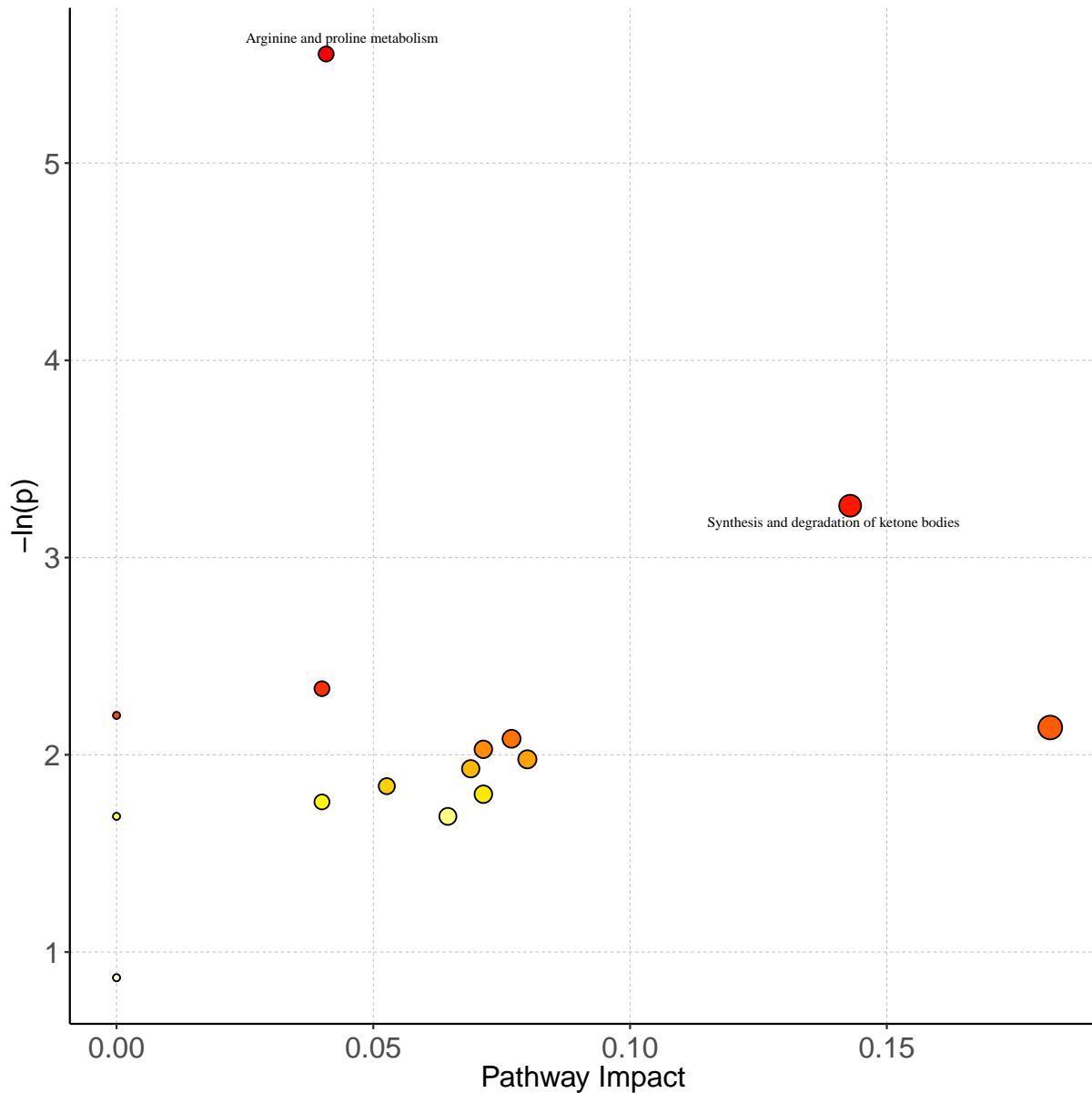

Supplement: Supplementary file 2 [file DataSheet1.zip › Supplementary File 2/Treatment/D_vs_H/07_Pathway_Analysis/Pathway_Analysis/Pathway_Bubbleplot.pdf]

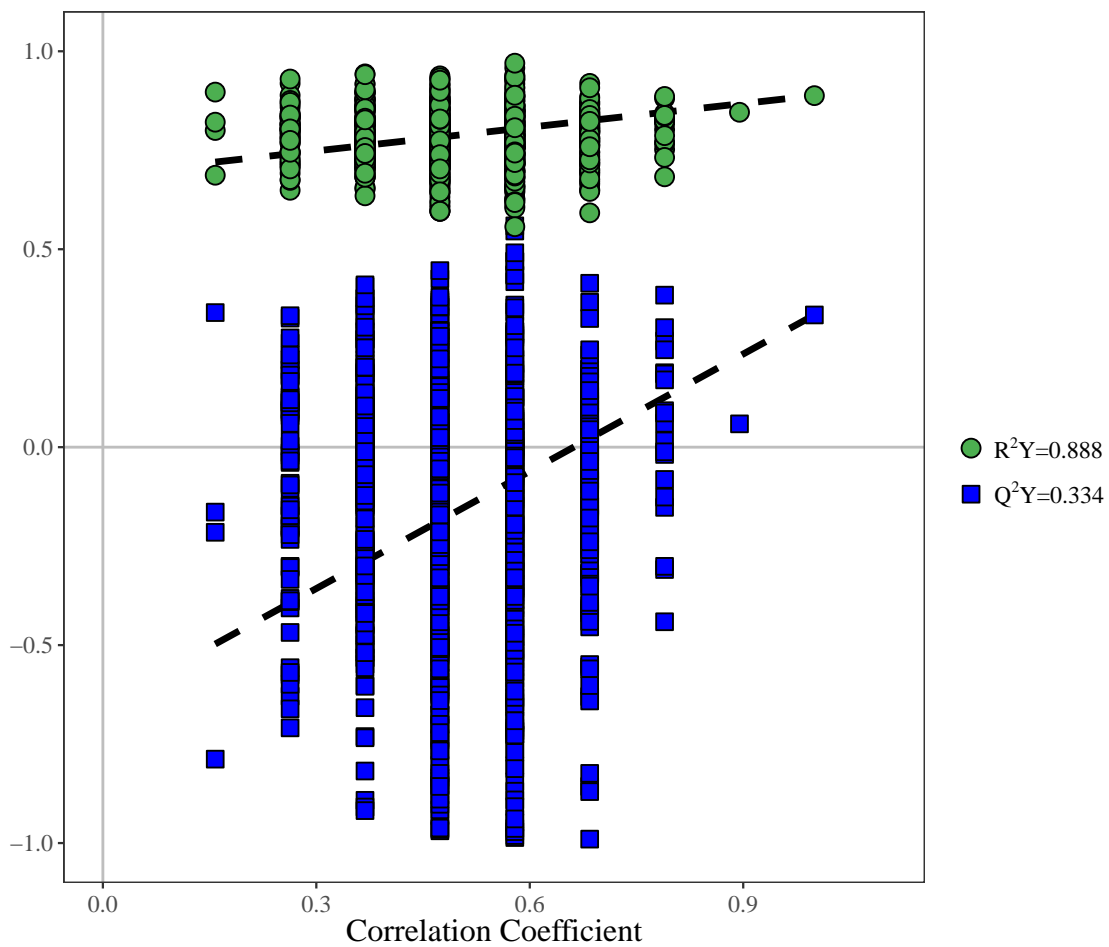

Supplement: Supplementary file 2 [file DataSheet1.zip › Supplementary File 2/Treatment/D_vs_H/04_OPLS_DA/Model_Validation/OPLSDA_Permutation.pdf]

# Scree plot

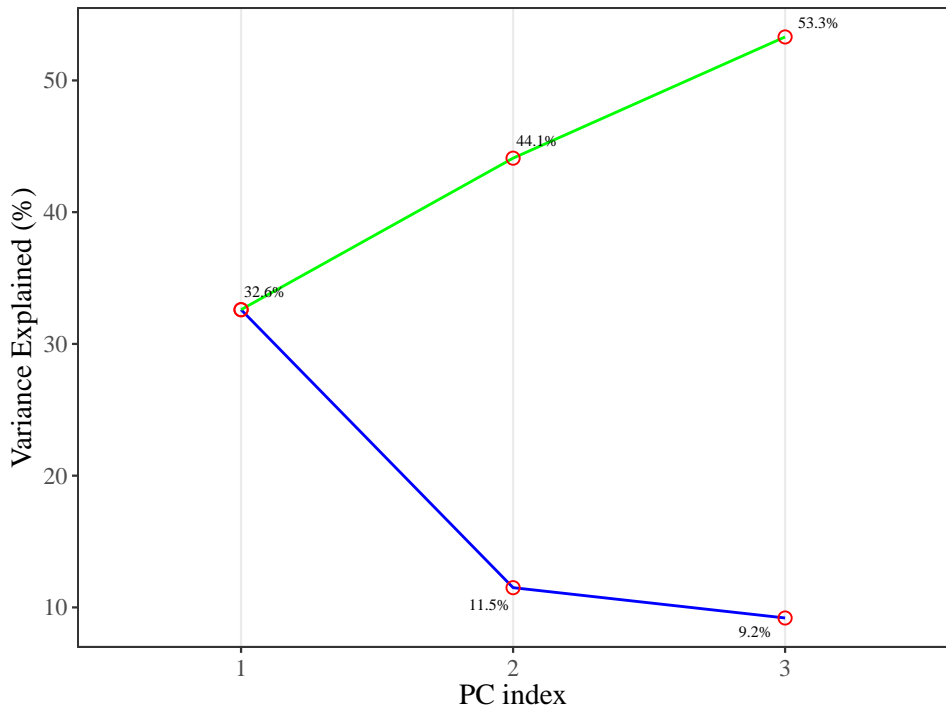

Supplement: Supplementary file 2 [file DataSheet1.zip › Supplementary File 2/Treatment/D_vs_H/02_PCA/All_Groups/PCA_Screeplot.pdf]

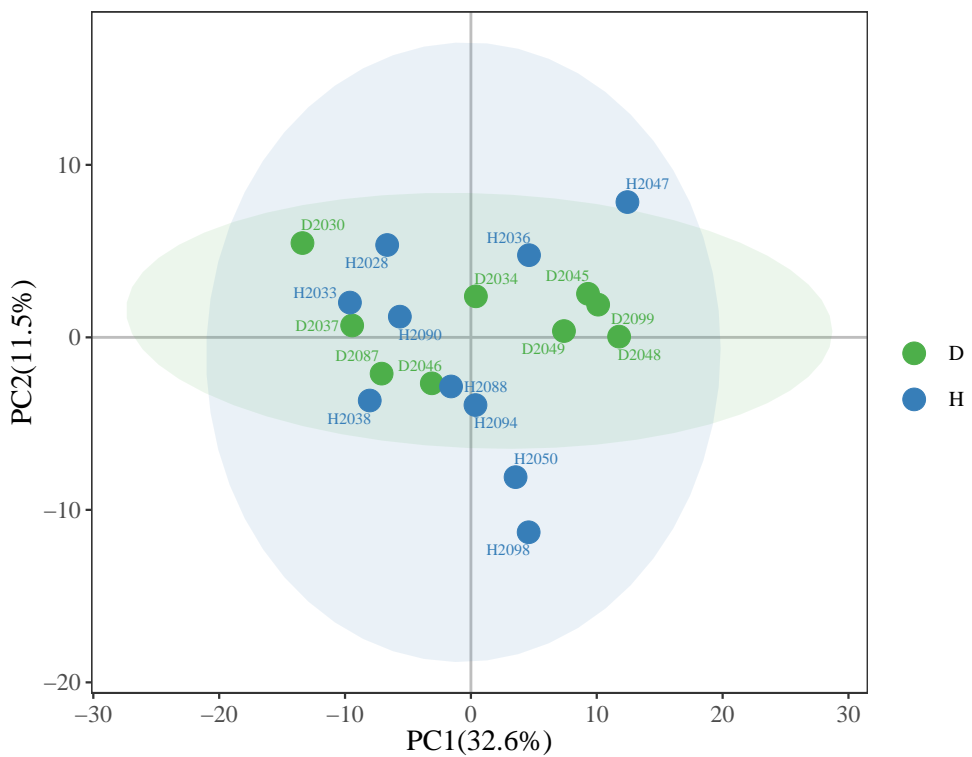

Supplement: Supplementary file 2 [file DataSheet1.zip › Supplementary File 2/Treatment/D_vs_H/02_PCA/All_Groups/PCA_Score_2D_Label.pdf]

PC3(9.2%)

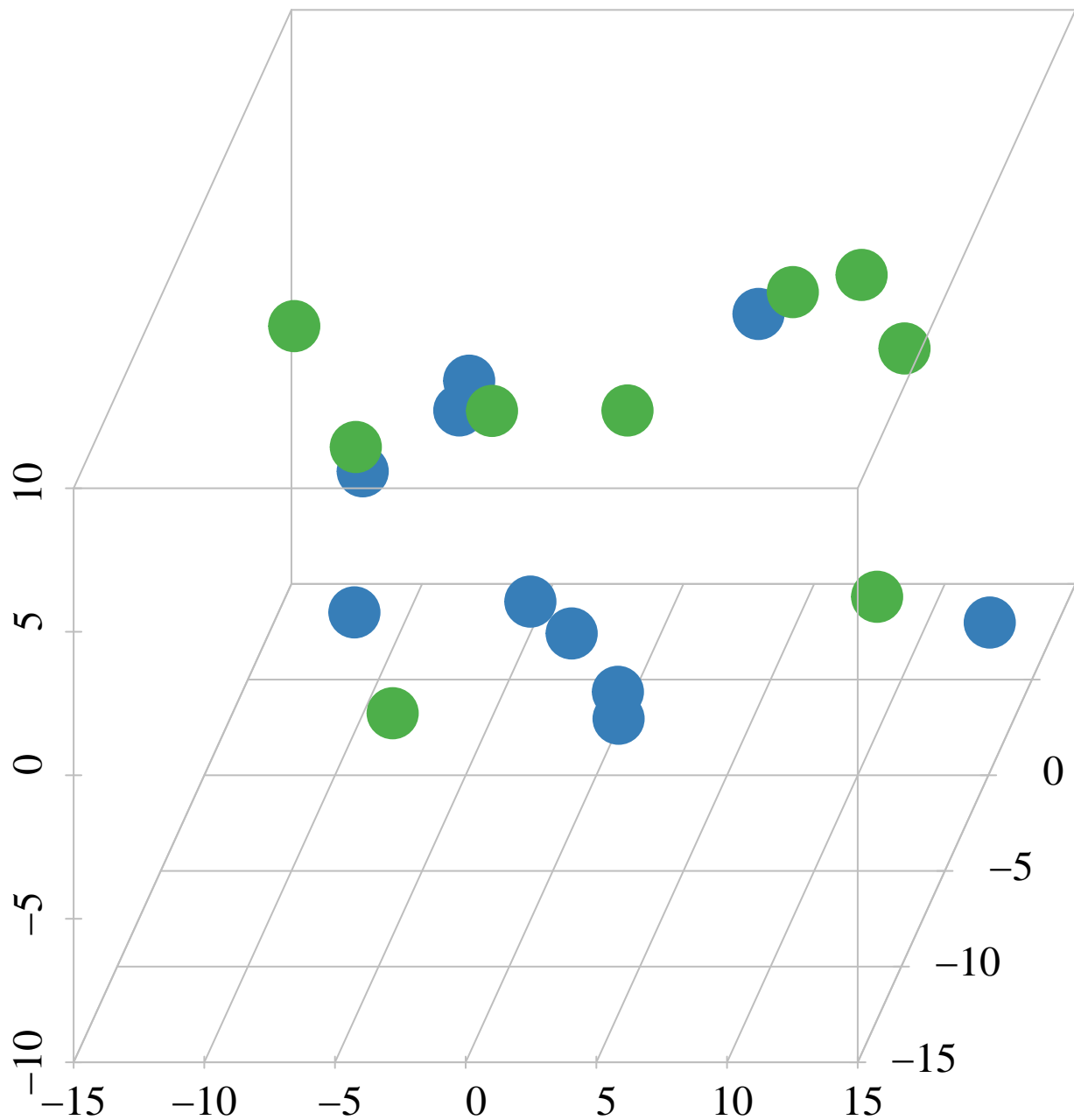

PC1(32.6%)

PC2(11.5%)

Supplement: Supplementary file 2 [file DataSheet1.zip › Supplementary File 2/Treatment/D_vs_H/02_PCA/All_Groups/PC123_Score_3D.pdf]

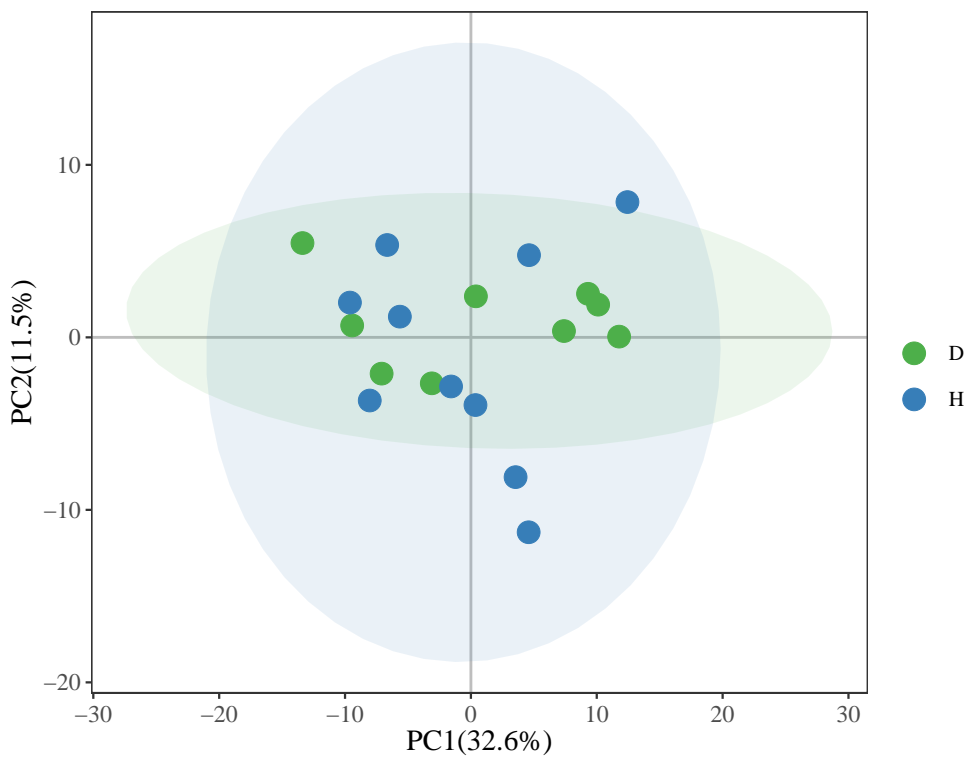

Supplement: Supplementary file 2 [file DataSheet1.zip › Supplementary File 2/Treatment/D_vs_H/02_PCA/All_Groups/PCA_Score_2D.pdf]

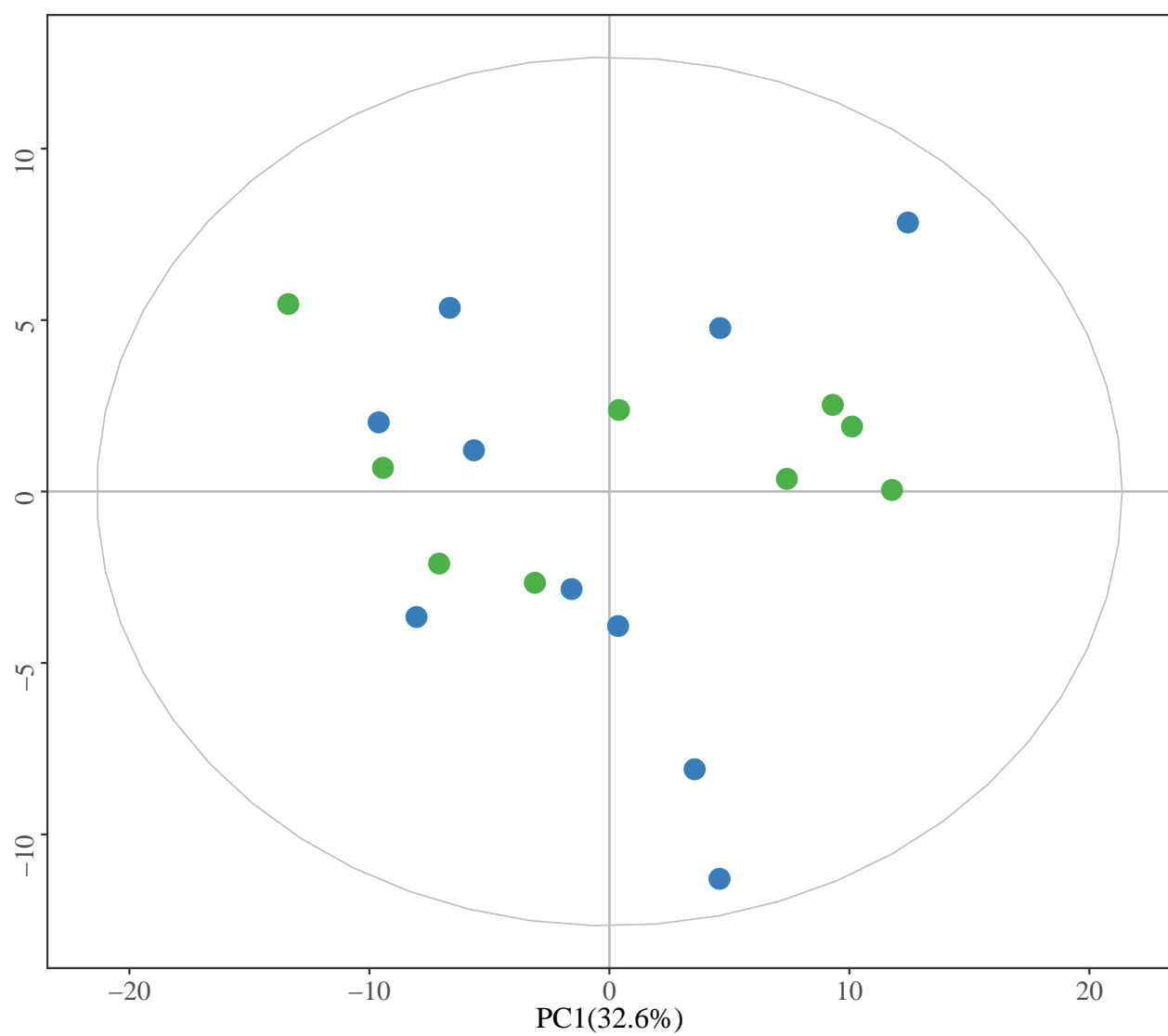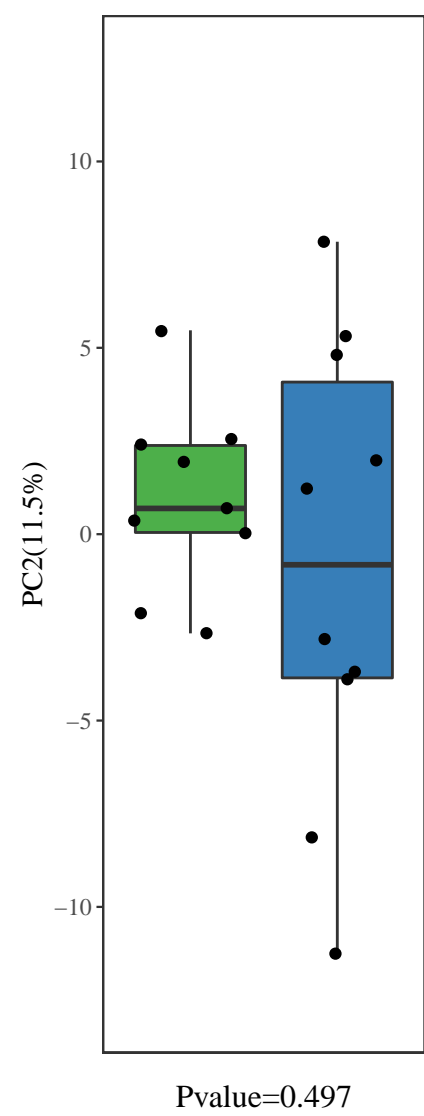

Pvalue=0.78

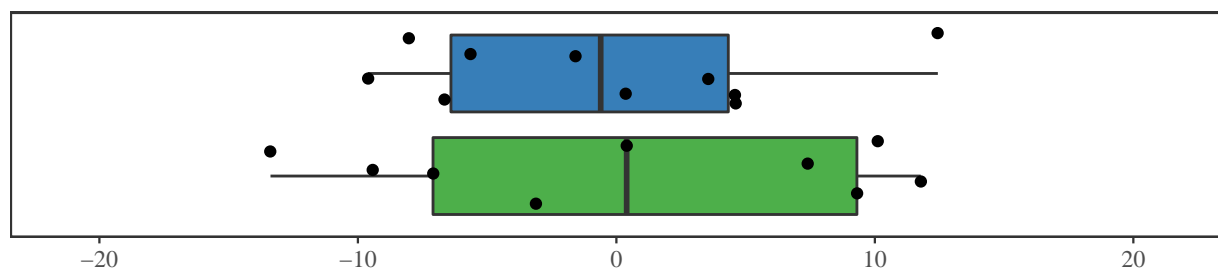

D  
H

Supplement: Supplementary file 2 [file DataSheet1.zip › Supplementary File 2/Treatment/D_vs_H/02_PCA/All_Groups/PCA_Score_with_Boxplot_with_Points.pdf]
